# Effects of patient race on processes and experiences of clinical interactions in US emergency departments: A mixed-methods systematic review

**DOI:** 10.1371/journal.pone.0325315

**Published:** 2025-06-25

**Authors:** Tommy J. Flynn, Bonnie Mowinski Jennings, David W. Wright, Ymir Vigfusson, Dian D. Evans, Vicki Hertzberg, Emily J. Burns, Katherine A. Yeager

**Affiliations:** 1 Department of Nursing, Benioff Children’s Hospital, University of California San Francisco, California, United States of America; 2 Nell Hodgson Woodruff School of Nursing, Emory University, Georgia, United States of America; 3 Department of Emergency Medicine, School of Medicine, Emory University, Georgia, United States of America; 4 Department of Computer Science, Emory University, Georgia, United States of America; Neurocrine Biosciences Inc, UNITED STATES OF AMERICA

## Abstract

**Background:**

Evidence shows that patients identified as Black experience worse emergency department (ED) care compared to patients identified as White. Clinical interactions (CI) are thought to affect racial disparities, but no systematic reviews to date have synthesized the evidence on the effects of patient race on CI processes and experiences.

**Objective:**

We synthesized evidence from published studies comparing observed and/or patient-reported CI processes and experiences between patients identified as Black and White in US EDs.

**Methods:**

This was a mixed-methods systematic review following the Joanna Briggs Institute guidelines and registered a priori with PROSPERO (CRD42021281653). We used a broad search strategy to query six databases. Peer-reviewed original research reports comparing processes or experiences of CIs between Black and White patients in US EDs from 2004 to 2024 were eligible for inclusion.

**Results:**

Nine studies met inclusion criteria. Of these, three focused on observational CI processes and six focused on patient-reported CI experiences. Evidence of differences in CI processes and patient-reported experiences between Black and White patients was inconclusive. We identified four themes across measures used in studies of patient reported CI experiences, however, including responsive nonverbal behavior, effective verbal communication, person centeredness, and patient satisfaction.

**Discussion:**

Research on the effects of patient race on ED CIs is lacking, especially research with observed real-world CI processes. Psychometrically robust instruments and conceptual clarity in the study of racial disparities in CI experiences are needed. We provide groundwork for future research development on racial disparities in ED CIs.

## Introduction

Racial healthcare disparities are unjust differences in service delivery and quality that lead to an unequal distribution of health across racial categories [1]. Researchers have described racial disparities in various emergency department (ED) processes, including race-based differences in wait times [2–4], triage acuity scores [5–7], pain assessments and treatments [8], diagnostic imaging [9], and clinical decisions [10,11]. Racial disparities in health and healthcare are increasingly recognized problems in the US and have been key priorities of National Institutes of Health (NIH) and National Academy of Medicine (NAM) research agendas. Minority health and health disparities represent one of five crosscutting themes that span all aspects of the NIH-Wide Strategic Plan [12]. Contributing to more equitable healthcare systems is central to NAM’s report *Future of Nursing 2020–2030: Charting a Path to Achieve Health Equity* [13].

The ED is unlike other settings in the US healthcare system. In the decades following World War II, EDs became healthcare access points for medically underserved and racially marginalized populations [14,15]. The Emergency Medical Treatment and Active Labor Act (EMTALA) of 1986 paved the way for substantial increases in ED use [16,17]. Today, the ED is a cornerstone of US healthcare that continues to provide care to millions of low-income and uninsured patients [18], offering 24-hour access to hospital-based resources [19]. In 2018, 21.3% of the US population accessed healthcare via an ED for a total of about 130 million visits [20,21]. Though most ED patients across the US are White, Black patients present to EDs more than twice as often as any other racial or ethnic group in the US [22].

Racial differences in ED use have been associated with social factors [23] but the causes for these differences remain speculative. Some evidence suggests the ED offers a unique healthcare environment that appeals particularly to people with histories and experiences of healthcare-related inequity. Brown et al. [24] surveyed a cross-section of adult ED patients to evaluate the effect of race, insurance status, age, and socioeconomic factors on patients’ preferred sources for routine healthcare. They reported that Black patients were more than twice as likely as White patients (OR 2.24, 95% CI [1.22, 4.08]) to prefer the ED as a usual source of care over other healthcare settings [24]. Other investigators have provided additional evidence suggesting that Black patients are more likely than White patients to prefer EDs as the first point of care [25–29]. A proposed reason for this preference is that the processes and patient experiences of face-to-face clinician-patient encounters in the ED may involve fewer relational and social forces that are so important in other healthcare contexts. Primary care settings, for example, differ from EDs in their reliance on preexisting patient-clinician relationships to achieve preferred clinical outcomes [30,31]. ED services, however, rely on more rigid temporal quality metrics and use more standardized clinical pathways, decision aids, and treatment guidelines that may work to limit relationally or socially derived variations in racially discordant clinical interactions [32–34].

Experiences and processes of clinical interactions (CIs) are extensively researched and represent one of the most commonly cited determinants of patient satisfaction in the ED literature [35–39]. CIs are face-to-face interpersonal events that occur in a healthcare context and are considered one pathway through which social and cultural forces perpetuate racial healthcare disparities [40]. Examining CIs remains a central phenomenon in research aimed at promoting health and healthcare equity in a range of healthcare settings [41,42].

In the past twenty years, substantial progress in research methods has helped advance understanding of the effects of racial identity on processes and experiences of CIs [43–45]. Researchers have studied CIs using two primary approaches to data generation: observing CI processes and surveying patients about their CI experiences. Observable *CI processes* are measurable interpersonal phenomena including cultural, social, temporal, environmental, cognitive, and behavioral dimensions that are thought to affect social behaviors and perceptions [46]. Researchers have studied observed CI processes using various qualitative and quantitative methodologies [47,48]. Patient-reported *CI experiences*, by contrast, refer to the same interpersonal phenomena yet the focus is on how the interpersonal phenomena are perceived by the people involved [49]. CI experiences research focuses on patient-perceived quality of clinicians’ behaviors and interpersonal dimensions including communication, shared decision-making, and empathy.

Evidence of racial disparities in CI processes and experiences has been reported across various healthcare settings [50,51]. To our knowledge, however, there have been no systematic reviews to date that provide a synthesis of evidence pertaining to CIs and racial disparities in US EDs. Our goal is to fill that gap to help inform and guide future ED disparities research. The purpose of this study, therefore, was to synthesize the evidence from published studies comparing observed and/or patient-reported CI processes and experiences between patients identified as Black or White in US EDs.

## Methods

A mixed-methods systematic review was conducted per the guidelines of the Joanna Briggs Institute [52] and reported according to Preferred Reporting Items for Systematic Reviews and Meta-Analyses recommendations [PRISMA-2020; [53,54]]. We chose to conduct a mixed-methods systematic review (MMSR) because they are ideal for understanding complex healthcare phenomena [55]. A research protocol was registered a priori with PROSPERO (CRD42021281653). We used EndNote (Clarivate, version 20) for citation management and Covidence systematic review software (Veritas Health Innovation, Melbourne, Australia. Available at www.covidence.org) for all elements of the screening, quality assessment, and data extraction processes.

### Information sources

PubMed/Medline (National Library of Medicine), Web of Science Core Collection (Clarivate), Embase (Elsevier), PsycInfo (EBSCO*host*), and the Cumulative Index to Nursing and Allied Health Literature (CINAHL, EBSCO*host*) were queried to ensure maximum coverage of literature from diverse disciplines [56]. We ran initial queries in October of 2021 and final queries in January of 2024. We used EndNote and Scopus (Elsevier B.V., 2023) to import records from the reference lists of included studies following the methods described by Bramer [57].

### Search strategy

We started by organizing key concepts from the research question into a modified population, intervention, comparison, outcomes, setting (i.e., PICOS) framework [58]. An information librarian with search strategy expertise collaborated with the review team to develop a search strategy based on the keywords. Various sources influenced our selection of terms, including: the “Medline®/PubMed® Health Disparities and Minority Health Search Strategy” [59]; the equity-focused strategy from Prady et al. [60]; and published research on ED healthcare disparities [61], clinical interactions [47,62–64], and ED patient experience [36,37,65]. We included an expansive list of associated search terms to maximize the volume of potentially relevant articles due to the transdisciplinary background and conceptual ambiguity surrounding our complex topic. The complete search strategy is presented in the supplemental materials (S1 Table).

### Eligibility criteria

We included peer-reviewed journal articles in which investigators reported qualitative, quantitative, and mixed-methods original research conducted in the US. Guided by our PICOS framework, articles were considered eligible if they were reports of research involving adult (ages ≥ 18 years) *Black patients’* (population), *clinical interactions* (intervention), *observed processes* or *patient experiences* (outcomes), compared to *White patients’* (comparator), in the *emergency department* (setting).

We chose to exclude articles published before 2004 because the Institute of Medicine [IOM; 40] report, *Unequal Treatment: Confronting Racial and Ethnic Disparities in Health Care*, altered the “groundwater of the research enterprise” on racial healthcare disparities [66]. The IOM report precipitated a global increase in disparities research [67] and endorsed a research agenda targeting structural inequity and discrimination in patient-clinician interactions to understand and reduce disparities [40].

### Selection process

We derived a decision algorithm from predefined eligibility criteria to guide the screening process. The first author was the primary reviewer and screened all records from databases and reference lists by title and abstract. Articles were passed to the full-text screening stage if they met eligibility criteria or if a decision about eligibility was impossible with the information available in the title and abstract (e.g., methods not reported in the abstract). Two independent reviewers screened the full text of articles included from title and abstract screening stage. The screening team met regularly throughout the screening process to discuss eligibility decisions and reach consensus on any inter-reviewer discrepancies.

### Study risk of bias assessment

Two reviewers independently assessed the quality of included articles using the QualSyst checklist. Designed to summarize the quality of studies from diverse scientific paradigms for systematic reviews, QualSyst [68] consists of two checklists, one for quantitative and another for qualitative research consisting of 14 and 10 items, respectively. Quantitative items included, for example: “question or objective sufficiently described,” “design evident and appropriate to answer study question,” “controlled for confounding,” and “results reported in sufficient detail” [68]. The qualitative checklist included: “context for study is clear,” “design evident and appropriate to answer study question,” “sampling strategy described, relevant, and justified,” and “conclusions supported by the results” [68]. We used the quantitative assessment tool for quantitative studies and the qualitative assessment tool for qualitative studies. Summary scores were calculated as the sum of points assigned by reviewers divided by the total points possible (irrelevant items excluded), reported as a proportional value between 0 and 1 representing lowest and highest quality scores, respectively. Although we did not plan to use quality scores for inclusion decisions, the inclusion cutoff points recommended by Kmet et al. [68] ranged from least conservative, greater than 0.55, to most conservative, or greater than 0.75.

### Data collection process

Two reviewers independently extracted data using a template designed a priori in Covidence. The extraction template focused on research methodology and design, concept definitions, methods, facility characteristics, sample characteristics, results, and sources of potential bias. Data extracted on methodology included research design and whether studies were qualitative, quantitative, or mixed-methods. Facility characteristics included geographic region and whether the ED was affiliated with an academic, public, private, urban, or rural hospital.

Operational definitions of relevant concepts, including race, ethnicity, and race categories, were extracted where reported. Sample sizes, demographic characteristics (age and sex), and compliance with PROGRESS-Plus reporting recommendations were extracted. PROGRESS-Plus, an acronym used to promote an equity lens in research [69], refers to factors that affect health opportunities and outcomes including place of residence, race/ethnicity, culture, occupation, gender and sex, religion, education, social capital, socioeconomic status, *plus* age, ability/disability, and sexual orientation [60,70]. We included these data to assess the degree to which extant ED disparities investigators have encompassed the intersectionality of social constructs. Outcomes were extracted as reported and we noted whether the results indicated any racial disparity in CI processes or experiences. Data points for other potential sources of bias including authors’ explicit limitations and reporting of conflicts of interest–including authors’ racial identities where available–were extracted. As with other stages of our review, differences in extracted data points were synthesized or corrected iteratively by consensus.

### Synthesis methods

The heterogenous definitions and methods researchers use in studies of racial disparities in CI experiences and processes, and the explorative nature of our review, led us to seek qualitative data to inform the conceptual development of future research. Our analysis was guided by the convergent integrated synthesis approach described by Stern et al. [52]. Convergent integrated synthesis is recommended for research questions answerable with evidence synthesis using multiple or mixed research designs. Our synthesis process started with quantitative-to-qualitative data transformation (i.e., qualitization), informed by Bazeley [71], Sandelowski et al. [72], and Hong et al. [73]. Qualitized data were presented as declarative standalone sentences before being categorized thematically with qualitative study data and, finally, integrated to meet our research purpose [52].

## Results

**Fig 1:**
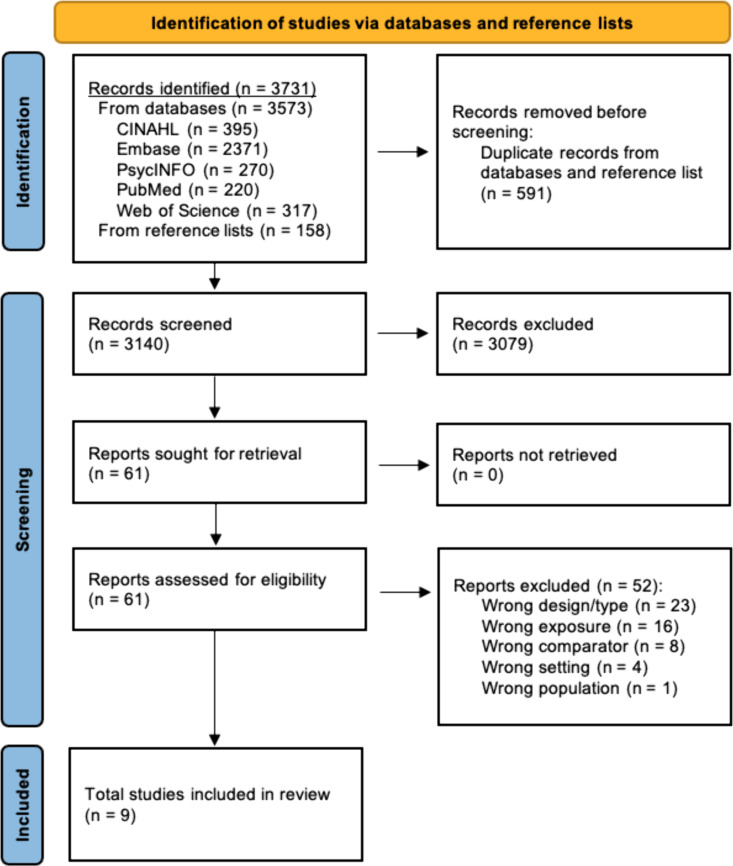
Data sources & screening process (PRISMA) flow diagram. PRISMA 2020 flow diagram for new systematic reviews which included searches of databases, registers and other sources from Page et al. [53], distributed in accordance with the terms of the Creative Commons Attribution (CC BY 4.0) license, available at https://creativecommons.org/licenses/by/4.0/.

**Fig 2:**
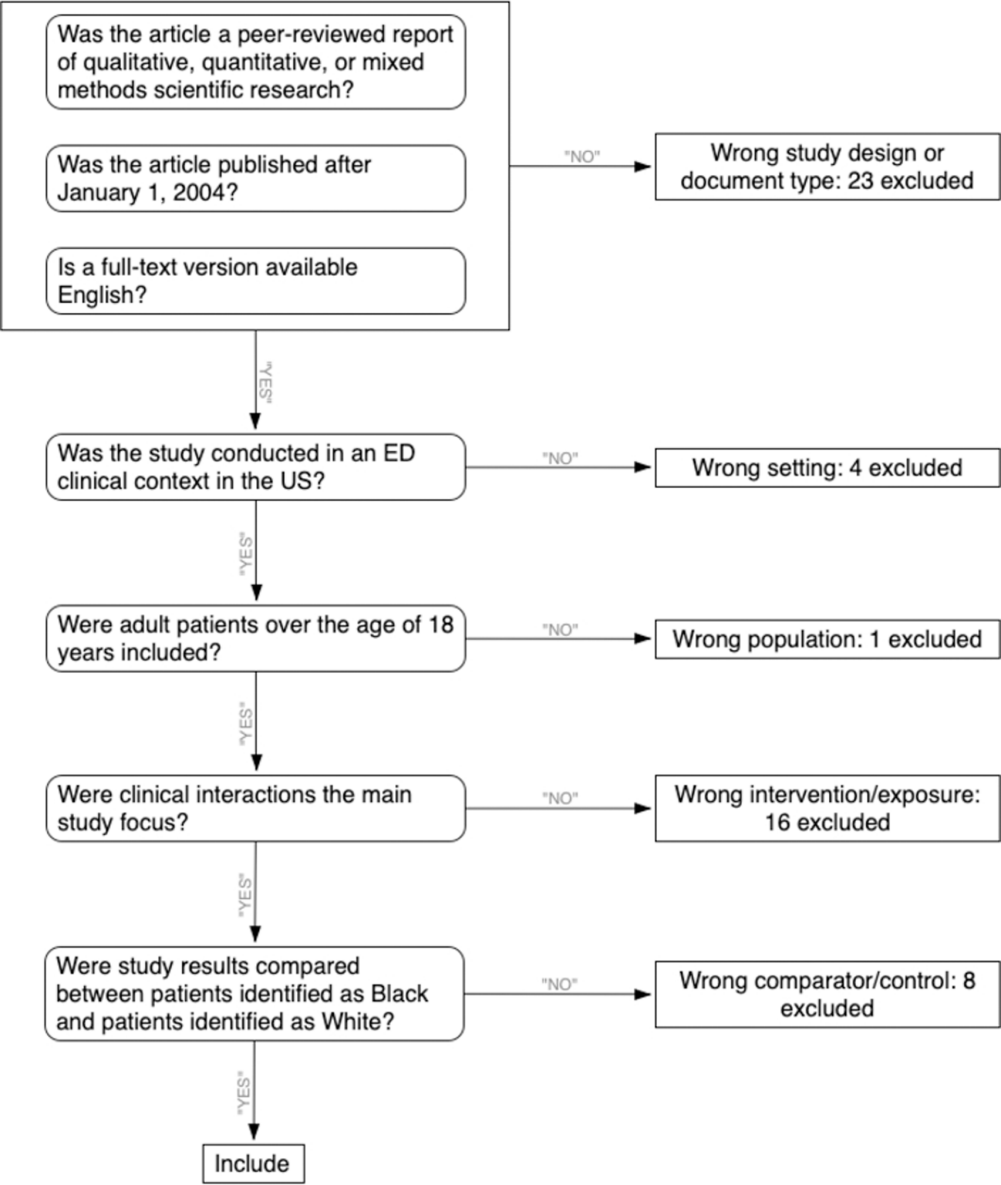
Literature screening decision tree. A decision tree, based on the inclusion and exclusion criteria, guided reviewers’ decisions. Reviewers answered a series of questions to help identify articles for inclusion based on publication type (Peer reviewed research article published in English after January 1, 2004), setting (ED in the US), exposure (clinical interactions), and comparator (Compared patients identified as Black or White). ED = emergency department.

There were 3,573 citations discovered from database queries and 158 from reference lists for a total of 3,731 citations (Fig 1). Duplicate articles were removed (*n* = 591) before the first author screened the remaining 3,140 records by title and abstract. As shown in Fig 1, this screening yielded 61 reports that were eligible for full-text screening. When the independent screening results were compared, there was 92 percent agreement among screeners and Cohen’s Kappa of 0.62 indicating substantial precision in screening decisions [74]. Of the 61 articles, 52 did not meet the inclusion criteria (see Fig 2), yielding nine reports for inclusion in this review. Of the nine, two [75,76] were based on data from the Reactions to Acute Care and Hospitalization [ReACH; 77] study. We included both reports as independent studies because they had different objectives and compared different variables from different samples within the ReACH study. Nine studies, therefore, were included in the final synthesis. Investigators from three studies focused on racial disparities in observed CI processes, and investigators from six studies focused on racial disparities in patient-reported CI experiences.

Of the nine studies included for review, eight used quantitative methods [75,76,78–83] and one used a sequential mixed-methods approach [84]. We extracted data only from the qualitative arm of the mixed-methods study by Aysola et al. [84] because the quantitative arm did not meet inclusion criteria. There were no randomized or blinded designs used in the studies in our sample, therefore three of the 14 quantitative checklist items were not applicable per the QualSyst manual [68]. QualSyst summary scores ranged between 0.73–1.00 (*M* = 0.82, SD = 0.1) and, therefore, met conservative quality thresholds described by Kmet et al. [68]. Results from the quality assessments for each study are presented in S2 Table.

### Sample and setting characteristics

The nine studies included a combined sample of 20,888 ED patients with 3,386 (16.2%) identified as Black and 13,620 (65.2%) as White. Race and ethnicity were inconsistently operationalized and reported across studies. Some authors reported on additional racial identities and there were, therefore, at least 1,838 (8.8%) patients identified as Asian, American Indian or Alaskan Native, Native Hawaiian or Other Pacific Islander, Multiracial, or Other Race. Liyanage-Don et al. [76] and Parast et al. [81] were the only authors who reported ethnicity separately from race. Investigators in three other studies operationalized ethnicity as a race category and the remaining four studies did not mention ethnicity.

Investigators under-reported PROGRESS-Plus variables in the articles that qualified for this review (Table 1). All investigators reported participants’ race, age, and sex (not to be confused with gender). Other PROGRESS-Plus variables were inconsistently reported in other articles, and no investigators addressed participants’ gender identities, sexual orientations, religious affiliations, occupations, culture, or disability.

**Table 1:** PROGRESS-Plus variables reported in included articles.

Article	Race	Gender	Age	Education	SES ^b^	Residence ^c^	Sexual Orientation	Religion	Occupation	Culture	Disability
Group 1: Observed CI Processes											
Aysola et al., 2021^a^	Yes	No	Yes	No	No	No	No	No	No	No	No
Conteh et al., 2023	Yes	No	Yes	No	No	No	No	No	No	No	No
Schnitzer et al., 2020	Yes	No	Yes	No	Yes	Yes	No	No	No	No	No
Group 2: Patient Reported CI Experiences											
Agarwal et al., 2022	Yes	No	Yes	No	No	No	No	No	No	No	No
Cornelius et al., 2018	Yes	No	Yes	Yes	No	No	No	No	No	No	No
Lee et al., 2008	Yes	No	Yes	Yes	Yes	No	No	No	No	No	No
Liyanage-Don et al., 2021	Yes	No	Yes	Yes	No	No	No	No	No	No	No
McCarthy et al., 2013	Yes	No	Yes	No	No	No	No	No	No	No	No
Parast et al., 2021	Yes	No	Yes	Yes	No	No	No	No	No	No	No

SES = Socioeconomic status; CI = Clinical Interactions

^a^Only the qualitative arm of the sequential mixed-methods study was included, all other studies used quantitative methods.

^b^Socioeconomic status was operationalized by proxy using patient insurance data.

^c^Place of residence was operationalized by proxy using “homeless” status in one study

Six of the nine studies in this review were set in urban areas, five in the Northeastern US [75,76,79,80,82,84] and one in the Southeastern US [78]. Two sets of investigators used national samples. Parast et al. [81] surveyed patients from a nationally representative sample of various ED types and geographic regions. Conteh et al. [83] sampled all patients from 186 Hospital Corporation of America (HCA) Healthcare hospital EDs. Parast et al. [81] and Conteh et al. [83] were, therefore, the only investigators to analyze data from rural areas, non-academic facilities, or geographic regions outside the North- and South-eastern US. Geographic context has been associated with racial disparities in measures of patient experience [85], though the included studies were not designed to investigate cultural or systematic influences.

We organized eligible studies into two groups according to study focus: (1) studies of observed CI processes (hereafter referred to as group 1) and (2) studies of patient-reported CI experiences (hereafter referred to as group 2). Abstracted data and qualitized study results are reported in Tables 2 and 3 for groups 1 and 2, respectively. Group 1 consisted of three studies that compared observed CI processes for Black and White patients using either quantitative data generated from electronic health record abstractions [80,83] or qualitative data generated from investigators’ direct observations [84]. Group 2 consisted of six studies that used quantitative data to compare Black to White patient-reported CI experiences [Table 3; 75, 76, 78, 79, 81, 82]. Our qualitization and synthesis findings are organized by group, as presented in Tables 2 and 3, and discussed in the following sections.

**Table 2:** Group 1: Studies focused on observed processes of clinical interactions (n = 3).

Article	Outcomes	Design	Sample & Setting	Variable (Definition/Source)	Key Findings
Aysola et al., 2021	Determinants of racial/ethnic disparities in ED throughput times	Sequential explanatory mixed-methods study (qualitative arm)	Qualitative observations of clinical interactions (eight 120m sessions) in an academic urban ED in the Northeast from 2017–2018 and semi-structured interviews (n = 10 patients, 50% Black, 50% White, n = 9 Physicians)	Physician- and staff-patient interactions (face-to-face encounters/direct observation) Patient and clinician race (observer perceived/direct observation)	Physicians with observed throughput disparities were less engaged in care coordination, advocated less, and used more negative verbal and nonverbal behaviors (i.e., frequent interruptions, dismissive, less physical proximity) for Black patients compared to White.
Conteh et al., 2023	Racial disparities in restraint use (chemical compared to and physical restraints)	Retrospective cohort study	Patients ≥16^*^ years old chemically or physically restrained with diagnosis of psychiatric or substance use disorder at 186 HCA Healthcare hospital EDs from 2016–2019 (n = 12,229, 15% Black, 73% White, 9.8% Hispanic, 2.1% Other) Exclusions: Missing Race or BMI data	Chemical^†^ or physical restraint use (EHR) Patient race (undefined/unknown) Other: Timing of restraint, length of stay, heart rate, BMI, comorbidities (EHR)	There was no discernible correlation between race and the application of restraints (chemical compared to physical).
Schnitzer et al., 2020	Racial disparities in the use of physical restraints	Retrospective review	Adult ED patients from an academic urban ED in the Northeast from 2016–2018 (n = 2,658, 11.5% Black, 67.5% White, 1% Hispanic, 2.5% Asian, 9.5% other, 7.8% missing or declined to answer) Exclusions: None	Use of behavioral physical restraints (restraint ordered/EHR) Patient race (self-identified/EHR) Other: age, sex, diagnosis, insurance, homelessness, history of violence (EHR)	ED Clinicians were more likely to order physical restraints for Black patients compared to White. The disparity was increased for males and patients with substance abuse disorders, psychiatric disorders, homelessness, public or no insurance, and history of violence.

ED = Emergency department; EHR = electronic health record; BMI = body mass index; HCA = Hospital Corporation of America

* Participants’ mean age of 47 years indicated the majority of the sample was over 18 years and therefore reflects our population of interest. The authors retained this study for analysis because of its importance and the otherwise limited number of studies on this topic.

† Chemical restraints were defined as any intravenous, intramuscular, or oral administration of benzodiazepines, ketamine, antihistamines, or anti-psychotic medications.

**Table 3:** Group 2: Studies focused on patient-reported experiences of clinical interactions (n = 6).

Article	Outcome	Design	Sample & Setting	Variables (Measure/Source)	Findings
Agarwal et al., 2022	Patient perceived impact of race on care	Prospective cohort study	Adult patients with mobile-phone numbers on record discharged from two Northeastern academic urban EDs (n = 462, 60% Black, 31% White, 4% Asian, 4.1% other/unknown) Exclusions: No cell phone number available	Patient experience (0–5 scale, 0 = poor, 5 = excellent) Effect of race on experience (positive, negative, or none) Impact of race on respect, quality, and communication (0–5 scale, 0 = not affected, 5 = strongly affected) Patient race (source not reported)	Compared to White patients, Black patients were more likely to report a positive ED experience and that race affected their experience. Black patients who reported that their race negatively impacted care also reported that race affected respect, quality, and communication.
Cornelius et al., 2018	Patient perceptions of clinician-patient communication	Observational cohort study	English or Spanish speaking adults with ACS in a Northeastern academic urban ED from 2013–2016 (n = 876, 20% Black, 17% White, 7% other, 56% Hispanic) Exclusions: non-terminal cardiovascular illness, mental illness, cognitive impairment, alcohol or substance abuse	Physician-patient communication (Physician focused items from IPC survey/patient-reported) Patient race (self-identified) Other: Partner status, sex, age, education, primary language (EHR)	No meaningful associations between patient perceived communication and Black or White patient race were observed.
Lee et al., 2008	Patient perceptions of clinicians’ interpersonal behaviors	Cross-sectional survey	Adult patients in an academic ED in the Southeast in 2004 (n = 372, 29% Black, 63% White, 8% Other) Exclusions: ESI level 1 triage (most acute), non-English speaking, mental health complaint	Patient-reported interpersonal aspects of care measured in four dimensions: affiliation, satisfaction, trust, and participation (ad hoc scale) Patient race (self-identified) Other: age, sex, household income, insurance, recent healthcare use, ED referral source	Black patients reported lower levels of trust, but not affiliation, participation, or satisfaction compared to White patients
Liyanage-Don et al., 2021	Patient perceptions of interpersonal care	Observational cohort study (secondary data analysis)	English or Spanish speaking adults with ACS in a Northeastern academic urban ED from 2013–2016 (n = 933, 23.3% Black, 24.1% White, 1.7% Asian, 10.5% multiracial, 34.2% other, 6.2% declined to answer) Exclusions: non-terminal cardiovascular illness, mental illness, cognitive impairment, alcohol or substance abuse	Perceived interpersonal care (IPC survey/patient-reported) Patient race (self-identified) ED crowding (EDWIN)	There was no evident association between patient perceived interpersonal care and patient race, regardless of ED crowding
McCarthy et al., 2013	Patient perceptions of communication with the medical team	Cross-sectional survey	Adult patients in an academic urban ED in the Northeast from 2007–2008 (n = 226, 41% Black, 48% White, 11% other) Exclusions: Mental health complaints, critically ill or physically unstable, non-English speaking, in custody	Quality of team communication tasks (CAT-T/patient reported) Patient race (self-identified) Other: Age, sex, ED disposition, wait time, ED length of stay, and ED census	Patient-perceived quality of ED team communication was not meaningfully associated with patient race
Parast et al., 2021	Patient experiences of care	Cross-sectional survey	Adult patients from a national sample of EDs in 2016 (n = 3122, 11.9% Black, 64% White, 10.3% Hispanic, 13% other) Exclusions: Primary mental health or substance abuse diagnosis; died in the ED; transferred from outside facility; admitted to hospital; discharged to an inpatient setting or nursing facility; free-standing ED	Patient experience (ED CAHPS®; formerly “EDPEC”) Patient race (self-identified) Other: sex, age, education, primary language, self-reported health, self-reported mental health, rural-urban commuting area, usual sources of care, recent healthcare use, reason for ED visit, arrival mode, perceived importance of getting timely care, proxy helped with survey, and response percentile by survey mode.	Black ED patients tended to have more positive experiences with clinician communication and medication-related communication compared to White ED patients

Note: ED = emergency department; EHR = electronic health record; ACS = Acute Chest Syndrome; ESI = Emergency Severity Index; CAT-T = Communication Assessment Tool for Teams; ED CAHPS® = Consumer Assessment of Healthcare Providers and Systems for the Emergency Department; EDPEC = Emergency Department Patient Experiences of Care Survey; EDWIN = Emergency Department Work Index

†Experiences were more positive for Black patients compared to White patients

### Group 1: Observed clinical interaction processes

Investigators from three studies focused on racial disparities in observed CI processes in the ED (Table 2). Two of those studies were retrospective cohort designs [80,83] and one was a prospective sequential explanatory mixed-methods design [84]. The two quantitative studies focused on restraint practices in the ED. The application of physical restraints in the ED context is relevant to our research question as a proxy for adverse CI processes indicative of a breakdown in clinician-patient interpersonal dimensions. Physical restraints can have adverse effects on patients’ experiences of care and wellbeing [86] and should only be applied after other less restrictive interventions have failed [87]. There is, however, a complex interplay of factors external to the ED that influence racial disparities in the use of restraints [88]. We were not able to determine the context or rationale for restraint use in these studies.

Schnitzer et al. [80] studied racial disparities in the application of physical restraints, and Conteh et al. [83] studied the relationships between patient race and the type of restraint used—chemical compared to physical restraints. Schnitzer et al. [80] analyzed data from 2,658 adult patients who visited an academic urban ED in the Northeastern US between 2016 and 2018 and concluded that Black patients were more likely than White patients to be physically restrained with a risk ratio of 1.22 and 95% CI [1.05, 1.40]. Conteh et al. [83] studied the records of 12,229 patients from 186 Hospital Corporation of America (HCA) Healthcare hospital EDs and found no statistically significant correlation between patient race and the use of chemical compared to physical restraints.

In the qualitative arm of sequential mixed-methods explanatory study, Aysola et al. [84] used data from observed CIs to identify contributors to disparities in ED throughput times they observed in the quantitative arm. Prior to that, however, using quantitative data generated from time-stamped electronic medical records, the authors reported that Black patients waited, on average, 29 minutes longer from arrival to decision to admit than White patients. Qualitative data were generated from both direct observations of CIs in the ED and semi-structured interviews with 10 patients (*n* = 5 Black, *n* = 5 White) and 9 Physicians. Observational data were generated by two to three researchers during eight purposively sampled 2-hour sessions using field-note templates to create contemporaneous reports.

Aysola et al. [84] reported that clinicians’ communication behaviors with and in reference to patients, approaches to patient advocacy, and methods of patient prioritization varied between Black and White patients. Clinicians were observed interrupting, avoiding eye contact, and ignoring the requests of Black patients more than with their White patients [84]. The authors concluded that these differences in observed clinician behaviors may contribute to disparate ED throughput times for Black patients compared to White patients [84]. The study by Aysola et al. [84] was unique among included studies for two reasons: it was the only study that generated data from directly observed CIs and it was the only study in which race was operationalize as perceived by the observer.

### Group 2: Patient-reported clinical interaction experiences

Investigators from six quantitatively designed studies compared dimensions of patient-reported CI experiences between Black and White ED patients (Table 3). These studies included a combined sample of 5,991 patients consisting of 20.7% Black patients (*n* = 1,242) and 48.1% White patients (*n* = 2,883). The remaining 31.2% (*n* = 1,866) of the sample consisted of patients who reported their race as Multiracial (5.6%), Asian (1.7%), Hispanic (operationalized as race; 43.9%), other (45.7%), and unknown or declined to answer (3.2%). Four of these studies were set in academic urban EDs in the Northeast [75,76,79,82], one was conducted in an academic urban ED in the Southeast [78], and one used a nationally representative sample of various ED types [81].

Each of the six studies used different instruments or instrument components to measure various aspects of patients’ CI experiences. Parast et al. [81] used the Emergency Department Consumer Assessment of Healthcare Providers and Systems [ED CAHPS; formerly the Emergency Department Patient Experience of Care survey; [89,90]]. McCarthy et al. [79] measured patients’ communication experiences using the Communication Assessment Tool for Teams [CAT-T; 91]. Studies based on the ReACH study data used different items from the Interpersonal Processes of Care (IPC) scale to measure patients’ experiences of interpersonal care [91,92]. Liyanage-Don et al. [76] used all 18 items of the IPC scale, which includes items regarding non-physician staff. Cornelius et al. [75] used a subset of IPC items to focus on patients’ experiences with physicians. Lee et al. [78] created four scales to measure patients’ experiences of interpersonal subdomains including satisfaction, affiliation, participation, and trust. Finally, Agarwal et al. [82] used a series of questions with scaled responses sent to patients via text messages to measure their overall satisfaction with ED care and the degree to which they believed their races influenced the respect, communication, and quality they experienced from ED staff.

Three of the six studies in group 2 (50%) reported evidence of differences in patient-reported CI experiences for Black compared to White ED patients. Lee et al. [78] found that Black patients reported lower levels of trust in ED clinicians compared to White patients but found no differences in the domains of affiliation (i.e., non-verbal communication), participation (i.e., person-centeredness), or satisfaction. Parast et al. [81] and Agarwal et al. [82] both found that Black patients reported more positive experiences in the ED compared to White patients. No differences were observed for Black compared to White ED patient-reported experiences in measures of team communication [CAT-T; 79] or any of the Interpersonal Processes of Care domains [IPC; 75, 76].

Comparative analyses were impossible given the heterogeneity of methods and instruments used. We identified four themes that were consistent across the six studies in which patients’ CI experiences were quantified: (1) *responsive nonverbal behavior*, (2) *effective verbal communication*, (3) *person centeredness*, and (4) *patient satisfaction*. These four themes are discussed below and tabulated in the supplemental materials (S3 Table). These themes represent the aspects of patients’ CI experiences previously studied in relation to racial healthcare disparities, although the findings need to be regarded tentatively given that the psychometric properties of some of the instruments were not as robust as desired.

### Responsive nonverbal communication

Most scale items from the six studies of patient-reported CI experiences were used to ask about patients’ perceptions of clinicians’ nonverbal behaviors (33 of 58 items, or 56.9%). Nonverbal communication refers to the interpersonal exchange of information through “nonlinguistic, informative aspects of behavior and appearance, including head and body features or movements, touch, interpersonal distance, and paralanguage” [93]. Patient perceived clinician respect, understanding, empathy, warmth, trust, listening, discrimination, racism, and other perceived traits or states were the focus of items in this theme. Examples of questions related to nonverbal communication included: “How often did doctors really respect you as a person” [IPC; [92]]; “The medical team understood my main health concerns” [CAT-T; [94]]; “My doctor was friendly and warm toward me” [“affiliation” scale from Lee et al.; [78]]; and “How often did nurses listen carefully to you” [ED CAHPS; [89]].

Lee et al. [78] developed a trust scale consisting of two items that we categorized as nonverbal communication because they assessed patients’ evaluations of clinicians’ intentions: “If a mistake was made in my treatment, my doctor would try to hide it from me,” and “My doctor sometimes pretends to know things when really not sure” [78]. We also included items from Agarwal et al. [82] that assessed the patient-reported influence of their race on the respect, communication, and quality of service they experienced in the ED. Items included the stem, “Did you feel your race affected …” [82]. Assuming the absence of explicit discrimination, these items evaluate nonverbal aspects of interpersonal behaviors.

### Effective verbal communication

With the effective verbal communication theme, we categorized scale items targeting patient-perceived quality of communication and availability of information regarding wait times, physical examination findings and diagnostic test results, diagnoses, and treatment options including risks and follow-up care. Effective verbal communication items included, for example: “How often did doctors speak too fast” [IPC; 92]; “The medical team checked to be sure I understood everything” [CAT-T; 94]; “I was kept well informed about delays in my care” [“satisfaction” scale from Lee et al.; 78]; and “Did the doctors or nurses ask about all of the medicines you were taking” [ED CAHPS; 89].

Effectiveness in healthcare refers to care that reliably achieves the desired outcomes according to best scientific evidence [95]. Effective verbal communication in healthcare, therefore, refers to the role of spoken language in achieving desired evidence-based outcomes. From the clinician’s perspective in the context of CIs, the desired outcomes from verbal communication include solving medical problems and addressing patients’ needs [96]. From the patient’s perspective, however, the desired outcomes of verbal communication include adequate information and the degree to which it helps patients understand their health and healthcare needs [97].

### Person centeredness

Patient experience scales from the reviewed studies included eight items relevant to person centeredness. Person centeredness, or person centered care, refers to an approach to healthcare that actively incorporates the perspectives, preferences, and experiences of patients and their families in the processes of healthcare planning and delivery [98]. Shared decision making is a primary dimension of person centeredness [99]. Shared decision making is a multidimensional transactional process designed to transfer power in medical decision-making from clinicians to patients [31,100]. Shared decision making is an especially important tool for empowering marginalized patient populations in the ED [101].

Person centeredness, and therefore shared decision making, included scale items pertaining to patient engagement, care coordination, and especially, shared decision making. Items targeting patients’ perceptions of person centeredness included, for example: “How often did the doctor give you different treatment options/choices” [IPC; 93]; “The medical team involved me in decisions as much as I wanted” [CAT-T; 91]; and “Before you left the emergency room, did someone ask if you would be able to get this follow-up care” [ED CAHPS; 89]. Lee et al. [78] measured “participation” using three items that targeted aspects of shared decision making [102]: “My doctor listened to my wishes about my care,” “My doctor included me in decision-making about my care,” and “I had enough to say about my treatment.”

### Patient satisfaction

Patient satisfaction, defined as the extent to which patients’ experiences of the ED satisfied their expectations and goals [103], can be affected by various factors that are not related to CIs (e.g., ED facilities). It has been, however, an indicator commonly used by researchers to investigate disparities in CI experiences. All the included studies collected patient-reported data on patient satisfaction. Items in this domain ranged from questions about wait times to patients’ expectations. Among the most common items was: “Would you recommend this emergency room to your friends and family?” with response options varying from four to six point scales (e.g., “Definitely No,” “Probably No,” “Probably Yes,” and “Definitely Yes;” or 0–5, 0 = Strongly Disagree, 5 = Strongly Agree).

### Summary of findings

A summary of our findings is presented in Table 4. The evidence of disparities in observed processes and patient-reported experienced of CIs in the ED was inconsistent. Two of three studies in group 1 (studies of observed CI processes) reported differences that negatively affected the care of patients identified as Black. Three of six studies in group 2 (studies of patient-reported CI experiences) reported differences, but two of those three found more positive CI experiences reported by Black patients compared to White patients. Extant evidence of racial disparities in ED CIs, therefore, is inconclusive regarding both observed CI processes and patient-reported CI experiences.

**Table 4:** Presence and direction of differences between Black and White ED patients reported from included studies.

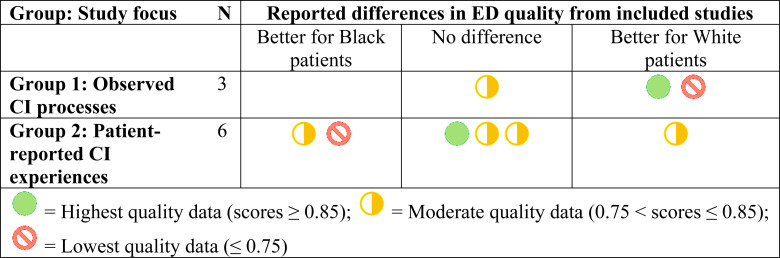

Quality scores assessed with the QualSyst checklists for quantitative, qualitative, and mixed-methods research [68]. ED = emergency department, CI = clinical interaction

## Discussion

We set out to synthesize the quantitative and qualitative evidence of disparities in ED CI processes and experiences for Black compared to White patients in the US. The extant evidence is mixed and insufficient to draw any conclusions about how patient race affects CIs in the ED. Studies on racial disparities in ED CIs are widely variable in terms of their foci, methodologies, theoretical foundations, and especially approaches to measurement. Despite these limitations, we identified common themes across studies of patient-reported CI experiences including responsive nonverbal behavior, effective verbal communication, person centeredness, and patient satisfaction. Investigators’ singularity of mind in assessing aspects of these themes when studying racial ED disparities should be noted and expanded in future research.

Higher ED visit rates among Black patients compared to other racial groups are frequently attributed to differences in access or use of primary care services [104]. Another explanation could be that, as reported by Parast et al. [81] and Agarwal et al. [82], Black patients may have more positive experiences in the ED compared to their experiences in other healthcare settings. Existing evidence suggests that Black patients perceive worse CI experiences than White patients in non-ED healthcare settings [43,105,106]. Two studies included in this review [81,82], however, reported evidence that Black patients had more positive ED CI experiences when compared to White patients. An interesting research question for future investigators, therefore, is whether Black patients have more positive experiences of CIs in the ED compared to their experiences of CIs in other healthcare contexts (e.g., primary care). We are not aware of any studies that compare patient-reported CI experiences of Black or White patients in primary care to their CI experiences in the ED. Despite potentially better patient-reported CI experiences, observed CI processes were worse for Black patients compared to White patients in two of three group 2 studies.

The findings of Schnitzer et al. [80], that the use of physical restraints in ED patients identified as Black is higher compared to White, have been supported by two other studies. A multicenter retrospective cohort study of 2,458 physically restrained ED patients under involuntary mandatory emergency psychiatric evaluation found similarly disparate physical restraint practices for Black (adjusted odds ratio [aOR] = 1.22, 95% CI [1.01, 1.48]) and Hispanic (aOR = 1.45, CI [1.22, 1.73]) patients compared to White patients [107]. Another study of three EDs in a Northeastern academic healthcare system compared demographic characteristics of 7,090 physically restrained patients and also found that Black patients were more likely to be restrained than White patients [88]. Restraint use likely reflects, however, more systemic inequities beyond the scope of the ED CI.

Theoretical questions emerged in our review of the ED racial disparities literature that researchers will need to address to advance the science. Most importantly, we found definitions and operationalizations of race and ethnicity to be inadequate. Self-identified race was the most cited operational definition used in generating race data in our sample and in the health sciences literature [108–112]. It should not be assumed, however, that self-identified race is the definition of race that leads to healthcare disparities because it may not be the same race as perceived and recorded by healthcare system representatives. Aysola et al. [84], as one of the more recent studies, operationalized patient race as observer perceived race, also known as socially assigned race. Socially assigned race refers to others’ perceptions of one’s race and is the definition associated with race bias, race discrimination, and racism [112]. Observer perceived race, therefore, may be more appropriate when operationalizing the concept of race in healthcare disparities research.

We identified methodological issues primarily in the survey instruments used to measure patients’ CI experiences in the group 2 studies. The validity and reliability of the patient-reported instruments used by Agarwal et al. [82], Cornelius et al. [75], and Lee et al. [78] were not as robust as desired for several reasons. For instance, using select scales or items from existing instruments may alter their psychometric properties [113].

Patient race has been linked to temporal measures of ED quality in previous research, including research on wait times and overall ED lengths of stay [114]. Temporal quality indicators related to ED throughput are critical to ED efficiency and quality assurance [33]. Racial disparities in temporal measures of observed CI processes have also been demonstrated by investigators studying CIs but not in the ED setting [115–118]. We found only one study that observed real-world ED CIs to understand racial disparities in temporal quality markers, yet they did not measure any temporal aspects of the CIs they observed. The lack of research on racial disparities in observed CI processes represents a substantial gap in the literature.

Lack of trust in healthcare professionals and institutions, or medical mistrust, is commonly referenced in the literature on racial healthcare disparities, especially in relation to Black people in the US [119]. Investigators of one study in this review found that Black patients experienced lower trust in ED Physicians when compared to White patients [78]. Despite its common place in literature, medical mistrust is a poorly defined, understood, and operationalized concept [120]. Medical mistrust, therefore, represents another gap in the literature on racial healthcare disparities [121].

Cultural and structural contexts across US geographic regions represent additional challenges that merit careful consideration by disparities researchers. Four of the six included studies were conducted in the Northeast, one in the Southeast, and two surveyed nationally representative samples. W.E.B. DuBois referred to the US South as a vast opportunity for the study of racial contacts between Blacks and Whites [122]. The variability of structural and cultural racism across different US community settings and geographic regions has unknown effects on healthcare disparities, though the historical evolution of modern social structures and problems likely plays a role.

We mirror the calls of generations of scholars [123–125] in recommending that researchers use a carefully reflexive approach–if not a culturally humble approach [126]–to investigations using the concepts of race, ethnicity, or inequity in all research disciplines. Race is a complex, multifaceted social construct that is both dynamic and context-specific [108]. Ethnicity is an equally hazardous concept. No authors from the studies we reviewed provided a definition of race or ethnicity. Furthermore, no articles included in our review reported all the recommended PROGRESS-Plus variables. Authors referenced gender and sex interchangeably and operationalized both as dichotomous (i.e., either female or male) variables. Because these variables were largely missing from our sample, it is impossible to determine the extent to which other important social variables may have confounded the findings.

Individuals in the ED enact various roles to accomplish the goals of the healthcare organization: people seeking healthcare take on the role of the patient and those providing healthcare take on the roles of physicians, registered nurses, care technicians, respiratory therapists, dentists, psychologists, and more. Most studies in this review targeted physician-patient interactions, specifically. Most scales instructed patients to think of the “doctor” or “medical team” responsible for their care when responding to items. Three items from the ED CAHPS [89] survey and four from the IPC scale focused specifically on CIs with nurses or other ED team members. Four items from the IPC focus on negative or disrespectful behaviors of “office staff” [92]. While broad and unspecific terms like “office staff” make it difficult to understand where specific problems lie, it is important to study all types of healthcare professionals and representatives. Limiting research to studies of physician-patient CIs is problematic because any clinician-patient encounter can affect patient experiences, clinical processes, health outcomes, and, therefore, racial healthcare disparities.

## Limitations

Known and unknown sources of bias can limit the reliability and generalizability of research findings. We limited bias by registering a protocol with PROSPERO a priori and reporting the review in accordance with PRISMA-2020 guidelines. We were aware of several specific limitations that should be noted. First, all stages of the screening process would ideally be done by two reviewers with a third reviewer for conflict resolution. Because of resource limitations, a single reviewer conducted the title and abstract screening process. The quality assessment tool we used may not have been the best choice for this topic, though QualSyst has been widely used in synthesis research since its creation by Kmet et al. [68]. QualSyst did not include assessment of the validity and reliability of patient-reported measures which we found to be lacking in several studies as previously mentioned. It was our impression that the quality of studies was overestimated. We only used quality scores, however, to guide our findings and not as inclusion criteria.

Authors’ self-identified racial group affiliations may introduce bias to research. A majority-White review team admittedly complicates both the ethics and the potential utility of our findings. Scholars have questioned the reliability of health disparities research designed and conducted by White investigators [127]. Racial disparities research has become a major research focus in recent years, and new scholars to the field, labelled “health equity tourists” [127] by some, may lack the experience or perspectives necessary for conducting reliable, rigorous research.

Finally, despite meeting inclusion criteria for this review, studies of racial disparities in ED patient restraint practices may be poor proxies for evidence of adverse CI processes. Racial disparities that exist beyond the ED environment in the larger social and cultural context may be stronger predictors of ED restraint practices than anything that happens within the ED. Racial disparities in incarceration rates [128,129] or lack of access to mental health care [130], for example, may predispose some populations (e.g., incarcerated patients, patients with schizophrenia) to being restrained in the ED. Future research on racial disparities in restraint practices should take care to account and control for the external ecology of factors.

## Conclusion

This review advances current knowledge by synthesizing and revealing important gaps in the peer-reviewed research literature on observed processes and patient-reported experiences of CIs in the ED for Black compared to White patients in the US. We found the literature to be sparse and the research designs and findings too heterogenous to make meaningful comparisons. Interesting and important gaps in the knowledge were identified. First, there is scarce research on observed CI processes using real-world ED data. We found only one study of this type and additional research is urgently needed to move the science forward. Research focused on understanding the differences in Black and White patients’ CI experiences in EDs compared to other healthcare contexts is also needed given the contradictions currently presented in the literature. Finally, theoretical and conceptual clarity, methodological consistency, and measurement rigor are lacking from research in this area. Investigators designing future studies about race and CIs in the ED should carefully consider their operationalization of social constructs (e.g., race, ethnicity).

## Supporting information

S1 Table Complete search strategy arranged by PICOS element. ^a^ PICOS = Population, Intervention, Comparison/Control, Outcome, Setting/Context. ^b^ Web of Science: Core Collection; “apply equivalent subjects” unchecked. ^c^ Embase; “Automatic mapping” unchecked. ^d^ CINAHL; “Apply equivalent subjects” unchecked. (DOCX)

S2 Table Quality assessment results organized by study focus: Experiences and processes of clinical interactions. Note: The QualSyst quantitative checklist has 14 items, three of which are not shown because they were not applicable to the reviewed studies. The Qualitative checklist has 10 items, all of which are shown. CI = clinical interaction. ª Publication year. ^b^ Item 11 not applicable to the qualitative data from Aysola et al., 2021.
(DOCX)

S3 Table Themes from measures of patients’ clinical interaction experiences. Note: ED = emergency department; ER = emergency room; IPC = Interpersonal Processes of Care survey; ED CAHPS = Emergency Department Consumer Assessment of Healthcare Providers & Systems survey; CAT-T = Communication Assessment Tool for Teams. ^a^ Subdomains were only identified for the IPC scale.
(DOCX)

S4 Table All studies identified in the literature search. Note: Exclusion reasons were recorded only for articles screened by full text.
(PDF)

S1 File International prospective register of systematic reviews (PROSPERO) Protocol. (PDF)

S2 File Raw data extracted from included studies. (PDF)
