# Supplementary material for: Effects of patient race on processes and experiences of clinical interactions in US emergency departments: A mixed-methods systematic review
Source: PLoS One. 2025 Jun 25;20(6):e0325315. doi: 10.1371/journal.pone.0325315 (PMC12192141; doi:10.1371/journal.pone.0325315)
Supplement: S1 Table — (DOCX) [file pone.0325315.s001.docx]

S1 Table. Complete search strategy arranged by PICOS element.

| **PICOS^a^ Element** | **PubMed/MEDLINE** | **Web of Science^b^** | **Embase^c^** | **CINAHL^d^** | **PsychINFO** |
| --- | --- | --- | --- | --- | --- |
| #1 (Population): Patients identified as Black | "African Continental Ancestry Group"[MH] OR "African Americans"[MH] OR "African American"[TW] OR "African-American"[TW] OR "Black American"[TW] OR "Black Americans"[TW] OR "African ancestry"[TW] OR "People of Color"[TW] OR "underserved"[TW] OR "vulnerable population*"[TW] OR "Black"[TW] OR "Racial"[TW] OR “Racial/Ethnic”[TW] OR "Race/Ethnicity"[TW] OR "Minorities"[TW] OR "Minority"[TW] OR “Minority Groups”[MH] OR "Black-White"[TW] OR "White-Black"[TW] OR “ethnic*”[TW] OR “race”[TW] | TS=("African Americans" OR "African American" OR "African-American" OR "Black American" OR "African ancestry" OR "People of Color" OR "Black Americans" OR "underserved" OR "vulnerable population*" OR "Black" OR "Racial" OR "Race/Ethnic*" OR Racial/Ethnic OR "Minorities" OR "Minority" OR "Black-White" OR "White-Black" OR “ethnic*” OR “race”) | ('African American'/exp OR 'Black person'/mj OR 'race'/exp OR 'vulnerable population'/exp OR ‘African Americans’ OR ‘African American’ OR ‘African-American’ OR ‘Black American’ OR ‘African ancestry’ OR ‘People of Color’ OR ‘Black Americans’ OR ‘underserved’ OR ‘vulnerable population*’ OR ‘Black’ OR ‘Racial’ OR ‘Race/Ethnic*’ OR ‘Racial/Ethnic’ OR ‘Minorities’ OR ‘Minority’ OR ‘Black-White’ OR ‘White-Black’ OR ‘ethnic*’ OR ‘race’) | (MH "Black Persons" OR "African Americans" OR "African American" OR "African-American" OR "Black American" OR "African ancestry" OR "People of Color" OR "Black Americans" OR "underserved" OR "vulnerable population*" OR "Black" OR "Racial" OR "Race/Ethnicity" OR “Racial/Ethnic” OR "Minorities" OR "Minority" OR "Black-White" OR "White-Black" OR “ethnic*” OR “race”) | (DE "Blacks" OR "African Americans" OR "African American" OR "African-American" OR "Black American" OR "African ancestry" OR "People of Color" OR "Black Americans" OR "underserved" OR "vulnerable population*" OR "Black" OR "Racial" OR "Race/Ethnicity" OR  “Racial/Ethnic” OR "Minorities" OR "Minority" OR "Black-White" OR "White-Black" OR ethnic* OR race) |
| #2 (Intervention/ Exposure): Clinical Interactions | (("clinician-patient"[TW] OR "patient-clinician"[TW]) AND "interaction*"[TW]) OR "Social Interaction"[MH] OR "Interpersonal Relations"[TW] OR "Interpersonal Relations"[MH] OR "Physician-Patient"[TW] OR "Nurse-Patient"[TW] OR "Professional-patient"[TW] OR "Patient-practitioner Relations*"[TW] OR "Clinician-Patient Relations*"[TW] OR "relationship-based care"[TW] OR "Nurse-Patient Relations"[MH] OR "Physician-Patient Relations"[MH] OR "Professional-Patient Relations"[MH] OR "relationship-centered"[TW] OR "client-centered"[TW] OR "patient-centered"[TW] OR "person-centered"[TW] OR "Social Skills"[MH] OR "Social Perception"[MH] OR "Sociological Factors"[MH] OR "Patient Participation"[MH] OR "Communication"[MH] OR "Communication"[TW] OR "Communicate"[TW] OR “advocacy”[TW] OR “advocate”[TW] OR "Nonverbal Communication"[MH] OR "nonverbal"[TW] OR "Communication Barriers"[MH] OR "Verbal Behavior"[MH] OR "word use"[TW] OR "linguistics"[TW] OR "discourse"[TW] OR "language use"[TW] OR "Attitude of Health Personnel"[MH] OR "Helping Behavior"[MH] OR "Decision Making"[MH] OR "Mirror neurons"[TW] OR "Mirroring"[TW] OR "Theory of mind"[TW] OR "Sensitivity"[TW] OR "empathy"[MH] OR "empathy"[TW] OR "Clinical Empathy"[TW] OR "Empathic connection"[TW] OR ("understanding"[TW] AND "emotion"[TW]) OR "emotional understanding"[TW] OR "Emotional Intelligence"[TW] OR "belonging"[TW] OR "exchange"[TW] OR "attachment"[TW] OR "role taking"[TW] OR "person perception"[TW] OR "emotional intelligence"[TW] OR "social skills"[TW] OR "empathic behavior"[TW] OR "interactive competence"[TW] OR "social factors"[TW] OR "social norms"[TW] OR "social rules"[TW] OR "affect"[TW] OR "affectivity"[TW] OR "affective"[TW] OR "self-awareness"[TW] OR "similarity"[TW] OR "familiarity"[TW] OR "affection"[TW] OR "sympathy"[TW] OR "compassion"[TW] OR "symbolic interactionism"[TW] OR "perspective taking"[TW] OR "Responsiveness"[TW] OR "responsive"[TW] OR "responsiveness"[TW] OR "responsivity"[TW] OR "response"[TW] OR "rapport"[TW] OR "Gaze cueing"[TW] OR "Mental imagery"[TW] OR "Mental rotation"[TW] OR "Perspective-taking"[TW] OR "Social cognition"[TW] OR "Visual cognition" OR "culturally competent care"[MH] OR "culturally appropriate care"[TW] OR "cultural competence"[TW] OR "racial discrimination"[TW] OR "social discrimination"[MH] OR "racial prejudice"[TW] OR "social marginalization"[MH] OR "racial segregation"[TW] OR "social segregation"[MH] OR "racism"[MH] OR "racism"[TW] OR "prejudice"[MH] OR "Racial Bias"[TW] OR "Race Relations" [MH] OR "unconscious bias"[TW] OR "bias"[TW] OR "attitude"[TW] OR "implicit bias"[TW] OR "latent bias"[TW] OR "power, psychological"[MH] OR ("power"[TW] AND "psychological"[TW]) OR "psychological power"[TW] OR "power"[TW] OR "Mistrust"[TW] OR "Trust"[TW] OR "Self-Disclosure"[TW] OR "Discourse Analysis"[TW] OR "face-to-face"[TW] OR "gestures"[TW] OR "Facial Displays"[TW] OR "Facial Expressions"[TW] OR "Cues"[TW] OR “eye contact”[TW] OR "Patient-Centered Care"[MH] OR "Person-Centered*"[TW] OR "Patient-Centered*"[TW] | TS=(interaction* OR "clinician-patient" OR "patient-clinician" OR "Interpersonal" OR relations* OR "Physician-Patient Interaction" OR "Nurse-Patient Interaction" OR "Professional-patient interaction" OR "Patient-practitioner Relations" OR "Clinician-Patient Relations" OR "Clinician-Patient Relationship" OR "relationship-based care" OR "Nurse-Patient Relations" OR "Physician-Patient Relations" OR "Professional-Patient Relations" OR "relationship-centered" OR "client-centered" OR "patient-centered" OR "person-centered" OR "Social Skills" OR "Social Perception" OR "Sociological Factors" OR "Patient Participation" OR "Communication" OR "Communicate" OR advocacy OR advocate OR Nonverbal OR "Communication Barriers" OR "Verbal Behavior" OR "word use" OR "linguistics" OR "discourse" OR "language use" OR "Attitude of Health Personnel" OR "Helping Behavior" OR "Decision Making" OR "Mirror neurons" OR "Mirroring" OR "Theory of mind" OR "Sensitivity" OR "empathy" OR "empathy" OR "Clinical Empathy" OR "Empathic connection" OR "understanding" OR "emotion" OR "emotional understanding" OR "Emotional Intelligence" OR "belonging" OR "exchange" OR "attachment" OR "role taking" OR "person perception" OR "emotional intelligence" OR "social skills" OR "empathic behavior" OR "interactive competence" OR "social factors" OR "social norms" OR "social rules" OR "affect" OR "affectivity" OR "affective" OR "self-awareness" OR "similarity" OR "familiarity" OR "affection" OR "sympathy" OR "compassion" OR "symbolic interactionism" OR "perspective taking" OR "Responsiveness" OR "responsive" OR "responsiveness" OR "responsivity" OR "response" OR "rapport" OR "Gaze cueing" OR "Mental imagery" OR "Mental rotation" OR "Perspective-taking" OR "Social cognition" OR "Visual cognition" OR "culturally competent care" OR "culturally appropriate care" OR "cultural competence" OR "racial discrimination" OR "social discrimination" OR "racial prejudice" OR "social marginalization" OR "racial segregation" OR "social segregation" OR "racism" OR "racism" OR "prejudice" OR "Racial Bias" OR "Race Relations" OR "unconscious bias" OR "bias" OR "attitude" OR "implicit bias" OR "latent bias" OR "psychological power" OR "power" OR "Mistrust" OR "Trust" OR "Self-Disclosure" OR "Discourse Analysis" OR "face-to-face" OR "gestures" OR "Facial Displays" OR "Facial Expressions" OR "Cues" OR “eye contact” OR "Patient-Centered*" OR "Person-Centered*") | ('doctor patient relationship'/exp OR 'nurse patient relationship'/exp OR ‘Clinical interactions’ OR 'social interaction'/exp OR ‘interpersonal’ OR ‘relations*’ OR ‘Physician-Patient Interaction’ OR ‘Nurse-Patient Interaction’ OR ‘Professional-patient interaction’ OR ‘Patient-practitioner Relations’ OR ‘Clinician-Patient Relations’ OR ‘Clinician-Patient Relationship’ OR ‘relationship-based care’ OR 'professional-patient relationship'/exp/mj OR ‘relationship-centered’ OR ‘client-centered’ OR ‘patient-centered’ OR ‘person-centered’ OR 'social competence'/exp OR ‘Social Perception’ OR ‘Sociological Factors’ OR 'social aspects and related phenomena'/exp OR 'patient attitude'/exp OR ‘Patient Participation’ OR 'interpersonal communication'/de OR 'interpersonal communication'/mj OR ‘Communication’ OR ‘Communicate’ OR ‘advocacy’ OR ‘advocate’ OR 'nonverbal communication'/exp OR ‘nonverbal’ OR 'communication barrier'/exp OR 'verbal behavior'/exp OR ‘Communication Barriers’ OR ‘Verbal Behavior’ OR ‘word use’ OR ‘linguistics’ OR ‘discourse’ OR ‘language use’ OR 'health personnel attitude'/exp OR ‘Attitude of Health Personnel’ OR 'cooperation'/exp OR ‘Helping Behavior’ OR 'decision making'/exp OR 'mirror neuron'/exp OR 'mirroring'/exp OR ‘Theory of mind’ OR ‘Sensitivity’ OR 'empathy'/exp OR ‘Clinical Empathy’ OR ‘Empathic connection’ OR ‘understanding’ OR ‘emotion’/mj OR ‘emotional understanding’ OR ‘Emotional Intelligence’ OR ‘belonging’ OR ‘exchange’ OR ‘attachment’ OR ‘role taking’ OR ‘person perception’ OR ‘social skills’ OR ‘empathic behavior’ OR ‘interactive competence’ OR ‘social factors’ OR ‘social norms’ OR ‘social rules’ OR 'affect'/exp OR ‘affectivity’ OR ‘affective’ OR ‘self-awareness’ OR ‘similarity’ OR ‘familiarity’ OR ‘affection’ OR 'sympathy'/exp OR 'compassion'/exp OR ‘symbolic interactionism’ OR ‘perspective taking’ OR ‘Responsiveness’ OR ‘responsive’ OR ‘responsivity’ OR ‘response’ OR ‘rapport’ OR ‘Gaze cueing’ OR ‘Mental imagery’ OR ‘Mental rotation’ OR ‘Perspective-taking’ OR ‘Social cognition’ OR ‘Visual cognition’ OR 'transcultural care'/exp OR ‘culturally competent care’ OR ‘culturally appropriate care’ OR ‘cultural competence’ OR ‘racial discrimination’ OR ‘social discrimination’ OR ‘racial prejudice’ OR ‘social marginalization’ OR ‘racial segregation’ OR ‘social segregation’ OR ‘racism’ OR 'prejudice'/exp OR ‘Racial Bias’ OR 'race relation'/exp OR ‘unconscious bias’ OR 'cognitive bias'/exp OR ‘bias’ OR ‘attitude’ OR ‘implicit bias’ OR ‘latent bias’ OR 'behavior'/mj OR ‘psychological power’ OR ‘Mistrust’ OR ‘Trust’/exp OR ‘Self-Disclosure’ OR ‘Discourse Analysis’ OR ‘face-to-face’ OR 'gesture'/exp OR ‘Facial Displays’ OR ‘Facial Expressions’ OR ‘Cues’ OR ‘eye contact’ OR ‘Person-Centered’ OR ‘Person-Centered*’ OR ‘Patient-Centered*’) | (MH "Physician-Patient Relations" OR MH "Race Relations" OR MH "Racism" OR MH "Cultural Bias" OR MH "Prejudice" OR MH "Empathy" OR MH "Emotional Intelligence" OR "Clinical interactions" OR "clinician-patient" OR "patient-clinician" OR "interaction*" OR "Interpersonal" OR relations* OR "Physician-Patient Interaction" OR "Nurse-Patient Interaction" OR "Professional-patient interaction" OR "Patient-practitioner Relations" OR "Clinician-Patient Relations" OR "Clinician-Patient Relationship" OR "relationship-based care" OR "Nurse-Patient Relations" OR "Physician-Patient Relations" OR "Professional-Patient Relations" OR "relationship-centered" OR "client-centered" OR "patient-centered" OR "person-centered" OR "Social Skills" OR "Social Perception" OR "Sociological Factors" OR "Patient Participation" OR "Communication" OR "Communicate" OR advocacy OR advocate OR "nonverbal" OR "Communication Barriers" OR "Verbal Behavior" OR "word use" OR "linguistics" OR "discourse" OR "language use" OR "Attitude of Health Personnel" OR "Helping Behavior" OR "Decision Making" OR "Mirror neurons" OR "Mirroring" OR "Theory of mind" OR "Sensitivity" OR "empathy" OR "empathy" OR "Clinical Empathy" OR "Empathic connection" OR "understanding" OR "emotion" OR "emotional understanding" OR "Emotional Intelligence" OR "belonging" OR "exchange" OR "attachment" OR "role taking" OR "person perception" OR "emotional intelligence" OR "social skills" OR "empathic behavior" OR "interactive competence" OR "social factors" OR "social norms" OR "social rules" OR "affect" OR "affectivity" OR "affective" OR "self-awareness" OR "similarity" OR "familiarity" OR "affection" OR "sympathy" OR "compassion" OR "symbolic interactionism" OR "perspective taking" OR "Responsiveness" OR "responsive" OR "responsiveness" OR "responsivity" OR "response" OR "rapport" OR "Gaze cueing" OR "Mental imagery" OR "Mental rotation" OR "Perspective-taking" OR "Social cognition" OR "Visual cognition" OR "culturally competent care" OR "culturally appropriate care" OR "cultural competence" OR "racial discrimination" OR "social discrimination" OR "racial prejudice" OR "social marginalization" OR "racial segregation" OR "social segregation" OR "racism" OR "racism" OR "prejudice" OR "Racial Bias" OR "Race Relations" OR "unconscious bias" OR "bias" OR "attitude" OR "implicit bias" OR "latent bias" OR "psychological power" OR "power" OR "Mistrust" OR "Trust" OR "Self-Disclosure" OR "Discourse Analysis" OR "face-to-face" OR "gestures" OR "Facial Displays" OR "Facial Expressions" OR "Cues" OR “eye contact” OR "Patient-Centered*" OR "Person-Centered*") | ("Physician-Patient Relations" OR DE "Racial and Ethnic Attitudes" OR DE "Racial and Ethnic Relations" OR DE "Racism" OR "Cultural Bias" OR DE "Cultural Competence" OR DE "Prejudice" OR DE "Empathy" OR DE "Emotional Intelligence" OR "Clinical interactions" OR "clinician-patient" OR "patient-clinician" OR "interaction*" OR "Interpersonal" OR relations* OR "Physician-Patient Interaction" OR "Nurse-Patient Interaction" OR "Professional-patient interaction" OR "Patient-practitioner Relations" OR "Clinician-Patient Relations" OR "Clinician-Patient Relationship" OR "relationship-based care" OR "Nurse-Patient Relations" OR "Physician-Patient Relations" OR "Professional-Patient Relations" OR "relationship-centered" OR "client-centered" OR "patient-centered" OR "person-centered" OR "Social Skills" OR "Social Perception" OR "Sociological Factors" OR "Patient Participation" OR "Communication" OR Communicate OR advocacy OR advocate OR "nonverbal" OR "Communication Barriers" OR "Verbal Behavior" OR "word use" OR "linguistics" OR "discourse" OR "language use" OR "Attitude of Health Personnel" OR "Helping Behavior" OR "Decision Making" OR "Mirror neurons" OR "Mirroring" OR "Theory of mind" OR "Sensitivity" OR "empathy" OR "empathy" OR "Clinical Empathy" OR "Empathic connection" OR "understanding" OR "emotion" OR "emotional understanding" OR "Emotional Intelligence" OR "belonging" OR "exchange" OR "attachment" OR "role taking" OR "person perception" OR "emotional intelligence" OR "social skills" OR "empathic behavior" OR "interactive competence" OR "social factors" OR "social norms" OR "social rules" OR "affect" OR "affectivity" OR "affective" OR "self-awareness" OR "similarity" OR "familiarity" OR "affection" OR "sympathy" OR "compassion" OR "symbolic interactionism" OR "perspective taking" OR "Responsiveness" OR "responsive" OR "responsiveness" OR "responsivity" OR "response" OR "rapport" OR "Gaze cueing" OR "Mental imagery" OR "Mental rotation" OR "Perspective-taking" OR "Social cognition" OR "Visual cognition" OR "culturally competent care" OR "culturally appropriate care" OR "cultural competence" OR "racial discrimination" OR "social discrimination" OR "racial prejudice" OR "social marginalization" OR "racial segregation" OR "social segregation" OR "racism" OR "racism" OR "prejudice" OR "Racial Bias" OR "Race Relations" OR "unconscious bias" OR "bias" OR "attitude" OR "implicit bias" OR "latent bias" OR "psychological power" OR "power" OR "Mistrust" OR "Trust" OR "Self-Disclosure" OR "Discourse Analysis" OR "face-to-face" OR "gestures" OR "Facial Displays" OR "Facial Expressions" OR "Cues" OR “eye contact” OR "Patient-Centered*" OR "Person-Centered*") |
| #3 (Comparison/ control): Patients identified as White | ((“Social”[TW] OR “Racial”[TW] OR “Interpersonal”[TW] OR “clinician-patient”[TW] OR “patient-clinician”[TW] OR “dyad”[TW]) AND (“Concordance”[TW] OR “Discordance”[TW])) OR (“Discrimination” OR “Racism”[MH] OR “Racism”[TW] OR "healthcare disparities"[MAJR] OR “disparity”[TW] OR “disparities”[TW] OR “equality”[TW] OR “equity”[TW] OR “equitable”[TW] OR “inequality”[TW] OR “inequity”[TW] OR “inequitable”[TW] OR "unequal"[TW] OR "othering"[TW] OR "Health Status Disparities"[Mesh] OR "Health care inequality"[TW]) | TS= (“Social Discordance” OR “Racial Discordance” OR “Interpersonal Discordance” OR “Clinician-Patient Discordance” OR “Patient-Clinician Discordance” OR “Dyad Discordance” OR “Social Concordance” OR “Racial Concordance” OR “Interpersonal Concordance” OR “clinician-patient Concordance” OR “patient-clinician Concordance” OR “Dyad Concordance” OR “Discrimination” OR “Racism” OR "healthcare disparities" OR disparity OR disparities OR equality OR equity OR inequality OR inequity OR inequitable OR equitable OR "Health Care Disparities" OR “Health care inequality”) | (‘Social Discordance’ OR ‘Racial Discordance’ OR ‘Interpersonal Discordance’ OR ‘Clinician-Patient Discordance’ OR ‘Patient-Clinician Discordance’ OR ‘Dyad Discordance’ OR ‘Social Concordance’ OR ‘Racial Concordance’ OR ‘Interpersonal Concordance’ OR ‘clinician-patient Concordance’ OR ‘patient-clinician Concordance’ OR ‘Dyad Concordance’) OR ('discrimination'/exp OR 'racism'/exp/mj OR 'health care disparity'/exp OR 'health disparity'/exp OR 'health care quality'/exp OR ‘healthcare disparities’ OR ‘disparity’ OR ‘disparities’ OR ‘difference*’ OR ‘equality’ OR ‘equity’ OR ‘inequality’ OR ‘inequity’ OR ‘inequitable’ OR ‘equitable’ OR ‘Health Status Disparities’ OR ‘Health care inequality’) | (“Social Discordance” OR “Racial Discordance” OR “Interpersonal Discordance” OR “Clinician-Patient Discordance” OR “Patient-Clinician Discordance” OR “Dyad Discordance” OR “Social Concordance” OR “Racial Concordance” OR “Interpersonal Concordance” OR “clinician-patient Concordance” OR “patient-clinician Concordance” OR “Dyad Concordance”) OR (MH "Healthcare Disparities" OR "healthcare disparities" OR disparity OR disparities OR difference* OR equality OR equity OR inequality OR inequity OR inequitable OR equitable OR "Health Status Disparities" OR “Health care inequality” OR "Quality of Health Care" OR “Discrimination” OR “Racism”) | (“Social Discordance” OR “Racial Discordance” OR “Interpersonal Discordance” OR “Clinician-Patient Discordance” OR “Patient-Clinician Discordance” OR “Dyad Discordance” OR “Social Concordance” OR “Racial Concordance” OR “Interpersonal Concordance” OR “clinician-patient Concordance” OR “patient-clinician Concordance” OR “Dyad Concordance”) OR (DE "Health Disparities" OR "healthcare disparities" OR disparity OR disparities OR difference* OR equality OR DE "Equity" OR equity OR inequality OR inequity OR inequitable OR equitable OR "Health Status Disparities" OR “Health care inequality” OR "Quality of Health Care" OR “social justice” OR “discrimination” OR “racism”) |
| #4 (Outcome): Processes or experience | ("Patient Reported Outcome Measures"[Mesh] OR "Patient Satisfaction"[MH] OR "Patient Experience"[TW] OR "process assessment, health care"[MH] OR "process measures"[TW] OR quality[TW] OR "patient-reported experience"[TW] OR "EDPEC"[TW] OR “ED Experience”[TW] OR "Discourse Analysis"[TW] OR "Interaction Analysis"[TW] OR “Time*”[TW] OR “Wait time”[TW] OR “Waited”[TW] OR “waiting”[TW] OR (“patient”[TW] AND "Experience*”[TW]) OR (“patient”[TW] AND perception*[TW]) OR (patient[TW] AND satisfaction[TW])) | TS=("Patient Reported Outcome Measures" OR "Patient Satisfaction" OR "Patient Experience" OR "process measures" OR quality OR "patient-reported experience" OR "EDPEC" OR “Emergency Department Patient Experience of Care” OR "Discourse Analysis" OR "Interaction Analysis" OR “Time*” OR “wait time” OR waited OR waiting OR (patient AND experience*) OR (patient AND perception*) OR (patient AND satisfaction)) | ('patient satisfaction'/exp OR 'patient-reported outcome'/exp OR 'patient experience'/exp OR ‘process assessment’ OR ‘process measures’ OR ‘quality’ OR 'health care quality'/exp/mj OR ‘patient-reported experience’ OR ‘EDPEC’ OR ‘Discourse Analysis’ OR ‘Interaction Analysis’ OR ‘Time*’ OR ‘wait time’ OR ‘waited’ OR ‘waiting’ OR (‘patient’ AND ‘experience*’) OR (‘patient’ AND ‘perception*’) OR (‘patient’ AND ‘satisfaction’)) | (MH "Patient Satisfaction" OR "Patient Reported Outcome Measures" OR "Patient Satisfaction" OR "Patient Experience" OR "process measures" OR quality OR "patient-reported experience" OR "EDPEC" OR “Emergency Department Patient Experience of Care” OR "Discourse Analysis" OR "Interaction Analysis" OR Time* OR “wait time” OR waited OR waiting OR (patient AND experience*) OR (patient AND perception*) OR (patient AND satisfaction)) | (DE "Client Satisfaction" OR "Patient Satisfaction" OR "Patient Reported Outcome Measures" OR "Patient Experience" OR "process measures" OR quality OR "patient-reported experience" OR "EDPEC" OR “Emergency Department Patient Experience of Care” OR "Discourse Analysis" OR "Interaction Analysis" OR Time* OR “wait time” OR waited OR waiting OR (patient AND experience*) OR (patient AND perception*) OR (patient AND satisfaction)) |
| #5 (Setting/ Context): Emergency Department | ("ED"[TW] OR "Emergency Service, Hospital"[MH] OR "Emergency Medical Services"[Mesh:NoExp] OR “Emergency Care”[TW] OR "ER"[TW]) | TS=("Emergency Department" OR “Emergency Care” OR "Emergency Medical Services" OR "ER") | ('hospital emergency service'/exp/mj OR ‘Emergency Department’:ab,ti OR ‘ED’:ab,ti OR ‘Emergency Services’:ab,ti) | (MH "Emergency Service" OR “Emergency Department” OR "ED" OR "Emergency Care" OR "Emergency Medical Services" OR "ER”) | (DE "Emergency Medicine") OR "Emergency Service" OR “Emergency Department” OR "ED" OR "Emergency Care" OR "Emergency Medical Services") |
| #6 | #1 AND #2 AND #3 AND #4 AND #5 | | | | |
| #7 (Filters): English, after 2003 | Eng[lang] AND ("2004/01/01"[PDAT] : "2024"[PDAT]) | LA=(English) AND (PY=(2004-2024)) | [english]/lim AND [2004-2024]/py | Limiters - Published Date: -20240101; English Language | Limiters - Published Date: -20240101; English Language |
| #8 | #6 AND #7 | | | | |

^a^ PICOS = Population, Intervention, Comparison/Control, Outcome, Setting/Context

^b^ Web of Science: Core Collection; “Apply equivalent subjects” unchecked

^c^ Embase; “Automatic mapping” unchecked

^d^ CINAHL; “Apply equivalent subjects” unchecked
