# Supplementary material for: Effects of patient race on processes and experiences of clinical interactions in US emergency departments: A mixed-methods systematic review
Source: PLoS One. 2025 Jun 25;20(6):e0325315. doi: 10.1371/journal.pone.0325315 (PMC12192141; doi:10.1371/journal.pone.0325315)
Supplement: S2 Table — Note: The QualSyst quantitative checklist has 14 items, three of which are not shown because they were not applicable to the reviewed studies. The Qualitative checklist has 10 items, all of which are shown. CI = clinical interaction. ª Publication year. b Item 11 not applicable to the qualitative data from Aysola et al., 2021. (DOCX) [file pone.0325315.s002.docx]

S2 Table. Quality assessment results organized by study focus: Experiences and processes of clinical interactions.

| First author, Year^a^ | Item 1: Purpose | Item 2: Design | Item 3: Sampling | Item 4: Variables | Item 5: Outcomes | Item 6: Sample | Item 7: Analysis | Item 8: Variance | Item 9: Confounding | Item 10: Results | Item 11: Conclusions | Score |
| --- | --- | --- | --- | --- | --- | --- | --- | --- | --- | --- | --- | --- |
| Group 1: Studies of observed CI processes | | | | | | | | | | | | |
| Aysola et al., 2021^b^ | Yes (2) | Yes (2) | Yes (2) | Partial (1) | Partial (1) | Partial (1) | Partial (1) | Yes (2) | Partial (1) | Yes (2) |  | 0.75 |
| Conteh et al., 2022 | Yes (2) | Partial (1) | Partial (1) | Yes (2) | Yes (2) | Yes (2) | Partial (1) | Yes (2) | Yes (2) | Yes (2) | Partial (1) | 0.82 |
| Schnitzer et al., 2020 | Yes (2) | Yes (2) | Yes (2) | Yes (2) | Partial (1) | Yes (2) | Yes (2) | Yes (2) | Yes (2) | Yes (2) | Yes (2) | 0.96 |
| Group 2: Studies of patient-reported CI experiences | | | | | | | | | | | | |
| Agarwal et al., 2022 | Partial (1) | Yes (2) | Yes (2) | Yes (2) | Partial (1) | Yes (2) | Yes (2) | Yes (2) | No (0) | Partial (1) | Partial (1) | 0.73 |
| Cornelius et al., 2018 | Yes (2) | Yes (2) | Partial (1) | Yes (2) | Partial (1) | Yes (2) | Yes (2) | Partial (1) | Yes (2) | Partial (1) | Partial (1) | 0.77 |
| Lee et al., 2008 | Partial (1) | Yes (2) | Partial (1) | Partial (1) | Partial (1) | Partial (1) | Yes (2) | Yes (2) | Yes (2) | Yes (2) | Yes (2) | 0.77 |
| Liyanage-Don et al., 2021 | Yes (2) | Yes (2) | Yes (2) | Yes (2) | Yes (2) | Yes (2) | Yes (2) | Yes (2) | Yes (2) | Yes (2) | Yes (2) | 1.00 |
| McCarthy et al., 2013 | Yes (2) | Yes (2) | Partial (1) | Partial (1) | Yes (2) | Partial (1) | Yes (2) | Yes (2) | Yes (2) | Partial (1) | Yes (2) | 0.82 |
| Parast et al., 2021 | Yes (2) | Yes (2) | Yes (2) | Yes (2) | Partial (1) | Yes (2) | Yes (2) | No (0) | Partial (1) | Partial (1) | Yes (2) | 0.77 |

*Note*: The QualSyst quantitative checklist has 14 items, three of which are not shown because they were not applicable to the reviewed studies. The Qualitative checklist has 10 items, all of which are shown. CI = clinical interaction

ª Publication year; ^b^ Item 11 not applicable to the qualitative data from Aysola et al., 2021.
