# Supplementary material for: Effects of patient race on processes and experiences of clinical interactions in US emergency departments: A mixed-methods systematic review
Source: PLoS One. 2025 Jun 25;20(6):e0325315. doi: 10.1371/journal.pone.0325315 (PMC12192141; doi:10.1371/journal.pone.0325315)
Supplement: S3 Table — Note: ED = emergency department; ER = emergency room; IPC = Interpersonal Processes of Care survey; ED CAHPS = Emergency Department Consumer Assessment of Healthcare Providers & Systems survey; CAT-T = Communication Assessment Tool for Teams. a Subdomains were only identified for the IPC scale. (DOCX) [file pone.0325315.s003.docx]

S3 Table. Themes from measures of patients' clinical interaction experiences.

| Scale/Source | Item (response options) | Author-Identified Domain (subdomain^a^) |
| --- | --- | --- |
| Theme: Responsive Nonverbal Communication | | |
| IPC | How often did doctors really find out what your concerns were? (0 – 5; 1=Never; 5=Always) | Communication (Elicited concerns, responded) |
| IPC | How often did doctors let you say what you thought was important? (0 – 5; 1=Never; 5=Always) | Communication (Elicited concerns, responded) |
| IPC | How often did doctors take your health concerns seriously? (0 – 5; 1=Never; 5=Always) | Communication (Elicited concerns, responded) |
| IPC | How often did doctors show concern about your feelings? (0 – 5; 1=Never; 5=Always) | Interpersonal Style (Compassionate, respectful) |
| IPC | How often did doctors really respect you as a person? (0 – 5; 1=Never; 5=Always) | Interpersonal Style (Compassionate, respectful) |
| IPC | How often did doctors treat you as an equal? (0 – 5; 1=Never; 5=Always) | Interpersonal Style (Compassionate, respectful) |
| IPC | How often did doctors discriminate against you because of your race or ethnicity? (0 – 5; 1=Never; 5=Always) | Interpersonal Style (Discrimination) |
| IPC | How often did doctors pay less attention to you because of your race or ethnicity? (0 – 5; 1=Never; 5=Always) | Interpersonal Style (Discrimination) |
| IPC | How often did office staff have a negative attitude toward you? (0 – 5; 1=Never; 5=Always) | Interpersonal Style (Disrespectful staff) |
| IPC | How often was office staff rude to you? (0 – 5; 1=Never; 5=Always) | Interpersonal Style (Disrespectful staff) |
| IPC | How often did office staff talk down to you? (0 – 5; 1=Never; 5=Always) | Interpersonal Style (Disrespectful staff) |
| IPC | How often did staff give you a hard time? (0 – 5; 1=Never; 5=Always) | Interpersonal Style (Disrespectful staff) |
| Agarwal et al., 2022 | Do you think your race played a role in any aspect of your experience in the ER? (Yes, in a positive way; Yes, in a negative way; No, it did not) | Impact of race on satisfaction |
| Agarwal et al., 2022 | Did you feel your race affected the quality of service you experienced? (0 – 5; 0 = Did Not Affect; 5 = Strongly Affected) | Impact of race on quality of care |
| Agarwal et al., 2022 | Did you feel your race affected how much respect you were treated with? (0 – 5; 0 = Did Not Affect; 5 = Strongly Affected) | Impact of race on respect |
| Agarwal et al., 2022 | Did you feel your race affected the communication between you and the care team? (0 – 5; 0 = Did Not Affect; 5 = Strongly Affected) | Impact of race on communication |
| ED CAHPS | How often did nurses treat you with courtesy and respect? (Never; Sometimes; Usually; Always) | Doctor & Nurse Communication |
| ED CAHPS | How often did nurses listen carefully to you? (Never; Sometimes; Usually; Always) | Doctor & Nurse Communication |
| ED CAHPS | How often did doctors treat you with courtesy and respect? (Never; Sometimes; Usually; Always) | Doctor & Nurse Communication |
| ED CAHPS | How often did doctors listen carefully to you? (Never; Sometimes; Usually; Always) | Doctor & Nurse Communication |
| Lee et al., 2008 | My doctor paid attention to what I had to say. (0 – 5; 0 = Strongly Disagree; 5 = Strongly Agree) | Affiliation |
| Lee et al., 2008 | My doctor respected me. (0 – 5; 0 = Strongly Disagree; 5 = Strongly Agree) | Affiliation |
| Lee et al., 2008 | My doctor was friendly and warm toward me. (0 – 5; 0 = Strongly Disagree; 5 = Strongly Agree) | Affiliation |
| Lee et al., 2008 | My doctor was patient with my questions or worries. (0 – 5; 0 = Strongly Disagree; 5 = Strongly Agree) | Affiliation |
| Lee et al., 2008 | If a mistake was made in my treatment, my doctor would try to hide it from me. (0 – 5; 0 = Strongly Disagree; 5 = Strongly Agree) | Trust |
| Lee et al., 2008 | My doctor sometimes pretends to know things when really not sure. (0 – 5; 0 = Strongly Disagree; 5 = Strongly Agree) | Trust |
| CAT-T | The medical team greeted me in a way that made me feel comfortable (1 – 5; 1 = Poor; 5 = Excellent) | Interpersonal & Communication Skills |
| CAT-T | The medical team treated me with respect (1 – 5; 1 = Poor; 5 = Excellent) | Interpersonal & Communication Skills |
| CAT-T | The medical team showed interest in ideas about my health (1 – 5; 1 = Poor; 5 = Excellent) | Interpersonal & Communication Skills |
| CAT-T | The medical team understood my main health concerns (1 – 5; 1 = Poor; 5 = Excellent) | Interpersonal & Communication Skills |
| CAT-T | The medical team paid attention to me (1 – 5; 1 = Poor; 5 = Excellent) | Interpersonal & Communication Skills |
| CAT-T | The medical team let me talk without interruptions (1 – 5; 1 = Poor; 5 = Excellent) | Interpersonal & Communication Skills |
| CAT-T | The medical team showed care and concern (1 – 5; 1 = Poor; 5 = Excellent) | Interpersonal & Communication Skills |
| CAT-T | The medical team spent the right amount of time with me (1 – 5; 1 = Poor; 5 = Excellent) | Interpersonal & Communication Skills |
| Theme: Effective Verbal Communication | | |
| IPC | How often did doctors speak too fast? (0 – 5; 1=Never; 5=Always) | Communication (Hurried communication) |
| IPC | How often did doctors use words that were hard to understand? (0 – 5; 1=Never; 5=Always) | Communication (Hurried communication) |
| IPC | How often did doctors explain your test results such as blood tests, x-rays, or other tests? (0 – 5; 1=Never; 5=Always) | Communication (Explained results, medications) |
| IPC | How often did doctors clearly explain the results of your physical exam? (0 – 5; 1=Never; 5=Always) | Communication (Explained results, medications) |
| ED CAHPS | How often did nurses explain things in a way you could understand? (Never; Sometimes; Usually; Always) | Doctor & Nurse Communication |
| ED CAHPS | How often did doctors explain things in a way you could understand? (Never; Sometimes; Usually; Always) | Doctor & Nurse Communication |
| ED CAHPS | Did the doctors or nurses ask about all of the medicines you were taking? (Yes; No) | Communication about medications |
| ED CAHPS | Before giving you any new medicine, did the doctors or nurses tell you what the medicine was for? (Yes Definitely; Yes Somewhat; No) | Communication about medications |
| ED CAHPS | Before giving you any new medicine, did the doctors or nurses describe possible side effects to you in a way you could understand? (Yes Definitely; Yes Somewhat; No) | Communication about medications |
| ED CAHPS | Did doctors and nurses give you as much information as you wanted about the results of … tests? (Yes Definitely; Yes Somewhat; No) | Receipt of sufficient information from doctors & nurses about test results |
| Lee et al., 2008 | I was kept well informed about delays in my care. (0 – 5; 0 = Strongly Disagree; 5 = Strongly Agree) | Satisfaction |
| CAT-T | The medical team gave me as much information as I wanted (1 – 5; 1 = Poor; 5 = Excellent) | Interpersonal & Communication Skills |
| CAT-T | The medical team talked in terms I could understand (1 – 5; 1 = Poor; 5 = Excellent) | Interpersonal & Communication Skills |
| CAT-T | The medical team checked to be sure I understood everything (1 – 5; 1 = Poor; 5 = Excellent) | Interpersonal & Communication Skills |
| CAT-T | The medical team encouraged me to ask questions (1 – 5; 1 = Poor; 5 = Excellent) | Interpersonal & Communication Skills |
| Theme: Person Centeredness | | |
| IPC | How often did the doctor involve you with decisions regarding your healthcare/medical treatment? (0 – 5; 1=Never; 5=Always) | Decision Making (Patient-centered decision making) |
| IPC | How often did the doctor give you different treatment options/choices? (0 – 5; 1=Never; 5=Always) | Decision Making (Patient-centered decision making) |
| ED CAHPS | Before you left the emergency room, did someone ask if you would be able to get this follow-up care? (Yes Definitely; Yes Somewhat; No) | Discussions with hospital staff about a patient’s ability to receive follow-up care |
| Lee et al., 2008 | My doctor listened to my wishes about my care. (0 – 5; 0 = Strongly Disagree; 5 = Strongly Agree) | Participation |
| Lee et al., 2008 | I had enough to say about my treatment. (0 – 5; 0 = Strongly Disagree; 5 = Strongly Agree) | Participation |
| Lee et al., 2008 | My doctor included me in decision-making about my care. (0 – 5; 0 = Strongly Disagree; 5 = Strongly Agree) | Participation |
| CAT-T | The medical team involved me in decisions as much as I wanted (1 – 5; 1 = Poor; 5 = Excellent) | Interpersonal & Communication Skills |
| CAT-T | The medical team discussed next steps, including any follow-up plans (1 – 5; 1 = Poor; 5 = Excellent) | Interpersonal & Communication Skills |
| Theme: Patient Satisfaction | | |
| ED CAHPS | When you first arrived at the emergency room, how long was it before someone talked to you about the reason why you were there? (< 5 Minutes; 5 -15 Minutes; > 15 Minutes) | Getting timely care |
| ED CAHPS | Did you get care within 30 minutes of getting to the emergency room? (Yes; No) | Getting timely care |
| ED CAHPS | Would you recommend this emergency room to your friends and family? (Definitely No; Probably No; Probably Yes; Definitely Yes) | Willingness to recommend the ED |
| Lee et al., 2008 | In terms of meeting my expectation for treatment and care, I am very satisfied with my overall care. (0 – 5; 0 = Strongly Disagree; 5 = Strongly Agree) | Satisfaction |
| Lee et al., 2008 | I am likely to recommend this ER to others. (0 – 5; 0 = Strongly Disagree; 5 = Strongly Agree) | Satisfaction |
| Lee et al., 2008 | If I have another problem requiring emergency care, I will return to the same ER. (0 – 5; 0 = Strongly Disagree; 5 = Strongly Agree) | Satisfaction |
| Agarwal et al., 2022 | How would you rate the experience from your most recent ER visit? (0 – 5; 5 = Excellent) | Satisfaction |

*Note*: ED = emergency department; ER = emergency room; IPC = Interpersonal Processes of Care survey; ED CAHPS = Emergency Department Consumer Assessment of Healthcare Providers & Systems survey; CAT-T = Communication Assessment Tool for Teams

^a^ Subdomains were only identified for the IPC scale
