# Supplementary material for: Effects of patient race on processes and experiences of clinical interactions in US emergency departments: A mixed-methods systematic review
Source: PLoS One. 2025 Jun 25;20(6):e0325315. doi: 10.1371/journal.pone.0325315 (PMC12192141; doi:10.1371/journal.pone.0325315)
Supplement: S1 File — (PDF) [file pone.0325315.s005.pdf]

To enable PROSPERO to focus on COVID-19 submissions, this registration record has undergone basic automated checks for eligibility and is published exactly as submitted. PROSPERO has never provided peer review, and usual checking by the PROSPERO team does not endorse content. Therefore, automatically published records should be treated as any other PROSPERO registration. Further detail is provided here.

Citation

Tommy Flynn, Kate Yeager, Bonnie Jennings. Racial Inequities in U.S. ED Clinical Interactions: A Systematic Review. PROSPERO 2021 CRD42021281653 Available from: https://www.crd.york.ac.uk/prospero/display_record.php?ID=CRD42021281653

Review question

We set out to answer the following research questions (RQ) about emergency department (ED) patient race and the quality of clinician-patient interactions (clinical interactions):

RQ-1: How do aspects of clinical interactions in EDs vary between patients identified as Black compared to patients identified as White; and

RQ-2: How do patients self-identified as Black experience clinical interactions differently from patients self-identified as White in U.S. EDs?

Answers to these questions will come from research on racial variability in observed or patient-reported measures of the quality of interpersonal clinical interactions between ED clinicians and patients.

Searches [1 change]

This review follows the standards described in the Preferred Reporting Items for Systematic Reviews and Meta-Analyses 2020 (PRISMA-2020) Statement (Page, McKenzie, et al., 2021) and Explanation and Elaboration (Page, Moher, et al., 2021). Our search strategy was developed using steps suggested by Bramer et al. (2018) and meets the guidelines established in the new PRISMA search strategy extension (PRISMA-S; Rethlefsen et al., 2021). We selected the following databases for searches to be conducted between September and October 2021: PubMed (National Library of Medicine), MEDLINE (Ovid), Web of Science Core Collection (Clarivate), Embase (Elsevier), PsycINFO (EBSCOhost), and CINAHL (EBSCOhost). A health sciences informationist (S.L.) with systematic review expertise reviewed the search strategies and translated queries to each databases. The PI will conduct the searches and export results. Additional literature sources include citation-chasing of referenced and citing sources for all included articles.

Search Terms:

We began the search-term selection process with words and phrases from open-source and published search strategies including the “MEDLINE®/PubMed® Health Disparities and Minority Health Search Strategy” (National Library of Medicine, 2019), the equity focused strategy from Prady et al. (2018), and searches from prior reviews on topics on, or related to, our research questions. Relevant research topics from existing systematic reviews include racial disparities in healthcare (Dehon et al., 2017; Health Services Research & Development Service et al., 2007; Lee et al., 2019; Owens et al., 2020; Shan et al., 2021), ED patient experience (Bull et al., 2021; Gordon et al., 2010; Sonis et al., 2018), and clinician-patient interactions (Graham & Smith, 2016; Wanko Keutchafo et al., 2020).

Search strategy

https://www.crd.york.ac.uk/PROSPEROFILES/281653_STRATEGY_20211027.pdf

Types of study to be included

The screening process and study selection are based on our research questions. We will include peer-reviewed articles reporting qualitative, quantitative, and mixed-method studies. Observational, interventional,

comparative effectiveness, experimental, and randomized controlled study types may be included. Literature reviews, editorials, commentaries, dissertations, theses, and articles reporting studies that used experimental vignettes (Sheringham et al., 2021) or simulations (Fasano et al., 2021) will be excluded. PICOS eligibility criteria will limit the inclusion of records to studies that (P) focus on care for patients perceived or self-identified as Black, (I) consider mechanisms, processes, or outcomes clinical interactions, (C) compare clinical interactions between Black and White adult (age >18 years) patients, (O) use observed or patient-reported data-generating methods, and (S) take place in a U.S. emergency department context.

Condition or domain being studied

Racial disparities in the observed (i.e., measured) or perceived (i.e., patient-reported) quality of interpersonal clinician-patient clinical interactions compared across Black and White patients.

Participants/population

ED Patients perceived or self-identified as Black

Intervention(s), exposure(s)

Interpersonal clinical interactions between clinicians and patients in the ED.

Comparator(s)/control

Patients perceived or self-identified as White.

Context

Studies conducted in hospital-based emergency department services in the United States.

Main outcome(s)

We are interested in the processes and patient-reported experiences of ED clinical interactions. Therefore, our main outcomes include measures of interpersonal aspects and patient experiences of interpersonal clinical interactions. Clinical interactions are extensively researched and represent the most commonly cited determinant of patient experience in the emergency department quality literature (Boudreaux & O'Hea, 2004; Byczkowski et al., 2016; Gordon et al., 2010; Parast et al., 2019; Sonis et al., 2018; Soremekun et al., 2011; Taylor & Benger, 2004; Tefera et al., 2016; Welch, 2010). Appropriate data for our purposes may be generated qualitatively (e.g., interview, focus group) quantitatively (e.g., observation, survey), or with mixed methods. Patient-reported measures may include (but are not limited to) the Emergency Department Consumer Assessment of Healthcare Providers and Systems (ED-HCAHPS) survey (Centers for Medicare & Medicaid Services, 2020; Roberts et al., 2020; Weinick et al., 2014); the Consultation and Relational Empathy (CARE) scale (Mercer, 2004). Similarly, observational methods may include interaction analysis (Brauner et al., 2018; Huber & Froehlich, 2020), linguistic analysis (Xu & Taylor, 2021), pragmatics (e.g., evaluation of nonverbal behavior, mimicry, listening; Blanch-Hartigan et al., 2018; Montague & Asan, 2014; Wright et al., 2012), proxemics (e.g., proximity to patient; McCall, 2017).

Measures of effect

Anticipated heterogeneity of research designs and methods preclude the need for measures of effect.

Additional outcome(s) [1 change]

Not applicable

Measures of effect

Not applicable

Data extraction (selection and coding)

Search results will be manually imported into the EndNote reference management software from Clarivate (Version 20.1). We will upload the resulting EndNote libraries to Covidence, a web-based systematic review application (Covidence, https://covidence.org). The subsequent screening process, quality assessment, and data extraction stages will use the Covidence platform. In stage two, the titles and abstracts of all database query results will be screened by the PI. All articles that meet eligibility criteria will be screened in full by two independent reviewers. The reviewers who evaluate the full text of articles will also assess their respective research quality to complete stages three and four simultaneously. All screening decisions will be explained

using predefined Covidence screening and data extraction documentation. Annotations explaining reviewers' inclusion and exclusion decisions will be recorded and summarily reported. The PI will then use EndNote to automatically import reference lists (see Bramer, 2018) from included studies. Citing articles of included studies will be identified using Dimensions (Free version, https://app.dimension.ai). Citations identified through reference lists and citing articles will then be screened for eligibility in the same way.

Data extraction will commence after articles are screened and final full-text selections have been identified from all sources. Two reviewers will extract data on study design, phenomena of interest (dependent variables), determining factors (independent variables), covariates and mediators, measures, contextual factors (e.g., U.S. geographic region) reported conflicts of interest, definitions and operationalizations of key terms (e.g., race, patient experience, etc.), research results, and all available PROGRESS-Plus variables including: place of residence, race/ethnicity, occupation, gender, religion, education, social capital, socioeconomic status, plus age, ability/disability, sexual orientation (Attwood et al., 2016; Prady et al., 2018).

Risk of bias (quality) assessment

The QualSyst scoring system was designed to evaluate the reliability (i.e., quality, risk of bias) of research findings from studies with varying methodological or philosophical traditions (Kmet et al., 2004). QualSyst summary score calculations indicate the degree to which articles meet quality criteria detailed in the methodology-concordant checklist (i.e., qualitative or quantitative).

The quantitative QualSyst checklist considers the following elements of quality research reporting: clearly defined research questions or objectives, appropriateness of design and methods, sampling, blinding, exposure and outcome measures, subject and comparison group characteristics, analytical methods, estimate of variance, approach to confounding, detail of results, and the logic of conclusions. Each of the 14 quantitative checklist items can be answered with a “yes,” “partial,” “no,” or “not applicable” (NA). “Yes” answers receive a score of two points, partials receive one point, no answers receive zero, and NAs are removed from the final calculation. The summary score is calculated as the total sum divided by the total possible sum (Kmet et al., 2004).

Strategy for data synthesis

We will conduct a detailed narrative synthesis of included studies.Grouping and coding of data will be based on each articles' primary concepts, measures, and QualSyst reliability assessment results. Articles measuring clinician empathy using the Consultation and Relational Empathy (CARE; Mercer, 2004), for example, would be grouped with studies using the same scale and adjacent to studies the same domain (i.e., clinician empathy) using a different measure (e.g., the Empathic Expressions Scale (EES; Suwinyattichaiporn et al., 2021).

Analysis of subgroups or subsets

We will conduct a detailed narrative synthesis of included studies.

Contact details for further information

Tommy Flynn
tommy.flynn@emory.edu

Organisational affiliation of the review

Emory University
www.emory.edu

Review team members and their organisational affiliations [1 change]

Mr Tommy Flynn. Emory University
Dr Kate Yeager. Emory University
Dr Bonnie Jennings. Emory University

Collaborators [1 change]

Dr Sharon Leslie. Emory University

Type and method of review [1 change]

Service delivery, Synthesis of qualitative studies, Systematic review

Anticipated or actual start date
01 September 2021

Anticipated completion date
18 December 2021

Funding sources/sponsors
NA

Conflicts of interest

Language
English

Country
United States of America

Stage of review
Review Ongoing

Subject index terms status
Subject indexing assigned by CRD

Subject index terms
MeSH headings have not been applied to this record

Date of registration in PROSPERO
25 October 2021

Date of first submission
24 September 2021

Stage of review at time of this submission [2 changes]

**Stage**	**Started**	**Completed**
Preliminary searches	Yes	Yes
Piloting of the study selection process	Yes	Yes
Formal screening of search results against eligibility criteria	Yes	Yes
Data extraction	No	No
Risk of bias (quality) assessment	No	No
Data analysis	No	No

The record owner confirms that the information they have supplied for this submission is accurate and complete and they understand that deliberate provision of inaccurate information or omission of data may be construed as scientific misconduct.

The record owner confirms that they will update the status of the review when it is completed and will add

publication details in due course.

Versions

25 October 2021
25 October 2021
27 October 2021
16 November 2021
