# Supplementary material for: Effects of patient race on processes and experiences of clinical interactions in US emergency departments: A mixed-methods systematic review
Source: PLoS One. 2025 Jun 25;20(6):e0325315. doi: 10.1371/journal.pone.0325315 (PMC12192141; doi:10.1371/journal.pone.0325315)
Supplement: S2 File — (PDF) [file pone.0325315.s006.pdf]

Study ID	Article ID	First Author	Year Published	Title	Objective or Aims	Research Question or Hypothesis	Methods	Design	Definition of Race	Definition of Ethnicity	Race_BLK_Def	Race_WHT_Def	Race_Notes	Region
**Lee 2008**	#244	Lee	2008	Association between patient race/ ethnicity and perceived interpersonal aspects of care in the emergency department	To determine the effects of patient race on perceptions of clinicians' interpersonal behaviors including affiliation, satisfaction, trust, and participation.		Quantitative	Quantitative cross-sectional survey		self reported with race and ethnicity as one category				South
**McCarthy 2013**	#806	McCarthy	2013	Emergency department team communication with the patient: The patient's perspective	To describe patients' perceptions of communication with the medical team (primary), and to evaluate associations between patient demographics, ED operational metrics, and team communication (secondary).		Quantitative	Quantitative cross-sectional survey						Northeast
**Cornelius 2018**	#1606	Cornelius	2018	The presence of companions during emergency department evaluation and its impact on perceptions of clinician-patient communication	To examine whether the presence of companions in the ED affects ACS patient perceptions of clinician-patient communication, and to assess the effects of companion closeness (secondary).	"We hypothesized that patients would perceive strongest communication when close others were present. We further explored whether effects differed across demographic factors associated with healthcare disparities (ie, race/ethnicity, English as a first language and education)" (p 701).	Quantitative	Quantitative observational cohort study						Northeast
**Parast 2021**	#2481	Parast	2021	Racial/Ethnic Differences in Emergency Department Utilization and Experience	To examine racial/ethnic differences in self-reported reasons for ED utilization, ED utilization in the past 6 months, and reported sources of usual care (primary); and examine [racial/ethnic] differences ED experiences of care in terms of including timeliness, communication about medications, and follow-up care. In addition, we investigated whether racial/ethnic		Quantitative	Quantitative cross-sectional survey			Patients who self-identified as "not Spanish/Hispanic/ Latino" and no racial groups other than "Black or African American" were considered Black.	Patients who self-identified as "not Spanish/Hispanic/ Latino" and no racial groups other than "White" were considered White.	All patients who self-identified with racial categories other than non-Hispanic Black or non-Hispanic White were considered "other."	West; Midwest; South; Northeast
**Liyanage-Don 2021**	#1606	Liyanage-Don	2021	Associations between emergency department crowding and perceptions of interpersonal care in patients presenting with suspected acute coronary syndrome	Determine whether ED crowding is associated with poorer perceptions of interpersonal care among patients with suspected ACS		Quantitative	Quantitative observational cohort study (secondary data analysis)						Northeast
**Agarwal 2022**	#3188	Agarwal	2022	Assessing experiences of racism among Black and White patients in the emergency department	tested a novel approach for assessing the experience of patients discharged from the emergency department (ED) to explore and understand the experience of racism in healthcare	When, where, and how experiences of racism in healthcare are happening?	Quantitative	Observational cross-sectional survey			"those identifying as Black or White"	"those identifying as Black or White"		Northeast
**Schnitzer 2020**	#2227	Schnitzer	2020	Disparities in Care: The Role of Race on the Utilization of Physical Restraints in the Emergency Setting	To determine the role of race in emergency physical restraint" (p 944).	We hypothesized that patients who identify as Black or African American would be more likely than patients who identify as white to undergo physical restraint in the ED.	Quantitative	Quantitative retrospective chart analysis					Race was "patient self-selected at [ED] registration" and extracted from medical records	Northeast
**Aysola 2021**	#2290	Aysola	2021	Understanding Contributors to Racial/ Ethnic Disparities in Emergency Department Throughput Times: a Sequential Mixed Methods Analysis	To characterize contributors to observed racial/ethnic disparities in ED throughput times		Mixed (both qualitative and quantitative)	Sequential explanatory mixed-methods study			Non-Hispanic Black	Non-Hispanic White	Research assistants asked participants to confirm their race/ethnicity noted in their patient records or provide their race/ ethnicity if a community member	Northeast

Study ID	Article ID	First Author	Year Published	Title	Objective or Aims	Research Question or Hypothesis	Methods	Design	Definition of Race	Definition of Ethnicity	Race_BLK_Def	Race_WHT_Def	Race_Notes	Region
**Conteh 2023**	#3140	Conteh	2023	Disparities in the Use of Chemical and Physical Restraints in the Emergency Department by Race/Ethnicity	To evaluate the relationships between race/ethnicity and the application of chemical compared to physical restraints among ED patients with acute agitation and aggression who received restraints	Does the use of chemical vs. physical restraints differ by race and/or ethnicity in this large US national cohort of ED patients presenting with agitation and/or aggression, and are there differences in time to application of restraints or differences in morbidity or mortality rates.	Quantitative	Retrospective observational cohort study		Hispanic was a category of race/ethnicity	"Black"	"White"		West; Midwest; South; Northeast

Study ID	Facility	Sample Size	Eligibility	Race/Ethnicity Source	CI Process vs Experience	Key concepts ,	CI Concepts/ Constructs	KC_1 KC Variable Type	Definitions of CI Concepts	Measures of CI Concepts	KC_1 KC_Notes
**Lee 2008**	Academic	430	Included: Adult ED patients; ;Excluded: Non-English speaking, altered mental status, level 1 acuity level.	Self-reported	Experience	Interpersonal aspects of care: Affiliation, Satisfaction, Trust, and Participa	Interpersonal aspects of care: Affiliation, Satisfaction, Trust, and Participation	Dependent	Affiliation: patients' perception of shared identity with clinicians;<br>Satisfaction: degree to which care meets or exceeds patients' expectations;<br>Trust: patients' beliefs about clinicians' motivations and responsiveness to patient vulnerability;<br>Participation: patients' perceived involvement in clinical decision-making	Four Likert style (1 = low, 5 = high) surveys:<br>Affiliation scale with four items (Chronbach's alpha 0.93); Satisfaction scale with four items (Chronbach's alpha 0.84); Trust scale with two items (Chronbach's alpha 0.67); and Participation scale with four items (Chronbach's alpha 0.85).	(p 81)
**McCarthy 2013**	Academic; Urban	226	ED patients over age 18 years; Excluded mental health complaints, critically ill or physically unstable patients, non-English speaking, or in police custody	Self-reported	Experience	Team communication, Operational characteristics	Team communication	Dependent	Team communication: No explicit definition;<br>Operational care: ED disposition, wait time, time from triage to physician evaluation, total ED length of stay, and ED daily census	The Communication Assessment Tool for Teams (CAT-T), verbally administered at the end of the ED encounter, is a Likert-style scale used to assess team communication tasks considered important to patients and clinicians. Medical team referred to "All those who have taken care of you in the ED today" (p 263).	"The CAT was adapted [from a previously validated instrument] for team environments (CAT-T) by making minor changes to the instructions and question item-stems to broaden the focus of assessment from a single doctor to the medical team." The CAT-T "assesses key attributes of communication that are important to patients and are deemed by physicians
**Cornelius 2018**	Academic; Urban	876	Adult ED patients with ACS, English or Spanish speaking; Excluded: terminal illness (non-cardiac), mental illness, cognitive impairment, alcohol or substance abuse; unavailable for follow up;	Self-reported	Experience	Doctor-patient communication, partner status	Doctor-patient communication	Dependent	Strong communication was "characterized by clarity, concern, and collaboration" (p 701).	Doctor-patient communication was measured using the 14 "doctor-specific" Likert-style items (1 = never, 5 = always) from the Interpersonal Processes of Care survey.	The phrase "doctor-patient" and "clinician-patient" were used interchangeably in reference to communication; the former appeared twice in the article and the latter appeared 28 times.; Definition in first line of text: Strong clinician,patient communication, characterized by clarity, concern and collaboration, is associated with patient satisfaction and
**Parast 2021**	Academic; Private; Public; Urban; Rural	3122	Included: Adults ≥ 18 years<br>Excluded: Primary mental health or substance abuse diagnosis, died in the ED, transferred from outside facility, admitted to hospital, or discharged to an	Self-reported	Experience		Patient experience	Dependent	Six dimensions of patient experience were assessed: getting timely care, communication with doctors and nurses, communication about medications, communication about test results, communication about follow-up care, and willingness to recommend the ED to others (excluded from analysis).	The ED CAHPS (formerly "EDPEC DTC") survey consists of six measures as follows: three composite measures for 1) getting timely care, 2) doctor and nurse communication, and 3) communication about medications; and three single-item measures of 4) receipt of sufficient information from doctors and nurses about test results, 5) discussions with hospital staff about ability to receive follow-up care, and 6) willingness to recommend the ED.	"top-box scores, in which the best or most positive response option was coded as 100and all other response options were coded as 0";<br>The "EDPEC" is now called the "ED CAHPS¬Æ"
**Liyanage-Don 2021**	Academic; Urban	933	Adult ED patients with ACS (unstable angina or non-ST-elevation myocardial infarction), English or Spanish speaking; Excluded: Patients with terminal non-cardiovascular illness, severe mental illness, cognitive	Self-reported	Experience		Interpersonal care	Independent	Psychosocial aspects of patient-physician interactions (i.e., interpersonal care) include effective communication, patient-centered decision-making, and interpersonal style.	The Interpersonal Processes of Care (IPC) survey assesses three domains: communication, patient-centered decision-making, and interpersonal style. Each IPC domain comprises subdomains of 2–4 Likert-style (1 = never, 5 = always) items.	
**Agarwal 2022**	Academic; Urban	462		Self-reported	Experience		Patient experience	Dependent	Perceived impact of race on ED care	Likert scale: 0 to 5 (0 = not affected, 5 = strongly affected)	How strongly did patients' race affect the overall care experience
**Schnitzer 2020**	Academic; Urban	195092	All adult ED visits with any chief complaint	Self-reported	Process		Restraint use	Independent	Restraint is defined as any manual, physical, or mechanical device; material; or equipment that immobilizes or reduces the ability to move the arms, legs, body, or head freely" (p 945).	Restraint order in the medical record	Race categories included American Indian or Alaskan Native, Asian, Black, Hispanic or Latino, white, other, and unavailable. ‚ÄúHispanic or Latino,Äù is included during patient registration in both self-selected race and ethnicity fields.
**Aysola 2021**	Academic; Urban	19	Eligibility unreported for qualitative arm	Self-reported race for quantitative portion and perceived patient race for qualitative component	Process		Provider [clinician]-patient interactions		Qualitative element focused on data pertaining to clinician behaviors and clinical interactions, specifically, clinicians' communications with and about patients.	Four qualitative data sources included 1) semi-structured interviews with patients and community members; 2) general observations of the ED; 3) observations of specific ED physicians; followed by 4) semi-structured interviews of observed physicians.	

Study ID	Facility	Sample Size	Eligibility	Race/Ethnicity Source	CI Process vs Experience	Key concepts ,	CI Concepts/ Constructs	KC_1 KC Variable Type	Definitions of CI Concepts	Measures of CI Concepts	KC_1 KC_Notes
**Conteh 2023**	Private	12229		Self-reported from EHR	Process			Dependent	chemical restraints; Defined as receiving any benzodiazepine, ketamine, anti-histamine, and/or anti-psychotic medication by intravenous, intramuscular, or oral route while in the ED	Databased EHR data	

Study ID	Other variables, covariates, or concepts	KC_2 KC Variable Type	Definitions and measures	KC_2 KC_Measure	KC_2 KC_Notes	Covariates	KC_3 KC Variable Type	KC_3 KC_Definition	KC_3 KC_Measure	KC_3 KC_Notes
**Lee 2008**	Age, gender [sex], household income, insurance, healthcare utilization, and ED referral	Dependent	Healthcare utilization included patient-reported visits to Eds or clinics in the preceding 6 months.<br>ED referral source options included the following: patient self-referral; telephone referral from insurance company, hospital nurse, or physician office; or direct referral after a physician office visit.		(p 81)		Dependent			(p 81)
**McCarthy 2013**	Patient demographics other than race included age and sex. Covariates included ED disposition, wait time, ED length of stay, and ED crowding	Covariates	ED disposition refers to patient status at the end of the ED visit, including hospital admission and discharge to community. Wait time was measured as the duration (in minutes) from triage to physician evaluation, and ED length of stay was the time (in minutes) from ED triage to ED departure. ED crowding was measured with ED daily census.							
**Cornelius 2018**	Partner status; Medical characteristics	Independent	Partner status indicated the presence of a "close other," "non-close other," or "no one" during the ED visit who could "serve as an advocate or help patients remember disposition plans and instructions" (p 701).<br>Patients' medical characteristics were presence of acute chest syndrome (ACS), Charlson Comorbidity Index, Global Registry of Acute Coronary Events (GRACE) risk score, and age.		"Three non-overlapping categories of companions were created: (1) bringing a close other to the ED (ie, spouse/partner and/or child); (2) bringing a non-		Other/Controls			
**Parast 2021**	Patient gender [sex], age, education, primary language, self-reported health, self-reported mental health, rural-urban commuting area, usual sources of care, healthcare utilization, reason for ED visit, mode of arrival, perceived importance of getting timely care, whether a proxy helped with survey completion, and response percentile.	Independent	Healthcare utilization was operationalized as visits to the ED or usual source of care in the past 6 months. Response percentile refers to the rank-ordered number of days between discharge date and end of data collection activities for a given respondent relative to all other respondents within an ED and specific survey mode.		All self-identified Hispanics were characterized as "Hispanic" in comparisons with "White" and "Black"					
**Liyanage-Don 2021**	ED Crowding	Dependent	The Emergency Department Work Index (EDWIN) is a validated measure incorporating patient volume and triage levels, physician staffing, and bed availability. EDWIN Scores < 1.5 indicate manageable ED crowding, scores 1.5 – 2 indicate a busy ED, and EDs with scores > 2 are considered overcrowded. EDWIN score was calculated on presentation and hourly for the duration of patients' ED stay.							
**Agarwal 2022**		Dependent	How strongly did patients' race affect the respect they experienced. 0 to 5 (0 = not affected, 5 = strongly affected)				Dependent	How strongly did patients' race affect the quality they experienced; 0 to 5 (0 = not affected, 5 = strongly affected)		
**Schnitzer 2020**	Patient age, sex, diagnosis, insurance, homelessness, and history of violence	Dependent								
**Aysola 2021**	NA		NA							

Study ID	Other variables, covariates, or concepts	KC_2 KC Variable Type	Definitions and measures	KC_2 KC_Measure	KC_2 KC_Notes	Covariates	KC_3 KC Variable Type	KC_3 KC_Definition	KC_3 KC_Measure	KC_3 KC_Notes
**Conteh 2023**		Dependent	Physical restraints; Defined as having any type of physical restraint applied at any time in the ED	Databased EHR data			Dependent	time to administration; Time from ED admission to first chemical restraint dose	Database EHR data	

Study ID	KC_4 KC_Term	KC_4 KC Variable Type	KC_4 KC_Definition	KC_4 KC_Measure	KC_4 KC_Notes	KC_5 KC_Term	KC_5 KC Variable Type	KC_5 KC_Definition	KC_5 KC_Measure	KC_5 KC_Notes	Year data collection started	Year data collection finished	Response rate or missing data	Patients (n)	Patients (%)	Clinicians (n)
**Lee 2008**		Dependent			(p 81)						2004	2004	74.0% response rate (n = 430), but the reported sample reported was less (n = 372).	372	100.00	0
**McCarthy 2013**		Covariates									2007	2008	Response rate was 65.3% including 44 take-home surveys	226	100.00	0
**Cornelius 2018**	Demographics	Other/Controls	Age, sex								2013	2016	40% refusal rate and 12.4% excluded	876	100.00	0
**Parast 2021**											2016	2016	20.25%	3122	100.00	0
**Liyanage-Don 2021**											2013	2016	93.3%	933	100.00	0
**Agarwal 2022**		Dependent	How strongly did patients' race affect the communication they experienced; 0 to 5 (0 = not affected, 5 = strongly affected)				Dependent	Overall care experience; 0-5, 5 = Excellent			2021	2021	13%	462	100.00	0
**Schnitzer 2020**											2016	2018	NA	120469	100.00	0
**Aysola 2021**											2017	2018	NA	10	52.60	9

Study ID	KC_4 KC_Term	KC_4 KC Variable Type	KC_4 KC_Definition	KC_4 KC_Measure	KC_4 KC_Notes	KC_5 KC_Term	KC_5 KC Variable Type	KC_5 KC_Definition	KC_5 KC_Measure	KC_5 KC_Notes	Year data collection started	Year data collection finished	Response rate or missing data	Patients (n)	Patients (%)	Clinicians (n)
**Conteh 2023**	number of doses of chemical restraint	Dependent	Total number of chemical restraint doses given	Databased EHR data		hospital length of stay	Independent	For admitted patients, total days in the hospital	Databased EHR data		2016	2019	NA	12229	100.00	0

Study ID	Sample Demographics:	Clinicians (%)	Black (n)	Black (%)	White (n)	White (%)	Hispanic (n)	Hispanic (%)	Other (n)	Other (%)	Age (mean, SD)	Female (n, %)	Male (n, %)	PROGRESS_Race	PROGRESS_Gender	PROGRESS_Age	PROGRESS_Education
**Lee 2008**		0.00	108	31.00	235	52.00	NA	NA	29	17.00	44.8 (17)	201 (?)	171 (?)	Yes	Yes	Yes	Yes
**McCarthy 2013**		0.00	92	40.70	109	48.20	NA	NA	25	11.10	45-64 (median)	53.5%	46.5%	Yes	Yes	Yes	No
**Cornelius 2018**		0.00	176	20.09	149	17.01	489	55.82	62	7.08	60.80 (12.95)	404 (46.12%)	472 (53.88%)	Yes	Yes	Yes	Yes
**Parast 2021**		0.00	372	12.00	2022	65.00	323	10.00	405	13.00	35-54 (median)	1926 (61.7%)	1196 (38.3%)	Yes	Yes	Yes	Yes
**Liyanage-Don 2021**		0.00	217	23.30	225	24.10	507	54.30	417	44.70	60.7 (13.1)	431 (46.2%)	502 (53.8%)	Yes	Yes	Yes	Yes
**Agarwal 2022**		0.00	277	59.95	143	30.95					42.9 (17.3)	310 (67.1)	137 (29.7)	Yes	Yes	Yes	No
**Schnitzer 2020**		0.00	19506	10.00	132256	67.80	2552	1.30	40778	20.90	50.3 (20.2)	93879 (48.1)	101205 (51.9%)	Yes	Yes	Yes	No
**Aysola 2021**	(1) semi-structured interviews with patients and community members (n=10); (2) general observations of the ED (conducted eight 120-min	47.40	5	50.00	5	50.00	NA	NA	NA	NA	NA	NA	NA	Yes	Yes	Yes	No

Study ID	Sample Demographics:	Clinicians (%)	Black (n)	Black (%)	White (n)	White (%)	Hispanic (n)	Hispanic (%)	Other (n)	Other (%)	Age (mean, SD)	Female (n, %)	Male (n, %)	PROGRESS_Race	PROGRESS_Gender	PROGRESS_Age	PROGRESS_Education
**Conteh 2023**		0.00	1833	15.0	8937	73.1	NA	NA	NA	NA	NA	NA	NA	Yes	Yes	Yes	No

Study ID	PROGRESS_SES	PROGRESS_PoR	PROGRESS_Sexuality	PROGRESS_Religion	PROGRESS_Occupation	PROGRESS_Culture	PROGRESS_Disability	Results	Conclusions	Explicit Limitations
**Lee 2008**	Yes	No	No	No	No	No	No	Black patient race predicted lower patient-reported trust, but not affiliation, participation, or satisfaction.	African-American patients demonstrated significantly lower mean scores for trust of healthcare providers than Caucasians and significantly lower levels of participation. African-American race/ethnicity continued to be a significant predictor of lower levels of trust (but not participation) after accounting for age, gender, education, household income, health insurance, healthcare received in last six months and route of referral to the ED.	Unable to determine the clinical meaning of the difference in trust scale results; single center study limits external validity; homogenous clinical and research staff prevented accounting for racial concordance; non-responder race was generated from research assistant perception.
**McCarthy 2013**	No	No	No	No	No	Bo	No	No association between patient-perceived communication and race	"Our multivariable analysis found no association be-tween patient demographic [or operational] factors and patient perceptions of communication" (p 266).	Convenience sampling and research assistants' potentially tacit exclusion of dissatisfied patients (sampling bias); in-person survey (social desirability bias);
**Cornelius 2018**	No	No	No	No	No	No	No	No association between patient-perceived interpersonal care and race.	Neither patient demographic variables nor medical characteristics were related with clinician-patient communication.	Self-reported patient experience data may not capture intended outcomes; Did not consider whether the presence of "family members" made a difference; Did not consider the effects of patient acuity.
**Parast 2021**	No	No	No	No	No	No	No	Black ED patients reported more positive experiences with nurse and doctor communication and medication-related communication compared to White ED patients.	"We found that Hispanic and Black ED patients reported higher ED utilization, more often lacked a usual source of care, and reported better experiences with ED care compared to White patients" (p 7).	Voluntary participation of emergency departments characterized as large and based in a hospital; low response rate; no information about past experiences of ED care, insurance status, staff race/ethnicity, or languages spoken by staff.
**Liyanage-Don 2021**	Yes	No	No	No	No	No	No	No association between patient-perceived interpersonal care and race.	No differences in admission or average EDWIN scores were found between patients with optimal versus suboptimal IPC scores. Higher admission EDWIN score was associated with modestly lower IPC score in both unadjusted (β=–1.70, 95% CI –3.15 to –0.24, p=0.02) and adjusted linear models (β=–1.77, 95% CI –3.31 to –0.24, p=0.02; (table 1 shows no diff in suboptimal IPC or optimal IPC by race)	Our study may have been underpowered to detect statistically significant associations between perceptions of interpersonal care and crowding later in the ED course. Other limitations include the cross-sectional design, lack of detailed data on ED utilization during the study period (eg, volume, visit time, patient disposition) and limited generalizability to patients without ACS.
**Agarwal 2022**	No	No	No	No	No	No	No	Black ED patients more likely to report positive ED experiences, although also more likely to perceive race as influencing that care.	The study had 4 key findings. First, we demonstrate the ability to engage patients to understand the intersection of their clinical care and race. Second, we find an early signal and key difference in how Black and White patients perceive the impact of their race on received ED care. Third, Black patients who report race negatively impacting their care cite respect, quality, and communication as significant manifestations of racism. Fourth, an overall positive patient ED care experience may mask the racism some patients experience.	Single system, nonresponse bias, "digital-divide," did not assess concordance, reliance on self-report, only Black and White patients
**Schnitzer 2020**	Yes	Yes	No	No	No	No	No	Black men were at an increased risk of physical restraint use compared to White men after accounting for insurance status, age, diagnosis, history of violence, and homelessness.	There was an overall effect of race on patient restraint (p<0.0001). This effect remained when controlling for sex, insurance, age, diagnosis, homelessness, and violence: there were no identified confounders.<br>The relative risk of restraint for Black patients was 1.37 (95% CI = [1.20, 1.57]), compared to 1.13 (95% CI = [1.07, 1.19]) for White patients.	Only patients' "first" reported race category was extracted from electronic medical records. Single site study.
**Aysola 2021**	No	No	No	No	No	No	No	Differences were qualitatively observed in the way White clinicians perceive, prioritize, and communicate with/about Black patients compared to White patients.	5 themes emerged from data synthesis: 1) Patient perceptions of ED wait time align by race/ethnicity, but recommendations differ; 2) Provider communications with or in reference to patients differ by race/ethnicity; 3) physicians differ in their approach to prioritizing patients; 4) physician communication styles and attitudes; 5) Physicians avoid discussing race/ethnicity.<br><br>"Our qualitative data suggest that the manner in which physicians, and healthcare personnel more generally,	1. sequential mixed methods design circumscribed sample size of physician interviews and targeted observations.<br>2. Determined race/ethnicity in observational data as perception versus self report.<br>3. No non-Hispanic Black physicians in cohort studied by direct observation.<br>4. Used ED timestamp data which has some evidence of lower precision than passive observations.

Study ID	PROGRESS_SES	PROGRESS_PoR	PROGRESS_Sexuality	PROGRESS_Religion	PROGRESS_Occupation	PROGRESS_Culture	PROGRESS_Disability	Results	Conclusions	Explicit Limitations
**Conteh 2023**	Yes	No	No	No	No	No	No	No statistically significant correlation between patient race and chemical vs physical restraint use	In this national ED study, there was no statistically significant correlation between Black or other race and the application of chemical compared to physical restraints among patients who presented to the ED with agitation or aggression. Black patients who received chemical or physical restraint and were subsequently admitted in our sample were less likely to experience in-hospital mortality compared to White patients (p=0.038) but were more likely to be discharged to another facility, i.e., rehabilitation or prison.	Can only determine correlation, not causation. Unable to determine context. Disparities in control variables may have led to underestimating the relationship. Missing data (race, sex, BMI) led to the exclusion of 4482 patients (22.9%).

Study ID	Other Limitations	Addressed Conflicts of interest	Author Race Reported	Summary Score	Results support CI racial [illegible]	Extractors
**Lee 2008**	Convenience sampling; patients were approached after the physician assessment & treatment plan were complete; measured constructs at a single time point; unvalidated measures adapted from several other questionnaires	No	No	0.77	Yes	Emily Burns, Tommy Flynn
**McCarthy 2013**		No	No	0.82	No	Kate Yeager, Tommy Flynn
**Cornelius 2018**		Yes	No	0.77	No	Kate Yeager, Tommy Flynn
**Parast 2021**	All patients who self-identified as Spanish/Hispanic/Latino ethnicity were categorized as Hispanic, regardless of self-identified race. All patients who self-identified as Asian, Native Hawaiian or Pacific Islander, or American Indian or Alaska Native, and without a reported race/ethnicity were classified as "Other." Patients	Yes	No	0.77	Yes	Kate Yeager, Tommy Flynn
**Liyanage-Don 2021**		Yes	No	1.00	No	Emily Burns, Tommy Flynn
**Agarwal 2022**	Did not define race, did not assess the perceived direction of the impact of race on care (whether race made it better or worse), did not use the word racism.	Yes	No	0.73	Yes	Kate Yeager, Tommy Flynn
**Schnitzer 2020**	Chart extraction of race data are known to be problematic for research use.	Yes	No	0.96	Yes	Kate Yeager, Tommy Flynn
**Aysola 2021**	Qualitative findings focused on physician-patient concordance, but all physicians sampled were White. No qualitative patient data were represented in the findings. Discordant interactions involved non-physician clinicians. No demographic information was reported for interviewees.	Yes	Yes	0.75	Yes	Emily Burns, Tommy Flynn

Study ID	Other Limitations	Addressed Conflicts of interest	Author Race Reported	Summary Score	Results support CI racial [illegible]	Extractors
**Conteh 2023**	Only included patients who had received either chemical or physical restraints.	Yes	No	0.82	No	Kate Yeager, Tommy Flynn
