# Supplementary material for: Effects of patient race on processes and experiences of clinical interactions in US emergency departments: A mixed-methods systematic review
Source: PLoS One. 2025 Jun 25;20(6):e0325315. doi: 10.1371/journal.pone.0325315 (PMC12192141; doi:10.1371/journal.pone.0325315)
Supplement: S4 Table — Note: Exclusion reasons were recorded only for articles screened by full text. (PDF) [file pone.0325315.s004.pdf]

S4 Table. All studies identified in the literature search

Covidence #	Title	Authors	Year	Journal	Volume	Issue	Pages	DOI	Author Year	Status	Notes
244	Association between patient race/ethnicity and perceived interpersonal aspects of care in the emergency department	Jin, S. L.; Tamayo-Sarver, J.; Kinneer, P.; Hobgood, C.	2008	Journal of the National Medical Association	100	1	79-85	10.1016/s0027-9684(15)31179-2	Lee 2008	Included	
806	Emergency department team communication with the patient: The patient's perspective	McCarthy, D. M.; Ellison, E. P.; Venkatesh, A. K.; Engel, K. G.; Cameron, K. A.; Makoul, G.; Adams, J. G.	2013	Journal of Emergency Medicine	45	2	262-270	10.1016/j.jemermed.2012.07.052	McCarthy 2013	Included	
1606	The presence of companions during emergency department evaluation and its impact on perceptions of clinician-patient communication	Cornelius, T.; Moise, N.; Birk, J. L.; Edmondson, D.; Chang, B. P.	2018	Emergency Medicine Journal	35	11	701-703	10.1136/emermed-2018-207735	Cornelius 2018	Included	

S4 Table. All studies identified in the literature search

1606	Associations between emergency department crowding and perceptions of interpersonal care in patients presenting with suspected acute coronary syndrome	Liyanage-Don, Nadia A; Edelman, David S; Chang, Bernard P; Schultebraucks, Katharina; Thanataveerat, Anusorn; Kronish, Ian M	2021	Emergency Medicine Journal	NA	NA	emermed-2020-210493	10.1136/emermed-2020-210493	Cornelius 2018	Included	
2227	Disparities in Care: The Role of Race on the Utilization of Physical Restraints in the Emergency Setting	Schnitzer, K.; Merideth, F.; Macias-Konstantopoulos, W.; Hayden, D.; Shtasel, D.; Bird, S.	2020	Academic Emergency Medicine	27	10	943-950	10.1111/acem.14092	Schnitzer 2020	Included	
2290	Understanding Contributors to Racial/Ethnic Disparities in Emergency Department Throughput Times: a Sequential Mixed Methods Analysis	Aysola, J.; Clapp, J. T.; Sullivan, P.; Brennan, P. J.; Higginbotham, E. J.; Kearney, M. D.; Xu, C.; Thomas, R.; Griggs, S.; Abdirisak, M.; Hilton, A.; Omole, T.; Foster, S.; Mamtani, M.	2021	Journal of General Internal Medicine	NA	NA	NA	10.1007/s11606-021-07028-5	Aysola 2021	Included	

S4 Table. All studies identified in the literature search

2481	Racial/Ethnic Differences in Emergency Department Utilization and Experience	Parast, L.; Mathews, M.; Martino, S.; Lehrman, W. G.; Stark, D.; Elliott, M. N.	2021	Journal of General Internal Medicine	NA	NA	NA	10.1007/s11606-021-06738-0	Parast 2021	Included	
3140	Disparities in the Use of Chemical and Physical Restraints in the Emergency Department by Race/Ethnicity	Conteh, E.; Alorda, A.; Lebowitz, D.; MacIntosh, T.	2023	Journal of racial and ethnic health disparities	NA	NA	NA	10.1007/s40615-022-01504-2	Conteh 2023	Included	
3188	Assessing experiences of racism among Black and White patients in the emergency department	Agarwal, A.K.; Sagan, C.; Gonzales, R.; Nijim, S.; Merchant, R.M.; Asch, D.A.; South, E.C.	2022	JACEP Open	3	6	NA	10.1002/emp2.12870	Agarwal 2022	Included	
813	Name calling in the emergency department: How do patients want to be addressed?	Minnick, N.; Nouhan, P. P.	2013	Academic Emergency Medicine	20	5	S153	10.1111/acem.12115	Minnick 2013	Excluded	Exclusion reason: Wrong study design or document type; Tommy [illegible]

S4 Table. All studies identified in the literature search

2556	Racial disparities in boarding in an urban academic emergency department	Tran, F.; Resnick-Ault, D.; Olson, K.; Carlson, N.; Michael, S. S.	2021	Academic Emergency Medicine	28	SUPP L 1	S263	10.1111/acem.14249	Tran 2021	Excluded	Exclusion reason: Wrong study design or document type; Tommy Flynn (2021-11-21 [illegible]
815	Association of patient, operational, and care factors with emergency department satisfaction in dental pain, back pain, and headache patients	Morgan, M.; Isenberger, K. M.; Lefevere, R. C.; Thomas, A. J.; Salzman, J. G.	2013	Annals of Emergency Medicine	62	4	S52	10.1016/j.annemergmed.2013.07.424	Morgan 2013	Excluded	Exclusion reason: Wrong study design or document type; Tommy Flynn (2021-11-21 21:42:48)(Excluded): Abstract;
1923	Significant differences in patient acuity assessments between emergency department patients and providers	Mace, S. E.; Fares, S.; Ryan, D.; Stoianoff, S.; Joiner, A. P.	2019	Academic Emergency Medicine	26	NA	S227	10.1111/acem.13756	Mace 2019	Excluded	Exclusion reason: Wrong study design or document type; Tommy Flynn (2021-11-21 21:41:56)(Excluded): Abstract;

S4 Table. All studies identified in the literature search

1079	Predictors of patient satisfaction with pain management in the emergency department	Fallon, E.; Rubal-Peace, G.; Patanwala, A.	2015	Pharmacotherapy	35	11	e268	10.1002/phar.1659	Fallon 2015	Excluded	Exclusion reason: Wrong study design or document type; Tommy Flynn (2021-11-21 21:23:16)(Excluded): Abstract;
2226	151 Analysis of Race and Sex Disparities in the Emergency Department	Schmeitzel, J. L.; Litzenberger, S.; Stoltzfus, J.; Melanson, S.; Stankewicz, H.	2020	Annals of Emergency Medicine	76	4	S59	10.1016/j.annemergmed.2020.09.163	Schmeitzel 2020	Excluded	Exclusion reason: Wrong study design or document type; Tommy Flynn (2021-11-21 21:23:06)(Excluded): Abstract;
697	Determinants of health care access and use in an ED population	Schumacher, J.; Hendry, P.; Hall, A.; Davis, T.; Arnold, C.; Kalynych, C.; Carden, D. L.	2012	Academic Emergency Medicine	19	NA	S215-S216	10.1111/j.1553-2712.2012.01332.x	Schumacher 2012	Excluded	Exclusion reason: Wrong study design or document type; Tommy Flynn (2021-11-21 [illegible]

S4 Table. All studies identified in the literature search

2168	Association between emergency department crowding and patient perceptions [illegible]	Liyanage-Don, N.; Edelman, D. S.; Chang, B.; Schultebraucks, K.; Thanataveerat, A.; Edmondson, D.; [illegible]	2020	Journal of General Internal Medicine	35	SUPPL 1	S32-S33	10.1007/s11606-020-05890-3	Liyanage-Don 2020	Excluded	Exclusion reason: Wrong study design or document type; Tommy Flynn (2021-[illegible]
314	Repetitive questions in the emergency department:	Ha, J.; Brickman, K.	2009	Annals of Emergency Medicine	54	3	S125	NA	Ha 2009	Excluded	Exclusion reason: Wrong study design or document
2115	Language discordance is associated with suboptimal patient-provider communication in the emergency department	Edelman, D. S.; Palmer, D.; Romero, E.; Chang, B.; Kronish, I.	2020	Journal of General Internal Medicine	35	SUPPL 1	S186	10.1007/s11606-020-05890-3	Edelman 2020	Excluded	Exclusion reason: Wrong study design or document type; Tommy Flynn (2021-11-20 04:26:27)(Select): Abstract; [illegible]
1208	Language used in medical records may reflect (unconscious) bias	Beach, M. C.; O'Conor, K.; Haywood, C.; Lanzkron, S.; Saha, S.	2016	Journal of General Internal Medicine	31	2	S290	NA	Beach 2016	Excluded	Exclusion reason: Wrong study design or document type; Tommy [illegible]
1489	Patient perceptions of physician empathy among emergency department patients [illegible]	Lin, M. P.; Probst, M. A.; Hall, C.; Neumann, D.; Butler, K.; Runyon, M. S.; Hess, E. P.; Kuehl, D. R.; Puskarich, M.; [illegible]	2017	Academic Emergency Medicine	24	NA	S76-S77	10.1111/acem.13203	Lin 2017	Excluded	Exclusion reason: Wrong study design or document type; Tommy Flynn (2021-11-17[illegible]

S4 Table. All studies identified in the literature search

1800	A mixed Methods analysis to evaluate emergency	Aysola, J.; Foster, S.; Mamtani, M.; Clapp, J.; Kearney, M.; Higginbotham, E.	2019	Journal of General Internal Medicine	34	2	S109-S110	10.1007/11606.1525-1497	Aysola 2019	Excluded	Exclusion reason: Wrong study design or document
1059	The relationships between patients' perceptions of	Bucco, Theresa	2015	NA	NA	NA	193 p-193 p	NA	Bucco 2015	Excluded	Exclusion reason: Wrong study design
793	Use of teachback discharge instructions does not improve patient satisfaction	Kinsella, Y.; Gross, M.; Shin, N.; Aginam, N.; Jones, S.; Carpenter, C. R.; Kaphingst, K.;	2013	Academic Emergency Medicine	20	5	S225	10.1111/acem.12115	Kinsella 2013	Excluded	Exclusion reason: Wrong study design or document type;
1191	Disparities in acute sepsis care: A systematic review	Yamane, D.; Huancahuari, N.; Hou, P.; Schuur, J.	2015	Critical Care	19	NA	S7-S8	10.1186/cc14102	Yamane 2015	Excluded	Exclusion reason: Wrong study design or document type;
1327	Racial and socioeconomic disparities in emergency Department care of	Sano, E. D.; Tichter, A. M.; Chang, B. P.; Suh, E. H.; Bhatt, B. A.	2016	Academic Emergency Medicine	23	NA	S208	10.1111/acem.12974	Sano 2016	Excluded	Exclusion reason: Wrong study design or document type;
1420	A Systematic Review of the Impact of Physician Implicit Racial Bias on Clinical Decision Making	Dehon, E.; Weiss, N.; Jones, J.; Faulconer, W.; Hinton, E.; Sterling, S.	2017	Acad Emerg Med	24	8	895-904	10.1111/acem.13214	Dehon 2017	Excluded	Exclusion reason: Wrong study design or document type;

S4 Table. All studies identified in the literature search

2122	231EMF Investigating Psychosocial Factors, Health Behaviors, and Diabetic Control in [illegible]	Flessel, A. E.; Reed, B.; Lovelace, A.; Levy, P.	2020	Annals of Emergency Medicine	76	4	S90	10.1016/j.annemergmed.2020.09.244	Flessel 2020	Excluded	Exclusion reason: Wrong study design or document type;
3014	Racial and Ethnic Disparities in Discharge Against Medical Advice	Tsai, J.; Janke, A.; Venkatesh, A.K.; Khidir, H.	2023	Academic Emergency Medicine	30	NA	136	10.1111/acem.14718	Tsai 2023	Excluded	Exclusion reason: Wrong study design or document
3018	Microaggressions and Discrimination Towards Patients During Emergency Department Care: A Mixed-Methods Study	Osuji, E.; Punches, B.; Bischof, J.J.; Li-Sauerwine, S.; Young, H.; Lyons, M.S.; Southerland, L.T.	2023	Academic Emergency Medicine	30	NA	206	10.1111/acem.14718	Osuji 2023	Excluded	Exclusion reason: Wrong study design or document type;
3026	Inequities in Queue Jumping in the Emergency Department Waiting Room	Sangal, R.; Su, H.; Khidir, H.; Parwani, V.; Liebhardt, B.; Ulrich, A.; Pinker, E.; Meng, L.; Venkatesh, A.K.	2023	Academic Emergency Medicine	30	NA	213	10.1111/acem.14718	Sangal 2023	Excluded	Exclusion reason: Wrong study design or document type;
3119	Racial Disparities in Emergency Restraint Use for Agitated Patients	Stone, D.	2023	Western Journal of Emergency Medicine	24	2.1	S3-S4	NA	Stone 2023	Excluded	Exclusion reason: Wrong study design or document type;

S4 Table. All studies identified in the literature search

2839	Emergency department care experiences among members of equity-deserving groups: quantitative results from a cross-sectional mixed methods study	Bartels, S.A.; MacKenzie, M.; Douglas, S.L.; Collier, A.; Pritchard, J.; Purkey, E.; Messenger, D.; Walker, M.	2023	BMC Emergency Medicine	23	1	NA	10.1186/s12873-023-00792-z	Bartels 2023	Excluded	Exclusion reason: Wrong Setting: Research conducted outside the United States ;
1238	Healthcare injustice in patients with sickle cell	Ezenwa, M. O.; Patil, C.; Shi, K.; Molokie, R. E.	2016	International Journal of Human	9	2	109-119	10.1108/IJHRH-07-2014-0015	Ezenwa 2016	Excluded	Exclusion reason: Wrong Setting: Non-
1610	Health literacy and quality of physician-trauma patient	Dameworth, J. L.; Weinberg, J. A.; Goslar, P. W.; Stout, D. J.; Israr, S.;	2018	J Trauma Acute Care Surg	85	1	193-197	10.1097/ta.0000000000001934	Dameworth 2018	Excluded	Exclusion reason: Wrong Setting: Non-ED Healthcare
2589	Disparities in physician care: Experiences and perceptions of a multi-ethnic America	Blendon, R. J.; Buhr, T.; Cassidy, E. F.; P√©rez, D. J.; Sussman, T.; Benson, J. M.; Herrmann, M. J.	2008	Health Affairs	27	2	507-517	10.1377/hlthaff.27.2.507	Blendon 2008	Excluded	Exclusion reason: Wrong Setting: Non-ED Healthcare Setting;

S4 Table. All studies identified in the literature search

585	What reasons do adolescents give for presenting to the emergency department over their primary care [illegible]	Weiss, A.; D'Angelo, L.; Tuchman, L.; Rucker, A.	2011	Journal of Adolescent Health	48	2	S39	10.1016/j.jadohealth.2010.11.087	Weiss 2011	Excluded	Exclusion reason: Wrong Population: Not adult ED patients;
843	Racial disparity in emergency department triage	Schrader, C. D.; Lewis, L. M.	2013	J Emerg Med	44	2	511-8	10.1016/j.jemermed.2012.05.010	Schrader 2013	Excluded	Exclusion reason: Wrong Intervention: Not about Clinical Interactions (i.e., face-to-face [illegible]
49	Patient concerns about medical errors in emergency	Burroughs, T. E.; Waterman, A. D.; Gallagher, T. H.; Waterman, B.;	2005	Academic Emergency Medicine	12	1	57-64	10.1197/j.aem.2004.08.052	Burroughs 2005	Excluded	Exclusion reason: Wrong Intervention: Not about
112	Disparities in analgesia and opioid prescribing practices for patients with musculoskeletal pain in the emergency [illegible]	Heins, J. K.; Heins, A.; Grammas, M.; Costello, M.; Huang, K.; Mishra, S.	2006	J Emerg Nurs	32	3	219-24	10.1016/j.jen.2006.01.010	Heins 2006	Excluded	Exclusion reason: Wrong Intervention: Not about Clinical Interactions (i.e., face-to-face [illegible]

S4 Table. All studies identified in the literature search

130	Alcohol-related problems: Emergency physicians' current practice and attitudes	O'Rourke, M.; Richardson, L. D.; Wilets, I.; D'Onofrio, G.	2006	Journal of Emergency Medicine	30	3	263-268	10.1016/j.jemermed.2005.05.031	O'Rourke 2006	Excluded	Exclusion reason: Wrong Intervention: Not about Clinical Interactions (i.e., face-to-face encounters between patients and healthcare
145	"Should I give you my smoking lecture now or later?" Characterizing emergency [illegible]	Vokes, N. I.; Bailey, J. M.; Rhodes, K. V.	2006	Annals of Emergency Medicine	48	4	406-414, 414.e1-4147	NA	Vokes 2006	Excluded	Exclusion reason: Wrong Intervention: Not about Clinical Interactions [illegible]

S4 Table. All studies identified in the literature search

341	Multicenter Study of Knowledge About Human Papilloma Virus and Attitudes Among Emergency Department Patients	Millen, J. C.; Ginde, A. A.; Anderson, A. T.; Fang, P.; Camargo Jr, C. A.	2009	Journal of Pediatric and Adolescent Gynecology	22	6	356-359	10.1016/j.jpag.2009.03.001	Millen 2009	Excluded	Exclusion reason: Wrong Intervention: Not about Clinical Interactions (i.e., face-to-face encounters between patients and healthcare professionals);
704	Patients with severe traumatic brain injury transferred to a Level i or II trauma center: [illegible]	Sugerman, D. E.; Xu, L.; Pearson, W. S.; Faul, M.	2012	Journal of Trauma and Acute Care Surgery	73	6	1491-1499	10.1097/TA.0b013e3182782675	Sugerman 2012	Excluded	Exclusion reason: Wrong Intervention: Not about Clinical Interactions [illegible]
711	Characteristics of frequent geriatric users of an urban emergency department	Wajnberg, A.; Hwang, U.; Torres, L.; Yang, S.	2012	Journal of Emergency Medicine	43	2	376-381	10.1016/j.jemermed.2011.06.056	Wajnberg 2012	Excluded	Exclusion reason: Wrong Intervention: Not about Clinical Interactions (i.e., face-to-face encounters between [illegible]

S4 Table. All studies identified in the literature search

716	Do Ethnic Differences Still Exist in Pain Assessment and Treatment in the Emergency [illegible]	Ware, L. J.; Epps, C. D.; Clark, J.; Chatterjee, A.	2012	Pain Management Nursing	13	4	194-201	10.1016/j.pmn.2010.06.001	Ware 2012	Excluded	Exclusion reason: Wrong Intervention: Not about Clinical Interactions [illegible]
718	How Low-Income Residents Decide Between Emergency and Primary Health Care for Non-Urgent Treatment	Wilkin, Holley A.; Cohen, Elizabeth L.; Tannebaum, Michael A.	2012	Howard Journal of Communications	23	2	157-174	10.1080/10646175.2012.667725	Wilkin 2012	Excluded	Exclusion reason: Wrong Intervention: Not about Clinical Interactions (i.e., face-to-face encounters [illegible]

S4 Table. All studies identified in the literature search

831	Low-dose ketamine analgesia: Patient and physician experience in the ED	Richards, J. R.; Rockford, R. E.	2013	American Journal of Emergency Medicine	31	2	390-394	10.1016/j.ajem.2012.07.027	Richards 2013	Excluded	Exclusion reason: Wrong Intervention: Not about Clinical Interactions (i.e., face-to-face encounters between patients and healthcare professionals);
1123	Demographic, operational, and healthcare utilization factors associated with emergency [illegible]	Morgan, M. W.; Salzman, J. G.; Le Fevere, R. C.; Thomas, A. J.; Isenberger, K. M.	2015	Western Journal of Emergency Medicine	16	4	516-526	10.5811/westjem.2015.4.25074	Morgan 2015	Excluded	Exclusion reason: Wrong Intervention: Not about Clinical Interactions [illegible]

S4 Table. All studies identified in the literature search

1259	Association of Insurance Status with Severity and Management in ED Patients with [illegible]	Hasegawa, K.; Stoll, S. J.; Ahn, J.; Kysia, R. F.; Sullivan, A. F.; Camargo, C. A.	2016	Western Journal of Emergency Medicine	17	1	22-27	10.5811/westjem.2015.11.28715	Hasegawa 2016	Excluded	Exclusion reason: Wrong Intervention: Not about Clinical
1735	Factors Associated With Patient Involvement in Emergency Care Decisions: A Secondary Analysis of the Chest Pain Choice Multicenter Randomized Trial	Probst, M. A.; Tschatscher, C. F.; Lohse, C. M.; Fernanda Bellolio, M.; Hess, E. P.	2018	Academic Emergency Medicine	25	10	1107-1117	10.1111/acem.13503	Probst 2018	Excluded	Exclusion reason: Wrong Intervention: Not about Clinical Interactions (i.e., face-to-face encounters between patients and
2086	Patient motivation for presenting to emergency department for vaginal bleeding in early pregnancy [illegible]	Bucher, J.; Mody, B.; Chin, M.; West, L.; Ohman-Strickland, P.; Joel, M.; Fastenau, J.; McCoy, J.	2020	American Journal of Emergency Medicine	38	5	1025-1030	10.1016/j.ajem.2019.10.011	Bucher 2020	Excluded	Exclusion reason: Wrong Intervention: Not about Clinical Interactions [illegible]
3660	Patient sex, racial and ethnic disparities in emergency department triage: A multi-site [illegible]	Patel, M. D.; Lin, P.; Cheng, Q.; Argon, N. T.; Evans, C. S.; Linthicum, B.; Liu, Y.; Mehrotra, A.; Murphy, L.; Ziya, S.	2023	American Journal of Emergency Medicine	76	NA	29-35	10.1016/j.ajem.2023.11.008	Patel 2023	Excluded	Exclusion reason: Wrong Intervention: Not about Clinical Interactions [illegible]

S4 Table. All studies identified in the literature search

2681	Resuscitating the physician-patient relationship: Emergency department communication in an academic medical center	Rhodes, K. V.; Vieth, T.; He, T.; Miller, A.; Howes, D. S.; Bailey, O.; Walter, J.; Frankel, R.; Levinson, W.	2004	Annals of Emergency Medicine	44	3	262-267	10.1016/j.annemergmed.2004.02.035	Rhodes 2004	Excluded	Exclusion reason: Wrong Comparator: No comparison between care of patients identified as [illegible]
514	"It's a wild thing, waiting to get me": Stance analysis of African Americans with diabetes	Davis, B. H.; Pope, C.; Mason, P. R.; Magwood, G.; Jenkins, C. M.	2011	Diabetes Educator	37	3	409-418	10.1177/0145721711404439	Davis 2011	Excluded	Exclusion reason: Wrong Comparator: No comparison
1062	Patient treatment in emergency department hallways and patient-physician [illegible]	Carter, E. J.; Edmondson, D.; Suh, E. H.; Bleck, R.; Oyola, D. A.; Underwood Iii, J. P.	2015	Psychosomatic Medicine	77	3	A106	NA	Carter 2015	Excluded	Exclusion reason: Wrong Comparator: No comparison [illegible]
1758	A Qualitative Analysis of Patients‚Äô Perceptions of Shared Decision [illegible]	Schoenfeld, E. M.; Goff, S. L.; Downs, G.; Wenger, R. J.; Lindenauer, P. K.; Mazor, K. M.	2018	Academic Emergency Medicine	25	7	716-727	10.1111/acem.13416	Schoenfeld 2018	Excluded	Exclusion reason: Wrong Comparator: No comparison
2381	A qualitative study of emergency department patients who survived an opioid [illegible]	Hawk, K.; Grau, L. E.; Fiellin, D. A.; Chawarski, M.; O‚ÄôConnor, P. G.; Cirillo, N.; Breen, C.; [illegible]	2021	Academic Emergency Medicine	28	5	542-552	10.1111/acem.14197	Hawk 2021	Excluded	Exclusion reason: Wrong Comparator: No comparison [illegible]

S4 Table. All studies identified in the literature search

2571	Improving perceptions of empathy in patients undergoing low-yield computerized tomographic imaging in the [illegible]	Lin, Michelle P.; Probst, Marc A.; Puskarich, Michael A.; Dehon, Erin; Kuehl, Damon R.; Wang, Ralph C.; Hess, Erik P.; Butler, Katie; Runyon, [illegible]	2018	Patient Education and Counseling	101	4	717-722	10.1016/j.pec.2017.11.012	Lin 2018	Excluded	Exclusion reason: Wrong Comparator: No comparison between care of patients identified as [illegible]
2704	Analysis of factors affecting satisfaction in the emergency department: A survey of 1019 patients	Topacoglu, H.; Karcioglu, O.; Ozucelik, N.; Ozsarac, M.; Degerli, V.; Sarikaya, S.; Cimrin, A. H.; Soysal, S.	2004	Advances in Therapy	21	6	380-388	10.1007/BF02850102	Topacoglu 2004	Excluded	Exclusion reason: Wrong Comparator: No comparison between care of patients identified as [illegible]
3155	Patient perceptions of microaggressions and discrimination toward patients during emergency department care	Punches, B.E.; Osuji, E.; Bischof, J.J.; Li-Sauerwine, S.; Young, H.; Lyons, M.S.; Southerland, L.T.	2023	Academic Emergency Medicine	NA	NA	NA	10.1111/acem.14767	Punches 2023	Excluded	Exclusion reason: Wrong Comparator: No comparison between care of patients identified as Black and patients identified as White.;

S4 Table. All studies identified in the literature search

1	Successful school-based intervention for inner-city children with persistent asthma	Anderson, M. E.; Freas, M. R.; Wallace, A. S.; Kempe, A.; Gelfand, E. W.; Liu, A. H.	2004	Journal of Asthma	41	4	445-453	10.1081/JAS-120033987	Anderson 2004	Irrelevant	Excluded by TI/AB review
2	Latino Children with Asthma: Rates and Risks for Medical Care Utilization	Berg, J.; Wahlgren, D. R.; Hofstetter, C. R.; Meltzer, S. B.; Meltzer, E. O.; Matt, G. E.; Martinez-	2004	Journal of Asthma	41	2	147-157	10.1081/JAS-120026072	Berg 2004	Irrelevant	Excluded by TI/AB review
3	Role of Culture, Race, and Ethnicity in Child and Adolescent Psychiatry	Bird, H√©ctor R.; Wiener, Jerry M.; Dulcan, Mina K.	2004	The American Psychiatric Publishing textbook of child and adolescent psychiatry., 3rd ed.	NA	NA	45-55	NA	Bird 2004	Irrelevant	Excluded by TI/AB review

S4 Table. All studies identified in the literature search

4	How healthy are we?: A national study of well-being at midlife	Brim, Orville Gilbert; Ryff, Carol D.; Kessler, Ronald C.	2004	The John D. and Catherine T. MacArthur foundation	NA	NA	NA	NA	Brim 2004	Irrelevant	Excluded by TI/AB review
5	Perception of health and quality of life in minorities after mild-to-moderate traumatic brain injury	Brown, S. A.; McCauley, S. R.; Levin, H. S.; Contant, C.; Boake, C.	2004	Applied Neuropsychology	11	1	54-64	10.1207/s15324826an1101_7	Brown 2004	Irrelevant	Excluded by TI/AB review
7	Implementation of Community Consultation for Waiver of Informed Consent in Emergency Research: One Institutional [illegible]	Dix, E. S.; Esposito, D.; Spinosa, F.; Olson, N.; Chapman, S.	2004	Journal of Investigative Medicine	52	2	113-116	10.1136/jim-52-02-20	Dix 2004	Irrelevant	Excluded by TI/AB review
8	Statistics for research, 3rd ed	Dowdy, Shirley; Wearden, Stanley; Chilko, Daniel	2004	Wiley series in probability and	NA	NA	NA	10.1002/0471477435	Dowdy 2004	Irrelevant	Excluded by TI/AB review
9	Assessment of the standardized reporting guidelines ECG classification system: The presenting ECG [illegible]	Forest, R. S.; Shofer, F. S.; Sease, K. L.; Hollander, J. E.	2004	Annals of Emergency Medicine	44	3	206-212	10.1016/j.annemergmed.2004.02.031	Forest 2004	Irrelevant	Excluded by TI/AB review

S4 Table. All studies identified in the literature search

10	Patient preference for route of pain medication delivery	Fosnocht, D. E.; Hollifield, M. B.; Swanson, E. R.	2004	Journal of Emergency Medicine	26	1	11-Jul	10.1016/j.jemermed.2003.03.002	Fosnocht 2004	Irrelevant	Excluded by TI/AB review
11	Providing optimal emergency care for medically underserved children	Friedlaender, E. Y.; Alessandrini, E. A.	2004	Clinical Pediatric Emergency Medicine	5	2	109-119	10.1016/j.cpem.2004.01.001	Friedlaender 2004	Irrelevant	Excluded by TI/AB review

S4 Table. All studies identified in the literature search

12	Emergency department use and perceived delay in accessing illness care among children with Medicaid	Galbraith, A. A.; Semura, J.; McAninch-Dake, B.; Anderson, N.; Christakis, D. A.	2004	Ambulatory Pediatrics	4	6	509-513	10.1367/A04-008R.1	Galbraith 2004	Irrelevant	Excluded by TI/AB review
13	A comparison of the influence of hospital-trained, ad hoc, and telephone interpreters on perceived satisfaction of limited English- [illegible]	Garcia, E. A.; Roy, L. C.; Okada, P. J.; Perkins, S. D.; Wiebe, R. A.	2004	Pediatric Emergency Care	20	6	373-378	10.1097/01.pec.0000133611.42699.08	Garcia 2004	Irrelevant	Excluded by TI/AB review

S4 Table. All studies identified in the literature search

15	Racial comparisons of health care and glycemic control for African American and white diabetic adults in an urban managed care organization	Gary, T. L.; McGuire, M.; McCauley, J.; Brancati, F. L.	2004	Disease Managemen t	7	1	25-34	10.1089/10 935070432 2918970	Gary 2004	Irrelevant	Excluded by TI/AB review
16	Insurance status and the treatment of myocardial infarction at academic centers [corrected] [published erratum appears in ACAD EMERG MED	Hiestand, B. C.; Prall, D. M.; Lindsell, C. J.; Hoekstra, J. W.; Pollack, C. V.; Hollander, J. E.; Tiffany, B. R.; Peacock, W. F.; Diercks, D. B.;	2004	Academic Emergency Medicine	11	4	343-348	NA	Hiestand 2004	Irrelevant	Excluded by TI/AB review
17	Insurance status and the treatment of myocardial infarction at	Hiestand, B. C.; Prall, D. M.; Lindsell, C. J.; Hoekstra, J. W.;	2004	Academic Emergency Medicine	11	4	343-348	10.1197/j.ae m.2003.12. 017	Hiestand 2004	Irrelevant	Excluded by TI/AB review

S4 Table. All studies identified in the literature search

18	Patient race/ethnicity and quality of patient-physician communication during medical visits	Johnson, R. L.; Roter, D.; Powe, N. R.; Cooper, L. A.	2004	American Journal of Public Health	94	12	2084-2090	10.2105/AJPH.94.12.2084	Johnson 2004	Irrelevant	Excluded by TI/AB review
19	An experience in creating a men's health electronic registry in a	Joy, S. V.; Gouveia-Pisano, J. A.; Diaz, S. M.	2004	Journal of Clinical Outcomes Managemen	11	4	223-228	NA	Joy 2004	Irrelevant	Excluded by TI/AB review
20	Culture and the psychiatric emergency service	Lettich, Louise; Tseng, Wen-Shing; Streltzer, Jon	2004	Cultural competence in clinical psychiatry.	NA	NA	53-65	NA	Lettich 2004	Irrelevant	Excluded by TI/AB review

S4 Table. All studies identified in the literature search

21	Using Telemedicine to Provide Pediatric Subspecialty Care to Children with Special Health Care Needs in an Underserved Rural [illegible]	Marcin, J. P.; Ellis, J.; Mawis, R.; Nagrampa, E.; Nesbitt, T. S.; Dimand, R. J.	2004	Pediatrics	113	1 I	6-Jan	10.1542/peds.113.1.1	Marcin 2004	Irrelevant	Excluded by TI/AB review
22	Are Low-Income Elderly Patients at Risk for Poor Diabetes Care?	McCall, D. T.; Sauaia, A.; Hamman, R. F.; Reusch, J. E.; Barton, P.	2004	Diabetes Care	27	5	1060-1065	10.2337/diacare.27.5.1060	McCall 2004	Irrelevant	Excluded by TI/AB review
23	Therapy of heart failure in African Americans: lessons from an urban public hospital	Mehta, S. K.; Toto, K. H.; Nelson, L. L.; Drazner, M. H.	2004	Congestive heart failure (Greenwich, Conn.)	10	1	40-43	10.1111/j.1527-5299.2004.02025.x	Mehta 2004	Irrelevant	Excluded by TI/AB review

S4 Table. All studies identified in the literature search

24	Disparity and priorities	Meryn, Siegfried	2004	Journal of Men's Health & Gender	1	3-Feb	133-135	10.1016/j.jmhg.2004.07.037	Meryn 2004	Irrelevant	Excluded by TI/AB review
25	Cancer, culture, and communication	Moore, Rhonda J.; Spiegel, David	2004	NA	NA	NA	NA	10.1007/b105731	Moore 2004	Irrelevant	Excluded by TI/AB review
26	Caring for refugees in emergency departments in New Zealand	Mortensen, A.; Young, N.	2004	Nursing Praxis in New Zealand	20	2	24-35	NA	Mortensen 2004	Irrelevant	Excluded by TI/AB review
27	Emergency room utilization in Copenhagen: a comparison of immigrant groups and Danish-born residents	Norredam, M.; Krasnik, A.; Moller Sorensen, T.; Keiding, N.; Joost Michaelsen, J.; Sonne Nielsen, A.	2004	Scand J Public Health	32	1	53-9	10.1080/14034940310001659	Norredam 2004	Irrelevant	Excluded by TI/AB review

S4 Table. All studies identified in the literature search

28	Building Coalitions for Tobacco Control and Prevention in the 21st Century	Northridge, Mary E.	2004	American Journal of Public Health	94	2	178-180	10.2105/AJPH.94.2.178	Northridge 2004	Irrelevant	Excluded by TI/AB review
29	The effect of limited English Proficiency on admission rates	Rogers, A. J.; Delgado, C. A.; Simon, H. K.	2004	American Journal of Emergency Medicine	22	7	534-536	10.1016/j.ajem.2004.08.012	Rogers 2004	Irrelevant	Excluded by TI/AB review
30	Pneumococcal vaccination in the emergency department: An assessment of need	Rudis, M. I.; Stone, S. C.; Goad, J. A.; Lee, V. W.; Chitchyan, A.; Newton, K. I.	2004	Annals of Emergency Medicine	44	4	386-392	10.1016/j.annemergmed.2004.05.007	Rudis 2004	Irrelevant	Excluded by TI/AB review
31	Inadequate Analgesia in Emergency Medicine	Rupp, T.; Delaney, K. A.	2004	Annals of Emergency Medicine	43	4	494-503	10.1016/j.annemergmed.2003.11.019	Rupp 2004	Irrelevant	Excluded by TI/AB review

S4 Table. All studies identified in the literature search

33	Are there racial differences in the way patients with psychotic disorders are treated at their first hospitalization?	Sohler, Nancy L.; Bromet, Evelyn J.; Lavelle, Janet; Craig, Thomas J.; Mojtabai, Ramin	2004	Psychological Medicine	34	4	705-718	10.1017/S0033291703001363	Sohler 2004	Irrelevant	Excluded by TI/AB review
34	A patient education intervention does not improve satisfaction with	Sun, B. C.; Brinkley, M.; Morrissey, J.; Rice, P.; Stair, T.	2004	Annals of Emergency Medicine	44	4	378-383	10.1016/j.annemergmed.2004.02.002	Sun 2004	Irrelevant	Excluded by TI/AB review
35	Factors affecting pain scores during female urethral catheterization	Tanabe, P.; Steinmann, R.; Anderson, J.; Johnson, D.; Metcalf, S.; Ring-Hurn, E.	2004	Academic Emergency Medicine	11	6	699-702	NA	Tanabe 2004	Irrelevant	Excluded by TI/AB review
36	Why culture and language matter: The clinical consequences of providing [illegible]	Taveras, E. M.; Flores, G.	2004	Clinical Pediatric Emergency Medicine	5	2	76-84	10.1016/j.cpem.2004.01.004	Taveras 2004	Irrelevant	Excluded by TI/AB review

S4 Table. All studies identified in the literature search

37	Patient satisfaction in emergency medicine	Taylor, C.; Benger, J. R.	2004	Emergency Medicine Journal	21	5	528-532	10.1136/emj.2002.003723	Taylor 2004	Irrelevant	Excluded by TI/AB review
38	Family reports of care denials for children enrolled in TennCare	Valet, R. S.; Kutny, D. F.; Hickson, G. B.; Cooper, W. O.	2004	Pediatrics	114	1	e37-42	NA	Valet 2004	Irrelevant	Excluded by TI/AB review
39	Annual review of nursing research, Vol 22, 2004: Eliminating health disparities among racial and ethnic minorities in the [illegible]	Villarruel, Antonia M.; Porter, Cornelia P.	2004	Annual review of nursing research; Vol 24; ISSN: 0739-6686 (Print)	24	NA	NA	NA	Villarruel 2004	Irrelevant	Excluded by TI/AB review

S4 Table. All studies identified in the literature search

40	Patterns of hospital-based pediatric care across diverse ethnicities: the case of pneumonia	Washington, E. L.; Shen, J. J.; Bell, R.; Coleman, C.; Shi, L.	2004	J Health Care Poor Underserved	15	3	462-73	10.1353/hpu.2004.0050	Washington 2004	Irrelevant	Excluded by TI/AB review
41	Hispanic healthcare disparities: challenging the myth of a monolithic Hispanic [illegible]	Weinick, R. M.; Jacobs, E. A.; Stone, L. C.; Ortega, A. N.; Burstin, H.	2004	Med Care	42	4	313-20	10.1097/01.mlr.0000118705.27241.7c	Weinick 2004	Irrelevant	Excluded by TI/AB review
45	Cultural competence in the emergency department	Wolf, K. E.; Calmes, D.	2004	Topics in Emergency Medicine	26	1	13-Sep	NA	Wolf 2004	Irrelevant	Excluded by TI/AB review

S4 Table. All studies identified in the literature search

46	Correlates of Health Insurance among Persons with Schizophrenia in a Statewide Behavioral Health Care System	Yanos, P. T.; Lu, W.; Minsky, S.; Kiely, G. L.	2004	Psychiatric Services	55	1	79-82	10.1176/appi.ps.55.1.79	Yanos 2004	Irrelevant	Excluded by TI/AB review
47	Acute stroke care in the US: Results from 4 pilot prototypes of the Paul Coverdell National Acute Stroke Registry	Arora, S.; Broderick, J. P.; Frankel, M.; Heinrich, J. P.; Hickenbottom, S.; Karp, H.; LaBresh, K. A.; Malarcher, A.; Mensah, G.; Moomaw, C. J.; Reeves, M. J.; [illegible]	2005	Stroke	36	6	1232-1240	10.1161/01.STR.0000165902.18021.5b	Arora 2005	Irrelevant	Excluded by TI/AB review
48	Cultural competence and health care disparities: key	Betancourt, J. R.; Green, A. R.; Carrillo, J. E.; Park, E. R.	2005	Health Affairs	24	2	499-505	10.1377/hlthaff.24.2.499	Betancourt 2005	Irrelevant	Excluded by TI/AB review
51	Characteristics of families that complain following pediatric emergency visits	Carroll, K. N.; Cooper, W. O.; Blackford, J. U.; Hickson, G. B.	2005	Ambulatory Pediatrics	5	6	326-331	10.1367/A04-187R1.1	Carroll 2005	Irrelevant	Excluded by TI/AB review

S4 Table. All studies identified in the literature search

53	Use of asthma guidelines by primary care providers to reduce hospitalizations and emergency department visits in poor, minority, urban children	Cloutier, M. M.; Hall, C. B.; Wakefield, D. B.; Bailit, H.	2005	Journal of Pediatrics	146	5	591-597	10.1016/j.jpeds.2004.12.017	Cloutier 2005	Irrelevant	Excluded by TI/AB review
54	Emancipatory Discourses and the Ethics and Politics of Interpretation	Denzin, Norman K.; Denzin, Norman K.; Lincoln, Yvonna S.	2005	The Sage handbook of qualitative research., 3rd ed.	NA	NA	933-958	NA	Denzin 2005	Irrelevant	Excluded by TI/AB review
55	Children's Health Care in the First National Healthcare Quality Report and National Healthcare Disparities Report	Dougherty, D.; Meikle, S. F.; Owens, P.; Kelley, E.; Moy, E.; Dougherty, Denise; Meikle, Susan F.; Owens, Pamela; Kelley, Ed; Moy, [illegible]	2005	Medical Care	43	3	I-58	10.1097/00005650-200503001-00009	Dougherty 2005	Irrelevant	Excluded by TI/AB review

S4 Table. All studies identified in the literature search

56	Health-related quality of life in overweight and nonoverweight black and white adolescents	Fallon, E. M.; Tanofsky-Kraff, M.; Norman, A. C.; McDuffie, J. R.; Taylor, E. D.; Cohen, M. L.; Young-Hyman, D.; Keil, M.; Kolotkin, R. L.; Yanovski, J. A.; Fallon, Erica M.; Tanofsky-Kraff, Marian; Norman, Anne-Caroline; McDuffie, Jennifer R.; Taylor, Erica D.; Cohen, Marc L.;	2005	Journal of Pediatrics	147	4	443-450	10.1016/j.jpeds.2005.05.039	Fallon 2005	Irrelevant	Excluded by TI/AB review
57	The psychology of rights and duties: Empirical contributions and normative commentaries	Finkel, Norman J.; Moghaddam, Fathali M.	2005	The law and public policy	NA	NA	NA	10.1037/10872-000	Finkel 2005	Irrelevant	Excluded by TI/AB review
58	Changing attitudes about pain and pain control in emergency medicine	Fosnocht, D. E.; Swanson, E. R.; Barton, E. D.	2005	Emergency Medicine Clinics of North America	23	2	297-306	10.1016/j.emc.2004.12.003	Fosnocht 2005	Irrelevant	Excluded by TI/AB review

S4 Table. All studies identified in the literature search

60	Acute musculoskeletal pain in the emergency department: a review of the [illegible]	Garbez, R.; Puntillo, K.	2005	AACN clinical issues	16	3	310-319	10.1097/00044067-200507000-00005	Garbez 2005	Irrelevant	Excluded by TI/AB review
61	Improving the quality of life of erectile dysfunction (ED) patients through penile implants	Gheiler, J.; Sharpe, I.	2005	Ethnicity and Disease	15	3 SUPPL. 4	S4-41-S4-42	NA	Gheiler 2005	Irrelevant	Excluded by TI/AB review
62	Impact of providing a medical home to the uninsured: Evaluation of a statewide program	Gill, J. M.; Fagan, H. B.; Townsend, B.; Mainous, A. G.	2005	Journal of Health Care for the Poor and Underserved	16	3	515-535	10.1353/hpu.2005.0050	Gill 2005	Irrelevant	Excluded by TI/AB review
63	Pediatric traumatic brain injury: Do racial/ethnic disparities exist in brain injury severity, mortality, or medical [illegible]	Howard, I.; Joseph, J. G.; Natale, J. E.	2005	Ethnicity & Disease	15	4	S51-S56	NA	Howard 2005	Irrelevant	Excluded by TI/AB review

S4 Table. All studies identified in the literature search

64	Association of race/ethnicity with emergency department wait times	James, C. A.; Bourgeois, F. T.; Shannon, M. W.	2005	Pediatrics	115	3	e310-5	10.1542/peds.2004-1541	James 2005	Irrelevant	Excluded by TI/AB review
68	Patient and partner satisfaction with the Mentor Alpha-1 inflatable penile prosthesis	Jensen, J. B.; Madsen, S. S.; Larsen, E. H.; Jensen, K. M. E.; Kirkeby, H. J.	2005	Scandinavian Journal of Urology and Nephrology	39	1	66-68	10.1080/00365590410018756	Jensen 2005	Irrelevant	Excluded by TI/AB review
69	Quality of life and facial trauma: Psychological and body image effects	Levine, E.; Degutis, L.; Pruzinsky, T.; Shin, J.; Persing, J. A.	2005	Annals of Plastic Surgery	54	5	502-510	10.1097/01.sap.0000155282.48465.94	Levine 2005	Irrelevant	Excluded by TI/AB review
70	Antivirals and antibiotics for influenza in the United States, 1995-2002	Linder, J. A.; Bates, D. W.; Platt, R.	2005	Pharmacoepidemiology and Drug Safety	14	8	531-536	10.1002/pds.1067	Linder 2005	Irrelevant	Excluded by TI/AB review
71	Can education policy be health policy? Implications of research on the [illegible]	Low, M. D.; Low, B. J.; Baumler, E. R.; Huynh, P. T.	2005	Journal of Health Politics, Policy & Law	30	6	1131-1162	NA	Low 2005	Irrelevant	Excluded by TI/AB review

S4 Table. All studies identified in the literature search

72	Emergency department HIV testing and counseling: An ongoing experience in a low-prevalence area	Lyons, M. S.; Lindsell, C. J.; Ledyard, H. K.; Frame, P. T.; Trott, A. T.	2005	Annals of Emergency Medicine	46	1	22-28	10.1016/j.annemergmed.2004.12.022	Lyons 2005	Irrelevant	Excluded by TI/AB review
73	Creating new inequalities: Contradictions of reform	McNeil, Linda M.; Shapiro, H. Svi; Purpel, David E.	2005	Critical social issues in American education: Democracy and meaning in a [illegible]	NA	NA	77-88	NA	McNeil 2005	Irrelevant	Excluded by TI/AB review
74	Performance of a population-based cardiac risk stratification tool in Asian patients with chest pain [corrected] [illegible]	Miller, C. D.; Lindsell, C. J.; Anantharaman, V.; Lim, S.; Greenway, J.; Pollack, C. V.; Tiffany, B. R.; Hollander, J. E.; [illegible]	2005	Academic Emergency Medicine	12	5	423-430	NA	Miller 2005	Irrelevant	Excluded by TI/AB review

S4 Table. All studies identified in the literature search

75	Psychotherapy with women: Exploring diverse contexts and identities	Mirkin, Marsha Pravder; Suyemoto, Karen L.; Okun, Barbara F.	2005	NA	NA	NA	NA	NA	Mirkin 2005	Irrelevant	Excluded by TI/AB review
76	The epidemiology of bipolar disorder: Sociodemographic, disability and service utilization data from the [illegible]	Morgan, V. A.; Mitchell, P. B.; Jablensky, A. V.	2005	Bipolar Disorders	7	4	326-337	10.1111/j.1399-5618.2005.00229.x	Morgan 2005	Irrelevant	Excluded by TI/AB review
77	Clinical decision-making in psychiatric emergency services (PES)	Muroff, Jordana R.	2005	NA	65	NA	3993-3993	NA	Muroff 2005	Irrelevant	Excluded by TI/AB review
78	Analyzing Talk and Text	Per√§kyl√§, Anssi; Denzin, Norman K.; Lincoln, Yvonna S.	2005	The Sage handbook of qualitative research., 3rd ed.	NA	NA	869-886	NA	Per√§kyl√§ 2005	Irrelevant	Excluded by TI/AB review

S4 Table. All studies identified in the literature search

79	'Multiple margins' (being older, a woman, or a visible minority) constrained older women's access to Canadian health care	Pirner, D.	2005	Evidence Based Nursing	8	4	128-128	NA	Pirner 2005	Irrelevant	Excluded by TI/AB review
80	Orofacial pain: patient satisfaction and delay of urgent care	Riley, J. L., 3rd; Gilbert, G. H.; Heft, M. W.	2005	Public Health Rep	120	2	140-9	10.1177/003335490512000207	Riley 2005	Irrelevant	Excluded by TI/AB review
82	Demographic predictors of chronic disease management	Robinson, S. A.	2005	NA	NA	Ph.D.	106 p-106 p	NA	Robinson 2005	Irrelevant	Excluded by TI/AB review
83	Researchers' understanding of the federal guidelines for waiver of and exception from informed consent	Sanders, A. B.; Hiller, K.; Duldner, J.	2005	Academic Emergency Medicine	12	11	1045-1049	10.1197/j.aem.2005.06.013	Sanders 2005	Irrelevant	Excluded by TI/AB review
84	Reducing emergency department visits and	Self, T. H.; Chrisman, C. R.; Mason, D. L.; Rumbak, M. J.	2005	Journal of Asthma	42	10	807-812	10.1080/02770900500369835	Self 2005	Irrelevant	Excluded by TI/AB review

S4 Table. All studies identified in the literature search

85	Racial variations in treatment and outcomes of black and white patients with high-risk non-ST-elevation acute [illegible]	Sonel, A. F.; Good, C. B.; Mulgund, J.; Roe, M. T.; Gibler, W. B.; Smith, S. C., Jr.; Cohen, M. G.; Pollack, C. V., Jr.; [illegible]	2005	Circulation	111	10	1225-1232	NA	Sonel 2005	Irrelevant	Excluded by TI/AB review
86	Why can't we get it right?: Designing high-quality professional development for standards-based [illegible]	Speck, Marsha; Knipe, Caroll	2005	NA	NA	NA	NA	NA	Speck 2005	Irrelevant	Excluded by TI/AB review
87	Practice guidelines for the diagnosis and management of skin and soft-	Stevens, D. L.; Bisno, A. L.; Chambers, H. F.; Everett, E. D.;	2005	Clinical Infectious Diseases	41	10	1373-1406	10.1086/497143	Stevens 2005	Irrelevant	Excluded by TI/AB review

S4 Table. All studies identified in the literature search

88	Peer review and feedback can modify pain treatment patterns for Emergency Department patients with fractures	Sucov, A.; Nathanson, A.; McCormick, J.; Proano, L.; Reinert, S. E.; Jay, G.	2005	American Journal of Medical Quality	20	3	138-143	10.1177/10628606042 74384	Sucov 2005	Irrelevant	Excluded by TI/AB review
89	From the Editor--Harrowing Deaths in Prison	Thomas, Sandra P.	2005	Issues in Mental Health Nursing	26	6	559-560	10.1080/01612840590 959308	Thomas 2005	Irrelevant	Excluded by TI/AB review
90	Resident physicians' preparedness to provide cross-cultural care	Weissman, J. S.; Betancourt, J.; Campbell, E. G.; Park, E. R.; Kim, M.; Clarridge, B.; [illegible]	2005	JAMA: Journal of the American Medical [illegible]	294	9	1058-1067	10.1001/jam a.294.9.105 8	Weissman 2005	Irrelevant	Excluded by TI/AB review

S4 Table. All studies identified in the literature search

91	Study: pain management is inconsistent in EDs	NA	2006	ED Nursing	9	12	139-141	NA	NA	Irrelevant	Excluded by TI/AB review
92	Diabetes self-management education program for Medicaid recipients: A continuous quality improvement process	Balamurugan, A.; Ohsfeldt, R.; Hughes, T.; Phillips, M.	2006	Diabetes Educator	32	6	893-900	10.1177/0145721706294787	Balamurugan 2006	Irrelevant	Excluded by TI/AB review
93	What providers from general emergency departments say about implementing a pediatric asthma pathway	Butterfoss, F. D.; Major, D. A.; Clarke, S. M.; Cardenas, R. A.; Isaacman, D. J.; Mason, J. D.; Clements, D. L.	2006	Clinical Pediatrics	45	4	325-333	10.1177/000992280604500404	Butterfoss 2006	Irrelevant	Excluded by TI/AB review

S4 Table. All studies identified in the literature search

94	Consumer and family experiences in the emergency department	Cerel, J.; Currier, G. W.; Conwell, Y.	2006	Journal of Psychiatric Practice	12	6	341-347	10.1097/00131746-200611000-00002	Cerel 2006	Irrelevant	Excluded by TI/AB review
95	Health Care for Children and Youth in the United States: Annual Report on Patterns of Coverage, Utilization, Quality, and Expenditures by a County Level of Urban Influence	Chevarley, F. M.; Owens, P. L.; Zodet, M. W.; Simpson, L. A.; McCormick, M. C.; Dougherty, D.	2006	Ambulatory Pediatrics	6	5	241-264	10.1016/j.ambp.2006.06.004	Chevarley 2006	Irrelevant	Excluded by TI/AB review
96	Providing health care services to the formerly homeless: A quasi-experimental evaluation	Ciaranello, A. L.; Molitor, F.; Leamon, M.; Kuenneth, C.; Tancredi, D.; Diamant, A. L.; Kravitz, R. L.	2006	Journal of Health Care for the Poor and Underserved	17	2	441-461	10.1353/hpu.2006.0056	Ciaranello 2006	Irrelevant	Excluded by TI/AB review

S4 Table. All studies identified in the literature search

97	Pain and ethnicity in the United States: A systematic review	Cintron, A.; Morrison, R. S.	2006	Journal of Palliative Medicine	9	6	1454-1473	10.1089/jpm.2006.9.1454	Cintron 2006	Irrelevant	Excluded by TI/AB review
98	Asthma guideline use by pediatricians in private practices and asthma morbidity	Cloutier, M. M.; Wakefield, D. B.; Sangeloty-Higgins, P.; Delaronde, S.; Hall, C. B.	2006	Pediatrics	118	5	1880-1887	10.1542/peds.2006-1019	Cloutier 2006	Irrelevant	Excluded by TI/AB review
99	Addressing racism: Facilitating cultural competence in mental health and educational settings	Constantine, Madonna G.; Sue, Derald Wing	2006	NA	NA	NA	NA	NA	Constantine 2006	Irrelevant	Excluded by TI/AB review

S4 Table. All studies identified in the literature search

100	Disparities in complementary and alternative medical therapy recommendations for children in two different [illegible]	DeMattia, A.; Moskowitz, H.; Kemper, K. J.; Laraque, D.	2006	Ambulatory Pediatrics	6	6	312-317	10.1016/j.ambp.2006.08.004	DeMattia 2006	Irrelevant	Excluded by TI/AB review
101	After hours healthcare for older patients in New Zealand	Eastwood, A.; Dowell, A.	2006	New Zealand Medical Journal	119	1234	NA	NA	Eastwood 2006	Irrelevant	Excluded by TI/AB review
102	Chest Pain: Physician Perceptions and Decisionmaking in a London Emergency Department	Farmer, S. A.; Higginson, I. J.	2006	Annals of Emergency Medicine	48	1	77-85	10.1016/j.annemergmed.2005.12.002	Farmer 2006	Irrelevant	Excluded by TI/AB review

S4 Table. All studies identified in the literature search

105	Chest pain: Communication of symptoms and history in a	Farmer, S. A.; Roter, D. L.; Higginson, I. J.	2006	Patient Education and Counseling	63	2-Jan	138-144	10.1016/j.pec.2005.09.009	Farmer 2006	Irrelevant	Excluded by TI/AB review
107	Racial differences in medication switching and	Garver, D.; Lazarus, A.; Rajagopalan, K.; Lamerato, L.; Katz,	2006	Psychiatric Services	57	5	666-672	10.1176/ps.2006.57.5.666	Garver 2006	Irrelevant	Excluded by TI/AB review
109	Language and length of stay in the pediatric emergency department	Goldman, R. D.; Amin, P.; Macpherson, A.	2006	Pediatric Emergency Care	22	9	640-643	10.1097/01.pec.0000227865.38815.ec	Goldman 2006	Irrelevant	Excluded by TI/AB review
110	Perceptions of differential treatment from the viewpoints of attending physicians, residents, and [illegible]	Gonz√°lez-Espada, W. J.; Ibarra, M.; Ochoa, E. R.; Vargas, P. A.	2006	Hispanic Health Care International (Springer Publishing Company, Inc.)	4	3	159-169	10.1891/hhci-v4i3a005	Gonz√°lez-Espada 2006	Irrelevant	Excluded by TI/AB review
111	Stuck in neutral: continued challenges with healthcare disparities	Haley, L. L., Jr.	2006	Academic Emergency Medicine	13	2	191-194	NA	Haley 2006	Irrelevant	Excluded by TI/AB review

S4 Table. All studies identified in the literature search

116	Racial and ethnic disparities in the emergency department: a public health perspective	Heron, S. L.; Stettner, E.; Haley, L. L., Jr.	2006	Emerg Med Clin North Am	24	4	905-23	10.1016/j.emc.2006.06.009	Heron 2006	Irrelevant	Excluded by TI/AB review
118	Medical error identification, disclosure, and reporting: do emergency [illegible]	Hobgood, C.; Weiner, B.; Tamayo-Sarver, J. H.	2006	Academic Emergency Medicine	13	4	443-451	NA	Hobgood 2006	Irrelevant	Excluded by TI/AB review
119	A prospective study of the consulting behaviour of older people with knee pain	Jordan, K.; Jinks, C.; Croft, P.	2006	British Journal of General Practice	56	525	269-276	NA	Jordan 2006	Irrelevant	Excluded by TI/AB review
120	The Health Commons and care of New Mexico's uninsured	Kaufman, A.; Derksen, D.; Alfero, C.; DeFelice, R.; Sava, S.; Tomedi, A.; Baptiste, N.; Jaeger, L.; Powell, W.	2006	Annals of Family Medicine	4	SUPPL. 1	S22-S27	10.1370/afm.539	Kaufman 2006	Irrelevant	Excluded by TI/AB review

S4 Table. All studies identified in the literature search

121	Partners in School Asthma Management: Evaluation of a self-management program for children with [illegible]	Kay Bartholomew, L.; Sockrider, M. M.; Abramson, S. L.; Swank, P. R.; Czyzewski, D. I.; Tortolero, S. R.; Markham, C. M.; [illegible]	2006	Journal of School Health	76	6	283-290	10.1111/j.1746-1561.2006.00113.x	KayBartholomew 2006	Irrelevant	Excluded by TI/AB review
122	Community socioeconomic status and prehospital times in acute stroke and transient ischemic attack: Do poorer patients have [illegible]	Kleindorfer, D. O.; Lindsell, C. J.; Broderick, J. P.; Flaherty, M. L.; Woo, D.; Ewing, I.; Schmit, P.; Moomaw, C.; Alwell, K.; Pancioli, [illegible]	2006	Stroke	37	6	1508-1513	10.1161/01.STR.0000222933.94460.dd	Kleindorfer 2006	Irrelevant	Excluded by TI/AB review
123	The efficacy of asthma case management in an urban school district in reducing [illegible]	Levy, M.; Heffner, B.; Stewart, T.; Beeman, G.	2006	Journal of School Health	76	6	320-324	10.1111/j.1746-1561.2006.00120.x	Levy 2006	Irrelevant	Excluded by TI/AB review
124	Pain management in the emergency department	Lim, G. H.; Wee, F. C.; Seow, E.	2006	Hong Kong Journal of Emergency Medicine	13	1	38-45	10.1177/102490790601300105	Lim 2006	Irrelevant	Excluded by TI/AB review

S4 Table. All studies identified in the literature search

125	The Impact of Social Inequalities on Health Care	Lim, Russell F.; Lu, Francis G.; Hilty, Donald M.; Wedding, Danny; Stuber, Margaret L.	2006	Behavior & medicine., 4th ed.	NA	NA	287-299	NA	Lim 2006	Irrelevant	Excluded by TI/AB review
126	A randomized comparison of nitrous oxide plus hematoma block versus ketamine plus midazolam [illegible]	Luhmann, J. D.; Schootman, M.; Luhmann, S. J.; Kennedy, R. M.	2006	Pediatrics	118	4	e1078-e1086	10.1542/peds.2005-1694	Luhmann 2006	Irrelevant	Excluded by TI/AB review
127	Reducing frequent flyer emergency department visits	Michelen, W.; Martinez, J.; Lee, A.; Wheeler, D. P.	2006	Journal of Health Care for the Poor and Underserved	17	1 SUPPL.	59-69	10.1353/hpu.2006.0010	Michelen 2006	Irrelevant	Excluded by TI/AB review
128	Differences in triage thresholds for patients presenting with possible acute coronary syndromes: More than meets the eye	Milch, C. E.; Kent, D. M.; Ruthazer, R.; Pope, J. H.; Aufderheide, T. P.; McNutt, R. A.; Selker, H. P.	2006	Journal of Investigative Medicine	54	2	76-85	10.2310/6650.2006.05036	Milch 2006	Irrelevant	Excluded by TI/AB review

S4 Table. All studies identified in the literature search

131	Accelerating time to reperfusion in acute myocardial infarction: Prehospital and emergency [illegible]	Ornato, J. P.	2006	Reviews in Cardiovascular Medicine	7	SUPPL. 4	S49-S60	NA	Ornato 2006	Irrelevant	Excluded by TI/AB review
132	Preventing prejudice: A guide for counselors, educators, and parents, 2nd ed	Ponterotto, Joseph G.; Utsey, Shawn O.; Pedersen, Paul B.	2006	NA	NA	NA	NA	NA	Ponterotto 2006	Irrelevant	Excluded by TI/AB review
133	Impact of patient-centered decision support on quality of asthma care in	Porter, S. C.; Forbes, P.; Feldman, H. A.; Goldmann, D. A.	2006	Pediatrics	117	1	e33-e42	10.1542/peds.2005-0906	Porter 2006	Irrelevant	Excluded by TI/AB review
135	Patient perceptions of emergency physicians: The gender gap still exists	Prince, L. A.; Pipas, L.; Brown, L. H.	2006	Journal of Emergency Medicine	31	4	361-364	10.1016/j.jemermed.2006.04.011	Prince 2006	Irrelevant	Excluded by TI/AB review
136	Asthma care and management before an emergency department visit in [illegible]	Reeves, M. J.; Bohm, S. R.; Korzeniewski, S. J.; Brown, M. D.	2006	Pediatrics	117	4	S118-S126	10.1542/peds.2005-2000I	Reeves 2006	Irrelevant	Excluded by TI/AB review
137	The Paul Coverdell National Acute Stroke Registry. Initial Results from Four	Reeves, M. J.; Broderick, J. P.; Frankel, M.; LaBresh, K. A.; Schwamm, L.;	2006	American Journal of Preventive Medicine	31	6 SUPPL. 2	S202-S209	10.1016/j.amepre.2006.08.007	Reeves 2006	Irrelevant	Excluded by TI/AB review

S4 Table. All studies identified in the literature search

138	Cognition and Caregiving	Rowland, Christopher V., Jr.	2006	American Journal of Alzheimer's Disease and Other	21	4	225-225	10.1177/15333175062 90746	Rowland 2006	Irrelevant	Excluded by TI/AB review
139	Treating the World without Leaving Your ED:	Sheridan, I.	2006	Academic Emergency Medicine	13	8	896-903	10.1197/j.aem.2006.03.563	Sheridan 2006	Irrelevant	Excluded by TI/AB review
142	Contemporary youth culture: An international encyclopedia, Vol 1	Steinberg, Shirley; Parmar, Priya; Richard, Birgit	2006	NA	NA	NA	NA	NA	Steinberg 2006	Irrelevant	Excluded by TI/AB review
143	Indoor environmental exposures among children with asthma seen in an urban emergency department	Teach, S. J.; Crain, E. F.; Quint, D. M.; Hylan, M. L.; Joseph, J. G.	2006	Pediatrics	117	4	S152-S158	10.1542/peds.2005-2000M	Teach 2006	Irrelevant	Excluded by TI/AB review
144	Lay experiences and concerns with asthma in an urban hispanic community	Tumiel-Berhalter, L.; Zayas, L. E.	2006	Journal of the National Medical Association	98	6	875-880	NA	Tumiel-Berhalter 2006	Irrelevant	Excluded by TI/AB review

S4 Table. All studies identified in the literature search

146	Friendships Among Black, Latino, and Asian American Adolescents in an Urban Context	Way, Niobe; Becker, Bronwyn E.; Greene, Melissa L.; Balter, Lawrence; Tamis-LeMonda, Catherine S.	2006	Child psychology: A handbook of contemporary issues., 2nd ed.	NA	NA	415-443	NA	Way 2006	Irrelevant	Excluded by TI/AB review
147	Support for emergency department screening for intimate partner violence depends on perceived risk	Witting, M. D.; Furuno, J. P.; Hirshon, J. M.; Krugman, S. D.; P√©riss√©, A. R. S.; Limcangco, R.	2006	Journal of Interpersonal Violence	21	5	585-596	10.1177/0886260506286841	Witting 2006	Irrelevant	Excluded by TI/AB review
148	Prevalence and utility of DSM-IV eating disorder diagnostic criteria among youth	Ackard, D. M.; Fulkerson, J. A.; Neumark-Sztainer, D.	2007	International Journal of Eating Disorders	40	5	409-417	10.1002/eat.20389	Ackard 2007	Irrelevant	Excluded by TI/AB review

S4 Table. All studies identified in the literature search

149	Life-span adjustment of children to their parents' divorce	Amato, Paul R.; Ferguson, Susan J.	2007	Shifting the center: Understanding contemporary families., 3rd ed.	NA	NA	567-588	NA	Amato 2007	Irrelevant	Excluded by TI/AB review
150	Language ability: A neglected dimension in the profi ling of populations and health service users	Aspinall, P. J.	2007	Health Education Journal	66	1	90-106	10.1177/0017896907073797	Aspinall 2007	Irrelevant	Excluded by TI/AB review
151	Attitudes toward health care providers, collecting information about patients' race, [illegible]	Baker, D. W.; Hasnain-Wynia, R.; Kandula, N. R.; Thompson, J. A.; Brown, E. R.	2007	Medical Care	45	11	1034-1042	10.1097/mlr.0b013e318127148f	Baker 2007	Irrelevant	Excluded by TI/AB review

S4 Table. All studies identified in the literature search

152	Hospitalized patients with asthma who leave against medical advice: Characteristics, reasons, and outcomes	Baptist, A. P.; Warrier, I.; Arora, R.; Ager, J.; Massanari, R. M.	2007	Journal of Allergy and Clinical Immunology	119	4	924-929	10.1016/j.jaci.2006.11.695	Baptist 2007	Irrelevant	Excluded by TI/AB review
153	Quality of primary care and subsequent pediatric emergency department utilization	Brousseau, D. C.; Hoffmann, R. G.; Nattinger, A. B.; Flores, G.; Zhang, Y.; Gorelick, M.	2007	Pediatrics	119	6	1131-1138	10.1542/peds.2006-3518	Brousseau 2007	Irrelevant	Excluded by TI/AB review
154	Racial and ethnic disparities in the quality of asthma care	Cabana, M. D.; Lara, M.; Shannon, J.	2007	Chest	132	5	810S-817S	10.1378/chest.07-1910	Cabana 2007	Irrelevant	Excluded by TI/AB review

S4 Table. All studies identified in the literature search

155	Gender Bias in Cardiovascular Testing Persists after Adjustment for Presenting Characteristics and Cardiac Risk	Chang, A. M.; Mumma, B.; Sease, K. L.; Robey, J. L.; Shofer, F. S.; Hollander, J. E.	2007	Academic Emergency Medicine	14	7	599-605	10.1197/j.aem.2007.03.1355	Chang 2007	Irrelevant	Excluded by TI/AB review
157	Acute severe asthma: Triage, treatment and thereafter	Chua, F.; Lai, D.	2007	Current Anaesthesia and Critical Care	18	2	61-68	10.1016/j.cacc.2007.03.008	Chua 2007	Irrelevant	Excluded by TI/AB review
158	Pornography and black women's bodies	Collins, Patricia Hill; O'Toole, Laura L.; Schiffman, Jessica R.; Edwards, Marge L. Kiter	2007	Gender violence: Interdisciplinary perspectives., 2nd ed.	NA	NA	389-394	NA	Collins 2007	Irrelevant	Excluded by TI/AB review
159	Equitable access to dental care for an at-risk group: a review of services for Australian [illegible]	Davidson, N.; Skull, S.; Calache, H.; Chesters, D.; Chalmers, J.	2007	Aust N Z J Public Health	31	1	73-80	NA	Davidson 2007	Irrelevant	Excluded by TI/AB review

S4 Table. All studies identified in the literature search

160	Pharmacogenomics of Analgesic Agents	Drendel, A.	2007	Clinical Pediatric Emergency Medicine	8	4	262-267	10.1016/j.cpem.2007.08.001	Drendel 2007	Irrelevant	Excluded by TI/AB review
161	Factors associated with preference for sildenafil citrate and tadalafil for	Eardley, I.; Montorsi, F.; Jackson, G.; Mirone, V.; Chan, M. L. S.; Loughney,	2007	BJU International	100	1	122-129	10.1111/j.1464-410X.2007.06916.x	Eardley 2007	Irrelevant	Excluded by TI/AB review
162	Effect of race on asthma management and outcomes in a large, integrated managed care organization	Erickson, S. E.; Iribarren, C.; Tolstykh, I. V.; Blanc, P. D.; Eisner, M. D.	2007	Archives of Internal Medicine	167	17	1846-1852	10.1001/archinte.167.17.1846	Erickson 2007	Irrelevant	Excluded by TI/AB review

S4 Table. All studies identified in the literature search

163	Ethnicity and health	Field, David; Blakemore, K.; Taylor, Steve	2007	Sociology of health and health care., 4th ed.	NA	NA	69-92	NA	Field 2007	Irrelevant	Excluded by TI/AB review
164	Predictors of poor self-rated health at area level in the Eastern Regional Health Authority: a multilevel [illegible]	Fitzsimon, N.; Johnson, H.; Hayes, C.; Mulholland, D.; Kelleher, C. C.	2007	Irish Medical Journal	100	8	suppl 47-49	NA	Fitzsimon 2007	Irrelevant	Excluded by TI/AB review
165	Improving asthma-related health outcomes among low-income, multiethnic, school-aged children: [illegible]	Fox, P.; Porter, P. G.; Lob, S. H.; Boer, J. H.; Rocha, D. A.; Adelson, J. W.	2007	Pediatrics	120	4	e902-e911	10.1542/peds.2006-1805	Fox 2007	Irrelevant	Excluded by TI/AB review
166	Adolescents attending an adult emergency department: Their utilisation characteristics and [illegible]	Fry, R.; Kelleher, C.; Ryan, J.; Salter, N.; Murrin, C.	2007	Irish Medical Journal	100	7	NA	NA	Fry 2007	Irrelevant	Excluded by TI/AB review

S4 Table. All studies identified in the literature search

167	Factors associated with longer ED lengths of stay	Gardner, R. L.; Sarkar, U.; Maselli, J. H.; Gonzales, R.	2007	American Journal of Emergency Medicine	25	6	643-650	10.1016/j.ajem.2006.11.037	Gardner 2007	Irrelevant	Excluded by TI/AB review
168	Tadalafil is efficacious in Black American and Hispanic men with erectile dysfunction: Results from [illegible]	Gomery, P.; Bullock, A.; McGettigan, J.; Munarriz, R.; Natanegara, F.; Hague, J.; Kuepfer, C.; Abuja, S.; Wong, D.	2007	International Journal of Impotence Research	19	1	76-83	10.1038/sj.ijir.3901484	Gomery 2007	Irrelevant	Excluded by TI/AB review
169	Implicit bias among physicians and its prediction of thrombolysis decisions for black and white patients	Green, A. R.; Carney, D. R.; Pallin, D. J.; Ngo, L. H.; Raymond, K. L.; Iezzoni, L. I.; Banaji, M. R.	2007	Journal of General Internal Medicine	22	9	1231-1238	10.1007/s11606-007-0258-5	Green 2007	Irrelevant	Excluded by TI/AB review
172	Racial differences in battered women's experiences and preferences for treatment from physicians	Hamberger, L. K.; Ambuel, B.; Guse, C. E.	2007	Journal of Family Violence	22	5	259-265	10.1007/s10896-007-9071-5	Hamberger 2007	Irrelevant	Excluded by TI/AB review

S4 Table. All studies identified in the literature search

173	Associations of patient health-related problem solving with disease control, Emergency Department visits, and [illegible]	Hill-Briggs, F.; Gemmell, L.; Kulkarni, B.; Klick, B.; Brancati, F. L.	2007	Journal of General Internal Medicine	22	5	649-654	10.1007/s11606-006-0091-2	Hill-Briggs 2007	Irrelevant	Excluded by TI/AB review
174	The emergency department for routine healthcare: Race/ethnicity, socioeconomic status, and perceptual factors	Hong, R.; Baumann, B. M.; Boudreaux, E. D.	2007	Journal of Emergency Medicine	32	2	149-158	10.1016/j.jemermed.2006.05.042	Hong 2007	Irrelevant	Excluded by TI/AB review
175	Muslim patients and health disparities in the UK and the US	Laird, L. D.; Amer, M. M.; Barnett, E. D.; Barnes, L. L.; Laird, Lance D.; Amer, Mona M.; Barnett, Elizabeth D.; Barnes, Linda L.	2007	Archives of Disease in Childhood	92	10	922-926	10.1136/adc.2006.104364	Laird 2007	Irrelevant	Excluded by TI/AB review

S4 Table. All studies identified in the literature search

176	Prevalence and correlates of erectile dysfunction by race and ethnicity among men aged [illegible]	Laumann, Edward O.; West, Suzanne; Glasser, Dale; Carson, Culley; Rosen, Raymond; Kang, Jeong-han	2007	Journal of Sexual Medicine	4	1	57-65	10.1111/j.1743-6109.2006.00340.x	Laumann 2007	Irrelevant	Excluded by TI/AB review
178	Ethnicity/race and outcome in the treatment of depression: Results from STARD	Lesser, Ira M.; Castro, Daniel B.; Gaynes, Bradley N.; Gonzalez, Jodi; Rush, A. John; Alpert, Jonathan E.; Trivedi, Madhukar; Luther, James F.; [illegible]	2007	Medical Care	45	11	1043-1051	10.1097/MLR.0b013e3181271462	Lesser 2007	Irrelevant	Excluded by TI/AB review
179	Fair Access to and Use of Assessment in Counseling	Loesch, Larry C.; Lee, Courtland C.	2007	Counseling for social justice., 2nd ed.	NA	NA	201-222	NA	Loesch 2007	Irrelevant	Excluded by TI/AB review
180	Cross-Cultural Communication in a Shrinking World	Loitman, Jane E.; Meek, Gloria	2007	PsycCRITIQUES	52	33	NA	10.1037/a0007019	Loitman 2007	Irrelevant	Excluded by TI/AB review

S4 Table. All studies identified in the literature search

181	Erectile dysfunction, discrepancy between high prevalence and low utilization of treatment options: Results from the [illegible]	May, M.; Gralla, O.; Knoll, N.; Fenske, S.; Spivak, I.; R√∂nnebeck, C.; Hoffmann, M.; Lenk, S.; Hoschke, B.	2007	BJU International	100	5	1110-1115	10.1111/j.1464-410X.2007.07101.x	May-07	Irrelevant	Excluded by TI/AB review
182	Use of asthma specialist care in high-risk inner-city Black children	Okelo, S. O.; Patino, C. M.; Hansel, N. N.; Eggleston, P. A.; Curtin-Brosnan, J.; Krishnan, J. A.; Rand, C. S.; Diette, [illegible]	2007	Pediatric Asthma, Allergy & Immunology	20	4	255-262	10.1089/pai.2007.003	Okelo 2007	Irrelevant	Excluded by TI/AB review
183	Comparing attitudes of the public and medical staff towards witnessed resuscitation in an [illegible]	Ong, M. E. H.; Chung, W. L.; Mei, J. S. E.	2007	Resuscitation	73	1	103-108	10.1016/j.resuscitation.2006.08.007	Ong 2007	Irrelevant	Excluded by TI/AB review
184	The impact of long-term conventional treatment for overlap syndrome (obstructive sleep apnea and chronic obstructive pulmonary [illegible]	Perimenis, P.; Karkoulias, K.; Konstantinopoulos, A.; Alchanatis, M.; Perimeni, P. P.; Athanasopoulos, A.; Spyropoulos, K.	2007	Respiratory Medicine	101	2	210-216	10.1016/j.rmed.2006.06.012	Perimenis 2007	Irrelevant	Excluded by TI/AB review

S4 Table. All studies identified in the literature search

185	On supervision: Psychoanalytic and Jungian analytic perspectives	Petts, Ann; Shapley, Bernard	2007	NA	NA	NA	NA	NA	Petts 2007	Irrelevant	Excluded by TI/AB review
186	Provision of pharmaceutical care to patients with limited English proficiency	Phokeo, V.; Hyman, I.	2007	American Journal of Health-System Pharmacy	64	4	423-429	10.2146/ajhp060082	Phokeo 2007	Irrelevant	Excluded by TI/AB review
187	Judging mental disorder in youths: Effects of client, clinician, and contextual differences	Pottick, Kathleen J.; Kirk, Stuart A.; Hsieh, Derek K.; Tian, Xin	2007	Journal of Consulting and Clinical Psychology	75	1	8-Jan	10.1037/0022-006X.75.1.1	Pottick 2007	Irrelevant	Excluded by TI/AB review

S4 Table. All studies identified in the literature search

188	The acoustic characteristics of professional opera singers performing in chorus versus	Reid, K. L. P.; Davis, P.; Oates, J.; Cabrera, D.; Ternstr√∂m, S.; Black, M.; Chapman, J.	2007	Journal of Voice	21	1	35-45	10.1016/j.jvoice.2005.08.010	Reid 2007	Irrelevant	Excluded by TI/AB review
189	Factors associated with reluctance to use an emergency department in a multi-ethnic	Reime, B.; Tu, A. W.; Tzianetas, R.; Ratner, P. A.	2007	Canadian Journal of Public Health	98	3	222-227	NA	Reime 2007	Irrelevant	Excluded by TI/AB review
190	Everything you wanted to know about health care but were afraid to ask	Resnick, Robert J.	2007	PsycCRITIQUES	52	38	NA	10.1037/a0008511	Resnick 2007	Irrelevant	Excluded by TI/AB review
191	Factors underlying racial disparities in hospital care of congestive heart failure	Shen, J. J.; Washington, E. L.; Chung, K.; Bell, R.	2007	Ethnicity & Disease	17	2	206-213	NA	Shen 2007	Irrelevant	Excluded by TI/AB review
192	Emergency department equipment for obese patients: Perceptions of adequacy	Singh, N.; Arthur, H. M.; Worster, A.; Iacobellis, G.; Sharma, A. M.	2007	Journal of Advanced Nursing	59	2	140-145	10.1111/j.1365-2648.2007.04266.x	Singh 2007	Irrelevant	Excluded by TI/AB review

S4 Table. All studies identified in the literature search

193	The demographic bias of Email as a survey method in a pediatric emergency population	Taylor, B. W.	2007	International Journal of Medical Informatics	76	SUPPL. 3	S392-S396	10.1016/j.ijmedinf.2007.04.003	Taylor 2007	Irrelevant	Excluded by TI/AB review
194	Relationship of body mass index with asthma indicators in Head Start children	Vargas, P. A.; Perry, T. T.; Robles, E.; Jo, C. H.; Simpson, P. M.; Magee, J. M.; Feild, C. R.; Hakkak, [illegible]	2007	Annals of Allergy, Asthma & Immunology	99	1	22-28	10.1016/s1081-1206(10)60616-3	Vargas 2007	Irrelevant	Excluded by TI/AB review
195	Challenges & struggles: lived experiences of individuals with mental illness, substance abuse, [illegible]	Villena, A. L. D.	2007	NA	NA	Ph.D.	213 p-213 p	NA	Villena 2007	Irrelevant	Excluded by TI/AB review
196	Patients' complaints in a hospital emergency department in Singapore	Wong, L. L.; Ooi, S. B. S.; Goh, L. G.	2007	Singapore Medical Journal	48	11	990-995	NA	Wong 2007	Irrelevant	Excluded by TI/AB review

S4 Table. All studies identified in the literature search

197	Disparities in adult awareness of heart attack warning signs and symptoms--14 states, 2005	NA	2008	MMWR Morb Mortal Wkly Rep	57	7	175-9	NA	NA	Irrelevant	Excluded by TI/AB review
199	Building better oral health: a dental home for all Texans	NA	2008	Texas dental journal	Suppl	NA	Jan-56	NA	NA	Irrelevant	Excluded by TI/AB review
200	2008 SNRS abstracts -- D - E	NA	2008	Southern Online Journal of Nursing Research	8	4	4-Apr	NA	NA	Irrelevant	Excluded by TI/AB review

S4 Table. All studies identified in the literature search

201	What affects time to care in emergency room appendicitis patients?	NA	2008	Medical Care	46	4	417-422	10.1097/mlr.0b013e31815c1e66	NA	Irrelevant	Excluded by TI/AB review
203	2008 SNRS abstracts -- L	NA	2008	Southern Online Journal of Nursing Research	8	4	8-Aug	NA	NA	Irrelevant	Excluded by TI/AB review
204	2008 SNRS abstracts -- H	NA	2008	Southern Online Journal of Nursing Research	8	4	6-Jun	NA	NA	Irrelevant	Excluded by TI/AB review

S4 Table. All studies identified in the literature search

205	Stuck in the mud	Allen, Richard E.	2008	Annals of Family Medicine	6	1	80-82	10.1370/afm.756	Allen 2008	Irrelevant	Excluded by TI/AB review
206	Differences in the patterns of health care system distrust between blacks and whites	Armstrong, K.; McMurphy, S.; Dean, L. T.; Micco, E.; Putt, M.; Halbert, C. H.; Schwartz, J. S.; Sankar, P.; [illegible]	2008	J Gen Intern Med	23	6	827-33	10.1007/s11606-008-0561-9	Armstrong 2008	Irrelevant	Excluded by TI/AB review
208	Doctor's perception of doctor-patient relationships in emergency departments: What roles do gender [illegible]	Babitsch, B.; Braun, T.; Borde, T.; David, M.; Babitsch, Birgit; Braun, Tanja; Borde, Theda; David, Matthias	2008	BMC Health Services Research	8	NA	82-82	10.1186/1472-6963-8-82	Babitsch 2008	Irrelevant	Excluded by TI/AB review
209	Culture-specific programs for children and adults from minority groups who have [illegible]	Bailey, E. J.; Kruske, S. G.; Morris, P. S.; Cates, C. J.; Chang, A. B.	2008	Cochrane Database of Systematic Reviews	NA	2	NA	10.1002/14651858.CD006580.pub2	Bailey 2008	Irrelevant	Excluded by TI/AB review

S4 Table. All studies identified in the literature search

210	Impact of sexual assault nurse examiners on the evaluation of sexual assault in a pediatric emergency department	Bechtel, K.; Ryan, E.; Gallagher, D.	2008	Pediatric Emergency Care	24	7	442-447	10.1097/PEC.0b013e31817de11d	Bechtel 2008	Irrelevant	Excluded by TI/AB review
211	Why Do Parents Bring Children to the Emergency Department for	Berry, A.; Brousseau, D.; Brotanek, J. M.; Tomany-Korman, S.;	2008	Ambulatory Pediatrics	8	6	360-367	10.1016/j.ambp.2008.07.001	Berry 2008	Irrelevant	Excluded by TI/AB review
212	Access to appointments based on insurance status in Washington, D.C	Blanchard, J.; Ogle, K.; Thomas, O.; Lung, D.; Asplin, B.; Lurie, N.	2008	Journal of Health Care for the Poor and Underserved	19	3	687-696	10.1353/hpu.0.0036	Blanchard 2008	Irrelevant	Excluded by TI/AB review
213	Systems Training for Emotional Predictability and Problem Solving (STEPPS) for outpatients with borderline [illegible]	Blum, N.; St John, D.; Pfohl, B.; Stuart, S.; McCormick, B.; Allen, J.; Arndt, S.; Black, D. W.; Blum, Nancee; St John, Don; Pfohl, Bruce; [illegible]	2008	American Journal of Psychiatry	165	4	468-478	10.1176/appi.ajp.2007.07071079	Blum 2008	Irrelevant	Excluded by TI/AB review

S4 Table. All studies identified in the literature search

215	A participatory action research pilot study of urban health disparities using rapid assessment response and evaluation	Brown, D. R.; Hern√°ndez, A.; Saint-Jean, G.; Evans, S.; Tafari, I.; Brewster, L. G.; Celestin, M. J.; G√≥mez-Estefan, C.; Regalado, F.; Akal, [illegible]	2008	American Journal of Public Health	98	1	28-38	10.2105/AJPH.2006.091363	Brown 2008	Irrelevant	Excluded by TI/AB review
216	Patient perceptions and acceptance of routine emergency department HIV testing	Brown, J.; Kuo, I.; Bellows, J.; Barry, R.; Bui, P.; Wohlgemuth, J.; Wills, E.; Parikh, N.	2008	Public Health Reports	123	SUPPL. 3	21-26	10.1177/00333549081230s304	Brown 2008	Irrelevant	Excluded by TI/AB review
217	Improving antibiotic prescribing for adults with community [illegible]	Buising, K. L.; Thursky, K. A.; Black, J. F.; MacGregor, L.; Street, A. C.; [illegible]	2008	BMC Medical Informatics and Decision [illegible]	8	NA	NA	10.1186/1472-6947-8-35	Buising 2008	Irrelevant	Excluded by TI/AB review
218	Strategies to improve acute stroke care of patients in rural and other geographically [illegible]	Callison, R. C.; Leira, E. C.	2008	Current Treatment Options in Neurology	10	6	450-454	10.1007/s11940-008-0047-4	Callison 2008	Irrelevant	Excluded by TI/AB review

S4 Table. All studies identified in the literature search

219	Omalizumab treatment in children 6 to 18 years old with severe asthma at a children's medical center	Campbell, J. M.; Wofford, J. D.; Knutsen, A. P.	2008	Pediatric Asthma, Allergy & Immunology	21	3	129-135	10.1089/pai.2008.0502	Campbell 2008	Irrelevant	Excluded by TI/AB review
220	Society of Chest Pain Centers accreditation improves ACC/AHA guidelines compliance for [illegible]	Chandra, A.; Peacock, W. F.; McCord, J. K.; Ou, F.; Roe, M. T.; Peterson, E. D.; Ohman, M.; Gibler, W. B.; Cairns, C. B.	2008	Critical Pathways in Cardiology	7	3	197	10.1097/HPC.0b013e318188d93e	Chandra 2008	Irrelevant	Excluded by TI/AB review
221	Oxycodone versus codeine for triage pain in children with suspected forearm fracture: A randomized controlled trial	Charney, R. L.; Yan, Y.; Schootman, M.; Kennedy, R. M.; Luhmann, J. D.	2008	Pediatric Emergency Care	24	9	595-600	10.1097/PEC.0b013e3181850ca3	Charney 2008	Irrelevant	Excluded by TI/AB review

S4 Table. All studies identified in the literature search

222	Gender disparity in analgesic treatment of emergency department patients with acute [illegible]	Chen, E. H.; Shofer, F. S.; Dean, A. J.; Hollander, J. E.; Baxt, W. G.; Robey, J. L.; Sease, K. L.; Mills, A. M.	2008	Acad Emerg Med	15	5	414-8	10.1111/j.1553-2712.2008.00100.x	Chen 2008	Irrelevant	Excluded by TI/AB review
226	Considerations in culturally directed asthma disease management	Cloutier, M. M.	2008	Disease Management & Health Outcomes	16	2	95-105	10.2165/00115677-200816020-00004	Cloutier 2008	Irrelevant	Excluded by TI/AB review
227	Toothache pain: a comparison of visits to physicians, emergency [illegible]	Cohen, L. A.; Bonito, A. J.; Akin, D. R.; Manski, R. J.; Macek, M. D.; Edwards, R. R.; [illegible]	2008	J Am Dent Assoc	139	9	1205-16	10.14219/jada.archive.2008.0336	Cohen 2008	Irrelevant	Excluded by TI/AB review
230	Provocateur: Images of women and minorities in advertising, 3rd ed	Cortese, Anthony J.	2008	NA	NA	NA	NA	NA	Cortese 2008	Irrelevant	Excluded by TI/AB review

S4 Table. All studies identified in the literature search

231	Assessment of "best practice" treatment patterns for a "radiation oncology community outreach group" engaged in cancer disparities outcomes	de Andrade, R. S.; Proctor, J. W.; Rakfal, S. M.; Werts, E. D.; Schenken, L. L.; Saw, C. B.; Dougherty, M.; Stefanik, D.; de Andrade, Regiane S.; Proctor, Julian W.; Rakfal, Susan M.; Werts, E. Day; Schenken, Larry L.;	2008	Journal of the American College of Radiology	5	4	571-578	10.1016/j.jacr.2007.10.005	deAndrade 2008	Irrelevant	Excluded by TI/AB review
232	Treatment of lesbian, gay, bisexual, and transgender patients	Drescher, Jack; McCommon, Benjamin H.; Jones, Billy E.; Hales, Robert E.; Yudofsky,	2008	The American Psychiatric Publishing textbook of	NA	NA	1471-1487	NA	Drescher 2008	Irrelevant	Excluded by TI/AB review
233	Triage presenting complaint descriptions bias emergency	Dutch, M. J.; Taylor, D. M.; Dent, A. W.	2008	Academic Emergency Medicine	15	8	731-735	10.1111/j.1553-2712.2008.00177.x	Dutch 2008	Irrelevant	Excluded by TI/AB review

S4 Table. All studies identified in the literature search

234	Ethnic wait time differences in analgesic administration in the emergency department	Epps, C. D.; Ware, L. J.; Packard, A.	2008	Pain Manag Nurs	9	1	26-32	10.1016/j.pmn.2007.07.005	Epps 2008	Irrelevant	Excluded by TI/AB review
237	Language disparities and timely care for children in managed care Medicaid	Galbraith, A. A.; Semura, J. I.; McAninch-Dake, R. J.; Anderson, N.; Christakis, D. A.	2008	American Journal of Managed Care	14	7	417-426	NA	Galbraith 2008	Irrelevant	Excluded by TI/AB review
238	Cultural issues	Gaw, Albert C.; Hales, Robert E.; Yudofsky, Stuart C.;	2008	The American Psychiatric	NA	NA	1530-1554	NA	Gaw 2008	Irrelevant	Excluded by TI/AB review

S4 Table. All studies identified in the literature search

239	Compliance with recommended cancer screening among emergency	Ginde, A. A.; Millen, J. C.; Love, J. S.; Pang, J. M.; Camargo Jr, C. A.	2008	Academic Emergency Medicine	15	5	483-486	10.1111/j.1553-2712.2008.00103.x	Ginde 2008	Irrelevant	Excluded by TI/AB review
240	Quality of Pharmacotherapy and Outcomes for Older Veterans Discharged from the Emergency Department	Hastings, S. N.; Schmader, K. E.; Sloane, R. J.; Weinberger, M.; Pieper, C. F.; Goldberg, K. C.; Oddone, E. Z.	2008	Journal of the American Geriatrics Society	56	5	875-880	10.1111/j.1532-5415.2008.01648.x	Hastings 2008	Irrelevant	Excluded by TI/AB review
241	Black intimacies: Love, sex, and relationships--The pursuit of intimacy	Hill, Shirley A.; Spade, Joan Z.; Valentine, Catherine G.	2008	The kaleidoscope of gender: Prisms, patterns, and possibilities., 2nd ed.	NA	NA	414-426	NA	Hill 2008	Irrelevant	Excluded by TI/AB review

S4 Table. All studies identified in the literature search

242	Patient race/ethnicity, age, gender and education are not related to preference for or response to [illegible]	Hobgood, C.; Tamayo-Sarver, J. H.; Weiner, B.	2008	Quality and Safety in Health Care	17	1	65-70	10.1136/qshc.2006.021758	Hobgood 2008	Irrelevant	Excluded by TI/AB review
243	Cultural and linguistic competence and eliminating disparities	Isaacs, Mareasa R.; Jackson, Vivian Hopkins; Hicks, Regenia; Wang, Ed K. S.; Stroul, Beth A.; Blau, Gary M.	2008	The system of care handbook: Transforming mental health services for children, youth, and families.	NA	NA	301-328	NA	Isaacs 2008	Irrelevant	Excluded by TI/AB review
249	A plan for action: Key perspectives from the racial/ethnic disparities strategy forum	King, R. K.; Green, A. R.; Tan-Mcgrory, A.; Donahue, E. J.; Kimbrough-Sugick, J.; Betancourt, J. R.	2008	Milbank Quarterly	86	2	241-272	10.1111/j.1468-0009.2008.00521.x	King 2008	Irrelevant	Excluded by TI/AB review

S4 Table. All studies identified in the literature search

250	Disclosing intimate partner violence to health care clinicians - What a difference the setting makes: A qualitative study	Liebschutz, J.; Battaglia, T.; Finley, E.; Averbuch, T.	2008	BMC Public Health	8	NA	NA	10.1186/1471-2458-8-229	Liebschutz 2008	Irrelevant	Excluded by TI/AB review
252	Cosmetic outcomes of absorbable versus nonabsorbable sutures in pediatric facial lacerations	Luck, R. P.; Flood, R.; Eyal, D.; Saludades, J.; Hayes, C.; Gaughan, J.	2008	Pediatric Emergency Care	24	3	137-142	10.1097/PEC.0b013e3181666f87	Luck 2008	Irrelevant	Excluded by TI/AB review
254	High prevalence of triple-negative tumors in an urban cancer center	Lund, M. J. B.; Butler, E. N.; Bumpers, H. L.; Okoli, J.; Rizzo, M.; Hatchett, N.; Green,	2008	Cancer	113	3	608-615	10.1002/cncr.23569	Lund 2008	Irrelevant	Excluded by TI/AB review
255	Vulnerable older people in the community: Relationship between the vulnerable elders [illegible]	McGee, Hannah M.; O'Hanlon, Ann; Barker, Maja; Hickey, Anne; Montgomery, Anthony; Conroy, [illegible]	2008	Journal of the American Geriatrics Society	56	1	15-Aug	10.1111/j.1532-5415.2007.01540.x	McGee 2008	Irrelevant	Excluded by TI/AB review

S4 Table. All studies identified in the literature search

256	Racial disparities in access to care for men in a public assistance program for prostate cancer	Miller, D. C.; Gelberg, L.; Kwan, L.; Stepanian, S.; Fink, A.; Andersen, R. M.; Litwin, M. S.	2008	Journal of Community Health	33	5	318-335	10.1007/s10900-008-9105-9	Miller 2008	Irrelevant	Excluded by TI/AB review
259	Does poorer familiarity with medicare translate into worse access to health care?	Morgan, R. O.; Teal, C. R.; Hasche, J. C.; Petersen, L. A.; Byrne, M. M.; Paterniti, D. A.; [illegible]	2008	Journal of the American Geriatrics Society	56	11	2053-2060	10.1111/j.1532-5415.2008.01993.x	Morgan 2008	Irrelevant	Excluded by TI/AB review
261	Estimating the empirical Lorenz curve and Gini coefficient in the	Moskowitz, C. S.; Seshan, V. E.; Riedel, E. R.; Begg, C. B.; Moskowitz,	2008	Statistics in Medicine	27	16	3191-3208	10.1002/sim.3151	Moskowitz 2008	Irrelevant	Excluded by TI/AB review
262	The effect of patient origin and relevance of contact on patient and caregiver satisfaction in the	Mygind, A.; Norredam, M.; Nielsen, A. S.; Bagger, J.; Krasnik, A.	2008	Scandinavian Journal of Public Health	36	1	76-83	10.1177/1403494807085302	Mygind 2008	Irrelevant	Excluded by TI/AB review
263	Clinical microsystems, part 1. The building blocks of health systems	Nelson, E. C.; Godfrey, M. M.; Batalden, P. B.; Berry, S. A.; Bothe, A. E., Jr.; McKinley, K. E.; Melin, C. N.; [illegible]	2008	Joint Commission Journal on Quality & Patient Safety	34	7	367-378	NA	Nelson 2008	Irrelevant	Excluded by TI/AB review

S4 Table. All studies identified in the literature search

264	Comparison of acute pain relief, after intravenous morphine administration, among different ethnic groups who [illegible]	Nik Hisamuddin, N. A. R.; Kamaruddin, J.; Idzwan, Z. M.; Rashidi, A.	2008	Journal of Emergency Medicine, Trauma and Acute Care	8	2	73-77	NA	NikHisamuddin 2008	Irrelevant	Excluded by TI/AB review
265	Case study: an ethical dilemma involving a dying patient	Pacsi, A. L.	2008	The Journal of the New York State Nurses' Association	39	1	7-Apr	NA	Pacsi 2008	Irrelevant	Excluded by TI/AB review
266	Is consent "informed" when patients receive care from medical trainees?	Pallin, D. J.; Harris, R.; Johnson, C. I.; Giraldez, E.	2008	Academic Emergency Medicine	15	12	1304-1308	10.1111/j.1553-2712.2008.00269.x	Pallin 2008	Irrelevant	Excluded by TI/AB review

S4 Table. All studies identified in the literature search

267	Access to Care Issues for African American Communities: Implications for STD Disparities	Parrish, D. D.; Kent, C. K.	2008	Sexually Transmitted Diseases	35	12	S19-S22	10.1097/OLQ.0b013e31818f2ae1	Parrish 2008	Irrelevant	Excluded by TI/AB review
268	Language interpreter utilization in the emergency department setting: A clinical review	Ramirez, D.; Engel, K. G.; Tang, T. S.	2008	Journal of Health Care for the Poor and Underserved	19	2	352-362	10.1353/hpu.0.0019	Ramirez 2008	Irrelevant	Excluded by TI/AB review
270	Inter-rater reliability of data elements from a prototype of the Paul Coverdell National Acute Stroke Registry	Reeves, M. J.; Mullard, A. J.; Wehner, S.	2008	BMC Neurology	8	NA	NA	10.1186/1471-2377-8-19	Reeves 2008	Irrelevant	Excluded by TI/AB review

S4 Table. All studies identified in the literature search

273	Ethnic/Racial diversity and posttraumatic distress in the	Santos, M. R.; Russo, J.; Aisenberg, G.; Uehara, E.; Ghesquiere, A.;	2008	Psychiatry	71	3	234-45	10.1521/psyc.2008.71.3.234	Santos 2008	Irrelevant	Excluded by TI/AB review
274	The kaleidoscope of gender: Prisms, patterns, and possibilities, 2nd ed	Spade, Joan Z.; Valentine, Catherine G.	2008	NA	NA	NA	NA	NA	Spade 2008	Irrelevant	Excluded by TI/AB review
275	Preferences for cardiac tests and procedures may partially explain sex but not race disparities	Takakuwa, K. M.; Shofer, F. S.; Limkakeng, A. T., Jr.; Hollander, J. E.	2008	Am J Emerg Med	26	5	545-50	10.1016/j.ajem.2007.08.020	Takakuwa 2008	Irrelevant	Excluded by TI/AB review
279	'Race' matters: racialization and egalitarian discourses involving	Tang, S. Y.; Browne, A. J.	2008	Ethnicity & health	13	2	109-127	10.1080/13557850701830307	Tang 2008	Irrelevant	Excluded by TI/AB review

S4 Table. All studies identified in the literature search

280	Factors associated with longer time from symptom onset to hospital presentation for [illegible]	Ting, H. H.; Bradley, E. H.; Wang, Y.; Lichtman, J. H.; Nallamothu, B. K.; Sullivan, M. D.; [illegible]	2008	Arch Intern Med	168	9	959-68	10.1001/archinte.168.9.959	Ting 2008	Irrelevant	Excluded by TI/AB review
281	Racial and cultural aspects of psychotherapy and supervision	Toldson, Ivory A.; Utsey, Shawn; Hess, Allen K.; Hess, Kathryn D.; Hess, Tanya H.	2008	Psychotherapy supervision: Theory, research, and [illegible]	NA	NA	537-559	NA	Toldson 2008	Irrelevant	Excluded by TI/AB review
282	Are there racial and ethnic differences in the administration of analgesia?	Willens, Joyce S.	2008	Pain Management Nursing	9	2	45-45	10.1016/j.pmn.2008.04.001	Willens 2008	Irrelevant	Excluded by TI/AB review
283	Racial differences in follow-up of abnormal mammography findings among economically disadvantaged women	Adams, S. A.; Smith, E. R.; Hardin, J.; Prabhu-Das, I.; Fulton, J.; Hebert, J. R.; Adams, Swann A.; Smith, Emily R.; Hardin, James; Prabhu-Das, Irene; Fulton, Jeanette; [illegible]	2009	Cancer (0008543X)	115	24	5788-5797	10.1002/cncr.24633	Adams 2009	Irrelevant	Excluded by TI/AB review

S4 Table. All studies identified in the literature search

284	Advances in adult asthma diagnosis and treatment and health outcomes, education, delivery, and quality in 2008	Apter, A. J.	2009	Journal of Allergy and Clinical Immunology	123	1	35-40	10.1016/j.jaci.2008.11.017	Apter 2009	Irrelevant	Excluded by TI/AB review
285	Patient characteristics associated with improved outcomes with use of an inhaled [illegible]	Bacharier, L. B.; Guilbert, T. W.; Zeiger, R. S.; Strunk, R. C.; Morgan, W. J.; Lemanske Jr, R. F.; Moss, M.; Szefler, S. [illegible]	2009	Journal of Allergy and Clinical Immunology	123	5	1077-1082.e5	10.1016/j.jaci.2008.12.1120	Bacharier 2009	Irrelevant	Excluded by TI/AB review
286	Culture-specific programs for children and adults from minority groups who have asthma	Bailey, E. J.; Cates, C. J.; Kruske, S. G.; Morris, P. S.; Brown, N.; Chang, A. B.	2009	Cochrane Database of Systematic Reviews	NA	2	NA	10.1002/14651858.CD006580.pub4	Bailey 2009	Irrelevant	Excluded by TI/AB review
287	House calls for seniors: Building and sustaining a model of care for homebound seniors	Beck, R. A.; Arizmendi, A.; Purnell, C.; Fultz, B. A.; Callahan, C. M.	2009	Journal of the American Geriatrics Society	57	6	1103-1109	10.1111/j.1532-5415.2009.02278.x	Beck 2009	Irrelevant	Excluded by TI/AB review

S4 Table. All studies identified in the literature search

288	Lower copay and oral administration: Predictors of first-fill adherence to new asthma [illegible]	Berger, Z.; Kimbrough, W.; Gillespie, C.; Boscarino, J. A.; Wood, G. C.; Qian, Z.; Jones, J. B.; [illegible]	2009	American Health and Drug Benefits	2	4	174-179	NA	Berger 2009	Irrelevant	Excluded by TI/AB review
289	Feasibility of an interactive voice response tool for adolescent assault victims	Blackstone, M. M.; Wiebe, D. J.; Mollen, C. J.; Kalra, A.; Fein, J. A.	2009	Academic Emergency Medicine	16	10	956-962	10.1111/j.1553-2712.2009.00519.x	Blackstone 2009	Irrelevant	Excluded by TI/AB review
290	Substance use disorders in the African American community	Bonhomme, Jean J. E.; Braithwaite, Ronald L.; Moore, Melita; Braithwaite, Ronald L.; Taylor, Sandra E.; Treadwell, Henrie M.	2009	Health issues in the Black community., 3rd ed.	NA	NA	345-361	NA	Bonhomme 2009	Irrelevant	Excluded by TI/AB review
291	Reproducing race: An ethnography of pregnancy as a site of racialization	Bridges, Khiara M.	2009	NA	69	NA	4007-4007	NA	Bridges 2009	Irrelevant	Excluded by TI/AB review

S4 Table. All studies identified in the literature search

292	Impact of a household environmental intervention delivered by lay health workers on asthma symptom control in urban, disadvantaged children with [illegible]	Bryant-Stephens, T.; Kurian, C.; Guo, R.; Zhao, H.	2009	American Journal of Public Health	99 Suppl 3	NA	S657-665	NA	Bryant-Stephens 2009	Irrelevant	Excluded by TI/AB review
293	A framework for cross-cultural practice in school settings	Caple, Frances Smalls; Salcido, Ramon M.; Massat, Carol Rippey; Constable, Robert; McDonald, Shirley; Flynn, John P.	2009	School social work: Practice, policy, and research., 7th ed.	NA	NA	339-361	NA	Caple 2009	Irrelevant	Excluded by TI/AB review
294	Racial Differences in Trust and Regular Source of Patient Care and the Implications for Prostate [illegible]	Carpenter, W. R.; Godley, P. A.; Clark, J. A.; Talcott, J. A.; Finnegan, T.; Mishel, M.; Bensen, J.; Rayford, W.; Su, L. [illegible]	2009	Cancer	115	21	5048-5059	10.1002/cncr.24539	Carpenter 2009	Irrelevant	Excluded by TI/AB review
295	Race/Ethnicity differences in the inpatient management of acute asthma in the United States	Chandra, D.; Clark, S.; Camargo, C. A., Jr.	2009	Chest	135	6	1527-1534	10.1378/chest.08-1812	Chandra 2009	Irrelevant	Excluded by TI/AB review

S4 Table. All studies identified in the literature search

296	Health care quality-improvement approaches to reducing child health disparities	Chin, M. H.; Alexander-Young, M.; Burnet, D. L.	2009	Pediatrics	124	SUPPL. 3	S224-S236	10.1542/peds.2009-1100K	Chin 2009	Irrelevant	Excluded by TI/AB review
297	Primary Household Language and Asthma Care among Latino [illegible]	Claudio, L.; Stingone, J. A.	2009	Journal of Health Care for the Poor and Underserve[illegible]	20	3	766-779	NA	Claudio 2009	Irrelevant	Excluded by TI/AB review
298	Low-income and minority patient satisfaction with visits to emergency departments and physician offices for dental [illegible]	Cohen, L. A.; Harris, S. L.; Bonito, A. J.; Manski, R. J.; Macek, M. D.; Edwards, R. R.; Khanna, N.; Plowden, K. O.	2009	The Journal of the American College of Dentists	76	3	23-31	NA	Cohen 2009	Irrelevant	Excluded by TI/AB review
299	Low-risk acute heart failure patients: External validation of the society of chest [illegible]	Collins, S. P.; Lindsell, C. J.; Naftilan, A. J.; Peacock, W. F.; Diercks, D.; [illegible]	2009	Critical Pathways in Cardiology	8	3	99-103	10.1097/HPC.0b013e3181b5a534	Collins 2009	Irrelevant	Excluded by TI/AB review

S4 Table. All studies identified in the literature search

300	A randomized controlled trial of interventions to enhance patient-physician	Cooper, L. A.; Roter, D. L.; Bone, L. R.; Larson, S. M.; Miller, E. R., 3rd; Barr, M. S.; Carson,	2009	Implementation Science	4	NA	7-Jul	10.1186/1748-5908-4-7	Cooper 2009	Irrelevant	Excluded by TI/AB review
301	Race and racism	Durrheim, Kevin; Hook, Derek; Riggs, Damien W.; Fox, Dennis; Prilleltensky, Isaac; Austin, Stephanie	2009	Critical psychology: An introduction., 2nd ed.	NA	NA	197-214	NA	Durrheim 2009	Irrelevant	Excluded by TI/AB review
302	Cross cultural awareness and social justice in counseling	Ellis, Cyrus Marcellus; Carlson, Jon	2009	NA	NA	NA	NA	NA	Ellis 2009	Irrelevant	Excluded by TI/AB review
303	Disparities in asthma care and outcome	Emmett, G. A.; Rothkopf, A. C.	2009	International Journal of Child & Adolescent Health	2	1	13-18	NA	Emmett 2009	Irrelevant	Excluded by TI/AB review

S4 Table. All studies identified in the literature search

305	Improving asthma outcomes in minority children: a randomized, controlled trial of parent mentors	Flores, G.; Bridon, C.; Torres, S.; Perez, R.; Walter, T.; Brotanek, J.; Lin, H.; Tomany-Korman, S.	2009	Pediatrics	124	6	1522-32	10.1542/peds.2009-0230	Flores 2009	Irrelevant	Excluded by TI/AB review
310	Urban minority children with asthma: substantial morbidity, compromised quality and access to specialists, and [illegible]	Flores, G.; Snowden-Bridon, C.; Torres, S.; Perez, R.; Walter, T.; Brotanek, J.; Lin, H.; Tomany-Korman, S.	2009	J Asthma	46	4	392-8	10.1080/02770900802712971	Flores 2009	Irrelevant	Excluded by TI/AB review
312	Predictors of retention in HIV care among a national cohort of US veterans	Giordano, T. P.; Hartman, C.; Gifford, A. L.; Backus, L. I.; Morgan, R. O.	2009	HIV Clinical Trials	10	5	299-305	10.1310/hct1005-299	Giordano 2009	Irrelevant	Excluded by TI/AB review
313	Scalds in pediatric emergency department: A 5-year experience	Guzel, A.; Aksu, B.; Aylan√ß, H.; Duran, R.; Karasalihoglu, S.	2009	Journal of Burn Care and Research	30	3	450-456	10.1097/BCR.0b013e3181a28cac	Guzel 2009	Irrelevant	Excluded by TI/AB review

S4 Table. All studies identified in the literature search

315	Demographic influences and use of demographically corrected norms in neuropsychological assessment	Heaton, Robert K.; Ryan, Lee; Grant, Igor; Grant, Igor; Adams, Kenneth M.	2009	Neuropsychological assessment of neuropsychiatric and neuromedical disorders., 3rd ed.	NA	NA	127-155	NA	Heaton 2009	Irrelevant	Excluded by TI/AB review
316	Ensuring color-blind care	Heins, A.	2009	Emergency Medicine (00136654)	41	3	15-17	NA	Heins 2009	Irrelevant	Excluded by TI/AB review
317	Physician ethnicity predicts successful ED analgesia	Heins, A.; Homel, P.; Safdar, B.; Todd, K.	2009	Journal of Pain	10	4	S34	10.1016/j.jpain.2009.01.145	Heins 2009	Irrelevant	Excluded by TI/AB review

S4 Table. All studies identified in the literature search

318	Hurt Running from Police? No Chance of (Pain) Relief: The Social Construction of Deserving Patients in Emergency Departments	Hinze, S. W.; Webster, N. J.; Chirayath, H. T.; Tamayo-Sarver, J. H.	2009	Social Sources of Disparities in Health and Health Care and Linkages to Policy, Population [illegible]	27	NA	235-261	NA	Hinze 2009	Irrelevant	Excluded by TI/AB review
319	New-onset geriatric epilepsy care: Race, setting of diagnosis, and choice of antiepileptic drug	Hope, O. A.; Zeber, J. E.; Kressin, N. R.; Bokhour, B. G.; Vancott, A. C.; Cramer, J. A.; Amuan, M. E.; [illegible]	2009	Epilepsia	50	5	1085-1093	10.1111/j.1528-1167.2008.01892.x	Hope 2009	Irrelevant	Excluded by TI/AB review
321	Accidents waiting to happen: Decreasing access to emergency departments in rural areas in the [illegible]	Hsia, R. Y.; Shen, Y.	2009	Annals of Emergency Medicine	54	3	S85	NA	Hsia 2009	Irrelevant	Excluded by TI/AB review
322	Population differences in breast cancer: survey in indigenous African women [illegible]	Huo, D.; Ikpatt, F.; Khramtsov, A.; Dangou, J. M.; Nanda, R.; Dignam, J.; Zhang, B.; Grushko, T.; Zhang, [illegible]	2009	Journal of Clinical Oncology	27	27	4515-4521	10.1200/JCO.2008.19.6873	Huo 2009	Irrelevant	Excluded by TI/AB review

S4 Table. All studies identified in the literature search

323	Paying for quality care: implications for racial and ethnic health disparities in pediatric asthma	James, C. V.; Rosenbaum, S.	2009	Pediatrics	123 Suppl 3	NA	S205-10	10.1542/peds.2008-2233L	James 2009	Irrelevant	Excluded by TI/AB review
324	Self-reported influences of hopelessness, health literacy, lifestyle action, [illegible]	Joyner-Grantham, J.; Mount, D. L.; McCorkle, O. D.; Simmons, D. R.; Ferrario, C. M.; [illegible]	2009	American Journal of the Medical Sciences	338	5	368-372	10.1097/MAJ.0b013e3181b473dc	Joyner-Grantham 2009	Irrelevant	Excluded by TI/AB review
325	Prevalence of metabolic syndrome and differences in health habits among sufferers among U.S. ethnic groups	Kannan, H.; Wagner, S.; Bolge, S. C.	2009	Value in Health	12	3	A131	10.1111/j.1524-4733.2009.00537-2.x	Kannan 2009	Irrelevant	Excluded by TI/AB review
326	African American men and self-efficacy in preventing prostate cancer	Kendrick, Lorna; Montgomery, Susanne; Ouattara, Dorena; Flaskerud, Jacquelyn H.	2009	Issues in Mental Health Nursing	30	5	342-343	10.1080/01612840902754669	Kendrick 2009	Irrelevant	Excluded by TI/AB review

S4 Table. All studies identified in the literature search

327	Ethnic Variation in Acute Myocardial Infarction Presentation and Access to Care	King, K. M.; Khan, N. A.; Quan, H.	2009	American Journal of Cardiology	103	10	1368-1373	10.1016/j.amjcard.2009.01.344	King 2009	Irrelevant	Excluded by TI/AB review
328	Physician and patient satisfaction with the new AMS 700	Knoll, L. D.; Henry, G.; Culkin, D.; Ohl, D. A.; Otheguy, J.; Shabsigh, R.;	2009	Journal of Sexual Medicine	6	6	1773-1778	10.1111/j.1743-6109.2009.01251.x	Knoll 2009	Irrelevant	Excluded by TI/AB review
329	Couples, gender, and power: Creating change in intimate relationships	Knudson-Martin, Carmen; Mahoney, Anne Rankin	2009	NA	NA	NA	NA	10.1891/978082611756 4	Knudson-Martin 2009	Irrelevant	Excluded by TI/AB review
330	Cultural proficiency in addressing health disparities	Kosoko-Lasaki, Sade; Cook, Cynthia Theresa; O'Brien, Richard L.	2009	NA	NA	NA	NA	NA	Kosoko-Lasaki 2009	Irrelevant	Excluded by TI/AB review

S4 Table. All studies identified in the literature search

331	Barriers to asthma management among urban families: Caregiver and child perspectives	Laster, N.; Holsey, C. N.; Shendell, D. G.; McCarty, F. A.; Celano, M.	2009	Journal of Asthma	46	7	731-739	10.1080/02770900903082571	Laster 2009	Irrelevant	Excluded by TI/AB review
332	Current Adolescent Health Services, Settings, and Providers	Lawrence, R. S.	2009	Adolescent Health Services: Missing Opportunities	NA	NA	135-193	NA	Lawrence 2009	Irrelevant	Excluded by TI/AB review
333	Do prolonged emergency department waiting times reduce emergency research consent rates?	Limkakeng, A. T.; Glickman, S. W.; Freeman, D.; Drake, W.; Mani, G.; Chandra, A.; Cairns, C. B.	2009	Annals of Emergency Medicine	54	3	S49-S50	NA	Limkakeng 2009	Irrelevant	Excluded by TI/AB review

S4 Table. All studies identified in the literature search

334	Emergency department boarding is associated with higher medication-related errors but fewer laboratory errors during the early admission period	Liu, S. W.; Chang, Y.; Weissman, J.; Griffey, R.; Hamedani, A.; Thomas, J.; Nergui, S.; Singer, S.	2009	Annals of Emergency Medicine	54	3	S66	NA	Liu 2009	Irrelevant	Excluded by TI/AB review
335	Couples' communication and congruence of their perceptions about ED and PDE5 inhibitor use: Findings from a qualitative study	McGraw, S. A.; Cameron, A.; Rosen, R.; Mason, E.; Elstad, E.; Wong, D.; Yurgin, N.	2009	Journal of Sexual Medicine	6	NA	74	10.1111/j.1743-6109.2008.01125-1.x	McGraw 2009	Irrelevant	Excluded by TI/AB review
336	Head injury attenders in the emergency department: The impact of advice and factors associated with [illegible]	McMillan, T. M.; McKenzie, P.; Swann, I. J.; Weir, C. J.; McAviney, A.	2009	Brain Injury	23	6	509-515	10.1080/02699050902970729	McMillan 2009	Irrelevant	Excluded by TI/AB review

S4 Table. All studies identified in the literature search

337	Utilization of Medical Treatments and Adherence to Antiretroviral Therapy among HIV-Positive Adults with Histories of Childhood Sexual Abuse	Meade, C. S.; Hansen, N. B.; Kochman, A.; Sikkema, K. J.	2009	AIDS Patient Care and STDs	23	4	259-266	10.1089/apc.2008.0210	Meade 2009	Irrelevant	Excluded by TI/AB review
338	Variation in prehospital and discharge quality of care among acute myocardial infarction patients at a safety net hospital	Mehler, P. S.; Sabel, A.; Trantanella, A.; Krantz, M. J.	2009	Crit Pathw Cardiol	8	3	127-30	10.1097/HPC.0b013e3181b720c3	Mehler 2009	Irrelevant	Excluded by TI/AB review
339	HIV testing in US EDs, 1993-2004	Merchant, R. C.; Catanzaro, B. M.	2009	American Journal of Emergency Medicine	27	7	868-874	10.1016/j.ajem.2008.06.019	Merchant 2009	Irrelevant	Excluded by TI/AB review
340	Characteristics and Predictors of Frequent	Milbrett, P.; Halm, M.	2009	Journal of Emergency Nursing	35	3	191-198	10.1016/j.jen.2008.04.032	Milbrett 2009	Irrelevant	Excluded by TI/AB review

S4 Table. All studies identified in the literature search

342	Adolescents and asthma: Why bother with our meds?	Naimi, D. R.; Freedman, T. G.; Ginsburg, K. R.; Bogen, D.; Rand, C. S.; Apter, A. J.	2009	Journal of Allergy and Clinical Immunolog y	123	6	1335-1341	10.1016/j.jaci.2009.02.022	Naimi 2009	Irrelevant	Excluded by TI/AB review
343	Fibrinolytics for acute myocardial infarction in emergency departments	Niska, R. W.	2009	Annals of Emergency Medicine	54	3	S7	NA	Niska 2009	Irrelevant	Excluded by TI/AB review
344	Emergency medical practice: advancing cultural competence and reducing health care disparities	Padela, A. I.; Punekar, I. R.	2009	Acad Emerg Med	16	1	69-75	10.1111/j.1553-2712.2008.00305.x	Padela 2009	Irrelevant	Excluded by TI/AB review
348	Retrospective audit of Taranaki children undergoing dental care under general [illegible]	Page, L. A. F.	2009	New Zealand Dental Journal	105	1	12-Aug	NA	Page 2009	Irrelevant	Excluded by TI/AB review
349	Survey of medical decisionmaking: Ability of the public to self-triage and [illegible]	Plonk, T.; Gough, J. E.; Brewer, K. L.	2009	Annals of Emergency Medicine	54	3	S49	NA	Plonk 2009	Irrelevant	Excluded by TI/AB review

S4 Table. All studies identified in the literature search

350	Impact of positive and negative beliefs about inhaled corticosteroids on adherence in inner- [illegible]	Ponieman, D.; Wisnivesky, J. P.; Leventhal, H.; Musumeci-Szab√≥, T. J.; Halm, E. A.	2009	Annals of Allergy, Asthma and Immunolog y	103	1	38-42	10.1016/S1 081-1206(10)60 141-X	Ponieman 2009	Irrelevant	Excluded by TI/AB review
351	The use of text messaging to improve asthma control: A study of short message service (SMS)	Prabhakaran, L.; Chee, J.; Kc, C.; Mun, W. W.	2009	Respirology	14	NA	A217	10.1111/j.1 440-1843.2009. 01658.x	Prabhakar an 2009	Irrelevant	Excluded by TI/AB review
352	IMPACT DC: Reconceptualizing the Role of the Emergency Department for Urban Children with Asthma	Quint, D. M.; Teach, S. J.	2009	Clinical Pediatric Emergency Medicine	10	2	115-121	10.1016/j.cp em.2009.03. 003	Quint 2009	Irrelevant	Excluded by TI/AB review
353	Association of Medical Home Care and Disparities in Emergency Care Utilization Among [illegible]	Raphael, J. L.; Zhang, Y. Q.; Liu, H.; Tapia, C. D.; Giardino, A. P.	2009	Academic Pediatrics	9	4	242-248	10.1016/j.ac ap.2009.05. 002	Raphael 2009	Irrelevant	Excluded by TI/AB review

S4 Table. All studies identified in the literature search

354	Handbook of peer interactions, relationships, and groups	Rubin, Kenneth H.; Bukowski, William M.; Laursen, Brett	2009	Social, emotional, and personality development in context	NA	NA	NA	NA	Rubin 2009	Irrelevant	Excluded by TI/AB review
355	Impact of Physician and Patient Gender on Pain Management in the Emergency Department-A Multicenter Study	Safdar, B.; Heins, A.; Homel, P.; Miner, J.; Neighbor, M.; DeSandre, P.; Todd, K. H.	2009	Pain Medicine	10	2	364-372	10.1111/j.1526-4637.2008.00524.x	Safdar 2009	Irrelevant	Excluded by TI/AB review
358	Significant barriers to kidney transplant evaluation and listing: A single-center analysis of 3000+ referrals	Schold, J. D.; Gregg, J. A.; Tomlin, D.; Patton, P.; Meier-Kriesche, H. U.	2009	American Journal of Transplantation	9	NA	352-353	NA	Schold 2009	Irrelevant	Excluded by TI/AB review
359	Health and black older adults: Insights from a life-course perspective	Sellers, Sherrill L.; Govia, Ishtar O.; Jackson, James S.; Braithwaite, Ronald L.; Taylor, Sandra E.; [illegible]	2009	Health issues in the Black community., 3rd ed.	NA	NA	95-116	NA	Sellers 2009	Irrelevant	Excluded by TI/AB review

S4 Table. All studies identified in the literature search

360	Use of emergency departments for conditions related to poor oral healthcare: implications for [illegible]	Shortridge, E. F.; Moore, J. R.	2009	Journal of public health management and practice : JPHMP	15	3	238-245	NA	Shortridge 2009	Irrelevant	Excluded by TI/AB review
362	Burden of chronic obstructive pulmonary disease in medicare beneficiaries residing in long-term care facilities	Simoni-Wastila, L.; Blanchette, C. M.; Qian, J.; Yang, H. w K.; Zhao, L.; Zuckerman, I. H.; Pak, G. H.; Silver, H.; Dalal, A. A.	2009	American Journal Geriatric Pharmacotherapy	7	5	262-270	10.1016/j.amjopharm.2009.11.003	Simoni-Wastila 2009	Irrelevant	Excluded by TI/AB review
363	Disproportionate use of psychiatric emergency services by African Americans	Snowden, L. R.; Catalano, R.; Shumway, M.	2009	Psychiatric Services	60	12	1664-1671	10.1176/appi.ps.60.12.1664	Snowden 2009	Irrelevant	Excluded by TI/AB review
364	Survey of physicians regarding clinical pharmacy services	Szczesiul, J. M.; Fairbanks, R. J.; Hildebrand, J. M.; Hays, D. P.; Shah,	2009	American Journal of Health-System	66	6	576-579	10.2146/ajhp080083	Szczesiul 2009	Irrelevant	Excluded by TI/AB review

S4 Table. All studies identified in the literature search

365	Computerized decision support to reduce potentially inappropriate prescribing to older emergency department patients: A randomized, controlled trial	Terrell, K. M.; Perkins, A. J.; Dexter, P. R.; Hui, S. L.; Callahan, C. M.; Miller, D. K.	2009	Journal of the American Geriatrics Society	57	8	1388-1394	10.1111/j.1532-5415.2009.02352.x	Terrell 2009	Irrelevant	Excluded by TI/AB review
366	Communication in end-stage cancer: review of the literature and future research	Trice, E. D.; Prigerson, H. G.; Trice, Elizabeth D.; Prigerson, Holly G.	2009	Journal of health communication	14	NA	95-108	10.1080/10810730902806786	Trice 2009	Irrelevant	Excluded by TI/AB review
367	HIV and Hepatitis C in patients with schizophrenia	Wainberg, Milton L.; Cournos, Francine; McKinnon, Karen; Berkman, Alan; Guimar√£es, Mark Drew Crosland; Meyer, Jonathan M.; Nasrallah, Henry A.	2009	Medical illness and schizophrenia., 2nd ed.	NA	NA	247-274	NA	Wainberg 2009	Irrelevant	Excluded by TI/AB review

S4 Table. All studies identified in the literature search

368	Learning how to measure the well-being of OVC in a maturing HIV/AIDS crisis	Wallis, A.; Dukay, V.	2009	J Health Care Poor Underserved	20	4 Suppl	170-84	10.1353/hpu.0.0230	Wallis 2009	Irrelevant	Excluded by TI/AB review
369	Liaison psychiatry with Aboriginal and Torres Strait Islander peoples	Wand, A. P. F.; Corr, M. J.; Eades, S. J.	2009	Australian and New Zealand Journal of Psychiatry	43	6	509-517	10.1080/00048670902873649	Wand 2009	Irrelevant	Excluded by TI/AB review
370	Effect of race on the safety and efficacy outcomes of gemcitabine plus paclitaxel treated patients with metastatic [illegible]	Wang, Y.; Tai, D.; Zhao, L.; Gill, J.; Obasaju, C. K.	2009	Journal of Clinical Oncology	27	NA	1070-1070	NA	Wang 2009	Irrelevant	Excluded by TI/AB review
371	Deciding to place a relative in long-term care: 'We really don't do that'	Winslow, Betty Wehtje; Flaskerud, Jacquelyn H.	2009	Issues in Mental Health Nursing	30	3	197-198	10.1080/01612840802694627	Winslow 2009	Irrelevant	Excluded by TI/AB review

S4 Table. All studies identified in the literature search

372	Revisiting predictors of parental health care-seeking behaviors for nonurgent conditions at one inner-city hospital	Zandieh, S. O.; Gershel, J. C.; Briggs, W. M.; Mancuso, C. A.; Kuder, J. M.	2009	Pediatric Emergency Care	25	4	238-243	NA	Zandieh 2009	Irrelevant	Excluded by TI/AB review
373	Factors predicting adherence with psychiatric follow-up appointments for patients assessed by the liaison psychiatric [illegible]	Agyapong, V. I. O.; Rogers, C.; MacHale, S.; Cotter, D.	2010	International Journal of Psychiatry in Medicine	40	2	217-228	10.2190/PM.40.2.g	Agyapong 2010	Irrelevant	Excluded by TI/AB review
374	Effectiveness of interventions targeting frequent users of emergency departments: A systematic review	Althaus, F.; Daeppen, J. B.; Paroz, S.; Hugli, O.; Peytremann-Bridevaux, I.; Ghali, W.; Bodenmann, P.	2010	Journal of General Internal Medicine	25	NA	S266-S267	10.1007/s11606-010-1338-5	Althaus 2010	Irrelevant	Excluded by TI/AB review

S4 Table. All studies identified in the literature search

375	Ethnic diversity and pathways to care for a first episode of psychosis in Ontario	Archie, S.; Akhtar-Danesh, N.; Norman, R.; Malla, A.; Roy, P.; Zipursky, R. B.	2010	Schizophr Bull	36	4	688-701	10.1093/schbul/sbn137	Archie 2010	Irrelevant	Excluded by TI/AB review
376	National profile of nonemergent pediatric emergency department visits	Ben-Isaac, E.; Schrager, S. M.; Keefer, M.; Chen, A. Y.	2010	Pediatrics	125	3	454-459	10.1542/peds.2009-0544	Ben-Isaac 2010	Irrelevant	Excluded by TI/AB review
377	Enhancing the provision of fertility-sparing treatment for ectopic pregnancies: A nursing administered Ectopic Pregnancy Program	Black, A. Y.; Singh, S. S.	2010	Journal of Minimally Invasive Gynecology	17	6	S145	NA	Black 2010	Irrelevant	Excluded by TI/AB review

S4 Table. All studies identified in the literature search

378	Intervention attendance among emergency department patients with alcohol-and drug-use disorders	Blow, F. C.; Walton, M. A.; Murray, R.; Cunningham, R. M.; Chermack, S. T.; Barry, K. L.; Ilgen, M. A.; Booth, B. M.	2010	Journal of Studies on Alcohol and Drugs	71	5	713-719	10.15288/jsad.2010.71.713	Blow 2010	Irrelevant	Excluded by TI/AB review
379	Assessment of disparities in the use of anxiolysis and sedation among children undergoing laceration repair	Brodzinski, H.; Iyer, S.; Grupp-Phelan, J.	2010	Academic Pediatrics	10	3	194-199	10.1016/j.acap.2009.12.003	Brodzinski 2010	Irrelevant	Excluded by TI/AB review
380	Analysis of risk factors and HIV knowledge of an adolescent cohort using emergency department (ED)-based multimedia [illegible]	Calderon, Y.; Cowan, E.; Chou, K.; Mathew, S.; Fettig, J.; Chin, R.; Rosenberg, M.; Leider, J.	2010	HIV Medicine	11	NA	69	10.1111/j.1468-1293.2010.00841-2.x	Calderon 2010	Irrelevant	Excluded by TI/AB review
381	Results from a multimedia testing and counseling program in an urban emergency department	Calderon, Y.; Cowan, E.; Fettig, J.; Egbuta, K.; Leider, J.	2010	Retrovirology	7	NA	P97	10.1186/1742-4690-7-S1-P97	Calderon 2010	Irrelevant	Excluded by TI/AB review

S4 Table. All studies identified in the literature search

382	Results from a multimedia testing and counselling program in an urban emergency department	Calderon, Y.; Cowan, E.; Fettig, J.; Egbuta, K.; Leider, J.	2010	HIV Medicine	11	NA	108	10.1111/j.1468-1293.2010.00841-2.x	Calderon 2010	Irrelevant	Excluded by TI/AB review
383	Treatment adherence among low-income, african american children with persistent asthma	Celano, M. P.; Linzer Sr, J. F.; Demi, A.; Bakeman, R.; Smith, C. O.; Croft, S.; Kobrynski, L. J.	2010	Journal of Asthma	47	3	317-322	10.3109/02770900903580850	Celano 2010	Irrelevant	Excluded by TI/AB review
384	Alcohol screeing and Brief Intervention in the emergency department settings: Evidence [illegible]	Clapp, J. D.; Woodruff, S. I.	2010	Alcoholism: Clinical and Experimental Research	34	6	287A	10.1111/j.1530-0277.2010.01211.x	Clapp 2010	Irrelevant	Excluded by TI/AB review

S4 Table. All studies identified in the literature search

385	An emergency department patient's perception of safety	Clark, Paul Richard	2010	NA	NA	Ph.D.	265 p-265 p	NA	Clark 2010	Irrelevant	Excluded by TI/AB review
386	Comparison of patient centeredness of visits to emergency departments, physicians, and dentists for dental problems and injuries	Cohen, L. A.; Bonito, A. J.; Eicheldinger, C.; Manski, R. J.; Macek, M. D.; Edwards, R. R.; Khanna, N.	2010	The Journal of the American College of Dentists	77	4	49-58	NA	Cohen 2010	Irrelevant	Excluded by TI/AB review
387	Bereavement outcomes associated with family witnessed resuscitations in an urban ED setting	Compton, S.; Griffin, M. J.; Waselewsky, D.; Mango, L.; Zalenski, R. J.	2010	Academic Emergency Medicine	17	NA	S66	10.1111/j.1553-2712.2010.00743.x	Compton 2010	Irrelevant	Excluded by TI/AB review

S4 Table. All studies identified in the literature search

388	Quality of life after robot assisted compared to open radical prostatectomy: Initial experience [illegible]	Cooperberg, M.; Sadetsky, N.; Sanda, M.; Carroll, P.	2010	Journal of Urology	183	4	e232-e233	10.1016/j.juro.2010.02.864	Cooperberg 2010	Irrelevant	Excluded by TI/AB review
389	Asthma and behavior in homeless 4- to 7-year-olds	Cutuli, J. J.; Herbers, J. E.; Rinaldi, M.; Masten, A. S.; Oberg, C. N.	2010	Pediatrics	125	1	145-151	10.1542/peds.2009-0103	Cutuli 2010	Irrelevant	Excluded by TI/AB review
390	Efficacy of brief interventions for hazardous and harmful drinkers in the emergency department	D'Onofrio, G.; Fiellin, D.; Pantalon, M.; Degutis, L. C.; Chawarski, M.; Owens, P. H.; Busch, S.; Bernstein, [illegible]	2010	Academic Emergency Medicine	17	NA	S75	10.1111/j.1553-2712.2010.00743.x	D'Onofrio 2010	Irrelevant	Excluded by TI/AB review
391	Brief intervention for hazardous and harmful drinkers in the ED	D'Onofrio, G.; Fiellin, D. A.; Pantalon, M. V.; Degutis, L. C.; Owens, P. H.; Bernstein, S. L.; Busch, S.; Chawarski, M. C.; O'Connor, P. G.	2010	Alcoholism: Clinical and Experimental Research	34	NA	81A	10.1111/j.1530-0277.2010.01292-3.x	D'Onofrio 2010	Irrelevant	Excluded by TI/AB review

S4 Table. All studies identified in the literature search

392	Population density and racial differences in the performance of emergency medical services	David, G.; Harrington, S. E.	2010	Journal of Health Economics	29	4	603-615	10.1016/j.jhealeco.2010.03.004	David 2010	Irrelevant	Excluded by TI/AB review
393	Parental knowledge and use of preventive asthma care measures in two pediatric	Deis, J. N.; Spiro, D. M.; Jenkins, C. A.; Buckles, T. L.; Arnold, D. H.	2010	Journal of Asthma	47	5	551-556	10.3109/02770900903560225	Deis 2010	Irrelevant	Excluded by TI/AB review
394	A U.S. survey of patients with Parkinson's disease: Satisfaction with medical care and [illegible]	Dorsey, E. R.; Voss, T. S.; Shprecher, D. R.; Deuel, L. M.; Beck, C. A.; Gardiner, I. F.; Coles, M. A.; Burns, [illegible]	2010	Movement Disorders	25	13	2128-2135	10.1002/mds.23160	Dorsey 2010	Irrelevant	Excluded by TI/AB review
395	Relationship of insurance status to hospital length of stay after traumatic	Englum, B. R.; Bolorundoro, O. B.; Villegas, C.; Stevens, K.; Haut, E.;	2010	Journal of Surgical Research	158	2	283	NA	Englum 2010	Irrelevant	Excluded by TI/AB review
396	Emergency department tachypnea predicts transfer to a higher level of care in the [illegible]	Farley, H.; Zubrow, M. T.; Gies, J.; Kolm, P.; Mascioli, S.; Mahoney, D. D.; Weintraub, W. S.	2010	Academic Emergency Medicine	17	7	718-722	10.1111/j.1553-2712.2010.00796.x	Farley 2010	Irrelevant	Excluded by TI/AB review

S4 Table. All studies identified in the literature search

397	Certification empowerment, and intent to leave current position and the profession among critical care nurses	Fitzpatrick, Joyce J.; Campo, Theresa M.; Graham, Gregory; Lavandero, Ram√≥n	2010	American Journal of Critical Care	19	3	218-226	10.4037/ajcc2010442	Fitzpatrick 2010	Irrelevant	Excluded by TI/AB review
398	"my child can't keep anything down!": Interviewing	Graham, J. M.; Fitzpatrick, E. A.; Black, K. J. L.	2010	Pediatric Emergency Care	26	4	251-256	10.1097/PEC.0b013e3181d6d9de	Graham 2010	Irrelevant	Excluded by TI/AB review
399	Obesity and Asthma Morbidity in a Community-Based Adult Cohort in a Large Urban Area: The Chicago Initiative to Raise Asthma Health Equity (CHIRAH)	Grammer, L. C.; Weiss, K. B.; Pedicano, J. B.; Kimmel, L. G.; Curtis, L. M.; Catrambone, C. D.; Lyttle, C. S.; Sharp, L. K.; Sadowski, L. S.	2010	Journal of Asthma	47	5	491-495	10.3109/02770901003801980	Grammer 2010	Irrelevant	Excluded by TI/AB review

S4 Table. All studies identified in the literature search

402	Health care resource utilization among adults with type 2 diabetes mellitus, hypertension, and [illegible]	Grandy, S.; Fox, K. M.	2010	Value in Health	13	3	A167	10.1016/S1098-3015(10)72810-2	Grandy 2010	Irrelevant	Excluded by TI/AB review
403	Handbook of African American health	Hampton, Robert L.; Gullotta, Thomas P.; Crowel, Raymond L.	2010	NA	NA	NA	NA	NA	Hampton 2010	Irrelevant	Excluded by TI/AB review
404	Mammography Promotion in the Emergency Department: A Pilot Study	Hatcher, J.; Rayens, M. K.; Schoenberg, N. E.	2010	Public Health Nursing	27	6	520-527	10.1111/j.1525-1446.2010.00894.x	Hatcher 2010	Irrelevant	Excluded by TI/AB review
405	Physician Race/Ethnicity Predicts Successful Emergency	Heins, A.; Homel, P.; Safdar, B.; Todd, K.	2010	Journal of Pain	11	7	692-697	10.1016/j.jpain.2009.10.017	Heins 2010	Irrelevant	Excluded by TI/AB review

S4 Table. All studies identified in the literature search

406	Patient preference for cardiac diagnostic testing	Hollander, J. E.; Mumma, B.; Baumann, B. M.; Diercks, D. B.; Takakuwa, K.; Shofer, F. S.	2010	Academic Emergency Medicine	17	NA	S138	10.1111/j.1553-2712.2010.00743.x	Hollander 2010	Irrelevant	Excluded by TI/AB review
407	The quality of emergency department pain care for older adult patients	Hwang, U.; Richardson, L. D.; Harris, B.; Morrison, R. S.	2010	Journal of the American Geriatrics Society	58	11	2122-2128	10.1111/j.1532-5415.2010.03152.x	Hwang 2010	Irrelevant	Excluded by TI/AB review
409	Blunt traumatic brain injuries in children-predictors of outcome	Islam, S.; Vick, L. R.; Schwannebeck, J.	2010	Journal of Surgical Research	158	2	275	NA	Islam 2010	Irrelevant	Excluded by TI/AB review

S4 Table. All studies identified in the literature search

410	Nurse and physician preferences for end-of-life care for trauma patients	Jacobs, L. M.; Burns, K. J.; Jacobs, B. B.	2010	Journal of Trauma - Injury, Infection and Critical	69	6	1567-1573	10.1097/TA.0b013e3181f65921	Jacobs 2010	Irrelevant	Excluded by TI/AB review
411	Self-reported discharge instruction adherence among different racial groups seen in the emergency [illegible]	James, T. D.; Smith, P. C.; Brice, J. H.	2010	Journal of the National Medical Association	102	10	931-936	10.1016/S0027-9684(15)30712-4	James 2010	Irrelevant	Excluded by TI/AB review
412	A review of barriers to thrombolytic therapy: Implications for nursing care in the	Johnson, M.; Bakas, T.	2010	Journal of Neuroscience Nursing	42	2	88-94	10.1097/JNN.0b013e3181ce5a48	Johnson 2010	Irrelevant	Excluded by TI/AB review
413	Consultation, referral and ethnicity: The role of primary care in accessing mental health services	Johnson, Marion; Weich, Scott	2010	Ethnicity and Inequalities in Health and Social Care	3	1	14-Jun	10.5042/eihsc.2010.0143	Johnson 2010	Irrelevant	Excluded by TI/AB review

S4 Table. All studies identified in the literature search

414	Case-Mix Adjustment and the Comparison of Community Health Center Performance on Patient Experience [illegible]	Johnson, M. L.; Rodriguez, H. P.; Solorio, M. R.	2010	Health Services Research	45	3	670-690	10.1111/j.1475-6773.2010.01101.x	Johnson 2010	Irrelevant	Excluded by TI/AB review
415	Feasibility of web-based asthma education for urban teenagers: Intervention compliance and computer access	Joseph, C. L.; Johnson, D.; Saltzgaber, J.; Havstad, S.	2010	American Journal of Respiratory and Critical Care Medicine	181	1	NA	NA	Joseph 2010	Irrelevant	Excluded by TI/AB review

S4 Table. All studies identified in the literature search

416	Perceived discrimination and use of health care services in a North Carolina population of Latino immigrants	Keller, S. C.; Silberberg, M.; Hartmann, K. E.; Michener, J. L.	2010	Hispanic Health Care International (Springer Publishing Company, Inc.)	8	1	13-Apr	10.1891/1540-4153.8.1.4	Keller 2010	Irrelevant	Excluded by TI/AB review
417	Understanding disparities in emergency department visits for asthma	Kimmins, Brandon M.	2010	NA	70	NA	4118-4118	NA	Kimmins 2010	Irrelevant	Excluded by TI/AB review
418	Pharmacogenetic considerations in the treatment of cancer pain	Klepstad, P√•l; Bruera, Eduardo D.; Portenoy, Russell K.	2010	Cancer pain: Assessment and management., 2nd ed.	NA	NA	180-194	NA	Klepstad 2010	Irrelevant	Excluded by TI/AB review

S4 Table. All studies identified in the literature search

419	Emergency department arrival times after transient ischemic attack	Kohake, S.; Khoury, J.; Alwell, K. A.; Moomaw, C. J.; Kissela, B. M.; Woo, D.; Adeoye, O.;	2010	Stroke	41	4	e344	10.1161/01.str.0000366115.56266.0a	Kohake 2010	Irrelevant	Excluded by TI/AB review
420	Emergency department reliance: a discriminatory measure of	Kroner, E. L.; Hoffmann, R. G.; Brousseau, D. C.	2010	Pediatrics	125	1	133-8	10.1542/peds.2009-0960	Kroner 2010	Irrelevant	Excluded by TI/AB review
421	Handbook of diversity in feminist psychology	Landrine, Hope; Russo, Nancy Felipe	2010	NA	NA	NA	NA	NA	Landrine 2010	Irrelevant	Excluded by TI/AB review
422	Mental health systems in a cross-cultural context	Lefley, Harriet P.; Scheid, Teresa L.; Brown, Tony N.	2010	A handbook for the study of mental health: Social contexts,	NA	NA	135-161	NA	Lefley 2010	Irrelevant	Excluded by TI/AB review

S4 Table. All studies identified in the literature search

423	Exploring beliefs of the four major ethnic groups in Melbourne regarding healthcare and treatment	Leong, K.; Weiland, T. J.; Dent, A. W.	2010	Aust Health Rev	34	4	458-66	10.1071/ah09782	Leong 2010	Irrelevant	Excluded by TI/AB review
426	Association of payer status with length of stay in patients hospitalized for injury in 2006 NHAMCS-ED [illegible]	Liu, J.; Shi, L.	2010	Value in Health	13	3	A87	10.1016/S1098-3015(10)72413-X	Liu 2010	Irrelevant	Excluded by TI/AB review
427	Differences in attitude towards cadaveric organ donation: Observations in a multiracial [illegible]	Loch, A.; Hilmi, I. N.; Mazam, Z.; Pillay, Y.; Choon, D. S. K.	2010	Hong Kong Journal of Emergency Medicine	17	3	236-243	10.1177/102490791001700306	Loch 2010	Irrelevant	Excluded by TI/AB review

S4 Table. All studies identified in the literature search

428	How benefits of expressive writing vary as a function of writing instructions, ethnicity and ambivalence over emotional [illegible]	Lu, Q.; Stanton, A. L.	2010	Psychology and Health	25	6	669-684	10.1080/08870440902883196	Lu 2010	Irrelevant	Excluded by TI/AB review
430	The relation between negative life events and asthma morbidity in inner city adolescents with asthma	Mantakos, S. M.; Eakin, M.; Rand, C. S.; Riekert, K. A.	2010	American Journal of Respiratory and Critical Care Medicine	181	1	NA	NA	Mantakos 2010	Irrelevant	Excluded by TI/AB review
431	Do hospitals provide lower quality of care to black patients for pneumonia?	Mayr, F. B.; Yende, S.; D'Angelo, G.; Barnato, A. E.; Kellum, J. A.; Weissfeld, L.; Yealy, [illegible]	2010	Critical Care Medicine	38	3	759-765	10.1097/CCM.0b013e3181c8fd58	Mayr 2010	Irrelevant	Excluded by TI/AB review
432	Telemedicine in urban and suburban childcare and elementary schools lightens family burdens	McConnochie, K. M.; Wood, N. E.; Herendeen, N. E.; ten Hoopen, C. B.; Roghmann, K. J.	2010	Telemedicine journal and e-health : the official journal of the American Telemedicine [illegible]	16	5	533-542	10.1089/tmj.2009.0138	McConnochie 2010	Irrelevant	Excluded by TI/AB review

S4 Table. All studies identified in the literature search

433	Investigating reasons for socioeconomic inequalities in breast cancer survival in New Zealand	McKenzie, F.; Ellison-Loschmann, L.; Jeffreys, M.	2010	Cancer Epidemiol	34	6	702-8	10.1016/j.canep.2010.07.007	McKenzie 2010	Irrelevant	Excluded by TI/AB review
434	Effects of hospitalists on 1-year postdischarge resource utilization by medicare beneficiaries	Meltzer, D.; Chung, J.	2010	Journal of Hospital Medicine	5	NA	50-51	10.1002/jhm.705	Meltzer 2010	Irrelevant	Excluded by TI/AB review

S4 Table. All studies identified in the literature search

435	The impact of crowding on time until abdominal CT interpretation in emergency department patients with acute abdominal pain	Mills, A. M.; Baumann, B. M.; Chen, E. H.; Zhang, K. Y.; Glaspey, L. J.; Hollander, J. E.; Pines, J. M.	2010	Postgraduate Medicine	122	1	75-81	10.3810/pgm.2010.01.2101	Mills 2010	Irrelevant	Excluded by TI/AB review
437	Chart Card: Feasibility of a Tool for Improving Emergency	Neumayr, L.; Pringle, S.; Giles, S.; Quirolo, K. C.; Paulukonis, S.; Vichinsky, E. P.; Treadwell, M. J.	2010	Journal of the National Medical Association	102	11	1017-1023	10.1016/S0027-9684(15)30728-8	Neumayr 2010	Irrelevant	Excluded by TI/AB review
438	Patient choice of provider type in the emergency	Padela, A. I.; Schneider, S. M.; He, H.; Ali, Z.;	2010	Emerg Med J	27	6	465-9	10.1136/emj.2008.070383	Padela 2010	Irrelevant	Excluded by TI/AB review

S4 Table. All studies identified in the literature search

441	A pilot study to examine research subjects' perception of participating in research in the emergency [illegible]	Paradis, C.; Phelan, M. P.; Brinich, M.	2010	Journal of Medical Ethics	36	10	580-587	10.1136/jme.2009.032904	Paradis 2010	Irrelevant	Excluded by TI/AB review
442	Housing as an intervention on hospital use: access among chronically homeless persons with disabilities	Parker, D.	2010	Journal of urban health : bulletin of the New York Academy of Medicine	87	6	912-919	NA	Parker 2010	Irrelevant	Excluded by TI/AB review
443	The association between hospital-level left-without-being seen (LWBS) rates and 90-day mortality [illegible]	Pines, J.; Baldassari, L.; Fischer, C.; Goyal, M.; Milzman, D.; Napoli, A. M.; Soelin, H.	2010	Academic Emergency Medicine	17	NA	S86	10.1111/j.1553-2712.2010.00743.x	Pines 2010	Irrelevant	Excluded by TI/AB review

S4 Table. All studies identified in the literature search

444	The effect of emergency department crowding on analgesia in patients with back [illegible]	Pines, J. M.; Shofer, F. S.; Isserman, J. A.; Abbuhl, S. B.; Mills, A. M.	2010	Academic Emergency Medicine	17	3	276-283	10.1111/j.1553-2712.2009.00676.x	Pines 2010	Irrelevant	Excluded by TI/AB review
445	Factors associated with healthcare visits by young children for nontoxic poisoning exposures	Polivka, B. J.; Casavant, M.; Baker, S. D.	2010	Journal of Community Health	35	6	572-578	NA	Polivka 2010	Irrelevant	Excluded by TI/AB review
446	Integrated systems to improve care for very high intensity users of hospital emergency [illegible]	Rea, H.; Kenealy, T.; Horwood, F.; Sheridan, N.; Parsons, M.; Wemekamp, B.; [illegible]	2010	The New Zealand medical journal	123	1320	76-85	NA	Rea 2010	Irrelevant	Excluded by TI/AB review
447	The impact of race and ethnicity on care in the pediatric emergency department	Riera, A.; Walker, D. M.	2010	Curr Opin Pediatr	22	3	284-9	10.1097/MOP.0b013e32833973a5	Riera 2010	Irrelevant	Excluded by TI/AB review

S4 Table. All studies identified in the literature search

451	Pain and psychological symptom differences in African Americans and European Americans [illegible]	Roane, B.; Soward, A.; Orrey, D.; Swor, R.; Liberzon, I.; Clauw, D.; McLean, S.	2010	Journal of Pain	11	4	S5	10.1016/j.jpain.2010.01.027	Roane 2010	Irrelevant	Excluded by TI/AB review
452	Adolescents' perceptions of interpersonal	Rutherford, K. A.; Pitetti, R. D.; Zuckerbraun, N. S.;	2010	Pediatric Emergency Care	26	4	257-273	10.1097/PEC.0b013e3181d6da09	Rutherford 2010	Irrelevant	Excluded by TI/AB review
454	Delivering quality care: The power of the patient narrative	Sallah, David	2010	Ethnicity and Inequalities in Health and Social Care	3	1	4-Feb	10.5042/eihsc.2010.0142	Sallah 2010	Irrelevant	Excluded by TI/AB review
455	A Comparison of Frequent and Infrequent Visitors to an Urban Emergency Department	Sandoval, E.; Smith, S.; Walter, J.; Schuman, S. A. H.; Olson, M. P.; Striefler, R.; Brown, S.; Hickner, J.	2010	Journal of Emergency Medicine	38	2	115-121	10.1016/j.jemermed.2007.09.042	Sandoval 2010	Irrelevant	Excluded by TI/AB review

S4 Table. All studies identified in the literature search

456	Provider type and patient satisfaction with procedural pain management in the ED	Scott, S.; Hoonpongsimanont, W.; Nagurka, R.; Lamba, S.; Cummins, M. A.; Rosania, A.; Compton, S.	2010	Academic Emergency Medicine	17	NA	S27	10.1111/j.1553-2712.2010.00743.x	Scott 2010	Irrelevant	Excluded by TI/AB review
457	Hospice, opiates, and acute care service use among the elderly before death from heart failure or cancer	Setoguchi, S.; Glynn, R. J.; Stedman, M.; Flavell, C. M.; Levin, R.; Stevenson, L. W.	2010	American Heart Journal	160	1	139-144	10.1016/j.ahj.2010.03.038	Setoguchi 2010	Irrelevant	Excluded by TI/AB review
458	Out-of-hospital cardiac arrest in South Asian and white populations in London: Database evaluation of [illegible]	Shah, A. S.; Bhopal, R.; Gadd, S.; Donohoe, R.	2010	Heart	96	1	27-29	10.1136/hrt.2009.170183	Shah 2010	Irrelevant	Excluded by TI/AB review

S4 Table. All studies identified in the literature search

460	Epiglottitis in the United States: National trends, variances, prognosis, and management	Shah, R. K.; Stocks, C.	2010	Laryngoscope	120	6	1256-1262	10.1002/lary.20921	Shah 2010	Irrelevant	Excluded by TI/AB review
461	Race, Ethnicity, and Pain among the US Adult Population	Shavers, V. L.; Bakos, A.; Sheppard, V. B.	2010	Journal of Health Care for the Poor and Underserved	21	1	177-220	10.1353/hpu.0.0255	Shavers 2010	Irrelevant	Excluded by TI/AB review
462	Effect of point-of-care computer reminders on physician behaviour: A systematic review	Shojania, K. G.; Jennings, A.; Mayhew, A.; Ramsay, C.; Eccles, M.; Grimshaw, J.	2010	CMAJ	182	5	E216-E225	10.1503/cmaj.090578	Shojania 2010	Irrelevant	Excluded by TI/AB review
463	Emergency department use by older adults in the last months of life	Smith, A.; McCarthy, E.; Cenzer, I. S.; Weber, E.; Covinsky, K.	2010	Journal of General Internal Medicine	25	NA	S268	10.1007/s11606-010-1338-5	Smith 2010	Irrelevant	Excluded by TI/AB review

S4 Table. All studies identified in the literature search

464	Emergency department use by older adults in the last months of life	Smith, A. K.; Stijacic Cenzer, I.; McCarthy, E.; Weber, E.; Covinsky, K.	2010	Journal of the American Geriatrics Society	58	NA	S2	10.1111/j.1532-5415.2010.02850.x	Smith 2010	Irrelevant	Excluded by TI/AB review
465	The role of ethnicity, sex, and language on delay to hospital arrival for acute ischemic stroke	Smith, M. A.; Lisabeth, L. D.; Bonikowski, F.; Morgenstern, L. B.; Smith, Melinda A.; Lisabeth, Lynda D.; Bonikowski, Frank; [illegible]	2010	Stroke (00392499)	41	5	905-909	10.1161/STROKEAHA.110.578112	Smith 2010	Irrelevant	Excluded by TI/AB review
466	Hallway patients reduce overall ED satisfaction scores	Stiffler, K. A.; Wilber, S. T.; Billings, N.	2010	Academic Emergency Medicine	17	NA	S88	10.1111/j.1553-2712.2010.00743.x	Stiffler 2010	Irrelevant	Excluded by TI/AB review
467	Knowledge and Attitudes of Emergency Department Patients Regarding Radiation Risk of CT: Effects of Age, Sex, Race, Education, Insurance, Body Mass Index, Pain, and Seriousness of [illegible]	Takakuwa, K. M.; Estepa, A. T.; Shofer, F. S.	2010	American Journal of Roentgenology	195	5	1151-1158	10.2214/AJR.09.3847	Takakuwa 2010	Irrelevant	Excluded by TI/AB review

S4 Table. All studies identified in the literature search

468	Bridge leadership: Connecting educational leadership and social justice to improve schools	Tooms, Autumn K.; Boske, Christa	2010	Educational leadership for social justice	NA	NA	NA	NA	Tooms 2010	Irrelevant	Excluded by TI/AB review
469	North Carolina Emergency Department data: January 1, 2007-December 31, 2007	Waller, A.; Hakenewerth, A.; Tintinalli, J.; Ising, A.	2010	North Carolina medical journal	71	1	15-25	NA	Waller 2010	Irrelevant	Excluded by TI/AB review
470	Effects of a brief intervention for reducing violence and alcohol misuse	Walton, M. A.; Chermack, S. T.; Shope, J. T.; Bingham, C. R.;	2010	JAMA - Journal of the American	304	5	527-535	10.1001/jama.2010.1066	Walton 2010	Irrelevant	Excluded by TI/AB review
471	Level of pain and waiting time in the emergency department	Wheeler, E.; Hardie, T.; Klemm, P.; Akanji, I.; Schonewolf, E.; Scott, J.; Sterling, B.	2010	Pain Manag Nurs	11	2	108-14	10.1016/j.pmn.2009.06.005	Wheeler 2010	Irrelevant	Excluded by TI/AB review
476	Marital status and mental health	Williams, Kristi; Frech, Adrianne; Carlson, Daniel L.; Scheid, Teresa L.; Brown, Tony N.	2010	A handbook for the study of mental health:	NA	NA	306-320	NA	Williams 2010	Irrelevant	Excluded by TI/AB review

S4 Table. All studies identified in the literature search

477	Quality improvement evaluation of community asthma initiative: One year follow-up, [illegible]	Woods, E.; Bhaumik, U.; Nethersole, S.; Sommer, S.; Wilkinson, R.; Chan, E.; Burack, A.; Mannix, L.; Sesma, [illegible]	2010	Journal of Adolescent Health	46	2	S9	10.1016/j.jadohealth.2009.11.022	Woods 2010	Irrelevant	Excluded by TI/AB review
478	Medication Adherence is a Mediator of the Relationship Between¬†Ethnicity and Event-Free [illegible]	Wu, J. R.; Lennie, T. A.; De Jong, M. J.; Frazier, S. K.; Heo, S.; Chung, M. L.; Moser, D. K.	2010	Journal of Cardiac Failure	16	2	142-149	10.1016/j.cardfail.2009.10.017	Wu 2010	Irrelevant	Excluded by TI/AB review
481	Clinician personality and the evaluation of higher-risk patient symptoms	Allen, A. S.; Orav, E. J.; Lee, T. H.; Sequist, T. D.	2011	Journal of patient safety	7	3	122-126	NA	Allen 2011	Irrelevant	Excluded by TI/AB review
482	Effectiveness of interventions targeting frequent users of emergency departments: A systematic review	Althaus, F.; Paroz, S.; Hugli, O.; Ghali, W. A.; Daeppen, J. B.; Peytremann-Bridevaux, I.; Bodenmann, P.	2011	Annals of Emergency Medicine	58	1	41-52	10.1016/j.annemergmed.2011.03.007	Althaus 2011	Irrelevant	Excluded by TI/AB review

S4 Table. All studies identified in the literature search

483	Emergency department-initiated tobacco dependence treatment	Anders, M. E.; Sheffer, C. E.; Barone, C. P.; Holmes, T. M.; Simpson, D. D.; Duncan, A. M.	2011	American Journal of Health Behavior	35	5	546-556	10.5993/AJHB.35.5.4	Anders 2011	Irrelevant	Excluded by TI/AB review
484	Problem solving to improve adherence and asthma outcomes in urban adults with moderate or severe asthma: A randomized [illegible]	Apter, A. J.; Wang, X.; Bogen, D. K.; Rand, C. S.; McElligott, S.; Polsky, D.; Gonzalez, R.; Priolo, C.; Adam, B.; Geer, S.; Ten Have, T.	2011	Journal of Allergy and Clinical Immunology	128	3	516-523.e5	10.1016/j.jaci.2011.05.010	Apter 2011	Irrelevant	Excluded by TI/AB review
485	Demographics and risk factors of patients who decline opt-in hiv screenings in an urban emergency [illegible]	Arfai, N.; Squires, K.; Ezeala, Y.; Carolfi, L.; Moon, J.; Lopez, B.; Brigham, J.	2011	Annals of Emergency Medicine	58	4	S200	10.1016/j.annemergmed.2011.06.094	Arfai 2011	Irrelevant	Excluded by TI/AB review
486	The relevance of WHO injury surveillance guidelines for evaluation: learning from the aboriginal [illegible]	Auer, A. M.; Dobmeier, T. M.; Haglund, B. J.; Tillgren, P.	2011	BMC Public Health	11	NA	744	10.1186/1471-2458-11-744	Auer 2011	Irrelevant	Excluded by TI/AB review

S4 Table. All studies identified in the literature search

487	Examining effectiveness of medical interpreters in emergency departments for Spanish-speaking patients with [illegible]	Bagchi, A. D.; Dale, S.; Verbitsky-Savitz, N.; Andrecheck, S.; Zavotsky, K.; Eisenstein, R.	2011	Annals of Emergency Medicine	57	3	248-256.e1-e4	10.1016/j.annemergmed.2010.05.032	Bagchi 2011	Irrelevant	Excluded by TI/AB review
488	Pediatric emergency medicine performance measures: Variation in [illegible]	Bajaj, L.; Brousseau, D. C.; Lichenstein, R.; Brown, K. M.; Schuur, J.; Alpern, E. R.; Gorelick, M.; Chamberlain, J. M.; [illegible]	2011	Pediatric Emergency Care	27	10	1003	NA	Bajaj 2011	Irrelevant	Excluded by TI/AB review
489	Socioeconomic status, health care use, and outcomes: persistence of disparities over time	Begley, C.; Basu, R.; Lairson, D.; Reynolds, T.; Dubinsky, S.; Newmark, M.; Barnwell, F.; Hauser, A.; Hesdorffer, D.	2011	Epilepsia	52	5	957-64	10.1111/j.1528-1167.2010.02968.x	Begley 2011	Irrelevant	Excluded by TI/AB review

S4 Table. All studies identified in the literature search

491	Low health literacy and health outcomes: an updated systematic review	Berkman, N. D.; Sheridan, S. L.; Donahue, K. E.; Halpern, D. J.; Crotty, K.	2011	Ann Intern Med	155	2	97-107	10.7326/0003-4819-155-2-201107190-00005	Berkman 2011	Irrelevant	Excluded by TI/AB review
494	Patients with sickle cell disease have increased sensitivity to cold and heat stimuli, 2	Brandow, A. M.; Kattappuram, R.; Stucky, C. L.; Hillery, C. A.; Panepinto, J. A.	2011	Blood	118	21	NA	NA	Brandow 2011	Irrelevant	Excluded by TI/AB review
495	Exploring heart failure-related emergency department visits and hospitalizations from the patient perspective	Briggs-Malonson, M.	2011	Annals of Emergency Medicine	58	4	S224-S225	10.1016/j.annemergmed.2011.06.167	Briggs-Malonson 2011	Irrelevant	Excluded by TI/AB review

S4 Table. All studies identified in the literature search

496	Effects of a school-based intervention for urban adolescents with asthma a controlled trial	Bruzzese, J. M.; Sheares, B. J.; Vincent, E. J.; Du, Y.; Sadeghi, H.; Levison, M. J.; Mellins, R. B.; Evans, D.	2011	American Journal of Respiratory and Critical Care Medicine	183	8	998-1006	10.1164/rccm.201003-0429OC	Bruzzese 2011	Irrelevant	Excluded by TI/AB review
497	Apha-asp project chance: A student pharmacist led initiative to increase medication [illegible]	Byrns, J.; Gates, A.; Helmlinger, K.	2011	Journal of the American Pharmacists Association	51	2	256	10.1331/JAPhA.2011.11517	Byrns 2011	Irrelevant	Excluded by TI/AB review
498	The phenomenon of Obama and the agenda for education: Can	Carr, Paul R.; Porfilio, Brad J.	2011	Critical constructions: Studies on	NA	NA	NA	NA	Carr 2011	Irrelevant	Excluded by TI/AB review

S4 Table. All studies identified in the literature search

499	Does late night hip surgery affect outcome?	Chacko, A. T.; Ramirez, M. A.; Ramappa, A. J.; Richardson, L. C.; Appleton, P. T.; Rodriguez, E. K.	2011	Journal of Trauma	71	2	447-453	10.1097/TA.0b013e3182231ad7	Chacko 2011	Irrelevant	Excluded by TI/AB review
500	Preferences for technology-based interventions among adult emergency department patients with drug [illegible]	Choo, E. K.; Ranney, M. L.; Wong, Z.; Mello, M. J.	2011	Annals of Emergency Medicine	58	4	S198	10.1016/j.annemergmed.2011.06.089	Choo 2011	Irrelevant	Excluded by TI/AB review
501	A comparative evaluation of the process of developing and implementing an emergency [illegible]	Christopoulos, K. A.; Koester, K.; Weiser, S.; Lane, T.; Myers, J. J.; Morin, S. F.	2011	Implementation science : IS	6	NA	30	NA	Christopoulos 2011	Irrelevant	Excluded by TI/AB review
502	Deciding to use the emergency room: A qualitative survey of older veterans	Claver, Maria L.	2011	Journal of Gerontological Social Work	54	3	292-308	10.1080/01634372.2011.552938	Claver 2011	Irrelevant	Excluded by TI/AB review

S4 Table. All studies identified in the literature search

503	Comparison of patient visits to emergency departments, physician offices, and dental offices [illegible]	Cohen, L. A.; Bonito, A. J.; Eicheldinger, C.; Manski, R. J.; MacEk, M. D.; Edwards, R. R.; Khanna, N.	2011	Journal of Public Health Dentistry	71	1	13-22	10.1111/j.1752-7325.2010.00195.x	Cohen 2011	Irrelevant	Excluded by TI/AB review
504	Behavioral and socioeconomic correlates of dental problem experience and patterns of health care-seeking	Cohen, L. A.; Bonito, A. J.; Eicheldinger, C.; Manski, R. J.; Macek, M. D.; Edwards, R. R.; Khanna, N.	2011	Journal of the American Dental Association	142	2	137-149	10.14219/jada.archive.2011.0056	Cohen 2011	Irrelevant	Excluded by TI/AB review
505	Family-witnessed resuscitation: Bereavement	Compton, S.; Levy, P.; Griffin, M.; Waselewsky, D.;	2011	Journal of Palliative Medicine	14	6	715-721	10.1089/jpm.2010.0463	Compton 2011	Irrelevant	Excluded by TI/AB review
508	Racial-ethnic disparities in stroke care: the American	Cruz-Flores, S.; Rabinstein, A.; Biller, J.; Elkind, M. S.; Griffith, P.; Gorelick,	2011	Stroke (00392499)	42	7	2091-2116	10.1161/STR.0b013e3182213e24	Cruz-Flores 2011	Irrelevant	Excluded by TI/AB review

S4 Table. All studies identified in the literature search

511	Attitudes of cost and related barriers to prescription medications	Dalawari, P.; Patel, N. M.; Bzdawka, W.; Petrone, J. S.; Liou, V. D.	2011	Annals of Emergency Medicine	58	4	S280	10.1016/j.annemergmed.2011.06.336	Dalawari 2011	Irrelevant	Excluded by TI/AB review
512	The quality of emergency medical services	David, G.; Harrington, S. E.	2011	LDI Issue Brief	17	3	4-Jan	NA	David 2011	Irrelevant	Excluded by TI/AB review
513	Ethnic differences in reported pain regions - Results of a survey at three	David, M.; Pant, H. A.; Oligm√ºller, A. K.; Babitsch, B.; Borde, T.	2011	Geburtshilfe und Frauenheilkunde	71	1	44-52	10.1055/s-0030-1250722	David 2011	Irrelevant	Excluded by TI/AB review

S4 Table. All studies identified in the literature search

515	Acute Coronary Syndrome: Factors Affecting Time to Arrival in a Diverse Urban Setting	Deshmukh, Mrualini; Joseph, Michael; Verdecias, Niko; Malka, Edmond; LaRosa, Judith	2011	Journal of Community Health	36	6	895-902	10.1007/s10900-011-9465-4	Deshmukh 2011	Irrelevant	Excluded by TI/AB review
517	Patient satisfaction with rapid HIV testing in the emergency department	Donnell-Fink, L.; Reichmann, W. M.; Arbelaez, C.; Case, A. L.; Katz, J. N.; Losina, E.; Walensky, R. P.	2011	Annals of Emergency Medicine	58	1 SUPPL.	S49-S52	10.1016/j.annemergmed.2011.03.024	Donnell-Fink 2011	Irrelevant	Excluded by TI/AB review
518	A prospective 2-year study of emergency department patients with early-phase primary psychosis or substance-induced psychosis	Drake, R. E.; Caton, C. L. M.; Xie, H.; Hsu, E.; Gorroochurn, P.; Samet, S.; Hasin, D. S.	2011	American Journal of Psychiatry	168	7	742-748	10.1176/appi.ajp.2011.10071051	Drake 2011	Irrelevant	Excluded by TI/AB review
519	Disparities in asthma care and outcome	Emmett, Gary A.; Rothkopf, Amy C.; Merrick, Joav	2011	Child and adolescent health yearbook, 2009.	NA	NA	17-23	NA	Emmett 2011	Irrelevant	Excluded by TI/AB review

S4 Table. All studies identified in the literature search

520	Awareness of heart attack symptoms among US adults in 2007,	Fang, J.; Gillespie, C.; Keenan, N. L.; Greenlund, K. J.	2011	Future Cardiology	7	3	311-320	10.2217/fca.10.111	Fang 2011	Irrelevant	Excluded by TI/AB review
521	Retrospective review of poison control center cases involving cocaine exposures in children, 1997-2010	Fleurat, M.; Armenian, P.; Olson, K. R.	2011	Clinical Toxicology	49	6	551-552	10.3109/15563650.2011.598695	Fleurat 2011	Irrelevant	Excluded by TI/AB review
522	Review of Racism and discourse in Latin America	Floyd, Simeon	2011	Language in Society	40	5	670-671	10.1017/S0047404511000807	Floyd 2011	Irrelevant	Excluded by TI/AB review
523	Prevalence and sociodemographic correlates of mental health problems among emergency [illegible]	Funn, J.; Woodruff, S. I.	2011	Journal of Dual Diagnosis	7	2-Jan	14-25	10.1080/15504263.2011.569192	Funn 2011	Irrelevant	Excluded by TI/AB review
524	Comparison of substance abuse treatment utilization and preferences among [illegible]	Goebert, D.; Nishimura, S.	2011	Journal of Substance Use	16	2	161-170	10.3109/14659891.2011.554594	Goebert 2011	Irrelevant	Excluded by TI/AB review

S4 Table. All studies identified in the literature search

525	Non-symptom-related factors contributing to delay in seeking medical care by patients with heart failure: A narrative review	Gravely, S.; Tamim, H.; Smith, J.; Daly, T.; Grace, S. L.	2011	Journal of Cardiac Failure	17	9	779-787	10.1016/j.cardfail.2011.05.003	Gravely 2011	Irrelevant	Excluded by TI/AB review
526	‚ÄúI want to be taking my own last breath,Äù: Patients' reflections on illness when presenting to an emergency department at the end of life (723)	Grudzen, C.; Stone, S.; Mohanty, S.; Lorenz, K. A.; Torres, J.; Ortiz, J.; Timmermans, S.	2011	Journal of Pain and Symptom Management	41	1	285-286	10.1016/j.jpainsymman.2010.10.202	Grudzen 2011	Irrelevant	Excluded by TI/AB review
527	A comparison of patient and staff attitudes about emergency department-based HIV testing in 2 urban hospitals	Hecht, C. R.; Smith, M. D.; Radonich, K.; Kozlovskaya, O.; Totten, V. Y.	2011	Annals of Emergency Medicine	58	1 SUPPL.	S28-S32	10.1016/j.annemergmed.2011.03.020	Hecht 2011	Irrelevant	Excluded by TI/AB review

S4 Table. All studies identified in the literature search

528	A prospective, single-center study to evaluate the use of skin adhesive in the closure of scalp wounds	Hendry, P. L.; Kalynych, C. J.; Webb, L.; Westenbarger, R.; Lissoway, J.; Kumar, V.	2011	Annals of Emergency Medicine	58	4	S288	10.1016/j.annemergmed.2011.06.359	Hendry 2011	Irrelevant	Excluded by TI/AB review
529	Trust vs. opportunity - Impact of age, race, and sex on patient participation in research in emergency [illegible]	Holland, C. K.; Hart, K. W.; McTaggart, C. J.; Lindsell, C. J.	2011	Academic Emergency Medicine	18	5	S219-S220	10.1111/j.1553-2712.2011.01073.x	Holland 2011	Irrelevant	Excluded by TI/AB review
530	Predictors of influenza vaccination among emergency medical services personnel	Hubble, Michael W.; Zontek, Tracy L.; Richards, Michael E.	2011	Prehospital Emergency Care	15	2	175-183	10.3109/10903127.2010.541982	Hubble 2011	Irrelevant	Excluded by TI/AB review

S4 Table. All studies identified in the literature search

531	The effect of patient health literacy on preventable hospitalizations from the emergency department	Jackman, K. M.; Kanthala, A. R.; Bennett, D. L.; Hall, A. G.; Carden, D. L.	2011	Academic Emergency Medicine	18	5	S70	10.1111/j.1553-2712.2011.01073.x	Jackman 2011	Irrelevant	Excluded by TI/AB review
532	Racial Differences in Location Before Hospice Enrollment and Association with Hospice Length of [illegible]	Johnson, Kimberly S.; Kuchibhatla, Maragatha; Tulsky, James A.	2011	Journal of the American Geriatrics Society	59	4	732-737	10.1111/j.1532-5415.2011.03326.x	Johnson 2011	Irrelevant	Excluded by TI/AB review
533	Race/ethnicity-related and payer-related disparities in the timeliness of emergency care in U.S. emergency [illegible]	Johnston, V.; Bao, Y.	2011	J Health Care Poor Underserved	22	2	606-20	10.1353/hpu.2011.0050	Johnston 2011	Irrelevant	Excluded by TI/AB review
534	Sickle cell day hospital at memorial regional hospital - A solution that works	Jordan, L.; Etienne, L.; Brownlee, J.; Venkataramani, M.; Pierre-Louis, R. L.; Whiteman, L.; McFarland, S. A.	2011	American Journal of Hematology	86	10	E19-E21	10.1002/ajh.22152	Jordan 2011	Irrelevant	Excluded by TI/AB review

S4 Table. All studies identified in the literature search

535	The impact of the medicare doughnut hole on medication use behaviors and	Joshi, N.; Banahan, B. F. I.	2011	Value in Health	14	3	A43	NA	Joshi 2011	Irrelevant	Excluded by TI/AB review
536	Which patients leave the emergency department without being seen	Kamali, M.; Jain, M.; Jain, A. R.; Schneider, S. M.	2011	Academic Emergency Medicine	18	5	S68	10.1111/j.1553-2712.2011.01073.x	Kamali 2011	Irrelevant	Excluded by TI/AB review
537	How comfortable are patients with different triage personnel, and	Kamali, M. F.; Jain, A.; Jain, M.; Schneider, S. M.	2011	Annals of Emergency Medicine	58	4	S235	10.1016/j.annemergmed.2011.06.201	Kamali 2011	Irrelevant	Excluded by TI/AB review
538	Duration of patients visits to the emergency department	Karaca, Z.; Wong, H.; Mutter, R.	2011	Value in Health	14	3	A19	NA	Karaca 2011	Irrelevant	Excluded by TI/AB review
539	Characteristics of homeless individuals using inpatient and emergency department services	Karaca, Z.; Wong, H.; Mutter, R.	2011	Value in Health	14	3	A25	NA	Karaca 2011	Irrelevant	Excluded by TI/AB review

S4 Table. All studies identified in the literature search

540	Door-to-imaging times in patients presenting with acute stroke	Kelly, A. G.; Hellkamp, A. S.; Olson, D. W.; Smith, E. E.; Schwamm, L. H.	2011	Circulation: Cardiovascular Quality and Outcomes	4	6	NA	NA	Kelly 2011	Irrelevant	Excluded by TI/AB review
541	The role of cultural factors and sexual education on etiology of vaginismus	Kilic¬∏, A.; Bakil c¬∏i, A.	2011	Journal of Sexual Medicine	8	NA	285	NA	Kilic¬∏ 2011	Irrelevant	Excluded by TI/AB review
542	Family-based and cultural protective factors in urban children with asthma	Koinis-Mitchell, D.; McQuaid, E.; Kopel, S.; Potter, C.; Salcedo, L.; York, D.	2011	American Journal of Respiratory and Critical Care Medicine	183	1	NA	NA	Koinis-Mitchell 2011	Irrelevant	Excluded by TI/AB review
543	Factors associated with histologic versus cytologic diagnosis of lung cancer	Lathan, C. S.; Cronin, A.; Weeks, J. C.	2011	Journal of Clinical Oncology	29	15	NA	NA	Lathan 2011	Irrelevant	Excluded by TI/AB review

S4 Table. All studies identified in the literature search

544	Irinotecan compared to etoposide in combination with platinum analog in extensive disease small cell lung cancer: Systematic [illegible]	Lima, J. P. S. N.; Dos Santos, L. V.; Sasse, E. C.; Sasse, A. D.	2011	Journal of Thoracic Oncology	6	6	S313-S314	NA	Lima 2011	Irrelevant	Excluded by TI/AB review
545	Promoting best-care practices in childhood asthma: Quality improvement in community health centers	Lob, S. H.; Boer, J. H.; Porter, P. G.; N√∫√±ez, D.; Fox, P.	2011	Pediatrics	128	1	20-28	10.1542/peds.2010-1962	Lob 2011	Irrelevant	Excluded by TI/AB review
546	Optimizing patient/caregiver satisfaction through quality of communication in the pediatric emergency [illegible]	Locke, R.; Stefano, M.; Koster, A.; Taylor, B.; Greenspan, J.	2011	Pediatric Emergency Care	27	11	1016-1021	10.1097/PEC.0b013e318235be06	Locke 2011	Irrelevant	Excluded by TI/AB review
547	Routine, rapid HIV testing of medicine service admissions in the emergency department	Lubelchek, R. J.; Kroc, K. A.; Levine, D. L.; Beavis, K. G.; Roberts, R. R.	2011	Annals of Emergency Medicine	58	1 SUPPL.	S65-S70	10.1016/j.annemergmed.2011.03.027	Lubelchek 2011	Irrelevant	Excluded by TI/AB review

S4 Table. All studies identified in the literature search

548	Developing cross-cultural competence: A guide for working with children and their families, 4th ed	Lynch, Eleanor W.; Hanson, Marci J.	2011	NA	NA	NA	NA	NA	Lynch 2011	Irrelevant	Excluded by TI/AB review
549	Time to pain medication in children with long bone fractures presenting to [illegible]	Madhok, M.; Liu, M.	2011	Regional Anesthesia and Pain Medicine	36	5	E244-E245	NA	Madhok 2011	Irrelevant	Excluded by TI/AB review
550	Racial disparity in analgesic treatment for ED patients with abdominal or back pain	Mills, A. M.; Shofer, F. S.; Boulis, A. K.; Holena, D. N.; Abbuhl, S. B.	2011	American Journal of Emergency Medicine	29	7	752-756	10.1016/j.ajem.2010.02.023	Mills 2011	Irrelevant	Excluded by TI/AB review

S4 Table. All studies identified in the literature search

551	Impact of sex and ethnicity of patient and physician provider on pain medication usage in emergency department [illegible]	Milzman, D. P.; Dirkes, B.; Vieth, J.; Napoli, A.	2011	Annals of Emergency Medicine	58	4	S254	10.1016/j.annemergmed.2011.06.258	Milzman 2011	Irrelevant	Excluded by TI/AB review
552	Quality of life assessment after completion of a community based inner city latino asthma program: The children's respira education program	Montalvo Stanton, E.; Suarez, B.; Martinez, E.; Compton, S.	2011	American Journal of Respiratory and Critical Care Medicine	183	1	NA	NA	Montalvo Stanton 2011	Irrelevant	Excluded by TI/AB review
553	Do health beliefs influence the likelihood of patient compliance in prospective, longitudinal studies? Insight [illegible]	Muchayi, T.; Levy, P. D.; Brody, A.; Mahn, J.; Marinica, A.; Carroll, J.; Nasser, S.; Hedquist, L.; Zhang, S.; Flack, J. M.	2011	Academic Emergency Medicine	18	5	S64-S65	10.1111/j.1553-2712.2011.01073.x	Muchayi 2011	Irrelevant	Excluded by TI/AB review
554	Consumer-Directed Personal Care: Comparing Aged and Non-	Newcomer, R.; Kang, T.; Faucett, J.	2011	Home Health Care Services Quarterly	30	4	178-197	10.1080/01621424.2011.622245	Newcomer 2011	Irrelevant	Excluded by TI/AB review

S4 Table. All studies identified in the literature search

556	Clinical significance of severe gastric retention in patients with gastroparesis	Nguyen, L. A. B.; Wang, J.; Wilson, L.; Farrugia, G.; Parkman, H. P.; Koch, K. L.; Snape, W. J.; Hasler, W. L.; Abell, T. L.; [illegible]	2011	Gastroenterology	140	5	S807	10.1016/S0016-5085(11)63345-3	Nguyen 2011	Irrelevant	Excluded by TI/AB review
557	Supporting mcrn research through improving clinical trial delivery by hospital [illegible]	Norton, C.; Sinclair, A.; Marriott, J. F.	2011	Archives of Disease in Childhood	96	4	NA	10.1136/adc.2011.211243.11	Norton 2011	Irrelevant	Excluded by TI/AB review
558	SMART tags: A surrogate standard for mass casualty triage in Australia	Norton, I.	2011	EMA - Emergency Medicine Australasia	23	NA	5	10.1111/j.1742-6723.2011.01364.x	Norton 2011	Irrelevant	Excluded by TI/AB review

S4 Table. All studies identified in the literature search

559	Building care systems to improve access for high-risk and vulnerable veteran populations	O'Toole, T. P.; Pirraglia, P. A.; Dosa, D.; Bourgault, C.; Redihan, S.; O'Toole, M. B.; Blumen, J.; Primary Care-Special [illegible]	2011	Journal of General Internal Medicine	26 Suppl 2	NA	683-688	10.1007/s11606-011-1818-2	O'Toole 2011	Irrelevant	Excluded by TI/AB review
560	Addressing health disparities for minority patients with asthma: A systematic review	Press, V. G.; Pincavage, A. T.; Pappalardo, A. A.; Conwell, W. D.; Prochaska, M. H.; Alexander-Young, M.; Arora, V. M.	2011	Journal of General Internal Medicine	26	NA	S213	10.1007/s11606-011-1730-9	Press 2011	Irrelevant	Excluded by TI/AB review
561	Emergency department patient preferences for technology-based behavioral interventions	Ranney, M.; Choo, E.; Baum, A.; Wang, Y.; Clark, M.; Mello, M. J.	2011	Academic Emergency Medicine	18	5	S102-S103	10.1111/j.1553-2712.2011.01073.x	Ranney 2011	Irrelevant	Excluded by TI/AB review

S4 Table. All studies identified in the literature search

562	Association of incarceration with trauma, substance use related health consequences, and health care utilization	Redmond, N.; Hicks, L. S.; Cheng, D. M.; Allensworth-Davies, D.; Winter, M. R.; Samet, J. H.; Saitz, R.	2011	Journal of General Internal Medicine	26	NA	S160	10.1007/s11606-011-1730-9	Redmond 2011	Irrelevant	Excluded by TI/AB review
563	Inappropriate pediatric emergency medical services utilization redefined	Richards, M. E.; Hubble, M. W.; Zwehl-Burke, S.	2011	Pediatric Emergency Care	27	6	514-518	10.1097/PEC.0b013e31821c98bf	Richards 2011	Irrelevant	Excluded by TI/AB review

S4 Table. All studies identified in the literature search

564	The development of a motivational interviewing intervention to promote medication adherence among [illegible]	Riekert, K. A.; Borrelli, B.; Bilderback, A.; Rand, C. S.	2011	Patient Education and Counseling	82	1	117-122	10.1016/j.pec.2010.03.005	Riekert 2011	Irrelevant	Excluded by TI/AB review
566	Ethnic differences in responses to pain management	Russell, I. Jon	2011	Journal of Musculoskeletal Pain	19	1	7-May	10.3109/10582452.2010.544444	Russell 2011	Irrelevant	Excluded by TI/AB review
567	Patient insight into the health risks associated with obesity	Ryan, M.; Kanthala, A.; Cantrell, A.	2011	Annals of Emergency Medicine	58	4	S224	10.1016/j.annemergmed.2011.06.165	Ryan 2011	Irrelevant	Excluded by TI/AB review
568	Quality of non-small cell lung cancer (NSCLC) care in the Veterans Health [illegible]	Ryoo, J. J.; Kim, B.; Ordin, D. L.; Oishi, S. M.; Asch, S. M.; Antonio, A. L. M.; He, R.; Gould, M. [illegible]	2011	Journal of Clinical Oncology	29	15	NA	NA	Ryoo 2011	Irrelevant	Excluded by TI/AB review

S4 Table. All studies identified in the literature search

569	Predictors of transport by private vehicle versus ambulance to the emergency department in patients with st-elevation myocardial infarction and impact on door-to- [illegible]	Scherer, T. M.; Russ, S.; Jenkins, C. A.; Jones, I. D.; Cunningham, B. L.; Barrett, T. W.	2011	Annals of Emergency Medicine	58	4	S302-S303	10.1016/j.annemergmed.2011.06.402	Scherer 2011	Irrelevant	Excluded by TI/AB review
570	Effects of a firm model innovation on resident and patient satisfaction	Schmit, J.; Lo, M. C.; Vega, A.	2011	Journal of General Internal Medicine	26	NA	S575	10.1007/s11606-011-1730-9	Schmit 2011	Irrelevant	Excluded by TI/AB review
571	Predictors of nonadherence to national hospital quality measures for heart failure [illegible]	Shahian, D. M.; Nordberg, P.; Meyer, G. S.; Mort, E.; Atamian, S.; Liu, X.; Karson, A. S.; [illegible]	2011	American Journal of Medicine	124	7	636-646	10.1016/j.amjmed.2011.03.021	Shahian 2011	Irrelevant	Excluded by TI/AB review
573	Emergency department crowding and analgesic delay in pediatric sickle cell pain crises	Shenoi, R.; Ma, L.; Syblik, D.; Yusuf, S.	2011	Pediatric Emergency Care	27	10	911-917	10.1097/PEC.0b013e3182302871	Shenoi 2011	Irrelevant	Excluded by TI/AB review

S4 Table. All studies identified in the literature search

574	Attempts to reach the oldest and frailest: recruitment, adherence, and retention of urban [illegible]	Stineman, M. G.; Strumpf, N.; Kurichi, J. E.; Charles, J.; Grisso, J. A.; Jayadevappa, R.	2011	The Gerontologist	51 Suppl 1	NA	S59-72	10.1093/geront/gnr012	Stineman 2011	Irrelevant	Excluded by TI/AB review
575	Recruitment of ischemic stroke patients in clinical trials in general practice and implications for generalizability of results	Suri, M. F. K.; Qureshi, A. I.; Ezzeddine, M. A.	2011	Stroke	42	3	e295	10.1161/STR.0b013e3182074d9b	Suri 2011	Irrelevant	Excluded by TI/AB review
576	Rural caregiving in the United States: Research, practice, policy	Talley, Ronda C.; Chwalisz, Kathleen; Buckwalter, Kathleen C.	2011	Caregiving: Research, practice, policy	NA	NA	NA	10.1007/978-1-4614-0302-9	Talley 2011	Irrelevant	Excluded by TI/AB review
577	Comparative effectiveness of asthma interventions within a practice [illegible]	Tapp, Hazel; Hebert, Lisa; Dulin, Michael	2011	BMC Health Services Research	11	1	188-188	10.1186/1472-6963-11-188	Tapp 2011	Irrelevant	Excluded by TI/AB review

S4 Table. All studies identified in the literature search

579	Suicidal risk behavior in emerging adult ed patients screened for substance use	Tarantino, N.; Kuperminc, G.	2011	Journal of Adolescent Health	48	2	S70	10.1016/j.jadohealth.2010.11.149	Tarantino 2011	Irrelevant	Excluded by TI/AB review
580	The association between bioimpedance vector analysis measurements and disposition	Tuy, T.; Talati, A.; Fiessinger, L.; Peacock, W. F.	2011	Annals of Emergency Medicine	58	4	S213	10.1016/j.annemergmed.2011.06.132	Tuy 2011	Irrelevant	Excluded by TI/AB review
581	Bioimpedance vector analysis for volume assessment in dyspneic patients	Tuy, T. T.; Talati, A.; Fiessinger, L.; Peacock, W. F.	2011	Annals of Emergency Medicine	58	4	S212	10.1016/j.annemergmed.2011.06.130	Tuy 2011	Irrelevant	Excluded by TI/AB review

S4 Table. All studies identified in the literature search

582	Effect of closure of a local safety-net hospital on primary care physicians' perceptions of their role in patient care	Walker, K. O.; Clarke, R.; Ryan, G.; Brown, A. F.	2011	Annals of Family Medicine	9	6	496-503	NA	Walker 2011	Irrelevant	Excluded by TI/AB review
583	Effect of providing tailored primary care on health care utilization and return to jail among recently released prisoners: A randomized controlled trial	Wang, E. A. H.; Hong, C. S.; Shavit, S.; Kessell, E.; Sanders, R.; Moos, K.; Kushel, M. B.	2011	Journal of General Internal Medicine	26	NA	S251-S252	10.1007/s11606-011-1730-9	Wang 2011	Irrelevant	Excluded by TI/AB review

S4 Table. All studies identified in the literature search

584	A comparison of resource utilization between emergency physicians and pediatric emergency physicians	Weiner, S. G.; Ruffing, R. P.; Barnewolt, B. A.	2011	Academic Emergency Medicine	18	5	S18-S19	10.1111/j.1553-2712.2011.01073.x	Weiner 2011	Irrelevant	Excluded by TI/AB review
586	Increased acceptance rates of HIV screening using opt-out consent methods in an urban emergency department	White, D. A. E.; Sadoun, T.; Tran, T.; Alter, H. J.	2011	Journal of Acquired Immune Deficiency Syndromes	58	3	277-282	10.1097/QAI.0b013e318231916d	White 2011	Irrelevant	Excluded by TI/AB review
587	Implementation of a guideline for the treatment of pain, sedation, agitation and neuromuscular blockade in the mechanically ventilated adult patient in the [illegible]	White, K. E.; Szumita, P. M.; Gilboy, N.; Keenan, H. A.; Arbelaez, C.	2011	Open Access Emergency Medicine	3	NA	21-27	10.2147/OAEM.S17042	White 2011	Irrelevant	Excluded by TI/AB review

S4 Table. All studies identified in the literature search

588	Self-reported violence-related outcomes for adolescents within eight weeks of emergency [illegible]	Wiebe, D.; Blackstone, M.; Mollen, C.; Culyba, A.; Fein, J.	2011	Journal of Adolescent Health	48	2	S86-S87	10.1016/j.jadohealth.2010.11.181	Wiebe 2011	Irrelevant	Excluded by TI/AB review
589	The acceptability of mental health screening in a pediatric emergency department	Williams, J. R.; Ho, M. L.; Grupp-Phelan, J.	2011	Pediatric Emergency Care	27	7	611-615	10.1097/PEC.0b013e318222554e	Williams 2011	Irrelevant	Excluded by TI/AB review
590	Making education count: The nurse's role in asthma education using a medical home model of care	Wood, M. R.; Bolyard, D.	2011	Journal of Pediatric Nursing	26	6	552-558	10.1016/j.pedn.2010.06.009	Wood 2011	Irrelevant	Excluded by TI/AB review

S4 Table. All studies identified in the literature search

591	Presenting blood pressure in traumatic brain injury: A bimodal distribution of death	Zafar, S. N.; Millham, F. H.; Chang, Y.; Fikry, K.; Alam, H. B.; King, D. R.; Velmahos, G. C.; De Moya, M. A.	2011	Journal of Trauma - Injury, Infection and Critical Care	71	5	1179-1184	10.1097/TA.0b013e3182140d38	Zafar 2011	Irrelevant	Excluded by TI/AB review
592	Does gun accessibility lead to violence-related injury?	Zun, L.; Zun, L.	2011	Academic Emergency Medicine	18	5	S218	10.1111/j.1553-2712.2011.01073.x	Zun 2011	Irrelevant	Excluded by TI/AB review

S4 Table. All studies identified in the literature search

593	Etiology of uncompleted exercise stress testing after ED chest pain evaluation	Zwischenberger, B. A.; Moore, B. J.; Luber, S. D.; Dallo, F. J.	2011	American Journal of Emergency Medicine	29	4	427-431	10.1016/j.ajem.2010.01.001	Zwischenberger 2011	Irrelevant	Excluded by TI/AB review
594	Proceedings of Research in Clinical Practice 2011	NA	2012	Qjm	105	6	NA	NA	NA	Irrelevant	Excluded by TI/AB review
595	Disparities between public and private hospitals within a single academic center in the management of gallstone pancreatitis	Abouzeid, M.; Graffeo, C. S.; Nguyen, A.; Marr, M.; Ayo, D.; Obeid, N.; Bryk, D.; Leon Pachter, H.; Cohen, S. M.	2012	Pancreas	41	8	1344	10.1097/MPA.0b013e318270446a	Abouzeid 2012	Irrelevant	Excluded by TI/AB review

S4 Table. All studies identified in the literature search

596	Validation of the care transition measure in multi-ethnic South-East Asia in Singapore	Bakshi, Anu Birla; Wee, Shiou-Liang; Tay, Charlene; Wong, Loong-Mun; Leong, Ian Yi-Onn; Merchant, Reshma A.; Luo, Nan	2012	BMC Health Services Research	12	1	256-256	10.1186/1472-6963-12-256	Bakshi 2012	Irrelevant	Excluded by TI/AB review
598	Gender, age and ethnic aspects of analgesia in acute abdominal pain: is analgesia even across the groups?	Banz, V. M.; Christen, B.; Paul, K.; Martinolli, L.; Candinas, D.; Zimmermann, H.; Exadaktylos, A. K.	2012	Internal Medicine Journal	42	3	281-288	10.1111/j.1445-5994.2010.02255.x	Banz 2012	Irrelevant	Excluded by TI/AB review
599	Pain care in the emergency department: Is it satisfactory?	Barlow, M.; Hwang, U.	2012	Journal of the American Geriatrics Society	60	NA	S83	10.1111/j.1532-5415.2012.04000.x	Barlow 2012	Irrelevant	Excluded by TI/AB review

S4 Table. All studies identified in the literature search

600	A systematic review of the effectiveness of patient-centred care on emergency room visits, hospitalizations, [illegible]	Barnes, Cheryl; Cauvin, Eugene; Duran-Kim, Meral; Montalbano, Lisa; Londrigan, Marie	2012	JBI Library of Systematic Reviews	10	14	832-894	10.11124/jbisrir-2012-67	Barnes 2012	Irrelevant	Excluded by TI/AB review
601	Short communication routine HIV testing in the emergency [illegible]	Batey, D. S.; Hogan, V. L.; Cantor, R.; Hamlin, C. M.; Ross-Davis, K.; Nevin, C.; Zimmerman, C.; [illegible]	2012	AIDS Research and Human Retroviruses	28	4	352-356	10.1089/aid.2011.0074	Batey 2012	Irrelevant	Excluded by TI/AB review
602	Burden of comorbid depression/anxiety among medicare beneficiaries with chronic obstructive pulmonary disease	Blanchette, C. M.; Roberts, M.; Petersen, H.; Fuhlbrigge, A.	2012	Value in Health	15	4	A52	10.1016/j.jval.2012.03.288	Blanchette 2012	Irrelevant	Excluded by TI/AB review
603	The appropriateness of, and compliance with, telephone	Blank, L.; Coster, J.; O'Cathain, A.; Knowles, E.; Tosh, J.; Turner, J.;	2012	Journal of Advanced Nursing	68	12	2610-2621	10.1111/j.1365-2648.2012.06052.x	Blank 2012	Irrelevant	Excluded by TI/AB review
605	Computer access and internet use by urban and suburban emergency department customers	Bond, M. C.; Klemt, R.; Merlis, J.; Kopinski, J. E.; Hirshon, J. M.	2012	Journal of Emergency Medicine	43	1	159-165	10.1016/j.jemermed.2011.03.034	Bond 2012	Irrelevant	Excluded by TI/AB review

S4 Table. All studies identified in the literature search

606	Web-based emergency department patient satisfaction	Broadwater-Hollifield, C.; Fair, J.; Podolsky, S.; Carey, J.; Stephen,	2012	Annals of Emergency Medicine	60	4	S33	10.1016/j.annemergmed.2012.06.366	Broadwater-Hollifield 2012	Irrelevant	Excluded by TI/AB review
607	Factors influencing emergency department preference for access to healthcare	Brown, L. E.; Burton, R.; Hixon, B.; Kakade, M.; Bhagalia, P.; Vick, C.; Edwards, A.; Hawn, M. T.	2012	Western Journal of Emergency Medicine	13	5	410-415	10.5811/westjem.2011.11.6820	Brown 2012	Irrelevant	Excluded by TI/AB review
608	Patient attitudes and expectations towards HIV testing	Burgess, K. R.; Chandar, A. K.; Mohs, H.; Radonich, K.; Manik, A.; Totten, V. Y.	2012	Academic Emergency Medicine	19	NA	S211	10.1111/j.1553-2712.2012.01332.x	Burgess 2012	Irrelevant	Excluded by TI/AB review
609	The greater cincinnati pediatric clinic repository: A novel framework for childhood asthma and allergy research	Butsch Kovacic, M.; Biagini Myers, J. M.; Lindsey, M.; Patterson, T.; Sauter, S.; Ericksen, M. B.; Ryan, P.; Assa'Ad, A.; Lierl, M.; Fischer, T.; [illegible]	2012	Pediatric, Allergy, Immunology, and Pulmonology	25	2	104-113	10.1089/ped.2011.0116	ButschKovacic 2012	Irrelevant	Excluded by TI/AB review

S4 Table. All studies identified in the literature search

610	PDE5 inhibitor treatment persistence and adherence in latin american men with erectile dysfunction	Cairoli, C. E.; Magno, L. A.; Henneges, C.	2012	Journal of Sexual Medicine	9	NA	314	10.1111/jsm.12007-1	Cairoli 2012	Irrelevant	Excluded by TI/AB review
611	Association of enrolment in primary care networks with diabetes care and outcomes among First Nations and low-income Albertans	Campbell, D. J.; Ronksley, P. E.; Hemmelgarn, B. R.; Zhang, J.; Barnabe, C.; Tonelli, M.; Manns, B.	2012	Open medicine : a peer-reviewed, independent, open-access journal	6	4	e155-165	NA	Campbell 2012	Irrelevant	Excluded by TI/AB review
612	Does cognitive dysfunction affect quality of life assessment in emergency department geriatric patients?	Carpenter, C. R.; Wahidi, L.	2012	Academic Emergency Medicine	19	NA	S292	10.1111/j.1553-2712.2012.01332.x	Carpenter 2012	Irrelevant	Excluded by TI/AB review

S4 Table. All studies identified in the literature search

613	Encyclopedia of body image and human appearance, Vol 2	Cash, Thomas F.	2012	NA	NA	NA	NA	NA	Cash 2012	Irrelevant	Excluded by TI/AB review
614	Prescribing patterns and predictors for heart failure therapy in elderly patients receiving home health care	Chang, E.; Tien, Y.; Tang, Y.; Reist, J.; Doucette, W.	2012	Journal of the American Pharmacists Association	52	2	216	10.1331/JAPhA.2012.12510	Chang 2012	Irrelevant	Excluded by TI/AB review
615	The association between medical home readiness, quality of care, and resource use	Chen, L. M.; Sakshaug, J.; Miller, D. C.; Wu, M.; Hollingsworth, J. M.	2012	Journal of General Internal Medicine	27	NA	S315	NA	Chen 2012	Irrelevant	Excluded by TI/AB review

S4 Table. All studies identified in the literature search

616	Exploration of clinically significant adverse events in adult non-traumatic emergency [illegible]	Chern, C. H.; Wang, L. M.; How, C. K.	2012	Journal of Acute Medicine	2	1	19-25	10.1016/j.jacme.2012.03.001	Chern 2012	Irrelevant	Excluded by TI/AB review
617	The perceived barriers to treatment seeking behaviour in erectile dysfunction (ED) [illegible]	Chia Yew, K.; Dw, T.; Kw, T.; Ah, R.; George Eng Geap, L.	2012	International Journal of Urology	19	NA	203	10.1111/j.1442-2042.2012.03167.x	ChiaYew 2012	Irrelevant	Excluded by TI/AB review
618	Communicating with patients with limited english proficiency: Analysis of interpreter use and comfort with a second language	Chiu, D. T.; Fisher, J.; Landry, A.	2012	Academic Emergency Medicine	19	NA	S89	10.1111/j.1553-2712.2012.01332.x	Chiu 2012	Irrelevant	Excluded by TI/AB review
619	Understanding patient acceptance and refusal of HIV testing in the emergency department	Christopoulos, K. A.; Weiser, S. D.; Koester, K. A.; Myers, J. J.; White, D. A.; Kaplan, B.; Morin, S. F.	2012	BMC Public Health	12	NA	3	10.1186/1471-2458-12-3	Christopoulos 2012	Irrelevant	Excluded by TI/AB review

S4 Table. All studies identified in the literature search

620	The head-off environmental asthma in Louisiana (HEAL) study-methods and study population	Chulada, P. C.; Kennedy, S.; Mvula, M. M.; Jaffee, K.; Wildfire, J.; Thornton, E.; Cohn, R. D.; Faye Grimsley, L.; Mitchell, H.; El-Dahr, J.; Sterling, Y.; Martin, W. J.; White, [illegible]	2012	Environmental Health Perspectives	120	11	1592-1599	10.1289/ehp.1104239	Chulada 2012	Irrelevant	Excluded by TI/AB review
621	The effect of adherence to combination therapy of long acting beta agonist and inhaled corticosteroid (LABA = ICS) on emergency department (ED) utilization in newly [illegible]	Chung, J.; Nichol, M. B.	2012	Value in Health	15	4	A57	10.1016/j.jval.2012.03.317	Chung 2012	Irrelevant	Excluded by TI/AB review
622	Puerto Rican Families' Experiences of Asthma and Use of the Emergency Department for Asthma Care	Coffey, J.; Cloutier, M.; Meadows-Oliver, M.; Terrazos, C.	2012	Journal of Pediatric Health Care	26	5	356-363	10.1016/j.pedhc.2011.01.006	Coffey 2012	Irrelevant	Excluded by TI/AB review

S4 Table. All studies identified in the literature search

623	HIV screening acceptance in an ethnically diverse urban emergency department	Cohen, H.; McArthur, L.; Likourezos, A.; Weiner, C.; Sable, K.; Nehrbass, E.; Rizkalla, C.; Vazquez, H.; [illegible]	2012	Academic Emergency Medicine	19	NA	S297	10.1111/j.1553-2712.2012.01332.x	Cohen 2012	Irrelevant	Excluded by TI/AB review
624	Comprehensive care for sickle cell disease immigrant patients: A reproducible model achieving high adherence to minimum standards of care	Colombatti, R.; Montanaro, M.; Guasti, F.; Rampazzo, P.; Meneghetti, G.; Giordan, M.; Basso, G.; Sainati, L.	2012	Pediatric Blood and Cancer	59	7	1275-1279	10.1002/pbc.24110	Colombatti 2012	Irrelevant	Excluded by TI/AB review
625	The impact of health literacy and socioeconomic status on asthma disparities	Curtis, L. M.; Wolf, M. S.; Weiss, K. B.; Grammer, L. C.	2012	Journal of Asthma	49	2	178-183	10.3109/02770903.2011.648297	Curtis 2012	Irrelevant	Excluded by TI/AB review

S4 Table. All studies identified in the literature search

626	Perceptions of erectile dysfunction as a disease entity and	Cy, K.; Kw, T.; Dw, T.; Ah, R.; George, E.	2012	International Journal of Urology	19	NA	390	10.1111/j.1442-2042.2012.03167.x	Cy 2012	Irrelevant	Excluded by TI/AB review
627	Estimates of schizophrenia-related direct medical costs using attributable and incremental cost approaches	Desai, P.; Lawson, K.	2012	Value in Health	15	4	A86-A87	10.1016/j.jval.2012.03.474	Desai 2012	Irrelevant	Excluded by TI/AB review
628	Identifying characteristics of patients with high schizophreniarelated costs	Desai, P.; Lawson, K.; Rascati, K. L.; Barner, J. C.	2012	Value in Health	15	4	A86	10.1016/j.jval.2012.03.473	Desai 2012	Irrelevant	Excluded by TI/AB review

S4 Table. All studies identified in the literature search

629	The impact of watching cartoons for distraction during painful procedures in the emergency department	Downey, L. V.; Zun, L. S.	2012	Pediatric Emergency Care	28	10	1033-1035	10.1097/PEC.0b013e31826cac1a	Downey 2012	Irrelevant	Excluded by TI/AB review
630	Physician rating of medication adherence among urban early adolescents with asthma	Eakin, M. N.; Borrelli, B.; Rand, C. S.; Riekert, K. A.	2012	American Journal of Respiratory and Critical Care Medicine	185	NA	NA	NA	Eakin 2012	Irrelevant	Excluded by TI/AB review
631	Asthma in head start children: Effects of the breathmobile program and family communication on asthma outcomes	Eakin, M. N.; Rand, C. S.; Bilderback, A.; Bollinger, M. E.; Butz, A.; Kandasamy, V.; Riekert, K. A.	2012	Journal of Allergy and Clinical Immunology	129	3	664-670	10.1016/j.jaci.2011.10.013	Eakin 2012	Irrelevant	Excluded by TI/AB review

S4 Table. All studies identified in the literature search

633	Cancer disparities: Causes and evidence-based solutions	Elk, Ronit; Landrine, Hope	2012	NA	NA	NA	NA	NA	Elk 2012	Irrelevant	Excluded by TI/AB review
634	Ethnic differences in caregiver quality of life in pediatric asthma	Everhart, R. S.; Koinis-Mitchell, D.; McQuaid, E. L.; Kopel, S.; Seifer, R.; Canino, G.; Fritz, G.	2012	Journal of Developmental and Behavioral Pediatrics	33	8	599-607	10.1097/DBP.0b013e318264c2b7	Everhart 2012	Irrelevant	Excluded by TI/AB review
635	Toward understanding the difference between using patients or encounters in the accounting of emergency department utilization	Fertel, B. S.; Hart, K. W.; Lindsell, C. J.; Ryan, R. J.; Lyons, M. S.	2012	Annals of Emergency Medicine	60	6	693-698	10.1016/j.annemergmed.2012.05.009	Fertel 2012	Irrelevant	Excluded by TI/AB review

S4 Table. All studies identified in the literature search

636	Noncompliance with follow-up appointments to outpatient specialty clinics after pediatric emergency [illegible]	Fishman, H. R.; Chu, R.; Singh, N.; Thabane, L.; Kam, A.	2012	Canadian Journal of Emergency Medicine	14	NA	S53	NA	Fishman 2012	Irrelevant	Excluded by TI/AB review
637	A randomized controlled trial of a comprehensive migraine intervention prior to discharge from an emergency department	Friedman, B. W.; Solorzano, C.; Norton, J.; Adewumni, V.; Campbell, C. M.; Esses, D.; Bijur, P. E.; Solomon, S.; Lipton, R. B.; John Gallagher, E.	2012	Academic Emergency Medicine	19	10	1151-1157	10.1111/j.1553-2712.2012.01458.x	Friedman 2012	Irrelevant	Excluded by TI/AB review
638	A Qualitative Analysis of the Factors Associated with Unanticipated Short-Term Death [illegible]	Gabayan, G. Z.; Sun, B. C.; Asch, S. M.; Timmermans, S.; Hoffman, J. R.; Derose, S. F.	2012	Annals of Emergency Medicine	60	4	S74	10.1016/j.annemergmed.2012.06.182	Gabayan 2012	Irrelevant	Excluded by TI/AB review
639	Examining the signs and symptoms experienced by	Greenslade, J. H.; Cullen, L.; Parsonage, W.; Reid, C. M.; Body, R.;	2012	Annals of Emergency Medicine	60	6	777-785.e3	10.1016/j.annemergmed.2012.05.008	Greenslade 2012	Irrelevant	Excluded by TI/AB review

S4 Table. All studies identified in the literature search

640	Comparison of throughput times for limited English proficiency patient visits in the emergency department between different [illegible]	Grover, A.; Deakyne, S.; Bajaj, L.; Roosevelt, G. E.	2012	Journal of immigrant and minority health / Center for Minority Public [illegible]	14	4	602-607	10.1007/s10903-011-9532-z	Grover 2012	Irrelevant	Excluded by TI/AB review
642	Inhaled corticosteroid and long-acting beta-agonist medication compliance in patients with moderate and severe asthma	Guo, J. J.; Kelton, C. M.; Tsai, K.; Cai, B.; Bian, B.; Wigle, P. R.	2012	Value in Health	15	4	A57	10.1016/j.jval.2012.03.314	Guo 2012	Irrelevant	Excluded by TI/AB review
643	The complex association of race and leaving the pediatric emergency department without being seen by a physician	Harrison, B.; Finkelstein, M.; Puumala, S.; Payne, N. R.	2012	Pediatr Emerg Care	28	11	1136-45	10.1097/PEC.0b013e31827134db	Harrison 2012	Irrelevant	Excluded by TI/AB review

S4 Table. All studies identified in the literature search

647	Older veterans and emergency department discharge information	Hastings, S.; Stechuchak, K.; Oddone, E.; Weinberger, M.; Tucker, D.; Knaack, W.; Schmader, K.	2012	BMJ Quality and Safety	21	10	835-842	10.1136/bmjqs-2011-000538	Hastings 2012	Irrelevant	Excluded by TI/AB review
649	Incarceration among adults who are in the public mental health system: Rates, risk factors, and short-term outcomes	Hawthorne, W. B.; Aarons, G. A.; Folsom, D. P.; Conklin, R. M.; Sommerfeld, D. H.; Solorzano, E.; Lanouette, N. M.; Lindamer, L. A.; Lewis, M.; Jeste, D. V.	2012	Psychiatric Services	63	1	26-32	10.1176/appi.ps.201000505	Hawthorne 2012	Irrelevant	Excluded by TI/AB review
650	Long-term healthcare costs and functional outcomes associatedwith [illegible]	Haynes, V. S.; Zhu, B.; Stauffer, V.; Kinon, B. J.; Xu, L.; Ascher-Svanum, H.	2012	Schizophrenia Research	136	NA	S228-S229	NA	Haynes 2012	Irrelevant	Excluded by TI/AB review

S4 Table. All studies identified in the literature search

651	Discerning the feasibility of developing a smartphone application based intervention promoting HPV [illegible]	Hill, M. J.; Granado, M.; Williams, M.	2012	Annals of Emergency Medicine	60	4	S84	10.1016/j.annemergmed.2012.06.209	Hill 2012	Irrelevant	Excluded by TI/AB review
652	Shared decision making in the selection of outpatient analgesics for older emergency department patients	Isaacs, C. G.; Kistler, C.; Hunold, K.; Pereira, G.; Buchbinder, M.; McLean, S. A.; Platts-Mills, T. F.	2012	Academic Emergency Medicine	19	NA	S290-S291	10.1111/j.1553-2712.2012.01332.x	Isaacs 2012	Irrelevant	Excluded by TI/AB review
653	Shared decision making in the selection of outpatient analgesics for older emergency department patients	Isaacs, C. G.; Kistler, C. E.; Hunold, K.; Pereira, G.; Buchbinder, M.; McLean, S. A.; Platts-Mills, T. F.	2012	Journal of the American Geriatrics Society	60	NA	S82	10.1111/j.1532-5415.2012.04000.x	Isaacs 2012	Irrelevant	Excluded by TI/AB review

S4 Table. All studies identified in the literature search

654	Active surveillance for prostate cancer: An Australian experience	Ischia, J. J.; Pang, C. Y.; Tay, Y. K.; Li Wai Suen, C. F. D.; Aw, H. C.; Frydenberg, M.	2012	BJU International	109	SUPP L. 3	40-43	10.1111/j.1464-410X.2012.11045.x	Ischia 2012	Irrelevant	Excluded by TI/AB review
655	Study protocol for Women of Color and Asthma Control: a randomized controlled trial of [illegible]	Janevic, M. R.; Sanders, G. M.; Thomas, L. J.; Williams, D. M.; Nelson, B.; Gilchrist, E.; Johnson, T. R.; [illegible]	2012	BMC Public Health	12	NA	76	10.1186/1471-2458-12-76	Janevic 2012	Irrelevant	Excluded by TI/AB review
658	Disparities in potentially avoidable emergency department (ED) care: ED visits for ambulatory care sensitive conditions	Johnson, P. J.; Ghildayal, N.; Ward, A. C.; Westgard, B. C.; Boland, L. L.; Hokanson, J. S.	2012	Med Care	50	12	1020-8	10.1097/MLR.0b013e318270bad4	Johnson 2012	Irrelevant	Excluded by TI/AB review

S4 Table. All studies identified in the literature search

660	Implementing performance improvement in New Zealand	Jones, P.; Chalmers, L.; Wells, S.; Ameratunga, S.; Carswell, P.; Ashton,	2012	BMC Health Services Research	12	NA	NA	10.1186/1472-6963-12-45	Jones 2012	Irrelevant	Excluded by TI/AB review
661	Emergency department patients self-report higher patient inertia, hopelessness, and harmful lifestyle choices than [illegible]	Joyner, J.; Moore, A. R.; Mount, D. L.; Simmons, D. R.; Ferrario, C. M.; Cline, D. M.	2012	Journal of Clinical Hypertension	14	12	828-835	10.1111/jch.12001	Joyner 2012	Irrelevant	Excluded by TI/AB review
662	Effect of screening for partner violence on women's quality of life: A randomized controlled trial	Klevens, J.; Kee, R.; Trick, W.; Garcia, D.; Angulo, F. R.; Jones, R.; Sadowski, L. S.	2012	JAMA - Journal of the American Medical Association	308	7	681-689	10.1001/jama.2012.6434	Klevens 2012	Irrelevant	Excluded by TI/AB review

S4 Table. All studies identified in the literature search

663	Using research to demonstrate and improve quality of care in student-led clinics	Knudsen, J.; Huppert, L.; Dempsey, K. E.; Donelan, K.; Berman, R.	2012	Journal of General Internal Medicine	27	NA	S572	NA	Knudsen 2012	Irrelevant	Excluded by TI/AB review
664	The acceptability and expectations towards PDE5-I in ED treatment in cultural and socio-economically diverse Asian populations	Kong, C.; Tiang, K.; Ng, K. L.; Lee, G.; Abdul Razack, A.	2012	Urology	80	3	S43	10.1016/S0090-4295(12)00880-1	Kong 2012	Irrelevant	Excluded by TI/AB review

S4 Table. All studies identified in the literature search

665	Assessing clinical research participants' experiences	Kost, R. G.; Lee, L. M.; Yessis, J.; Henderson, D. K.; Coller, B. S.	2012	Clinical and Translational Science	5	2	198	10.1111/j.1752-8062.2012.00398.x	Kost 2012	Irrelevant	Excluded by TI/AB review
666	Describing adherence to recommended preventative care behaviors among adults with sickle cell disease	Lanzkron, S.; Haywood, C.; Onojobi, G. T.; Strouse, J. J.; Beach, M. C.	2012	Blood	120	21	NA	NA	Lanzkron 2012	Irrelevant	Excluded by TI/AB review
667	Accepting ED: The challenges and obstacles ahead	Lee, G.	2012	Journal of Sexual Medicine	9	NA	99-100	10.1111/j.1743-6109.2012.02645.x	Lee 2012	Irrelevant	Excluded by TI/AB review

S4 Table. All studies identified in the literature search

668	Are patients with longer emergency department wait times less likely to consent to research?	Limkakeng Jr, A. T.; Glickman, S. W.; Shofer, F.; Mani, G.; Drake, W.; Freeman, D.; Ascher, S.; Pietrobon, R.; Cairns, C. B.	2012	Academic Emergency Medicine	19	4	396-401	10.1111/j.1553-2712.2012.01310.x	LimkakengJr 2012	Irrelevant	Excluded by TI/AB review
669	Frequent users of the emergency department: Do they make visits that can be [illegible]	Liu, S. W.; Nagurney, J. T.; Chang, Y.; Smulowitz, P. B.; Parry, B. A.; [illegible]	2012	Annals of Emergency Medicine	60	4	S155	10.1016/j.annemergmed.2012.06.472	Liu 2012	Irrelevant	Excluded by TI/AB review
670	Use of interpreters by physicians for hospitalized limited english proficiency patients and its impact on patient outcomes	Lopez, L.; Soukup, J. R.; Hicks, L. R.	2012	Journal of General Internal Medicine	27	NA	S342-S343	NA	Lopez 2012	Irrelevant	Excluded by TI/AB review
671	The impact of frequent attenders to the emergency	Lownes, S.; Jones, E.	2012	Emergency Medicine Journal	29	NA	A13	10.1136/emermed-2012-201246.11	Lownes 2012	Irrelevant	Excluded by TI/AB review

S4 Table. All studies identified in the literature search

672	21-Gene recurrence scores	Lund, M. J.; Mosunjac, M.; Davis, K. M.; Gabram-Mendola, S.; Rizzo, M.; Bumpers, H. L.; Hearn, S.; Zelnak, A.; Styblo, T.; O'Regan, R. M.	2012	Cancer	118	3	788-796	10.1002/cncr.26180	Lund 2012	Irrelevant	Excluded by TI/AB review
673	Attitudes of ED staff to the presence of family during cardiopulmonary resuscitation: A Trinidad and Tobago perspective	Mahabir, D.; Sammy, I.	2012	Emergency Medicine Journal	29	10	817-820	10.1136/emermed-2011-200742	Mahabir 2012	Irrelevant	Excluded by TI/AB review
674	The health care experiences of Travellers compared to the general population: the All-Ireland Traveller Health [illegible]	McGorrian, Catherine; Frazer, Kate; Daly, Leslie; Moore, Ronnie G.; Turner, Jill; Sweeney, Mary Rose; Staines, [illegible]	2012	Journal of Health Services Research & Policy	17	3	173-180	10.1258/jhsrp.2011.011079	McGorrian 2012	Irrelevant	Excluded by TI/AB review

S4 Table. All studies identified in the literature search

675	A cluster of nonspecific adverse events in a military reserve unit following pandemic influenza A (H1N1) 2009 vaccination-Possible stimulated reporting?	McNeil, M. M.; Arana, J.; Stewart, B.; Hartshorn, M.; Hrncir, D.; Wang, H.; Lamias, M.; Locke, M.; Stamper, J.; Tokars, J. I.; Engler, R. J.	2012	Vaccine	30	14	2421-2426	10.1016/j.vaccine.2012.01.072	McNeil 2012	Irrelevant	Excluded by TI/AB review
676	Association of age, gender, and race with intensity of end-of-life care for medicare beneficiaries with cancer	Miesfeldt, S.; Murray, K.; Lucas, L.; Chang, C. H.; Goodman, D.; Morden, N. E.	2012	Journal of Palliative Medicine	15	5	548-554	10.1089/jpm.2011.0310	Miesfeldt 2012	Irrelevant	Excluded by TI/AB review
677	Attitudes toward health care robot assistants in the ED: A survey Of ED patients and visitors	Miller, K. F.; Self, W. H.; McNaughton, C. D.; Mion, L. C.; Storrow, A. B.	2012	Academic Emergency Medicine	19	NA	S116	10.1111/j.1553-2712.2012.01332.x	Miller 2012	Irrelevant	Excluded by TI/AB review

S4 Table. All studies identified in the literature search

678	Online counselling for eating disorders: Reaching an underserved population?	Moessner, M.; Bauer, S.	2012	Journal of Mental Health	21	4	336-345	10.3109/09638237.2011.643512	Moessner 2012	Irrelevant	Excluded by TI/AB review
679	Understanding the Critical Role of Social Work in Safety Net Medical Settings: Framework for Research and Practice in the Emergency [illegible]	Moore, M.; Ekman, E.; Shumway, M.	2012	Social Work in Health Care	51	2	140-148	10.1080/00981389.2011.610872	Moore 2012	Irrelevant	Excluded by TI/AB review
680	The Cognitive Assessment System: From theory to practice	Naglieri, Jack A.; Otero, Tulio M.; Flanagan, Dawn P.; Harrison, Patti L.	2012	Contemporary intellectual assessment: Theories, tests, and issues., 3rd ed.	NA	NA	376-399	NA	Naglieri 2012	Irrelevant	Excluded by TI/AB review
681	Cranial Computed Tomography Use Among Children With Minor Blunt Head Trauma	Natale, J. E.; Joseph, J. G.; Rogers, A. J.; Mahajan, P.; Cooper, A.; Wisner, D. H.; Miskin, M. L.; Hoyle, J. D.; Atabaki, S. M.; [illegible]	2012	Archives of Pediatrics & Adolescent Medicine	166	8	732-737	NA	Natale 2012	Irrelevant	Excluded by TI/AB review

S4 Table. All studies identified in the literature search

682	The Breast Cancer Quality of Care Study (BQUAL): a multi-center study to determine causes for noncompliance with breast cancer [illegible]	Neugut, A. I.; Hillyer, G. C.; Kushi, L. H.; Lamerato, L.; Nathanson, S. D.; Ambrosone, C. B.; Bovbjerg, D. H.; Mandelblatt, J. S.; Magai, C.; Tsai, W. [illegible]	2012	Breast J	18	3	203-13	10.1111/j.1524-4741.2012.01240.x	Neugut 2012	Irrelevant	Excluded by TI/AB review
683	Characteristics of people with epilepsy who attend emergency departments: Prospective study of metropolitan [illegible]	Noble, A. J.; Goldstein, L. H.; Seed, P.; Glucksman, E.; Ridsdale, L.	2012	Epilepsia	53	10	1820-1828	10.1111/j.1528-1167.2012.03586.x	Noble 2012	Irrelevant	Excluded by TI/AB review
684	Ethnicity and reported pain scores among children with long-bone fractures requiring emergency care	Ortega, H. W.; Velden, H. V.; Lin, C. W.; Reid, S.	2012	Pediatric Emergency Care	28	11	1146-1149	10.1097/PEC.0b013e31827134f6	Ortega 2012	Irrelevant	Excluded by TI/AB review

S4 Table. All studies identified in the literature search

685	Managing diversity in today's workplace: Strategies for employees and employers, Vol 1: Gender, race, sexual orientation, ethnicity, and power, Vol 2: Work-family	Paludi, Michele A.	2012	Women and careers in management	NA	NA	NA	NA	Paludi 2012	Irrelevant	Excluded by TI/AB review
686	Concentration of hospital care for acute sickle cell disease-related visits	Panepinto, J.; Owens, P.; Mosso, A.; Steiner, C.; Brousseau, D.	2012	Pediatric Blood and Cancer	59	4	685-689	10.1002/pbc.24028	Panepinto 2012	Irrelevant	Excluded by TI/AB review
687	Stakeholder perceptions of a nurse led walk-in centre	Parker, Rhian M.; Desborough, Jane L.; Forrest, Laura E.	2012	BMC Health Services Research	12	1	382-382	10.1186/1472-6963-12-382	Parker 2012	Irrelevant	Excluded by TI/AB review
688	More educated emergency department patients are less likely to receive opioids for acute [illegible]	Platts-Mills, T. F.; Hunold, K. M.; Bortsov, A. V.; Soward, A. C.; Peak, D. A.; Jones, J. S.; Swor, R. A.; Lee, D. [illegible]	2012	PAIN	153	5	967-973	10.1016/j.pain.2012.01.013	Platts-Mills 2012	Irrelevant	Excluded by TI/AB review

S4 Table. All studies identified in the literature search

689	Do some trusts deliver a consistently better experience for patients? An analysis of patient experience across acute care surveys in English NHS trusts	Raleigh, Veena S.; Frosini, Francesca; Sizmur, Steve; Graham, Chris	2012	BMJ Quality & Safety	21	5	381-390	NA	Raleigh 2012	Irrelevant	Excluded by TI/AB review
690	The impact of asthma medication guidelines on asthma controller use and on asthma exacerbation rates comparing 19971998 and 20042005	Rank, M. A.; Liesinger, J. T.; Ziegenfuss, J. Y.; Branda, M. E.; Lim, K. G.; Yawn, B. P.; Shah, N. D.	2012	Annals of Allergy, Asthma and Immunology	108	1	13-Sep	10.1016/j.anai.2011.09.009	Rank 2012	Irrelevant	Excluded by TI/AB review
691	Patient perceptions of medical imaging from the emergency department	Repplinger, M. D.; Li, A.; Svenson, J. E.	2012	Academic Emergency Medicine	19	NA	S207	10.1111/j.1553-2712.2012.01332.x	Repplinger 2012	Irrelevant	Excluded by TI/AB review

S4 Table. All studies identified in the literature search

692	Correlation between unexpected return visits to a pediatric emergency department and the office hours and scheduling times of the primary care [illegible]	Rogers, B.; Attia, M.; Mercado, A.	2012	Pediatric Emergency Care	28	10	1100	NA	Rogers 2012	Irrelevant	Excluded by TI/AB review
693	Many patients who present to the emergency department don't believe their weight poses health risks: A disconnect [illegible]	Ryan, M.	2012	Academic Emergency Medicine	19	NA	S210	10.1111/j.1553-2712.2012.01332.x	Ryan 2012	Irrelevant	Excluded by TI/AB review
694	Novel approach to assessing pain burden in youth with sickle cell disease	Santanelli, J. P.; Zempsky, W. T.; O'Hara, E.	2012	American Journal of Hematology	87	7	E62-E63	10.1002/ajh.23331	Santanelli 2012	Irrelevant	Excluded by TI/AB review

S4 Table. All studies identified in the literature search

695	Prevalence and ecg findings for patients with false-positive cardiac catheterization laboratory activation among [illegible]	Sawyer, K. N.; Robinson, A. L.; Roberts, C. S.; Kurz, M. C.; Kontos, M. C.	2012	Academic Emergency Medicine	19	NA	S102-S103	10.1111/j.1553-2712.2012.01332.x	Sawyer 2012	Irrelevant	Excluded by TI/AB review
696	Childhood abuse, head injuries, and use of medical emergency services in people with severe mental Illness	Schneeberger, A. R.; Muenzenmaier, K. H.; Battaglia, J.; Castille, D.; Link, B. G.	2012	Journal of Aggression, Maltreatment and Trauma	21	5	570-582	10.1080/10926771.2012.678468	Schneeberger 2012	Irrelevant	Excluded by TI/AB review
698	Disparities among pediatric ED visits for unintentional injury in the United States	Schwartz, J.; McKay, M. P.	2012	Academic Emergency Medicine	19	NA	S340-S341	10.1111/j.1553-2712.2012.01332.x	Schwartz 2012	Irrelevant	Excluded by TI/AB review
699	Project professionalism	Slick, J.	2012	Annals of Emergency Medicine	60	4	S161-S162	10.1016/j.annemergmed.2012.06.491	Slick 2012	Irrelevant	Excluded by TI/AB review

S4 Table. All studies identified in the literature search

700	Identifying racial/ethnic disparities in diabetes management in federally supported health center patients	Sripipatana, A.; Lebrun, L. A.; Ngo-Metzger, Q.	2012	Journal of General Internal Medicine	27	NA	S215-S216	NA	Sripipatana 2012	Irrelevant	Excluded by TI/AB review
701	Transitional care of cardiac patients with psychiatric disorders: How bridging the discharge gap effectively measures-up	Stalhbaum, D. E.; Bumpus, S. M.; Smith, C. A.; Kline-Rogers, E.; Mahmood, R. Z.; Montgomery, D.; Krishnan, S. M.; Froehlich, J. B.; Eagle, K. A.; Rubenfire, M.	2012	Circulation: Cardiovascular Quality and Outcomes	5	3	NA	NA	Stalhbaum 2012	Irrelevant	Excluded by TI/AB review
702	Visible now? Black educational choices for the few, the desperate, and the far between	Stevenson, Howard C.; Slaughter-Defoe, Diana T.; Arrington, Edith G.; Johnson, Deborah J.; Stevenson, Howard C.; Slaughter-Defoe, Diana T.; Arrington, [illegible]	2012	Black educational choice: Assessing the private and public alternatives to traditional [illegible]	NA	NA	268-274	NA	Stevenson 2012	Irrelevant	Excluded by TI/AB review

S4 Table. All studies identified in the literature search

703	A mobile phone text message program to measure oral antibiotic use and provide feedback [illegible]	Suffoletto, B.; Calabria, J.; Ross, A.; Callaway, C.; Yealy, D. M.	2012	Academic Emergency Medicine	19	8	949-958	10.1111/j.1553-2712.2012.01411.x	Suffoletto 2012	Irrelevant	Excluded by TI/AB review
705	An assessment on Thai emergency medical services performance: The patient perspective	Suriyawongpaisal, P.; Tansirisithikul, R.; Srithamrongsawat, S.	2012	Journal of the Medical Association of Thailand	95	1	111-118	NA	Suriyawongpaisal 2012	Irrelevant	Excluded by TI/AB review
706	Are clinical diagnoses of Alzheimer's disease and other dementias affected by education and self-reported race?	Teresi, J. A.; Grober, E.; Eimicke, J. P.; Ehrlich, A. R.	2012	Psychological Assessment	24	3	531-544	10.1037/a0027008	Teresi 2012	Irrelevant	Excluded by TI/AB review
709	ACCEPT (Asian constraints and	Tiang, K. W.; Neo, N. Y.; Lim, K. L.;	2012	BJU International	109	NA	52-53	10.1111/j.1464-	Tiang 2012	Irrelevant	Excluded by TI/AB review
710	Predicting and decreasing low-acuity ED visits for an academic	Wagle, N.; Chokshi, D.; Gandy, L.; Goulart, D.; Hynes-Payack, M.;	2012	Journal of General Internal Medicine	27	NA	S533-S534	NA	Wagle 2012	Irrelevant	Excluded by TI/AB review

S4 Table. All studies identified in the literature search

712	Emergency department and hospital utilization among the homebound elderly	Wajnberg, A.; Smith, K. L.; Russell, D.; Ross, J. S.; Doucette, J.; Sofianou, A.; Federman, A.	2012	Journal of General Internal Medicine	27	NA	S182-S183	NA	Wajnberg 2012	Irrelevant	Excluded by TI/AB review
713	No difference in emergency department length of stay for limited english proficiency patients with [illegible]	Wallbrecht, J.; Hodes-Villamar, L.; Weiss, S.; Ernst, A.	2012	Annals of Emergency Medicine	60	4	S72	10.1016/j.annemergmed.2012.06.176	Wallbrecht 2012	Irrelevant	Excluded by TI/AB review
714	Professionalism module: An online discussion forum	Wallner, C. F.	2012	Annals of Emergency Medicine	60	4	S158	10.1016/j.annemergmed.2012.06.481	Wallner 2012	Irrelevant	Excluded by TI/AB review
715	Outcomes from the evaluation of an emergency department-based mental health [illegible]	Wand, T.; White, K.; Patching, J.; Dixon, J.; Green, T.	2012	Journal of the American Academy of Nurse [illegible]	24	3	149-159	10.1111/j.1745-7599.2011.00709.x	Wand 2012	Irrelevant	Excluded by TI/AB review
717	Development of palliative medicine consensus guidelines for consults in the emergency [illegible]	Waugh, D.	2012	Journal of Pain and Symptom Management	43	2	472-473	10.1016/j.jpainsymman.2011.12.265	Waugh 2012	Irrelevant	Excluded by TI/AB review

S4 Table. All studies identified in the literature search

719	Community Asthma Initiative: Evaluation of a quality improvement program for comprehensive asthma care	Woods, E. R.; Bhaumik, U.; Sommer, S. J.; Ziniel, S. I.; Kessler, A. J.; Chan, E.; Wilkinson, R. B.; Sesma, M. N.; Burack, A. B.; Klements, E. M.; [illegible]	2012	Pediatrics	129	3	465-472	10.1542/peds.2010-3472	Woods 2012	Irrelevant	Excluded by TI/AB review
721	Evaluation of the community asthma initiative: Adolescent vs. child outcomes and GIS mapping	Woods, E. R.; Fleegler, E.; Chan, E.; Bhaumik, U.; Sommer, S. J.; Ortiz, M. P.; Lorenzi, M.; Burack, A. B.; Klements, E. M.; Dickerson, D. U.; Nethersole, S.	2012	Journal of Adolescent Health	50	2	S83-S84	10.1016/j.jadohealth.2011.10.221	Woods 2012	Irrelevant	Excluded by TI/AB review
722	Creating models for global health and primary care: A team-based approach for managing global [illegible]	Wu, C. A.; Kakoza, R. M.; O'Brien, J. E.; Morris, C. A.; Tishler, L.; Rhatigan, J. J.; Ellner, A. L.	2012	Journal of General Internal Medicine	27	NA	S519-S520	NA	Wu 2012	Irrelevant	Excluded by TI/AB review

S4 Table. All studies identified in the literature search

723	The influence of physician seniority on disparities of admit/discharge decision making for ED patients	Wu, K. H.; Chen, I. C.; Li, C. J.; Li, W. C.; Lee, W. H.	2012	American Journal of Emergency Medicine	30	8	1555-1560	10.1016/j.ajem.2012.01.011	Wu 2012	Irrelevant	Excluded by TI/AB review
724	Telestroke consultations are executed quickly and efficiently	Yang, J. P.; Grujovski, A.; Wu, T. C.; Tegeler, C. H.; Xian, Y.; Olson, D. W. M.; Kolls, B. J.	2012	Circulation: Cardiovascular Quality and Outcomes	5	3	NA	NA	Yang 2012	Irrelevant	Excluded by TI/AB review
725	Evaluation of specialized medication packaging combined with medication therapy management: Adherence, [illegible]	Zillich, A. J.; Jaynes, H. A. W.; Snyder, M. E.; Harrison, J.; Hudmon, K. S.; De Moor, C.; French, D. D.	2012	Medical Care	50	6	485-493	10.1097/MLR.0b013e3182549d48	Zillich 2012	Irrelevant	Excluded by TI/AB review

S4 Table. All studies identified in the literature search

726	10th International Congress on Adolescent Health	NA	2013	Turkish Archives of Pediatrics	48	NA	1	NA	NA	Irrelevant	Excluded by TI/AB review
727	Disparities between public and private hospitals within a single academic center in the management of [illegible]	Abouzeid, M.; Graffeo, C. S.; Nguyen, A.; Marr, M.; Ayo, D.; Obeid, N.; Bryk, D.; Leon Pachter, H.; Cohen, S. M.	2013	Pancreatology	13	2	e11	NA	Abouzeid 2013	Irrelevant	Excluded by TI/AB review
728	The association of health literacy with adherence and outcomes in	Apter, A. J.; Wan, F.; Reisine, S.; Bender, B.; Rand, C.; Bogen, D. K.; Bennett, I. M.;	2013	Journal of Allergy and Clinical Immunolog	132	2	321-327	10.1016/j.jaci.2013.02.014	Apter 2013	Irrelevant	Excluded by TI/AB review

S4 Table. All studies identified in the literature search

729	The feasibility, acceptability, and preliminary effectiveness of a patient advocate for improving asthma outcomes	Apter, A. J.; Wan, F.; Reisine, S.; Bogen, D.; Rand, C. S.; Bender, B.; Gonzalez, R.; Priolo, C.; Roy, J.	2013	American Journal of Respiratory and Critical Care Medicine	187	NA	NA	NA	Apter 2013	Irrelevant	Excluded by TI/AB review
730	Feasibility, acceptability and preliminary effectiveness of patient advocates for improving asthma outcomes in adults	Apter, A. J.; Wan, F.; Reisine, S.; Bogen, D. K.; Rand, C.; Bender, B.; Bennett, I. M.; Gonzalez, R.; Priolo, C.; Sonnad, S. S.; Bryant-Stephens, T.; Ferguson, M.; Boyd, [illegible]	2013	Journal of Asthma	50	8	850-860	10.3109/02770903.2013.812655	Apter 2013	Irrelevant	Excluded by TI/AB review
732	Impact of educational attainment on health-related quality of life and [illegible]	Aquino-Beaton, C.; Persselin, J. E.; Weinreb, A.; Fang, M. A.; Singh, J. A.; Duffy, E.; Elashoff, [illegible]	2013	Arthritis and Rheumatism	65	NA	S846	10.1002/art.38216	Aquino-Beaton 2013	Irrelevant	Excluded by TI/AB review
733	Linking pediatric primary care obesity management to community programs	Ariza, A. J.; Hartman, J.; Grodecki, J.; Clavier, A.; Ghaey, K.; Elsner, M.; Moore, C.; Reina, O. O.; Binns, H. J.	2013	Journal of Health Care for the Poor and Underserved	24	SUPPL.2	158-167	10.1353/hpu.2013.0112	Ariza 2013	Irrelevant	Excluded by TI/AB review

S4 Table. All studies identified in the literature search

734	Trial to examine text-message based mhealth in ed patients with diabetes (TExT-MED)	Arora, S.; Burner, E.; Lam, J.; De Santos, R.; Meeks, A.; Menchine, M.	2013	Academic Emergency Medicine	20	5	S180	10.1111/acem.12115	Arora 2013	Irrelevant	Excluded by TI/AB review
735	Assessing the satisfaction of mobile health (mHealth) amongst ED inner-city patients with diabetes who received the TExT-MED intervention	Arora, S.; Burner, E.; Lam, J.; De Santos, R.; Menchine, M.	2013	Academic Emergency Medicine	20	5	S181	10.1111/acem.12115	Arora 2013	Irrelevant	Excluded by TI/AB review
736	Association of length of stay and disposition in pediatric emergency department patients	Barata, I. A.; Bailey, K.; Wu, M.; Rosen, L.; Sharieff, G. Q.; Dietrich, A. M.; Benjamin, L.; Amato, C. S.; Stephens, A.; Ward, M. F.; Mace, S. E.	2013	Academic Emergency Medicine	20	5	S36	10.1111/acem.12115	Barata 2013	Irrelevant	Excluded by TI/AB review

S4 Table. All studies identified in the literature search

737	A comparison of length of stay between admitted and discharged pediatric patients in the emergency department	Barata, I. A.; Mace, S. E.; Wu, M.; Rosen, L.; Bailey, K.; Stephens, A.; Dietrich, A. M.; Sharieff, G.; Amato, C. S.; Benjamin, L.	2013	Annals of Emergency Medicine	62	4	S111	10.1016/j.annemergmed.2013.07.132	Barata 2013	Irrelevant	Excluded by TI/AB review
738	Factors associated with length of stay in pediatric patients in the emergency department	Barata, I. A.; Mace, S. E.; Wu, M.; Rosen, L.; Bailey, K.; Stephens, A.; Dietrich, A. M.; Sharieff, G.; [illegible]	2013	Annals of Emergency Medicine	62	4	S109	10.1016/j.annemergmed.2013.07.126	Barata 2013	Irrelevant	Excluded by TI/AB review
739	Factors associated with patients declining kiosk-facilitated HIV self-testing in the emergency department	Beck, K.; Megan, G. K.; Woodfield, A.; Peterson, S.; Gaydos, C. A.; Rothman, R. E.; Hsieh, Y. H.	2013	Academic Emergency Medicine	20	5	S282-S283	10.1111/acem.12115	Beck 2013	Irrelevant	Excluded by TI/AB review
740	Linking cultural competence training to improved health outcomes: Perspectives from [illegible]	Betancourt, Joseph R.; Green, Alexander R.; LaVeist, Thomas A.; Isaac, Lydia A.	2013	Race, ethnicity, and health: A public health reader., 2nd [illegible]	NA	NA	689-695	NA	Betancourt 2013	Irrelevant	Excluded by TI/AB review

S4 Table. All studies identified in the literature search

741	Pathological gambling: Relationship to obesity, self-reported chronic medical [illegible]	Black, D. W.; Shaw, M.; McCormick, B.; Allen, J.	2013	Comprehensive Psychiatry	54	2	97-104	10.1016/j.comppsych.2012.07.001	Black 2013	Irrelevant	Excluded by TI/AB review
742	Propofol for procedural sedation in the emergency department: A qualitative systematic review	Black, E.; Campbell, S. G.; Magee, K.; Zed, P. J.	2013	Annals of Pharmacotherapy	47	6	856-868	10.1345/aph.1R743	Black 2013	Irrelevant	Excluded by TI/AB review
743	Nerve blocks for initial pain management of femoral fractures	Black, K. J.; Bevan, C. A.; Murphy, N. G.; Howard, J. J.	2013	Cochrane Database of Systematic Reviews	2013	12	NA	10.1002/14651858.CD009587.pub2	Black 2013	Irrelevant	Excluded by TI/AB review
744	Coordination of emergency department psychiatric care with psychiatry	Bregman, Benjamin L.; Powsner, Seth; Zun, Leslie S.; Chepenik, Lara G.; Mallory, Mary Nan S.	2013	Behavioral emergencies for the emergency physician.	NA	NA	291-296	10.1017/CBO9781139088077.044	Bregman 2013	Irrelevant	Excluded by TI/AB review

S4 Table. All studies identified in the literature search

745	Impact of a computerized order set on adherence to centers for disease control guidelines for the treatment of victims of sexual [illegible]	Britton, D. J.; Bloch, R. B.; Strout, T. D.; Baumann, M. R.	2013	Journal of Emergency Medicine	44	2	528-535	10.1016/j.jemermed.2012.06.016	Britton 2013	Irrelevant	Excluded by TI/AB review
746	Confronting racism in higher education: Problems and possibilities for fighting ignorance, bigotry and isolation	Brooks, Jeffrey S.; Arnold, Noelle Witherspoon	2013	Educational leadership for social justice	NA	NA	NA	NA	Brooks 2013	Irrelevant	Excluded by TI/AB review
747	Understanding symptom experiences of older individuals with acute coronary syndrome	Bruno, Carolynn M.	2013	NA	NA	Ph.D.	274 p-274 p	NA	Bruno 2013	Irrelevant	Excluded by TI/AB review
748	Racial disparities in treatment adherence among youth with chronic kidney disease under medicaid [illegible]	Burgardt, S.; Fenton, N.; Ko, Z.; Bickford, K.; Ashkin, E.; Weber, S.; Ferris, M.	2013	Blood Purification	35	3-Jan	170	10.1159/000345306	Burgardt 2013	Irrelevant	Excluded by TI/AB review

S4 Table. All studies identified in the literature search

749	Latino fathers	Cabrera, Natasha J.; Aldoney, Daniela; Tamis-LeMonda, Catherine S.; Cabrera, Natasha J.; Tamis-LeMonda, Catherine S.	2013	Handbook of father involvement : Multidisciplinary perspectives ., 2nd ed.	NA	NA	244-260	NA	Cabrera 2013	Irrelevant	Excluded by TI/AB review
750	Analysis of HIV testing acceptance and risk factors of an adolescent cohort using	Calderon, Y.; Chou, K.; Cowan, E.; Rhee, J. Y.; Mathew, S.; Ghosh, R.; Fettig, J.; Nickerson, J.; Leider,	2013	Sexually Transmitted Diseases	40	8	624-628	10.1097/01.OLQ.0000430800.07217.01	Calderon 2013	Irrelevant	Excluded by TI/AB review
751	Assessing health professionals‚Äô perceptions of family presence during resuscitation: A [illegible]	Chapman, Rose; Watkins, Rochelle; Bushby, Angela; Combs, Shane	2013	International emergency nursing	21	1	17-25	10.1016/j.ienj.2011.10.003	Chapman 2013	Irrelevant	Excluded by TI/AB review

S4 Table. All studies identified in the literature search

753	Public perceptions of hospital responsibilities to those presenting without medical injury or illness during a disaster	Charney, R. L.; Rebmann, T.; Esguerra, C. R.; Lai, C. W.; Dalawari, P.	2013	Journal of Emergency Medicine	45	4	578-584	10.1016/j.jemermed.2013.05.010	Charney 2013	Irrelevant	Excluded by TI/AB review
755	Health Care Expenditures Among Asian American Subgroups	Chen, J.; Vargas-Bustamante, A.; Ortega, A. N.	2013	Medical Care Research and Review	70	3	310-329	10.1177/1077558712465773	Chen 2013	Irrelevant	Excluded by TI/AB review
756	Life after sepsis therapy: A one-year follow-up study of early goal-directed therapy outcomes	Chettipally, U. K.; Reed, M. E.; Huang, J.; Rauchwerger, A. S.; Ballard, D. W.; Offerman, S. R.; Vinson, D. R.	2013	Annals of Emergency Medicine	62	4	S132-S133	10.1016/j.annemergmed.2013.07.198	Chettipally 2013	Irrelevant	Excluded by TI/AB review
757	Improvements in health care use associated with community coalitions: long-term results of the allies against asthma initiative	Clark, N. M.; Lachance, L. L.; Benedict, M. B.; Doctor, L. J.; Gilmore, L.; Kelly, C. S.; Krieger, J.; Lara, M.; Meurer, J.; Milanovich, A. F.; Nicholas, E.; Song, [illegible]	2013	American Journal of Public Health	103	6	1124-1127	10.2105/AJPH.2012.300983	Clark 2013	Irrelevant	Excluded by TI/AB review

S4 Table. All studies identified in the literature search

758	A qualitative assessment of emergency department patients' [illegible]	Cowan, E.; Leider, J.; Velastegui, L.; Wexler, J.; Velloza, J.; Calderon, Y.	2013	Academic Emergency Medicine	20	3	287-294	10.1111/acem.12090	Cowan 2013	Irrelevant	Excluded by TI/AB review
759	Maternal somatic symptoms, psychosocial correlates, and subsequent pediatric emergency department use	Dang, A. T.; Ho, M.; Kroenke, K.; Grupp-Phelan, J.	2013	Pediatric Emergency Care	29	2	170-174	10.1097/PEC.0b013e3182809a10	Dang 2013	Irrelevant	Excluded by TI/AB review
760	Providing Culturally Competent Care During Disasters: Strategies for Nurses	Danna, Denise; Bennett, Marsha J.	2013	Journal of Continuing Education in Nursing	44	4	151-153	10.3928/00220124-20130327-13	Danna 2013	Irrelevant	Excluded by TI/AB review

S4 Table. All studies identified in the literature search

761	Biological and Psychosocial Predictors of Psychological Functioning among African American Breast Cancer Survivors	Davis, Claudia Maggie	2013	NA	NA	Ph.D.	149 p-149 p	NA	Davis 2013	Irrelevant	Excluded by TI/AB review
762	Does overcrowding and health insurance type impact patient	de Araujo, P.; Khraiche, M.; Tukan, A.	2013	Health Economics Review	3	1	7-Jan	10.1186/2191-1991-3-25	deAraujo 2013	Irrelevant	Excluded by TI/AB review
763	Common lung conditions: asthma	Delzell, J. E., Jr.	2013	FP Essent	409	NA	6-Nov	NA	Delzell 2013	Irrelevant	Excluded by TI/AB review

S4 Table. All studies identified in the literature search

764	Seasonal recurrent myopericarditis	Divoky, L.; Wilford, R.	2013	Journal of General Internal Medicine	28	NA	S387-S388	NA	Divoky 2013	Irrelevant	Excluded by TI/AB review
765	Randomized controlled trial of universal postnatal nurse home visiting: Impact on emergency care	Dodge, K. A.; Goodman, W. B.; Murphy, R. A.; O'Donnell, K.; Sato, J.	2013	Pediatrics	132	SUPPL.2	S140-S146	10.1542/peds.2013-1021M	Dodge 2013	Irrelevant	Excluded by TI/AB review

S4 Table. All studies identified in the literature search

766	Differential pathways to care in first episode psychosis: Adolescents versus adults	Dominguez, M. D. G.; Fisher, H. L.; Johnson, S.; Hodes, M.	2013	European Child and Adolescent Psychiatry	22	2	S167	10.1007/s00787-013-0423-9	Dominguez 2013	Irrelevant	Excluded by TI/AB review
767	Management of migraine in a tertiary care pediatric emergency department	Eapen, A.; Agarwal, R.; Thomas, R.; Sivaswamy, L.	2013	Annals of Neurology	74	NA	S169	10.1002/ana.24069	Eapen 2013	Irrelevant	Excluded by TI/AB review
768	Lower urinary tract symptoms and falls in women: A case control study	Edwards, R.; Hunter, K.; Wagg, A.	2013	Neurourology and Urodynamics	32	6	825-826	10.1002/nau.22472	Edwards 2013	Irrelevant	Excluded by TI/AB review

S4 Table. All studies identified in the literature search

769	Health literacy and asthma outcomes among older adults	Federman, A. D.; Wisnivesky, J. P.; Sofianou, A.; Leventhal, H.; Wolf, M. S.	2013	American Journal of Respiratory and Critical Care Medicine	187	NA	NA	NA	Federman 2013	Irrelevant	Excluded by TI/AB review
770	Realtime 24/7/365 management of pediatric diabetes by novel utilization of pedi-flite, a hospital-based transport team	Franklin, B. E.; Crisler, S. C.; Shappley, R.; Armour, M. M.; McCommon, D. T.; Ferry, R. J.	2013	Endocrine Reviews	34	3	NA	NA	Franklin 2013	Irrelevant	Excluded by TI/AB review
771	Structural and Hidden Barriers to a Local Primary Health Care Infrastructure: Autonomy, Decisions About Primary Health Care, and the [illegible]	Freed, C. R.; Hansberry, S. T.; Arrieta, M. I.	2013	Social Determinants, Health Disparities and Linkages to Health and Health Care	31	NA	57-81	NA	Freed 2013	Irrelevant	Excluded by TI/AB review

S4 Table. All studies identified in the literature search

772	Unscheduled return visits to the emergency department: the impact of language	Gallagher, R. A.; Porter, S.; Monuteaux, M. C.; Stack, A. M.	2013	Pediatr Emerg Care	29	5	579-83	10.1097/PEC.0b013e31828e62f4	Gallagher 2013	Irrelevant	Excluded by TI/AB review
774	Missed Opportunities: Refusal to Confirm Reactive Rapid HIV Tests in the Emergency Department	Ganguli, I.; Collins, J. E.; Reichmann, W. M.; Losina, E.; Katz, J. N.; Arbelaez, C.; Donnell-Fink, L. A.; Walensky, R. P.	2013	PLoS One	8	1	NA	10.1371/journal.pone.0053408	Ganguli 2013	Irrelevant	Excluded by TI/AB review
775	Office-based itervention at residents clinic reduces emergency department visits for asthma for an Inner-City population	Geeti, A.; Ravel, J.; Smith, M.; Adjepong, Y.; Gupta, M.; Cloutier, M. M.	2013	American Journal of Respiratory and Critical Care Medicine	187	NA	NA	NA	Geeti 2013	Irrelevant	Excluded by TI/AB review

S4 Table. All studies identified in the literature search

776	Women's health psychology	Geller, Pamela A.; Nelson, Alexandra R.; Bonacquisti, Alexa; Nezu, Arthur M.; Nezu, Christine Maguth; Geller, Pamela A.; Weiner, [illegible]	2013	Handbook of psychology: Health psychology., Vol. 9, 2nd ed.	NA	NA	477-511	NA	Geller 2013	Irrelevant	Excluded by TI/AB review
777	Ethnic variations in pathways into early intervention	Ghali, Sharif; Fisher, Helen L.; Joyce, John; Major,	2013	The British Journal of Psychiatry	202	4	277-283	10.1192/bjp.bp.111.097865	Ghali 2013	Irrelevant	Excluded by TI/AB review
778	Healthy people 2010 leading health indicators: how children with special health care needs fared	Ghandour, R. M.; Grason, H. A.; Schempf, A. H.; Strickland, B. B.; Kogan, M. D.; Jones, J. R.; Nichols, D.	2013	American Journal of Public Health	103	6	e99-e106	10.2105/AJPH.2012.301001	Ghandour 2013	Irrelevant	Excluded by TI/AB review
779	Retention in mental health care of portuguese-speaking patients	Gon√ßalves, M.; Cook, B.; Mulvaney-Day, N.; Alegria, M.; Kinrys, G.	2013	Transcultural Psychiatry	50	1	92-107	10.1177/1363461512474622	Gon√ßalves 2013	Irrelevant	Excluded by TI/AB review
781	A population based analysis of contemporary rates of reoperation for penile prosthesis procedures	Grewal, S.; Vetter, J.; Brandes, S.; Strope, S.	2013	Journal of Urology	189	4	e688-e689	10.1016/j.juro.2013.02.3087	Grewal 2013	Irrelevant	Excluded by TI/AB review

S4 Table. All studies identified in the literature search

782	Derivation and validation of clinical prediction rules for reduced vancomycin susceptibility in Staphylococcus [illegible]	Han, J. H.; Bilker, W. B.; Edelstein, P. H.; Mascitti, K. B.; Lautenbach, E.	2013	Epidemiology and Infection	141	1	165-173	10.1017/S0950268812000295	Han 2013	Irrelevant	Excluded by TI/AB review
783	Altered Mental Status in Older Patients in the Emergency Department	Han, J. H.; Wilber, S. T.	2013	Clinics in Geriatric Medicine	29	1	101-136	10.1016/j.cger.2012.09.005	Han 2013	Irrelevant	Excluded by TI/AB review
784	Comparison of embryo transfer in egg donation recipients with transvaginal and transabdominal [illegible]	Hauzman, E.; Kohls, G.; Barrio, A.; Mart√≠nez-Salazar, J.; Iglesias, C.; Garc√≠a Velasco, J. A.	2013	Human Reproduction	28	NA	i241	10.1093/humrep/det213	Hauzman 2013	Irrelevant	Excluded by TI/AB review

S4 Table. All studies identified in the literature search

785	Follow-up patterns among children with anaphylaxis and other allergic diseases referred from an urban emergency department to [illegible]	Hemler, J.; Troger, A.; Mansoor, D. K.; Sharma, H.; Mikhail, I.	2013	Journal of Allergy and Clinical Immunology	131	2	AB201	10.1016/j.jaci.2012.12.1385	Hemler 2013	Irrelevant	Excluded by TI/AB review
786	EMS and hospital stroke care coordination not associated with	Herman, M. P.; Albright, K. C.; Monlezun, D. J.; Scullen, T.; Siegler,	2013	Annals of Neurology	74	NA	S12	10.1002/ana.24068	Herman 2013	Irrelevant	Excluded by TI/AB review
787	Treatment of stage I and II non-small cell lung cancer: Diagnosis and management of lung cancer, 3rd ed: American college of chest physicians [illegible]	Howington, J. A.; Blum, M. G.; Chang, A. C.; Balekian, A. A.; Murthy, S. C.	2013	Chest	143	5 SUPPL	e278S-e313S	10.1378/chest.12-2359	Howington 2013	Irrelevant	Excluded by TI/AB review
788	Patient race/ethnicity and quality of patient-physician communication during medical [illegible]	Johnson, Rachel L.; Roter, Debra; Powe, Neil R.; Cooper, Lisa A.; LaVeist, Thomas A.; Isaac, Lydia A.	2013	Race, ethnicity, and health: A public health reader., 2nd [illegible]	NA	NA	569-585	NA	Johnson 2013	Irrelevant	Excluded by TI/AB review

S4 Table. All studies identified in the literature search

789	Obesity and Asthma: Impact on Severity, Asthma Control, and Response to Therapy	Juel, Caroline Trunk-Black; Ulrik, Charlotte Suppli	2013	Respiratory Care	58	5	867-873	10.4187/respcare.02202	Juel 2013	Irrelevant	Excluded by TI/AB review
791	Use and perceived value of health information exchange: one public healthcare	Kaelber, D. C.; Waheed, R.; Einstadter, D.; Love, T. E.; Cebul, R. D.	2013	The American journal of managed care	19	10	SP337-SP343	NA	Kaelber 2013	Irrelevant	Excluded by TI/AB review
792	Impact of gender on patient preferences for technology-based behavioral interventions	Kim, D. J.; Choo, E. K.; Ranney, M. L.	2013	Academic Emergency Medicine	20	5	S180	10.1111/acem.12115	Kim 2013	Irrelevant	Excluded by TI/AB review

S4 Table. All studies identified in the literature search

794	Exploring health disparities in integrated communities	LaVeist, Thomas A.; LaVeist, Thomas A.; Isaac, Lydia A.	2013	Race, ethnicity, and health: A public health reader., 2nd ed.	NA	NA	419-435	NA	LaVeist 2013	Irrelevant	Excluded by TI/AB review

S4 Table. All studies identified in the literature search

795	Race, ethnicity, and health: A public health reader, 2nd ed	LaVeist, Thomas A.; Isaac, Lydia A.	2013	NA	NA	NA	NA	NA	LaVeist 2013	Irrelevant	Excluded by TI/AB review
796	Attitudes about racism, medical mistrust, and satisfaction with care among	LaVeist, Thomas A.; Nickerson, Kim J.; Bowie, Janice V.; LaVeist, Thomas A.; Isaac, Lydia A.	2013	Race, ethnicity, and health: A public health	NA	NA	541-556	NA	LaVeist 2013	Irrelevant	Excluded by TI/AB review
797	Multicenter analysis of factors associated with delayed analgesia and response to	Lema, P. C.; Radeos, M.; Kovach, C.; Corujo, O.; Ali, Z.; Franklin, J.; Ladkany, D.;	2013	Annals of Emergency Medicine	62	4	S10-S11	10.1016/j.annemergmed.2013.07.310	Lema 2013	Irrelevant	Excluded by TI/AB review
798	Inequitable access to deceased donor kidney transplantation (KTX) in a state with multiple donor service	Lewis, R.; Sankar, A.; Pittman, J.	2013	American Journal of Transplantation	13	NA	265-266	10.1111/ajt.12266	Lewis 2013	Irrelevant	Excluded by TI/AB review

S4 Table. All studies identified in the literature search

799	Race/ethnicity, the social environment, and health	Lillie-Blanton, Marsha; LaVeist, Thomas A.; LaVeist, Thomas A.; Isaac, Lydia A.	2013	Race, ethnicity, and health: A public health reader., 2nd ed.	NA	NA	439-454	NA	Lillie-Blanton 2013	Irrelevant	Excluded by TI/AB review
800	Does treatment for pain influence emergency medicine research participation?	Limkakeng, A. T.; Srinivasan, A.; Freiermuth, C.; Drake, W.; Freeman, D.; Tanabe, P.; Pietrobon, R.	2013	Academic Emergency Medicine	20	5	S226	10.1111/acem.12115	Limkakeng 2013	Irrelevant	Excluded by TI/AB review
801	Quantitative measures of estrogen receptor expression in relation to breast cancer-specific mortality risk among white women and black women	Ma, H. Y.; Lu, Y. N.; Marchbanks, P. A.; Folger, S. G.; Strom, B. L.; McDonald, J. A.; Simon, M. S.; Weiss, L. K.; Malone, K. E.; Burkman, R. T.; Sullivan-Halley, J.; Deapen, D. M.; Press, M. F.; Bernstein, L.	2013	Breast Cancer Research	15	5	NA	10.1186/bcr3486	Ma 2013	Irrelevant	Excluded by TI/AB review

S4 Table. All studies identified in the literature search

802	Emergency service use in heart failure patients on hospice	Mac Kenzie, M. A.	2013	Circulation: Cardiovascular Quality and Outcomes	6	3	NA	NA	MacKenzie 2013	Irrelevant	Excluded by TI/AB review
803	Language affects length of stay in emergency departments in Queensland public hospitals	Mahmoud, I.; Hou, X. Y.; Chu, K.; Clark, M.	2013	World Journal of Emergency Medicine	4	1	9-May	10.5847/wjem.j.1920-8642.2013.01.001	Mahmoud 2013	Irrelevant	Excluded by TI/AB review
804	Health behavior, access to care, and attitudes regarding emergency situations and the use of 911 among reach musc telestroke consults	Malek, A. M.; Adams, R. J.; Debenham, E.; Hyacinth, H. I.; Lackland, D. T.	2013	American Journal of Epidemiology	177	NA	S129	10.1093/aje/kwt103	Malek 2013	Irrelevant	Excluded by TI/AB review

S4 Table. All studies identified in the literature search

805	Doctors, have you ever heard about sleep disturbance, erectile dysfunction, and epilepsy?	Matos, Gabriela; Scorza, Fulvio A.; Cavalheiro, Esper A.; Tufik, Sergio; Andersen, Monica L.	2013	Epilepsy & Behavior	28	1	9-Aug	10.1016/j.yebeh.2013.03.021	Matos 2013	Irrelevant	Excluded by TI/AB review
807	Investigating the distribution of teacher quality by race/ethnicity and socioeconomic status of students by school in one large school district	McGlohn, Robin West	2013	NA	73	NA	NA	NA	McGlohn 2013	Irrelevant	Excluded by TI/AB review
808	Disparities in Outcomes following Admission for	McNabb-Baltar, J.; Trinh, Q. D.; Barkun, A. N.	2013	PLoS One	8	3	NA	10.1371/journal.pone.0059487	McNabb-Baltar 2013	Irrelevant	Excluded by TI/AB review
809	Development and pilot implementation of a locally developed Trauma Registry: Lessons learnt in a low-income [illegible]	Mehmood, A.; Razzak, J. A.; Kabir, S.; MacKenzie, E. J.; Hyder, A. A.	2013	BMC Emergency Medicine	13	1	NA	10.1186/1471-227X-13-4	Mehmood 2013	Irrelevant	Excluded by TI/AB review

S4 Table. All studies identified in the literature search

810	Does preexisting antiplatelet treatment influence postthrombolysis intracranial hemorrhage in	Meurer, W. J.; Kwok, H.; Skolarus, L. E.; Adelman, E. E.; Kade, A. M.; Kalbfleisch, J.; Frederiksen, S. M.;	2013	Academic Emergency Medicine	20	2	146-154	10.1111/acem.12077	Meurer 2013	Irrelevant	Excluded by TI/AB review
811	Providing adolescent sexual health care in the pediatric emergency	Miller, M. K.; Kelly, P.; Goggin, K.; O'Malley, D.; Owens, R.; Maliszewski, G. A.;	2013	Journal of Adolescent Health	52	2	S47	10.1016/j.jadohealth.2012.10.112	Miller 2013	Irrelevant	Excluded by TI/AB review
812	Unmet legal needs among emergency department patients	Miller, M. W.; Ayres, L.; Lowenstein, S.	2013	Academic Emergency Medicine	20	5	S102	10.1111/acem.12115	Miller 2013	Irrelevant	Excluded by TI/AB review
814	Use of psychopharmacologic medications in adolescents with restrictive eating disorders: Analysis of data	Monge, M. C.; Forman, S. F.; McKenzie, N. M.; Rosen, D. S.; Mammel, K. A.; Callahan, S. T.; Hehn, R.; Rome, E.	2013	Journal of Adolescent Health	52	2	S39	10.1016/j.jadohealth.2012.10.094	Monge 2013	Irrelevant	Excluded by TI/AB review
816	Feasibility of a computer-assisted alcohol SBIRT program in an urban emergency department: patient	Murphy, M. K.; Bijur, P. E.; Rosenbloom, D.; Bernstein, S. L.; Gallagher, E. J.	2013	Addiction science & clinical practice	8	1	2	10.1186/1940-0640-8-2	Murphy 2013	Irrelevant	Excluded by TI/AB review

S4 Table. All studies identified in the literature search

817	Implementation of a care transitions model for low-income older adults: A high-risk, vulnerable [illegible]	Ohuabunwa, U.; Jordan, Q.; Shah, S.; Fost, M.; Flacker, J.	2013	Journal of the American Geriatrics Society	61	6	987-992	10.1111/jgs.12276	Ohuabunwa 2013	Irrelevant	Excluded by TI/AB review
818	Assessing and treating Asian Americans: Recent advances in mental health research	Okazaki, Sumie; Ling, Ariane; Paniagua, Freddy A.; Yamada, Ann-Marie	2013	Handbook of multicultural mental health: Assessment and [illegible]	NA	NA	305-325	10.1016/B978-0-12-394420-7.00016-3	Okazaki 2013	Irrelevant	Excluded by TI/AB review
819	Parent retention, use and understanding of asthma action plans	Okelo, S. O.; Phipatanakul, W.; Bilderback, A. L.; Perzanowski, M. S.; Balcer-Whaley, S.; Matsui, E. C.	2013	American Journal of Respiratory and Critical Care Medicine	187	NA	NA	NA	Okelo 2013	Irrelevant	Excluded by TI/AB review

S4 Table. All studies identified in the literature search

820	Post-traumatic stress disorder in veterans of operation enduring freedom/operation Iraqi freedom: Review of treatment compared to evidence-based practice guidelines	Oliveira, R.; Moore, T. A.; Mascarenas, C. A.; Rogers, C.	2013	Journal of Pharmacy Practice	26	3	333	10.1177/08 971900134 87674	Oliveira 2013	Irrelevant	Excluded by TI/AB review
821	Lower health literacy is associated with poorer health status and outcomes in chronic obstructive pulmonary disease	Omachi, T. A.; Sarkar, U.; Yelin, E. H.; Blanc, P. D.; Katz, P. P.	2013	Journal of General Internal Medicine	28	1	74-81	10.1007/s11 606-012-2177-3	Omachi 2013	Irrelevant	Excluded by TI/AB review
822	Proportional Differences in Emergency Room Adult Patients with PTSD, Mood Disorders, and Anxiety for a Large Ethnically [illegible]	Onoye, Jane; Helm, Susana; Koyanagi, Chad; Fukuda, Michael; Hishinuma, Earl; Takeshita, Junji; Ona, Celia	2013	Journal of Health Care for the Poor & Underserve d	24	2	928-942	10.1353/hp u.2013.005 4	Onoye 2013	Irrelevant	Excluded by TI/AB review

S4 Table. All studies identified in the literature search

823	African american men's and women's perceptions of clinical trials research: Focusing on prostate cancer among a high- risk population in the South	Owens, O. L.; Jackson, D. D.; Thomas, T. L.; Friedman, D. B.; H√©bert, J. R.	2013	Journal of Health Care for the Poor and Underserved	24	4	1784-1800	10.1353/hpu.2013.0187	Owens 2013	Irrelevant	Excluded by TI/AB review
824	Patient survey of the rationale of using EMS services vs. Alternate transportation for medical emergencies	Patel, V.; Wall, E.; Zabar, B.; Quass, J.; Rabrich, J.	2013	Academic Emergency Medicine	20	5	S273	10.1111/acem.12115	Patel 2013	Irrelevant	Excluded by TI/AB review

S4 Table. All studies identified in the literature search

825	Racial disparities in ordering laboratory and radiology tests for pediatric patients in the emergency department	Payne, N. R.; Puumala, S. E.	2013	Pediatric Emergency Care	29	5	598-606	10.1097/PEC.0b013e31828e6489	Payne 2013	Irrelevant	Excluded by TI/AB review
826	Handbook of marriage and the family, 3rd ed	Peterson, Gary W.; Bush, Kevin R.	2013	NA	NA	NA	NA	10.1007/978-1-4614-3987-5	Peterson 2013	Irrelevant	Excluded by TI/AB review
827	A Clinical Improvement Project: Reducing Default Rates at a Fast Track Asthma Clinic	Prabhakaran, L.; Nur Adillah, A. Y.; Abisheganaden, J.; Lim, G. H.; Chee, J. W. Y.; Lee, T. W.; Sattar, F.; Liew, C. [illegible]	2013	Journal of Asthma and Allergy Educators	4	1	14-May	10.1177/2150129712452305	Prabhakaran 2013	Irrelevant	Excluded by TI/AB review

S4 Table. All studies identified in the literature search

828	Procalcitonin-guided therapy in critically ill patients with sepsis: A systematic review and meta-analysis	Prkno, A.; Wacker, C.; Brunkhorst, F. M.; Schlattmann, P.	2013	Infection, Supplement	41	1	S47	10.1007/s15010-013-0513-0	Prkno 2013	Irrelevant	Excluded by TI/AB review
829	Predicting and preventing avoidable hospital admissions: A review	Purdy, S.; Huntley, A.	2013	Journal of the Royal College of Physicians of Edinburgh	43	4	340-344	10.4997/JRCPE.2013.415	Purdy 2013	Irrelevant	Excluded by TI/AB review
830	Promoting educational equity in disadvantaged youth: The role of resilience and social-emotional learning	Reyes, Jazmin A.; Elias, Maurice J.; Parker, Sarah J.; Rosenblatt, Jennifer L.; Goldstein, Sam; Brooks, Robert B.	2013	Handbook of resilience in children., 2nd ed.	NA	NA	349-370	10.1007/978-1-4614-3661-4_20	Reyes 2013	Irrelevant	Excluded by TI/AB review

S4 Table. All studies identified in the literature search

833	Early response to intravenous fluid for children with acute migraine in the emergency department	Richer, L.; Craig, W.; Rowe, B.	2013	Cephalalgia	33	NA	25	10.1177/0333102413490487	Richer 2013	Irrelevant	Excluded by TI/AB review
834	Does a wireless incentive structure improve retention, subject satisfaction, and safety in intimate [illegible]	Rodgers, M. A.; Hanlon, A. L.; Datner, E. M.; Rhodes, K. V.	2013	Academic Emergency Medicine	20	5	S104	10.1111/acem.12115	Rodgers 2013	Irrelevant	Excluded by TI/AB review
835	Integrated model of palliative care in the emergency department	Rosenberg, M.; Rosenberg, L.	2013	Western Journal of Emergency Medicine	14	6	633-636	10.5811/westjem.2013.5.14674	Rosenberg 2013	Irrelevant	Excluded by TI/AB review
836	Individualized medication assessment and planning: optimizing medication use in [illegible]	Roth, M. T.; Ivey, J. L.; Esserman, D. A.; Crisp, G.; Kurz, J.; Weinberger, M.	2013	Pharmacotherapy	33	8	787-797	NA	Roth 2013	Irrelevant	Excluded by TI/AB review

S4 Table. All studies identified in the literature search

838	Use of the Kansas City cardiomyopathy questionnaire to assess acute heart failure patients	Sauser, K.; Spertus, J. A.; Pierchala, L.; Pang, P. S.	2013	Circulation: Cardiovascular Quality and Outcomes	6	3	NA	NA	Sauser 2013	Irrelevant	Excluded by TI/AB review
839	Disparities in access to specialized epilepsy care among people with epilepsy	Schiltz, N. K.; Koroukian, S. M.; Love, T. E.; Singer, M. E.; Kaiboriboon, K.	2013	Epilepsy Currents	13	NA	315	NA	Schiltz 2013	Irrelevant	Excluded by TI/AB review
840	Disparities in access to specialized epilepsy care	Schiltz, N. K.; Koroukian, S. M.; Singer, M. E.; Love, T. E.; Kaiboriboon, K.	2013	Epilepsy Res	107	2-Jan	172-80	10.1016/j.eplepsyres.2013.08.003	Schiltz 2013	Irrelevant	Excluded by TI/AB review
842	The relationship between emergency department length of stay and severe [illegible]	Schorr, C.; Hunter, K.; Swierczynski, P.; Schrift, D.; Torjman, M.; Zanotti-Cavazzoni, S.; [illegible]	2013	Critical Care Medicine	41	12	A270-A271	10.1097/01.ccm.0000440309.17645.85	Schorr 2013	Irrelevant	Excluded by TI/AB review

S4 Table. All studies identified in the literature search

846	Evaluating the relationship between opioid analgesics and patient satisfaction among emergency department patients	Schwartz, T. M.; Tai, M.; Babu, K. M.; Merchant, R. C.	2013	Annals of Emergency Medicine	62	4	S126-S127	10.1016/j.annemergmed.2013.07.182	Schwartz 2013	Irrelevant	Excluded by TI/AB review
847	An audit of acute dermatology referrals at a district general hospital	Shaheen, B.; Soon, C.	2013	British Journal of Dermatology	169	NA	34	10.1111/bjd.12306	Shaheen 2013	Irrelevant	Excluded by TI/AB review
848	Emergency medicine patient satisfaction with email follow-up reminders	Sharp, B. R.; Maier, P.; Juneja, N.; Fowler, J.; Hornacek, B.; Simmons, S.	2013	Annals of Emergency Medicine	62	4	S51-S52	10.1016/j.annemergmed.2013.07.423	Sharp 2013	Irrelevant	Excluded by TI/AB review

S4 Table. All studies identified in the literature search

849	Decision-making processes of patients who use the emergency department for primary care needs	Shaw, E. K.; Howard, J.; Clark, E. C.; Etz, R. S.; Arya, R.; Tallia, A. F.	2013	Journal of Health Care for the Poor and Underserved	24	3	1288-1305	10.1353/hpu.2013.0140	Shaw 2013	Irrelevant	Excluded by TI/AB review
851	Ethnic Disparities trump other risk factors in determining delay to emergency department arrival in acute ischemic stroke	Siegler, J. E.; Boehme, A. K.; Albright, K. C.; Martin-Schild, S.	2013	Ethnicity and Disease	23	1	29-34	NA	Siegler 2013	Irrelevant	Excluded by TI/AB review
854	Patient-perceived ‚Äúacceptable risk‚Äù of chest computed tomography in trauma pregnancy	Silverman, E. C.; Rodriguez, R. M.; Farner, M. C.; Patterson, K. L.; Baumann, B. M.	2013	Academic Emergency Medicine	20	5	S250-S251	10.1111/acem.12115	Silverman 2013	Irrelevant	Excluded by TI/AB review

S4 Table. All studies identified in the literature search

855	Women with inflammatory bowel disease utilize the emergency department more than men despite comparable access to outpatient gastroenterology providers	Sivaraman, A.; Al Jaser, W.; Vermani, S.; Borum, M.	2013	Inflammatory Bowel Diseases	19	NA	S63	10.1097/01.MIB.0000438775.36464.b1	Sivaraman 2013	Irrelevant	Excluded by TI/AB review
856	An Application of the Andersen Model of Health Utilization to the Understanding of the Role of Race-Concordant Doctor-Patient Relationships in [illegible]	Smith, G. H.; Scheid, T. L.	2013	Social Determinants, Health Disparities and Linkages to Health and Health Care	31	NA	187-214	NA	Smith 2013	Irrelevant	Excluded by TI/AB review

S4 Table. All studies identified in the literature search

857	Health related quality of life in Hispanic versus non-Hispanic White congestive heart failure patients and their relationship to hospital admissions and emergency department visits	Snowden, S.; Borrego, M.; Warner, T.; Nawarskas, J.; Anderson, J.	2013	Pharmacotherapy	33	10	e264	10.1002/phar.1356	Snowden 2013	Irrelevant	Excluded by TI/AB review
858	Mobile clinic in massachusetts associated with cost savings from lowering blood pressure and emergency [illegible]	Song, Z.; Hill, C.; Bennet, J.; Vavasis, A.; Oriol, N. E.	2013	Health Affairs	32	1	36-44	10.1377/hlthaff.2011.1392	Song 2013	Irrelevant	Excluded by TI/AB review
859	Older fallers attended to by an ambulance but not transported to hospital: a vulnerable population at high risk of future falls	Tiedemann, A.; Mikolaizak, A. S.; Sherrington, C.; Segin, K.; Lord, S. R.; Close, J. C.	2013	Australian and New Zealand journal of public health	37	2	179-185	NA	Tiedemann 2013	Irrelevant	Excluded by TI/AB review

S4 Table. All studies identified in the literature search

860	Characteristics and cognitions associated with hazardous drinking amongyoung adult	Truong, S.; Kesinger, M.; Charry, D.; Rubiano, A.; Suffoletto, B.	2013	Alcoholism: Clinical and Experimental Research	37	NA	147A	10.1111/acer.12162	Truong 2013	Irrelevant	Excluded by TI/AB review
861	Quality of care in the ICU from the perspective of relatives	Van Mol, M.; Bakker, E.; Rensen, A.; Menheere, I.; Verharen, L.	2013	Critical Care	17	NA	S199-S200	10.1186/cc12481	VanMol 2013	Irrelevant	Excluded by TI/AB review
862	Risk factors for homelessness among community mental health patients with severe mental illness	van Wormer, Rupert Talmage	2013	NA	74	NA	NA	NA	vanWormer 2013	Irrelevant	Excluded by TI/AB review
863	The GAy MEn Sex StudieS: Erectile dysfunction among Belgian gay men	Vansintejan, J.; Vandevoorde, J.; Devroey, D.	2013	International Journal of General Medicine	6	NA	527-534	10.2147/IJGM.S45783	Vansintejan 2013	Irrelevant	Excluded by TI/AB review

S4 Table. All studies identified in the literature search

864	Income-related inequality in health and health care utilization in Chile, 2000-2009	V√°squez, F.; Paraje, G.; Estay, M.	2013	Rev Panam Salud Publica	33	2	98-106, 2 p preceding 98	10.1590/s1020-49892013000200004	V√°squez 2013	Irrelevant	Excluded by TI/AB review
865	Nonemergent emergency department use among patients with a usual source of care	Villani, J.; Mortensen, K.	2013	Journal of the American Board of Family Medicine	26	6	680-691	10.3122/jabfm.2013.06.120327	Villani 2013	Irrelevant	Excluded by TI/AB review
866	Sickle cell disease: A quality improvement initiative for emergency department providers	Walker, Pretrescia M.	2013	NA	NA	D.N.P.	59 p-59 p	NA	Walker 2013	Irrelevant	Excluded by TI/AB review

S4 Table. All studies identified in the literature search

867	The influence of primary care provider type on adolescent visits to the emergency	Weiss, A. L.; Rucker, A. C.	2013	Journal of Adolescent Health	52	2	S45	10.1016/j.jadohealth.2012.10.107	Weiss 2013	Irrelevant	Excluded by TI/AB review
868	Factors associated with emergency department visits for asthma exacerbation	Wells, R. E.; Fitzgerald, J.; Garb, J.; Visintainer, P.; Kleppel, R.; Rothberg, M.	2013	American Journal of Respiratory and Critical Care Medicine	187	NA	NA	NA	Wells 2013	Irrelevant	Excluded by TI/AB review
869	US socioeconomic and racial differences in health: Patterns and explanations	Williams, David R.; Collins, Chiquita; LaVeist, Thomas A.; Isaac, Lydia A.	2013	Race, ethnicity, and health: A public health reader., 2nd ed.	NA	NA	375-418	NA	Williams 2013	Irrelevant	Excluded by TI/AB review
870	Racial disparity in duration of patients' visits to the emergency department: Teaching versus non-teaching hospitals	Wong, H.; Karaca, Z.	2013	Value in Health	16	3	A244-A245	10.1016/j.jval.2013.03.1245	Wong 2013	Irrelevant	Excluded by TI/AB review

S4 Table. All studies identified in the literature search

871	Trends of genital and nongenital community-acquired methicillin-resistant staphylococcus aureus infections in an urban [illegible]	Woods, D. L.; Amrute, K. V.	2013	Journal of Lower Genital Tract Disease	17	1	11-Jun	10.1097/LGT.0b013e31825677c0	Woods 2013	Irrelevant	Excluded by TI/AB review
872	Association between medication adherence report scale (MARS-5) and asthma outcomes in inner city children with [illegible]	Yang, S.; Tsoukleris, M. G.; Lewis-Land, C.; Walker, J.; Bollinger, M. E.; Butz, A.	2013	Journal of Allergy and Clinical Immunology	131	2	AB133	10.1016/j.jaci.2012.12.1144	Yang 2013	Irrelevant	Excluded by TI/AB review
873	Working with families from religious fundamentalist backgrounds	Yates, Jennifer D.; Cole, Allan Hugh, Jr.; Franklin, Cynthia; Harris, Mary Beth; Allen-Meares, Paula	2013	The school services sourcebook: A guide for school-based professionals., 2nd ed.	NA	NA	671-685	NA	Yates 2013	Irrelevant	Excluded by TI/AB review

S4 Table. All studies identified in the literature search

874	Assessments of perceived racial stereotypes, discrimination, and racism	Yoo, Hyung Chol; Pituc, Stephanie T.; Geisinger, Kurt F.; Bracken, Bruce A.; Carlson, Janet F.; Hansen, Jo-Ida C.; Kuncel, Nathan R.; Reise, Steven P.; [illegible]	2013	APA handbook of testing and assessment in psychology, Vol. 2: [illegible]	NA	NA	427-451	10.1037/14048-025	Yoo 2013	Irrelevant	Excluded by TI/AB review
875	Blacks arrive later after stroke onset than whites in chicago	Aggarwal, N.; O'Neill, K.; Beckstrom, H.; Song, S.; Hoelzel, S.; Joyce, B.; Gavras, H.; Martin, S.; [illegible]	2014	Stroke	45	NA	NA	NA	Aggarwal 2014	Irrelevant	Excluded by TI/AB review
876	Adolescents and young adults on the acute medical unit: How might we do it better?	Albon, L.; Vaughan, L.	2014	Clinical Medicine, Journal of the Royal College of Physicians of London	14	6	618-622	10.7861/clinmedicine.14-6-618	Albon 2014	Irrelevant	Excluded by TI/AB review
877	Effectiveness of the chest pain choice decision aid in emergency department patients with low- [illegible]	Anderson, R. T.; Montori, V. M.; Shah, N. D.; Ting, H. H.; Pencille, L. J.; Demers, M.; Kline, J. A.; Diercks, D. B.; [illegible]	2014	Trials	15	1	NA	10.1186/1745-6215-15-166	Anderson 2014	Irrelevant	Excluded by TI/AB review

S4 Table. All studies identified in the literature search

878	Geriatric emergency medicine service: a novel approach to an emerging trend	Argento, V.; Calder, G.; Ferrigno, R.; Skudlarska, B.	2014	Connecticut medicine	78	6	339-343	NA	Argento 2014	Irrelevant	Excluded by TI/AB review
879	How Patient Interactions With a Computer-Based Video Intervention Affect Decisions to Test for HIV	Aronson, I. D.; Rajan, S.; Marsch, L. A.; Bania, T. C.	2014	Health education & behavior : the official publication of the Society for Public Health Education	41	3	259-266	10.1177/1090198113509106	Aronson 2014	Irrelevant	Excluded by TI/AB review
880	The crisis of women and at-risk populations needing dental care in Tennessee: challenges and engagement efforts	Aubertin, M. A.; Woods, M.; Wasson, W.	2014	J Tenn Dent Assoc	94	1	38-45; quiz 46-7	NA	Aubertin 2014	Irrelevant	Excluded by TI/AB review

S4 Table. All studies identified in the literature search

881	Association between emergency department use and the discrimination perceived by [illegible]	Baggio, S.; Bodenmann, P.; Daeppen, J. B.; Burnand, B.; Hugli, O.; Ruggeri, O.; Wasserfallen, J. B.; Moschetti, K.; [illegible]	2014	Journal of General Internal Medicine	29	NA	S31	NA	Baggio 2014	Irrelevant	Excluded by TI/AB review
882	Risk factors associated with antihypertensive medication nonadherence in a statewide medicaid population	Bailey, J. E.; Hajjar, M.; Shoib, B.; Tang, J.; Ray, M. M.; Wan, J. Y.	2014	American Journal of the Medical Sciences	348	5	410-415	10.1097/MAJ.0b013e31825ce50f	Bailey 2014	Irrelevant	Excluded by TI/AB review

S4 Table. All studies identified in the literature search

883	Factors associated with a willingness to accept rapid HIV testing in an urban emergency department	Bamford, L.; Ellenberg, J. H.; Hines, J.; Vinnard, C.; Jasani, A.; Gross, R.	2014	AIDS and behavior	18	2	250-253	10.1007/s10461-013-0452-x	Bamford 2014	Irrelevant	Excluded by TI/AB review
884	Disparate patterns of hospitalisation reflect unmet needs and persistent ethnic inequalities in mental health care: The Scottish health [illegible]	Bansal, Narinder; Bhopal, Raj; Netto, Gina; Lyons, Donald; Steiner, Markus F. C.; Sashidharan, Sashi P.	2014	Ethnicity & health	19	2	217-239	10.1080/13557858.2013.814764	Bansal 2014	Irrelevant	Excluded by TI/AB review

S4 Table. All studies identified in the literature search

885	Cardiology clinic follow-up did not decrease return visits to the ED for chest pain patients	Barksdale, A.; Hackman, J.; Bonham, A.; Gratton, M.	2014	American Journal of Emergency Medicine	32	10	1208-1211	10.1016/j.ajem.2014.07.002	Barksdale 2014	Irrelevant	Excluded by TI/AB review
886	Emergency department triage pain protocol reduces time to receiving analgesics in patients with painful conditions	Barksdale, A. N.; Hackman, J.; Williams, K.; Gratton, M.	2014	Academic Emergency Medicine	21	5	S217	10.1111/acem.12365	Barksdale 2014	Irrelevant	Excluded by TI/AB review
887	Initial choice of oral glucose-lowering medication for diabetes mellitus: [illegible]	Berkowitz, S. A.; Krumme, A. A.; Avorn, J.; Brennan, T.; Matlin, O. S.; Spettell, C. M.; [illegible]	2014	JAMA Internal Medicine	174	12	1955-1962	10.1001/jamainternmed.2014.5294	Berkowitz 2014	Irrelevant	Excluded by TI/AB review

S4 Table. All studies identified in the literature search

888	Pain management: Association with patient satisfaction among Emergency Department patients	Bhakta, H. C.; Marco, C. A.	2014	Journal of Emergency Medicine	46	4	456-464	10.1016/j.jemermed.2013.04.018	Bhakta 2014	Irrelevant	Excluded by TI/AB review
889	Impact of learners on emergency medicine attending physician productivity	Bhat, R.; Dubin, J.; Maloy, K.	2014	Western Journal of Emergency Medicine	15	1	41-44	10.5811/westjem.2013.7.15882	Bhat 2014	Irrelevant	Excluded by TI/AB review
890	A remote monitoring and telephone nurse coaching intervention to reduce readmissions among patients with heart failure: Study protocol for the Better Effectiveness	Black, J. T.; Romano, P. S.; Sadeghi, B.; Auerbach, A. D.; Ganiats, T. G.; Greenfield, S.; Kaplan, S. H.; Ong, M. K.	2014	Trials	15	1	NA	10.1186/1745-6215-15-124	Black 2014	Irrelevant	Excluded by TI/AB review

S4 Table. All studies identified in the literature search

891	Evaluation of a community-wide stroke preparedness intervention: Results of the ASPIRE study	Boden-Albala, B.; Edwards, D. F.; Wing, J. J.; Gibbons, M. C.; Hsia, A.; Kidwell, C. S.	2014	Stroke	45	NA	NA	NA	Boden-Albala 2014	Irrelevant	Excluded by TI/AB review
892	Emergency physician adherence to a chest pain risk stratification decision aid	Bringolf, J.; Elliott, S. B.; Miller, C. D.; Riley, R. F.; Hiestand, B. C.; Mahler, S. A.	2014	Annals of Emergency Medicine	64	4	S16	10.1016/j.annemergmed.2014.07.069	Bringolf 2014	Irrelevant	Excluded by TI/AB review
893	Improving outcomes for underserved adolescents with asthma	Britto, M. T.; Vockell, A. L. B.; Munafo, J. K.; Schoettker, P. J.; Wimberg, J. A.; Pruett, R.; Yi, M. S.; Byczkowski, T. L.	2014	Pediatrics	133	2	e418-e427	10.1542/peds.2013-0684	Britto 2014	Irrelevant	Excluded by TI/AB review
894	Feasibility and acute care utilization outcomes of a post-acute transitional telemonitoring program for underserved heart [illegible]	Broad, J.; Davis, C.; Bender, M.; Smith, T.	2014	Journal of Cardiac Failure	20	8	S116	10.1016/j.cardfail.2014.06.328	Broad 2014	Irrelevant	Excluded by TI/AB review

S4 Table. All studies identified in the literature search

895	Web-based emergency department patient satisfaction surveys may introduce potential [illegible]	Broadwater-Hollifield, C.; Fair, J.; Podolsky, S.; Carey, J.; Vlasic, K.; Stephen, R.; Madsen, T.; Mallin, M.	2014	Journal of Emergency Medicine	46	3	404-409	10.1016/j.jemermed.2013.08.077	Broadwater-Hollifield 2014	Irrelevant	Excluded by TI/AB review
896	Perception of recovery after pediatric mild traumatic brain injury is influenced by the "good old days" [illegible]	Brooks, B. L.; Kadoura, B.; Turley, B.; Crawford, S.; Mikrogianakis, A.; Barlow, K. M.	2014	Archives of Clinical Neuropsychology	29	2	186-193	10.1093/arclin/act083	Brooks 2014	Irrelevant	Excluded by TI/AB review
897	The management of pregnancy-related nausea and vomiting in the Emergency Department: Are [illegible]	Buchanan, G.; Nicholson, T.	2014	BJOG: An International Journal of Obstetrics and Gynaecolog	121	NA	15	10.1111/1471-0528.13165	Buchanan 2014	Irrelevant	Excluded by TI/AB review
898	Impact of text message medication reminders on medication adherence and [illegible]	Buis, L.; Brody, A.; Mango, L. M.; Dawood, R.; Schwiebert, L.; Yarandi, H.; Artinian, N.; Levy, P. [illegible]	2014	Circulation	130	NA	NA	NA	Buis 2014	Irrelevant	Excluded by TI/AB review
899	Feasibility and evaluation of a pilot community health worker intervention to reduce hospital [illegible]	Burns, M. E.; Galbraith, A. A.; Ross-Degnan, D.; Balaban, R. B.	2014	International Journal for Quality in Health Care	26	4	358-365	10.1093/intqhc/mzu046	Burns 2014	Irrelevant	Excluded by TI/AB review

S4 Table. All studies identified in the literature search

900	Improving preventive care in high risk children with asthma: Lessons learned	Butz, A. M.; Halterman, J.; Bellin, M.; Kub, J.; Tsoukleris, M.; Frick, K. D.; Thompson, R. E.; Land, C.; Bollinger, M. E.	2014	Journal of Asthma	51	5	498-507	10.3109/02770903.2014.892608	Butz 2014	Irrelevant	Excluded by TI/AB review
901	Review of The Latino threat: Constructing immigrants, citizens and the nation, 2nd ed	Carballeira, Catherine	2014	Journal of Sociology and Social Welfare	41	1	171-173	NA	Carballeira 2014	Irrelevant	Excluded by TI/AB review

S4 Table. All studies identified in the literature search

902	Perceptions of Australian emergency staff towards patients presenting with deliberate self-poisoning: a [illegible]	Chapman, R.; Martin, C.	2014	International emergency nursing	22	3	140-145	10.1016/j.ienj.2014.03.002	Chapman 2014	Irrelevant	Excluded by TI/AB review
903	Evaluation of staff cultural awareness before and after attending cultural awareness training [illegible]	Chapman, R.; Martin, C.; Smith, T.	2014	International emergency nursing	22	4	179-184	10.1016/j.ienj.2013.11.001	Chapman 2014	Irrelevant	Excluded by TI/AB review
904	Emotional, physical and sexual abuse: Impact in children and social minorities	Corona, Giovanni; Jannini, Emmanuele A.; Maggi, Mario	2014	NA	NA	NA	NA	10.1007/978-3-319-06787-2	Corona 2014	Irrelevant	Excluded by TI/AB review

S4 Table. All studies identified in the literature search

905	Ultrasound use in Australasian emergency departments: A survey of Australasian College for Emergency Medicine Fellows and Trainees	Craig, S.; Egerton-Warburton, D.; Mellett, T.	2014	EMA - Emergency Medicine Australasia	26	3	268-273	10.1111/1742-6723.12231	Craig 2014	Irrelevant	Excluded by TI/AB review
906	Randomized controlled trial of cognitive behavioral therapy and acceptance and commitment [illegible]	Craske, M. G.; Niles, A. N.; Burklund, L. J.; Wolitzky-Taylor, K. B.; Vilardaga, J. C. P.; Arch, J. J.; Saxbe, D. E.; [illegible]	2014	Journal of Consulting and Clinical Psychology	82	6	1034-1048	10.1037/a0037212	Craske 2014	Irrelevant	Excluded by TI/AB review
907	Prospective, 10-year evaluation of the impact of Hispanic ethnicity on pain management [illegible]	Craven, P.; Cinar, O.; Fosnocht, D.; Carey, J.; Carey, A.; Rogers, L.; Vlasic, K.; Madsen, T.	2014	The American journal of emergency medicine	32	9	1055-1059	10.1016/j.ajem.2014.06.026	Craven 2014	Irrelevant	Excluded by TI/AB review
909	Ed assault-injured youth: Two-year prospective cohort study of violent injury & mortality	Cunningham, R.; Carter, P.; Zimmerman, M.; Blow, F.; Booth, B.; Walton, M.	2014	Academic Emergency Medicine	21	5	S145	10.1111/acem.12365	Cunningham 2014	Irrelevant	Excluded by TI/AB review

S4 Table. All studies identified in the literature search

910	Parental asthma education and risks for nonadherence to pediatric asthma treatments	Delgado, E. M.; Cho, C. S.; Gildengorin, G.; Leibovich, S. A.; Morris, C. R.	2014	Pediatric Emergency Care	30	11	782-787	10.1097/PEC.0000000000000259	Delgado 2014	Irrelevant	Excluded by TI/AB review
911	The effectiveness of text messaging programs on adherence to treatment regimens among adults aged 18 to 45 years [illegible]	DiBello, K.; Boyar, K.; Abrenica, S.; Worral, P. S.	2014	JBI Database of Systematic Reviews and Implementation Reports	12	4	485-532	10.11124/jbisrir-2014-1413	DiBello 2014	Irrelevant	Excluded by TI/AB review
912	"rewarding and challenging at the same time": Emergency medicine residents' experiences caring for patients who are homeless	Doran, K. M.; Curry, L. A.; Vashi, A. A.; Platis, S.; Rowe, M.; Gang, M.; Vaca, F. E.	2014	Academic Emergency Medicine	21	6	673-679	10.1111/acem.12388	Doran 2014	Irrelevant	Excluded by TI/AB review

S4 Table. All studies identified in the literature search

913	Concomitant alcohol intoxication delays antibiotic treatment of sepsis in adult emergency department patients	Dryer, A.; Kopelman, Z.; Dattilo, A.; Ward, M. F.; Schneider, S.; D'Amore, J.	2014	Annals of Emergency Medicine	64	4	S34	10.1016/j.annemergmed.2014.07.119	Dryer 2014	Irrelevant	Excluded by TI/AB review
914	Management of pediatric migraine in a tertiary care versus community based emergency department: An observational pilot study	Eapen, A.; Sivaswamy, L.; Agarwal, R.; Thomas, R.	2014	Pediatric Neurology	50	2	164-170	10.1016/j.pediatrneurol.2013.10.005	Eapen 2014	Irrelevant	Excluded by TI/AB review
915	The Care Transitions Innovation (C-TraIn) for Socioeconomically Disadvantaged Adults: Results of [illegible]	Englander, H.; Michaels, L.; Chan, B.; Kansagara, D.	2014	Journal of General Internal Medicine	29	11	1460-1467	10.1007/s11606-014-2903-0	Englander 2014	Irrelevant	Excluded by TI/AB review

S4 Table. All studies identified in the literature search

916	Event-level analysis of antecedents for youth violence: Comparison of dating violence with non-dating [illegible]	Epstein-Ngo, Q. M.; Walton, M. A.; Chermack, S. T.; Blow, F. C.; Zimmerman, M. A.; Cunningham, R. M.	2014	Addictive Behaviors	39	1	350-353	10.1016/j.addbeh.2013.10.015	Epstein-Ngo 2014	Irrelevant	Excluded by TI/AB review
917	Multiculturalism and diversity in clinical supervision: A competency-based approach	Falender, Carol A.; Shafranske, Edward P.; Falicov, Celia J.	2014	NA	NA	NA	NA	10.1037/14370-000	Falender 2014	Irrelevant	Excluded by TI/AB review
918	Identifying barriers to timely care for febrile children with sickle cell disease and implementing an educational intervention targeting families [illegible]	Faulk, K.; McKinney, C.; Rose, L.; Rose, S.; Gillespie, A.; Coughlin, R.; Hilden, J.; Nuss, R.; Brown, A.	2014	Pediatric Blood and Cancer	61	NA	S26-S27	10.1002/pbc.25032	Faulk 2014	Irrelevant	Excluded by TI/AB review

S4 Table. All studies identified in the literature search

919	Characteristics and outcomes of older adults with early versus late onset asthma	Federman, A.; Herscher, M.; Ray, M.; Busse, P. J.; Wolf, M. S.; Wisnivesky, J. P.	2014	American Journal of Respiratory and Critical Care Medicine	189	NA	NA	NA	Federman 2014	Irrelevant	Excluded by TI/AB review

S4 Table. All studies identified in the literature search

920	Asthma outcomes are poor among older adults with low health literacy	Federman, A. D.; Wolf, M. S.; Sofianou, A.; O'Conor, R.; Martynenko, M.; Halm, E. A.; Leventhal, H.; Wisnivesky, J. P.	2014	Journal of Asthma	51	2	162-167	10.3109/02770903.2013.852202	Federman 2014	Irrelevant	Excluded by TI/AB review
921	Emergency care nurses: certification, empowerment, and work-related variables	Fitzpatrick, J. J.; Campo, T. M.; Gacki-Smith, J.	2014	Journal of emergency nursing: JEN : official publication [illegible]	40	2	e37-e43	10.1016/j.jen.2013.01.021	Fitzpatrick 2014	Irrelevant	Excluded by TI/AB review
923	Attitudes Toward Patients With Sickle Cell Disease in a Multicenter Sample of Emergency Department Providers	Freiermuth, Caroline E.; Haywood Jr, Carlton; Silva, Susan; Cline, David M.; Kayle, Mariam; Sullivan, Dori; Thornton, Victoria; Tanabe, Paula	2014	Advanced emergency nursing journal	36	4	335-347	10.1097/TME.0000000000000036	Freiermuth 2014	Irrelevant	Excluded by TI/AB review

S4 Table. All studies identified in the literature search

924	Influence of physician sex on the ability of patients to identify their emergency department attending	Golden, S. K.; Sharp, B.; Patterson, B.; Hamedani, A.	2014	Annals of Emergency Medicine	64	4	S88-S89	10.1016/j.annemergmed.2014.07.275	Golden 2014	Irrelevant	Excluded by TI/AB review
925	Developing and implementing an interdisciplinary transition of care program utilizing medication therapy management and the patient-centered medical home model along the U.S.-Mexico	Gomez, J.; Padilla, M.	2014	Journal of the American Pharmacists Association	54	2	e137-e138	10.1331/JAPhA.2014.14511	Gomez 2014	Irrelevant	Excluded by TI/AB review
926	Impact of substance abuse on timely treatment with rt-PA: A population-based study	Goodall, E. B.; Kimmel, P. L.; Sucharew, H.; Alwell, K.; Moomaw, C. J.; De Los Rios La Rosa, F.; Woo, D.; Flaherty, M.; Khatri, P.; Adeoye, O.; Ferioli, S.; Mackey, J.; Martini, S.;	2014	Stroke	45	NA	NA	NA	Goodall 2014	Irrelevant	Excluded by TI/AB review

S4 Table. All studies identified in the literature search

927	Lessons from sexual and reproductive health voucher program design and function: a comprehensive review	Grainger, C.; Gorter, A.; Okal, J.; Bellows, B.	2014	Int J Equity Health	13	NA	33	10.1186/1475-9276-13-33	Grainger 2014	Irrelevant	Excluded by TI/AB review
929	Statewide collaboration in rural stemi system development in resource limited environments- where you live shouldn't determine if you live: North Dakota mission: Lifeline	Haldis, T.; Sather, J.; Reddy, K.; Oatfield, R.; Almanaseer, Y.; AbouFakher, R.; Cook, M.; Moe, P.	2014	Circulation: Cardiovascular Quality and Outcomes	7	NA	NA	NA	Haldis 2014	Irrelevant	Excluded by TI/AB review
930	The relationship between a chief complaint of "altered mental status" and [illegible]	Han, J. H.; Schnelle, J. F.; Ely, E. W.	2014	Academic Emergency Medicine	21	8	937-940	10.1111/acem.12436	Han 2014	Irrelevant	Excluded by TI/AB review

S4 Table. All studies identified in the literature search

931	Risk of rehospitalization among elders with limited function and social support	Hansen, L. O.; Williams, M. V.	2014	Journal of the American Geriatrics Society	62	NA	S120	10.1111/jgs.12870	Hansen 2014	Irrelevant	Excluded by TI/AB review
932	Social issues in China: Gender, ethnicity, labor, and the environment	Hao, Zhidong; Chen, Sheying	2014	International perspectives on social policy, administration, and practice	NA	NA	NA	10.1007/978-1-4614-2224-2	Hao 2014	Irrelevant	Excluded by TI/AB review
933	The real-life perception of efficacy, attitude, satisfaction and safety of vardenafil therapy (REPEAT): A prospective, non-interventional, observational study	Hartmann, U.; Hanisch, J. U.; Mattern, A.	2014	Aging Male	17	2	117-124	10.3109/13685538.2013.873783	Hartmann 2014	Irrelevant	Excluded by TI/AB review

S4 Table. All studies identified in the literature search

934	Beliefs regarding mammography screening among women visiting the emergency department for nonurgent care	Hatcher-Keller, J.; Rayens, M. K.; Dignan, M.; Schoenberg, N.; Allison, P.	2014	Journal of emergency nursing: JEN : official publication of the Emergency Department Nurses Association	40	2	e27-e35	10.1016/j.jen.2013.01.015	Hatcher-Keller 2014	Irrelevant	Excluded by TI/AB review
936	Emergency medicine physician attitudes toward	Hill, M.; Okugo, G.	2014	Human Vaccines and	10	9	2551-2556	10.4161/21645515.2014.970933	Hill 2014	Irrelevant	Excluded by TI/AB review

S4 Table. All studies identified in the literature search

937	Review of Multicultural children's literature: Through the eyes of many children, 4th ed	Huang, Qiaoya	2014	International Journal of Bilingual Education and Bilingualis	17	6	748-752	10.1080/13670050.2014.909111	Huang 2014	Irrelevant	Excluded by TI/AB review
938	Which features of primary care affect unscheduled secondary care use? A systematic review	Huntley, A.; Lasserson, D.; Wye, L.; Morris, R.; Checkland, K.; England, H.; Salisbury, C.; Purdy, S.	2014	BMJ Open	4	5	NA	10.1136/bmjopen-2013-004746	Huntley 2014	Irrelevant	Excluded by TI/AB review

S4 Table. All studies identified in the literature search

939	Differences in depressive symptoms between Korean and American outpatients with major depressive disorder	Jeon, H. J.; Walker, R. S.; Inamori, A.; Hong, J. P.; Cho, M. J.; Baer, L.; Clain, A.; Fava, M.; Mischoulon, D.	2014	International Clinical Psychopharmacology	29	3	150-156	10.1097/YIC.0000000000000019	Jeon 2014	Irrelevant	Excluded by TI/AB review

S4 Table. All studies identified in the literature search

940	Differences in nursing practice environment among US acute care unit types: A descriptive study	JiSun, Choi; Boyle, Diane K.	2014	International Journal of Nursing Studies	51	11	1441-1449	10.1016/j.ijnurstu.2014.03.001	JiSun 2014	Irrelevant	Excluded by TI/AB review
941	High density lipoprotein levels in blacks presenting with acute coronary syndromes	Kemeyou, L.; Dodani, S.; McCurdy, K.; Joseph, A.; Kraemer, D.; Smotherman, C.; Velarde, G. P.	2014	Journal of the American College of Cardiology	63	12	A64	10.1016/S0735-1097(14)60064-4	Kemeyou 2014	Irrelevant	Excluded by TI/AB review
942	Impact of gender on patient preferences for technology-based behavioral interventions	Kim, D. J.; Choo, E. K.; Ranney, M. L.	2014	Western Journal of Emergency Medicine	15	5	593-599	10.5811/westjem.2014.4.21448	Kim 2014	Irrelevant	Excluded by TI/AB review

S4 Table. All studies identified in the literature search

943	Clinical predictors of severe exacerbations in patients with COPD	Krahnke, J. S.; Zhao, H.; Kim, V.; Marchetti, N.; Foreman, M. G.; Make, B. J.; Criner, G. J.	2014	American Journal of Respiratory and Critical Care Medicine	189	NA	NA	NA	Krahnke 2014	Irrelevant	Excluded by TI/AB review
944	Clinical factors associated with frequent exacerbations in mild to moderate COPD	Krahnke, J. S.; Zhao, H.; Kim, V.; Marchetti, N.; Foreman, M. G.; Make, B. J.; Criner, G. J.	2014	American Journal of Respiratory and Critical Care Medicine	189	NA	NA	NA	Krahnke 2014	Irrelevant	Excluded by TI/AB review
945	Chronic obstructive pulmonary disease illness and medication beliefs are associated with [illegible]	Krauskopf, K.; Federman, A. D.; Kale, M. S.; Sigel, K. M.; Martynenko, M.; O'Conor, R.; Wolf, M. S.; Leventhal, H.; [illegible]	2014	American Journal of Respiratory and Critical Care Medicine	189	NA	NA	NA	Krauskopf 2014	Irrelevant	Excluded by TI/AB review
946	Applying new strategies for the national adaptation, updating, and dissemination of trustworthy guidelines: Results [illegible]	Kristiansen, A.; Brandt, L.; Agoritsas, T.; Akl, E. A.; Berge, E.; Jacobsen, A. F.; Granan, L. P.; Halvorsen, S.; Guyatt, G.; Vandvik, P. O.	2014	Chest	146	3	735-761	10.1378/chest.13-2993	Kristiansen 2014	Irrelevant	Excluded by TI/AB review
947	Comparison of two prehospital systems that triage	Krumperman, K.; Weiss, S.; Lynne, F.	2014	Academic Emergency Medicine	21	5	S79	10.1111/acem.12365	Krumperman 2014	Irrelevant	Excluded by TI/AB review

S4 Table. All studies identified in the literature search

948	Public Emergency Room Overcrowding in the Era of Mass Imprisonment	Lara-Millan, A.	2014	American Sociological Review	79	5	866-887	10.1177/00031224145 49552	Lara-Millan 2014	Irrelevant	Excluded by TI/AB review
949	Commentary on: Disparities in patients presenting to the emergency department with potential acute coronary syndrome: It matters if you are Black or White	Lefler, L. L.	2014	Heart and Lung: Journal of Acute and Critical Care	43	4	265-266	10.1016/j.hrtlng.2014.05.007	Lefler 2014	Irrelevant	Excluded by TI/AB review
950	Geospatial relationships of air pollution and acute asthma events across the Detroit-Windsor [illegible]	Lemke, L. D.; Lamerato, L. E.; Xu, X. H.; Booze, J. C.; Reiners, J. J.; Raymond, D. M.; Villeneuve, P. J.; [illegible]	2014	Journal of Exposure Science and Environmental Epidemiolo	24	4	346-357	10.1038/jes.2013.78	Lemke 2014	Irrelevant	Excluded by TI/AB review

S4 Table. All studies identified in the literature search

951	A collaborative model for physician practices and home care agencies	Li, I.; Jurkovitz, C.; Laughery, T.; Zhang, Z.; Bucher, G.; Hughes, V.; Bittner-Fagan, H.	2014	Journal of the American Geriatrics Society	62	NA	S226	10.1111/jgs.12870	Li 2014	Irrelevant	Excluded by TI/AB review
952	Patient and provider perceptions of why patients seek care in emergency departments	Lobachova, L.; Brown, D. F. M.; Sinclair, J.; Chang, Y.; Thielker, K. Z.; Nagurney, J. T.	2014	Journal of Emergency Medicine	46	1	104-112	10.1016/j.jemermed.2013.04.063	Lobachova 2014	Irrelevant	Excluded by TI/AB review
953	Intensive care unit admission from the acute care floor: Is it associated with family or nurse perceptions of lower quality of [illegible]	Long, A. C.; Engelberg, R. A.; Kross, E. K.; Nielsen, E. L.; Back, A. L.; Downey, L.; Curtis, J. R.	2014	American Journal of Respiratory and Critical Care Medicine	189	NA	NA	NA	Long 2014	Irrelevant	Excluded by TI/AB review

S4 Table. All studies identified in the literature search

954	The disparity study: Factors associated with time to antibiotics in the surviving sepsis campaign database	Madsen, T. E.; Napoli, A. M.	2014	Academic Emergency Medicine	21	5	S280	10.1111/acem.12365	Madsen 2014	Irrelevant	Excluded by TI/AB review
955	The DISPARITY-II study: Delays to antibiotic administration in women with severe sepsis or [illegible]	Madsen, T. E.; Napoli, A. M.	2014	Academic Emergency Medicine	21	12	1499-1502	10.1111/acem.12546	Madsen 2014	Irrelevant	Excluded by TI/AB review
957	The DISPARITY-II Study: Delays to Antibiotic Administration in Women With Severe Sepsis or [illegible]	Madsen, Tracy E.; Napoli, Anthony M.; Zehtabchi, Shariar	2014	Academic Emergency Medicine	21	12	1499-1502	10.1111/acem.12546	Madsen 2014	Irrelevant	Excluded by TI/AB review
958	Diabetes to Go: Knowledge- and Competency-Based Hospital Survival Skills Diabetes [illegible]	Magee, M. F.; Khan, N. H.; Desale, S.; Nassar, C. M.	2014	The Diabetes educator	40	3	344-350	NA	Magee 2014	Irrelevant	Excluded by TI/AB review

S4 Table. All studies identified in the literature search

959	The acceptability of incorporating a youth smoking prevention intervention in the pediatric emergency department	Mahabee-Gittens, E. M.; Chen, C.; Huang, B.; Gordon, J. S.	2014	Journal of Health Care for the Poor and Underserved	25	2	787-800	10.1353/hpu.2014.0091	Mahabee-Gittens 2014	Irrelevant	Excluded by TI/AB review
960	Satisfaction with emergency department service among non-English-speaking background patients	Mahmoud, I.; Hou, X. Y.; Chu, K.; Clark, M.; Eley, R.	2014	EMA - Emergency Medicine Australasia	26	3	256-261	10.1111/1742-6723.12214	Mahmoud 2014	Irrelevant	Excluded by TI/AB review
961	Patient Awareness and Perception of Stroke Symptoms and the Use of 911	Malek, A. M.; Adams, R. J.; Debenham, E.; Boan, A. D.; Kazley, A. S.; Hyacinth, H. I.; Voeks, J. H.; Lackland, D. T.	2014	Journal of Stroke & Cerebrovascular Diseases	23	9	2362-2371	10.1016/j.jstrokecerebrovasdis.2014.05.011	Malek 2014	Irrelevant	Excluded by TI/AB review

S4 Table. All studies identified in the literature search

962	Enrollment in a patient-centered medical home modifies the relationship between adherence [illegible]	McNaughton, C.; Kripalani, S.; Rothman, R.; Storrow, A.; Wallston, K.; Roumie, C.	2014	Academic Emergency Medicine	21	5	S151	10.1111/acem.12365	McNaughton 2014	Irrelevant	Excluded by TI/AB review
963	The relationship between health literacy and health outcomes: A mixed-methods study of African- [illegible]	Melton, Courtnee Erin	2014	NA	75	NA	NA	NA	Melton 2014	Irrelevant	Excluded by TI/AB review
964	The relation between health insurance and health care disparities among adults with disabilities	Miller, N. A.; Kirk, A.; Kaiser, M. J.; Glos, L.	2014	NA	104	(Miller N.A.) Nancy A. Miller, Adele [illegible]	e85-93	10.2105/AJPH.2013.301478	Miller 2014	Irrelevant	Excluded by TI/AB review
968	Implementation of an acute venous thromboembolism clinical pathway reduces healthcare utilization and mitigates health [illegible]	Misky, G. J.; Carlson, T.; Thompson, E.; Trujillo, T.; Nordenholz, K.	2014	Journal of Hospital Medicine	9	7	430-435	10.1002/jhm.2186	Misky 2014	Irrelevant	Excluded by TI/AB review

S4 Table. All studies identified in the literature search

969	Nurses,Äô Knowledge and Attitudes Toward Pain in the Emergency Department	Moceri, Joane T.; Drevdahl, Denise J.	2014	JEN: Journal of Emergency Nursing	40	1	12-Jun	10.1016/j.jen.2012.04.014	Moceri 2014	Irrelevant	Excluded by TI/AB review
972	Consensus document of ACEMC, CISMEL, SIBioC and SIMeL for use [illegible]	Morelli, B.	2014	Clinical Chemistry and Laboratory Medicine	52	11	eA408-eA409	10.1515/cclm-2014-0902	Morelli 2014	Irrelevant	Excluded by TI/AB review
973	Consensus document of acemc, cismel, sibioc and simel for use of d-dimer in patiens with suspected venous [illegible]	Morelli, B.	2014	Biochimica Clinica	38	5	424-425	NA	Morelli 2014	Irrelevant	Excluded by TI/AB review
974	A transitional care model for low-income older adults does not reduce readmission rates	Nadash, P.	2014	Evidence-based nursing	17	3	98	10.1136/eb-2013-101508	Nadash 2014	Irrelevant	Excluded by TI/AB review

S4 Table. All studies identified in the literature search

975	Association between patients and emergency department operational characteristics and patient satisfaction scores in a pediatric population	Nichol, J. R.; Fu, R.; French, K.; Momberger, J.; Handel, D. A.	2014	Academic Emergency Medicine	21	5	S291-S292	10.1111/acem.12365	Nichol 2014	Irrelevant	Excluded by TI/AB review
976	Validation of parental reports of asthma trajectory, burden, and risk by using the pediatric asthma control and communication instrument	Okelo, S. O.; Eakin, M. N.; Riekert, K. A.; Teodoro, A. P.; Bilderback, A. L.; Thompson, D. A.; Loiaza-Martinez, A.; Rand, C. S.; Thyne, S.; Diette, G. B.; Patino, C. M.	2014	Journal of Allergy and Clinical Immunology: In Practice	2	2	186-192.e7	10.1016/j.jaip.2013.10.005	Okelo 2014	Irrelevant	Excluded by TI/AB review

S4 Table. All studies identified in the literature search

977	An analysis of the effect of race and sex on the reimbursement of emergency physician professional [illegible]	Onyekwere, U.; O'Neill, J. M.; Hegde, G. G.; Shang, J.; Kekre, S.; Campbell, T. P.; Venkat, A.	2014	Academic Emergency Medicine	21	5	S19	10.1111/acem.12365	Onyekwere 2014	Irrelevant	Excluded by TI/AB review
978	Emergency department length of stay: Accuracy of patient estimates	Parker, B. T.; Marco, C.	2014	Western Journal of Emergency Medicine	15	2	170-175	10.5811/westjem.2013.9.15816	Parker 2014	Irrelevant	Excluded by TI/AB review
979	Patient perceptions of asthma-related financial burden: Public vs private health insurance in the United States	Patel, M. R.; Caldwell, C. H.; Song, P. X. K.; Wheeler, J. R. C.	2014	Annals of Allergy, Asthma and Immunology	113	4	398-403	10.1016/j.anai.2014.07.004	Patel 2014	Irrelevant	Excluded by TI/AB review

S4 Table. All studies identified in the literature search

980	Risk factors and outcomes associated with patient perceptions of asthma-related financial burden: [illegible]	Patel, M. R.; Wheeler, J. R. C.; Caldwell, C. H.; Song, P. X. K.; Clark, N.	2014	American Journal of Respiratory and Critical Care Medicine	189	NA	NA	NA	Patel 2014	Irrelevant	Excluded by TI/AB review
981	Gender differences in patient-described pain, stress, and anxiety among patients undergoing treatment for painful conditions in the emergency [illegible]	Patel, R.; Biros, M. H.; Moore, J.; Miner, J. R.	2014	Academic Emergency Medicine	21	12	1478-1484	10.1111/acem.12543	Patel 2014	Irrelevant	Excluded by TI/AB review
982	Gender Differences in Patient-described Pain, Stress, and Anxiety Among Patients [illegible]	Patel, Roma; Biros, Michelle H.; Moore, Johanna; Miner, James R.; Choo, Esther	2014	Academic Emergency Medicine	21	12	1478-1484	10.1111/acem.12543	Patel 2014	Irrelevant	Excluded by TI/AB review
983	The association of gender with pain measurements	Patel, R.; Miner, J. R.	2014	Academic Emergency Medicine	21	5	S65-S66	10.1111/acem.12365	Patel 2014	Irrelevant	Excluded by TI/AB review

S4 Table. All studies identified in the literature search

984	Inpatient hospital outcomes following injury in Suriname: lessons for prevention	Pracht, E.	2014	Glob Health Promot	21	1	29-39	10.1177/17579759135 09655	Pracht 2014	Irrelevant	Excluded by TI/AB review
988	Challenges contributing to disrupted transition from paediatric to adult diabetes care in [illegible]	Pyatak, E. A.; Sequeira, P. A.; Whittemore, R.; Vigen, C. P.; Peters, A. L.; Weigensberg, M. J.	2014	Diabetic Medicine	31	12	1615-1624	10.1111/dme.12485	Pyatak 2014	Irrelevant	Excluded by TI/AB review
989	Protecting the heart of the American Athlete: Proceedings of the American college of cardiology sports and exercise cardiology think tank October 18, [illegible]	Rooks, Y. L.; Matherne, G. P.; Whitehead, J.; Henkel, D.; Asif, I. M.; Dreese, J. C.; Weiner, R. B.; Hutchinson, B. A.; Tavares, L.; Krueger, S.; Gordon, M. J.; [illegible]	2014	Journal of the American College of Cardiology	64	20	2146-2171	10.1016/j.jacc.2014.08.027	Rooks 2014	Irrelevant	Excluded by TI/AB review
990	Streamlining HIV testing in the emergency department-leveraging kiosks to provide true universal [illegible]	Rothman, R. E.; Gauvey-Kern, M.; Woodfield, A.; Peterson, S.; Tizenberg, B.; Kennedy, J.; Bush, D.; Locke, W.; [illegible]	2014	Telemedicine journal and e-health : the official journal of the American [illegible]	20	2	122-127	10.1089/tmj.2013.0045	Rothman 2014	Irrelevant	Excluded by TI/AB review

S4 Table. All studies identified in the literature search

991	Compliance with home-based fortification strategies for delivery of iron and zinc: Its effect on haematological and growth markers among 6-24 months old children in North India	Sazawal, S.; Dhingra, P.; Dhingra, U.; Gupta, S.; Iyengar, V.; Menon, V. P.; Sarkar, A.; Black, R. E.	2014	Journal of Health, Population and Nutrition	32	2	217-226	NA	Sazawal 2014	Irrelevant	Excluded by TI/AB review
992	Characteristics of patients who accept and decline ED rapid HIV testing	Schechter-Perkins, E. M.; Koppelman, E.; Mitchell, P. M.; Morgan, J. R.; Kutzen, R.; Drainoni, M. L.	2014	American Journal of Emergency Medicine	32	9	1109-1112	10.1016/j.ajem.2014.05.034	Schechter-Perkins 2014	Irrelevant	Excluded by TI/AB review
993	Frequent callers to and users of emergency medical systems: A systematic review	Scott, J.; Strickland, A. P.; Warner, K.; Dawson, P.	2014	Emergency Medicine Journal	31	8	684-691	10.1136/emermed-2013-202545	Scott 2014	Irrelevant	Excluded by TI/AB review

S4 Table. All studies identified in the literature search

994	Transitions in care: Improving the hospital discharge process for epilepsy patients	Secore, K.; Caller, T.; Rosenbaum, R.; Kleen, J.; Kaspar, J.; Harrington, J.; Jobst, B.	2014	Epilepsy Currents	14	NA	269-270	NA	Secore 2014	Irrelevant	Excluded by TI/AB review
995	What do patients want? Survey of patient desires for education in an urban university hospital	Seibert, T.; Veazey, K.; Leccese, P.; Druck, J.	2014	Western Journal of Emergency Medicine	15	7	764-769	10.5811/westjem.2014.9.20674	Seibert 2014	Irrelevant	Excluded by TI/AB review
996	Costs and clinical quality among medicare beneficiaries: Associations with health center penetration of low- [illegible]	Sharma, R.	2014	Medicare and Medicaid Research Review	4	3	NA	10.5600/mmrr.004.03.a05	Sharma 2014	Irrelevant	Excluded by TI/AB review

S4 Table. All studies identified in the literature search

997	You've got mail. . .And need follow-up	Sharp, B.; Maier, P.; Singal, B.; Fowler, J.; Simmons, S.	2014	Academic Emergency Medicine	21	5	S86	10.1111/acem.12365	Sharp 2014	Irrelevant	Excluded by TI/AB review
998	Diagnostic Overshadowing and Other Challenges	Shefer, G.; Henderson, C.; Howard, L. M.; Murray, J.;	2014	PLoS One	9	11	NA	10.1371/journal.pone.0111682	Shefer 2014	Irrelevant	Excluded by TI/AB review
999	The quality of care in acute coronary syndrome and its association with mortality in the united arab emirates: Data [illegible]	Shehab, A.; Yasin, J.; Al-Dabbagh, B.; Hashim, M. J.; Almahmeed, W.; Bustani, N.; Agrawal, A.; Yusufali, A. H.; [illegible]	2014	Gazzetta Medica Italiana Archivio per le Scienze Mediche	173	8-Jul	377-382	NA	Shehab 2014	Irrelevant	Excluded by TI/AB review
1000	Factors affecting patient compliance in the acute setting: An analysis of 20,000 imaging reports	Shuaib, W.; Vijayasarathi, A.; Johnson, J. O.; Salastekar, N.; He, Q.; Maddu, K. K.; Khosa, F.	2014	Emergency Radiology	21	4	373-379	10.1007/s10140-014-1209-1	Shuaib 2014	Irrelevant	Excluded by TI/AB review

S4 Table. All studies identified in the literature search

1001	Utilization of a state run public private emergency transportation service exclusively for childbirth: the Janani (maternal) Express program in Madhya Pradesh, India	Sidney, K.; Ryan, K.; Diwan, V.; De Costa, A.	2014	PLoS One	9	5	e96287	10.1371/journal.pone.0096287	Sidney 2014	Irrelevant	Excluded by TI/AB review
1002	The association of food insecurity and diabetes control among low-income individuals	Silverman, J. B.; Krieger, J.; Kiefer, M. M.; Hebert, P.; Drain, N.; Robinson, J.; Taylor, L.; Kapp, J.; Nelson, K. M.	2014	Journal of General Internal Medicine	29	NA	S220	NA	Silverman 2014	Irrelevant	Excluded by TI/AB review
1003	Different faces of discrimination: perceived discrimination among homeless adults with mental illness in healthcare settings	Skosireva, A.; O'Campo, P.; Zerger, S.; Chambers, C.; Gapka, S.; Stergiopoulos, V.	2014	BMC Health Services Research	14	NA	376	10.1186/1472-6963-14-376	Skosireva 2014	Irrelevant	Excluded by TI/AB review

S4 Table. All studies identified in the literature search

1005	Acute paediatric asthma exacerbations are associated with altered mucus quality, elevated sputum neutrophils, eosinophils and mediators associated with inflammation and viral infection	Staley, K. G.; May, R. D.; Woods, J.; Cohen, S.; Herath, A.; Monteiro, W.; Rousseau, K.; Thornton, D.; Brightling, C. E.; Gaillard, E. A.	2014	American Journal of Respiratory and Critical Care Medicine	189	NA	NA	NA	Staley 2014	Irrelevant	Excluded by TI/AB review
1006	Patient-centered medical home features and expenditures by medicare beneficiaries	Stockbridge, E. L.; Philpot, L. M.; Pa«µan, J. A.	2014	American Journal of Managed Care	20	5	379-385	NA	Stockbridge 2014	Irrelevant	Excluded by TI/AB review

S4 Table. All studies identified in the literature search

1007	Emergency department evaluation of conducted energy weapon (CEW)-injured patients	Strote, J.; Hutson, H. R.	2014	Journal of Emergency Medicine	46	4	533	10.1016/j.jemermed.2011.10.034	Strote 2014	Irrelevant	Excluded by TI/AB review
1008	Can response-adaptive randomization increase participation in acute stroke trials?	Tehranisa, J. S.; Meurer, W. J.	2014	Stroke	45	7	2131-2133	10.1161/STROKEAHA.114.005418	Tehranisa 2014	Irrelevant	Excluded by TI/AB review
1009	Comparison of toxicity between single modality radiation therapy and combined modality radiation therapy among low risk prostate cancer patients	Tomaszewski, J.; Jiang, R.; Ward, K.; Canter, D.	2014	Journal of Urology	191	4	e517	10.1016/j.juro.2014.02.1451	Tomaszewski 2014	Irrelevant	Excluded by TI/AB review

S4 Table. All studies identified in the literature search

1010	Adherence and health disparities associated with common clinical practice guidelines in the emergency department	Trent, S. A.; Quinlan, M.; Susalla, M.; Johnson, M. A.; Byyny, R. L.; Sankoff, J.; Havranek, E. P.; Haukoos, J. S.	2014	Academic Emergency Medicine	21	5	S136-S137	10.1111/acem.12365	Trent 2014	Irrelevant	Excluded by TI/AB review
1011	The impact of emergency medicine residents on the operational efficiency in a busy level 1	Trop, A. M.; Robinson, R.	2014	Annals of Emergency Medicine	64	4	S2	10.1016/j.annemergmed.2014.07.029	Trop 2014	Irrelevant	Excluded by TI/AB review
1012	Barriers and facilitators to pediatric emergency telemedicine in the United States	Uscher-Pines, L.; Kahn, J. M.	2014	Telemed J E Health	20	11	990-6	10.1089/tmj.2014.0015	Uscher-Pines 2014	Irrelevant	Excluded by TI/AB review

S4 Table. All studies identified in the literature search

1013	Tough guys or sensitive guys? Disentangling the role of examiner sex on patient pain reports	Vigil, J. M.; Alcock, J.	2014	Pain Research & Managemen t	19	1	E9-E12	10.1155/20 14/531396	Vigil 2014	Irrelevant	Excluded by TI/AB review
1014	Handbook of evidence-based practices for emotional and behavioral disorders: Applications in schools	Walker, Hill M.; Gresham, Frank M.	2014	NA	NA	NA	NA	NA	Walker 2014	Irrelevant	Excluded by TI/AB review

S4 Table. All studies identified in the literature search

1015	No difference in emergency department length of stay for patients with limited proficiency in English	Wallbrecht, J.; Hodes-Villamar, L.; Weiss, S. J.; Ernst, A. A.	2014	South Med J	107	1	5-Jan	10.1097/smj.0000000000000037	Wallbrecht 2014	Irrelevant	Excluded by TI/AB review
1018	ETHNIC MINORITIES AND THEIR HEALTH NEEDS: CRISIS OF PERCEPTION [illegible]	Wankhade, Paresh; Cook, Ian G.; Halsall, Jamie P.	2014	Illness, Crisis & Loss	22	2	145-158	10.2190/IL.22.2.e	Wankhade 2014	Irrelevant	Excluded by TI/AB review
1020	Regional disparities in breast cancer outcomes and the process of care	Weber, J. J.; Kachare, S. D.; Vohra, N. A.; Fitzgerald, T. F.; Wong, J. H.	2014	Am Surg	80	7	669-74	NA	Weber 2014	Irrelevant	Excluded by TI/AB review
1022	Utilization of triage phone encounters and after hours appointments in [illegible]	Webster, J.; Stevens, L.; Waters, T.; McAneny, B. L.	2014	Journal of Clinical Oncology	32	15	NA	NA	Webster 2014	Irrelevant	Excluded by TI/AB review
1023	Patient attitudes regarding consent for emergency department computed tomographies	Weigner, M. B.; Basham, H. F.; Dewar, K. M.; Rupp, V. A.; Cornelius, L.; Greenberg, M. R.	2014	Western Journal of Emergency Medicine	15	1	NA	10.5811/westjem.2013.5.15893	Weigner 2014	Irrelevant	Excluded by TI/AB review

S4 Table. All studies identified in the literature search

1024	A randomized clinical trial to test the efficacy of a family-focused, culturally informed therapy for schizophrenia	Weisman de Mamani, A.; Weintraub, M. J.; Gurak, K.; Maura, J.	2014	Journal of family psychology : JFP : journal of the Division of Family [illegible]	28	6	800-810	10.1037/fam0000021	Weisman deMamani 2014	Irrelevant	Excluded by TI/AB review
1025	‚ÄúVulnerable patients in the ED‚Äù didactic curriculum: Using patient voices to increase empathy and knowledge for emergency medicine residents	Whitehead, A.; Wilbur, L.; Walthall, J. D.	2014	Academic Emergency Medicine	21	5	S342	10.1111/acem.12365	Whitehead 2014	Irrelevant	Excluded by TI/AB review
1026	Racial disparities in diagnosis in patients presenting to emergency rooms: Persisting problem of misdiagnosis?	William, L.	2014	Comprehensive Psychiatry	55	8	e53	10.1016/j.comppsych.2014.08.026	William 2014	Irrelevant	Excluded by TI/AB review

S4 Table. All studies identified in the literature search

1027	Factors Influencing 30-Day Emergency Visits and Readmissions after Sleeve Gastrectomy: Results from a Community Bariatric Center	Willson, T. D.; Gomberawalla, A.; Mahoney, K.; Lutfi, R. E.	2014	Obesity Surgery	NA	NA	NA	10.1007/s11695-014-1546-x	Willson 2014	Irrelevant	Excluded by TI/AB review
1028	The maximizing independence (MIND) at home care coordination program for elders with memory disorders: Effect [illegible]	Wong, S. K.; Roth, D. L.; Huang, J.; Black, B.; Johnston, D.; Rabins, P.; Gitlin, L.; Lyketsos, C.; Samus, Q.	2014	Journal of the American Geriatrics Society	62	NA	S9	10.1111/jgs.12870	Wong 2014	Irrelevant	Excluded by TI/AB review

S4 Table. All studies identified in the literature search

1029	Adherence to controller medications for asthma is poor	Wu, A. C.; Butler, M.; Li, L.; Fung, V.; Kharbanda, E.; Vollmer, W. M.; Miroshnik, I.; Lieu, T.; Soumerai, S.	2014	American Journal of Respiratory and Critical Care Medicine	189	NA	NA	NA	Wu 2014	Irrelevant	Excluded by TI/AB review
1030	Pilot study on association of single-nucleotide polymorphisms with hydromorphone analgesia in emergency department	Xia, S.; Persaud, S.; Birnbaum, A.	2014	Academic Emergency Medicine	21	5	S22-S23	10.1111/acem.12365	Xia 2014	Irrelevant	Excluded by TI/AB review
1031	Quality of care for incident lupus nephritis among medicaid beneficiaries in the United States	Yazdany, J.; Feldman, C. H.; Liu, J.; Ward, M. M.; Fischer, M. A.; Costenbader, K. H.	2014	Arthritis Care and Research	66	4	617-624	10.1002/acr.22182	Yazdany 2014	Irrelevant	Excluded by TI/AB review

S4 Table. All studies identified in the literature search

1032	Do primary care physicians who deliver better quality of care also use less health care resources?	Yelibi, C.; Ward, C. E.; Chang, Y.; Ashburner, J. M.; Hong, C. S.; Atlas, S. J.	2014	Journal of General Internal Medicine	29	NA	S75	NA	Yelibi 2014	Irrelevant	Excluded by TI/AB review
1033	Targeted therapy at the end of life and the impact on health care utilization	Yeung, H.; Salehi, P.; Don Murphy, J.	2014	Journal of Clinical Oncology	32	31	NA	NA	Yeung 2014	Irrelevant	Excluded by TI/AB review
1034	Liquid medication dosing errors in children: Role of provider counseling strategies	Yin, H. S.; Dreyer, B. P.; Moreira, H. A.; Van Schaick, L.; Rodriguez, L.; Boettger, S.; Mendelsohn, A. L.	2014	Academic Pediatrics	14	3	262-270	10.1016/j.acap.2014.01.003	Yin 2014	Irrelevant	Excluded by TI/AB review

S4 Table. All studies identified in the literature search

1035	Emergency department patient knowledge, opinions, and risk tolerance regarding computed tomography scan radiation	Youssef, N. A.; Gordon, A. J.; Moon, T. H.; Patel, B. D.; Shah, S. J.; Casey, E. M.; McGowan, A. J.; Weiner, S. G.	2014	Journal of Emergency Medicine	46	2	208-214	10.1016/j.jemermed.2013.07.016	Youssef 2014	Irrelevant	Excluded by TI/AB review
1036	Trajectories of Adolescent Alcohol Use in the Year Following a Brief Alcohol Intervention	Abar, C. C.; Hernandez, L.; Rodriguez, A. M.; Spirito, A.	2015	Journal of Studies on Alcohol and Drugs	76	5	710-720	NA	Abar 2015	Irrelevant	Excluded by TI/AB review
1037	Racial Disparities in End-of-Life Care Among Patients With Prostate Cancer: A Population-Based [illegible]	Abdollah, Firas; Sammon, Jesse D.; Majumder, Kaustav; Reznor, Gally; Gandaglia, Giorgio; Sood, Akshay; [illegible]	2015	Journal of the National Comprehensive Cancer Network	13	9	1131-1138	NA	Abdollah 2015	Irrelevant	Excluded by TI/AB review

S4 Table. All studies identified in the literature search

1040	Frequent emergency department use among non-elderly adults: Analysis of multi-state medicaid claims data	Agarwal, P.; Bias, T.; Frisbee, S.; Sambamoorthi, N.; Bose, S.; Madhavan, S. S.; Sambamoorthi, U.	2015	Value in Health	18	3	A266	NA	Agarwal 2015	Irrelevant	Excluded by TI/AB review
1041	Using process indicators to optimize service completion of an ED drug and alcohol brief intervention program	Akin, J.; Johnson, J. A.; Seale, J. P.; Kuperminc, G. P.	2015	American Journal of Emergency Medicine	33	1	37-42	10.1016/j.ajem.2014.10.021	Akin 2015	Irrelevant	Excluded by TI/AB review
1042	Using IT to improve access, communication, and asthma in African American and Hispanic/Latino Adults: Rationale, [illegible]	Apter, A. J.; Bryant, Stephens; Morales, K. H.; Wan, F.; Hardy, S.; Reed-Wells, S.; Dominguez, M.; Gonzalez, R.; Mak, N.; Nardi, A.; Park, [illegible]	2015	Contemporary Clinical Trials	44	NA	119-128	10.1016/j.cct.2015.08.001	Apter 2015	Irrelevant	Excluded by TI/AB review

S4 Table. All studies identified in the literature search

1043	Fentanyl buccal tablet versus oral oxycodone for Emergency Department treatment of	Arthur, A. O.; Mushtaq, N.; Mumma, S.; Thomas, S. H.	2015	Journal of Emergency Medicine, Trauma and Acute Care	2015	NA	NA	10.5339/jemtac.2015.6	Arthur 2015	Irrelevant	Excluded by TI/AB review
1044	Association of payment source with emergency department use by patients with diabetes in the United States	Asao, K.; Lee, J. M.; Wang, L.; Kocak, M.; McEwen, L. N.; Herman, W. H.; Bailey, J. E.	2015	Diabetes	64	NA	A436	10.2337/db1514721800	Asao 2015	Irrelevant	Excluded by TI/AB review
1045	The Effect of Structural Violence on Patients with	Bahr, N. C.; Song, J.	2015	Journal of Health Care for the Poor and	26	3	648-661	10.1353/hpu.2015.0094	Bahr 2015	Irrelevant	Excluded by TI/AB review
1046	Race and social problems: Restructuring inequality	Bangs, Ralph; Davis, Larry E.	2015	NA	NA	NA	NA	10.1007/978-1-4939-0863-9	Bangs 2015	Irrelevant	Excluded by TI/AB review

S4 Table. All studies identified in the literature search

1047	The Influence of Health Care Professional Characteristics on Pain Management	Bartley, E. J.; Boissoneault, J.; Vargovich, A. M.; Wandner, L. D.; Hirsh, A. T.; Lok, B.	2015	Pain Medicine	16	1	99-111	10.1111/pme.12591	Bartley 2015	Irrelevant	Excluded by TI/AB review
1048	The role of stable housing as a determinant of poverty-related quality of life in vulnerable individuals	Baumstarck, K.; Boyer, L.; Auquier, P.	2015	International Journal for Quality in Health Care	27	5	356-360	10.1093/intqhc/mzv052	Baumstarck 2015	Irrelevant	Excluded by TI/AB review
1050	Effect of a pharmacist counseling intervention on healthcare	Bell, S. P.; Schnipper, J. L.; Goggins, K.; Bian, A.; Shintani, A.; Roumie, C.; Dalal,	2015	Journal of General Internal Medicine	30	NA	S55	NA	Bell 2015	Irrelevant	Excluded by TI/AB review

S4 Table. All studies identified in the literature search

1051	External validation of the Ottawa subarachnoid hemorrhage clinical decision rule in patients with acute headache	Bellolio, M. F.; Hess, E. P.; Gilani, W. I.; Vandyck, T. J.; Ostby, S. A.; Schwarz, J. A.; Lohse, C. M.; Rabinstein, A. A.	2015	American Journal of Emergency Medicine	33	2	244-249	10.1016/j.ajem.2014.11.049	Bellolio 2015	Irrelevant	Excluded by TI/AB review
1052	The factors influencing utilization of hospital services by adult sickle cell disease patients: A systematic review protocol	Benenson, I.; Jadotte, Y. T.; Echevarria, M.	2015	JBI Database of Systematic Reviews and Implementation Reports	13	9	18-29	10.11124/jbisrir-2015-2247	Benenson 2015	Irrelevant	Excluded by TI/AB review
1053	A youth suicide prevention plan for Canada: A systematic review of reviews	Bennett, K.; Rhodes, A. E.; Duda, S.; Cheung, A. H.; Manassis, K.; Links, P.; Mushquash, C.; Braunberger, P.; Newton, A. S.; Kutcher, S.; Bridge, J. A.; Santos, R. G.; [illegible]	2015	Canadian Journal of Psychiatry	60	6	245-257	10.1177/070674371506000603	Bennett 2015	Irrelevant	Excluded by TI/AB review

S4 Table. All studies identified in the literature search

1054	Evaluation of Simple Triage Algorithm and Rapid Treatment and Sort, Assess, Lifesaving, Interventions, Treatment, and Transportation mass casualty triage methods for sensitivity, specificity, and predictive values	Bhalla, M. C.; Frey, J.; Rider, C.; Nord, M.; Hegerhorst, M.	2015	American Journal of Emergency Medicine	33	11	NA	10.1016/j.ajem.2015.08.021	Bhalla 2015	Irrelevant	Excluded by TI/AB review
1055	Frequent users of the emergency department in a universal health	Bodenmann, P.; Velonaki, V. S.; Baggio, S.; Iglesias, K.; Moschetti, K.;	2015	Journal of General Internal Medicine	30	NA	S167	NA	Bodenmann 2015	Irrelevant	Excluded by TI/AB review
1056	Predictors of patient adherence to follow-up recommendations after an ED visit	Broadwater-Hollifield, C.; Madsen, T. E.; Porucznik, C. A.; Sundwall, D. N.; Youngquist, S. T.; [illegible]	2015	American Journal of Emergency Medicine	33	10	1368-1373	10.1016/j.ajem.2015.07.032	Broadwater-Hollifield 2015	Irrelevant	Excluded by TI/AB review

S4 Table. All studies identified in the literature search

1057	Germs are germs, and why not take a risk? Patients' expectations for prescribing antibiotics in an inner-city emergency [illegible]	Broniatowski, D. A.; Klein, E. Y.; Reyna, V. F.	2015	Medical decision making : an international journal of the Society for Medical Decision [illegible]	35	1	60-67	10.1177/0272989X14553472	Broniatowski 2015	Irrelevant	Excluded by TI/AB review
1060	Issues in the assessment and treatment of African American patients	Burroughs-Gardner, Tracee; Primm, Annelle B.; Lawson, William B.; Cohen, Deborah; Lim, Russell F.	2015	Clinical manual of cultural psychiatry., 2nd ed.	NA	NA	77-125	NA	Burroughs-Gardner 2015	Irrelevant	Excluded by TI/AB review
1061	Comparison of prognostic factors between hispanic and non-hispanic adolescents with eating disorders	Cabral, M. D. I.; Siqueira, L. M.; Hospital, M. M.	2015	Journal of Adolescent Health	56	2	S89-S90	10.1016/j.jadohealth.2014.10.179	Cabral 2015	Irrelevant	Excluded by TI/AB review
1063	Travel distances, socioeconomic characteristics, and health disparities in nonurgent and frequent use of Hospital Emergency [illegible]	Chen, B. K.; Cheng, X.; Bennett, K.; Hibbert, J.	2015	BMC Health Serv Res	15	NA	203	10.1186/s12913-015-0864-6	Chen 2015	Irrelevant	Excluded by TI/AB review

S4 Table. All studies identified in the literature search

1065	Gender differences in stemi activation for patients presenting to the emergency department	Choi, K.; Shofer, F. S.; Mills, A. M.	2015	Academic Emergency Medicine	22	5	S146	10.1111/acem.12644	Choi 2015	Irrelevant	Excluded by TI/AB review
1066	The Relationship Among Heart Failure Disease Management,	Chung, E. S.; Bartone, C.; Daly, K.; Menon, S.; McDonald, M.	2015	The Journal of medical practice management	31	3	172-178	NA	Chung 2015	Irrelevant	Excluded by TI/AB review
1068	Patients' attitudes regarding tattooed physicians: The art study	Cohen, M.; Jeanmonod, D.; Stankewicz, H.; Coppersmith, V.; Berrios, M.; Habeeb, K.; Jeanmonod, R.	2015	Annals of Emergency Medicine	66	4	S97	NA	Cohen 2015	Irrelevant	Excluded by TI/AB review

S4 Table. All studies identified in the literature search

1069	Medication review and transitions of care: A case report of a decade-old medication error	Comer, R.; Lizer, M.	2015	Consultant Pharmacist	30	3	130-135	10.4140/TCP.n.2015.130	Comer 2015	Irrelevant	Excluded by TI/AB review
1070	Factors associated with follow-up attendance among rape victims seen in acute medical care	Darnell, D.; Peterson, R.; Berliner, L.; Stewart, T.; Russo, J.; Whiteside, L.; Zatzick, D.	2015	Psychiatry (New York)	78	1	89-101	10.1080/00332747.2015.1015901	Darnell 2015	Irrelevant	Excluded by TI/AB review
1071	Racial differences in opiate administration for pain relief at an academic emergency department	Dickason, R. M.; Chauhan, V.; Mor, A.; Ibler, E.; Kuehnle, S.; Mahoney, D.; Armbrecht, E.; Dalawari, P.	2015	West J Emerg Med	16	3	372-80	10.5811/westjem.2015.3.23893	Dickason 2015	Irrelevant	Excluded by TI/AB review

S4 Table. All studies identified in the literature search

1074	Impact of gender and age on the response to therapy in the asthma clinical research network	Dunn, R. M.; Lehman, E.; Chinchilli, V. M.; Martin, R. J.; Boushey, H. A.; Israel, E.; Kraft, M.; Lazarus, S. C.; Lemanske, R. F.; Lugogo, N.; Peters, S. P.; Sorkness, C. A.; Szefler, S. J.; [illegible]	2015	American Journal of Respiratory and Critical Care Medicine	191	NA	NA	NA	Dunn 2015	Irrelevant	Excluded by TI/AB review
1075	Unplanned Transfer to Emergency Departments for Frail Elderly Residents of Aged Care Facilities: A Review of Patient and Organizational Factors	Dwyer, R.; Stoelwinder, J.; Gabbe, B.; Lowthian, J.	2015	Journal of the American Medical Directors Association	16	7	551-562	10.1016/j.jamda.2015.03.007	Dwyer 2015	Irrelevant	Excluded by TI/AB review
1076	Current worldwide nuclear cardiology practices andradiationexposure: Results from the 65country [illegible]	Einstein, A. J.; Pascual, T. B.; Mercuri, M.; Karthikeyan, G.; Vitola, J. V.; Mahmarian, J. J.; [illegible]	2015	European Heart Journal	36	26	1689-1696	10.1093/eurheartj/ehv117	Einstein 2015	Irrelevant	Excluded by TI/AB review

S4 Table. All studies identified in the literature search

1077	Persistent differences in asthma self-efficacy by race, ethnicity, and income in adults [illegible]	Ejebe, I. H.; Jacobs, E. A.; Wisk, L. E.	2015	Journal of Asthma	52	1	105-113	10.3109/02770903.2014.947429	Ejebe 2015	Irrelevant	Excluded by TI/AB review
1078	Satisfaction with care and decision making among parents/caregivers in the pediatric intensive care unit: A comparison between English-speaking whites and Latinos	Epstein, D.; Unger, J. B.; Ornelas, B.; Chang, J. C.; Markovitz, B. P.; Dodek, P. M.; Heyland, D. K.; Gold, J. I.	2015	Journal of Critical Care	30	2	236-241	10.1016/j.jcrc.2014.11.009	Epstein 2015	Irrelevant	Excluded by TI/AB review
1080	Patient compliance to filling discharge medication prescriptions from the emergency department	Farris, B.; Jacknin, G.; Kiser, T.; Shakowski, C.; Mueller, S.	2015	Pharmacotherapy	35	11	e298	10.1002/phar.1659	Farris 2015	Irrelevant	Excluded by TI/AB review
1081	Homeless patients in France: A national case-cohort prospective study	Feral-Pierssens, A. L.; Aubry, A.; Truchot, J.; Raynal, P. A.; Hutin, A.; Debruyne, G.; Joly, L. M.; Juvin, P.; Riou, B.; Freund, Y.	2015	Annals of Emergency Medicine	66	4	S104	NA	Feral-Pierssens 2015	Irrelevant	Excluded by TI/AB review

S4 Table. All studies identified in the literature search

1082	The relationship between emergency department utilization and health care professional [illegible]	Fertel, B. S.; Podolsky, S. R.; Hart, K. W.; Powell, L.; Jennings, M.; Mace, S. E.	2015	Annals of Emergency Medicine	66	4	S13	NA	Fertel 2015	Irrelevant	Excluded by TI/AB review
1083	Emf identification of emergency medicine fatigue at-risk periods using actigraphy and computer modeling: A pilot study	Fox, C.; Hall, V.; Schaefer, T.; Wolford, R.	2015	Annals of Emergency Medicine	66	4	S146-S147	NA	Fox 2015	Irrelevant	Excluded by TI/AB review

S4 Table. All studies identified in the literature search

1084	A nurse practitioner run urgent heart failure clinic: Timely treatment can decrease costly hospital admissions/readmissions	Frazier, S.; Baksh, G.; Black, S.	2015	Heart and Lung	44	6	555-556	NA	Frazier 2015	Irrelevant	Excluded by TI/AB review
1085	Barriers to Homeless Persons Acquiring Health Insurance Through the Affordable Care Act	Fryling, L. R.; Mazanec, P.; Rodriguez, R. M.	2015	J Emerg Med	49	5	755-62.e2	10.1016/j.jemermed.2015.06.005	Fryling 2015	Irrelevant	Excluded by TI/AB review
1086	Transition readiness, asthma morbidity and asthma health related quality of [illegible]	Gibson-Scipio, W. M.; MacDonell, K.; Yarandi, H.; Pearson, C.; Wade, P.	2015	Journal of Adolescent Health	56	2	S126	10.1016/j.jadohealth.2014.10.252	Gibson-Scipio 2015	Irrelevant	Excluded by TI/AB review

S4 Table. All studies identified in the literature search

1087	Patient education and follow-up as an intervention for hypertensive patients discharged from an	Gleason-Comstock, J.; Streater, A.; Ager, J.; Goodman, A.; Brody, A.; Kivell, L.; Paranjpe, A.; Vickers, J.; Mango, [illegible]	2015	BMC Emergency Medicine	15	1	NA	10.1186/s12873-015-0052-3	Gleason-Comstock 2015	Irrelevant	Excluded by TI/AB review
1088	Palliative care domains for emergency medicine resident training: An expert consensus	Goett, R.; Shoenberger, J.; DeSandre, P.; Jubanyik, K.; Aberger, K.; Bigelow, S.; Brandtman, T.; Chan, G.; Rosenberg, M.; Wang, D.; Lamba, S.	2015	Annals of Emergency Medicine	66	4	S83	NA	Goett 2015	Irrelevant	Excluded by TI/AB review
1089	Redesigned geriatric emergency care may have helped reduce admissions of older adults to intensive care units	Grudzen, C.; Richardson, L. D.; Baumlin, K. M.; Winkel, G.; Davila, C.; Ng, K.; Hwang, U.	2015	Health Affairs	34	5	788-795	10.1377/hlthaff.2014.0790	Grudzen 2015	Irrelevant	Excluded by TI/AB review
1090	Racial/ethnic differences in destination emergency departments for acute care	Hanchate, A. D.; Paasche-Orlow, M.; Baker, W. E.; Dyer, S.; Feng, C.; Feldman, J.	2015	Journal of General Internal Medicine	30	NA	S242	NA	Hanchate 2015	Irrelevant	Excluded by TI/AB review

S4 Table. All studies identified in the literature search

1091	Patient- and Family-Centered Care as an approach to reducing disparities in asthma outcomes in urban African American children: [illegible]	Harper, F. W.; Eggly, S.; Crider, B.; Kobayashi, H.; Kathleen, R. N.; Meert, L.; Ball, A.; Penner, L. A.; Gray, H.; Albrecht, T. L.	2015	J Natl Med Assoc	107	2	17-Apr	10.1016/s0027-9684(15)30019-5	Harper 2015	Irrelevant	Excluded by TI/AB review
1092	Modern societal impacts of the model minority stereotype	Hartlep, Nicholas Daniel	2015	NA	NA	NA	NA	10.4018/978-1-4666-7467-7	Hartlep 2015	Irrelevant	Excluded by TI/AB review
1093	Killing the model minority	Hartlep, Nicholas D.; Porfilio, Bradley J.	2015	NA	NA	NA	NA	NA	Hartlep 2015	Irrelevant	Excluded by TI/AB review
1094	Use of point-of-care ultrasound in connecticut	Herbst, M. K.; Camargo, C. A.; Perez, A.; Moore, C.	2015	Journal of Emergency Medicine	48	2	191-196	10.1016/j.jemermed.2014.09.017	Herbst 2015	Irrelevant	Excluded by TI/AB review

S4 Table. All studies identified in the literature search

1095	Causes of delay in door-to-balloon time in south-east Asian patients undergoing primary percutaneous coronary intervention for ST-segment elevation myocardial	Ho, H. H.; Wong, S. W.; Mok, K. H.; Tan, M. C.; Sim, W. J.; Ang, A. S.; Quah, K. H.; Foo, D.; Ong, P. J. L.	2015	European Heart Journal	36	NA	141	10.1093/eurheartj/ehv398	Ho 2015	Irrelevant	Excluded by TI/AB review
1096	High-cost patients: ‚ÄúHot-spotters‚Äù don't explain the half of it	Hong, S. B. N.; Whitman, N.; Vakharia, N.; Taksler, G. B.; Rothberg, M. B.	2015	Journal of General Internal Medicine	30	NA	S174	NA	Hong 2015	Irrelevant	Excluded by TI/AB review
1097	Impact of case management on frequent users' quality of life: A randomized, controlled trial	Iglesias, K.; Moschetti, K.; Baggio, S.; Velonaki, V.; Ruggeri, O.; Hugli, O.; Burnand, B.; Wasserfallen, J. B.; Daeppen, J. B.; Bodenmann, P.	2015	Praxis	104	NA	45-46	10.1024/1661-8157/a001994	Iglesias 2015	Irrelevant	Excluded by TI/AB review

S4 Table. All studies identified in the literature search

1098	Reasons for and reservations about research participation in acutely injured adults	Irani, E.; Richmond, T. S.	2015	Journal of nursing scholarship : an official publication of Sigma [illegible]	47	2	161-169	10.1111/jnu.12120	Irani 2015	Irrelevant	Excluded by TI/AB review
1099	Factors Associated With Orthopedic Aftercare in a Publicly Insured Pediatric Emergency Department Population	Jamal, N.; Iqbal, S. F.; Ryan, L. M.	2015	Pediatric Emergency Care	31	10	704-707	10.1097/PEC.0000000000000562	Jamal 2015	Irrelevant	Excluded by TI/AB review
1101	Acceptability of Alternatives to Traditional Emergency Care: Patient Characteristics, Alternate Transport Modes, and Alternate [illegible]	Jones, C. M.; Wasserman, E. B.; Li, T.; Shah, M. N.	2015	Prehospital emergency care : official journal of the National Association of EMS Physicians [illegible]	19	4	516-523	10.3109/10903127.2015.1025156	Jones 2015	Irrelevant	Excluded by TI/AB review

S4 Table. All studies identified in the literature search

1102	Reducing Racial Disparities Improves ED Patient Outcomes	Katz, Alissa	2015	Emergency Medicine News	37	6	25-Jan	10.1097/01.EEM.0000466613.47628.41	Katz 2015	Irrelevant	Excluded by TI/AB review
1103	Out-of-pocket cost, expenditures, and health-related quality of life in managed care plan members	Kawatkar, A.	2015	Journal of Managed Care and Specialty Pharmacy	21	NA	S73	NA	Kawatkar 2015	Irrelevant	Excluded by TI/AB review
1104	The Influence of Setting on Care Coordination for Childhood Asthma	Kelly, R. P.; Stoll, S. C.; Bryant-Stephens, T.; Janevic, M. R.; Lara, M.; Ohadike, Y. U.; Persky, V.; Ramos-Valencia, G.; Uyeda, K.; Malveaux, F. J.	2015	Health Promotion Practice	16	6	867-877	10.1177/1524839915598499	Kelly 2015	Irrelevant	Excluded by TI/AB review

S4 Table. All studies identified in the literature search

1105	Emergency physician attitudes, preferences, and risk tolerance for stroke as a potential cause of dizziness symptoms	Kene, M. V.; Ballard, D. W.; Vinson, D. R.; Rauchwerger, A. S.; Iskin, H. R.; Kim, A. S.	2015	Western Journal of Emergency Medicine	16	5	768-776	10.5811/westjem.2015.7.26158	Kene 2015	Irrelevant	Excluded by TI/AB review
1106	Gender, age and ethnicity influence on pain levels and analgesic use in the acute whiplash injury	Koren, L.; Peled, E.; Trogan, R.; Norman, D.; Berkovich, Y.; Israelit, S.	2015	European Journal of Trauma and Emergency Surgery	41	3	287-291	10.1007/s00068-014-0419-2	Koren 2015	Irrelevant	Excluded by TI/AB review
1107	Two types of prehospital systems interventions that triage low-acuity patients to alternative sites of care	Krumperman, K.; Weiss, S.; Fullerton, L.	2015	Southern Medical Journal	108	7	381-386	10.14423/SMJ.0000000000000303	Krumperman 2015	Irrelevant	Excluded by TI/AB review

S4 Table. All studies identified in the literature search

1108	Coronary computed tomography angiography versus radionuclide myocardial perfusion imaging [illegible]	Levsky, J. M.; Spevack, D. M.; Travin, M. I.; Menegus, M. A.; Huang, P. W.; Clark, E. T.; Kim, C. W.; Hirschhorn, E.; Freeman, K. D.; [illegible]	2015	Annals of Internal Medicine	163	3	174-183	10.7326/M14-2948	Levsky 2015	Irrelevant	Excluded by TI/AB review
1109	Patient acuity: Disparity between patients and	Lightbody, A.; Mace, S.	2015	Annals of Emergency Medicine	66	4	S120	NA	Lightbody 2015	Irrelevant	Excluded by TI/AB review
1110	Delayed stress delta n-terminal pro-b type natriuretic peptide is significantly higher with myocardial ischemia	Limkakeng Jr, A. T.; Lokhnygina, Y.; Sandesara, H.; Drake, W.; Christenson, R.; Newby, L. K.	2015	Annals of Emergency Medicine	66	4	S38-S39	NA	LimkakengJr 2015	Irrelevant	Excluded by TI/AB review
1111	Delayed stress-delta high sensitivity troponin does not elevate with myocardial ischemia	Limkakeng Jr, A. T.; Lokhnygina, Y.; Sandesara, H.; Drake, W.; Christenson, R.; Newby, L. K.	2015	Annals of Emergency Medicine	66	4	S39-S40	NA	LimkakengJr 2015	Irrelevant	Excluded by TI/AB review
1112	Who attends Dunedin's free clinic? A study of patients facing cost	Loh, L.; Dovey, S.	2015	Journal of primary health care	7	1	16-23	NA	Loh 2015	Irrelevant	Excluded by TI/AB review

S4 Table. All studies identified in the literature search

1113	Reducing Childhood Asthma Triggers in Public Housing: Implementation and Outcomes from an East Harlem Community Health Worker Program	Lopez, R.; Chantarat, T.; Bozack, A.; Lopez, A.; Weiss, L.	2015	Environmental Justice	8	5	185-191	10.1089/env.2015.0017	Lopez 2015	Irrelevant	Excluded by TI/AB review
1114	Homeless and hungry? See the highland health advocates! A pilot study of a safety net hospital-based medical-legal partnership and resource referral desk	Losonczy, L. I.; Hsieh, D.; Hahn, C.; Trivedi, T.; Rodriguez, M.; Fahimi, J.; Alter, H.	2015	Academic Emergency Medicine	22	5	S122	10.1111/acem.12644	Losonczy 2015	Irrelevant	Excluded by TI/AB review

S4 Table. All studies identified in the literature search

1115	The use of the needle-free jet injection system with buffered lidocaine device does not change intravenous placement success in children in the emergency department	Lunoe, M. M.; Drendel, A. L.; Brousseau, D. C.	2015	Academic Emergency Medicine	22	4	447-451	10.1111/acem.12629	Lunoe 2015	Irrelevant	Excluded by TI/AB review
1116	Early Dialysis and Adverse Outcomes after Hurricane Sandy	Lurie, N.; Finne, K.; Worrall, C.; Jauregui, M.; Thaweethai, T.; Margolis, G.; Kelman, J.	2015	American Journal of Kidney Diseases	66	3	507-512	10.1053/j.ajkd.2015.04.050	Lurie 2015	Irrelevant	Excluded by TI/AB review
1117	Child mental health services in the emergency department disparities in access	Lynch, S.; Bautista, M.; Freer, C.; Kalynych, C.; Hendry, P.	2015	Pediatric Emergency Care	31	7	473-478	10.1097/PEC.0000000000000336	Lynch 2015	Irrelevant	Excluded by TI/AB review

S4 Table. All studies identified in the literature search

1118	The association between physical activity and sexual dysfunction in patients with diabetes mellitus [illegible]	Malavige, L. S.; Wijesekara, P.; Ranasinghe, P.; Levy, J. C.	2015	European Journal of Medical Research	20	1	NA	10.1186/s40001-015-0186-5	Malavige 2015	Irrelevant	Excluded by TI/AB review
1119	Expanding Telemedicine to Include Primary Care for the Urban Adult	Markwick, L.; McConnochie, K.; Wood, N.	2015	Journal of Health Care for the Poor and Underserved	26	3	771-776	10.1353/hpu.2015.0078	Markwick 2015	Irrelevant	Excluded by TI/AB review
1120	Acute care of older patients in the emergency department: Strategies to improve patient	McCabe, J. J.; Kennelly, S. P.	2015	Open Access Emergency Medicine	7	NA	45-54	10.2147/OAEM.S69974	McCabe 2015	Irrelevant	Excluded by TI/AB review

S4 Table. All studies identified in the literature search

1121	Association of anxiety and dyspnea in emergency department patients with acute heart failure	McCafferty, L. R.; Hart, K. W.; Lindsell, C. J.; Collins, S. P.	2015	Academic Emergency Medicine	22	5	S164	10.1111/acem.12644	McCafferty 2015	Irrelevant	Excluded by TI/AB review
1122	Acceptability of self-sample human papillomavirus testing among medically underserved women visiting the emergency department	Montealegre, J. R.; Landgren, R. M.; Anderson, M. L.; Hoxhaj, S.; Williams, S.; Robinson, D. J.; Scheurer, M. E.; Ramondetta, L. M.	2015	Gynecologic Oncology	138	2	317-322	10.1016/j.ygyno.2015.05.028	Montealegre 2015	Irrelevant	Excluded by TI/AB review
1124	Feasibility, acceptability, and initial efficacy of an online sexual health promotion	Mustanski, B.; Greene, G. J.; Ryan, D.; Whitton, S. W.	2015	Journal of sex research	52	2	220-230	10.1080/00224499.2013.867924	Mustanski 2015	Irrelevant	Excluded by TI/AB review
1125	Children's emergency department use for asthma, 2001-2010	Nath, J. B.; Hsia, R. Y.	2015	Acad Pediatr	15	2	225-30	10.1016/j.acap.2014.10.011	Nath 2015	Irrelevant	Excluded by TI/AB review

S4 Table. All studies identified in the literature search

1126	Association of hispanic ethnicity with acute ischemic stroke	Neil, W. P.; Raman, R.; Hemmen, T. M.; Ernstrom, K.; Meyer, B. C.; Meyer, D. M.;	2015	Ethnicity and Disease	25	1	19-23	NA	Neil 2015	Irrelevant	Excluded by TI/AB review
1129	Emergency physician's perception of cultural and linguistic barriers in immigrant care: [illegible]	Numeroso, F.; Benatti, M.; Pizzigoni, C.; Sartori, E.; Lippi, G.; Cervellin, G.	2015	World Journal of Emergency Medicine	6	2	111-117	10.5847/wjem.j.1920-8642.2015.02.005	Numeroso 2015	Irrelevant	Excluded by TI/AB review
1131	Preliminary analysis of use of a bidirectional text messaging system for ED follow-up versus standard-care follow-up	Obr, B. J.; Nugent, A.; Miller, M.; Harland, K.; Gadhok, K.	2015	Academic Emergency Medicine	22	5	S81	10.1111/acem.12644	Obr 2015	Irrelevant	Excluded by TI/AB review
1132	Primary health care accessibility challenges in remote indigenous communities in Canada's North	Oosterveer, T. M.; Young, T. K.	2015	Int J Circumpolar Health	74	NA	29576	10.3402/ijch.v74.29576	Oosterveer 2015	Irrelevant	Excluded by TI/AB review

S4 Table. All studies identified in the literature search

1133	Alternative care prior to emergency department visits in Alberta: Stop blaming the patient	Ospina, M.; Lowes, J.; Long, R.; Giese, E.; Vuyst, D.; Pang, P.; Chow, P.; Van Der Kley, K.; Arrotta, N.; Couperthwaite, S.; Kirkland, S. W.; [illegible]	2015	Canadian Journal of Emergency Medicine	17	NA	S76	10.1017/cem.2015.50	Ospina 2015	Irrelevant	Excluded by TI/AB review
1134	Self-Management and Health Care Use in an Adolescent and Young Adult Medicaid [illegible]	Phillips, G. A.; Fenton, N.; Cohen, S.; Javalkar, K.; Ferris, M.	2015	Preventing chronic disease	12	NA	E103	10.5888/pcd12.150023	Phillips 2015	Irrelevant	Excluded by TI/AB review
1135	Gender and spoken interaction	Pichler, Pia; Eppler, Eva M.	2015	NA	NA	NA	NA	NA	Pichler 2015	Irrelevant	Excluded by TI/AB review

S4 Table. All studies identified in the literature search

1136	Supported self-management for asthma	Pinnock, H.	2015	Breathe	11	2	99-109	10.1183/20734735.015614	Pinnock 2015	Irrelevant	Excluded by TI/AB review
1137	Implementing supported self-management for asthma: A systematic review and suggested hierarchy of [illegible]	Pinnock, H.; Epiphaniou, E.; Pearce, G.; Parke, H.; Greenhalgh, T.; Sheikh, A.; Griffiths, C. J.; Taylor, S. J. C.	2015	BMC Medicine	13	1	NA	10.1186/s12916-015-0361-0	Pinnock 2015	Irrelevant	Excluded by TI/AB review
1138	Do paramedics find it beneficial to learn the diagnosis given to their patients in the emergency department?	Pollard, John; Black, Sarah	2015	Emergency Medicine Journal	32	5	421-421	10.1136/emermed-2015-204877.2	Pollard 2015	Irrelevant	Excluded by TI/AB review
1139	Analgesia for fracture pain in children: methodological	Poonai, N.; Kilgar, J.; Mehrotra, S.	2015	Pain Management	5	6	435-445	10.2217/pmt.15.41	Poonai 2015	Irrelevant	Excluded by TI/AB review

S4 Table. All studies identified in the literature search

1140	Social and Medical Determinants of Cardiometabolic Health: The Big Picture	Puckrein, G. A.; Egan, B. M.; Howard, G.	2015	Ethn Dis	25	4	521-4	10.18865/ed.25.4.521	Puckrein 2015	Irrelevant	Excluded by TI/AB review
1142	A prospective feasibility trial of accucath 2.25 blood control intravascular [illegible]	Raio, C.; Ash, A.; Stankard, B.; Nelson, M.; Modayil, V.; Bajaj, T.; Snock, A.; Wie, B.; [illegible]	2015	Annals of Emergency Medicine	66	4	S137	NA	Raio 2015	Irrelevant	Excluded by TI/AB review
1143	The effectiveness of a patient navigator on patient satisfaction in adult patients in ambulatory care settings: A systematic review [illegible]	Ranaghan, C. P.; Boyle, K.; Fraser, P.; Meehan, M.; Moustapha, S.; Concert, C.	2015	JBI Database of Systematic Reviews and Implementation Reports	13	8	54-69	10.11124/jbisrir-2015-2323	Ranaghan 2015	Irrelevant	Excluded by TI/AB review
1144	Outcomes for Children with Chronic Conditions Associated with Parent- and Provider-reported Measures of the Medical Home	Raphael, J. L.; Cooley, W. C.; Vega, A.; Kowalkowski, M. A.; Tran, X.; Treadwell, J.; Giardino, A. P.; Giordano, T. P.	2015	Journal of Health Care for the Poor and Underserved	26	2	358-376	10.1353/hpu.2015.0051	Raphael 2015	Irrelevant	Excluded by TI/AB review

S4 Table. All studies identified in the literature search

1146	Health Literacy Impact on National Healthcare Utilization and Expenditure	Rasu, R. S.; Bawa, W. A.; Suminski, R.; Snella, K.; Warady, B.	2015	International journal of health policy and management	4	11	747-755	10.15171/ijhpm.2015.151	Rasu 2015	Irrelevant	Excluded by TI/AB review
1147	Tailored approaches to stroke health education (TASHE): study protocol for a randomized controlled trial	Ravenell, J.; Leighton-Herrmann, E.; Abel-Bey, A.; DeSorbo, A.; Teresi, J.; Valdez, L.; Gordillo, M.; Gerin, W.; Hecht, M.; Ramirez, M.; Noble, J.; Cohn, E.; [illegible]	2015	Trials	16	NA	176	10.1186/s13063-015-0703-4	Ravenell 2015	Irrelevant	Excluded by TI/AB review
1149	Brief motivational intervention for intimate partner violence and heavy drinking in the emergency [illegible]	Rhodes, K. V.; Rodgers, M.; Sommers, M.; Hanlon, A.; Chittams, J.; Doyle, A.; Datner, E.; Crits-[illegible]	2015	JAMA - Journal of the American Medical Association	314	5	466-477	10.1001/jama.2015.8369	Rhodes 2015	Irrelevant	Excluded by TI/AB review
1151	Palliative care in advanced lung disease: The challenge of integrating [illegible]	Rocker, G. M.; Simpson, A. C.; Horton, R.	2015	Chest	148	3	801-809	10.1378/chest.14-2593	Rocker 2015	Irrelevant	Excluded by TI/AB review

S4 Table. All studies identified in the literature search

1152	Improving emergency physician performance using audit and feedback: A systematic review and meta-analysis to identify features critical to success	Rogers, R. L.; Narvaez, Y.; Venkatesh, A. K.; Fleischman, W.; Hall, M. K.; Taylor, R. A.; Qureshi, R. A.; Hersey, D.; Sette, L.; Melnick, E. R.	2015	Academic Emergency Medicine	22	5	S61-S62	10.1111/acem.12644	Rogers 2015	Irrelevant	Excluded by TI/AB review
1153	Improving emergency physician performance using audit and feedback: A systematic review	Rogers, R. L. G.; Narvaez, Y.; Venkatesh, A. K.; Fleischman, W.; Hall, M. K.; Taylor, R. A.; Hersey, D.; Sette, L.; Melnick, E. R.	2015	American Journal of Emergency Medicine	33	10	1505-1514	10.1016/j.ajem.2015.07.039	Rogers 2015	Irrelevant	Excluded by TI/AB review

S4 Table. All studies identified in the literature search

1154	Using saline for myofascial pain syndromes	Roldan, C.; Hu, N.	2015	Journal of Pain	16	4	S90	NA	Roldan 2015	Irrelevant	Excluded by TI/AB review
1155	Health Literacy in Patients Seeking Orthopaedic Care: Results of the Literacy in Musculoskeletal [illegible]	Rosenbaum, A. J.; Pauze, D.; Pauze, D.; Robak, N.; Zade, R.; Mulligan, M.; Uhl, R. L.	2015	The Iowa orthopaedic journal	35	NA	187-192	NA	Rosenbaum 2015	Irrelevant	Excluded by TI/AB review
1156	The association between insurance type and cost-related delay in care: A survey of access to care in Massachusetts	Rowas, S. A.; Rothberg, M. B.; Goff, S. L.; Johnson, B.; Miller, J.; Friderici, J.; Lagu, T.	2015	Journal of General Internal Medicine	30	NA	S266	NA	Rowas 2015	Irrelevant	Excluded by TI/AB review

S4 Table. All studies identified in the literature search

1157	Care and outcomes of patients with in-hospital stroke	Saltman, A. P.; Silver, F. L.; Fang, J.; Stamplecoski, M.; Kapral, M. K.	2015	JAMA Neurology	72	7	749-755	10.1001/jamaneurol.2015.0284	Saltman 2015	Irrelevant	Excluded by TI/AB review
1158	‚ÄúSometimes you get to feel like the freak show,Äù: Transgender and gender-non- [illegible]	Samuels, E. A.; Choo, E.; Tape, C.; Garber, N.; Bowman, S.	2015	Annals of Emergency Medicine	66	4	S57	NA	Samuels 2015	Irrelevant	Excluded by TI/AB review
1159	Racial disparities in the surgical care of localized prostate cancer	Schmid, M.; Meyer, C.; Reznor, G.; Hanske, J.; Sammon, J.; Abdollah, F.; Sood, A.; Dalela, D.; [illegible]	2015	Journal of Urology	193	4	e658-e659	NA	Schmid 2015	Irrelevant	Excluded by TI/AB review
1160	Racial disparities in the surgical care of localized prostate cancer	Schmid, M.; Meyer, C. P.; Reznor, G.; Hanske, J.; Sammon, J. D.; Abdollah, F.; Dalela, D.; Kibel, A. S.; Chun, F. K. H.; Menon, M.; Fisch, M.; Sood, A.; Trinh, [illegible]	2015	European Urology, Supplements	14	2	e641-e641a	NA	Schmid 2015	Irrelevant	Excluded by TI/AB review

S4 Table. All studies identified in the literature search

1161	Analgesic Access for Acute Abdominal Pain in the Emergency Department Among Racial/Ethnic [illegible]	Shah, A. A.; Zogg, C. K.; Zafar, S. N.; Schneider, E. B.; Cooper, L. A.; Chapital, A. B.; Peterson, S. M.; Havens, J. M.; [illegible]	2015	Med Care	53	12	1000-9	10.1097/mlr.0000000000000444	Shah 2015	Irrelevant	Excluded by TI/AB review
1163	Who gets early tracheostomy? evidence of unequal treatment at 185 academic medical centers	Shaw, J. J.; Santry, H. P.	2015	Chest	148	5	1242-1250	10.1378/chest.15-0576	Shaw 2015	Irrelevant	Excluded by TI/AB review
1164	Assessing patient activation and health literacy in the emergency department	Sheikh, S.; Hendry, P.; Kalynych, C.; Owensby, B.; Johnson, J.; Carden, D. L.; Kraemer, D. F.	2015	Academic Emergency Medicine	22	5	S97-S98	10.1111/acem.12644	Sheikh 2015	Irrelevant	Excluded by TI/AB review

S4 Table. All studies identified in the literature search

1165	Mind the (knowledge) gap: The effect of a communication instrument on emergency department patients' comprehension of and satisfaction with care	Simmons, S.; Sharp, B.; Fowler, J.; Fowkes, H.; Paz-Arabo, P.; Dilt-Skaggs, M. K.; Singal, B.; Carter, T.	2015	Patient Education and Counseling	98	2	257-262	10.1016/j.pec.2014.10.020	Simmons 2015	Irrelevant	Excluded by TI/AB review
1166	Immigration and schooling: Redefining the 21st century America	Som√©, Touorizou Herv√©; Orelus, Pierre W.	2015	Critical constructions: Studies on education and society	NA	NA	NA	NA	Som√© 2015	Irrelevant	Excluded by TI/AB review

S4 Table. All studies identified in the literature search

1167	The majority of inflammatory bowel disease patients feel that an elimination diet trial had resulted in disease symptom improvement	Stein, D.; Ansari, I.; Cahill, J.; Agrawal, D.; Naik, A. S.; Zadvornova, Y.	2015	Gastroenterology	148	4	S840-S841	NA	Stein 2015	Irrelevant	Excluded by TI/AB review
1168	Effectiveness of housing first with intensive case management in an	Stergiopoulos, V.; Gozdzik, A.; Misir, V.; Skosireva, A.; Connelly, J.; Sarang,	2015	PLoS One	10	7	NA	10.1371/journal.pone.0130281	Stergiopoulos 2015	Irrelevant	Excluded by TI/AB review
1169	Hallway patients reduce overall Emergency Department Satisfaction	Stiffler, K. A.; Wilber, S. T.	2015	Journal of Emergency Medicine	49	2	211-216	10.1016/j.jemermed.2014.05.002	Stiffler 2015	Irrelevant	Excluded by TI/AB review
1170	The benefits of timely palliative care referral	Stine, J. E.; Doll, K. M.; Pierce, S. R.; Sullivan, S. A.; Gehrig, P. A.; Hanson, L.; Kim, K. H.	2015	Gynecologic Oncology	137	NA	50-51	10.1016/j.ygyno.2015.01.115	Stine 2015	Irrelevant	Excluded by TI/AB review

S4 Table. All studies identified in the literature search

1171	'Underclassism' and access to healthcare in urban centres	Tang, S. Y.; Browne, A. J.; Mussell, B.; Smye, V. L.; Rodney, P.	2015	Sociology of Health & Illness	37	5	698-714	10.1111/1467-9566.12236	Tang 2015	Irrelevant	Excluded by TI/AB review
1173	The specific psychological needs of older emergency department patients	Taylor, D.; Liu, B.; Liversidge, X.; Ling, S.; MacGibbon, P.	2015	EMA - Emergency Medicine Australasia	27	NA	49-50	10.1111/1742 6723.12416	Taylor 2015	Irrelevant	Excluded by TI/AB review
1174	Commentary on "Factors associated with emergency department visits in asthma exacerbation"	Trevor, J.; Dransfield, M.	2015	Southern Medical Journal	108	5	281-282	10.14423/SMJ.0000000000000276	Trevor 2015	Irrelevant	Excluded by TI/AB review
1175	Mental health in South Asia: Ethics, resources, programs and legislation, 2nd ed	Trivedi, Jitendra Kumar; Tripathi, Adarsh	2015	International library of ethics, law, and the new medicine; Vol 58; ISSN: 1567- [illegible]	58	NA	NA	10.1007/978-94-017-9017-8	Trivedi 2015	Irrelevant	Excluded by TI/AB review

S4 Table. All studies identified in the literature search

1176	Use of colocated multidisciplinary services to address family psychosocial needs at an urban pediatric primary care clinic	Vasan, A.; Solomon, B. S.	2015	Clinical Pediatrics	54	1	25-32	10.1177/0009922814541802	Vasan 2015	Irrelevant	Excluded by TI/AB review
1177	Gender and racial differences in high density lipoprotein levels in acute coronary syndromes	Velarde, G. P.; Bravo, K.; Ozaki, A.; Smotherman, C.; Kraemer, D.; Dodani, S.; Joseph, A.	2015	Journal of the American College of Cardiology	65	10	A95	NA	Velarde 2015	Irrelevant	Excluded by TI/AB review
1178	Increased patient satisfaction and decreased length of stay in an emergency department observation unit compared to inpatient [illegible]	Ventura, A. L.; Schaninger, C.; Hart, K. W.; Lindsell, C. J.; Miller, C. N.	2015	Academic Emergency Medicine	22	5	S184	10.1111/acem.12644	Ventura 2015	Irrelevant	Excluded by TI/AB review

S4 Table. All studies identified in the literature search

1179	Ethnic Disparities in Emergency Severity Index Scores among U.S. Veteran's Affairs Emergency Department Patients	Vigil, J. M.; Alcock, J.; Coulombe, P.; McPherson, L.; Parshall, M.; Murata, A.; Brislen, H.	2015	PLoS One	10	5	e0126792	10.1371/journal.pone.0126792	Vigil 2015	Irrelevant	Excluded by TI/AB review
1182	Protocol for suspected pediatric appendicitis limits computed tomography utilization	Wagenaar, A. E.; Tashiro, J.; Wang, B.; Curbelo, M.; Mendelson, K. L.; Perez, E. A.; Hogan, A. R.; Neville, H. L.; Sola, J. E.	2015	Journal of Surgical Research	199	1	153-158	10.1016/j.jss.2015.04.028	Wagenaar 2015	Irrelevant	Excluded by TI/AB review

S4 Table. All studies identified in the literature search

1183	Health outcomes in US children with abdominal pain at major emergency departments associated with race and socioeconomic status	Wang, L.; Haberland, C.; Thurm, C.; Bhattacharya, J.; Park, K. T.; Abe, T.	2015	PLoS One	10	8	NA	10.1371/journal.pone.0132758	Wang 2015	Irrelevant	Excluded by TI/AB review
1184	Tea consumption and health-related quality of life in elderly adults	Wang, P.	2015	Value in Health	18	7	A745	NA	Wang 2015	Irrelevant	Excluded by TI/AB review
1185	The psyche in the modern world: Psychotherapy and society	Warnecke, Tom	2015	United Kingdom Council for Psychotherapy series	NA	NA	NA	NA	Warnecke 2015	Irrelevant	Excluded by TI/AB review

S4 Table. All studies identified in the literature search

1186	Emergency department use: a reflection of poor primary care access?	Weisz, D.; Gusmano, M. K.; Wong, G.; Trombley, J.	2015	The American journal of managed care	21	2	e152-e160	NA	Weisz 2015	Irrelevant	Excluded by TI/AB review
1187	Do emergency department patients receive a pathological diagnosis? A nationally-representative sample	Wen, L. S.; Espinola, J. A.; Mosowsky, J. M.; Camargo, C. A.	2015	Western Journal of Emergency Medicine	16	1	50-54	10.5811/westjem.2014.12.23474	Wen 2015	Irrelevant	Excluded by TI/AB review
1188	High-sensitivity troponin assays for the early rule-out or diagnosis of acute myocardial infarction in people with acute chest pain: A systematic review [illegible]	Westwood, M.; Van Asselt, T.; Ramaekers, B.; Whiting, P.; Thokala, P.; Joore, M.; Armstrong, N.; Ross, J.; Severens, J.; Kleijnen, J.	2015	Health Technology Assessment	19	44	1-234	10.3310/hta19440	Westwood 2015	Irrelevant	Excluded by TI/AB review

S4 Table. All studies identified in the literature search

1189	Impact of highdeductible insurance on diabetes outpatient and high acuity care: The natural [illegible]	Wharam, J. F.; Zhang, F.; Eggleston, E.; Lu, C.; Soumerai, S. B.; Ross-Degnan, D.	2015	Journal of General Internal Medicine	30	NA	S181	NA	Wharam 2015	Irrelevant	Excluded by TI/AB review
1190	Review article: Emergency department models of care in the context of care quality and cost: A systematic review	Wylie, K.; Crilly, J.; Toloo, G.; Fitzgerald, G.; Burke, J.; Williams, G.; Bell, A.	2015	EMA - Emergency Medicine Australasia	27	2	95-101	10.1111/1742-6723.12367	Wylie 2015	Irrelevant	Excluded by TI/AB review
1192	Targeted therapy at the end-of-life and the impact on healthcare utilization	Yeung, H.; Murphy, J.; Salehi, P.	2015	Journal of Pain and Symptom Management	49	2	463-464	NA	Yeung 2015	Irrelevant	Excluded by TI/AB review
1193	Normal prehospital electrocardiography is linked to long-term survival in patients presenting [illegible]	Z√®gre-Hemsey, J. K.; Sommargren, C. E.; Asafu-Adjei, J. K.; Drew, B. J.	2015	Journal of Electrocardiology	48	4	520-526	10.1016/j.jelectrocard.2015.01.014	Z√®gre-Hemsey 2015	Irrelevant	Excluded by TI/AB review

S4 Table. All studies identified in the literature search

1194	Adherence to highly active antiretroviral therapy impact on clinical and economic outcomes for [illegible]	Zhang, S.; Rust, G.; Cardarelli, K.; Felizzola, J.; Fransua, M.; Stringer, H. G.	2015	AIDS Care - Psychological and Socio-Medical Aspects of AIDS/HIV	27	7	829-835	10.1080/09540121.2015.1021745	Zhang 2015	Irrelevant	Excluded by TI/AB review
1195	Acute care utilization in patients with concurrent behavioral health and complex chronic medical [illegible]	Abernathy, K.; Zhang, J.; Brownfield, E. L.; Mauldin, P. D.; Moran, W. P.; Davis, K. S.	2016	Journal of General Internal Medicine	31	2	S112	NA	Abernathy 2016	Irrelevant	Excluded by TI/AB review
1196	Association between diabetes knowledge and clinical outcomes in children with T1DM	Adamidis, A.; Gudala, M.; Knee, A.; Allen, H. F.; Moskovitz, A.; Tonyushkina, K. N.	2016	Endocrine Reviews	37	2	NA	10.1210/endo-meetings.2016.DGM.19.SAT-711	Adamidis 2016	Irrelevant	Excluded by TI/AB review
1197	Gender differences and characteristics of psychiatric patients assessed in the emergency department of a [illegible]	Agyapong, V.; Juhas, M.; Ritchie, A.; Kavanagh, A.	2016	European Psychiatry	33	NA	S439-S440	10.1016/j.eurpsy.2016.01.1598	Agyapong 2016	Irrelevant	Excluded by TI/AB review

S4 Table. All studies identified in the literature search

1198	Race, Medical Mistrust, and Segregation in Primary Care as Usual Source of Care: Findings from the Exploring Health Disparities in Integrated Communities	Arnett, M. J.; Thorpe, R. J.; Gaskin, D. J.; Bowie, J. V.; LaVeist, T. A.	2016	Journal of Urban Health-Bulletin of the New York Academy of Medicine	93	3	456-467	10.1007/s11524-016-0054-9	Arnett 2016	Irrelevant	Excluded by TI/AB review
1199	Ethnicity and pre-hospital care for people with suspected cardiac pain: cross-sectional study	Asghar, Z.; Phung, V. H.; Siriwardena, A. N.	2016	J Eval Clin Pract	22	5	721-5	10.1111/jep.12523	Asghar 2016	Irrelevant	Excluded by TI/AB review
1201	Access to the VA: Which veterans are seeking primary care services?	Augustine, M. R.; Nelson, K. M.; Fihn, S. D.; Wong, E.	2016	Journal of General Internal Medicine	31	2	S111	NA	Augustine 2016	Irrelevant	Excluded by TI/AB review

S4 Table. All studies identified in the literature search

1202	Misidentification of English Language Proficiency in Triage: Impact on Satisfaction and Door-to-Room [illegible]	Balakrishnan, V.; Roper, J.; Cossey, K.; Roman, C.; Jeanmonod, R.	2016	Journal of immigrant and minority health	18	2	369-373	10.1007/s10903-015-0174-4	Balakrishnan 2016	Irrelevant	Excluded by TI/AB review
1203	Technology-Based Interventions for Asthma,ÄîCan They Help Decrease Health Disparities?	Baptist, A. P.; Islam, N.; Joseph, C. L. M.	2016	Journal of Allergy and Clinical Immunology: In Practice	4	6	1135-1142	10.1016/j.jaip.2016.04.024	Baptist 2016	Irrelevant	Excluded by TI/AB review
1205	Cynicism and other attitudes towards patients in an emergency department in a [illegible]	Batley, N. J.; Nasreddine, Z.; Chami, A.; Zebian, D.; Bachir, R.; Abbas, H. A.	2016	BMC medical education	16	NA	36	10.1186/s12909-016-0539-y	Batley 2016	Irrelevant	Excluded by TI/AB review
1206	Implementation of a risk stratifcation and management pathway for acute chest pain in the [illegible]	Baugh, C. W.; Greenberg, J. O.; Mahler, S. A.; Kosowsky, J. M.; Schuur, J. D.; [illegible]	2016	Critical Pathways in Cardiology	15	4	131-137	10.1097/HPc.0000000000000095	Baugh 2016	Irrelevant	Excluded by TI/AB review
1207	Implementation of a Risk Stratification and Management Pathway for Acute [illegible]	Baugh, Christopher W.; Greenberg, Jeffrey O.; Mahler, Simon A.; Kosowsky, Joshua [illegible]	2016	Critical Pathways in Cardiology	15	4	131-137	NA	Baugh 2016	Irrelevant	Excluded by TI/AB review

S4 Table. All studies identified in the literature search

1209	Emergency Department Frequent Utilization for Non-Emergent Presentments: Results from a Regional Urban Trauma Center Study	Behr, J. G.; Diaz, R.	2016	PLoS One	11	1	NA	10.1371/journal.pone.0147116	Behr 2016	Irrelevant	Excluded by TI/AB review
1210	Public Health, Hypertension, and the Emergency Department	Brody, A.; Janke, A.; Sharma, V.; Levy, P.	2016	Current Hypertension Reports	18	6	7-Jan	10.1007/s11906-016-0654-5	Brody 2016	Irrelevant	Excluded by TI/AB review

S4 Table. All studies identified in the literature search

1211	A randomized controlled study of silver-based burns dressing in a pediatric emergency department	Brown, M.; Dalziel, S. R.; Herd, E.; Johnson, K.; Wong She, R.; Shepherd, M.	2016	Journal of Burn Care and Research	37	4	e340-e347	10.1097/BCR.0000000000000273	Brown 2016	Irrelevant	Excluded by TI/AB review
1212	The use and limits of eye-tracking in high-fidelity clinical scenarios: A pilot study	Browning, Mark; Cooper, Simon; Cant, Robyn; Sparkes, Louise; Bogossian, Fiona; Williams, Brett; O'Meara, Peter; Ross, Linda; Munro, Graham; Black, Barbara	2016	International emergency nursing	25	NA	43-47	10.1016/j.ienj.2015.08.002	Browning 2016	Irrelevant	Excluded by TI/AB review
1213	The postpartum visit: Predictors, disparities and opportunities	Bryant, A.; Blake-Lamb, T.; Hatoum, I.; Kotelchuck, M.	2016	Obstetrics and Gynecology	127	NA	74S-75S	10.1097/01.AOG.0000483710.53534.56	Bryant 2016	Irrelevant	Excluded by TI/AB review

S4 Table. All studies identified in the literature search

1214	Impact of a best practice alert on transfusion practices for solid tumor oncology	Buck, R.; Wachsberg, K.; Weaver, C.; Dombrowski, L.; Ma, M.; Lee, J.;	2016	Journal of Clinical Oncology	34	7	NA	NA	Buck 2016	Irrelevant	Excluded by TI/AB review
1215	Family-Centered Pediatric Emergency Care: A Framework for Measuring What Parents Want and Value	Byczkowski, T. L.; Gillespie, G. L.; Kennebeck, S. S.; Fitzgerald, M. R.; Downing, K. A.; Alessandrini, E. A.	2016	Academic Pediatrics	16	4	327-335	10.1016/j.acap.2015.08.011	Byczkowski 2016	Irrelevant	Excluded by TI/AB review
1217	Medication Adherence With Diabetes Medication: A Systematic Review of the Literature	Capoccia, K.; Odegard, P. S.; Letassy, N.	2016	The Diabetes educator	42	1	34-71	10.1177/0145721715619038	Capoccia 2016	Irrelevant	Excluded by TI/AB review

S4 Table. All studies identified in the literature search

1218	Reasons for Frequent Emergency Department Use by Medicaid Enrollees: A Qualitative Study	Capp, Roberta; Kelley, Lauren; Ellis, Peter; Carmona, Juan; Lofton, Adrienne; Cobbs,ÄêLomax, Darcey; D'Onofrio, Gail; Meisel, Zachary F.	2016	Academic Emergency Medicine	23	4	476-481	10.1111/acem.12952	Capp 2016	Irrelevant	Excluded by TI/AB review
1219	Development of a health disparities curriculum for an academic emergency medicine residency program	Caretta-Weyer, H. A.; Hess, J. M.	2016	Academic Emergency Medicine	23	NA	S289	10.1111/acem.12975	Caretta-Weyer 2016	Irrelevant	Excluded by TI/AB review
1220	Characteristics and outcomes of hospitalized community dwelling geriatric patients discharged to a skilled nursing facility	Carnahan, J. L.; Callahan, C. M.; Ambuehl, R.; Torke, A. M.	2016	Journal of General Internal Medicine	31	2	S152-S153	NA	Carnahan 2016	Irrelevant	Excluded by TI/AB review

S4 Table. All studies identified in the literature search

1221	Improved emergency department quality metrics, patient satisfaction scores, and revenue following implementation of lean flow principles and queuing theory-based operational changes	Carstairs, K. L.; Hollenbach, K. A.; Shah, S.; Putnam, K. L.; Weber, R.; Black, I.; McDonough, L.; Kaufman, I.; Davis, C. B.	2016	Academic Emergency Medicine	23	NA	S59-S60	10.1111/acem.12974	Carstairs 2016	Irrelevant	Excluded by TI/AB review
1222	Shared Decision Making With Vulnerable Populations in the Emergency Department	Castaneda-Guarderas, A.; Glassberg, J.; Grudzen, C. R.; Ngai, K. M.; Samuels-Kalow, M. E.; Shelton, E.; Wall, S. P.; Richardson, L. D.	2016	Academic Emergency Medicine	23	12	1410-1416	10.1111/acem.13134	Castaneda-Guarderas 2016	Irrelevant	Excluded by TI/AB review

S4 Table. All studies identified in the literature search

1224	Prospective randomized pilot trial measuring the feasibility and knowledge retention of opioid education in emergency [illegible]	Chakravarthy, B.; Somasundaram, S.; Mogi, J.; Burns, R.; Hoonpongsimanont, W.; Lotfipour, S.	2016	Annals of Emergency Medicine	68	4	S81	NA	Chakravarthy 2016	Irrelevant	Excluded by TI/AB review
1225	Investigation of racial disparities in early supportIVe medication use and end-of-life care among medicare beneficiaries with [illegible]	Check, D. K.; Samuel, C. A.; Rosenstein, D. L.; Dusetzina, S. B.	2016	Journal of Clinical Oncology	34	19	2265-2270	10.1200/JCO.2015.64.8162	Check 2016	Irrelevant	Excluded by TI/AB review
1228	Sex differences in STEMI activation for patients presenting to the ED 1939	Choi, Katherine; Shofer, Frances S.; Mills, Angela M.	2016	American Journal of Emergency Medicine	34	10	1939-1943	10.1016/j.ajem.2016.06.082	Choi 2016	Irrelevant	Excluded by TI/AB review
1229	"I Was a Spectacle... A Freak Show at the Circus": A Transgender Person's ED Experience and Implications for Nursing Practice	Cicero, E. C.; Perry Black, B.	2016	Journal of emergency nursing: JEN : official publication of the Emergency Department [illegible]	42	1	25-30	10.1016/j.jen.2015.08.012	Cicero 2016	Irrelevant	Excluded by TI/AB review

S4 Table. All studies identified in the literature search

1230	A Parent coach model for well-child care among low-income children: A randomized controlled trial	Coker, T. R.; Chacon, S.; Elliott, M. N.; Bruno, Y.; Chavis, T.; Biely, C.; Bethell, C. D.; Contreras, S.; Mimila, N. A.; Mercado, J.; Chung, P. J.	2016	Pediatrics	137	3	NA	10.1542/peds.2015-3013	Coker 2016	Irrelevant	Excluded by TI/AB review
1231	Association between medicare accountable care organization implementation and spending among clinically vulnerable beneficiaries	Colla, C. H.; Lewis, V. A.; Kao, L. S.; O'Malley, A. J.; Chang, C. H.; Fisher, E. S.	2016	JAMA Internal Medicine	176	8	1167-1175	10.1001/jamainternmed.2016.2827	Colla 2016	Irrelevant	Excluded by TI/AB review
1232	Public Perception of Emergency Medical Services in the United States	Crowe, Remle P.; Levine, Roger; Rodriguez, Severo; Larrimore, Ashley D.; Pirrallo, Ronald G.	2016	Prehospital & Disaster Medicine	31	NA	S112-S117	10.1017/S1049023X16001126	Crowe 2016	Irrelevant	Excluded by TI/AB review

S4 Table. All studies identified in the literature search

1233	Pelvic inflammatory disease: improving awareness, prevention, and treatment	Das, B. B.; Ronda, J.; Trent, M.	2016	Infection and Drug Resistance	9	NA	191-197	10.2147/IDR.S91260	Das 2016	Irrelevant	Excluded by TI/AB review
1234	Integrating palliative care in outpatient hematology clinic increases patient satisfaction, adherence to care [illegible]	Draliuk, R.; Perez, E.; Yosipovich, A.; Preis, M.	2016	Blood	128	22	NA	NA	Draliuk 2016	Irrelevant	Excluded by TI/AB review
1235	Patient navigation for patients frequently visiting the emergency department	Elseroad, S.; Seaberg, D.; Dumas, M.; Mendiratta, S.; Whittle, J.; Holcombe, J.; Glisson, C.	2016	Academic Emergency Medicine	23	NA	S220	10.1111/acem.12974	Elseroad 2016	Irrelevant	Excluded by TI/AB review

S4 Table. All studies identified in the literature search

1236	Acculturation and quality of life in urban, African American caregivers of children with asthma	Everhart, R. S.; Miadich, S. A.; Leibach, G. G.; Borschuk, A. P.; Koinis-Mitchell, D.	2016	J Asthma	53	9	983-8	10.3109/02770903.2016.1167904	Everhart 2016	Irrelevant	Excluded by TI/AB review
1239	Language Matters Race, Trust, and Outcomes in the Pediatric Emergency Department	Fields, A.; Abraham, M.; Gaughan, J.; Haines, C.; Hoehn, K. S.	2016	Pediatric Emergency Care	32	4	222-226	10.1097/PEC.0000000000000453	Fields 2016	Irrelevant	Excluded by TI/AB review
1240	Treating vulnerable populations of cancer survivors: A biopsychosocial approach	Fitzpatrick, Tanya R.	2016	NA	NA	NA	NA	10.1007/978-3-319-32364-0	Fitzpatrick 2016	Irrelevant	Excluded by TI/AB review

S4 Table. All studies identified in the literature search

1241	Language barrier affect on providing timely pain management for long bone fractures in a pediatric emergency Department	Gaba, M.; Vazquez, H.; Thompson, J.; See, F.; Rizkalla, C.	2016	Academic Emergency Medicine	23	NA	S132-S133	10.1111/acem.12974	Gaba 2016	Irrelevant	Excluded by TI/AB review
1242	Practice Variation in Management of Childhood Asthma Is Associated with Outcome Differences	Garbutt, J. M.; Yan, Y.; Strunk, R. C.	2016	Journal of Allergy and Clinical Immunology: In Practice	4	3	474-480	10.1016/j.jaip.2015.12.011	Garbutt 2016	Irrelevant	Excluded by TI/AB review
1244	Implementing Sexual Orientation and Gender Identity Data Collection in	German, D.; Kodadek, L.; Shields, R.; Peterson, S.; Snyder, C.; Schneider, E.; Vail,	2016	LGBT Health	3	6	416-423	10.1089/lgbt.2016.0069	German 2016	Irrelevant	Excluded by TI/AB review

S4 Table. All studies identified in the literature search

1248	Uninsured status may be more predictive of outcomes among the severely injured than minority race	Gerry, J. M.; Weiser, T. G.; Spain, D. A.; Staudenmayer, K. L.	2016	Injury	47	1	197-202	10.1016/j.injury.2015.09.003	Gerry 2016	Irrelevant	Excluded by TI/AB review
1249	Root Cause Analysis of Ambulatory Adverse Drug Events That Present to the Emergency Department	Gertler, S. A.; Coralic, Z.; L√≥pez, A.; Stein, J. C.; Sarkar, U.	2016	Journal of patient safety	12	3	119-124	10.1097/PTS.0000000000000072	Gertler 2016	Irrelevant	Excluded by TI/AB review
1250	The impact of bariatric surgery on the erectile function. Systematic review	Glina, F.; De Freitas Barbosa, J. W.; Nunes, V. M.; Glina, S.; Bernardo, W. M.	2016	Journal of Urology	195	4	e642-e643	NA	Glina 2016	Irrelevant	Excluded by TI/AB review

S4 Table. All studies identified in the literature search

1251	Lost in translation: impact of language barriers on children's healthcare	Goenka, P. K.	2016	Current Opinion in Pediatrics	28	5	659-666	10.1097/MOP.0000000000000000404	Goenka 2016	Irrelevant	Excluded by TI/AB review
1252	Identifying Victims of Human Trafficking in the Emergency Department	Greenbaum, J.	2016	Clinical Pediatric Emergency Medicine	17	4	241-248	10.1016/j.cpem.2016.09.006	Greenbaum 2016	Irrelevant	Excluded by TI/AB review
1253	The 2016 Academic Emergency Medicine Consensus [illegible]	Grudzen, C. R.; Anderson, J. R.; Carpenter, C. R.; Hess, E. P.	2016	Academic Emergency Medicine	23	12	1313-1319	10.1111/acem.13047	Grudzen 2016	Irrelevant	Excluded by TI/AB review
1254	Factors Associated With the Likelihood of Hospitalization Following Emergency Department Visits for Behavioral Health Conditions	Hamilton, J. E.; Desai, P. V.; Hoot, N. R.; Gearing, R. E.; Jeong, S.; Meyer, T. D.; Soares, J. C.; Begley, C. E.	2016	Acad Emerg Med	23	11	1257-1266	10.1111/acem.13044	Hamilton 2016	Irrelevant	Excluded by TI/AB review

S4 Table. All studies identified in the literature search

1258	Dietary energy density and postmenopausal breast cancer incidence in the cancer prevention study II nutrition cohort	Hartman, T. J.; Gapstur, S. M.; Gaudet, M. M.; Shah, R.; Flanders, W. D.; Wang, Y.; McCullough, M. L.	2016	Journal of Nutrition	146	10	2045-2050	10.3945/jn.116.234344	Hartman 2016	Irrelevant	Excluded by TI/AB review
1260	Triage sepsis alert and sepsis protocol lower times to fluids and antibiotics in the ED	Hayden, G. E.; Tuuri, R. E.; Scott, R.; Losek, J. D.; Blackshaw, A. M.; Schoenling, A. J.; Nietert, P. J.; Hall, G. A.	2016	American Journal of Emergency Medicine	34	1	9-Jan	10.1016/j.ajem.2015.08.039	Hayden 2016	Irrelevant	Excluded by TI/AB review
1261	The use of inhaled nitrous oxide for analgesia in adult ED patients: A pilot study	Herres, J.; Chudnofsky, C. R.; Manur, R.; Damiron, K.; Deitch, K.	2016	American Journal of Emergency Medicine	34	2	269-273	10.1016/j.ajem.2015.10.038	Herres 2016	Irrelevant	Excluded by TI/AB review

S4 Table. All studies identified in the literature search

1262	Involving patients with low-risk chest pain in discharge decisions: A multicenter trial	Hess, E. P.; Hollander, J. E.; Schaeffer, J. T.; Kline, J. A.; Torres, C. A.; Diercks, D. B.; Jones, R.; Shah, N. D.; Branda, M. E.; Herrin, J.; Castaneda-Guarderas, A.; Anderson, J.; Demers, M.; Leblanc, A.; Montori, V. M.	2016	Academic Emergency Medicine	23	NA	S117-S118	10.1111/acem.12974	Hess 2016	Irrelevant	Excluded by TI/AB review
1263	Effect of hospital safety-net burden on cost and outcomes after surgery	Hoehn, R. S.; Wima, K.; Vestal, M. A.; Weilage, D. J.; Hanseman, D. J.; Abbott, D. E.; Shah, S. A.	2016	JAMA Surgery	151	2	120-128	10.1001/jamasurg.2015.3209	Hoehn 2016	Irrelevant	Excluded by TI/AB review
1264	A Prospective Evaluation of Shared Decision-making Regarding Analgesics Selection for Older Emergency Department Patients with [illegible]	Holland, W. C.; Hunold, K. M.; Mangipudi, S. A.; Rittenberg, A. M.; Yosipovitch, N.; Platts-Mills, T. F.	2016	Academic Emergency Medicine	23	3	306-314	10.1111/acem.12888	Holland 2016	Irrelevant	Excluded by TI/AB review

S4 Table. All studies identified in the literature search

1265	Association between Physician Teamwork and Health System Outcomes after Coronary Artery Bypass Grafting	Hollingsworth, J. M.; Funk, R. J.; Garrison, S. A.; Owen-Smith, J.; Kaufman, S. A.; Pagani, F. D.; Nallamothu, B. K.	2016	Circulation: Cardiovascular Quality and Outcomes	9	6	641-648	10.1161/CIRCOUTCOMES.116.002714	Hollingsworth 2016	Irrelevant	Excluded by TI/AB review
1266	Baseline patient characteristics and early impact of collaborative depression care management on chronic disease outcomes in an [illegible]	Huang, I.; Rodriguez, G. M.; Small, K.; Peccoralo, L.	2016	Journal of General Internal Medicine	31	2	S134-S135	NA	Huang 2016	Irrelevant	Excluded by TI/AB review
1267	Ethnicity-based differences in the actions taken when patients decide that they may have symptoms of acute coronary syndrome	Iacoe, E.; Ratner, P.; Wong, S.; Mackay, M.	2016	NA	32	(Iacoe E.; Mackay M.) Providence Health [illegible]	S320	NA	Iacoe 2016	Irrelevant	Excluded by TI/AB review

S4 Table. All studies identified in the literature search

1268	Improving mental health care transitions for children and youth: a protocol to implement and evaluate an emergency [illegible]	Jabbour, M.; Reid, S.; Polihronis, C.; Cloutier, P.; Gardner, W.; Kennedy, A.; Gray, C.; Zemek, R.; Pajer, K.; Barrowman, N.; Cappelli, M.	2016	Implementation science : IS	11	1	90	10.1186/s13012-016-0456-9	Jabbour 2016	Irrelevant	Excluded by TI/AB review
1269	What strategies may be effective to address erectile dysfunction following treatment of prostate cancer in men of color?	Jeames, S.; Imholte, S.	2016	Cancer Epidemiology Biomarkers and Prevention	25	3	NA	10.1158/1538-7755.DISP15-C13	Jeames 2016	Irrelevant	Excluded by TI/AB review
1270	The Impact of Cognitive Stressors in the Emergency Department on Physician Implicit [illegible]	Johnson, T. J.; Hickey, R. W.; Switzer, G. E.; Miller, E.; Winger, D. G.; Nguyen, M.; Saladino, R. A.; [illegible]	2016	Acad Emerg Med	23	3	297-305	10.1111/acem.12901	Johnson 2016	Irrelevant	Excluded by TI/AB review

S4 Table. All studies identified in the literature search

1274	Association between the Shorter Stays in ED Target and the Quality of ED Discharge Summaries	Jones, P.; Wimsett, J.; Harper, A.	2016	EMA - Emergency Medicine Australasia	28	NA	27-28	10.1111/1742-6723.12614	Jones 2016	Irrelevant	Excluded by TI/AB review
1275	Organizational interventions improving access to community-based primary health care for vulnerable populations: a scoping review	Khanassov, V.; Pluye, P.; Descoteaux, S.; Haggerty, J. L.; Russell, G.; Gunn, J.; Levesque, J. F.	2016	International Journal for Equity in Health	15	1	Jan-34	10.1186/s12939-016-0459-9	Khanassov 2016	Irrelevant	Excluded by TI/AB review
1276	The contribution of motor vehicle emissions to ambient fine particulate matter public health impacts in New York City: A [illegible]	Kheirbek, I.; Haney, J.; Douglas, S.; Ito, K.; Matte, T.	2016	Environmental Health: A Global Access Science Source	15	1	NA	10.1186/s12940-016-0172-6	Kheirbek 2016	Irrelevant	Excluded by TI/AB review
1277	Characteristics of sickle cell patients with frequent ED visits and hospitalizations: Demographic, [illegible]	Kidwell, K. M.; Albo, C.; Pope, M.; Bowman, L.; Xu, H.; Wells, L.; Barrett, N.; Fields, S.; Bora, P.; Patel, N.; Allison, A.; [illegible]	2016	Blood	128	22	NA	NA	Kidwell 2016	Irrelevant	Excluded by TI/AB review

S4 Table. All studies identified in the literature search

1278	Quality improvement project: Implementation of an evidence-based treatment protocol for managing [illegible]	Kindall, T. L.; Turnham, N.	2016	Ochsner Journal	16	1	e49-e50	NA	Kindall 2016	Irrelevant	Excluded by TI/AB review
1279	School-based health centers to advance health equity: A community guide systematic review	Knopf, J. A.; Finnie, R. K. C.; Peng, Y.; Hahn, R. A.; Truman, B. I.; Vernon-Smiley, M.; Johnson, V. C.; Johnson, R. L.; Fielding, J. E.; [illegible]	2016	American Journal of Preventive Medicine	51	1	114-126	10.1016/j.amepre.2016.01.009	Knopf 2016	Irrelevant	Excluded by TI/AB review
1280	Factors that influence penile implant satisfaction from the Propper study	Kohler, T.; Bella, A.; Karpman, E.; Brant, W.; Kansas, B.; Christine, B.; Jones, L.; Khera, M.; Bennett, N.; Rhee, E.; Henry, G.	2016	Journal of Urology	195	4	e643	NA	Kohler 2016	Irrelevant	Excluded by TI/AB review
1281	Influential factors for penile implant patient satisfaction at 1 year from the propper registry	Kohler, T.; Henry, G.; Bella, A.; Brant, W.; Christine, B.; Bennett, N.; Khera, M.; Kansas, B.; [illegible]	2016	Journal of Sexual Medicine	13	5	S30-S31	NA	Kohler 2016	Irrelevant	Excluded by TI/AB review

S4 Table. All studies identified in the literature search

1282	Will she recover? a prolonged hospitalization for a comatose female with neuropsychiatric lupus	Kumar, S.; Geier, C.; Catalanotti, J.	2016	Journal of General Internal Medicine	31	2	S793	NA	Kumar 2016	Irrelevant	Excluded by TI/AB review
1283	Understanding health-care access and utilization disparities among Latino children in the United States	Langellier, B. A.; Chen, J.; Vargas-Bustamante, A.; Inkelas, M.; Ortega, A. N.	2016	Journal of child health care : for professionals working with children in the hospital [illegible]	20	2	133-144	10.1177/1367493514555587	Langellier 2016	Irrelevant	Excluded by TI/AB review
1284	Perceptions of emergency care by sexual and gender minorities in Colorado: Assessing barriers, quality, and factors affecting identity disclosure	LaPlant, W.; Kattari, L.; Zhan, J.; Druck, J.	2016	Academic Emergency Medicine	23	NA	S138-S139	10.1111/acem.12974	LaPlant 2016	Irrelevant	Excluded by TI/AB review

S4 Table. All studies identified in the literature search

1285	Provider-to-Provider Communication during Transitions of Care from Outpatient to [illegible]	Luu, N. P.; Pitts, S.; Petty, B.; Sawyer, M. D.; Dennison-Himmelfarb, C.; Boonyasai, R. T.; Maruthur, N. M.	2016	Journal of General Internal Medicine	31	4	417-425	10.1007/s11606-015-3547-4	Luu 2016	Irrelevant	Excluded by TI/AB review
1286	Implementation of an emergency department syphilis and HIV point-of-care screening process during an outbreak of syphilis in [illegible]	Manteuffel, J.; Markowitz, N.; Ham, D. C.; Slezak, M.; Perrotta, G.; Peters, P. J.; Mumby, K.; Dankerlui, D.; Fakile, Y.; Hoover, K. W.	2016	Annals of Emergency Medicine	68	4	S148	NA	Manteuffel 2016	Irrelevant	Excluded by TI/AB review
1287	Palliative Care in the Emergency Department	McEwan, A.; Silverberg, J. Z.	2016	Emergency Medicine Clinics of North America	34	3	667-685	10.1016/j.emc.2016.04.013	McEwan 2016	Irrelevant	Excluded by TI/AB review
1288	Perceived barriers to accessing mental health services among black and minority ethnic communities: A qualitative study in [illegible]	Memon, A.; Taylor, K.; Mohebati, L. M.; Sundin, J. N.; Cooper, M.; Scanlon, T.; De Visser, R.	2016	The Lancet	388	SPEC.ISS 1	76	NA	Memon 2016	Irrelevant	Excluded by TI/AB review

S4 Table. All studies identified in the literature search

1289	Improved adherence to surviving sepsis: Use of a guideline for patients with pediatric intestinal failure, fever and a central line	Mezoff, E. A.; Hariharan, S.; Dandoy, C. E.; Zhang, Y.; Chiarenzelli, J.; Troutt, M.; Simpkins, J.; Dewald, M.; Klotz, K.; Fei, L.; [illegible]	2016	Journal of Pediatric Gastroenterology and Nutrition	63	NA	S268	10.1097/01.mpg.0000503536.79797.66	Mezoff 2016	Irrelevant	Excluded by TI/AB review
1290	Eliminating inequities for women with disabilities: An agenda for health and wellness	Miles-Cohen, Shari E.; Signore, Caroline	2016	NA	NA	NA	NA	10.1037/14943-000	Miles-Cohen 2016	Irrelevant	Excluded by TI/AB review
1291	The self-regulation of prejudice	Monteith, Margo J.; Parker, Laura R.; Burns, Mason D.; Nelson, Todd D.	2016	Handbook of prejudice, stereotyping, and discrimination., 2nd ed.	NA	NA	409-432	NA	Monteith 2016	Irrelevant	Excluded by TI/AB review
1292	Cultural barriers to medication adherence in epilepsy	Montouris, G.; Hohler, A. D.	2016	CONTINUUM Lifelong Learning in Neurology	22	NA	266-269	10.1212/CON.0000000000000286	Montouris 2016	Irrelevant	Excluded by TI/AB review

S4 Table. All studies identified in the literature search

1293	Admissions after discharge from an emergency department for chest symptoms	Moore, B. J.; Coffey, R. M.; Heslin, K. C.; Moy, E.	2016	Diagnosis	3	3	103-113	10.1515/dx-2016-0014	Moore 2016	Irrelevant	Excluded by TI/AB review
1294	Perceptions of physicians, residents, nurses, and medical office assistants following implementation of screening, brief intervention, and referral to treatment: A multi-site review	Morley, J.; Kapoor, S.; Auerbach, M.; Kwon, N.; Morgenstern, J.; Conigliaro, J.	2016	Journal of General Internal Medicine	31	2	S336-S337	NA	Morley 2016	Irrelevant	Excluded by TI/AB review
1295	Cross-Cultural Telepsychiatry: An Innovative Approach to Assess and Treat Ethnic Minorities with Limited [illegible]	Mucic, D.	2016	Innovation in Medicine and Healthcare 2016	60	NA	49-58	10.1007/978-3-319-39687-3_5	Mucic 2016	Irrelevant	Excluded by TI/AB review

S4 Table. All studies identified in the literature search

1296	Parental decision making associated with pediatric emergency department use for asthma	Mudd, S. S.; Ogborn, C. J.; Bollinger, M. E.; Morphew, T.; Kub, J.; Lewis-Land, C.; Bellin, M. H.; Butz, [illegible]	2016	Annals of Allergy, Asthma and Immunolog y	117	5	490-494	10.1016/j.an ai.2016.08.0 31	Mudd 2016	Irrelevant	Excluded by TI/AB review
1297	Effect of medication adherence on health outcomes in patients with heart failure in a health information exchange	Murray, M.; Seamon, G.; Eckert, G.; Lane, K.; Tu, W.	2016	Pharmacoth erapy	36	12	e264-e265	10.1002/pha r.1877	Murray 2016	Irrelevant	Excluded by TI/AB review
1298	Peer support for achieving independence in diabetes (peeraid): Results of a randomized controlled trial of community health worker assisted self-management support among	Nelson, K. M.; Taylor, L.; Silverman, J. B.; Kiefer, M. M.; Hebert, P.; Lessler, D. S.; Krieger, J.	2016	Journal of General Internal Medicine	31	2	S333-S334	NA	Nelson 2016	Irrelevant	Excluded by TI/AB review

S4 Table. All studies identified in the literature search

1299	The Association Between Limited English Proficiency and Unplanned Emergency Department Revisit Within 72 Hours	Ngai, K. M.; Grudzen, C. R.; Lee, R.; Tong, V. Y.; Richardson, L. D.; Fernandez, A.	2016	Annals of Emergency Medicine	68	2	213-221	10.1016/j.annemergmed.2016.02.042	Ngai 2016	Irrelevant	Excluded by TI/AB review
1301	Patient-centered physical activity coaching in COPD (Walk On!): A study protocol for a pragmatic randomized controlled trial	Nguyen, H. Q.; Bailey, A.; Coleman, K. J.; Desai, S.; Fan, V. S.; Gould, M. K.; Maddock, L.; Miller, K.; Towner, W.; Xiang, A. H.; Moy, M. L.	2016	Contemporary Clinical Trials	46	NA	18-29	10.1016/j.cct.2015.10.010	Nguyen 2016	Irrelevant	Excluded by TI/AB review

S4 Table. All studies identified in the literature search

1302	Association between patient and emergency department operational characteristics and patient satisfaction scores in a pediatric population	Nichol, J. R.; Fu, R.; French, K.; Momberger, J.; Handel, D. A.	2016	Pediatric Emergency Care	32	3	139-141	10.1097/PEC.0000000000000723	Nichol 2016	Irrelevant	Excluded by TI/AB review
1303	School lunch consumption is associated with higher intakes of total fat, added sugars, and sodium in US children	O'Brien, B.; Vernarelli, J. A.	2016	FASEB Journal	30	NA	NA	NA	O'Brien 2016	Irrelevant	Excluded by TI/AB review
1304	Risk factors for admission in emerging adults (18-25) with asthma seeking care in urban emergency [illegible]	Olsen, E.; Ayaz, S. I.; Thomas, C.; Mika, V.; Gibson-Scipio, W.; Millis, S.; Pearson, C.	2016	Annals of Emergency Medicine	68	4	S53-S54	NA	Olsen 2016	Irrelevant	Excluded by TI/AB review

S4 Table. All studies identified in the literature search

1305	Self-Rated Health Among Patients with Coronary Artery Disease Enrolled in a Cardiovascular Risk Reduction Service	Olson, K. L.; Stiefel, M.; Ross, C.; Stadler, S.; Hornak, R.; Sandhoff, B.; Merenich, J. A.	2016	Population Health Management	19	1	24-30	10.1089/pop.2014.0178	Olson 2016	Irrelevant	Excluded by TI/AB review
1306	Needs and fears of young people presenting at accident and emergency [illegible]	Owens, C.; Hansford, L.; Sharkey, S.; Ford, T.	2016	British Journal of Psychiatry	208	3	286-291	10.1192/bjp.bp.113.141242	Owens 2016	Irrelevant	Excluded by TI/AB review
1307	Acute Health Care Utilization among Food-insecure Children in Primary Care Practices	Palakshappa, D.; Khan, S.; Feudtner, C.; Fiks, A. G.	2016	Journal of Health Care for the Poor and Underserved	27	3	1143-1158	10.1353/hpu.2016.0127	Palakshappa 2016	Irrelevant	Excluded by TI/AB review

S4 Table. All studies identified in the literature search

1308	Cherry Picking Patients: Examining the Interval between Patient Rooming and Resident Self-assignment	Patterson, B. W.; Batt, R. J.; Wilbanks, M. D.; Otles, E.; Westergaard, M. C.; Shah, M. N.; Asher, S. L.	2016	Academic Emergency Medicine	23	6	679-684	10.1111/acem.12895	Patterson 2016	Irrelevant	Excluded by TI/AB review
1309	Burden of hyperkalemia and treatment patterns in the emergency department setting: Results from the REVEAL-ED study	Peacock, W. F.; Miller, J.; Char, D.; Clark, C. L.; Singer, A. J.; Rafique, Z.; Rasmussen, H.; Turner, S.	2016	Annals of Emergency Medicine	68	4	S1	NA	Peacock 2016	Irrelevant	Excluded by TI/AB review
1310	Relationship between dietary energy density and dietary quality in overweight young children: A cross-sectional analysis	Poole, S. A.; Hart, C. N.; Jelalian, E.; Raynor, H. A.	2016	Pediatric Obesity	11	2	128-135	10.1111/ijpo.12034	Poole 2016	Irrelevant	Excluded by TI/AB review

S4 Table. All studies identified in the literature search

1313	Racial disparities in the pain management of children in the post anesthesia care unit	Porter, L.; Nafiu, O.	2016	Anesthesia and Analgesia	122	5	S262	10.1213/01.ane.0000499505.96779.a0	Porter 2016	Irrelevant	Excluded by TI/AB review
1314	A multidisciplinary approach to impact acute care utilization among [illegible]	Powell, R. E.; Axelrod, D.; Pulte, E. D.; Lovett, P.; Crawford, A. G.; McAna, J.; Ward, L.	2016	Journal of General Internal Medicine	31	2	S99-S100	NA	Powell 2016	Irrelevant	Excluded by TI/AB review
1315	Comparison of Emergency Department Wait Times in Adults with Sickle Cell Disease Versus Other Painful Etiologies	Pulte, D.; Lovett, P. B.; Axelrod, D.; Crawford, A.; McAna, J.; Powell, R.	2016	Hemoglobin	40	5	330-334	10.1080/03630269.2016.1232272	Pulte 2016	Irrelevant	Excluded by TI/AB review

S4 Table. All studies identified in the literature search

1317	The role of bias by emergency department providers in care for American indian children	Puumala, S. E.; Burgess, K. M.; Kharbanda, A. B.; Zook, H. G.; Castille, D. M.; Pickner, W. J.; Payne, N. R.	2016	Medical Care	54	6	562-569	10.1097/MLR.0000000000000000533	Puumala 2016	Irrelevant	Excluded by TI/AB review
1318	Relationship between racial disparities in ED wait times and illness severity	Qiao, W. P.; Powell, E. S.; Witte, M. P.; Zelder, M. R.	2016	American Journal of Emergency Medicine	34	1	15-Oct	10.1016/j.ajem.2015.08.052	Qiao 2016	Irrelevant	Excluded by TI/AB review
1321	Assessment of Pediatric Neurotrauma Imaging Appropriateness at a Level I Pediatric Trauma Center	Rao, S.; Rao, S.; Rincon, S.; Caruso, P.; Ptak, T.; Raja, A. S.; Prabhakar, A. M.; Harvey, H. B.	2016	Journal of the American College of Radiology	13	7	788-793	10.1016/j.jacr.2016.02.022	Rao 2016	Irrelevant	Excluded by TI/AB review

S4 Table. All studies identified in the literature search

1322	The Patient-Centered Medical Home: How Is It Related to Quality and Equity Among the General Adult Population?	Reibling, N.	2016	Medical Care Research and Review	73	5	606-623	10.1177/1077558715622913	Reibling 2016	Irrelevant	Excluded by TI/AB review
1323	Interprofessional Collaboration to Improve Discharge from Skilled Nursing Facility to Home: Preliminary Data on Postdischarge [illegible]	Reidt, Shannon L.; Holtan, Haley S.; Larson, Tom A.; Thompson, Bruce; Kerzner, Lawrence J.; Salvatore, Toni M.; Adam, Terrence J.	2016	Journal of the American Geriatrics Society	64	9	1895-1899	10.1111/jgs.14258	Reidt 2016	Irrelevant	Excluded by TI/AB review
1324	Evaluation of a Worksite Diabetes Education Program at a Large Urban Medical Center	Renda, S.; Baernholdt, M.; Becker, K.	2016	Workplace health & safety	64	1	17-23	10.1177/2165079915607869	Renda 2016	Irrelevant	Excluded by TI/AB review

S4 Table. All studies identified in the literature search

1325	Treatment discontinuation of oral hypoglycemic agents and healthcare utilization among patients with [illegible]	Reynolds, K.; An, J.; Wu, J.; Harrison, T. N.; Wei, R.; Stuart, B.; Martin, J. P.; Wlodarczyk, C. S.; Rajpathak, S. N.	2016	Journal of Diabetes and its Complications	30	8	1443-1451	10.1016/j.jdiacomp.2016.07.021	Reynolds 2016	Irrelevant	Excluded by TI/AB review
1326	Attachment-focused integrative reminiscence with older African Americans: a randomized controlled intervention study	Sabir, M.; Henderson, C. R.; Kang, S. Y.; Pillemer, K.	2016	Aging & mental health	20	5	517-528	10.1080/13607863.2015.1023764	Sabir 2016	Irrelevant	Excluded by TI/AB review
1328	Impact of a home-based primary care initiative on emergency department and hospital utilization in multimorbid disabled patients	Schamess, A.; Foraker, R.; Kretovics, M.; Tayal, N. H.	2016	Journal of General Internal Medicine	31	2	S881	NA	Schamess 2016	Irrelevant	Excluded by TI/AB review

S4 Table. All studies identified in the literature search

1329	The role of integrated care in reducing hospitalizations and health care spending	Schmidt, Eric M.	2016	NA	77	NA	NA	NA	Schmidt 2016	Irrelevant	Excluded by TI/AB review
1330	Population Health Considerations for Pediatric Asthma: Findings from the	Shaikh, U.; Byrd, R. S.	2016	Popul Health Manag	19	2	145-51	10.1089/pop.2015.0015	Shaikh 2016	Irrelevant	Excluded by TI/AB review
1331	Assessing patient activation and health literacy in the ED	Sheikh, S.; Hendry, P.; Kalynych, C.; Owensby, B.; Johnson, J.; Kraemer, D. F.; Carden, D.	2016	American Journal of Emergency Medicine	34	1	93-96	10.1016/j.ajem.2015.09.045	Sheikh 2016	Irrelevant	Excluded by TI/AB review
1332	Do patients hospitalised in high-minority hospitals experience more diversion and poorer outcomes? A retrospective multivariate analysis of Medicare patients in California	Shen, Y. C.; Hsia, R. Y.	2016	BMJ Open	6	3	e010263	10.1136/bmjopen-2015-010263	Shen 2016	Irrelevant	Excluded by TI/AB review

S4 Table. All studies identified in the literature search

1333	Two-site bouncebacks: A multilevel analysis of patients returning to the same or different hospital within 72-hours of initial emergency department presentation	Shy, B. D.; Kim, E. Y.; Genes, N.; Lowry, T.; Loo, G. T.; Hwang, U.; Richardson, L. D.; Shapiro, J. S.	2016	Annals of Emergency Medicine	68	4	S1-S2	NA	Shy 2016	Irrelevant	Excluded by TI/AB review
1334	Turkish health care providers' views on inappropriate use of emergency department: Who, when and why?	≈ûim≈üek, Perihan; G√ºrsoy, Ayla	2016	International emergency nursing	27	NA	31-36	10.1016/j.ienj.2015.11.004	≈ûim≈üek 2016	Irrelevant	Excluded by TI/AB review
1335	Parental perceptions of avoidability of their child's emergency	Singhal, A.; Caplan, D. J.; Jones, M. P.; Kuthy, R. A.; Momany, E. T.; Buresh, C. T.;	2016	Emergency Medicine Journal	33	5	313-318	10.1136/emermed-2015-204790	Singhal 2016	Irrelevant	Excluded by TI/AB review
1336	Racial-Ethnic Disparities in Opioid Prescriptions at Emergency	Singhal, A.; Tien, Y. Y.; Hsia, R. Y.	2016	PLoS One	11	8	e0159224	10.1371/journal.pone.0159224	Singhal 2016	Irrelevant	Excluded by TI/AB review

S4 Table. All studies identified in the literature search

1337	Health related quality of life and return to work after minor extremity injuries: A longitudinal study comparing upper versus lower extremity injuries	Sluys, K. P.; Shults, J.; Richmond, T. S.	2016	Injury	47	4	824-831	10.1016/j.injury.2016.02.019	Sluys 2016	Irrelevant	Excluded by TI/AB review
1338	The ICU-CARES Initiative: ICU Collection, Analysis, and Response to Evaluations of Satisfaction	Smith, Orla; Butorac, Elizabeth; Guiyab, Melissa; Lewis, Ellen; Marks, Nikki; Mustard, Mary; Wannamaker, Karen; Poon, Lisa; Friedrich, Jan; Baker, Andrew	2016	Canadian Journal of Critical Care Nursing	27	2	46-46	NA	Smith 2016	Irrelevant	Excluded by TI/AB review

S4 Table. All studies identified in the literature search

1339	Racial-Ethnic Differences in Health Service Use in a Large Sample of Homeless Adults With Mental Illness From Five Canadian Cities	Stergiopoulos, Vicky; Gozdzik, Agnes; Nisenbaum, Rosane; Vasiliadis, Helen-Maria; Chambers, Catharine; McKenzie, Kwame; Misir, Vachan	2016	Psychiatric Services	67	9	1004-1011	10.1176/appi.ps.201500287	Stergiopoulos 2016	Irrelevant	Excluded by TI/AB review
1345	Brief case management versus usual care for frequent users of emergency departments: the [illegible]	Stergiopoulos, V.; Gozdzik, A.; Tan de Bibiana, J.; Guimond, T.; Hwang, S. W.; Wasylenki, D. A.; [illegible]	2016	BMC Health Services Research	16	1	432	10.1186/s12913-016-1666-1	Stergiopoulos 2016	Irrelevant	Excluded by TI/AB review
1346	Satisfaction Data Collected by E-mail and Smartphone for Emergency Department Patients: How Do Responders Compare With Nonresponders?	Strickler, J. C.; Lopiano, K. K.	2016	The Journal of nursing administration	46	11	592-598	NA	Strickler 2016	Irrelevant	Excluded by TI/AB review

S4 Table. All studies identified in the literature search

1347	Satisfaction Data Collected by E-mail and Smartphone for Emergency Department Patients	Strickler, Jeffery C.; Lopiano, Kenneth K.	2016	JONA: The Journal of Nursing Administration	46	11	592-598	10.1097/NNA.0000000000000410	Strickler 2016	Irrelevant	Excluded by TI/AB review
1348	Neurocognitive assessment of Hispanic individuals residing in the [illegible]	Strutt, Adriana M.; Burton, Veronica J.; Resendiz, Claudia V.; Peery, Shelley; Ferraro, F. Richard	2016	Minority and cross-cultural aspects of neuropsych [illegible]	NA	NA	201-228	NA	Strutt 2016	Irrelevant	Excluded by TI/AB review
1349	Dying well: How equal is end of life care among gynecologic oncology patients?	Taylor, J. S.; Brown, A. J.; Prescott, L. S.; Sun, C. C.; Ramondetta, L. M.; Bodurka, D. C.	2016	Gynecologic Oncology	140	2	295-300	10.1016/j.ygyno.2015.12.012	Taylor 2016	Irrelevant	Excluded by TI/AB review
1350	Rates and risk factors for unplanned emergency department utilization and hospital readmission [illegible]	Telem, D. A.; Yang, J.; Altieri, M.; Patterson, W.; Peoples, B.; Chen, H.; Talamini, M.; Pryor, A. D.	2016	Annals of Surgery	263	5	956-960	10.1097/SLA.0000000000001536	Telem 2016	Irrelevant	Excluded by TI/AB review

S4 Table. All studies identified in the literature search

1351	Patient understanding of ‚Äúflare‚Äù and ‚Äúremission‚Äù: Lagging behind changing IBD management paradigms	Trivedi, I.; Reddy, S.; Rosh, J.; Keefer, L.	2016	Inflammatory Bowel Diseases	22	NA	S25	10.1097/01.MIB.0000480152.67789.53	Trivedi 2016	Irrelevant	Excluded by TI/AB review
1352	Presenting characteristics and processing times for culturally and linguistically diverse (CALD) [illegible]	Wechkunanukul, K.; Grantham, H.; Clark, R.	2016	Heart Lung and Circulation	25	NA	S5	10.1016/j.hlc.2016.06.010	Wechkunanukul 2016	Irrelevant	Excluded by TI/AB review
1353	The association between ethnicity and delay in seeking medical care for chest pain: A systematic review	Wechkunanukul, K.; Grantham, H.; Damarell, R.; Clark, R. A.	2016	JBI Database of Systematic Reviews and Implementation Reports	14	7	208-235	10.11124/JBISRIR-2016-003012	Wechkunanukul 2016	Irrelevant	Excluded by TI/AB review
1355	Adapting Conceptual Frameworks for Patient Engagement in Emergency Department Research	Wright, R.; Carden, D. L.; Hess, E. P.; Booker-Vaughns, J.; Dunn, P.; Kizzie-Gillett, C.; Vaughan, W.; Weingarten, G.; Williams, P.; Young-Brinn, A.; Grudzen, C.	2016	Academic Emergency Medicine	23	12	1332-1336	10.1111/acem.13066	Wright 2016	Irrelevant	Excluded by TI/AB review

S4 Table. All studies identified in the literature search

1356	Emergency physician utilization of ultrasound in Arizona	Wyman, M. T.; Amini, R.; Hernandez, N. C.; Adhikari, S.	2016	Journal of Investigative Medicine	64	1	146	10.1136/jim-d-15-00013.27	Wyman 2016	Irrelevant	Excluded by TI/AB review
1357	An Investigation of Service Quality,ÄîWillingness to Recommend Relationship [illegible]	Yavas, Ugur; Babakus, Emin; Westbrook, Kevin W.; Grant, Cori Cohen; Deitz, George D.; Rafalski, Ed	2016	Journal of Health Management	18	1	49-69	10.1177/0972063415625508	Yavas 2016	Irrelevant	Excluded by TI/AB review
1358	Family presence during management of acute deterioration: Clinician attitudes, beliefs and perceptions of current practices	Youngson, M. J.; Currey, J.; Considine, J.	2016	Australasian emergency nursing journal : AENJ	19	3	159-165	10.1016/j.aenj.2016.05.001	Youngson 2016	Irrelevant	Excluded by TI/AB review
1359	A meta-regression evaluating the effectiveness and prognostic factors of oral phosphodiesterase [illegible]	Yuan, J. Q.; Mao, C.; Yang, Z. Y.; Fu, X. H.; Wong, S. Y.; Tang, J. L.	2016	Asian Journal of Andrology	18	1	60-65	10.4103/1008-682X.154304	Yuan 2016	Irrelevant	Excluded by TI/AB review

S4 Table. All studies identified in the literature search

1360	Assuring quality psychosocial care for all patients: An analysis of institutional capacity to deliver services to minority patients	Zebrack, B.; Kayser, K.	2016	Psycho-Oncology	25	NA	33	10.1002/pon.4082	Zebrack 2016	Irrelevant	Excluded by TI/AB review
1361	Electrocardiographic indicators of acute coronary syndrome are more common in patients with ambulance transport [illegible]	Z√®gre-Hemsey, J. K.; Pickham, D.; Pelter, M. M.	2016	J Electrocardiol	49	6	944-950	10.1016/j.jelectrocard.2016.08.008	Z√®gre-Hemsey 2016	Irrelevant	Excluded by TI/AB review
1363	Ed physician adherence to IDSA treatment guidelines for skin and soft tissue infections: Risks of deviating from standard therapy	Zeoli, T.; Wilsterman, E.; Patrick Haran, J.	2016	Academic Emergency Medicine	23	NA	S192	10.1111/acem.12974	Zeoli 2016	Irrelevant	Excluded by TI/AB review

S4 Table. All studies identified in the literature search

1364	Males with inflammatory bowel disease have a high prevalence of hypogonadism and erectile dysfunction	Zibert, K.; Akhuemonkhan, E.; Hutfless, S.; Solveson, K.; Betteridge, J. D.; Singla, M. B.	2016	American Journal of Gastroenterology	111	NA	S301	10.1038/ajg.2016.358	Zibert 2016	Irrelevant	Excluded by TI/AB review
1365	Social work with lesbian, gay, bisexual, and transgender older adults	Zodikoff, Bradley D.; Butler, Sandra S.; Kaplan, Daniel B.; Berkman, Barbara	2016	The Oxford handbook of social work in health and aging., 2nd ed.	NA	NA	555-563	NA	Zodikoff 2016	Irrelevant	Excluded by TI/AB review
1366	Differential access to care: The role of age, insurance, and income on race/ethnicity-related disparities in adult perforated appendix admission rates	Zogg, C. K.; Scott, J. W.; Jiang, W.; Wolf, L. L.; Haider, A. H.	2016	Surgery	160	5	1145-1154	10.1016/j.surg.2016.06.002	Zogg 2016	Irrelevant	Excluded by TI/AB review

S4 Table. All studies identified in the literature search

1367	Adherence to combination antiretroviral treatment and clinical outcomes in a Medicaid sample of older HIV-infected [illegible]	Abara, W. E.; Adekeye, O. A.; Xu, J.; Rust, G.	2017	AIDS Care - Psychological and Socio-Medical Aspects of AIDS/HIV	29	4	441-448	10.1080/09540121.2016.1257774	Abara 2017	Irrelevant	Excluded by TI/AB review
1368	Association between survival time with metastatic breast cancer and aggressive end-of-life care	Accordino, M. K.; Wright, J. D.; Vasan, S.; Neugut, A. I.; Gross, T.; Hillyer, G. C.; Hershman, D. L.	2017	Breast Cancer Research and Treatment	166	2	549-558	10.1007/s10549-017-4420-4	Accordino 2017	Irrelevant	Excluded by TI/AB review
1369	The unmet supportive care needs of cancer survivors	Alananzeh, I.; Levsque, J.; Kwok, C.; Everett, B.	2017	Supportive Care in Cancer	25	2	S208	10.1007/s00520-017-3704-x	Alananzeh 2017	Irrelevant	Excluded by TI/AB review

S4 Table. All studies identified in the literature search

1370	Access to bariatric surgery: A statewide report on wait times for the surgical treatment of morbid obesity	Alvarez, R.; Cassidy, R. B.; Bonham, A. J.; Buda, C. M.; Varban, O. A.	2017	Journal of the American College of Surgeons	225	4	e104	NA	Alvarez 2017	Irrelevant	Excluded by TI/AB review
1371	Eliminating Disparities in Asthma Care: Identifying Broad Challenges in Quality [illegible]	Anarella, J. P.; Wagner, V. L.; McCauley, S. G.; Mane, J. B.; Waniewski, P. A.	2017	American journal of medical quality : the official journal of [illegible]	32	6	598-604	10.1177/1062860616682587	Anarella 2017	Irrelevant	Excluded by TI/AB review
1374	A patient advocate to facilitate access and improve communication, care, and outcomes [illegible]	Apter, A. J.; Morales, K. H.; Han, X.; Perez, L.; Huang, J.; Ndicu, G.; Localio, A.; Nardi, [illegible]	2017	Contemporary Clinical Trials	56	NA	34-45	10.1016/j.cct.2017.03.004	Apter 2017	Irrelevant	Excluded by TI/AB review
1375	The willingness of adolescents to commit to safe use, storage, and disposal of prescription opiates in the emergency [illegible]	Arora, S.; Grewal, S.; Escamilla, D.; Menchine, M.	2017	Annals of Emergency Medicine	70	4	S92	NA	Arora 2017	Irrelevant	Excluded by TI/AB review

S4 Table. All studies identified in the literature search

1376	Where do children with asthma die? a national perspective from 2003 to 2014	Arroyo, A. J. C.; Chee, C. P.; Wang, N. E.	2017	Journal of Allergy and Clinical Immunology	139	2	AB89	NA	Arroyo 2017	Irrelevant	Excluded by TI/AB review
1377	Management of acute low back pain in the ED: A systematic review	Ashbrook, J.; Rodgdakis, N.; Goodwin, P.; Yeowell, G.; Callaghan, M.	2017	Emergency Medicine Journal	34	12	889	10.1136/emermed-2017-207308.43	Ashbrook 2017	Irrelevant	Excluded by TI/AB review
1378	Pain Perception in Latino vs. Caucasian and Male vs. Female Patients: Is There Really a Difference?	Aufiero, Molly; Stankewicz, Holly; Quazi, Shaila; Jacoby, Jeanne; Stoltzfus, Jill	2017	Western Journal of Emergency Medicine: Integrating Emergency Care with Population Health	18	4	737-742	10.5811/westjem.2017.1.32723	Aufiero 2017	Irrelevant	Excluded by TI/AB review

S4 Table. All studies identified in the literature search

1379	Adherence to hydroxyurea, health-related quality of life domains, and patients' perceptions of sickle cell disease and hydroxyurea: A cross-sectional study in adolescents and young adults	Badawy, S. M.; Thompson, A. A.; Lai, J. S.; Penedo, F. J.; Rychlik, K.; Liem, R. I.	2017	Health and Quality of Life Outcomes	15	1	NA	10.1186/s12955-017-0713-x	Badawy 2017	Irrelevant	Excluded by TI/AB review
1380	Associations between perceived discrimination and health status among frequent Emergency Department users	Baggio, S.; Iglesias, K.; Hugli, O.; Burnand, B.; Ruggeri, O.; Wasserfallen, J. B.; Moschetti, K.; Staeger, P.; Alary, S.; Canepa Allen, M.; Daeppen, J. B.; Bodenmann, P.	2017	Eur J Emerg Med	24	2	136-141	10.1097/mej.0000000000000311	Baggio 2017	Irrelevant	Excluded by TI/AB review

S4 Table. All studies identified in the literature search

1382	Incidence of diagnostic error among patients hospitalized for CVT at a single urban center	Bakradze, E.; Liberman, A. L.	2017	Diagnosis	4	4	eA121	10.1515/dx-2017-0034	Bakradze 2017	Irrelevant	Excluded by TI/AB review
1383	Individual and county level predictors of asthma related emergency department visits among children on [illegible]	Baltrus, P.; Xu, J.; Immergluck, L.; Gaglioti, A.; Adesokan, A.; Rust, G.	2017	J Asthma	54	1	53-61	10.1080/02770903.2016.1196367	Baltrus 2017	Irrelevant	Excluded by TI/AB review
1385	Individual and county level predictors of asthma related emergency department visits among children on [illegible]	Baltrus, P.; Xu, J.; Immergluck, L.; Gaglioti, A.; Adesokan, A.; Rust, G.	2017	Journal of Asthma	54	1	53-61	10.1080/02770903.2016.1196367	Baltrus 2017	Irrelevant	Excluded by TI/AB review

S4 Table. All studies identified in the literature search

1386	The effect of patient death on medical students in the emergency department	Batley, N. J.; Bakhti, R.; Chami, A.; Jabbour, E.; Bachir, R.; El Khuri, C.; Mufarrij, A. J.	2017	BMC medical education	17	1	110	10.1186/s12909-017-0945-9	Batley 2017	Irrelevant	Excluded by TI/AB review
1387	California births, 2009-2011: Describing high utilizers of postpartum emergency department care	Batra, P.; Fridman, M.; Leng, M.; Gregory, K. D.	2017	American Journal of Obstetrics and Gynecology	216	1	S420	NA	Batra 2017	Irrelevant	Excluded by TI/AB review
1388	Cultural awareness	Battle, Brenda; Todic, Jelena; Potter, Patricia A.; Perry, Anne Griffin; Stockert, Patricia A.; Hall, Amy M.; Ostendorf, Wendy R.	2017	Fundamentals of nursing., 9th ed.	NA	NA	101-116	NA	Battle 2017	Irrelevant	Excluded by TI/AB review

S4 Table. All studies identified in the literature search

1389	Characterization of Stress in Low-Income, Inner-City Mothers of Children with Poorly Controlled Asthma	Bellin, M. H.; Collins, K. S.; Osteen, P.; Kub, J.; Bollinger, M. E.; Newsome, A.; Lewis-Land, C.; Butz, A. M.	2017	Journal of urban health : bulletin of the New York Academy of Medicine	94	6	814-823	10.1007/s11524-017-0162-1	Bellin 2017	Irrelevant	Excluded by TI/AB review
1390	Caregiver perception of asthma management of children in the context of poverty	Bellin, M. H.; Land, C.; Newsome, A.; Kub, J.; Mudd, S. S.; Bollinger, M. E.; Butz, A. M.	2017	Journal of Asthma	54	2	162-172	10.1080/02770903.2016.1198375	Bellin 2017	Irrelevant	Excluded by TI/AB review
1391	Passive ultra-brief video training improves performance of compression-only cardiopulmonary [illegible]	Benoit, J. L.; Vogele, J.; Hart, K. W.; Lindsell, C. J.; McMullan, J. T.	2017	Resuscitation	115	NA	116-119	10.1016/j.resuscitation.2017.04.008	Benoit 2017	Irrelevant	Excluded by TI/AB review
1393	Upper gastrointestinal (GI) bleed as a manifestation of poorly differentiated metastatic [illegible]	Bhardwaj, R.; Bhardwaj, G.; Karagozian, R.	2017	Journal of General Internal Medicine	32	2	S630	NA	Bhardwaj 2017	Irrelevant	Excluded by TI/AB review

S4 Table. All studies identified in the literature search

1394	A systematic review of antimicrobial stewardship interventions in the emergency department	Black, E.; Ramsey, T.; Losier, M.; Wilby, K.	2017	Canadian Journal of Hospital Pharmacy	70	1	68	NA	Black 2017	Irrelevant	Excluded by TI/AB review
1395	Multiorgan system failure with hemoptysis complicating acute chest syndrome	Bruehl, M. J.; Vece, T. J.	2017	American Journal of Respiratory and Critical Care Medicine	195	NA	NA	10.1164/ajrccm-conference.2017.A83	Bruehl 2017	Irrelevant	Excluded by TI/AB review
1396	A Pediatric Application of the STRAC Regional Hospital Trauma Registry Database: [illegible]	Buehner, M.; Aden, J.; Borgman, M.; Love, P.; Wright, B.; Edwards, M.	2017	Texas medicine	113	1	e1	NA	Buehner 2017	Irrelevant	Excluded by TI/AB review
1397	Predicting admission at triage: Are nurses better than a simple objective score?	Cameron, A.; Ireland, A. J.; McKay, G. A.; Stark, A.; Lowe, D. J.	2017	Emergency Medicine Journal	34	1	7-Feb	10.1136/emermed-2014-204455	Cameron 2017	Irrelevant	Excluded by TI/AB review

S4 Table. All studies identified in the literature search

1398	Employing a geospatial analysis of asthma-related emergency department visits to target community-based	Carlson, L. C.; Repucci, R.; Dworkis, D. A.; Peak, D. A.	2017	Annals of Emergency Medicine	70	4	S156-S157	NA	Carlson 2017	Irrelevant	Excluded by TI/AB review
1399	Early palliative care for patients with solid tumors and hematological malignancies: Impacton quality metrics and costs of care	Cassel, B.; McClish, D.; Albrecht, T.; Skoro, N.; Noreika, D.; Del Fabbro, E.	2017	Supportive Care in Cancer	25	2	S185	10.1007/s00520-017-3704-x	Cassel 2017	Irrelevant	Excluded by TI/AB review

S4 Table. All studies identified in the literature search

1400	Determinants of Pediatric Psychiatry Length of Stay in 2 Urban Emergency Departments	Chakravarthy, B.; Yang, A.; Ogbu, U.; Kim, C.; Iqbal, A.; Haight, J.; Anderson, C.; Dimassa, G.; Bruckner, T.; Bhargava, R.; Schreiber, M.; Lotfipour, S.	2017	Pediatric Emergency Care	33	9	613-619	10.1097/PEC.0000000000000509	Chakravarthy 2017	Irrelevant	Excluded by TI/AB review
1401	Electronic adherence monitoring device performance and patient acceptability: a randomized control trial	Chan, A. H. Y.; Stewart, A. W.; Harrison, J.; Black, P. N.; Mitchell, E. A.; Foster, J. M.	2017	Expert Review of Medical Devices	14	5	401-411	10.1080/17434440.2017.1322505	Chan 2017	Irrelevant	Excluded by TI/AB review

S4 Table. All studies identified in the literature search

1402	Jefferson ED-observation unit telehealth transition of care for heart failure (JETT-HFA): A mixed methods study	Chang, A. M.; Sanjeevan, I.; Jennings, E.; Watts, P.; Whellan, D. J.; Hollander, J. E.	2017	Journal of Cardiac Failure	23	8	S80-S81	NA	Chang 2017	Irrelevant	Excluded by TI/AB review
1403	Jefferson emergency department/observation unit telehealth transition of care for heart failure (JETT-HF): A [illegible]	Chang, A. M. M.; Sanjeevan, I.; Jennings, E.; Watts, P.; Whellan, D. J.; Hollander, J. E.	2017	Annals of Emergency Medicine	70	4	S43-S44	NA	Chang 2017	Irrelevant	Excluded by TI/AB review
1404	Caring for Young Children with Asthma: Perspectives from Urban Community Health Centers	Childs, E.; Laws, M. A.; Drainoni, M. L.; Nath, A.; Reid, M. J.; Sandel, M.; Cohen, R. T.	2017	Journal of Urban Health-Bulletin of the New York Academy of Medicine	94	6	824-834	10.1007/s11524-017-0186-6	Childs 2017	Irrelevant	Excluded by TI/AB review

S4 Table. All studies identified in the literature search

1405	Transgender and Gender Nonconforming in Emergency Departments: A	Chisolm-Straker, M.; Jardine, L.; Bennouna, C.; Morency-Brassard, N.; Coy, L.; Egemba,	2017	Transgend Health	2	1	16-Aug	10.1089/trgh.2016.0026	Chisolm-Straker 2017	Irrelevant	Excluded by TI/AB review
1408	Chronic obstructive pulmonary disease patients transition of care-factors associated with	Corpus, D.; Gastanaduy, M.; Price-Haywood, E.	2017	Ochsner Journal	17	3	e36	NA	Corpus 2017	Irrelevant	Excluded by TI/AB review
1409	Using peer navigators to address the integrated health care needs of homeless African Americans with serious mental illness	Corrigan, Patrick W.; Kraus, Dana J.; Pickett, Susan A.; Schmidt, Annie; Stellon, Ed; Hantke, Erin; Lara, Juana Lorena	2017	Psychiatric Services	68	3	264-270	10.1176/appi.ps.201600134	Corrigan 2017	Irrelevant	Excluded by TI/AB review
1410	Your finger or mine? Patient preferences on stool collection in the emergency department	Couperus, K.	2017	Annals of Emergency Medicine	70	4	S23	NA	Couperus 2017	Irrelevant	Excluded by TI/AB review

S4 Table. All studies identified in the literature search

1411	More to gain: Diet energy density is associated with smoking status in US adults	Cowan, A.; MaClean, R. R.; Vernarelli, J. A.	2017	FASEB Journal	31	1	NA	NA	Cowan 2017	Irrelevant	Excluded by TI/AB review
1412	The relationship between medication knowledge, perceived importance, and medication adherence	Coyne, C. J.; Ence, T.; Smyres, C.; Brennan, J.; Castillo, E.; Vilke, G. M.	2017	Annals of Emergency Medicine	70	4	S169	NA	Coyne 2017	Irrelevant	Excluded by TI/AB review

S4 Table. All studies identified in the literature search

1413	The role and interactions of 25-OHD, parathyroid hormone, and serum calcium in pediatric patients with fractures compared with healthy controls	Crabb, R.; Poon, S.; Olson, A.; Merwin, S.; Gecelter, R.; Wendolowski, S.; Philip, J.; Fishbein, J.; Avarello, J.	2017	Journal of Bone and Mineral Research	31	NA	NA	10.1002/jbmr.3107	Crabb 2017	Irrelevant	Excluded by TI/AB review
1414	Follow-up care after emergency department visits for mental and substance use disorders among medicaid beneficiaries	Croake, S.; Brown, J. D.; Miller, D.; Darter, N.; Patel, M. M.; Liu, J.; Scholle, S. H.	2017	Psychiatric Services	68	6	566-572	10.1176/appi.ps.201500529	Croake 2017	Irrelevant	Excluded by TI/AB review
1415	Frequent emergency department visitors are frequent primary care visitors and report unmet primary care needs	Cunningham, A.; Mautner, D.; Ku, B.; Scott, K.; LaNoue, M.	2017	Journal of Evaluation in Clinical Practice	23	3	567-573	10.1111/jep.12672	Cunningham 2017	Irrelevant	Excluded by TI/AB review

S4 Table. All studies identified in the literature search

1416	Initial experience with co-management of pediatric patients with functional abdominal pain between pediatric gastroenterolgoy [illegible]	D'Amico, S.; Goh, V. L.; Morera, C.; Goldstein, L.; Gardiner, P.; Neri, C.	2017	Journal of Pediatric Gastroenterology and Nutrition	65	NA	S265	10.1097/MPG.0000000000001805	D'Amico 2017	Irrelevant	Excluded by TI/AB review
1417	Effect of pulmonary pressure monitoring (Cardiomems heart [illegible]	Davidovich, D.; Jonsson, O.; Pelzel, J.; Bennett, M.; Bart, B.; MacKedanz, S. A.; Sexter, A.; [illegible]	2017	Journal of the American College of Cardiology	69	11	764	10.1016/S0735-1097(17)34153-0	Davidovich 2017	Irrelevant	Excluded by TI/AB review
1418	Estimation of medication adherence by pill count compared to liquid chromatography mass spectrometry in emergency [illegible]	Dawood, R.; McNaughton, C.; Mango, L. M.; Gowland, L.; Brody, A.; Flack, J.; Levy, P.	2017	Academic Emergency Medicine	24	NA	S235	10.1111/acem.13203	Dawood 2017	Irrelevant	Excluded by TI/AB review
1419	Medical Treatment for Spontaneous Anticoagulation-Related Intracerebral Hemorrhage in the Netherlands	de Schipper, L. J.; Baharoglu, M. I.; Roos, Y. B. W. E. M.; de Beer, F.	2017	Journal of Stroke and Cerebrovascular Diseases	26	7	1427-1432	10.1016/j.jstrokecerebrovasdis.2017.03.019	deSchipper 2017	Irrelevant	Excluded by TI/AB review

S4 Table. All studies identified in the literature search

1424	Acceptance of guidance to care at the emergency department following [illegible]	Dekker, W. P. H.; Vergouwen, A. C. M.; Buster, M. C. A.; Honig, A.	2017	BMC Psychiatry	17	1	NA	10.1186/s12888-017-1491-z	Dekker 2017	Irrelevant	Excluded by TI/AB review
1425	Prehospital Intubation is Associated with Favorable Outcomes and Lower Mortality in ProTECT III	Denninghoff, K. R.; Nu√±o, T.; Pauls, Q.; Yeatts, S. D.; Silbergleit, R.; Palesch, Y. Y.; Merck, L. H.; Manley, G. T.; Wright, D. W.	2017	Prehospital emergency care : official journal of the National Association of EMS Physicians [illegible]	21	5	539-544	10.1080/10903127.2017.1315201	Denninghoff 2017	Irrelevant	Excluded by TI/AB review
1426	The influence of psychiatric health on breast abscesses in the non-lactating patient: A community hospital's experience	DeStefano, L.; Fults, M.; Rao, S.; Matthews, J.; Malhan, S.; Schwartz, K.; Kish, K.	2017	Annals of Surgical Oncology	24	2	63-64	10.1245/s10434-017-5854-y	DeStefano 2017	Irrelevant	Excluded by TI/AB review

S4 Table. All studies identified in the literature search

1427	Observational study of alternative therapies among paediatric emergency department patients	Ding, J. L.; Taylor, D. M.; Lee, M.; Johnson, O. G.; Ashok, A.; Griffiths, M.; Simma, L.; Craig, S. S.; Cheek, J. A.; Babl, F. E.	2017	EMA - Emergency Medicine Australasia	29	2	136-142	10.1111/1742-6723.12744	Ding 2017	Irrelevant	Excluded by TI/AB review
1428	Beliefs about a complementary and alternative therapy-based chronic pain management program for a medicaid population	Donovan, E.; Ranney, M. L.; Patry, E. J.; McKenzie, M.; Baird, J.; Green, T. C.	2017	Pain Medicine (United States)	18	9	1805-1816	10.1093/pm/pnx051	Donovan 2017	Irrelevant	Excluded by TI/AB review
1429	Changes in health-related quality of life for geriatric patients after an emergency department visit	Dresden, S. M.; Lindquist, L. A.; Courtney, D. M.	2017	Annals of Emergency Medicine	70	4	S84-S85	NA	Dresden 2017	Irrelevant	Excluded by TI/AB review

S4 Table. All studies identified in the literature search

1430	Mean HEART scores for hospitalized chest pain patients are higher in more experienced [illegible]	Dubin, J.; Kiechle, E.; Wilson, M.; Timbol, C.; Bhat, R.; Milzman, D.	2017	American Journal of Emergency Medicine	35	1	122-125	10.1016/j.ajem.2016.10.037	Dubin 2017	Irrelevant	Excluded by TI/AB review
1431	30-day ED visit and readmission rate after a hospital at home admission vs traditional hospitalization	Escobar, C.; Soones, T. N.; Leff, B.; Marshall, J.; Silversmith, G.; Siu, A. L.; Wajnberg, A.; DeCherrie, L.	2017	Journal of General Internal Medicine	32	2	S726	NA	Escobar 2017	Irrelevant	Excluded by TI/AB review
1432	Disparities in quality of primary care by residents and staff physicians: Is there a conflict between training needs and equity [illegible]	Essien, U. R.; Abraham, J. R.; Ray, A.; He, W.; Chang, Y.; Singer, D. E.; Atlas, S. J.	2017	Journal of General Internal Medicine	32	2	S164	NA	Essien 2017	Irrelevant	Excluded by TI/AB review
1433	Black women,Äôs mental health: Balancing strength and vulnerability	Evans, Stephanie Y.; Bell, Kanika; Burton, Nsenga K.	2017	NA	NA	NA	NA	NA	Evans 2017	Irrelevant	Excluded by TI/AB review

S4 Table. All studies identified in the literature search

1434	Factors predicting self-reported medication low adherence in a large sample of adults in the US general population: [illegible]	Feehan, M.; Morrison, M. A.; Tak, C.; Morisky, D. E.; Deangelis, M. M.; Munger, M. A.	2017	BMJ Open	7	6	NA	10.1136/bmjopen-2016-014435	Feehan 2017	Irrelevant	Excluded by TI/AB review
1435	Emergency Department waiting times in a tertiary children's hospital in Israel: A retrospective cohort study	Feldman, O.; Allon, R.; Leiba, R.; Shavit, I.	2017	Israel Journal of Health Policy Research	6	1	NA	10.1186/s13584-017-0184-x	Feldman 2017	Irrelevant	Excluded by TI/AB review
1436	Design and Rationale of a Randomized Trial of a Care Transition Strategy in Patients with Acute Heart Failure Discharged from the [illegible]	Fermann, G. J.; Levy, P. D.; Pang, P.; Butler, J.; Ayaz, S. I.; Char, D.; Dunn, P.; Jenkins, C. A.; Kampe, C.; Khan, Y.; Kumar, V. A.; Lindenfeld, J.; Liu, D.; Miller, K.; [illegible]	2017	Circulation: Heart Failure	10	2	NA	10.1161/CIRCHEARTFAILURE.116.003581	Fermann 2017	Irrelevant	Excluded by TI/AB review

S4 Table. All studies identified in the literature search

1437	Prehospital intubation is associated with favorable outcomes in protect III	Figueroa, J.; Nu√±o, T.; Denninghoff, K. R.; Huang, C.; Pauls, Q.; Yeatts, S. D.; Silbergleit, R.; Palesch, Y. Y.; Merck, L. H.; Manley, G.; Calcaterra, R. W.	2017	Academic Emergency Medicine	24	NA	S196	10.1111/acem.13203	Figueroa 2017	Irrelevant	Excluded by TI/AB review
1438	Treatment-associated toxicities reported by patients with early-stage	Friese, C. R.; Harrison, J. M.; Janz, N. K.; Jagsi, R.; Morrow, M.; Li, Y.; Hamilton, A. S.;	2017	Cancer	123	11	1925-1934	10.1002/cncr.30547	Friese 2017	Irrelevant	Excluded by TI/AB review
1440	Assessing the feasibility, acceptability, and potential effectiveness of a behavioral-	Fritz, H.; Brody, A.; Levy, P.	2017	Contemporary Clinical Trials Communications	7	NA	166-171	10.1016/j.conctc.2017.06.009	Fritz 2017	Irrelevant	Excluded by TI/AB review
1441	Feasibility and Acceptability of a Colocated Homeless-Tailored Primary Care Clinic and	Gabrielian, S.; Chen, J. C.; Minhaj, B. P.; Manchanda, R.; Altman, L.; Koosis, E.; Gelberg, L.	2017	Journal of primary care & community health	8	4	338-344	10.1177/2150131917699751	Gabrielian 2017	Irrelevant	Excluded by TI/AB review

S4 Table. All studies identified in the literature search

1442	Evaluation and improvement of the UCLA ronald reagan emergency department pain management practices for long bone fracture	Gandhi, K.; Sachs, C.	2017	Academic Emergency Medicine	24	NA	S205	10.1111/acem.13203	Gandhi 2017	Irrelevant	Excluded by TI/AB review
1443	Developing an algorithm to approach patients who refuse care but lack capacity	George, M.; Wack, K.; Surapaneni, S.; Larson, S.	2017	Journal of General Internal Medicine	32	2	S484	NA	George 2017	Irrelevant	Excluded by TI/AB review

S4 Table. All studies identified in the literature search

1444	Parental management of discharge instructions: A systematic review	Glick, A. F.; Farkas, J. S.; Nicholson, J.; Dreyer, B. P.; Fears, M.; Bandera, C.; Stolper, T.; Gerber, N.; Shonna Yin, H.	2017	Pediatrics	140	2	NA	10.1542/peds.2016-4165	Glick 2017	Irrelevant	Excluded by TI/AB review
1445	Does stigmatizing language in patient charts negatively impact physician attitudes and clinical decision making? an experimental study	Goddu, A. P.; O'Conor, K.; Lanzkron, S.; Saheed, M.; Saha, S.; Peek, M. E.; Haywood, C.; Beach, M. C.	2017	Journal of General Internal Medicine	32	2	S169-S170	NA	Goddu 2017	Irrelevant	Excluded by TI/AB review

S4 Table. All studies identified in the literature search

1446	Intensive procedure preferences at the end of life (EOL) in older Latino adults with end stage renal disease (ESRD) on dialysis	Gonzalez, K.; Ulloa, J. G.; Moreno, G.; Echeverr√≠a, O.; Norris, K.; Talamantes, E.	2017	BMC Nephrology	18	1	NA	10.1186/s12882-017-0739-7	Gonzalez 2017	Irrelevant	Excluded by TI/AB review
1448	'They treated me like crap and I know it was because I was Native': The healthcare experiences of Aboriginal peoples living in Vancouver's inner city	Goodman, Ashley; Fleming, Kim; Markwick, Nicole; Morrison, Tracey; Lagimodiere, Louise; Kerr, Thomas	2017	Social Science & Medicine	178	NA	87-94	10.1016/j.socscimed.2017.01.053	Goodman 2017	Irrelevant	Excluded by TI/AB review

S4 Table. All studies identified in the literature search

1449	'They treated me like crap and I know it was because I was Native': The healthcare experiences of aboriginal peoples [illegible]	Goodman, Ashley; Fleming, Kim; Markwick, Nicole; Morrison, Tracey; Lagimodiere, Louise; Kerr, Thomas	2017	Social Science & Medicine	184	NA	187-187	10.1016/j.socscimed.2017.04.050	Goodman 2017	Irrelevant	Excluded by TI/AB review
1450	Racial and Ethnic Differences in Antibiotic Use for Viral Illness in Emergency Departments	Goyal, M. K.; Johnson, T. J.; Chamberlain, J. M.; Casper, T. C.; Simmons, T.; Alessandrini, E. A.; Bajaj, L.; Grundmeier, R. W.; Gerber, J. S.; Lorch, [illegible]	2017	Pediatrics	140	4	NA	10.1542/peds.2017-0203	Goyal 2017	Irrelevant	Excluded by TI/AB review
1454	Management of Chronic Wounds: Diagnosis, Preparation, Treatment, and Follow-up	Gupta, S.; Andersen, C.; Black, J.; de Leon, J.; Fife, C.; Lantis Ii, J. C.; Niezgoda, J.; Snyder, R.; Sumpio, B.; Tettelbach, W.; [illegible]	2017	Wounds : a compendium of clinical research and practice	29	9	S19-S36	NA	Gupta 2017	Irrelevant	Excluded by TI/AB review

S4 Table. All studies identified in the literature search

1455	Emergency department query for patient-centered approaches to sexual orientation and gender identity The EQUALITY [illegible]	Haider, A. H.; Schneider, E. B.; Kodadek, L. M.; Adler, R. R.; Ranjit, A.; Torain, M.; Shields, R. Y.; Snyder, C.; Schuur, J. D.; Vail, L.; [illegible]	2017	JAMA Internal Medicine	177	6	819-828	10.1001/jamainternmed.2017.0906	Haider 2017	Irrelevant	Excluded by TI/AB review
1456	What accounts for the variation in ‚Äúnon-emergency‚Äù emergency department use among medicare enrollees?	Hanchate, A. D.; Baker, W. E.; Paasche-Orlow, M.; Dyer, K. S.; Degeorge, J.; Feldman, J.	2017	Academic Emergency Medicine	24	NA	S20	10.1111/acem.13203	Hanchate 2017	Irrelevant	Excluded by TI/AB review
1457	What accounts for the variation in avoidable ed use among medicare enrollees?	Hanchate, A. D.; Baker, W. E.; Paasche-Orlow, M.; Dyer, S.; Degeorge, J.; Feldman, J.	2017	Journal of General Internal Medicine	32	2	S380	NA	Hanchate 2017	Irrelevant	Excluded by TI/AB review

S4 Table. All studies identified in the literature search

1458	Elderly patients are at increased risk for treatment failure in outpatient management of purulent skin infections	Haran, J. P.; Wilsterman, E.; Zeoli, T.; Beaudoin, F. L.; Tjia, J.; Hibberd, P. L.	2017	American Journal of Emergency Medicine	35	2	249-254	10.1016/j.ajem.2016.10.060	Haran 2017	Irrelevant	Excluded by TI/AB review
1459	Patient satisfaction of and barriers to routine opt-out HIV screening in the emergency department: Results from ‚Äúthe HIV tested [illegible]	Haukoos, J. S.; Bucossi, M.; Hopkins, E.; Finnegan, K.; Lyle, C.; Ruffner, A.; Signer, D.; Todorovic, T.; Viertel, V.	2017	Academic Emergency Medicine	24	NA	S98	10.1111/acem.13203	Haukoos 2017	Irrelevant	Excluded by TI/AB review
1460	Care for patients with high needs and high costs: The role of home visits	Hilgeman, B. C.; Mitchell, J. L.; MacKinney, T.	2017	Journal of General Internal Medicine	32	2	S739-S740	NA	Hilgeman 2017	Irrelevant	Excluded by TI/AB review

S4 Table. All studies identified in the literature search

1461	Factors associated with relapse in adult patients discharged from the emergency department following acute asthma: A systematic review	Hill, J.; Arrotta, N.; Villa-Roel, C.; Dennett, L.; Rowe, B. H.	2017	BMJ Open Respiratory Research	4	1	NA	10.1136/bmjresp-2016-000169	Hill 2017	Irrelevant	Excluded by TI/AB review
1462	The impact of mental health services in a pediatric emergency department the implications of having trained psychiatric [illegible]	Holder, S. M.; Rogers, K.; Peterson, E.; Shoenleben, R.; Blackhurst, D.	2017	Pediatric Emergency Care	33	5	311-314	10.1097/PEC.0000000000000836	Holder 2017	Irrelevant	Excluded by TI/AB review

S4 Table. All studies identified in the literature search

1463	Efficacy and Factors Associated with Treatment Response of Intravenous Immunoglobulin in Inpatients with Refractory Inflammatory Bowel Diseases	Horton, N.; Kochhar, G.; Patel, K.; Lopez, R.; Shen, B.	2017	Inflammatory Bowel Diseases	23	7	1080-1087	10.1097/MIB.0000000000001116	Horton 2017	Irrelevant	Excluded by TI/AB review
1464	Factors associated with patients who prefer HIV self-testing over health professional testing in an emergency department-based rapid HIV screening program	Hsieh, Y. H.; Beck, K. J.; Rothman, R. E.; Gauvey-Kern, M.; Woodfield, A.; Peterson, S.; Signer, D.; Gaydos, C. A.	2017	International Journal of STD and AIDS	28	11	1124-1129	10.1177/0956462416689629	Hsieh 2017	Irrelevant	Excluded by TI/AB review
1465	Influence of Healthcare Utilization and Social	Huang, J. C. L.	2017	Health and Health Care Concerns among	35	NA	211-230	NA	Huang 2017	Irrelevant	Excluded by TI/AB review

S4 Table. All studies identified in the literature search

1466	Pediatric Asthma Health Disparities: Race, Hardship, Housing, and Asthma in a National Survey	Hughes, H. K.; Matsui, E. C.; Tschudy, M. M.; Pollack, C. E.; Keet, C. A.	2017	Acad Pediatr	17	2	127-134	10.1016/j.acap.2016.11.011	Hughes 2017	Irrelevant	Excluded by TI/AB review
1468	The prevalence of racial and socioeconomic disparities in an outpatient transitional care clinic	Hussain, K.; McMahon, C.; Krallman, R.; Montgomery, D.; Feldeisen, T.; Morgan, S.; Koelling, T.; Rubenfire, M.; Kline-Rogers, E.; Eagle, K. A.; Bumpus, S. M.	2017	Circulation: Cardiovascular Quality and Outcomes	10	NA	NA	NA	Hussain 2017	Irrelevant	Excluded by TI/AB review
1469	Rapid progression of chronic hepatitis C virus infection following treatment failure with harvoni: A case report	Idowu, K.; Odigwe, R.; Ogundipe, T.; Aurangzeb, A.; Mubarak, H.; Adebogun, A.; Filani, O.; Sran, G.	2017	American Journal of Gastroenterology	112	NA	S1274	10.1038/ajg.2017.321	Idowu 2017	Irrelevant	Excluded by TI/AB review

S4 Table. All studies identified in the literature search

1470	Unmet Primary Care Needs in Diabetic Patients with Multimorbidity in a Medically Underserved Area	Jackson, B. M.; Gutierrez, M. L.; Relyea, G. E.; Carlton, E. L.; Ahn, S.; Binkley, B. L.; Bailey, J. E.	2017	Health Services Research and Managerial Epidemiolo gy	4	NA	9-Jan	10.1177/23 333928177 02760	Jackson 2017	Irrelevant	Excluded by TI/AB review
1471	Skills, expertise and role of Australian emergency clinicians in caring for people with advanced cancer	Jelinek, G. A.; Marck, C. H.; Weil, J.; Lane, H.; Philip, J.; Boughey, M.; Weiland, T. J.	2017	BMJ supportive & palliative care	7	1	81-87	10.1136/bm jspcare-2014-000671	Jelinek 2017	Irrelevant	Excluded by TI/AB review
1472	Estimating the Public Health Impact of Air Pollution for	Johnson, J. E.; Bael, D. L.; Sample, J. M.; Lindgren, P. G.; Kvale, D. L.	2017	Journal of public health management	23 Suppl 5 Supple ment,	NA	S45-S52	10.1097/PH H.0000000 000000613	Johnson 2017	Irrelevant	Excluded by TI/AB review
1473	Estimating the Public Health Impact of Air Pollution for Informing Policy in the Twin Cities: [illegible]	Johnson, Jean E.; Bael, David L.; Sample, Jeannette M.; Lindgren, Paula G.; Kvale, Dorian L.	2017	Journal of Public Health Managemen t & Practice	23	NA	S45-S52	10.1097/PH H.0000000 000000613	Johnson 2017	Irrelevant	Excluded by TI/AB review
1474	Affordability of and Access to Information About Health Insurance Among Immigrant and Non- [illegible]	Kang, Ye; McCormick, Danny; Zallman, Leah	2017	Journal of Immigrant & Minority Health	19	4	929-938	10.1007/s10 903-016-0479-y	Kang 2017	Irrelevant	Excluded by TI/AB review

S4 Table. All studies identified in the literature search

1475	Sepsis recidivism: Return visits and recurrence (S3R Analysis)	Kaplan, M.; Baker Chowdhury, M. A.; Hurwitz, J.; Metellus, V.; Martin, E.; Elie, M. C.	2017	Annals of Emergency Medicine	70	4	S60	NA	Kaplan 2017	Irrelevant	Excluded by TI/AB review
1476	Patient satisfaction following educational ultrasounds in the emergency [illegible]	Kapur, P.; Betz, M.; Chenkin, J.; Brick, C.	2017	Canadian Journal of Emergency Medicine	19	NA	S101	10.1017/cem.2017.270	Kapur 2017	Irrelevant	Excluded by TI/AB review

S4 Table. All studies identified in the literature search

1477	Acceptability of HIV Testing Sites Among Rural and Urban African Americans Who Use Cocaine	Keith Branham, D.; Borders, T. F.; Stewart, K. E.; Curran, G. M.; Booth, B. M.	2017	AIDS and behavior	21	2	576-586	10.1007/s10461-016-1527-2	KeithBranham 2017	Irrelevant	Excluded by TI/AB review
1478	Categorical Risk Perception Drives Variability in Antibiotic Prescribing in the Emergency Department: A [illegible]	Klein, E. Y.; Martinez, E. M.; May, L.; Saheed, M.; Reyna, V.; Broniatowski, D. A.	2017	Journal of General Internal Medicine	32	10	1083-1089	10.1007/s11606-017-4099-6	Klein 2017	Irrelevant	Excluded by TI/AB review

S4 Table. All studies identified in the literature search

1479	Social determinants, multimorbidity, and patterns of end-	Koroukian, S. M.; Schiltz, N. K.; Warner, D. F.; Given, C. W.;	2017	Journal of Geriatric Oncology	8	2	117-124	10.1016/j.jgo.2016.10.001	Koroukian 2017	Irrelevant	Excluded by TI/AB review
1480	Design of a pragmatic trial in minority children presenting to the emergency department with uncontrolled [illegible]	Krishnan, J. A.; Martin, M. A.; Lohff, C.; Mosnaim, G. S.; Margellos-Anast, H.; DeLisa, J. A.; McMahon, K.; Erwin, K.; Zun, L. [illegible]	2017	Contemporary Clinical Trials	57	NA	22-Oct	10.1016/j.cct.2017.03.015	Krishnan 2017	Irrelevant	Excluded by TI/AB review
1481	A qualitative study to identify parents' perceptions of and barriers to asthma management in children from South Asian and White British [illegible]	Lakhanpaul, M.; Culley, L.; Robertson, N.; Bird, D.; Hudson, N.; Johal, N.; McFeeters, M.; Angell, E.; Hamlyn-Williams, C.; Abbas, N.; [illegible]	2017	BMC Pulmonary Medicine	17	1	NA	10.1186/s12890-017-0464-9	Lakhanpaul 2017	Irrelevant	Excluded by TI/AB review
1483	Novel malignant bowel obstruction (MBO) management program for women with advanced gynecological cancer	Lee, Y. C.; Jivraj, N.; O'Brien, C.; Lau, J.; Chawla, T.; Shlomovitz, E.; Buchanan, S.; Croke, J.; Allard, J. P.; Dhar, P.; Laframboise, S.; Ferguson, S. E.; Dhani, N. C.; Butler, [illegible]	2017	Journal of Clinical Oncology	35	31	158	10.1200/JCO.2017.35.31_suppl.158	Lee 2017	Irrelevant	Excluded by TI/AB review

S4 Table. All studies identified in the literature search

1484	Association of structured asynchronous virtual visits for hypertension follow-up in primary care with [illegible]	Levine, D. M.; Dixon, R. F.; Linder, J. A.	2017	Journal of General Internal Medicine	32	2	S124-S125	NA	Levine 2017	Irrelevant	Excluded by TI/AB review
1485	Informed consent for opt-in HIV testing via tablet kiosk: an assessment of patient [illegible]	Lewis, M. K.; Hsieh, Y. H.; Gaydos, C. A.; Peterson, S. C.; Rothman, R. E.	2017	International Journal of STD and AIDS	28	13	1292-1298	10.1177/0956462417701009	Lewis 2017	Irrelevant	Excluded by TI/AB review
1486	Triangle inter professional partners for prevention (TIPP): Students collaborating to improve care for [illegible]	Li, T.; Broadhurst, R.; Zomorodi, M.; Jarmul, J. A.; Jones, A.; Skavroneck, S.; Park, M.; Weil, A.	2017	Journal of General Internal Medicine	32	2	S805-S806	NA	Li 2017	Irrelevant	Excluded by TI/AB review

S4 Table. All studies identified in the literature search

1487	Ethnic differences in pain management of long bone fractures in a pediatric rural population	Liao, L.; Reyes, L.	2017	Academic Emergency Medicine	24	NA	S46-S47	10.1111/acem.13203	Liao 2017	Irrelevant	Excluded by TI/AB review
1488	Sleep quality mediates race-related differences in pain intensity among innercity	Lillis, T. A.; Aranda, F.; Burns, J.; Burgess, H.; Purim-Shem-Tov, Y.; Hobfoll, S. E.	2017	Sleep	40	NA	A375-A376	NA	Lillis 2017	Irrelevant	Excluded by TI/AB review

S4 Table. All studies identified in the literature search

1490	A randomized comparative effectiveness trial of a transitional care clinic: 180-day effects	Liss, D. T.; Schaeffer-Pettigrew, C.; Finch, E.; Cooper, A. J.; Sheth, A.; Tejuosho, A. D.; Teter, C.; Ackermann, R. T.	2017	Journal of General Internal Medicine	32	2	S100	NA	Liss 2017	Irrelevant	Excluded by TI/AB review
1491	A quality improvement project to increase utilization of interpretive services for limited English proficiency patients seeking [illegible]	Lozoya, Evelyn	2017	NA	78	NA	NA	NA	Lozoya 2017	Irrelevant	Excluded by TI/AB review
1492	Is racism a factor in emergency department care of patients with suspected acute coronary [illegible]	Mackay, M.; Ratner, P.; Veenstra, G.; Scheuermeyer, F.; Grubisic, M.; Ramanathan, K.; Murray, C.; [illegible]	2017	Canadian Journal of Cardiology	33	10	S125-S126	10.1016/j.cjca.2017.07.244	Mackay 2017	Irrelevant	Excluded by TI/AB review

S4 Table. All studies identified in the literature search

1493	Emergency Department Use in the Perinatal Period: An Opportunity for Early Intervention	Malik, S.; Kothari, C.; MacCallum, C.; Liepman, M.; Tareen, S.; Rhodes, K. V.	2017	Annals of Emergency Medicine	70	6	835-839	10.1016/j.annemergmed.2017.06.020	Malik 2017	Irrelevant	Excluded by TI/AB review
1494	Quality of Life and Health Outcomes in Overweight and Non-Overweight Children With Asthma	Manion, A. B.; Velsor-Friedrich, B.	2017	Journal of pediatric health care : official publication of National Association of Pediatric Nurse Associates & Practitioners	31	1	37-45	10.1016/j.pedhc.2016.01.005	Manion 2017	Irrelevant	Excluded by TI/AB review
1495	An intensive transition navigator intervention improves transition readiness to adult care in youth with sickle cell disease	Manwani, D.; Davidson, L.; Silver, E.; Chhabra, R.; Minniti, C.; Rastogi, D.; Doyle, M.; Morrone, K. A.; Stein, R.; Oyeku, S.; Bauman, L.	2017	Blood	130	NA	NA	NA	Manwani 2017	Irrelevant	Excluded by TI/AB review

S4 Table. All studies identified in the literature search

1496	Is It Okay To Ask: Transgender Patient Perspectives on Sexual Orientation and Gender Identity Collection in Healthcare	Maragh-Bass, A. C.; Torain, M.; Adler, R.; Ranjit, A.; Schneider, E.; Shields, R. Y.; Kodadek, L. M.; Snyder, C. F.; German, D.; [illegible]	2017	Academic Emergency Medicine	24	6	655-667	10.1111/acem.13182	Maragh-Bass 2017	Irrelevant	Excluded by TI/AB review
1497	Risks, Benefits, and Importance of Collecting Sexual Orientation and Gender Identity Data in Healthcare Settings: A Multi- [illegible]	Maragh-Bass, A. C.; Torain, M.; Adler, R.; Schneider, E.; Ranjit, A.; Kodadek, L. M.; Shields, R.; German, D.; Snyder, C.; Peterson, S.; [illegible]	2017	LGBT Health	4	2	141-152	10.1089/lgbt.2016.0107	Maragh-Bass 2017	Irrelevant	Excluded by TI/AB review
1499	Correlates of suicidality in firefighter/EMS personnel	Martin, Colleen E.; Tran, Jana K.; Buser, Sam J.	2017	Journal of Affective Disorders	208	NA	177-183	10.1016/j.jad.2016.08.078	Martin 2017	Irrelevant	Excluded by TI/AB review
1500	Assessing compassion fatigue among emergency department professionals	Martin, E.; Elie, M. C.	2017	Annals of Emergency Medicine	70	4	S36	NA	Martin 2017	Irrelevant	Excluded by TI/AB review

S4 Table. All studies identified in the literature search

1501	Looking forward	McMillan, Tom M.; Ponsford, Jennie; Cullen, Breda; McMillan, Tom M.; Wood, Rodger Ll	2017	Neurobehavioural disability and social handicap following traumatic brain [illegible]	NA	NA	246-255	10.4324/9781315684710-18	McMillan 2017	Irrelevant	Excluded by TI/AB review
1502	Systolic Blood Pressure and Biochemical Assessment of Adherence: A Cross-Sectional Analysis in the [illegible]	McNaughton, C. D.; Brown, N. J.; Rothman, R. L.; Liu, D.; Kabagambe, E. K.; Levy, P. D.; Self, W. H.; Storrow, A. B.; Collins, S. P.; [illegible]	2017	Hypertension	70	2	307-314	10.1161/HYPERTENSIONAHA.117.09659	McNaughton 2017	Irrelevant	Excluded by TI/AB review
1503	Bridging the gap: National utilization of emergency services by transgender patients	Moravek, M. B.; Baker, R. M.; Marsh, E. E.; Randolph, J. F.	2017	Fertility and Sterility	108	3	e41	NA	Moravek 2017	Irrelevant	Excluded by TI/AB review
1504	Adrenaline in anaphylaxis treatment and self-administration: experience from an inner city	Mostmans, Y.; Grosber, M.; Blykers, M.; Mols, P.; Naeije, N.; Gutermuth, J.	2017	Allergy: European Journal of Allergy and Clinical Immunolog	72	3	492-497	10.1111/all.13060	Mostmans 2017	Irrelevant	Excluded by TI/AB review

S4 Table. All studies identified in the literature search

1505	Mindfulness: Past, present and future perspectives	Muireadhach, Boyd; Colin, Griogair	2017	Perspectives on cognitive psychology	NA	NA	NA	NA	Muireadhach 2017	Irrelevant	Excluded by TI/AB review
1506	Racial and social disparities on aggressiveness of end-of-life cancer care in a rural academic center	Namireddy, P.; Macherla, S.; McClain, J. T.; Muzafiar, M.	2017	Journal of Clinical Oncology	35	15	NA	NA	Namireddy 2017	Irrelevant	Excluded by TI/AB review
1507	Equality during end-of-life cancer care: Trends in aggressiveness of cancer care at the end of life	Namireddy, P.; McLawhorn, V.; Jonnalagadda, S.; Muzaffar, M.	2017	Journal of Clinical Oncology	35	31	77	10.1200/JCO.2017.35.31_suppl.77	Namireddy 2017	Irrelevant	Excluded by TI/AB review

S4 Table. All studies identified in the literature search

1508	Early palliative care is associated with improved quality of end-of-life care for womenwith high [illegible]	Nevadunsky, N. S.; Zanartu, C.; Pinto, P.; Barrera, R.; Van Arsdale, A. R.; Kuo, Y. S.; Rapkin, B. D.; Novetsky, A. P.; [illegible]	2017	Gynecologic Oncology	145	NA	26	10.1016/j.ygyno.2017.03.074	Nevadunsky 2017	Irrelevant	Excluded by TI/AB review
1509	Safer transitioning Optimising Frail Elderly Patients Care From Hospital to Home	O'Riordan, Yvonne; Bernard, Paul; Maloney, Paul; Enright, Alison; McGrath, Carmel	2017	International Journal of Integrated Care (IJIC)	17	NA	2-Jan	10.5334/ijic.3911	O'Riordan 2017	Irrelevant	Excluded by TI/AB review
1510	A community-partnered, participatory, cluster-randomized study of depression care quality [illegible]	Ong, Michael K.; Jones, Loretta; Aoki, Wayne; Belin, Thomas R.; Bromley, Elizabeth; Chung, Bowen; Dixon, Elizabeth; Johnson, [illegible]	2017	Psychiatric Services	68	12	1262-1270	10.1176/appi.ps.201600488	Ong 2017	Irrelevant	Excluded by TI/AB review

S4 Table. All studies identified in the literature search

1511	Plastic surgery telehealth consultation expedites Emergency Department treatment	Paik, A. M.; Granick, M. S.; Scott, S.	2017	Journal of Telemedicine and Telecare	23	2	321-327	10.1177/1357633X16639459	Paik 2017	Irrelevant	Excluded by TI/AB review
1513	Tea consumption and health-related quality of life in older adults	Pan, C. W.; Ma, Q.; Sun, H. P.; Xu, Y.; Luo, N.; Wang, P.	2017	Journal of Nutrition, Health and Aging	21	5	480-486	10.1007/s12603-016-0784-0	Pan 2017	Irrelevant	Excluded by TI/AB review
1514	Caring for Patients or Organs: New Therapies Raise New Dilemmas in the Emergency Department	Prabhu, A.; Parker, L. S.; DeVita, M. A.	2017	The American journal of bioethics : AJOB	17	5	16-Jun	10.1080/15265161.2017.1299239	Prabhu 2017	Irrelevant	Excluded by TI/AB review

S4 Table. All studies identified in the literature search

1515	Choosing wisely imaging recommendations: Initial implementation in new England	Raja, A. S.; Venkatesh, A.; Mick, N.; Zabbo, C. P.; Hasegawa, K.; Espinola, J. A.; Bittner, J. C.; [illegible]	2017	Western Journal of Emergency Medicine	18	3	454-458	10.5811/westjem.2017.1.32677	Raja 2017	Irrelevant	Excluded by TI/AB review
1517	Racial disparity in survival from estrogen and progesterone receptor-positive breast cancer: [illegible]	Rauscher, G. H.; Silva, A.; Pauls, H.; Frasor, J.; Bonini, M. G.; Hoskins, K.	2017	Breast Cancer Res Treat	163	2	321-330	10.1007/s10549-017-4166-z	Rauscher 2017	Irrelevant	Excluded by TI/AB review
1519	Emergency Department Use in a Cohort of Older Homeless Adults: Results From the HOPE HOME Study	Raven, M. C.; Tieu, L.; Lee, C. T.; Ponath, C.; Guzman, D.; Kushel, M.	2017	Academic Emergency Medicine	24	1	63-74	10.1111/acem.13070	Raven 2017	Irrelevant	Excluded by TI/AB review
1520	Racial disparities in the diagnosis and management of trigeminal neuralgia	Reinard, K.; Nerenz, D. R.; Basheer, A.; Tahir, R.; Jelsema, T.; Schultz, L.; Malik, G.; Air, E. L.; Schwalb, J. M.	2017	J Neurosurg	126	2	368-374	10.3171/2015.11.Jns151177	Reinard 2017	Irrelevant	Excluded by TI/AB review

S4 Table. All studies identified in the literature search

1521	Outcome of a transitional care clinic to reduce COPD readmissions in a high-risk inner city [illegible]	Reyes, F.; Lee, J.; Rahman, M.; Ramirez, M.	2017	Chest	152	4	A780	10.1016/j.chest.2017.08.811	Reyes 2017	Irrelevant	Excluded by TI/AB review
1522	Tourmaline US-MM6, an open-label, single-arm, multicenter study evaluating the effectiveness and safety of ixazomib [illegible]	Rifkin, R. M.; Boccia, R. V.; Kambhampati, S.; Richter, J. R.; Usmani, S. Z.; Aiello, J.; Sickler, B.; Schoeneich, M.; [illegible]	2017	Blood	130	NA	NA	NA	Rifkin 2017	Irrelevant	Excluded by TI/AB review
1523	Targeted approaches to improve outcomes for highest-cost patients	Rose, A. J.	2017	Israel Journal of Health Policy Research	6	1	NA	10.1186/s13584-017-0151-6	Rose 2017	Irrelevant	Excluded by TI/AB review
1524	A limited evaluation of the association of race and anesthetic medication administration: A single-center experience with [illegible]	Rosenbloom, J. M.; Senthil, K.; Long, A. S.; Robinson, W. R.; Peeples, K. N.; Fiadjoe, J. E.; Litman, R. S.	2017	Pediatric Anesthesia	27	11	1142-1147	10.1111/pan.13217	Rosenbloom 2017	Irrelevant	Excluded by TI/AB review

S4 Table. All studies identified in the literature search

1525	Technology-Enabled Population Health Management: Two Communities,Äô Use of an Electronic Care Alert System	Rowland, T.; Nielsen-Farrell, J.; Church, K.; Riddell, B.	2017	PM and R	9	5	S75-S84	10.1016/j.pmrj.2017.02.006	Rowland 2017	Irrelevant	Excluded by TI/AB review
1526	The performance of a novel patient reported measure of transitional care in the emergency department	Sabbatini, A. K.; Newson, J.; White, S.; Gallahue, F.	2017	Academic Emergency Medicine	24	NA	S222	10.1111/acem.13203	Sabbatini 2017	Irrelevant	Excluded by TI/AB review
1527	Operative fixation for clavicle fractures - socioeconomic differences persist despite overall population [illegible]	Schairer, W. W.; Nwachukwu, B. U.; Warren, R. F.; Dines, D. M.; Gulotta, L. V.	2017	Journal of Orthopaedic Trauma	31	6	e167-e172	10.1097/BOT.0000000000000820	Schairer 2017	Irrelevant	Excluded by TI/AB review
1530	Assessing the public's comprehension of Dietary guidelines: USE of MyPlate/MyPyramid tools is associated with [illegible]	Schwartz, J.; Vernarelli, J. A.	2017	FASEB Journal	31	1	NA	NA	Schwartz 2017	Irrelevant	Excluded by TI/AB review

S4 Table. All studies identified in the literature search

1531	Opioid prescriptions at emergency department discharge for back pain are associated with lower revisit [illegible]	Scott, C. P.; Shah, M. N.; Batt, R.; Otles, E.; Lester, C. A.; Patterson, B. W.; Sun, J.	2017	Academic Emergency Medicine	24	NA	S14	10.1111/acem.13203	Scott 2017	Irrelevant	Excluded by TI/AB review
1532	Patient Navigation for Patients Frequently Visiting the Emergency Department: A Randomized, Controlled Trial	Seaberg, D.; Elseroad, S.; Dumas, M.; Mendiratta, S.; Whittle, J.; Hyatte, C.; Keys, J.	2017	Academic Emergency Medicine	24	11	1327-1333	10.1111/acem.13280	Seaberg 2017	Irrelevant	Excluded by TI/AB review
1533	The magic clinic: An interdisciplinary geriatric consult clinic embedded in a patient-centered medical home	Simone, M. J.; Padgett, L.	2017	Journal of General Internal Medicine	32	2	S801-S802	NA	Simone 2017	Irrelevant	Excluded by TI/AB review

S4 Table. All studies identified in the literature search

1534	Early primary care follow-up after ED and hospital discharge - does it affect readmissions?	Sinha, S.; Seirup, J.; Carmel, A.	2017	Hospital practice (1995)	45	2	51-57	10.1080/21548331.2017.1283935	Sinha 2017	Irrelevant	Excluded by TI/AB review
1535	Thirty-day and 90-day hospital readmission after outpatient upper extremity hemodialysis access creation	Siracuse, J. J.; Shah, N. K.; Peacock, M. R.; Tahhan, G.; Kalish, J. A.; Rybin, D.; Eslami, M. H.; Farber, A.	2017	Journal of Vascular Surgery	65	5	1376-1382	10.1016/j.jvs.2016.11.023	Siracuse 2017	Irrelevant	Excluded by TI/AB review
1536	Improving self efficacy of medication adherence with literacy appropriate heart failure educational [illegible]	Smith, K. M.; Davidson, B.; Tanabe, P.	2017	Heart and Lung: Journal of Acute and Critical Care	46	3	218	10.1016/j.hrtlng.2017.04.029	Smith 2017	Irrelevant	Excluded by TI/AB review

S4 Table. All studies identified in the literature search

1537	The Role of Tonsillectomy in Adults with Tonsillar Hypertrophy and Obstructive Sleep Apnea	Smith, Matthew M.; Peterson, Ed; Yaremchuk, Kathleen L.	2017	Otolaryngology-Head & Neck Surgery	157	2	331-335	10.1177/0194599817698671	Smith 2017	Irrelevant	Excluded by TI/AB review
1538	Physician variability in management of emergency department patients with chest pain	Smulowitz, P. B.; Barrett, O.; Hall, M. M.; Grossman, S. A.; Ullman, E. A.; Novack, V.	2017	Western Journal of Emergency Medicine	18	4	592-600	10.5811/westjem.2017.2.32747	Smulowitz 2017	Irrelevant	Excluded by TI/AB review
1539	Beyond health insurance: Remaining	Sommers, B. D.; McMurtry, C.; Blendon, R.; Benson,	2017	Journal of General Internal	32	2	S130	NA	Sommers 2017	Irrelevant	Excluded by TI/AB review
1540	Association of neighborhood demographics with out-of-hospital cardiac arrest treatment and outcomes: Where you live may matter	Starks, M. A.; Schmicker, R. H.; Peterson, E. D.; May, S.; Buick, J. E.; Kudenchuk, P. J.; Drennan, I. R.; Herren, H.; Jasti, J.; Sayre, M.; Stub, D.; Vilke, G. M.; Stephens, S. W.; [illegible]	2017	JAMA Cardiology	2	10	1110-1118	10.1001/jamacardio.2017.2671	Starks 2017	Irrelevant	Excluded by TI/AB review

S4 Table. All studies identified in the literature search

1541	The effect of brief case management on emergency department use of frequent users in mental health: Findings of a randomized controlled trial	Stergiopoulos, V.; Gozdzik, A.; Cohen, A.; Guimond, T.; Hwang, S. W.; Kurdyak, P.; Leszcz, M.; Wasylenki, D.	2017	PLoS One	12	8	NA	10.1371/journal.pone.0182157	Stergiopoulos 2017	Irrelevant	Excluded by TI/AB review
1542	First-Trimester Vaginal Bleeding: Patient Expectations When Presenting to the Emergency Department	Strommen, J.; Masullo, L.; Crowell, T.; Moffett, P.	2017	Military medicine	182	11	e1824-e1826	10.7205/MILMED-D-17-00108	Strommen 2017	Irrelevant	Excluded by TI/AB review

S4 Table. All studies identified in the literature search

1543	Pulmonary embolism diagnosis and characteristics of prior potentially-related visits	Taylor, R. A.; Salem, I.; Moore, C.	2017	Academic Emergency Medicine	24	NA	S62	10.1111/acem.13203	Taylor 2017	Irrelevant	Excluded by TI/AB review
1544	Impact of an antimicrobial stewardship intervention on antibiotic prescribing practices for community-acquired acute [illegible]	Toy, C.; Peksa, G.; Wang, S.; Varughese, C.; Won, S.	2017	Open Forum Infectious Diseases	4	NA	S270	10.1093/ofid/ofx163.596	Toy 2017	Irrelevant	Excluded by TI/AB review
1545	Referral patterns, delays, and equity in access to advanced paediatric emergency care in [illegible]	Treleaven, E.; Pham, T. N.; Le, D. N.; Brooks, T. N.; Le, H. T.; Partridge, J. C.	2017	International Journal for Equity in Health	16	1	NA	10.1186/s12939-017-0703-y	Treleaven 2017	Irrelevant	Excluded by TI/AB review

S4 Table. All studies identified in the literature search

1547	What factors are associated with patient engagement in low-risk emergency department chest pain patients? A secondary analysis of a multi-center randomized controlled trial	Tschautscher, C. F.; Probst, M. A.; Demers, M.; Leblanc, A.; Lohse, C. M.; Hess, E. P.	2017	Academic Emergency Medicine	24	NA	S16	10.1111/acem.13203	Tschautscher 2017	Irrelevant	Excluded by TI/AB review
1548	Recurrent CSF Rhinorrhea Misdiagnosed as Chronic Allergic Rhinitis with Subsequent Development of Bacterial Meningitis	Ulrich, M. T.; Loo, L. K.; Ing, M. B.	2017	Case Reports in Medicine	2017	NA	NA	10.1155/2017/9012579	Ulrich 2017	Irrelevant	Excluded by TI/AB review
1549	Understanding health equity: A pilot project to collect sociodemographic information on emergency department [illegible]	Vaillancourt, S.; McGowan, M.; Semprun, C.; Hannam, P.; Bandiera, G.; Ovens, H. J.	2017	Canadian Journal of Emergency Medicine	19	NA	S119	10.1017/cem.2017.323	Vaillancourt 2017	Irrelevant	Excluded by TI/AB review

S4 Table. All studies identified in the literature search

1550	Exploring the impact of oral health on quality of life of eating disorders patients	Vecchiattini, R.; Manzato, E.; Mobilio, N.; Scardovelli, I.; Pisani, F. A.; Trombelli, L.	2017	Eating and Weight Disorders	22	3	566	10.1007/s40519-017-0388-7	Vecchiattini 2017	Irrelevant	Excluded by TI/AB review
1551	How nurse gender influences patient priority assignments in US emergency departments	Vigil, J. M.; Coulombe, P.; Alcock, J.; Stith, S. S.; Kruger, E.; Cichowski, S.	2017	PAIN	158	3	377-382	10.1097/j.pain.0000000000000725	Vigil 2017	Irrelevant	Excluded by TI/AB review

S4 Table. All studies identified in the literature search

1552	Implementation challenges of conducting a heart failure palliative care intervention pilot trial below the mason-dixon line	Wells, R.; Dionne-Odom, J. N.; Pamboukian, S.; Tallaj, J.; Azuero, A.; Keebler, K.; Ejem, D.; Kvale, E.; Swetz, K.; Steinhauser, K.; Durant, R.; Kono, A.; Bakitas, M.	2017	Journal of Cardiac Failure	23	8	S8	NA	Wells 2017	Irrelevant	Excluded by TI/AB review
1553	Hospitalization rates and predictors of rehospitalization among individuals with advanced cancer in the year after diagnosis	Whitney, R. L.; Bell, J. F.; Tancredi, D. J.; Romano, P. S.; Bold, R. J.; Joseph, J. G.	2017	Journal of Clinical Oncology	35	31	3610-3617	10.1200/JCO.2017.72.4963	Whitney 2017	Irrelevant	Excluded by TI/AB review
1554	Psychoanalytic perspectives on identity and difference: Navigating the divide	Willock, Brent; Bohm, Lori C.; Curtis, Rebecca Coleman	2017	NA	NA	NA	NA	NA	Willock 2017	Irrelevant	Excluded by TI/AB review

S4 Table. All studies identified in the literature search

1555	Does prehospital management by doctors affect outcome in major trauma? A systematic review	Wilson, Stephanie Laura; Gangathimmaih, Vinay; Wilson, Stephanie; Gangathimmaiah, Vinay	2017	Journal of Trauma & Acute Care Surgery	83	5	965-974	10.1097/TA.0000000000001559	Wilson 2017	Irrelevant	Excluded by TI/AB review
1556	Mobility assessments of geriatric emergency department patients: A systematic review	Yadav, K.; Eagles, D.; Perry, J. J.; √âmond, M.	2017	Academic Emergency Medicine	24	NA	S275	10.1111/acem.13203	Yadav 2017	Irrelevant	Excluded by TI/AB review
1557	Humanitarian healthcare: Treating Syrian patients in Israeli hospitals	Young, Savannah Spivey; Lewis, Denise C.; Oshri, Assaf; Gilbey, Peter; Eisenman, Arie; Schuster, Richard J.; Seponski, Desiree M.	2017	International Journal of Human Rights in Healthcare	10	2	95-109	10.1108/IJHRH-10-2016-0018	Young 2017	Irrelevant	Excluded by TI/AB review

S4 Table. All studies identified in the literature search

1558	Impact of patient socio-economic disadvantage and behavioral health on readmissions in Massachusetts' two largest safety net hospitals	Zallman, L.; Rao, S.; Kressin, N. R.; McCormick, D.	2017	Journal of General Internal Medicine	32	2	S218	NA	Zallman 2017	Irrelevant	Excluded by TI/AB review
1559	Healthcare Utilization by Frail, Community-Dwelling Older Veterans: A 1-Year Follow-up Study	Zylberglait Lisigurski, M.; Bueno, Y. A.; Karanam, C.; Andrade, A. D.; Akkineni, S.; Cevallos, V.; Ruiz, J. G.	2017	Southern Medical Journal	110	11	699-704	10.14423/SMJ.0000000000000722	ZylberglaitLisigurski 2017	Irrelevant	Excluded by TI/AB review
1560	Effects of Medicaid expansion on disparities in trauma care and outcomes in young adults	Akande, M.; Minneci, P. C.; Deans, K. J.; Xiang, H.; Chisolm, D. J.; Cooper, J. N.	2018	J Surg Res	228	NA	42-53	10.1016/j.jss.2018.02.058	Akande 2018	Irrelevant	Excluded by TI/AB review
1561	Impact of a physician at triage on emergency department	Allen, B.; Huang, C. W.; Chowdhury, M. A. B.; Balakrishnan, M. P.; Adrien, K.;	2018	Academic Emergency Medicine	25	NA	S69	10.1111/acem.13424	Allen 2018	Irrelevant	Excluded by TI/AB review

S4 Table. All studies identified in the literature search

1562	Academic status does not affect outcome following complex hepato-	Altieri, Maria S.; Yang, Jie; Groves, Donald; Yin, Donglei; Cagino,	2018	Surgical Endoscopy	32	5	2355-2364	10.1007/s00464-017-5931-0	Altieri 2018	Irrelevant	Excluded by TI/AB review
1563	Predictors of repeated acute hospital attendance for asthma in children: A systematic review [illegible]	Ardura-Garcia, C.; Stolbrink, M.; Zaidi, S.; Cooper, P. J.; Blakey, J. D.	2018	Pediatric Pulmonology	53	9	1179-1192	10.1002/ppul.24068	Ardura-Garcia 2018	Irrelevant	Excluded by TI/AB review
1564	A Cross-Sectional Study Evaluating the Use of Free Clinics in Syracuse, NY: Patient Demographics and Barriers to Accessing [illegible]	Arvisais-Anhalt, S.; MacDougall, M.; Rosenthal, M.; Congelosi, P.; Farrell, D. F.; Rosenbaum, P.	2018	J Community Health	43	6	1075-1084	10.1007/s10900-018-0524-y	Arvisais-Anhalt 2018	Irrelevant	Excluded by TI/AB review
1565	How Are Patients Accessing Primary Care Within the Patient-Centered Medical Home? Results From the Veterans Health Administration	Augustine, M. R.; Nelson, K. M.; Fihn, S. D.; Wong, E. S.	2018	Journal of Ambulatory Care Management	41	3	194-203	10.1097/JAC.0000000000000241	Augustine 2018	Irrelevant	Excluded by TI/AB review

S4 Table. All studies identified in the literature search

1566	Processes and outcomes of negotiating diagnostic uncertainty in	Baim-Lance, A.; Black, G.; Eng, J.; Ramsay, A.; Simister, R.; Fulop, N.	2018	Diagnosis	5	3	eA25	10.1515/dx-2018-0055	Baim-Lance 2018	Irrelevant	Excluded by TI/AB review
1567	Missed dialysis and emergency department visits: Assessing risk	Balhara, K. S.; Fisher, L. A.; Ramos, R.; Jaar, B. G.	2018	Academic Emergency Medicine	25	NA	S187-S188	10.1111/acem.13424	Balhara 2018	Irrelevant	Excluded by TI/AB review
1568	Hospital-based quality measures for pediatric mental health care	Bardach, N. S.; Burkhart, Q.; Richardson, L. P.; Roth, C. P.; Michael Murphy, J.; Parast,	2018	Pediatrics	141	6	NA	10.1542/peds.2017-3554	Bardach 2018	Irrelevant	Excluded by TI/AB review
1573	A cross-sectional study of non-attendance among patients at a US hemophilia treatment center 2010-2014	Barry, V.; Steffens, C.; Mattis, S.; Sidonio, R. F.; Tran, D. Q.; Kempton, C. L.	2018	Haemophilia	24	6	902-910	10.1111/hae.13553	Barry 2018	Irrelevant	Excluded by TI/AB review

S4 Table. All studies identified in the literature search

1574	Challenges to Prehospital Care in Honduras	Bast, Haley E.; Jenkins, J. Lee	2018	Prehospital & Disaster Medicine	33	6	637-639	10.1017/S1049023X1800081X	Bast 2018	Irrelevant	Excluded by TI/AB review
1575	A retrospective study of the effect of emergency medicine clinician performed ultrasound on [illegible]	Baston, C.; Anderson, B. J.; Reilly, J. P.; Shashaty, M. G. S.; Ittner, C. A.; McCarthy, M.; [illegible]	2018	American Journal of Respiratory and Critical Care Medicine	197	MeetingAbstracts	NA	NA	Baston 2018	Irrelevant	Excluded by TI/AB review
1576	Racial differences in presentations and predictors of acute pain after motor vehicle collision	Beaudoin, Francesca L.; Gutman, Roee; Wanting, Zhai; Merchant, Roland C.; Clark, Melissa A.; Bollen, Kenneth A.; Hendry, Phyllis; Kurz, Michael C.; Lewandowski, [illegible]	2018	PAIN	159	6	1056-1063	10.1097/j.pain.0000000000001186	Beaudoin 2018	Irrelevant	Excluded by TI/AB review

S4 Table. All studies identified in the literature search

1579	Improving Care of Inner-City Children with Poorly Controlled Asthma: What Mothers Want [illegible]	Bellin, Melissa H.; Newsome, Angelica; Lewis-Land, Cassie; Kub, Joan; Mudd, Shawna S.; Margolis, Rachel; Butz, Arlene [illegible]	2018	Journal of Pediatric Healthcare	32	4	387-398	10.1016/j.pedhc.2017.12.009	Bellin 2018	Irrelevant	Excluded by TI/AB review
1580	Quantification of intensive care unit care in an urban emergency department	Bissonette, A.; Jayaprakash, N.; Hrabec, D.; Pflaum-Carlson, J.; Gardner-Gray, J.; Hurst, G.; Coba, V.; Rivers, E.; Miller, J.	2018	Academic Emergency Medicine	25	NA	S228	10.1111/acem.13424	Bissonette 2018	Irrelevant	Excluded by TI/AB review
1581	Health system costs of potentially inappropriate prescribing in Ontario, Canada: A protocol for a population-based cohort study	Black, C. D.; Thavorn, K.; Coyle, D.; Smith, G.; Bjerre, L. M.	2018	BMJ Open	8	6	NA	10.1136/bmjopen-2018-021727	Black 2018	Irrelevant	Excluded by TI/AB review
1582	Blood pressure variability is associated with delayed time to vasopressors in early septic shock	Black, L.; Smotherman, C.; Gautam, S.; Miller, T.; Guirgis, F.	2018	Critical Care Medicine	46	NA	738	10.1097/01.ccm.0000529510.96655.c2	Black 2018	Irrelevant	Excluded by TI/AB review

S4 Table. All studies identified in the literature search

1583	Blood pressure fluctuations prior to vasopressor administration in early septic shock are not associated with worsening organ failure 48 [illegible]	Black, L. P.; Smotherman, C.; Miller, T.; Gautam, S.; Guirgis, F. W.	2018	Academic Emergency Medicine	25	NA	S151-S152	10.1111/acem.13424	Black 2018	Irrelevant	Excluded by TI/AB review
1584	Factors Influencing Physician Assistant Students' Attitudes Toward Deservingness of Care for Undocumented Mexicans	Bowen, D. J.; Mickus, M.; Rosales, A. N.	2018	J Physician Assist Educ	29	1	35-38	10.1097/jpa.0000000000000185	Bowen 2018	Irrelevant	Excluded by TI/AB review
1587	Barriers to Care for Persons With Sickle Cell Disease: The Case Manager's Opportunity to Improve Patient Outcomes	Brennan-Cook, J.; Bonnabeau, E.; Aponte, R.; Augustin, C.; Tanabe, P.	2018	Professional case management	23	4	213-219	10.1097/NCM.0000000000000260	Brennan-Cook 2018	Irrelevant	Excluded by TI/AB review

S4 Table. All studies identified in the literature search

1588	Improving Emergency Health Care Workers' Knowledge, Competency, and Attitudes Toward Lesbian, Gay, Bisexual, and Transgender Patients Through [illegible]	Bristol, S.; Kostelec, T.; MacDonald, R.	2018	J Emerg Nurs	44	6	632-639	10.1016/j.jen.2018.03.013	Bristol 2018	Irrelevant	Excluded by TI/AB review
1590	Patients' and Clinicians' Perceptions of Antibiotic Prescribing for Upper Respiratory Infections in the Acute Care Setting	Broniatowski, D. A.; Klein, E. Y.; May, L.; Martinez, E. M.; Ware, C.; Reyna, V. F.	2018	Medical decision making : an international journal of the Society for Medical Decision Making	38	5	547-561	10.1177/0272989X18770664	Broniatowski 2018	Irrelevant	Excluded by TI/AB review
1591	FACTORS ASSOCIATED WITH THE INITIATION OF BIOLOGIC DRUGS IN [illegible]	Bukstein, D.	2018	Annals of Allergy, Asthma and Immunology	121	5	S58-S59	10.1016/j.anai.2018.09.192	Bukstein 2018	Irrelevant	Excluded by TI/AB review

S4 Table. All studies identified in the literature search

1592	Patient-reported reasons for seeking emergency care at a freestanding emergency department compared to a hospital-based ED	Burke, R. C.; Simon, E. L.; Kesav, N.; Keaton, B.; Kukral, L.; Kiss, T. J.; Sanchez, J.; Jouriles, N. J.	2018	American Journal of Emergency Medicine	36	9	1702-1704	10.1016/j.ajem.2018.01.062	Burke 2018	Irrelevant	Excluded by TI/AB review
1593	Effect of academic status on outcomes of surgery for rectal cancer	Cagino, K.; Altieri, M. S.; Yang, J.; Nie, L.; Talamini, M.; Spaniolas, K.; Denoya, P.; Pryor, A.	2018	Surgical Endoscopy	32	6	2774-2780	10.1007/s00464-017-5977-z	Cagino 2018	Irrelevant	Excluded by TI/AB review
1594	Understanding health knowledge gaps to optimize transitions of care for young adults [illegible]	Calhoun, C.; Luo, L.; Baumann, A.; James, A.; King, A.	2018	Blood	132	NA	NA	10.1182/blood-2018-99-113150	Calhoun 2018	Irrelevant	Excluded by TI/AB review

S4 Table. All studies identified in the literature search

1595	Implementation and evaluation of summit, an ambulatory intensive care (A-ICU) model of primary care for high-utilizersata healthcare for the homeless site	Chan, B.; Mitchell, M.; Edwards, S. T.; Saha, S.; Korthuis, P. T.	2018	Journal of General Internal Medicine	33	2	791-792	NA	Chan 2018	Irrelevant	Excluded by TI/AB review
1596	Chronic pruritus severity and QoL impact on healthcare utilization among veterans: A [illegible]	Chisolm, S. S.; Chen, S. C.; Yeung, H.	2018	Journal of Investigative Dermatology	138	5	S56	NA	Chisolm 2018	Irrelevant	Excluded by TI/AB review
1597	Transgender and Gender-Nonconforming Patients in the Emergency Department: What Physicians Know, Think, and Do	Chisolm-Straker, M.; Willging, C.; Daul, A. D.; McNamara, S.; Sante, S. C.; Shattuck, D. G.; Crandall, C. S.	2018	Annals of Emergency Medicine	71	2	183-188.e1	10.1016/j.annemergmed.2017.09.042	Chisolm-Straker 2018	Irrelevant	Excluded by TI/AB review

S4 Table. All studies identified in the literature search

1599	Local prevalence of erectile dysfunction post trans-rectal ultrasound guided biopsy of the [illegible]	Chong, W.	2018	BJU International	122	NA	24	NA	Chong 2018	Irrelevant	Excluded by TI/AB review
1600	Insurance Status and Access to Urgent Primary Care Follow-up After an Emergency Department¬†Visit in 2016	Chou, Shih-Chuan; Deng, Yanhong; Smart, Jerry; Parwani, Vivek; Bernstein, Steven L.; Venkatesh, Arjun K.	2018	Annals of Emergency Medicine	71	4	487-496.e1	10.1016/j.annemergmed.2017.08.045	Chou 2018	Irrelevant	Excluded by TI/AB review
1602	Unique Stroke Symptoms in Women: A Review	Colsch, R.; Lindseth, G.	2018	Journal of Neuroscience Nursing	50	6	336-342	10.1097/JNN.0000000000000402	Colsch 2018	Irrelevant	Excluded by TI/AB review

S4 Table. All studies identified in the literature search

1603	Erectile dysfunction, twenty years after	Colson, M. H.; Cuzin, B.; Faix, A.; Grellet, L.; Huyghes, E.	2018	Sexologies	27	1	e1-e6	10.1016/j.sexol.2018.01.016	Colson 2018	Irrelevant	Excluded by TI/AB review
1605	Consensus-based clinical research priorities for emergency nursing in Australia	Considine, J.; Curtis, K.; Shaban, R. Z.; Fry, M.	2018	Australasian emergency care	21	2	43-50	10.1016/j.auec.2018.03.001	Considine 2018	Irrelevant	Excluded by TI/AB review
1607	Nonuremic calciphylaxis-an infrequent diagnosis without	Cote, P.; Swanson, K.; Ceesay, M.; Gannon, N.; Bhandari, S.; Jha, P.	2018	Journal of Hospital Medicine	13	4	NA	NA	Cote 2018	Irrelevant	Excluded by TI/AB review
1608	Copd care in the 21st century: A public health priority	Criner, R. N.; Han, M. K.	2018	Respiratory Care	63	5	591-600	10.4187/respcare.06276	Criner 2018	Irrelevant	Excluded by TI/AB review
1609	Trauma Patients' Attitudes toward Organ Donation at an Urban Level I Trauma Center	Cull, J. D.; Ivkovic, K.; Manning, B.; Chan, E. Y.	2018	Am Surg	84	9	1493-1498	NA	Cull 2018	Irrelevant	Excluded by TI/AB review

S4 Table. All studies identified in the literature search

1611	Can telemedicine-assisted para-medicine reduce emergency department visits and 30-day readmissions among high-risk heart failure patients? A pilot study	Daniels, B.; Del Toro, C.; Zelenetz, M.; Gogia, K.; Hafeez, B.; Greenwald, P.; Clark, S.; Steel, P.; Hsu, H.; Goyal, P.; Horn, E.; Sharma, R.	2018	Circulation	138	NA	NA	NA	Daniels 2018	Irrelevant	Excluded by TI/AB review
1612	Sociodemographic Influences of Emergency Department Care for Anxiety Disorders	Dark, T.; Rust, G.; Flynn, H. A.; Kinsell, H.; Harman, J. S.	2018	The journal of behavioral health services & research	45	4	593-604	10.1007/s11414-018-9598-7	Dark 2018	Irrelevant	Excluded by TI/AB review

S4 Table. All studies identified in the literature search

1615	Risk factors for severe hypoglycaemia in community-based patients with type 2 diabetes: The Fremantle Diabetes Study Phase II	Davis, T. M. E.; Davis, W. A.	2018	Diabetologia	61	NA	S452	10.1007/s00125-018-4693-0	Davis 2018	Irrelevant	Excluded by TI/AB review
1616	Integrated care and health disparities: Critical patient-provider considerations	Davis, Tamara S.; Peck, Adriane; Rooney, Ronald H.; Mirick, Rebecca G.	2018	Strategies for work with involuntary clients., 3rd ed.	NA	NA	382-404	10.7312/roon18266-015	Davis 2018	Irrelevant	Excluded by TI/AB review
1617	Exploring factors associated with asthma-related emergency department visits among adults: A path analysis approach	Deshpande, M.; Look, K. A.	2018	Research in Social & Administrative Pharmacy	14	1	46-52	10.1016/j.sapharm.2016.12.011	Deshpande 2018	Irrelevant	Excluded by TI/AB review

S4 Table. All studies identified in the literature search

1618	Women,Äôs Healthcare Utilization Following Routine Screening for Past-Year Intimate Partner Violence in the Veterans Health Administration	Dichter, M. E.; Sorrentino, A. E.; Haywood, T. N.; Bellamy, S. L.; Medvedeva, E.; Roberts, C. B.; Iverson, K. M.	2018	Journal of General Internal Medicine	33	6	936-941	10.1007/s11606-018-4321-1	Dichter 2018	Irrelevant	Excluded by TI/AB review
1619	Patterns of follow up after emergency room ophthalmic consultation	Dong, C. Q.; Farhat, B.; Kruh, J. N.	2018	Investigative Ophthalmology and Visual Science	59	9	NA	NA	Dong 2018	Irrelevant	Excluded by TI/AB review
1620	Benchmarking emergency department thoracotomy: Using trauma video review to generate procedural norms	Dumas, R. P.; Chreiman, K. M.; Seamon, M. J.; Cannon, J. W.; Reilly, P. M.; Christie, J. D.; Holena, D. N.	2018	Injury	49	9	1687-1692	10.1016/j.injury.2018.05.010	Dumas 2018	Irrelevant	Excluded by TI/AB review

S4 Table. All studies identified in the literature search

1621	Association Between Perceived Discrimination and Emergency Department Use Among Safety-Net Patients in the Southwestern United States	Enard, K. R.; Nevarez, L.; Ganelin, D. M.	2018	Southern Medical Journal	111	1	7-Jan	10.14423/SMJ.0000000000000753	Enard 2018	Irrelevant	Excluded by TI/AB review
1622	Approaches to community consultation in exception from informed consent: Analysis of scope, efficiency, and cost at two centers	Eubank, L.; Lee, K. S.; Seder, D. B.; Strout, T.; Darrow, M.; MacDonald, C.; May, T.; Riker, R. R.; Kern, K. B.	2018	Resuscitation	130	NA	81-87	10.1016/j.resuscitation.2018.06.031	Eubank 2018	Irrelevant	Excluded by TI/AB review

S4 Table. All studies identified in the literature search

1623	Patient nonadherence to filling discharge medication prescriptions from the emergency department: Barriers and clinical implications	Farris, B.; Shakowski, C.; Mueller, S. W.; Phong, S.; Kiser, T. H.; Jacknin, G.	2018	American Journal of Health-System Pharmacy	75	5	316-320	10.2146/ajhp170198	Farris 2018	Irrelevant	Excluded by TI/AB review
1624	Sex Differences in Health Care Utilization, End‚ÄêStage Renal¬†Disease, and Mortality	Feldman, Candace H.; Broder, Anna; Guan, Hongshu; Yazdany, Jinoos; Costenbader, Karen H.	2018	Arthritis & Rheumatology	70	3	417-426	10.1002/art.40392	Feldman 2018	Irrelevant	Excluded by TI/AB review
1625	Racial differences in objective and subjective sleep parameters: A sample of inner-city women	Fischer, A.; Aranda, F.; Lillis, T. A.; Burgess, H. J.; Burns, J. W.; Purim-Shem-Tov, Y.; Hobfoll, S. E.	2018	Sleep	41	NA	A263-A264	NA	Fischer 2018	Irrelevant	Excluded by TI/AB review

S4 Table. All studies identified in the literature search

1626	A fixed nitrous oxide/oxygen mixture as an analgesic for trauma patients in emergency department: study [illegible]	Gao, L. L.; Yang, L. S.; Zhang, J. J.; Wang, Y. L.; Feng, K.; Ma, L.; Yu, Y. Y.; Li, Q.; Wang, Q. H.; Bao, J. T.; Dai, Y. L.; Liu, Q.; Li, Y. [illegible]	2018	Trials	19	NA	NA	10.1186/s13063-018-2899-6	Gao 2018	Irrelevant	Excluded by TI/AB review
1627	Predictors of Hospice Enrollment for Patients With Advanced Heart Failure and Effects [illegible]	Gelfman, L. P.; Barr√≥n, Y.; Moore, S.; Murtaugh, C. M.; Lala, A.; Aldridge, M. D.; Goldstein, N. E.	2018	JACC: Heart Failure	6	9	780-789	10.1016/j.jchf.2018.04.009	Gelfman 2018	Irrelevant	Excluded by TI/AB review
1628	Urologists' attitudes to sexual complications of LUTS/BPH	Giona, S.; Ganguly, I.; Muir, G.	2018	World journal of urology	36	9	1449-1453	10.1007/s00345-018-2283-x	Giona 2018	Irrelevant	Excluded by TI/AB review
1629	Avoidable visits to the emergency department (ED) and their association with sex, age and race in a cohort of low socio-economic status patients on hemodialysis in the Bronx	Golestaneh, L.; Bellin, E.; Neugarten, J.; Lo, Y.	2018	PLoS One	13	8	NA	10.1371/journal.pone.0202697	Golestaneh 2018	Irrelevant	Excluded by TI/AB review

S4 Table. All studies identified in the literature search

1630	The public health impact of parent-reported childhood food allergies in the United States	Gupta, R. S.; Warren, C. M.; Smith, B. M.; Blumenstock, J. A.; Jiang, J.; Davis, M. M.; Nadeau, K. C.	2018	Pediatrics	142	6	NA	10.1542/peds.2018-1235	Gupta 2018	Irrelevant	Excluded by TI/AB review
1631	Assessment of Patient-Centered Approaches to Collect Sexual Orientation and Gender Identity Information in the Emergency [illegible]	Haider, A.; Adler, R. R.; Schneider, E.; Leitz, T. U.; Ranjit, A.; Ta, C.; Levine, A.; Harfouch, O.; Pelaez, D.; Kodadek, L.; Vail, L.; Snyder, C.; German, D.; [illegible]	2018	JAMA Network Open	1	8	NA	10.1001/jamanetworkopen.2018.6506	Haider 2018	Irrelevant	Excluded by TI/AB review
1634	Socioeconomics affecting quality outcomes in Asian trauma patients within the United States	Haines, K. L.; Agarwal, S.; Jung, H. S.	2018	J Surg Res	228	NA	63-67	10.1016/j.jss.2018.02.060	Haines 2018	Irrelevant	Excluded by TI/AB review

S4 Table. All studies identified in the literature search

1635	Caseworker-assigned discharge plans to prevent hospital readmission for acute exacerbations in [illegible]	Hall, K. K.; Petsky, H. L.; Chang, A. B.; O'Grady, K. F.	2018	Cochrane Database of Systematic Reviews	2018	11	NA	10.1002/14651858.CD012315.pub2	Hall 2018	Irrelevant	Excluded by TI/AB review
1636	Effect of the school-based telemedicine enhanced asthma management (SB-TEAM) program on asthma morbidity: A [illegible]	Halterman, J. S.; Fagnano, M.; Tajon, R. S.; Tremblay, P.; Wang, H.; Butz, A.; Perry, T. T.; McConnochie, K. M.	2018	JAMA Pediatrics	172	3	NA	10.1001/jamapediatrics.2017.4938	Halterman 2018	Irrelevant	Excluded by TI/AB review
1638	An emergency department team-based quality improvement initiative reduces narcotic and [illegible]	Harris, D.; Karogiannis, D.; Balfour, N.; Gerry, M.; Mushta, J.; Cabral, J.	2018	Canadian Journal of Emergency Medicine	20	NA	S50	10.1017/cem.2018.180	Harris 2018	Irrelevant	Excluded by TI/AB review

S4 Table. All studies identified in the literature search

1639	Health care use and health behaviors among young adults with history of parental incarceration	Heard-Garris, N.; Winkelman, T. N. A.; Choi, H.; Miller, A. K.; Kan, K.; Shlafer, R.; Davis, M. M.	2018	Pediatrics	142	3	NA	10.1542/peds.2017-4314	Heard-Garris 2018	Irrelevant	Excluded by TI/AB review
1640	Health and social work: Practice, policy, and research	Heyman, Janna C.; Congress, Elaine P.	2018	NA	NA	NA	NA	10.1891/9780826141644	Heyman 2018	Irrelevant	Excluded by TI/AB review
1641	Association Between Medication Adherence and the Outcomes of Heart Failure	Hood, S. R.; Giazzon, A. J.; Seamon, G.; Lane, K. A.; Wang, J.; Eckert, G. J.; Tu, W.; Murray, M. D.	2018	Pharmacotherapy	38	5	539-545	10.1002/phar.2107	Hood 2018	Irrelevant	Excluded by TI/AB review

S4 Table. All studies identified in the literature search

1642	Intervening to improve family and child asthma self-management	Horner, S. D.	2018	American Journal of Respiratory and Critical Care Medicine	197	MeetingAbstracts	NA	NA	Horner 2018	Irrelevant	Excluded by TI/AB review
1643	Reduction of Peripartum Racial and Ethnic Disparities A Conceptual	Howell, E. A.; Brown, H.; Brumley, J.; Bryant, A. S.; Caughey, A. B.; Cornell, A. M.;	2018	Obstetrics and Gynecology	131	5	770-782	10.1097/AOG.0000000000002475	Howell 2018	Irrelevant	Excluded by TI/AB review
1644	Reduction of Peripartum Racial and Ethnic Disparities: A	Howell, E. A.; Brown, H.; Brumley, J.; Bryant, A. S.; Caughey, A. B.;	2018	Jognn-Journal of Obstetric Gynecologi	47	3	275-289	10.1016/j.jogn.2018.03.004	Howell 2018	Irrelevant	Excluded by TI/AB review
1645	Reduction of Peripartum Racial and Ethnic Disparities: A Conceptual Framework and Maternal Safety Consensus Bundle	Howell, E. A.; Brown, H.; Brumley, J.; Bryant, A. S.; Caughey, A. B.; Cornell, A. M.; Grant, J. H.; Gregory, K. D.; Gullo, S. M.; Kozhimannil, K. B.; [illegible]	2018	Journal of Midwifery & Womens Health	63	3	366-376	10.1111/jmwh.12756	Howell 2018	Irrelevant	Excluded by TI/AB review

S4 Table. All studies identified in the literature search

1646	Increased length of emergency department boarding is not associated with increased mortality	Hrabec, D.; Jayaprakash, N.; Bissonette, A.; Chaudhry, K.; Miller, J.	2018	Academic Emergency Medicine	25	NA	S180	10.1111/acem.13424	Hrabec 2018	Irrelevant	Excluded by TI/AB review
1647	Medicaid Managed Care in Florida and Racial and Ethnic Disparities in Preventable Emergency Department Visits	Hu, T.; Mortensen, K.; Chen, J.	2018	Med Care	56	6	477-483	10.1097/mlr.0000000000000909	Hu 2018	Irrelevant	Excluded by TI/AB review

S4 Table. All studies identified in the literature search

1648	Neighborhood socioeconomic characteristics, healthcare spatial access, and emergency department visits for ambulatory care sensitive conditions for elderly	Huang, Y.; Meyer, P.; Jin, L.	2018	Prev Med Rep	12	NA	101-105	10.1016/j.pmedr.2018.08.015	Huang 2018	Irrelevant	Excluded by TI/AB review
1650	Association of shared decision-making on patient-reported health outcomes and healthcare utilization	Hughes, T. M.; Merath, K.; Chen, Q.; Sun, S.; Palmer, E.; Idrees, J. J.; Okunrintemi, V.; Squires, M.; Beal, E. W.; Pawlik, T. M.	2018	American Journal of Surgery	216	1	12-Jul	10.1016/j.amjsurg.2018.01.011	Hughes 2018	Irrelevant	Excluded by TI/AB review

S4 Table. All studies identified in the literature search

1651	Population and patient factors affecting emergency department attendance in [illegible]	Hull, S. A.; Homer, K.; Boomla, K.; Robson, J.; Ashworth, M.	2018	British Journal of General Practice	68	668	e157-e167	10.3399/bjgp18X694397	Hull 2018	Irrelevant	Excluded by TI/AB review
1652	Developing Standardized ,ÄúReceiver-Driven,Äù Handoffs Between Referring Providers and the Emergency [illegible]	Huth, K.; Stack, A. M.; Chi, G.; Shields, R.; Jorina, M.; West, D. C.; Landrigan, C. P.; Spector, N. D.; Starmer, A. J.	2018	Joint Commission Journal on Quality and Patient Safety	44	12	719-730	10.1016/j.jcjq.2018.05.003	Huth 2018	Irrelevant	Excluded by TI/AB review
1653	Using case management in a universal health coverage system to improve quality of life of frequent Emergency Department users: A randomized [illegible]	Iglesias, Katia; Baggio, St√©phanie; Moschetti, Karine; Wasserfallen, Jean-Blaise; Hugli, Olivier; Daeppen, Jean-Bernard; Burnand, Bernard; Bodenmann, Patrick	2018	Quality of Life Research: An International Journal of Quality of Life Aspects of [illegible]	27	2	503-513	10.1007/s11136-017-1739-6	Iglesias 2018	Irrelevant	Excluded by TI/AB review
1655	Restricted fluid bolus versus current practice in children with septic shock: The fish feasibility study and pilot [illegible]	Inwald, D.; Canter, R. R.; Woolfall, K.; O,ÄôHara, C. B.; Mouncey, P. R.; Zenasni, Z.; Hudson, N.; Saunders, S.; Carter, A.; Jones, N.; [illegible]	2018	Health Technology Assessment	22	51	1-105	10.3310/hta22510	Inwald 2018	Irrelevant	Excluded by TI/AB review

S4 Table. All studies identified in the literature search

1656	Peer to peer support and health care utilization among vulnerable older adults	Jacobs, E.; Schwei, R. J.; Broeckling, J.; Frumer, J.; Sauer, V.; DeYoung, K.; Mahoney, J. E.; Hetzel, S.; Kim, K.	2018	Journal of General Internal Medicine	33	2	287-288	NA	Jacobs 2018	Irrelevant	Excluded by TI/AB review
1657	Factors impacting time to antibiotic administration in patients with sepsis: A single-center study of electronic health record data	Jacobs, Z.; Prasad, P.; Fang, M.; Doernberg, S.; Abe-Jones, Y.; Kangelaris, K.	2018	Journal of Hospital Medicine	13	4	NA	NA	Jacobs 2018	Irrelevant	Excluded by TI/AB review
1658	A safe haven for the injured? Urban trauma care at the intersection of healthcare, law enforcement, and race	Jacoby, S. F.; Richmond, T. S.; Holena, D. N.; Kaufman, E. J.	2018	Social Science and Medicine	199	NA	115-122	10.1016/j.socscimed.2017.05.037	Jacoby 2018	Irrelevant	Excluded by TI/AB review

S4 Table. All studies identified in the literature search

1661	Health Care Service Use Among Elderly Seasonal Migrators	Jeffery, Molly Moore; Wolfson, Julian; Meier, Sarah K.; Dowd, Bryan E.; Abraham, Jean M.; Kane, Robert L.	2018	Population Health Managemen t	21	5	415-421	10.1089/po p.2017.015 5	Jeffery 2018	Irrelevant	Excluded by TI/AB review
1662	Enoxaparin dosing errors in the emergency department	Jellinek-Cohen, S. P.; Li, M.; Husk, G.	2018	World Journal of Emergency Medicine	9	3	195-202	10.5847/wje m.j.192086 42.2018.03. 006	Jellinek-Cohen 2018	Irrelevant	Excluded by TI/AB review
1663	Time to CT head in adult patients with suspected traumatic brain injury: Association with the 'Shorter Stays in Emergency [illegible]	Jones, Peter; Athaullah, Waheedah; Harper, Alana; Wells, Susan; LeFevre, James; Stewart, Joanna; Curtis, Elana; Reid, Papaarangi; [illegible]	2018	Injury	49	9	1680-1686	10.1016/j.in jury.2018.0 5.011	Jones 2018	Irrelevant	Excluded by TI/AB review
1664	Pilot study of a randomized trial to evaluate a Web-based intervention targeting adolescents presenting to the emergency department with acute asthma	Joseph, C. L. M.; Mahajan, P.; Stokes-Buzzelli, S.; Johnson, D. A.; Duffy, E.; Williams, R.; Zhang, T.; Ownby, D. R.; Considine, S.; Lu, M.	2018	Pilot and Feasibility Studies	4	1	NA	10.1186/s40 814-017-0147-6	Joseph 2018	Irrelevant	Excluded by TI/AB review

S4 Table. All studies identified in the literature search

1665	Association Between Race, Neighborhood, and Medicaid Enrollment and Outcomes in [illegible]	Joynt Maddox, K. E.; Chen, L. M.; Zuckerman, R.; Epstein, A. M.	2018	J Am Geriatr Soc	66	2	239-246	10.1111/jgs.15082	JoyntMaddox 2018	Irrelevant	Excluded by TI/AB review
1667	Poor dietary guidelines compliance among low-incomewomen eligible for supplemental nutrition assistance program-education (SNAP-Ed)	Jun, S.; Thuppal, S. V.; Maulding, M. K.; Eicher-Miller, H. A.; Savaiano, D. A.; Bailey, R. L.	2018	Nutrients	10	3	NA	10.3390/nu10030327	Jun-18	Irrelevant	Excluded by TI/AB review
1668	PROPER: Development of an Early Pediatric Intensive Care Unit Readmission Risk Prediction Tool	Kaur, H.; Naessens, J. M.; Hanson, A. C.; Fryer, K.; Nemergut, M. E.; Tripathi, S.	2018	Journal of Intensive Care Medicine	33	1	29-36	10.1177/0885066616665806	Kaur 2018	Irrelevant	Excluded by TI/AB review

S4 Table. All studies identified in the literature search

1669	Tensions and Coping Strategies in Ethnically Mixed Teams: Findings from a Study in Two Emergency [illegible]	Keshet, Yael; Abu-Khella, Ashraf; Raviv, Benidor; Popper-Giveon, Ariela; Strizhevski, Alexander	2018	Journal of Immigrant & Minority Health	20	4	951-962	10.1007/s10903-017-0603-7	Keshet 2018	Irrelevant	Excluded by TI/AB review
1670	Obstetric complications and delays in seeking emergency care in poor settings of northern India	Khan, Nizamuddin	2018	International Journal of Human Rights in Healthcare	11	5	333-346	10.1108/IJHRH-10-2017-0057	Khan 2018	Irrelevant	Excluded by TI/AB review
1671	Neighborhood and individual resilience in cardiovascular health of blacks: A multi-level investigation from morehouse-emory cardiovascular (MECA) center	Kim, J. H.; Topel, M.; Baltrus, P.; Pemu, P.; Ko, Y. A.; Lewis, T.; Vaccarino, V.; Liu, C.; Mubasher, M.; Searles, C.; Dunbar, S.; Quyyumi, A.; Taylor, H.	2018	Circulation	138	NA	NA	NA	Kim 2018	Irrelevant	Excluded by TI/AB review

S4 Table. All studies identified in the literature search

1672	Contingent engagement: What we learn from patients with complex health problems and low socioeconomic status	Komaromy, M.; Madden, E. F.; Zurawski, A.; Kalishman, S.; Barker, K.; O'Sullivan, P.; Jurado, M.; Arora, S.	2018	Patient Education and Counseling	101	3	524-531	10.1016/j.pec.2017.08.019	Komaromy 2018	Irrelevant	Excluded by TI/AB review
1673	The Fate of Radical Cystectomy Patients after Hospital Discharge: Understanding the Black Box of the [illegible]	Krishnan, N.; Li, B.; Jacobs, B. L.; Ambani, S. N.; Borza, T.; He, C.; Hollenbeck, B. K.; Morgan, T.; Hafez, K. S.; Weizer, A. Z.; Montgomery, J. S.; [illegible]	2018	European Urology Focus	4	5	711-717	10.1016/j.euf.2016.07.004	Krishnan 2018	Irrelevant	Excluded by TI/AB review
1674	Perception of metal detector and security officer usage in the emergency department	Krywko, D. M.; Allinder, R.; Jokl, P.; Saef, S.	2018	Academic Emergency Medicine	25	NA	S47	10.1111/acem.13424	Krywko 2018	Irrelevant	Excluded by TI/AB review
1675	Quality Improvement and Safety in Pediatric Emergency Medicine	Ku, B. C.; Chamberlain, J. M.; Shaw, K. N.	2018	Pediatric Clinics of North America	65	6	1269-+	10.1016/j.pcl.2018.07.010	Ku 2018	Irrelevant	Excluded by TI/AB review

S4 Table. All studies identified in the literature search

1676	Stroke Severity Affects Timing	Kwei, K. T.; Liang, J.; Wilson, N.; Tuhrim, S.; Dhamoon, M.	2018	Neurologist	23	3	79-82	10.1097/NRL.0000000000000157	Kwei 2018	Irrelevant	Excluded by TI/AB review
1677	Educational Text Messages Decreased Emergency Department Utilization Among Infant Caregivers: A Randomized Trial	Ladley, A.; Hieger, A. W.; Arthur, J.; Broom, M.	2018	Academic Pediatrics	18	6	636-641	10.1016/j.acap.2018.02.003	Ladley 2018	Irrelevant	Excluded by TI/AB review
1678	Understanding Disparities in Breast Cancer Care in Memphis, Tennessee	Lamb, Elena P.; Pritchard, F. Elizabeth; Nouer, Simonne S.; Tolley, Elizabeth A.; Boyd, Brandon S.; Davidson, Jesse T.; Munene, Gitonga; Fleming, Martin D.	2018	American Surgeon	84	5	620-627	10.1177/000313481808400510	Lamb 2018	Irrelevant	Excluded by TI/AB review
1680	The effects of medicaid coverage expansion on health-related quality of life and healthcare [illegible]	Lee, A.; An, J.	2018	Journal of Managed Care and Specialty Pharmacy	24	NA	S49-S50	NA	Lee 2018	Irrelevant	Excluded by TI/AB review

S4 Table. All studies identified in the literature search

1681	Reasons for Accident and Emergency department attendance by people with chronic obstructive pulmonary disease [illegible]	Lee, J. S.; Lempp, H.; Srivastava, V.; Barley, E.	2018	BMJ Open Respiratory Research	5	1	NA	10.1136/bmjresp-2017-000244	Lee 2018	Irrelevant	Excluded by TI/AB review
1682	Exploring the association between clinician burnout and adherence to back pain evaluation [illegible]	Lerner, A.; Griffith, K.; Drainoni, M. L.; Larochelle, M.; Linden, J.; Wallace, E.; Weber, K.; Liebschutz, J. M.	2018	Journal of General Internal Medicine	33	2	192	NA	Lerner 2018	Irrelevant	Excluded by TI/AB review
1683	Promoting resilience in diverse populations affected by youth violence	Levas, M. N.; Melzer-Lange, M.	2018	Pediatrics	141	1	NA	10.1542/peds.141.1-MeetingAbstract.355	Levas 2018	Irrelevant	Excluded by TI/AB review
1684	Predictors and reasons why patients decline to participate in high tech and novel sites of care: A home hospital experience	Levine, D. M.; Burke, K. P.; Paz, M.; Schnipper, J. L.	2018	Journal of General Internal Medicine	33	2	304	NA	Levine 2018	Irrelevant	Excluded by TI/AB review

S4 Table. All studies identified in the literature search

1685	Disparities in Health Literacy and Healthcare Utilization among Adolescents and Young Adults [illegible]	Levine, R.; Javalkar, K.; Nazareth, M.; Faldowski, R. A.; de Ferris, M. D. G.; Cohen, S.; Cuttance, J.; Hooper, S. R.; [illegible]	2018	Journal of Pediatric Nursing	38	NA	57-61	10.1016/j.pedn.2017.10.008	Levine 2018	Irrelevant	Excluded by TI/AB review
1686	Integrated Care with Indigenous Populations: Considering the Role of Health Care Systems in Health Disparities	Lewis, M. E.; Myhra, L. L.	2018	Journal of Health Care for the Poor and Underserved	29	3	1083-1107	10.1353/hpu.2018.0081	Lewis 2018	Irrelevant	Excluded by TI/AB review
1687	Barriers to Providing Prehospital Care to Ischemic Stroke Patients: Predictors and [illegible]	Li, T.; Cushman, J. T.; Shah, M. N.; Kelly, A. G.; Rich, D. Q.; Jones, C. M. C.	2018	Prehospital and disaster medicine	33	5	501-507	10.1017/S1049023X18000766	Li 2018	Irrelevant	Excluded by TI/AB review
1688	Examining the role of sickle cell disease patients as teachers in the emergency department: Patient [illegible]	Liederman, Z.; Quartey, N. K.; Ward, R.; Papadakos, J.	2018	Blood	132	NA	NA	10.1182/blood-2018-99-112199	Liederman 2018	Irrelevant	Excluded by TI/AB review

S4 Table. All studies identified in the literature search

1689	Exploring the association between clinician burnout and adherence to back pain evaluation and treatment guidelines in the [illegible]	Linden, J.; Lerner, A.; Griffith, K.; Drainoni, M. L.; LaRochelle, M.; Liebschutz, J.; Wallace, E.; Weber, K.	2018	Academic Emergency Medicine	25	NA	S213-S214	10.1111/acem.13424	Linden 2018	Irrelevant	Excluded by TI/AB review
1690	A National Study of US Emergency Departments: Racial Disparities in Hospitalizations for Heart Failure	Lo, A. X.; Donnelly, J. P.; Durant, R. W.; Collins, S. P.; Levitan, E. B.; Storrow, A. B.; Bittner, V.	2018	American Journal of Preventive Medicine	55	5	S31-S39	10.1016/j.amepre.2018.05.020	Lo 2018	Irrelevant	Excluded by TI/AB review
1691	Triage disparities in pediatric patients that present to the emergency department with abdominal pain	Long, K.	2018	Pediatrics	141	1	NA	10.1542/peds.141.1-MeetingAbstract.368	Long 2018	Irrelevant	Excluded by TI/AB review
1692	Preoperative Nomograms Predict Patient-Specific Cervical Spine Surgery Clinical and Quality of Life Outcomes	Lubelski, D.; Alentado, V.; Nowacki, A. S.; Shriver, M.; Abdullah, K. G.; Steinmetz, M. P.; Benzel, E. C.; Mroz, T. E.	2018	Clinical Neurosurgery	83	1	104-113	10.1093/neuros/nyx343	Lubelski 2018	Irrelevant	Excluded by TI/AB review

S4 Table. All studies identified in the literature search

1693	Relating activity and participation levels to glycemic control, emergency department use, and [illegible]	Malcolm, M. P.; Atler, K. E.; Schmid, A. A.; Klinedinst, T. C.; Grimm, L. A.; Marchant, T. P.; Marchant, D. R.	2018	Clinical Diabetes	36	3	232-243	10.2337/cd17-0118	Malcolm 2018	Irrelevant	Excluded by TI/AB review
1694	Material Needs of Emergency Department Patients: A Systematic Review	Malecha, P. W.; Williams, J. H.; Kunzler, N. M.; Goldfrank, L. R.; Alter, H. J.; Doran, K. M.	2018	Academic Emergency Medicine	25	3	330-359	10.1111/acem.13370	Malecha 2018	Irrelevant	Excluded by TI/AB review
1695	Feasibility of Implementing an Erectile Dysfunction Screening Tool in a Primary Care Setting	Manaen, Mercy Atieno	2018	Feasibility Of Implementing An Erectile Dysfunction Screening Tool In A [illegible]	NA	NA	1-Jan	NA	Manaen 2018	Irrelevant	Excluded by TI/AB review
1696	Provider variation in antibiotic prescribing and outcomes of respiratory tract infections	Manne, M.; Deshpande, A.; Hu, B.; Patel, A.; Taksler, G. B.; Misra-Hebert, A. D.; Jolly, S. E.; Brateanu, A.; Bales, R. W.; Rothberg, M. B.	2018	Southern Medical Journal	111	4	235-242	10.14423/SMJ.0000000000000795	Manne 2018	Irrelevant	Excluded by TI/AB review

S4 Table. All studies identified in the literature search

1697	Outcomes of systematic anticoagulation management in pharmacist and nurse specialized clinics	Manzoor, B. S.; Bauman, J.; Shapiro, N. L.; Stamos, T.; Galanter, W.; Nutescu, E. A.	2018	JACCP Journal of the American College of Clinical Pharmacy	1	2	68-73	10.1002/jac5.1051	Manzoor 2018	Irrelevant	Excluded by TI/AB review
1698	The interactive effects of social support and physical functioning on HIV medical outcomes among African Americans whom inject drugs	Maragh-Bass, A. C.; Denison, J. A.; Thorpe, R. J.; Knowlton, A. R.	2018	Journal of Ethnicity in Substance Abuse	17	4	401-419	10.1080/15332640.2016.1264337	Maragh-Bass 2018	Irrelevant	Excluded by TI/AB review
1699	Co-morbid Non-communicable Diseases and Associated Health Service Use in African and Caribbean Immigrants with HIV	Masindi, K. I.; Jembere, N.; Kendall, C. E.; Burchell, A. N.; Bayoumi, A. M.; Loutfy, M.; Raboud, J.; Rourke, S. B.; Luyombya, H.; Antoniou, T.	2018	Journal of immigrant and minority health	20	3	536-545	10.1007/s10903-017-0681-6	Masindi 2018	Irrelevant	Excluded by TI/AB review

S4 Table. All studies identified in the literature search

1700	Oregon's Emphasis On Equity Shows Signs Of Early Success For Black And American Indian Medicaid Enrollees	McConnell, K. J.; Charlesworth, C. J.; Meath, T. H. A.; George, R. M.; Kim, H.	2018	Health Affairs	37	3	386-393	10.1377/hlthaff.2017.1282	McConnell 2018	Irrelevant	Excluded by TI/AB review
1701	Frequent emergency department users: A statewide comparison before and after [illegible]	McConville, S.; Raven, M. C.; Sabbagh, S. H.; Hsia, R. Y.	2018	Health Affairs	37	6	881-889	10.1377/hlthaff.2017.0784	McConville 2018	Irrelevant	Excluded by TI/AB review
1702	Intravenous continuous infusion vs. Oral immediate-release diltiazem for acute [illegible]	Means, K. N.; Gentry, A. E.; Nguyen, T. T.	2018	Western Journal of Emergency Medicine	19	2	417-422	10.5811/westjem.2017.10.33832	Means 2018	Irrelevant	Excluded by TI/AB review
1703	Racial disparities in refusal of stroke thrombolysis in Chicago	Mendelson, S. J.; Aggarwal, N. T.; Richards, C.; O'Neill, K.; Holl, J. L.; Prabhakaran, S.	2018	Neurology	90	5	e359-e364	10.1212/WNL.0000000000004905	Mendelson 2018	Irrelevant	Excluded by TI/AB review

S4 Table. All studies identified in the literature search

1704	The Social Determinants of Trauma: A Trauma Disparities Scoping Review and Framework	Mikhail, J. N.; Nemeth, L. S.; Mueller, M.; Pope, C.; NeSmith, E. G.	2018	Journal of Trauma Nursing	25	5	266-281	10.1097/JTN.0000000000000388	Mikhail 2018	Irrelevant	Excluded by TI/AB review
1705	Health care costs of case management for frequent users of the emergency department: Hospital and insurance perspectives	Moschetti, K.; Iglesias, K.; Baggio, S.; Velonaki, V.; Hugli, O.; Burnand, B.; Daeppen, J. B.; Wasserfallen, J. B.; Bodenmann, P.	2018	PLoS One	13	9	NA	10.1371/journal.pone.0199691	Moschetti 2018	Irrelevant	Excluded by TI/AB review
1706	Knowledge on birth preparedness and complication readiness among expecting couples in rural Tanzania: [illegible]	Moshi, F. V.; Ernest, A.; Fabian, F.; Kibusi, S. M.	2018	PLoS One	13	12	NA	10.1371/journal.pone.0209070	Moshi 2018	Irrelevant	Excluded by TI/AB review

S4 Table. All studies identified in the literature search

1707	Race Based Discrimination in Emergency Department Utilization; A Quantitative Study	Mostajabi, Mahdis	2018	Medical Journal of Bakirkoy	14	2	165-175	10.5350/BTDMJB.20170113093319	Mostajabi 2018	Irrelevant	Excluded by TI/AB review
1709	Short-term feasibility and impact assessments of an automated messaging system for hypertension selfmanagement	Muldoon, M. F.; Allen, M.; Einhorn, J.; Irizarry, T.; Rollman, B.; Lora, B.; Suffoletto, B.; Thomas, K.	2018	Psychosomatic Medicine	80	3	A138-A139	10.1097/PSY.0000000000000578	Muldoon 2018	Irrelevant	Excluded by TI/AB review

S4 Table. All studies identified in the literature search

1710	Caring for refugees and other migrants in new mexico: A simple handout for hospitalists	Mupfumira, T.; Barrett, E.; Ferdowsian, H.	2018	Journal of Hospital Medicine	13	4	NA	NA	Mupfumira 2018	Irrelevant	Excluded by TI/AB review
1711	Comprehensive community-based intervention and asthma outcomes in African American adolescents	Naar, S.; Ellis, D.; Cunningham, P.; Pennar, A. L.; Lam, P.; Brownstein, N. C.; Bruzzese, J. M.	2018	Pediatrics	142	4	NA	10.1542/peds.2017-3737	Naar 2018	Irrelevant	Excluded by TI/AB review
1712	Injured Children Receive Twice the Radiation Dose at Nonpediatric Trauma Centers Compared With Pediatric Trauma [illegible]	Nabaweesi, R.; Ramakrishnaiah, R. H.; Aitken, M. E.; Rettiganti, M. R.; Luo, C.; Maxson, R. T.; Glasier, C. M.; Kenney, P. J.; [illegible]	2018	Journal of the American College of Radiology	15	1	58-64	10.1016/j.jacr.2017.06.035	Nabaweesi 2018	Irrelevant	Excluded by TI/AB review

S4 Table. All studies identified in the literature search

1713	Causes and predictors of early hospital readmission in systemic lupus erythematosus	Nangit, A.; Lin, C.; Ishimori, M. L.; Spiegel, B. M. R.; Weisman, M. H.	2018	Journal of Rheumatolo gy	45	7	929-933	10.3899/jrh eum.170176	Nangit 2018	Irrelevant	Excluded by TI/AB review
1714	Improving care delivery in critical access hospitals: Evaluating the quality environment and the 'critical' role of telemedicine on access and costs	Natafgi, Nabil M.	2018	NA	78	NA	NA	NA	Natafgi 2018	Irrelevant	Excluded by TI/AB review
1715	Geriatric emergency department innovations: Impact on patient experience	Neher, H.; Vargas-Torres, C.; Rivera-Reyes, L.; Sze, J.; Hwang, U.	2018	Journal of the American Geriatrics Society	66	NA	S309-S310	10.1111/jgs. 15376	Neher 2018	Irrelevant	Excluded by TI/AB review

S4 Table. All studies identified in the literature search

1716	Geriatric emergency department innovations: Impact on patient experience	Neher, H. R.; Vargas-Torres, C.; Rivera-Reyes, L.; Sze, J.; Hwang, U.	2018	Academic Emergency Medicine	25	NA	S51-S52	10.1111/acem.13424	Neher 2018	Irrelevant	Excluded by TI/AB review
1717	Emergency department providers with higher empathy scores report greater ability to provide substance [illegible]	Nelson, R. W.; Monks, S.; Woolard, R. H.	2018	Academic Emergency Medicine	25	NA	S93	10.1111/acem.13424	Nelson 2018	Irrelevant	Excluded by TI/AB review
1718	Unmet needs for social support and its impact on health status and urgent healthcare use among older adults	O'Conor, R.; Benavente, J. Y.; Arvanitis, M.; Curtis, L. M.; Federman, A.; Wolf, M. S.	2018	Journal of General Internal Medicine	33	2	383-384	NA	O'Conor 2018	Irrelevant	Excluded by TI/AB review

S4 Table. All studies identified in the literature search

1719	Supporting asthma self-management behaviors in aging adults (SAMBA): A randomized clinical trial	O'Conor, R.; Mindlis, I.; Hauser, D.; Hoy-Rosas, J.; Lopez, R.; Lurio, J.; Chen, L.; Erblich, J.; Wolf, M. S.; Esky, J. W.; Federman, A.	2018	Journal of General Internal Medicine	33	2	350	NA	O'Conor 2018	Irrelevant	Excluded by TI/AB review
1720	Appropriateness of patients transferred with orthopedic injuries: experience of a level I trauma center	O‚ÄôConnell, R. S.; Haug, E. C.; Malasitt, P.; Mallu, S.; Satpathy, J.; Isaacs, J.; Mounasamy, V.	2018	European Journal of Orthopaedic Surgery and Traumatolo gy	28	4	551-554	10.1007/s00 590-018- 2134-x	O‚ÄôCon nell 2018	Irrelevant	Excluded by TI/AB review

S4 Table. All studies identified in the literature search

1721	Association between race and ethnicity and hip fracture outcomes in a universally insured population	Okike, K.; Chan, P. H.; Prentice, H. A.; Paxton, E. W.; Navarro, R. A.	2018	Journal of Bone and Joint Surgery - American Volume	100	13	1126-1131	10.2106/JBJS.17.01178	Okike 2018	Irrelevant	Excluded by TI/AB review
1722	Disparities in Emergency Department Wait Time Among Patients with Mental Health and [illegible]	Opoku, S. T.; Apenteng, B. A.; Akowuah, E. A.; Bhuyan, S.	2018	The journal of behavioral health services & research	45	2	204-218	10.1007/s11414-017-9565-8	Opoku 2018	Irrelevant	Excluded by TI/AB review
1723	Variation in Triple Aim Measures: Implications of Clinical Signatures in Family Medicine Residency Programs	Page, C. P.; Reid, A.; Drostin, C.; Newton, W. P.	2018	Journal of graduate medical education	10	5	548-552	10.4300/JGME-D-17-00470.1	Page 2018	Irrelevant	Excluded by TI/AB review

S4 Table. All studies identified in the literature search

1724	High inpatient utilization among Veterans Health Administration patients with substance-use disorders and co- [illegible]	Painter, J. M.; Malte, C. A.; Rubinsky, A. D.; Campellone, T. R.; Gilmore, A. K.; Baer, J. S.; Hawkins, E. J.	2018	American Journal of Drug and Alcohol Abuse	44	3	386-394	10.1080/00952990.2017.1381701	Painter 2018	Irrelevant	Excluded by TI/AB review
1725	Development of New Quality Measures for Hospital-Based Care of Suicidal Youth	Parast, L.; Bardach, N. S.; Burkhart, Q.; Richardson, L. P.; Murphy, J. M.; Gidengil, C. A.; Britto, M. T.; Elliott, M. N.; Mangione-Smith, R.	2018	Academic Pediatrics	18	3	248-255	10.1016/j.acap.2017.09.017	Parast 2018	Irrelevant	Excluded by TI/AB review
1727	Injuries of Women Surviving Intimate Partner Strangulation and Subsequent Emergency Health [illegible]	Patch, M.; Anderson, J. C.; Campbell, J. C.	2018	Journal of emergency nursing: JEN : official publication [illegible]	44	4	384-393	10.1016/j.jen.2017.12.001	Patch 2018	Irrelevant	Excluded by TI/AB review

S4 Table. All studies identified in the literature search

1728	Effect of a Lay Health Worker Intervention on Goals-of-Care Documentation and on Health Care Use, Costs, and Satisfaction among Patients with Cancer: A Randomized	Patel, M. I.; Sundaram, V.; Desai, M.; Periyakoil, V. S.; Kahn, J. S.; Bhattacharya, J.; Asch, S. M.; Milstein, A.; Bundorf, M. K.	2018	JAMA Oncology	4	10	1359-1366	10.1001/jamaoncol.2018.2446	Patel 2018	Irrelevant	Excluded by TI/AB review
1729	Utilizing grip strength prior to three-piece ipp placements as a predictor for post- [illegible]	Patel, R.; Jahn, H.; Canter, D.; Laborde, E.	2018	Journal of Urology	199	4	e425	NA	Patel 2018	Irrelevant	Excluded by TI/AB review
1730	What red wine triggers tell us about migraine and cluster headaches	Pergolizzi, J.; Coluzzi, F.; Varrasi, G.; Mariano, D.; Magnusson, P.; Colucci, R.; LeQuang, J. A.	2018	Postgraduate Medicine	130	NA	37-38	10.1080/00325481.2018.1512253	Pergolizzi 2018	Irrelevant	Excluded by TI/AB review

S4 Table. All studies identified in the literature search

1731	Predicting hospital return after discharge from the pediatric inpatient service	Pershad, J.; Cross, C.; Ajayi, O.; Harrell, C.; Jones, T.	2018	Pediatrics	141	1	NA	10.1542/peds.141.1-MeetingAbstract.426	Pershad 2018	Irrelevant	Excluded by TI/AB review
1732	Community Perspectives on Emergency Department Use and Care for American Indian [illegible]	Pickner, W. J.; Ziegler, K. M.; Hanson, J. D.; Payne, N. R.; Zook, H. G.; Kharbanda, A. B.; Weber, T. L.; [illegible]	2018	Journal of racial and ethnic health disparities	5	5	939-946	10.1007/s40615-017-0442-1	Pickner 2018	Irrelevant	Excluded by TI/AB review
1736	Determinants of Pediatric EMS Utilization in Children with High-Acuity Conditions	Qui√±ones, Casandra; Shah, Manish I.; Cruz, Andrea T.; Graf, Jeanine M.; Mondragon, Juan A.; Camp, Elizabeth A.; Reddy, Pooja; Sampayo, Esther M.	2018	Prehospital Emergency Care	22	6	676-690	10.1080/10903127.2018.1445330	Qui√±ones 2018	Irrelevant	Excluded by TI/AB review

S4 Table. All studies identified in the literature search

1737	Disparities in follow-up care for ballistic and non-ballistic long bone lower extremity fractures	Rafael Arceo, S.; Runner, R. P.; Huynh, T. D.; Gottschalk, M. B.; Schenker, M. L.; Moore, T. J.	2018	Injury	49	12	2193-2197	10.1016/j.injury.2018.09.053	RafaelArceo 2018	Irrelevant	Excluded by TI/AB review
1738	PEDIATRIC INGESTIONS OF CHRISTMAS PAST, PRESENT AND FUTURE, A REVIEW OF CHRISTMAS TRENDS	Reeves, P. T.; Pasman, E.; Krishnamurthy, J.; Nylund, C. M.	2018	Gastroenterology	154	6	S-1073	10.1016/S0016-5085(18)33583-2	Reeves 2018	Irrelevant	Excluded by TI/AB review
1739	Effect of Race on Outcomes Following Early Coronary Computed Tomographic [illegible]	Reinhardt, Samuel W.; Babatunde, Adefolakemi; Novak, Eric; Brown, David L.	2018	Ethnicity & Disease	28	4	517-524	10.18865/ed.28.4.517	Reinhardt 2018	Irrelevant	Excluded by TI/AB review

S4 Table. All studies identified in the literature search

1740	Mortality Associated With Emergency Department Boarding Exposure: Are There Differences Between Patients Admitted to ICU and Non-ICU Settings?	Reznek, Martin A.; Upatising, Benjavan; Kennedy, Samantha J.; Durham, Natassia T.; Forster, Richard M.; Michael, Sean S.	2018	Medical Care	56	5	436-440	10.1097/MLR.0000000000000000902	Reznek 2018	Irrelevant	Excluded by TI/AB review
1741	Standardizing the evaluation of nonaccidental trauma in a large pediatric	Riney, L. C.; Frey, T. M.; Fain, E. T.; Duma, E. M.; Bennett, B. L.; Kurowski, E. M.	2018	Pediatrics	141	1	NA	10.1542/peds.2017-1994	Riney 2018	Irrelevant	Excluded by TI/AB review
1743	Patient uncertainty during an emergency department visit and 30-day	Rising, K. L.; LaNoue, M.; Gerolamo, A. M.; Gentsch, A.; Doty, A. M. B.; Chuang, [illegible]	2018	Academic Emergency Medicine	25	NA	S103-S104	10.1111/acem.13424	Rising 2018	Irrelevant	Excluded by TI/AB review
1744	Development and Implementation of a Universal Suicide Risk Screening Program in a [illegible]	Roaten, K.; Johnson, C.; Genzel, R.; Khan, F.; North, C. S.	2018	Joint Commission Journal on Quality and Patient Safety	44	1	11-Apr	10.1016/j.jcjq.2017.07.006	Roaten 2018	Irrelevant	Excluded by TI/AB review

S4 Table. All studies identified in the literature search

1745	Characteristics of patients with airway disease who opt-in to the bedside medication [illegible]	Rogers, L.; Agarwal, P.; Yu, R.; Reich, D. L.; Mazumdar, M.	2018	American Journal of Respiratory and Critical Care [illegible]	197	MeetingAbstracts	NA	NA	Rogers 2018	Irrelevant	Excluded by TI/AB review
1746	Current Pediatric Emergency Department Innovative Programs to Improve the Care of Psychiatric [illegible]	Roman, S. B.; Matthews-Wilson, A.; Dickinson, P.; Chenard, D.; Rogers, S. C.	2018	Child and Adolescent Psychiatric Clinics of North America	27	3	441-454	10.1016/j.chc.2018.02.004	Roman 2018	Irrelevant	Excluded by TI/AB review

S4 Table. All studies identified in the literature search

1747	Improving Quality of Care in Hospitals for Victims of Elder Mistreatment: Development of the Vulnerable Elder Protection Team	Rosen, T.; Mehta-Naik, N.; Elman, A.; Mulcare, M. R.; Stern, M. E.; Clark, S.; Sharma, R.; LoFaso, V. M.; Breckman, R.; Lachs, M.; Needell, N.	2018	Joint Commission Journal on Quality and Patient Safety	44	3	164-171	10.1016/j.jcjq.2017.08.010	Rosen 2018	Irrelevant	Excluded by TI/AB review

S4 Table. All studies identified in the literature search

1748	Risk Factors Associated with Emergency Department Return Visits Following Trauma System Discharge	Ruttan, T.; Lawson, K. A.; Piper, K.; Wilkinson, M.	2018	Pediatric Emergency Care	34	3	202-207	10.1097/PEC.0000000000001182	Ruttan 2018	Irrelevant	Excluded by TI/AB review
1749	Interactions with the healthcare system influence advance care planning activities: Results from a [illegible]	Sable-Smith, A.; Arnett, K. R.; Nowels, M. A.; Colborn, K.; Lum, H. D.; Nowels, D.	2018	Family Practice	35	3	307-311	10.1093/fampra/cmx113	Sable-Smith 2018	Irrelevant	Excluded by TI/AB review
1750	A 6-month prospective randomized controlled trial of remotely delivered group format epilepsy self-management versus waitlist control for high-risk people with epilepsy	Sajatovic, M.; Colon-Zimmermann, K.; Kahriman, M.; Fuentes-Casiano, E.; Liu, H.; Tatsuoka, C.; Cassidy, K. A.; Lhatoo, S.; Einstadter, D.; Chen, P.	2018	Epilepsia	59	9	1684-1695	10.1111/epi.14527	Sajatovic 2018	Irrelevant	Excluded by TI/AB review

S4 Table. All studies identified in the literature search

1751	"Sometimes You Feel Like the Freak Show": A¬†Qualitative Assessment of Emergency Care Experiences Among [illegible]	Samuels, E. A.; Tape, C.; Garber, N.; Bowman, S.; Choo, E. K.	2018	Ann Emerg Med	71	2	170-182.e1	10.1016/j.annemergmed.2017.05.002	Samuels 2018	Irrelevant	Excluded by TI/AB review
1756	Improving emergency department patient experience through implementation of an informational brochure	Sangal, R. B.; Orloski, C.; Shofer, F. S.; Mills, A.	2018	Academic Emergency Medicine	25	NA	S68-S69	10.1111/acem.13424	Sangal 2018	Irrelevant	Excluded by TI/AB review
1757	Disparities in care among patients presenting to the emergency department for nephrolithiasis	Schoenfeld, D.; Mohn, L.; Agalliu, I.; Stern, J.	2018	Journal of Clinical and Translational Science	NA	NA	69	10.1017/cts.2018.250	Schoenfeld 2018	Irrelevant	Excluded by TI/AB review

S4 Table. All studies identified in the literature search

1759	Healthcare disparities amongst vulnerable populations of Arabs and Jews in Israel	Shadmi, E.	2018	Isr J Health Policy Res	7	1	26	10.1186/s13584-018-0226-z	Shadmi 2018	Irrelevant	Excluded by TI/AB review
1761	Effect of patient and nurse ethnicity on emergency department analgesia for children with appendicitis in israeli government hospitals	Shavit, I.; Jacob, R.; Friedman, N.; Capua, T.; Klein, A.; Chistyakov, I.; Moldaver, I.; Krupik, D.; Munchak, I.; Abozaid, S.; Rimon, A.; Meirson, G.; Leiba, R.; Cohen, D. M[illegible]	2018	European Journal of Pain (United Kingdom)	22	10	1711-1717	10.1002/ejp.1257	Shavit 2018	Irrelevant	Excluded by TI/AB review

S4 Table. All studies identified in the literature search

1762	Gender differences in the comprehension of care plans in an emergency department setting	Shiber, S.; Zuker-Herman, R.; Drescher, M. J.; Glezerman, M.	2018	Israel Journal of Health Policy Research	7	1	NA	10.1186/s13584-018-0245-9	Shiber 2018	Irrelevant	Excluded by TI/AB review
1763	Receipt of cardiac screening does not influence 1-year post-cerebrovascular event mortality	Sico, J. J.; Baye, F.; Myers, L. J.; Concato, J.; Ferguson, J.; Cheng, E. M.; Jadbabaie, F.; Yu, Z.; Arling, G.; Zillich, A. J.; Reeves, M. J.; Williams, L. [illegible]	2018	Neurology: Clinical Practice	8	3	192-200	10.1212/CPJ.0000000000000465	Sico 2018	Irrelevant	Excluded by TI/AB review
1764	High users of healthcare: Strategies to improve care, reduce costs	Siekman, N.; Hilger, R.	2018	Cleveland Clinic journal of medicine	85	1	25-31	10.3949/ccjm.85a.16082	Siekman 2018	Irrelevant	Excluded by TI/AB review
1765	The magic clinic: Results from a 2-year pilot program providing interdisciplinary geriatric consultation	Simone, M. J.; Padgett, L.	2018	Journal of the American Geriatrics Society	66	NA	S41	10.1111/jgs.15376	Simone 2018	Irrelevant	Excluded by TI/AB review

S4 Table. All studies identified in the literature search

1766	Delivery Outcomes, Emergency Room Visits, and Psychological Aspects of Gestational [illegible]	Singh, H.; Soyoltulga, K.; Fong, T.; Billimek, J.	2018	The Diabetes educator	44	5	465-474	10.1177/0145721718795589	Singh 2018	Irrelevant	Excluded by TI/AB review
1768	Injury resulting from targeted violence: An emergency	Sivarajasingam, V.; Read, S.; Svobodova, M.; Wight, L.; Shepherd, J.	2018	Criminal Behaviour and Mental Health	28	3	295-308	10.1002/cbm.2066	Sivarajasingam 2018	Irrelevant	Excluded by TI/AB review
1769	Predictors of emergency department attendance by people with [illegible]	Sleeman, K. E.; Perera, G.; Stewart, R.; Higginson, I. J.	2018	Alzheimer's and Dementia	14	1	20-27	10.1016/j.jalz.2017.06.2267	Sleeman 2018	Irrelevant	Excluded by TI/AB review
1770	A randomized controlled trial comparing two vaso-occlusive episode (VOE) protocols in sickle cell disease (SCD)	Tanabe, P.; Silva, S.; Bosworth, H. B.; Crawford, R.; Paice, J. A.; Richardson, L. D.; Miller, C. N.; Glassberg, J.	2018	American Journal of Hematology	93	2	159-168	10.1002/ajh.24948	Tanabe 2018	Irrelevant	Excluded by TI/AB review

S4 Table. All studies identified in the literature search

1771	Health literacy and patient outcomes in chronic kidney disease: a systematic review	Taylor, D. M.; Fraser, S.; Dudley, C.; Oniscu, G. C.; Tomson, C.; Ravanan, R.; Roderick, P.	2018	Nephrology, dialysis, transplantation : official publication of the European Dialysis and Transplant Association - European Renal	33	9	1545-1558	10.1093/ndt/gfx293	Taylor 2018	Irrelevant	Excluded by TI/AB review
1773	Racial differences in predictors of timely antibiotics and mortality in suspected septic shock	Taylor, S.; Karvetski, C.; Roohoolahi, A.; Templin, M.; Taylor, B.	2018	Critical Care Medicine	46	NA	737	10.1097/01.ccm.0000529508.50913.9f	Taylor 2018	Irrelevant	Excluded by TI/AB review
1774	Racism by way of education: The politics of privilege and oppression	Thorne, Daryl H.; Anderson, Sharon K.; Middleton, Valerie A.	2018	Explorations in diversity: Examining the complexities [illegible]	NA	NA	276-285	NA	Thorne 2018	Irrelevant	Excluded by TI/AB review
1775	Postprandial insulin initiation-evaluation of	Tseng, C. L.; Soroka, O.; Pogach, L.	2018	Diabetes	67	NA	A108	NA	Tseng 2018	Irrelevant	Excluded by TI/AB review

S4 Table. All studies identified in the literature search

1776	Barriers to primary and emergency healthcare for trans adults	Vermeir, E.; Jackson, L. A.; Marshall, E. G.	2018	Culture Health & Sexuality	20	2	232-246	10.1080/13691058.2017.1338757	Vermeir 2018	Irrelevant	Excluded by TI/AB review
1777	Impact of community health workers in management of heart failure: A pilot study	Vohra, A. S.; Chua, R.; Alcain, C. F.; Coplan, M. J.; Spencer, K. T.; Liao, J. K.; Tabit, C. E.	2018	Circulation: Cardiovascular Quality and Outcomes	11	NA	NA	NA	Vohra 2018	Irrelevant	Excluded by TI/AB review
1778	Fewer immigrants have preventable ED visits in the United States	Wang, Y.; Wilson, F. A.; Stimpson, J. P.; Wang, H.; Palm, D. W.; Chen, B.; Chen, L. W.	2018	Am J Emerg Med	36	3	352-358	10.1016/j.ajem.2017.08.018	Wang 2018	Irrelevant	Excluded by TI/AB review

S4 Table. All studies identified in the literature search

1780	Are inner-city children with asthma receiving specialty care as recommended in national asthma guidelines?	Warman, K. L.; Silver, E. J.	2018	Journal of Asthma	55	5	517-524	10.1080/02770903.2017.1350966	Warman 2018	Irrelevant	Excluded by TI/AB review
1781	Conference proceedings of the 4th masterclass psychiatry: Transcultural	Werneke, Ursula; Werneke, Ursula; Bhugra, Dinesh; √ñhlund, Louise; Lieber, Ingrid; Bhui,	2018	Nordic Journal of Psychiatry	NA	NA	NA	10.1080/08039488.2018.1481525	Werneke 2018	Irrelevant	Excluded by TI/AB review
1782	Patient preference for pain medication in the emergency department is associated with	Whiteside, L. K.; Goldstick, J.; Dora-Laskey, A.; Thomas, L.; Walton, M.; Cunningham, R.; Bohnert, A. S. B.	2018	Western Journal of Emergency Medicine	19	4	722-730	10.5811/westjem.2018.4.37019	Whiteside 2018	Irrelevant	Excluded by TI/AB review
1783	Emergency department utilization by native American children	Zook, H. G.; Kharbanda, A. B.; Puumala, S. E.; Burgess, K. A.; Pickner, W.; Payne,	2018	Pediatric Emergency Care	34	11	802-809	10.1097/PEC.0000000000001289	Zook 2018	Irrelevant	Excluded by TI/AB review

S4 Table. All studies identified in the literature search

1784	Training to Improve Communication Quality: An Efficient Interdisciplinary [illegible]	Aaronson, E. L.; White, B. A.; Black, L.; Brown, D. F.; Benzer, T.; Castagna, A.; Raja, A. S.; Sonis, J.; Mort, E.	2019	American journal of medical quality : the official journal of [illegible]	34	3	260-265	10.1177/10628606187 99936	Aaronson 2019	Irrelevant	Excluded by TI/AB review
1786	Health Care Utilization of Opioid Overdose Decedents with No Opioid Analgesic Prescription History	Abbasi, A. B.; Salisbury-Afshar, E.; Jovanov, D.; Berberet, C.; Arunkumar, P.; Aks, S. E.; Layden, J. E.; Pho, M. T.	2019	Journal of urban health : bulletin of the New York Academy of Medicine	96	1	38-48	10.1007/s11 524-018-00329-x	Abbasi 2019	Irrelevant	Excluded by TI/AB review
1787	Higher odds of irritable bowel syndrome among hospitalized patients using cannabis: A propensity-matched analysis	Adejumo, A. C.; Ajayi, T. O.; Adegbala, O. M.; Bukong, T. N.	2019	European Journal of Gastroenterology and Hepatology	31	7	756-765	10.1097/MEG.000000000000138 2	Adejumo 2019	Irrelevant	Excluded by TI/AB review

S4 Table. All studies identified in the literature search

1788	Social factors in frequent callers: a description of isolation, poverty and quality of life in those calling emergency medical services frequently	Agarwal, G.; Lee, J.; McLeod, B.; Mahmuda, S.; Howard, M.; Cockrell, K.; Angeles, R.	2019	BMC Public Health	19	1	684	10.1186/s12889-019-6964-1	Agarwal 2019	Irrelevant	Excluded by TI/AB review
1789	Rationale and methods of an Evaluation of the Effectiveness of the Community Paramedicine at [illegible]	Agarwal, G.; Pirrie, M.; McLeod, B.; Angeles, R.; Tavares, W.; Marzanek, F.; Thabane, L.	2019	Trials	20	1	NA	10.1186/s13063-018-3107-4	Agarwal 2019	Irrelevant	Excluded by TI/AB review
1790	The influence of gender and underrepresented minority status on medical student ranking of residency programs	Agawu, Atu; Fahl, Corrinne; Alexis, Dominique; Diaz, Tomas; Harris, Diana; Harris, Mary C.; Aysola, Jaya; Cronholm, Peter F.; Higginbotham, Eve J.	2019	Journal of the National Medical Association	111	6	665-673	10.1016/j.jnma.2019.09.002	Agawu 2019	Irrelevant	Excluded by TI/AB review

S4 Table. All studies identified in the literature search

1791	Osteoporosis management outcomes in a southern california county health system	Ahn, L.; Thompson, J.; Collado, N.; Yu, M.; Lafian, A.; Downey, C.; Torralba, K.	2019	Arthritis and Rheumatolo gy	71	NA	429-430	10.1002/art. 41108	Ahn 2019	Irrelevant	Excluded by TI/AB review
1792	Predictors of alcohol-related hospital visits after propensity score matching for receipt of addictions [illegible]	Alapati, D.; Meanley, S.; Korthuis, P. T.; Bart, G.	2019	Journal of Addiction Medicine	13	3	E15	10.1097/A DM.000000 000000054 8	Alapati 2019	Irrelevant	Excluded by TI/AB review
1793	Receipt of Addiction Treatment Following Opioid-Related Overdose Among Medicaid-Enrolled Youth	Alinsky, R.; Zima, B.; Bagley, S.; Rodean, J.; Matson, P.; Adger, H.; Hadland, S.	2019	Journal of Adolescent Health	64	2	S17	10.1016/j.ja dohealth.20 18.10.046	Alinsky 2019	Irrelevant	Excluded by TI/AB review
1795	Longitudinal patterns of mexican and puerto rican children's asthma [illegible]	Arcoleo, K. J.; McGovern, C.; Kaur, K.; Halterman, J. S.; Mammen, J.; Crean, H.; Rastogi, D.; [illegible]	2019	Annals of the American Thoracic Society	16	6	715-723	10.1513/An nalsATS.20 1807-462OC	Arcoleo 2019	Irrelevant	Excluded by TI/AB review

S4 Table. All studies identified in the literature search

1797	Association of peak expiratory flow with forced expiratory volume during acute childhood asthma [illegible]	Arnold, D. H.; Burger, C. E.; Daly, C. H.; Kumar, V.	2019	Academic Emergency Medicine	26	NA	S67	10.1111/acem.13756	Arnold 2019	Irrelevant	Excluded by TI/AB review
1798	268 Shifting Away from Racial Bias	Ashour, E.; Lareaux, D.; Slovis, B.; Joshi, A. U.	2019	Annals of Emergency Medicine	74	4	S104-S105	10.1016/j.annemergmed.2019.08.226	Ashour 2019	Irrelevant	Excluded by TI/AB review
1799	Developmentofa novel measureof timelyaccess to primary care services	Augustine, M. R.; Batten, A. J.; Nelson, K. M.; Kaboli, P. J.	2019	Journal of General Internal Medicine	34	2	S184	10.1007/11606.1525-1497	Augustine 2019	Irrelevant	Excluded by TI/AB review
1801	Effect of Intensive Interdisciplinary Transitional Care for High-Need, High-Cost Patients on Quality, Outcomes, and [illegible]	Bailey, J. E.; Surbhi, S.; Wan, J. Y.; Munshi, K. D.; Waters, T. M.; Binkley, B. L.; Ugwueke, M. O.; Graetz, I.	2019	Journal of General Internal Medicine	34	9	1815-1824	10.1007/s11606-019-05082-8	Bailey 2019	Irrelevant	Excluded by TI/AB review

S4 Table. All studies identified in the literature search

1802	Variation of Follow-Up Rate After Psychiatric Hospitalization of Medicare Beneficiaries by Hospital Characteristics and Social Determinants of Health	Benjenk, I.; Chen, J.	2019	American Journal of Geriatric Psychiatry	27	2	138-148	10.1016/j.jagp.2018.08.006	Benjenk 2019	Irrelevant	Excluded by TI/AB review
1803	KAPOSI SARCOMA AND IRIS: IN THE ERA OF HAART	Berhane, F.; Kodadhala, V.; gebrekidan, Y.; Folawewo, K.; Thomas, A.; Poddar, V.	2019	Chest	156	4	A561-A562	10.1016/j.chest.2019.08.560	Berhane 2019	Irrelevant	Excluded by TI/AB review
1804	A critical introduction to psychology	Beshara, Robert K.	2019	NA	NA	NA	NA	NA	Beshara 2019	Irrelevant	Excluded by TI/AB review
1805	Diabetic hypoglycaemia during Ramadan fasting: A trans-national [illegible]	Beshyah, S. A.; Hassanein, M.; Ahmedani, M. Y.; Shaikh, S.; Ba-Essa, E. M.; Megallaa, M. [illegible]	2019	Diabetes Research and Clinical Practice	150	NA	315-321	10.1016/j.diabres.2019.01.039	Beshyah 2019	Irrelevant	Excluded by TI/AB review

S4 Table. All studies identified in the literature search

1806	Outcomes of Children of Parents in Medication-Assisted	Betcher, H. K.; Vande Voort, J. L.; Croarkin, P. E.; Gandhi, K. D.; Shekunov, J.;	2019	Primary Care Companion to the Journal of	21	4	NA	10.4088/PCC.19m02474	Betcher 2019	Irrelevant	Excluded by TI/AB review
1807	Decision-making in ambulance service non-conveyance-the DMASC survey	Black, S.; Frampton, I.	2019	Emergency Medicine Journal	36	1	E10	10.1136/emermed-2019-999.25	Black 2019	Irrelevant	Excluded by TI/AB review
1808	Critical essays on the new moral imperative for supporting marginalized students in PK-20 education	Blankenship, Rebecca J.	2019	Advances in early childhood and K-12 education (AECKE); ISSN: 2329-5929 (Print), 2329-5937 (Electronic)	NA	NA	NA	10.4018/978-1-5225-7787-4	Blankenship 2019	Irrelevant	Excluded by TI/AB review

S4 Table. All studies identified in the literature search

1809	Nursing updates: A solution for improving patient satisfaction?	Blutinger, E.; Sangal, R.; Shofer, F.; Kurkowski, J.; Westover, J.; Mamtani, M.	2019	Academic Emergency Medicine	26	NA	S302	10.1111/acem.13756	Blutinger 2019	Irrelevant	Excluded by TI/AB review
1810	Disparities in emergency department wait time and length of stay: A national perspective	Bove, T.; Thode, H. C.; Singer, A. J.; Bright, L.	2019	Academic Emergency Medicine	26	NA	S17	10.1111/acem.13756	Bove 2019	Irrelevant	Excluded by TI/AB review
1811	Effect of Patient Care Coordination on Hospital Encounters and Related Costs	Breckenridge, Ellen D.; Kite, Bobbie; Wells, Rebecca; Sunbury, Tenaya M.	2019	Population Health Management	22	5	406-414	10.1089/pop.2018.0176	Breckenridge 2019	Irrelevant	Excluded by TI/AB review

S4 Table. All studies identified in the literature search

1812	Legal Issues with Sexual and Gender Minority Patients in the Emergency Department	Brous, Edie	2019	Journal of Legal Nurse Consulting	30	2	30-37	NA	Brous 2019	Irrelevant	Excluded by TI/AB review
1813	Design and patient characteristics of the randomized controlled trial TExT- [illegible]	Burner, E.; Mercado, J.; Hernandez-Saenz, A.; Peters, A.; Baezconde-Garbanati, L.; Arora, [illegible]	2019	Contemporary Clinical Trials	80	NA	8-Jan	10.1016/j.cct.2019.03.003	Burner 2019	Irrelevant	Excluded by TI/AB review
1815	Comparative effectiveness trial of an obesity prevention intervention in EFNEP and SNAP-ED: [illegible]	Buscemi, J.; Odoms-Young, A.; Stolley, M. R.; Schiffer, L.; Blumstein, L.; Clark, M. H.; Berbaum, M. L.; McCaffrey, J.; Braunschweig, C.; [illegible]	2019	Nutrients	11	5	NA	10.3390/nu11051012	Buscemi 2019	Irrelevant	Excluded by TI/AB review
1816	Drinking and Intimate Partner Violence Severity Levels Among U.S. Ethnic Groups in an Urban Emergency Department	Caetano, R.; Cunradi, C. B.; Alter, H. J.; Mair, C.; Yau, R. K.	2019	Academic Emergency Medicine	26	8	897-907	10.1111/acem.13706	Caetano 2019	Irrelevant	Excluded by TI/AB review

S4 Table. All studies identified in the literature search

1817	IS OUR MED-REC A MED-WRECK? QUALITY IMPROVEMENT STUDY	Caputi, R.; Zuluaga, J. J.; Mohan, J.	2019	Chest	156	4	A1048	10.1016/j.chest.2019.08.968	Caputi 2019	Irrelevant	Excluded by TI/AB review
1818	Social isolation as a predictor of post-emergency department visit follow-up by older adults	Cayenne, N.; DuGoff, E. H.; Jacobsohn, G. C.; Mi, R. Z.; Cushman, J. T.; Jones, C.; Kind, A. J. H.; Lohmeier, M. T.; [illegible]	2019	Academic Emergency Medicine	26	NA	S126	10.1111/acem.13756	Cayenne 2019	Irrelevant	Excluded by TI/AB review
1819	Scared to go home? psychological distress and patient preferences for transient ischemic attack and minor stroke care	Chang, B.; Edmondson, D.; Elkind, M. S.; Willey, J.; Kronish, I.	2019	Academic Emergency Medicine	26	NA	S187	10.1111/acem.13756	Chang 2019	Irrelevant	Excluded by TI/AB review

S4 Table. All studies identified in the literature search

1820	The association of threat perception and patient treatment preferences in acute stroke	Chang, B. P.; Edmondson, D.; Birk, J. L.; Meli, L.; Sumner, J. A.; Derby, L.; Cornelius, T.; Kronish, I.	2019	Psychosomatic Medicine	81	4	A179-A180	10.1097/PSY.0000000000000699	Chang 2019	Irrelevant	Excluded by TI/AB review
1821	Patient preferences for inpatient vs outpatient management of tia and minor stroke and its association with psychological stress	Chang, B. P.; Willey, J.; Edmondson, D.; Miller, E.; Sanchez, G. J.; Elkind, M. S.; Kronish, I.	2019	Stroke	50	NA	NA	10.1161/str.50.suppl_1.TP301	Chang 2019	Irrelevant	Excluded by TI/AB review
1822	Inclusion of Nursing Homes and Long-term Residents in Medicare ACOs	Chang, C. H.; Mainor, A.; Raymond, S.; Peck, K.; Colla, C.; Bynum, J.	2019	Medical Care	57	12	990-995	10.1097/MLR.0000000000001223	Chang 2019	Irrelevant	Excluded by TI/AB review

S4 Table. All studies identified in the literature search

1823	Pulled away: the experience of bilingual nurses as ad hoc interpreters in the emergency department	Chang, H.; Hutchinson, C.; Gullick, J.	2019	Ethnicity & health	NA	NA	20-Jan	10.1080/13557858.2019.1613518	Chang 2019	Irrelevant	Excluded by TI/AB review
1824	Less is more: Reversal of valvular disease using patient-centered care without device therapy	Chen, L.; Guo, U.; Mehta, P.	2019	Journal of General Internal Medicine	34	2	S572-S573	10.1007/11606.1525-1497	Chen 2019	Irrelevant	Excluded by TI/AB review
1825	Investigating the causal effect of socioeconomic status on quality of care under a universal health [illegible]	Cho, H. E.; Wang, L.; Chen, J. S.; Liu, M. C.; Kuo, C. F.; Chung, K. C.	2019	BMC Health Services Research	19	1	NA	10.1186/s12913-019-4793-7	Cho 2019	Irrelevant	Excluded by TI/AB review

S4 Table. All studies identified in the literature search

1826	Patient factor disparities in imaging follow-up rates after incidental abdominal findings	Cho, J. K.; Zafar, H. M.; Lalevic, D.; Cook, T. S.	2019	American Journal of Roentgenology	212	3	589-595	10.2214/AJR.18.20083	Cho 2019	Irrelevant	Excluded by TI/AB review

S4 Table. All studies identified in the literature search

1827	An update to the local prevalence of erectile dysfunction post transrectal ultrasound guided biopsy of the prostate: The experience of a single institution	Chong, W.; Yeow, Y.; Wei Ronny, T. B.	2019	BJU International	123	NA	39	NA	Chong 2019	Irrelevant	Excluded by TI/AB review
1828	Infants Exposed To Homelessness: Health, Health Care Use, And Health Spending From Birth To Age Six	Clark, R. E.; Weinreb, L.; Flahive, J. M.; Seifert, R. W.	2019	Health affairs (Project Hope)	38	5	721-728	10.1377/hlthaff.2019.00090	Clark 2019	Irrelevant	Excluded by TI/AB review
1829	Effects of a standardized emergency department protocol on after- [illegible]	Clark, R. M.; Weingardt, D.; Goff, J. M.; Ketteler, E. R.	2019	Journal of Vascular Surgery: Venous and Lymphatic [illegible]	7	4	501-506	10.1016/j.jvsv.2018.11.010	Clark 2019	Irrelevant	Excluded by TI/AB review

S4 Table. All studies identified in the literature search

1830	effect of an interprofessional pain service on nonmalignant pain control	Coffey, Cory P.; Ulbrich, Timothy R.; Baughman, Kristin; Awad, Magdi H.	2019	American Journal of Health-System Pharmacy	76	NA	S49-S54	10.1093/ajhp/zxy084	Coffey 2019	Irrelevant	Excluded by TI/AB review
1831	Barriers Latino Patients Face in the Pediatric Emergency Department	Connors, Kaleigh Mary	2019	Pediatric nursing	45	1	37-46	NA	Connors 2019	Irrelevant	Excluded by TI/AB review
1832	Bringing close others to the emergency department for an acute coronary event is associated with increased [illegible]	Cornelius, T.; Meli, L.; Thorson, K. R.; Chang, B. P.; Edmondson, D.; West, T. V.	2019	General Hospital Psychiatry	56	NA	42-49	10.1016/j.genhosppsych.2018.12.002	Cornelius 2019	Irrelevant	Excluded by TI/AB review

S4 Table. All studies identified in the literature search

1833	Validity of the self-reported domains of subjective extent of nonadherence (Dose-nonadherence) scale in comparisonwith electronically [illegible]	Cornelius, T.; Voils, C. I.; Umland, R. C.; Kronish, I. M.	2019	Patient Preference and Adherence	13	NA	1677-1684	10.2147/PPA.S225460	Cornelius 2019	Irrelevant	Excluded by TI/AB review
1834	Social determinants of health and emergency department use among children [illegible]	Cortright, L.; Tumin, D.; Leonard, S.	2019	Pediatric Blood and Cancer	66	NA	S262-S263	10.1002/pbc.27713	Cortright 2019	Irrelevant	Excluded by TI/AB review
1835	Acute care model that reduces oncology-related unplanned hospitalizations to [illegible]	Coyle, Y. M.; Ogola, G. O.; MacLachlan, C. R.; Hinshelwood, M. M.; Fleming, N. S.	2019	Journal of Cancer Policy	21	NA	NA	10.1016/j.jcpo.2019.100193	Coyle 2019	Irrelevant	Excluded by TI/AB review
1836	The effects of a morphine shortage on emergency department pain control and patient satisfaction	Coyne, C. J.; Kettler, E. B.; Brennan, J. J.	2019	Academic Emergency Medicine	26	NA	S223	10.1111/acem.13756	Coyne 2019	Irrelevant	Excluded by TI/AB review

S4 Table. All studies identified in the literature search

1837	Review article: Clinical characteristics and outcomes of patient presentations to the emergency department via police: A scoping review	Crilly, J.; Johnston, A. N. B.; Wallis, M.; Polong-Brown, J.; Heffernan, E.; Fitzgerald, G.; Young, J. T.; Kinner, S.	2019	EMA - Emergency Medicine Australasia	31	4	506-515	10.1111/1742-6723.13300	Crilly 2019	Irrelevant	Excluded by TI/AB review
1838	Examining emergency department inequities: Do they exist?	Curtis, E.; Paine, S. J.; Jiang, Y.; Jones, P.; Tomash, I.; Raumati, I.; Reid, P.	2019	Emerg Med Australas	31	3	444-450	10.1111/1742-6723.13315	Curtis 2019	Irrelevant	Excluded by TI/AB review
1842	Socioeconomic position and use of healthcare in the last year of life: A systematic review and meta-analysis	Davies, J. M.; Sleeman, K. E.; Leniz, J.; Wilson, R.; Higginson, I. J.; Verne, J.; Maddocks, M.; Murtagh, F. E. [illegible]	2019	PLoS Medicine	16	4	NA	10.1371/journal.pmed.1002782	Davies 2019	Irrelevant	Excluded by TI/AB review

S4 Table. All studies identified in the literature search

1843	Real-world impact of a novel digital medicine program on adherence and utilization in pediatric pre-and post-transplant patients	Desai, D.; Sullivan, S.; Triplett, K. N.; Sacks, C.; Tran, M.	2019	Pediatric Transplantation	23	NA	NA	10.1111/petr.13447	Desai 2019	Irrelevant	Excluded by TI/AB review
1844	Gender Preferences of Patients When Selecting Orthopaedic Providers	Dineen, H. A.; Patterson, J. M. M.; Eskildsen, S. M.; Gan, Z. S.; Li, Q.; Patterson, B. C.; Draeger, R. W.	2019	Iowa Orthop J	39	1	203-210	NA	Dineen 2019	Irrelevant	Excluded by TI/AB review
1846	A genealogy of lesbian feminisms in New Zealand: Some implications for young lesbian health and wellbeing	Du Preez, Katie Palmer; Payne, Deborah; Giddings, Lynne	2019	Aporia	11	2	15-26	NA	DuPreez 2019	Irrelevant	Excluded by TI/AB review
1847	Identification of racial inequities in access to specialized	Eberly, L. A.; Richterman, A.; Beckett, A. G.; Wispelwey, B.;	2019	Circulation: Heart Failure	12	11	NA	10.1161/CIRCHEARTFAILURE.119.006214	Eberly 2019	Irrelevant	Excluded by TI/AB review

S4 Table. All studies identified in the literature search

1849	Management of acute retrobulbar haemorrhage: A survey of non-ophthalmic emergency [illegible]	Edmunds, M. R.; Haridas, A. S.; Morris, D. S.; Jamalapuram, K.	2019	Emergency Medicine Journal	36	4	245-247	10.1136/emermed-2018-207937	Edmunds 2019	Irrelevant	Excluded by TI/AB review
1850	Disparities in Quality of Primary Care by Resident and Staff Physicians: Is There a Conflict [illegible]	Essien, U. R.; He, W.; Ray, A.; Chang, Y.; Abraham, J. R.; Singer, D. E.; Atlas, S. J.	2019	J Gen Intern Med	34	7	1184-1191	10.1007/s11606-019-04960-5	Essien 2019	Irrelevant	Excluded by TI/AB review
1853	Racial and ethnic differences in the medical treatment of opioid use disorders within the VA healthcare system following [illegible]	Essien, U. R.; Sileanu, F.; Liebschutz, J. M.; Good, C.; Zhao, X.; Mor, M. K.; Fine, M. J.; Gellad, W. F.	2019	Journal of General Internal Medicine	34	2	S338	10.1007/11606.1525-1497	Essien 2019	Irrelevant	Excluded by TI/AB review
1854	Avoidable acute care use for vaccine-preventable illnesses among medicaid beneficiaries with lupus: [illegible]	Feldman, C.; Xu, C.; Costenbader, K.	2019	Arthritis and Rheumatology	71	NA	1657-1659	10.1002/art.41108	Feldman 2019	Irrelevant	Excluded by TI/AB review

S4 Table. All studies identified in the literature search

1855	Implementation of a local safety standard for invasive procedures (LocSSIP) [illegible]	Feneley, A.; Struthers, H.; Petrides, P.; Verma, S.; Black, S.	2019	Anaesthesia	74	NA	55	10.1111/anae.14740	Feneley 2019	Irrelevant	Excluded by TI/AB review
1856	Trauma-Informed Care for Violently Injured Patients in the Emergency Department	Fischer, K. R.; Bakes, K. M.; Corbin, T. J.; Fein, J. A.; Harris, E. J.; James, T. L.; Melzer-Lange, M. D.	2019	Annals of Emergency Medicine	73	2	193-202	10.1016/j.annemergmed.2018.10.018	Fischer 2019	Irrelevant	Excluded by TI/AB review
1857	Racial disparities in asthma-related health care use in the National Heart, Lung, and Blood Institute's Severe Asthma Research Program	Fitzpatrick, A. M.; Gillespie, S. E.; Mauger, D. T.; Phillips, B. R.; Bleecker, E. R.; Israel, E.; Meyers, D. A.; Moore, W. C.; Sorkness, R. L.; Wenzel, S. E.; [illegible]	2019	Journal of Allergy and Clinical Immunology	143	6	2052-2061	10.1016/j.jaci.2018.11.022	Fitzpatrick 2019	Irrelevant	Excluded by TI/AB review

S4 Table. All studies identified in the literature search

1858	The Use of an Electronic Health Record Patient Portal to Access Diagnostic Test Results by Emergency Patients at an Academic Medical Center: Retrospective Study	Foster, B.; Krasowski, M. D.	2019	Journal of Medical Internet Research	21	6	NA	10.2196/13791	Foster 2019	Irrelevant	Excluded by TI/AB review
1859	Predictors of emergency department use among individuals with current or previous experience of homelessness	Gabet, M.; Grenier, G.; Cao, Z.; Fleury, M. J.	2019	International Journal of Environmental Research and Public Health	16	24	NA	10.3390/ijerph16244965	Gabet 2019	Irrelevant	Excluded by TI/AB review
1860	GCS 15: When mild TBI isn,Äôt so mild	Ganti, L.; Stead, T.; Daneshvar, Y.; Bodhit, A. N.; Pulvino, C.; Ayala, S. W.; Peters, K. R.	2019	Neurological Research and Practice	NA	1	NA	10.1186/s42466-018-0001-1	Ganti 2019	Irrelevant	Excluded by TI/AB review

S4 Table. All studies identified in the literature search

1861	Effectiveness of integrative medicine group visits in chronic pain and depressive symptoms: A randomized controlled trial	Gardiner, P.; Luo, M.; D'Amico, S.; Gergen-Barnett, K.; White, L. F.; Saper, R.; Mitchell, S.; Liebschutz, J. M.	2019	PLoS One	14	12	NA	10.1371/journal.pone.0225540	Gardiner 2019	Irrelevant	Excluded by TI/AB review
1862	Unplanned return visits to the paediatric emergency department: Caregiver and physician perspectives	Gardner, K.; Taylor, B.	2019	Canadian Journal of Emergency Medicine	21	NA	S81	10.1017/cem.2019.241	Gardner 2019	Irrelevant	Excluded by TI/AB review

S4 Table. All studies identified in the literature search

1863	Barriers to self-compassion in the eating disorders: The factor structure of the fear of self-compassion scale	Geller, J.; Iyar, M. M.; Kelly, A. C.; Srikameswaran, S.	2019	Eating Behaviors	35	NA	NA	10.1016/j.eatbeh.2019.101334	Geller 2019	Irrelevant	Excluded by TI/AB review
1864	Multifaceted interventions improve medication adherence and reduce acute hospitalization rates in medicaid [illegible]	Gelzer, A. D.; Gao, W.; Keleti, D.; Donia, T.; Megargell, L.; Kreitman, J.; Michael, K. E.	2019	Journal of Asthma	56	2	190-199	10.1080/02770903.2018.1439954	Gelzer 2019	Irrelevant	Excluded by TI/AB review
1865	Radiologic findings in intimate partner violence	George, E.; Phillips, C. H.; Shah, N.; Lewis-O,ÄôConnor, A.; Rosner, B.; Stoklosa, H. M.; Khurana, B.	2019	Radiology	291	1	62-69	10.1148/radiol.2019180801	George 2019	Irrelevant	Excluded by TI/AB review
1866	Volunteer engagement in the emergency department: A scoping review	Glanz, S.; Ellis, B.; Nelson, M.; Thompson, C.; McLeod, S.; Melady, D.	2019	Canadian Journal of Emergency Medicine	21	NA	S96-S97	10.1017/cem.2019.283	Glanz 2019	Irrelevant	Excluded by TI/AB review

S4 Table. All studies identified in the literature search

1867	Code black: An evaluation of a standard operating procedure to expedite neurosurgical intervention in traumatic brain injury	Gleed, P.; Zolfaghari, P.; Patel, A.	2019	Journal of the Intensive Care Society	20	2	223-224	10.1177/1751143719835452	Gleed 2019	Irrelevant	Excluded by TI/AB review
1868	Impact of health service interventions on acute hospital use in community-dwelling persons with dementia: A [illegible]	Godard-Sebillotte, C.; Le Berre, M.; Schuster, T.; Trottier, M.; Vedel, I.	2019	PLoS One	14	6	NA	10.1371/journal.pone.0218426	Godard-Sebillotte 2019	Irrelevant	Excluded by TI/AB review
1869	Emergency Department-Provided Home Blood Pressure Devices Can Help Detect Undiagnosed Hypertension	Goldberg, E. M.; Wilson, T.; Jambhekar, B.; Marks, S. J.; Boyajian, M.; Merchant, R. C.	2019	High Blood Pressure and Cardiovascular Prevention	26	1	45-53	10.1007/s40292-019-00300-0	Goldberg 2019	Irrelevant	Excluded by TI/AB review

S4 Table. All studies identified in the literature search

1870	The association of neighborhood racial mix and ED visit count in a cohort of patients on hemodialysis	Golestaneh, L.; Farzami, A.; Madu, C.; Johns, T.; Melamed, M. L.; Norris, K. C.	2019	BMC Nephrol	20	1	343	10.1186/s12882-019-1520-x	Golestaneh 2019	Irrelevant	Excluded by TI/AB review
1872	Implementing a case management intervention for frequent users of the emergency department (I-CaM): an effectiveness- [illegible]	Grazioli, V. S.; Moullin, J. C.; Kasztura, M.; Canepa-Allen, M.; Hugli, O.; Griffin, J.; Vu, F.; Hudon, C.; Jackson, Y.; Wolff, H.; Burnand, B.; [illegible]	2019	BMC Health Services Research	19	1	28	10.1186/s12913-018-3852-9	Grazioli 2019	Irrelevant	Excluded by TI/AB review
1873	Trends in Emergency Department Use by Rural and Urban Populations in the United States	Greenwood-Ericksen, Margaret B.; Kocher, Keith	2019	JAMA Network Open	NA	NA	e191919-e191919	10.1001/jamanetworkopen.2019.1919	Greenwood-Ericksen 2019	Irrelevant	Excluded by TI/AB review
1874	Disparities in Pediatric Epilepsy Remission Are Associated With Race and Ethnicity	Gregerson, C. H. Y.; Bakian, A. V.; Wilkes, J.; Knighton, A. J.; Nkoy, F.; Sweney, M.; Filloux, F. M.; Bonkowsky, J. L.	2019	Journal of Child Neurology	NA	NA	NA	10.1177/0883073819866623	Gregerson 2019	Irrelevant	Excluded by TI/AB review

S4 Table. All studies identified in the literature search

1876	Men,Äôs health equity: A handbook	Griffith, Derek M.; Bruce, Marino A.; Thorpe, Roland J., Jr.	2019	NA	NA	NA	NA	10.4324/9781315167428	Griffith 2019	Irrelevant	Excluded by TI/AB review
1877	Racial Disparities in Asthma-Related Health Outcomes in Children with Severe/Difficult-to-Treat Asthma	Guilbert, T.; Zeiger, R. S.; Haselkorn, T.; Iqbal, A.; Alvarez, C.; Mink, D. R.; Chipps, B. E.; Szefler, S. J.	2019	Journal of Allergy and Clinical Immunology: In Practice	7	2	568-577	10.1016/j.jaip.2018.07.050	Guilbert 2019	Irrelevant	Excluded by TI/AB review
1878	Discharge against medical advice in culturally and linguistically diverse Australian children	Guo, X. Y.; Woolfenden, S.; McDonald, G.; Saavedra, A.; Lingam, R.	2019	Arch Dis Child	104	12	1150-1154	10.1136/archdischild-2019-317063	Guo 2019	Irrelevant	Excluded by TI/AB review

S4 Table. All studies identified in the literature search

1879	Patient Dissatisfaction With Primary Care and Nonurgent Emergency Department Use	Haichang, Xin	2019	Journal of Ambulatory Care Management	42	4	284-294	10.1097/JAC.0000000000000301	Haichang 2019	Irrelevant	Excluded by TI/AB review
1880	Review article: Common emergency department presenting [illegible]	Harel, N.; Moskovitch, J. T.; Weiland, T. J.; Augello, M. R.	2019	EMA - Emergency Medicine Australasia	31	2	183-192	10.1111/1742-6723.13161	Harel 2019	Irrelevant	Excluded by TI/AB review
1881	Satisfaction with Inflatable Penile Prosthesis in Patients with History of ischemic Priapism	Hawksworth, D.; Koomson, A.; Burnett, A.	2019	Journal of Sexual Medicine	16	4	S138-S139	10.1016/j.jsxm.2019.01.296	Hawksworth 2019	Irrelevant	Excluded by TI/AB review
1882	Satisfaction with inflatable penile prosthesis in patients with history of ischemic priapism	Hawksworth, D. J.; Koomson, A. B.; Burnett, A. L.	2019	European Urology, Supplements	18	1	e1617	10.1016/S1569-9056(19)31171-6	Hawksworth 2019	Irrelevant	Excluded by TI/AB review

S4 Table. All studies identified in the literature search

1883	Quality of urological cancer care at the end of life for dual eligible beneficiaries and the impact of delivery system integration	Herrel, L.; Zhu, Z.; Kaye, D.; Ellimoottil, C.; Dupree, J.; Hollenbeck, B.; Miller, D.	2019	Journal of Urology	201	4	e631	10.1097/01.JU.0000556251.02636.8e	Herrel 2019	Irrelevant	Excluded by TI/AB review
1884	Healthcare-seeking behaviour, barriers to care and predictors of symptom improvement among patients with [illegible]	Hertz, J. T.; Sakita, F. M.; Kweka, G. L.; Loring, Z.; Thielman, N. M.; Temu, G.; Bartlett, J. A.	2019	International health	NA	NA	NA	10.1093/inthealth/ihz095	Hertz 2019	Irrelevant	Excluded by TI/AB review
1885	Knowledge, attitudes, and preventative practices regarding ischemic heart disease among emergency [illegible]	Hertz, J. T.; Sakita, F. M.; Manavalan, P.; Mmbaga, B. T.; Thielman, N. M.; Staton, C. A.	2019	Public Health	175	NA	60-67	10.1016/j.puhe.2019.06.017	Hertz 2019	Irrelevant	Excluded by TI/AB review

S4 Table. All studies identified in the literature search

1886	Barriers to Primary Care Access in Rural Medically Underserved	Hewitt, Suzanne; McNiesh, Susan; Fink, Lilo	2019	Online Journal of Rural Nursing & Health Care	19	2	127-155	10.14574/ojrnhc.v19i2.582	Hewitt 2019	Irrelevant	Excluded by TI/AB review
1887	National and Regional Variation in Local Primary Care Physician Density Relative to the Uninsured and the Affordable [illegible]	Hill, Barry C.; Coster, Daniel; Black, David R.	2019	Inquiry (00469580)	56	NA	N.PAG-N.PAG	10.1177/0046958019873807	Hill 2019	Irrelevant	Excluded by TI/AB review
1888	Development of best practice guidelines for suicide-related crisis response and aftercare in the emergency department or other acute settings: A Delphi	Hill, N. T. M.; Shand, F.; Torok, M.; Halliday, L.; Reavley, N. J.	2019	BMC Psychiatry	19	1	NA	10.1186/s12888-018-1995-1	Hill 2019	Irrelevant	Excluded by TI/AB review
1889	Association of Sociodemographic Factors with Adherence to Age-Specific Guidelines for [illegible]	Hills-Dunlap, J. L.; Melvin, P.; Graham, D. A.; Kashtan, M. A.; Anandalwar, S. P.; Rangel, S. J.	2019	JAMA Pediatrics	173	7	640-647	10.1001/jamapediatrics.2019.1061	Hills-Dunlap 2019	Irrelevant	Excluded by TI/AB review

S4 Table. All studies identified in the literature search

1890	Socioeconomic disparities in IBD: Outcomes in emergency department utilization and outpatient follow-	Horton, N.; Kaur, R.; Sadiq, O.; Ghanimeh, M. A.; Singh, M.; Kaur, N.	2019	American Journal of Gastroenterology	114	NA	S9-S10	10.14309/01.ajg.0000613112.82877.65	Horton 2019	Irrelevant	Excluded by TI/AB review
1891	Culture and psychological interventions	Huang, Cindy Y.; Zane, Nolan; Matsumoto, David; Hwang, Hyisung C.	2019	The handbook of culture and psychology., 2nd ed.	NA	NA	468-506	10.1093/oso/9780190679743.003.0015	Huang 2019	Irrelevant	Excluded by TI/AB review
1892	Emergency medicine resident perception of abuse by consultants: Results of a national survey	Huber, M.; Lopez, J.; McLaughlin, T.; Tomanec, A.; Xu, K. T.; Messman, A. M.; Miller, M.; Richman, P. B.; Hopson, L. R.	2019	Academic Emergency Medicine	26	NA	S158	10.1111/acem.13756	Huber 2019	Irrelevant	Excluded by TI/AB review

S4 Table. All studies identified in the literature search

1893	Utilizing Grip Strength prior to Three-piece IPP Placements as a Predictor for Post-operative Patient Satisfaction. Current Pre- and Post-operative Follow up Data	Jahn, H.; Patel, R.; Canter, D.; Laborde, E.	2019	Journal of Sexual Medicine	16	4	S104	10.1016/j.jsxm.2019.01.220	Jahn 2019	Irrelevant	Excluded by TI/AB review
1894	Quantifying geographic barriers to trauma care: Urban-rural variation in prehospital [illegible]	Jarman, M. P.; Hashmi, Z.; Zerhouni, Y.; Udyavar, R.; Newgard, C.; Salim, A.; Haider, A. H.	2019	Journal of Trauma and Acute Care Surgery	87	1	173-180	10.1097/TA.0000000000002335	Jarman 2019	Irrelevant	Excluded by TI/AB review
1895	Evaluation of a mobile telesimulation unit to train rural and remote practitioners on high-acuity low-	Jewer, Jennifer; Parsons, Michael H.; Dunne, Cody; Smith, Andrew; Dubrowski, Adam	2019	Journal of Medical Internet Research	21	8	NA	10.2196/14587	Jewer 2019	Irrelevant	Excluded by TI/AB review

S4 Table. All studies identified in the literature search

1896	Racial and Ethnic Inequities in Postpartum Pain Evaluation and Management	Johnson, J. D.; Asiodu, I. V.; McKenzie, C. P.; Tucker, C.; Tully, K. P.; Bryant, K.; Verbiest, S.; Stuebe, A. M.	2019	Obstetrics and Gynecology	134	6	1155-1162	10.1097/AOG.0000000000003505	Johnson 2019	Irrelevant	Excluded by TI/AB review
1897	Effects of deprivation, ethnicity, gender and age on distal radius fracture incidence and surgical intervention rate	Johnson, N. A.; Jeffery, J.; Stirling, E.; Thompson, J.; Dias, J. J.	2019	Bone	121	NA	8-Jan	10.1016/j.bone.2018.12.018	Johnson 2019	Irrelevant	Excluded by TI/AB review
1898	Health disparities in the misdiagnosis of cellulitis	Johnson, R. K.; O'Connor, M. C.; Gyurjyan-Bunch, A.; Ferguson, K. P.; Sheehan, B. E.; Knapp, B.; Counselman, F.; Van Voorhees, A. S.	2019	Academic Emergency Medicine	26	NA	S304-S305	10.1111/acem.13756	Johnson 2019	Irrelevant	Excluded by TI/AB review

S4 Table. All studies identified in the literature search

1899	Multi-model implementation of evidence-based care in the treatment of opioid use disorder in Pennsylvania	Kawasaki, S.; Francis, E.; Mills, S.; Buchberger, G.; Hogentogler, R.; Kraschnewski, J.	2019	Journal of Substance Abuse Treatment	106	NA	58-64	10.1016/j.jsat.2019.08.016	Kawasaki 2019	Irrelevant	Excluded by TI/AB review
1900	Racial/Ethnic Disparities in Pain Treatment: Evidence From Oregon Emergency [illegible]	Kennel, J.; Withers, E.; Parsons, N.; Woo, H.	2019	Med Care	57	12	924-929	10.1097/mlr.0000000000001208	Kennel 2019	Irrelevant	Excluded by TI/AB review
1901	Controller adherence following hospital discharge in high risk children: A pilot randomized trial of text message reminders	Kenyon, C. C.; Gruschow, S. M.; Quarshie, W. O.; Griffis, H.; Leach, M. C.; Zorc, J. J.; Bryant-Stephens, T. C.; Miller, V. A.; Feudtner, C.	2019	Journal of Asthma	56	1	95-103	10.1080/02770903.2018.1424195	Kenyon 2019	Irrelevant	Excluded by TI/AB review

S4 Table. All studies identified in the literature search

1902	UNDERSTANDING FACTORS INFLUENCING OUTCOMES IN OLDER RURAL BREAST [illegible]	Kenzik, K.; Rocque, G. B.; Landier, W.; Bhatia, S.	2019	Journal of Geriatric Oncology	10	6	S17	10.1016/S1879-4068(19)31147-6	Kenzik 2019	Irrelevant	Excluded by TI/AB review
1903	Patients' reports of harm from failures of care coordination	Kern, L. M.; Reshetnyak, E.; Colantonio, L.; Muntner, P.; David Rhodes, J.; Casalino, L. P.; Rajan, M.; Pesko, M.; Marino, J.; Pinheiro, L. C.; Safford, M. M.	2019	Journal of General Internal Medicine	34	2	S310	10.1007/11606.1525-1497	Kern 2019	Irrelevant	Excluded by TI/AB review
1904	Listening to their heart: A randomized trial of narrative medicine	Kheirbek, R. E.; Marr, B.; Herr, D.	2019	Circulation	140	NA	NA	10.1161/circ.140.suppl_1.16456	Kheirbek 2019	Irrelevant	Excluded by TI/AB review

S4 Table. All studies identified in the literature search

1905	The Complex Association of Race/Ethnicity With Pain Treatment Quality in an Urban Medical Center With 2 Pediatric Emergency Departments	Kipping, E. M.; Chamberlain, J. M.	2019	Pediatr Emerg Care	35	12	815-820	10.1097/pec.0000000000001401	Kipping 2019	Irrelevant	Excluded by TI/AB review
1907	Restrictive interventions in Victorian emergency departments: What is really going on?	Knott, J.; Dobson, S.; Graudins, A.; Mitra, B.; Bartley, B.; Chapman, P.	2019	EMA - Emergency Medicine Australasia	31	NA	7	10.1111/1742-6723.13239	Knott 2019	Irrelevant	Excluded by TI/AB review
1908	Urban partnerships to address health literacy in low-income and [illegible]	Knox, Joshua F.; Vuyk, Amy; Keller, Abiola	2019	JAAPA: Journal of the American Academy of [illegible]	32	12	53-53	10.1097/01.JAA.0000603052.22021.c0	Knox 2019	Irrelevant	Excluded by TI/AB review

S4 Table. All studies identified in the literature search

1909	The Relationship Between Depressive Symptoms and Health Services [illegible]	Kong, D.; Li, M.; Wang, J.; Davitt, J. K.; Dong, X.	2019	The Gerontologist	59	3	447-455	10.1093/geront/gny010	Kong 2019	Irrelevant	Excluded by TI/AB review
1910	Correlates of Emergency Department Service Utilization Among U.S. Chinese Older Adults	Kong, D.; Li, M.; Wong, Y. L. I.; Wang, J.; Sun, B. C.; Dong, X.	2019	Journal of immigrant and minority health	21	5	938-945	10.1007/s10903-018-0828-0	Kong 2019	Irrelevant	Excluded by TI/AB review
1911	Clinical history of children with Crohn's disease who require first time surgery	Korotkaya, Y.; Naraparaju, G.; Miller, S.; Cuffari, C.	2019	Journal of Pediatric Gastroenterology and Nutrition	69	NA	NA	10.1097/MPG.0000000000002518	Korotkaya 2019	Irrelevant	Excluded by TI/AB review

S4 Table. All studies identified in the literature search

1912	An emergency department-based health behavior intervention to improve outpatient follow-up for chest pain patients	Kumar, V. A.; Fitz, H.; May, S.; Reed, B.; Mathew, S.; Lovelace, A.; Levy, P.; Limkakeng, A.	2019	Academic Emergency Medicine	26	NA	S156	10.1111/acem.13756	Kumar 2019	Irrelevant	Excluded by TI/AB review
1913	The impact of urban street tree species on air quality and respiratory illness: A spatial analysis of large-scale, high- [illegible]	Lai, Y.; Kontokosta, C. E.	2019	Health and Place	56	NA	80-87	10.1016/j.healthplace.2019.01.016	Lai 2019	Irrelevant	Excluded by TI/AB review
1914	A review of racial/ethnic disparities in pediatric trauma care, treatment, and outcomes	Laplant, M. B.; Hess, D. J.	2019	Journal of Trauma and Acute Care Surgery	86	3	540-550	10.1097/TA.0000000000002160	Laplant 2019	Irrelevant	Excluded by TI/AB review
1915	Are caregivers who respond to the child HCAHPS survey reflective of all hospitalized pediatric patients?	Lee, B.; Hollenbeck-Pringle, D.; Goldman, V.; Biondi, E.; Alverson, B.	2019	Hospital Pediatrics	9	3	162-169	10.1542/hpeds.2018-0139	Lee 2019	Irrelevant	Excluded by TI/AB review

S4 Table. All studies identified in the literature search

1916	The associations between social factors and suboptimal use of paediatric dental services (Tiny Teeth): a mixed method study	Lee-Barber, I.; Hann, G.; Singh, G.; Palit, V.; Al-Jaddir, G.	2019	The Lancet	394	NA	S66	10.1016/S0140-6736(19)32863-6	Lee-Barber 2019	Irrelevant	Excluded by TI/AB review
1917	Evaluating Suspected Acute MI in the Emergency Department: What Is and What Should Never Be	Levy, P. D.; Mahler, S. A.	2019	Journal of the American College of Cardiology	74	4	495-497	10.1016/j.jacc.2019.05.047	Levy 2019	Irrelevant	Excluded by TI/AB review
1918	Telemedicine for Interfacility Nurse Handoffs	Lieng, M. K.; Siefkes, H. M.; Rosenthal, J. L.; Sauers-Ford, H. S.; Mouzoon, J. L.; Sigal, I. S.; Dayal, P.; Chen, S. T.; McBeth, C. L.; Dial, S.; Dizon, G.; [illegible]	2019	Pediatric critical care medicine : a journal of the Society of Critical Care Medicine and the [illegible]	20	9	832-840	10.1097/PCC.0000000000002011	Lieng 2019	Irrelevant	Excluded by TI/AB review

S4 Table. All studies identified in the literature search

1920	Improving asthma control by facilitating patient-centered care at school through telemedicine	Lin, N. Y.; Miller, J. L.; Ramsey, R. R.; Hommel, K. A.; Guilbert, T. W.	2019	American Journal of Respiratory and Critical Care Medicine	199	9	NA	NA	Lin 2019	Irrelevant	Excluded by TI/AB review
1921	Impact of critical care recovery center on 2-year healthcare utilization outcomes	Lindroth, H.; Wang, S.; Xu, C.; Hanneman, P. H.; Gao, S.; Khan, S. H.; Jawed, Y. T.; Boustani, M.; Khan, B. A.	2019	American Journal of Respiratory and Critical Care Medicine	199	9	NA	NA	Lindroth 2019	Irrelevant	Excluded by TI/AB review
1922	Adherence trajectories of buprenorphine therapy among pregnant women [illegible]	Lo-Ciganic, W. H.; Donohue, J. M.; Kim, J. Y.; Krans, E. E.; Jones, B. L.; Kelley, D.; James, A. [illegible]	2019	Pharmacoepidemiology and Drug Safety	28	1	80-89	10.1002/pds.4647	Lo-Ciganic 2019	Irrelevant	Excluded by TI/AB review
1924	Racism Is Not a Factor in Door-to-electrocardiogram Times of Patients With Symptoms of Acute Coronary Syndrome: A Prospective, Observational [illegible]	Mackay, M. H.; Ratner, P. A.; Veenstra, G.; Scheuermeyer, F. X.; Grubisic, M.; Ramanathan, K.; Murray, C.; Humphries, K. H.	2019	Acad Emerg Med	26	5	491-500	10.1111/acem.13569	Mackay 2019	Irrelevant	Excluded by TI/AB review

S4 Table. All studies identified in the literature search

1928	Variation and Disparities in Awareness of Myocardial Infarction Symptoms Among Adults in the United States	Mahajan, Shiwani; Valero-Elizondo, Javier; Khera, Rohan; Desai, Nihar R.; Blankstein, Ron; Blaha, Michael J.; Virani, Salim S.; Kash, Bita A.; Zoghbi, William A.; Krumholz, Harlan M.; Nasir, Khurram	2019	JAMA Network Open	2	12	e1917885-e1917885	10.1001/jamanetworkopen.2019.17885	Mahajan 2019	Irrelevant	Excluded by TI/AB review
1930	Experiences of uk african-caribbean prostate cancer survivors of discharge to primary care	Margariti, C.; Gannon, K.; Thompson, R.; Walsh, J.; Green, J.	2019	Ethnicity & health	NA	NA	NA	10.1080/13557858.2019.1606162	Margariti 2019	Irrelevant	Excluded by TI/AB review
1931	The impact of a comprehensive electronic patient portal on the health service use: an interrupted time-series analysis	Mart√≠nez Nicol√°s, I.; L√™ Cook, B.; Flores, M.; Del Olmo Rodriguez, M.; Hern√°ndez Rodr√≠guez, C.; Llamas Sillero, P.; Baca-Garcia, E.	2019	European Journal of Public Health	29	3	413-418	10.1093/eurpub/cky257	Mart√≠nezNicol√°s 2019	Irrelevant	Excluded by TI/AB review

S4 Table. All studies identified in the literature search

1932	Mistreatment of providers by patients in emergency medicine	Massouleh, C.; Appelbaum, N.; Santen, S.; Moll, J.; Dhindsa, H. S.; Hemphill, R. R.; [illegible]	2019	Academic Emergency Medicine	26	NA	S144	10.1111/acem.13756	Massouleh 2019	Irrelevant	Excluded by TI/AB review
1933	Medicare‚Äôs bundled payments for care improvement initiative maintained quality of care for [illegible]	Maughan, B. C.; Kahvecioglu, D. C.; Marrufo, G.; Gerding, G. M.; Dennen, S.; Marshall, J. K.; Cooper, D. M.; [illegible]	2019	Health Affairs	38	4	561-568	10.1377/hlthaff.2018.05146	Maughan 2019	Irrelevant	Excluded by TI/AB review
1934	Demographic Differences in Disruptive Mood Dysregulation Disorder Symptoms in ADHD, Autism, and General Population Samples	Mayes, Susan D.; Calhoun, Susan L.; Waxmonsky, James G.; Kokotovich, Cari; Baweja, Raman; Lockridge, Robin; Bixler, Edward O.	2019	Journal of Attention Disorders	23	8	849-858	10.1177/1087054716664409	Mayes 2019	Irrelevant	Excluded by TI/AB review
1935	Promoting Affirmative Transgender Health Care Practice Within Hospitals: An IPE Standardized Patient Simulation for Graduate Health Care Learners	McCave, E. L.; Aptaker, D.; Hartmann, K. D.; Zucconi, R.	2019	MedEdPORTAL	15	NA	10861	10.15766/mep_2374-8265.10861	McCave 2019	Irrelevant	Excluded by TI/AB review

S4 Table. All studies identified in the literature search

1937	Do Employees From Less-Healthy Communities Use More Care and Cost More? Seeking to [illegible]	McIntire, R. K.; Romney, M. C.; Alonzo, G.; Hutt, J.; Bartolome, L.; Wood, G.; Klein, G.; Goldfarb, N. I.	2019	Preventing chronic disease	16	NA	E95	10.5888/pcd16.180631	McIntire 2019	Irrelevant	Excluded by TI/AB review
1938	Leadership and diversity in psychology: Moving beyond the limits	McIntosh, Maureen; Nicholas, Helen; Huq, Afreen Husain	2019	NA	NA	NA	NA	10.4324/9780429432606	McIntosh 2019	Irrelevant	Excluded by TI/AB review
1939	Racial and ethnic differences in the experience and treatment of noncancer pain	Meints, S. M.; Cortes, A.; Morals, C. A.; Edwards, R. R.	2019	Pain Management	9	3	317-334	10.2217/pmt-2018-0030	Meints 2019	Irrelevant	Excluded by TI/AB review
1940	'You have to be part of the process': A qualitative analysis of older African [illegible]	Mitchell, Jamie; Williams, Ed-Dee G.; Perry, Ramona; Lobo, Kavitha	2019	American journal of men's health	13	4	NA	10.1177/1557988319861569	Mitchell 2019	Irrelevant	Excluded by TI/AB review

S4 Table. All studies identified in the literature search

1941	"Just too busy living in the moment and surviving": barriers to accessing health care for structurally vulnerable populations at end-of-life	Mollison, A.; Giesbrecht, M.; Rose, C.; Showler, C.; Rounds, K.; Stajduhar, K. I.; Kvakic, K.; Teal, T.; Pauly, B.; McNeil, R.; Reimer-Kirkham, S.; Dosani, N.; Wallace, B.; Showler, G.; Meagher, C.; Gleave, D.	2019	BMC Palliative Care	18	1	N.PAG-N.PAG	10.1186/s12904-019-0396-7	Mollison 2019	Irrelevant	Excluded by TI/AB review
1943	Targeted Education Across Clinical Settings Improves	Molloy, M. J.; Tamaroff, J.; McDaniel, L.; Genies, M. C.	2019	Clinical Pediatrics	58	#####	1284-1290	10.1177/0009922819852982	Molloy 2019	Irrelevant	Excluded by TI/AB review
1944	Can asthma be well controlled with NAEPP guideline care in morbidly obese children? The Breathmobile	Morphew, T.; Galant, S. P.	2019	Annals of Allergy, Asthma and Immunology	122	2	167-174	10.1016/j.anai.2018.10.019	Morphew 2019	Irrelevant	Excluded by TI/AB review

S4 Table. All studies identified in the literature search

1945	Loneliness in Primary Care Patients: A Prevalence Study	Mullen, R. A.; Tong, S.; Sabo, R. T.; Liaw, W. R.; Marshall, J.; Nease, D. E.; Krist, A. H.; Frey, J. J.	2019	Annals of Family Medicine	17	2	108-115	10.1370/afm.2358	Mullen 2019	Irrelevant	Excluded by TI/AB review
1947	Is There Gender Discrimination in Acute Renal Colic Pain Management? A Retrospective Analysis in an Emergency Department Setting	Naamany, E.; Reis, D.; Zuker-Herman, R.; Drescher, M.; Glezerman, M.; Shiber, S.	2019	Pain Manag Nurs	20	6	633-638	10.1016/j.pmn.2019.03.004	Naamany 2019	Irrelevant	Excluded by TI/AB review
1950	Effect of Physical Activity Coaching on Acute Care and Survival among Patients with Chronic Obstructive Pulmonary [illegible]	Nguyen, H. Q.; Moy, M. L.; Liu, I. L. A.; Fan, V. S.; Gould, M. K.; Desai, S. A.; Towner, W. J.; Yuen, G.; Lee, J. S.; Park, S. J.; Xiang, A. H.	2019	JAMA Network Open	2	8	NA	10.1001/jamanetworkopen.2019.9657	Nguyen 2019	Irrelevant	Excluded by TI/AB review

S4 Table. All studies identified in the literature search

1951	Outpatient pneumonia management before and after the American	Noble, J.; Petrovich, M. P.; Brady, J.; Haslitt, D.; Desai, S. J.; Smith, E.; Sullivan, A.;	2019	Academic Emergency Medicine	26	NA	S99	10.1111/acem.13756	Noble 2019	Irrelevant	Excluded by TI/AB review
1952	Perceived Adequacy of Tangible Social Support and Associations with Health Outcomes Among Older Primary Care Patients	O‚ÄôConor, R.; Benavente, J. Y.; Arvanitis, M.; Curtis, L. M.; Eldeirawi, K.; Hasnain-Wynia, R.; Federman, A. D.; Hebert-Beirne, J.; Wolf, M. S.	2019	Journal of General Internal Medicine	34	11	2368-2373	10.1007/s11606-019-05110-7	O‚ÄôConor 2019	Irrelevant	Excluded by TI/AB review
1953	Association of Race and Ethnicity with Total Hip Arthroplasty Outcomes in a Universally Insured Population	Okike, K.; Chan, P. H.; Prentice, H. A.; Navarro, R. A.; Hinman, A. D.; Paxton, E. W.	2019	Journal of Bone and Joint Surgery - American Volume	101	13	1160-1167	10.2106/JBJS.18.01316	Okike 2019	Irrelevant	Excluded by TI/AB review

S4 Table. All studies identified in the literature search

1954	Association Between Care Management and Outcomes Among Patients With Complex Needs in Medicare Accountable Care Organizations	Ouayogod√©, Mari√©tou H.; Mainor, Alexander J.; Meara, Ellen; Bynum, Julie P. W.; Colla, Carrie H.	2019	JAMA Network Open	2	7	e196939-e196939	10.1001/jamanetworkopen.2019.6939	Ouayogod√© 2019	Irrelevant	Excluded by TI/AB review
1955	A scoping review on patient race, ethnicity, and care in the emergency department	Owens, A.; Holroyd, B.; McLane, P.	2019	Canadian Journal of Emergency Medicine	21	NA	S96	10.1017/cem.2019.281	Owens 2019	Irrelevant	Excluded by TI/AB review
1956	Environment and health	Park, Giyoung; Evans, Gary W.; Llewellyn, Carrie D.;	2019	The Cambridge handbook	NA	NA	32-36	NA	Park 2019	Irrelevant	Excluded by TI/AB review
1957	Enhancing community capacity to improve cancer care delivery and the effect on patient-reported outcomes, healthcare utilization and total [illegible]	Patel, M. I.; Ramirez, D.; Agajanian, R.; Agajanian, H. H.; Bhattacharya, J.; Coker, T.	2019	Journal of Clinical Oncology	37	NA	NA	10.1200/JCO.2019.37.15_suppl.6522	Patel 2019	Irrelevant	Excluded by TI/AB review

S4 Table. All studies identified in the literature search

1958	Enhancing community capacity to deliver value-based cancer care at the end-of-life	Patel, M. I.; Ramirez, D.; Agajanian, R.; Agajanian, H. H.; Coker, T.	2019	Journal of Clinical Oncology	37	NA	NA	10.1200/JCO.2019.37.27-suppl.4	Patel 2019	Irrelevant	Excluded by TI/AB review
1959	Does cross-cultural communication training for physicians improve pediatric asthma outcomes? A randomized trial	Patel, Minal R.; Song, Peter X. K.; Bruzzese, Jean-Marie; Hao, Wei; Evans, David; Thomas, Lara J.; Pinkett-Heller, Marcia; Meyerson, Karen; Brown, Randall W.	2019	Journal of Asthma	56	3	273-284	10.1080/02770903.2018.1455856	Patel 2019	Irrelevant	Excluded by TI/AB review
1963	Multidisciplinary Approach and Outcomes of Tele-neurology: A Review	Patel, U. K.; Malik, P.; DeMasi, M.; Lunagariya, A.; Jani, V. B.	2019	Cureus	11	4	e4410	10.7759/cureus.4410	Patel 2019	Irrelevant	Excluded by TI/AB review

S4 Table. All studies identified in the literature search

1965	Racial disparities in end-of-life care among Louisiana cancer decedents in a population-based study from 2011-2017	Perry, L. M.; Walsh, L. E.; Horswell, R.; Miele, L.; Chu, S.; Melancon, B.; Lefante, J.; Blais, C. M.; Hoerger, M.	2019	Journal of Clinical Oncology	37	31	NA	10.1200/JCO.2019.37.31-suppl.46	Perry 2019	Irrelevant	Excluded by TI/AB review
1966	A research and intervention agenda for children with incarcerated parents and their families	Poehlmann-Tynan, Julie; Eddy, J. Mark; Eddy, J. Mark; Poehlmann-Tynan, Julie	2019	Handbook on children with incarcerated parents: Research, policy, and practice., [illegible]	NA	NA	353-371	10.1007/978-3-030-16707-3_24	Poehlmann-Tynan 2019	Irrelevant	Excluded by TI/AB review
1967	Understanding geographical patterning of self-harm prevalence within a diverse urban population: [illegible]	Polling, C.; Bakolis, I.; Hotopf, M.; Hatch, S. L.	2019	The Lancet	394	NA	S78	10.1016/S0140-6736(19)32875-2	Polling 2019	Irrelevant	Excluded by TI/AB review
1968	Medical Mistrust, Racism, and Delays in Preventive Health Screening Among African-American Men	Powell, W.; Richmond, J.; Mohottige, D.; Yen, I.; Joslyn, A.; Corbie-Smith, G.	2019	Behavioral Medicine	45	2	102-117	10.1080/08964289.2019.1585327	Powell 2019	Irrelevant	Excluded by TI/AB review

S4 Table. All studies identified in the literature search

1969	Use of Emergency Departments for Preventative Care Among Adults in the United States: Estimates From the 2017 National [illegible]	Primm, K.; Muraleetharan, D.; Gilreath, T.	2019	J Emerg Med	57	4	578-586	10.1016/j.jemermed.2019.06.004	Primm 2019	Irrelevant	Excluded by TI/AB review
1970	Therapeutic Plasma Exchange (PLEX) in Thrombotic Microangiopathy (TMA): Experiences of an Inner-City Hospital over a Decade	Putta, A.; Shaka, H.; Banskota, S. U.; Singh, S. R. K.; Malapati, S.; Farooq, M. Z.; Batra, K. K.	2019	Blood	134	NA	3691	10.1182/blood-2019-124265	Putta 2019	Irrelevant	Excluded by TI/AB review
1972	Racial differences in sepsis recognition in the emergency department	Raman, J.; Johnson, T. J.; Hayes, K.; Balamuth, F.	2019	Pediatrics	144	4	NA	10.1542/peds.2019-0348	Raman 2019	Irrelevant	Excluded by TI/AB review

S4 Table. All studies identified in the literature search

1975	DIRECT VS INDIRECT ADMISSION TO INTENSIVE CARE FROM EMERGENCY CARE CENTER: A QUALITY IMPROVEMENT [illegible]	Ramos-Chaves, B.; Wiese-Rometsch, W.; Smith, R.; Geary, M.; Hamad, K.; Rodriguez, Y.; Dawkins, K.; George, J.; Seaman, J.	2019	Chest	156	4	A126	10.1016/j.chest.2019.08.202	Ramos-Chaves 2019	Irrelevant	Excluded by TI/AB review
1976	Impact of brain metastases on end-of-life health care utilization	Ray, E. M.; Dunham, L.; Reeder-Hayes, K. E.; Sanoff, H. K.	2019	Journal of Clinical Oncology	37	NA	NA	10.1200/JCO.2019.37.27-suppl.116	Ray 2019	Irrelevant	Excluded by TI/AB review
1977	Innovations in Practice: CUES-Ed: an in-service evaluation of a new universal cognitive behavioural early mental health intervention [illegible]	Redfern, A.; Jolley, S.; Bracegirdle, K.; Browning, S.; Plant, D.	2019	Child and Adolescent Mental Health	24	2	187-191	10.1111/camh.12309	Redfern 2019	Irrelevant	Excluded by TI/AB review

S4 Table. All studies identified in the literature search

1978	Implementation and evaluation of an integrated hospital-to-home transitional care intervention for older adults with stroke and multimorbidity: a feasibility study	Reid, Maureen; Valaitis, Ruta; Bartholomew, Amy; Fisher, Kathryn; Fleck, Rebecca; Ploeg, Jenny; Salerno, Jennifer; Thabane, Lehana; Gafni, Amiram; Archer, Norman	2019	International Journal of Integrated Care (IJIC)	19	S1	2-Jan	10.5334/ijic.s3367	Reid 2019	Irrelevant	Excluded by TI/AB review
1979	Patient Uncertainty as a Predictor of 30‚Äêday Return Emergency Department Visits: An Observational Study	Rising, Kristin L.; LaNoue, Marianna D.; Gerolamo, Angela M.; Doty, Amanda M. B.; Gentsch, Alexzandra T.; Powell, Rhea E.; Runyon, Michael S.	2019	Academic Emergency Medicine	26	5	501-509	10.1111/acem.13621	Rising 2019	Irrelevant	Excluded by TI/AB review
1980	Neuropathic Pain Is Associated with Poor Health-Related Quality of Life in Adolescents with Sickle Cell Disease	Roman, M.; Highland, J.; Retherford, D.; Pan, A.; Panepinto, J.; Brandow, A. M.	2019	Blood	134	NA	417	10.1182/blood-2019-127626	Roman 2019	Irrelevant	Excluded by TI/AB review
1982	Race/Ethnicity and Sex and Opioid Administration in the Emergency Room	Rosenbloom, J. M.; Burns, S. M.; Kim, E.; August, D. A.; Ortiz, V. E.; Houle, T. T.	2019	Anesth Analg	128	5	1005-1012	10.1213/ane.0000000000003517	Rosenbloom 2019	Irrelevant	Excluded by TI/AB review

S4 Table. All studies identified in the literature search

1984	A Mixed-Methods Evaluation of a Nurse-Led Community-Based Health Home for Ethnically Diverse Older Adults With Multimorbidity in the Adult Day Health Setting	Sadarangani, T.; Missaelides, L.; Eilertsen, E.; Jaganathan, H.; Wu, B.	2019	Policy, politics & nursing practice	20	3	131-144	10.1177/1527154419864301	Sadarangani 2019	Irrelevant	Excluded by TI/AB review
1985	Treatment of patients with sickle cell crises in the emergency department: Patients vs providers	Saef, S. H.; Griddine, A. N.; Tolley, J. H.; Thompson, L.; Holland, C.	2019	Academic Emergency Medicine	26	NA	S293	10.1111/acem.13756	Saef 2019	Irrelevant	Excluded by TI/AB review
1986	One-year follow-up of a remotely delivered epilepsy self-management program in high-risk people with epilepsy	Sajatovic, M.; Colon-Zimmermann, K.; Kahriman, M.; Fuentes-Casiano, E.; Burant, C.; Aebi, M. E.; Cassidy, K. A.; Lhatoo, S.; [illegible]	2019	Epilepsy and Behavior	96	NA	237-243	10.1016/j.yebeh.2019.04.034	Sajatovic 2019	Irrelevant	Excluded by TI/AB review

S4 Table. All studies identified in the literature search

1987	Asthma hospitalizations among homeless children in New York state	Sakai-Bizmark, R.; Chang, R. K. R.; Mena, L. A.; Webber, E. J.; Marr, E. H.; Kwong, K. Y.	2019	Pediatrics	144	2	NA	10.1542/peds.2018-2769	Sakai-Bizmark 2019	Irrelevant	Excluded by TI/AB review
1988	Treatment Works, So Who‚Äôs Afraid of PNES?	Salpekar, J. A.	2019	Epilepsy Currents	19	3	159-160	10.1177/1535759719841354	Salpekar 2019	Irrelevant	Excluded by TI/AB review

S4 Table. All studies identified in the literature search

1989	See-Hear-Feel-Speak: A Protocol for Improving Outcomes in Emergency Department Interactions with Patients with Autism Spectrum Disorder	Samet, D.; Luterman, S.	2019	Pediatric Emergency Care	35	2	157-159	10.1097/PEC.0000000000001734	Samet 2019	Irrelevant	Excluded by TI/AB review
1990	Spanish-Speaking Caregivers' Experience with an Emergency Department Pediatric Asthma-Care Bundle Quality Initiative	Santos Malav√©, C.; Diggs, D.; Sampayo, E. M.	2019	Journal of racial and ethnic health disparities	6	4	660-667	10.1007/s40615-019-00564-1	SantosMalav√© 2019	Irrelevant	Excluded by TI/AB review

S4 Table. All studies identified in the literature search

1992	Impact of Social Needs Navigation on Utilization Among High Utilizers in a Large Integrated Health System: a Quasi- [illegible]	Schickedanz, A.; Sharp, A.; Hu, Y. R.; Shah, N. R.; Adams, J. L.; Francis, D.; Rogers, A.	2019	Journal of General Internal Medicine	34	11	2382-2389	10.1007/s11606-019-05123-2	Schickedanz 2019	Irrelevant	Excluded by TI/AB review
1993	The GLMA handbook on LGBT health: Societal issues and health promotion, Vol 1	Schneider, Jason S.; Silenzio, Vincent M. B.; Erickson-Schroth, Laura	2019	NA	NA	NA	NA	NA	Schneider 2019	Irrelevant	Excluded by TI/AB review
1994	Implicit Bias: What Every Pediatrician Should Know About the Effect of Bias on Health and Future Directions	Schnierle, J.; Christian-Brathwaite, N.; Louisias, M.	2019	Current Problems in Pediatric and Adolescent Health Care	49	2	34-44	10.1016/j.cppeds.2019.01.003	Schnierle 2019	Irrelevant	Excluded by TI/AB review

S4 Table. All studies identified in the literature search

1995	Adverse events experienced by patients hospitalized without definite medical acuity: A [illegible]	Sekijima, A.; Sunga, C.; Bann, M.	2019	Journal of General Internal Medicine	34	2	S125	10.1007/11606.1525-1497	Sekijima 2019	Irrelevant	Excluded by TI/AB review
1996	Association of medication adherence and patient reported outcomes in Sarcoidosis	Sharp, M.; Brown, T.; Moller, D. R.; Rand, C. S.; Riekert, K. A.; Eakin, M.	2019	American Journal of Respiratory and Critical Care Medicine	199	9	NA	NA	Sharp 2019	Irrelevant	Excluded by TI/AB review
1997	Overdose risk in patients newly prescribed opioids compared to those with continued use	Sheikh, S.; Henson, M.; Booth-Norse, A.; Dodd, C.; Smotherman, C.; Edgerton, E.; Kalynych, C.; Hendry, P. L.; Goyal, M.	2019	Academic Emergency Medicine	26	NA	S207	10.1111/acem.13756	Sheikh 2019	Irrelevant	Excluded by TI/AB review

S4 Table. All studies identified in the literature search

1998	Elevated healthcare burden among patients with active generalized tonic-clonic (GTC) convulsions	Sheikh, S.; Thompson, N.; Choi, J.; Malhotra, M.; Jehi, L.	2019	Neurology	92	15	NA	NA	Sheikh 2019	Irrelevant	Excluded by TI/AB review
1999	Subjective well-being, parent,Äìadolescent relationship, and perceived parenting style among israeli adolescents [illegible]	Shenaar-Golan, Vered; Goldberg, Alon	2019	Journal of Youth Studies	22	8	1068-1082	10.1080/13676261.2018.1563289	Shenaar-Golan 2019	Irrelevant	Excluded by TI/AB review

S4 Table. All studies identified in the literature search

2000	Emergency department-mediated cancer diagnosis among older adults in the United States	Sheridan, P.; Lyratzopoulos, G.; Murphy, J. D.; Thompson, C. A.	2019	Journal of Clinical Oncology	37	NA	NA	10.1200/JCO.2019.37.27-suppl.139	Sheridan 2019	Irrelevant	Excluded by TI/AB review
2001	Atopic Dermatitis in US Adults: From Population to Health Care Utilization	Silverberg, J. I.; Gelfand, J. M.; Margolis, D. J.; Boguniewicz, M.; Fonacier, L.; Grayson, M. H.; Ong, P. Y.; Chiesa Fuxench, Z.; Simpson, E. L.	2019	J Allergy Clin Immunol Pract	7	5	1524-1532.e2	10.1016/j.jaip.2019.01.005	Silverberg 2019	Irrelevant	Excluded by TI/AB review

S4 Table. All studies identified in the literature search

2002	Visits for Nontraumatic Dental Conditions in Ontario's Health Care System	Singhal, S.; Qui√±onez, C.; Manson, H.	2019	JDR clinical and translational research	4	1	86-95	10.1177/2380084418801273	Singhal 2019	Irrelevant	Excluded by TI/AB review
2003	146 Patients With Hospital Delirium Are Most Often Identified in the Emergency Department and Experience Longer [illegible]	Singla, A.; Sinvani, L.; Kubiak, J.; Calandrella, C.; Brave, M.; Li, T.; Perera, T.; Van Loveren, K.; Becker, L.	2019	Annals of Emergency Medicine	74	4	S58	10.1016/j.annemergmed.2019.08.151	Singla 2019	Irrelevant	Excluded by TI/AB review
2004	Understanding determinants of acute stroke thrombolysis using the tailored implementation for chronic diseases framework: a qualitative study	Skolarus, L. E.; Neshewat, G. M.; Evans, L.; Green, M.; Rehman, N.; Landis-Lewis, Z.; Schrader, J. W.; Sales, A. E.	2019	BMC Health Services Research	19	1	182	10.1186/s12913-019-4012-6	Skolarus 2019	Irrelevant	Excluded by TI/AB review

S4 Table. All studies identified in the literature search

2005	Factors Associated with Length of Stay in Emergency Departments for Pediatric Patients with Psychiatric Problems	Smith, J. L.; De Nadai, A. S.; Petrila, J.; Storch, E. A.	2019	Pediatric Emergency Care	35	10	716-721	10.1097/PEC.0000000000001651	Smith 2019	Irrelevant	Excluded by TI/AB review
2006	Does physician race and gender affect patient confidence? evidence from national patient analogue experiments	Solnick, R.; Peyton, K.; Kraft-Todd, G.; Safdar, B.; Bright, L.	2019	Academic Emergency Medicine	26	NA	S17	10.1111/acem.13756	Solnick 2019	Irrelevant	Excluded by TI/AB review
2007	Racial disparities in setting of care in women with breast cancer in South Carolina	Sorrell, M.; Wickersham, K.; Adams, S. A.; Heiney, S. P.	2019	Journal of Clinical Oncology	37	NA	NA	10.1200/JCO.2019.37.15_suppl.e18146	Sorrell 2019	Irrelevant	Excluded by TI/AB review

S4 Table. All studies identified in the literature search

2008	Impact of Health Care and Socioeconomic Needs on Health Care Utilization and Disease Management: The University of New Mexico Hospital Care One Program	Soto Mas, Francisco; Iriart, Celia; Pedroncelli, Ronnie; Qualls, Clifford R.; Price, Brittany; Binder, Douglas S.	2019	Population Health Management	22	2	113-119	10.1089/pop.2018.0048	SotoMas 2019	Irrelevant	Excluded by TI/AB review
2009	Dissemination of the CAPABLE Model of Care in a Medicaid Waiver Program to Improve Physical Function	Spoelstra, S. L.; Sikorskii, A.; Gitlin, L. N.; Schueller, M.; Kline, M.; Szanton, S. L.	2019	Journal of the American Geriatrics Society	67	2	363-370	10.1111/jgs.15713	Spoelstra 2019	Irrelevant	Excluded by TI/AB review
2010	Use of social psychology to improve adherence to national bronchiolitis guidelines	Sprecher, E.; Chi, G.; Ozonoff, A.; Cox, J.; Patel, N.; Conroy, K.	2019	Pediatrics	143	1	NA	10.1542/peds.2017-4156	Sprecher 2019	Irrelevant	Excluded by TI/AB review

S4 Table. All studies identified in the literature search

2011	Disaster preparedness among Thai elderly emergency department [illegible]	Sri-On, J.; Vanichkulbodee, A.; Sinsuwan, N.; Rojsaengroeng, R.; Kamsom, A.; Liu, S.	2019	BMC Emergency Medicine	19	1	NA	10.1186/s12873-019-0269-7	Sri-On 2019	Irrelevant	Excluded by TI/AB review
2012	Exploring racial/ethnic disparities in hypertension care among patients served by health centers¬†in the United States	Sripipatana, A.; Pourat, N.; Chen, X.; Zhou, W.; Lu, C.	2019	Journal of Clinical Hypertension	21	4	489-498	10.1111/jch.13504	Sripipatana 2019	Irrelevant	Excluded by TI/AB review
2013	"Just too busy living in the moment and surviving": Barriers to accessing health care for [illegible]	Stajduhar, K. I.; Mollison, A.; Giesbrecht, M.; McNeil, R.; Pauly, B.; Reimer-Kirkham, S.; Dosani, N.; Wallace, B.; Showler, [illegible]	2019	BMC Palliative Care	18	1	NA	10.1186/s12904-019-0396-7	Stajduhar 2019	Irrelevant	Excluded by TI/AB review
2014	Stroke Warning Information and Faster Treatment (SWIFT): Cost-Effectiveness of a [illegible]	Stevens, E. R.; Roberts, E.; Kuczynski, H. C.; Boden-Albala, B.	2019	Value Health	22	11	1240-1247	10.1016/j.jval.2019.06.003	Stevens 2019	Irrelevant	Excluded by TI/AB review

S4 Table. All studies identified in the literature search

2018	Impact of a novel digital medicine program on adherence and utilization in pediatric transplant patients	Sullivan, S.; Tripltt, K. N.; Sacks, C.; Tran, M.; Desai, D. M.	2019	American Journal of Transplantation	19	NA	753	10.1111/ajt.15406	Sullivan 2019	Irrelevant	Excluded by TI/AB review
2019	Application of NICE or SNC guidelines may reduce the need for computerized tomographies in patients with mild traumatic brain [illegible]	Svensson, S.; Vedin, T.; Clausen, L.; Larsson, P. A.; Edelhamre, M.	2019	Scandinavian journal of trauma, resuscitation and emergency medicine	27	1	99	10.1186/s13049-019-0673-8	Svensson 2019	Irrelevant	Excluded by TI/AB review
2020	Improving healthcare utilization following bariatric surgery: Shifting care of nausea, vomiting, and [illegible]	Switzer, N. J.; Dang, J.; Warwar, S.; Noria, S.	2019	Surgical Endoscopy	33	NA	S114	10.1007/s00464-019-06705-1	Switzer 2019	Irrelevant	Excluded by TI/AB review
2021	Geographical Differences in Trigger Factors of Tension-Type Headaches and [illegible]	Tai, Mei-Ling Sharon; Yet, Sharon Xue Er; Lim, Ting Chung; Pow, Zhen Yuan; Goh, Cheng [illegible]	2019	Current Pain & Headache Reports	23	2	N.PAG-N.PAG	10.1007/s11916-019-0760-6	Tai 2019	Irrelevant	Excluded by TI/AB review

S4 Table. All studies identified in the literature search

2022	Impact of a Medication Therapy Management Clinic on Glycosylated Hemoglobin, Blood Pressure, and Resource Utilization	Tilton, J. J.; Edakkunnathu, M. G.; Moran, K. M.; Markel Vaysman, A.; DaPisa, J. L.; Goen, B. M.; Touchette, D. R.	2019	Annals of Pharmacotherapy	53	1	13-20	10.1177/1060028018794860	Tilton 2019	Irrelevant	Excluded by TI/AB review
2023	Federally Qualified Health Center Strategies for Integrating Care with Hospitals and Their Association with Measures of Communication	Timbie, J. W.; Kranz, A. M.; Mahmud, A.; Setodji, C. M.; Damberg, C. L.	2019	Joint Commission Journal on Quality and Patient Safety	45	9	620-628	10.1016/j.jcjq.2019.06.004	Timbie 2019	Irrelevant	Excluded by TI/AB review
2024	Adherence with psychotherapy and treatment outcomes for psychogenic nonepileptic seizures	Tolchin, B.; Dworetzky, B. A.; Martino, S.; Blumenfeld, H.; Hirsch, L. J.; Baslet, G.	2019	Neurology	92	7	E675-E679	10.1212/WNL.0000000000006848	Tolchin 2019	Irrelevant	Excluded by TI/AB review

S4 Table. All studies identified in the literature search

2027	Transportation preferences of patients discharged from the emergency department in the era of ridesharing	Tomar, A.; Ganesh, S. S.; Richards, J. R.	2019	Western Journal of Emergency Medicine	20	4	672-680	10.5811/westjem.2019.5.42762	Tomar 2019	Irrelevant	Excluded by TI/AB review
2028	Individual Characteristics of Resilience are Associated With Lower-Than-Expected Neighborhood Rates of Cardiovascular Disease in Blacks: Results From the	Topel, Matthew L.; Jeong Hwan, Kim; Mujahid, Mahasin S.; Yi-An, Ko; Vaccarino, Viola; Mubasher, Mohamed; Chang, Liu; Dunbar, Sandra; Sims, Mario; Taylor, Herman A.; Quyyumi, Arshed	2019	Journal of the American Heart Association	8	12	11-Jan	10.1161/JAHA.118.011633	Topel 2019	Irrelevant	Excluded by TI/AB review
2030	Association of Primary Care Continuity with Outcomes Following Transition to Adult Care for Adolescents with Severe Mental Illness	Toulany, A.; Stukel, T. A.; Kurdyak, P.; Fu, L.; Guttmann, A.	2019	JAMA Network Open	2	8	NA	10.1001/jamanetworkopen.2019.8415	Toulany 2019	Irrelevant	Excluded by TI/AB review
2031	Culture and Depression: Clinical Considerations for	Trinh, N. H.; Dean, T.	2019	Massachusetts General Hospital Guide to	NA	NA	45-58	NA	Trinh 2019	Irrelevant	Excluded by TI/AB review

S4 Table. All studies identified in the literature search

2032	Safer transitions in the care of the elderly: Identification of essential information in transitional care	Trivedi, S.; Beckett, S.; Dick, A.; Hartmann, R.; Roberts, C.; Lyster, K.; Stempien, J.	2019	Canadian Journal of Emergency Medicine	21	NA	S110-S111	10.1017/cem.2019.320	Trivedi 2019	Irrelevant	Excluded by TI/AB review
2033	Family Caregiver Skills Training to Improve Experiences of Care: a Randomized Clinical Trial	Van Houtven, C. H.; Smith, V. A.; Lindquist, J. H.; Chapman, J. G.; Hendrix, C.; Hastings, S. N.; Oddone, E. Z.; King, H. A.; Shepherd-Banigan, M.; Weinberger, M.	2019	Journal of General Internal Medicine	34	10	2114-2122	10.1007/s11606-019-05209-x	VanHoutven 2019	Irrelevant	Excluded by TI/AB review
2034	EQUIP Emergency: study protocol for an organizational intervention to	Varcoe, Colleen; Bungay, Vicky; Browne, Annette J.; Wilson, Erin; Wathen, C. Nadine;	2019	BMC Health Services Research	19	1	N.PAG-N.PAG	10.1186/s12913-019-4494-2	Varcoe 2019	Irrelevant	Excluded by TI/AB review

S4 Table. All studies identified in the literature search

2035	Exploring the components of an efficacious computer brief intervention for reducing marijuana use among adults [illegible]	Waller, R.; Bonar, E. E.; Fernandez, A. C.; Walton, M. A.; Chermack, S. T.; Cunningham, R. M.; Blow, F. C.	2019	Journal of Substance Abuse Treatment	99	NA	67-72	10.1016/j.jsat.2019.01.014	Waller 2019	Irrelevant	Excluded by TI/AB review
2036	Quality of end-of-life cancer care at minority-serving US cancer centers: A retrospective [illegible]	Wasp, G.; Alam, S. S.; Brooks, G. A.; Khayal, I. S.; Kapadia, N. S.; Carmicheal, D. Q.; [illegible]	2019	Journal of Clinical Oncology	37	NA	NA	10.1200/JCO.2019.37.15-suppl.6507	Wasp 2019	Irrelevant	Excluded by TI/AB review
2037	Combined analysis of two randomized controlled trials of budesonide/formoterol reliever therapy in adults with mild asthma	Weatherall, M.; Holliday, M.; Baggott, C.; Braithwaite, I.; Fingleton, J.; Hardy, J.; Hancox, R. J.; Harrison, T.; Papi, A.; Pavord, I.; [illegible]	2019	Thorax	74	NA	A212	10.1136/thorax-2019-BTSabstracts2019.369	Weatherall 2019	Irrelevant	Excluded by TI/AB review
2038	Variations in Responding to Chest Pain, Ambulance Utilisation and Clinical Outcomes Among 10 Ethnic Groups: Time, [illegible]	Wechkunanukul, K.; Ullah, S.	2019	Heart Lung and Circulation	28	NA	S378	10.1016/j.hlc.2019.06.568	Wechkunanukul 2019	Irrelevant	Excluded by TI/AB review

S4 Table. All studies identified in the literature search

2039	SUN-114 BARRRIERS TO PRE-EMPTIVE KIDNEY	Weststrate, H.; Wong, W.; Ronaldson, J.; Lalji, R.; Dickens, A.;	2019	Kidney International Reports	4	7	S204-S205	10.1016/j.ekir.2019.05.512	Weststrate 2019	Irrelevant	Excluded by TI/AB review
2040	Rehospitalization of advanced cancer patients in the year after diagnosis	Whitney, R. L.; Bell, J.; Tancredi, D. J.; Romano, P. S.; Bold, R. J.; Joseph, J. G.	2019	Journal of Clinical Oncology	34	26	10	10.1200/jco.2016.34.26-suppl.10	Whitney 2019	Irrelevant	Excluded by TI/AB review
2041	Impact of Guideline-Discordant Treatment on Cost and Health Care Utilization in Older Adults with Early-Stage Breast Cancer	Williams, C. P.; Kenzik, K. M.; Azuero, A.; Williams, G. R.; Pisu, M.; Halilova, K. I.; Ingram, S. A.; Yagnik, S. K.; Forero, A.; Bhatia, S.; Rocque, G. B.	2019	Oncologist	24	1	31-37	10.1634/theoncologist.2018-0076	Williams 2019	Irrelevant	Excluded by TI/AB review

S4 Table. All studies identified in the literature search

2042	Failingtodeliver: Improving transitionsofcare for peripartum women with medical comorbidities	Wiltjer, C.; Lora, M. H.	2019	Journal of General Internal Medicine	34	2	S530	10.1007/11606.1525-1497	Wiltjer 2019	Irrelevant	Excluded by TI/AB review
2043	Population health care for children with ongoing conditions	Wolfe, I.; Lemer, C.; Forman, J.; Newham, J.; Lingam, R.	2019	Israel Journal of Health Policy Research	8	NA	NA	10.1186/s13584-019-0336-2	Wolfe 2019	Irrelevant	Excluded by TI/AB review

S4 Table. All studies identified in the literature search

2044	Cyphp evelina london new care model to improve health, healthcare quality, and patterns of service use among children and young people	Wolfe, I.; Lemer, C.; Heys, M.; Ingrassia, A.; Newham, J.; Forman, J.; Satherley, R.; Lingam, R.	2019	Archives of Disease in Childhood	104	NA	A141	10.1136/archdischild-2019-rcpch.334	Wolfe 2019	Irrelevant	Excluded by TI/AB review
2045	Association of Race with Bariatric Surgery Outcomes	Wood, M. H.; Carlin, A. M.; Ghaferi, A. A.; Varban, O. A.; Hawasli, A.; Bonham, A. J.; Birkmeyer, N. J.; Finks, J. F.	2019	JAMA Surgery	154	5	NA	10.1001/jamasurg.2019.0029	Wood 2019	Irrelevant	Excluded by TI/AB review
2046	Patient Dissatisfaction With Primary Care and Nonurgent Emergency Department Use	Xin, H.	2019	The Journal of ambulatory care management	42	4	284-294	10.1097/JAC.0000000000000301	Xin 2019	Irrelevant	Excluded by TI/AB review

S4 Table. All studies identified in the literature search

2047	The impact of depression in cancer patients on healthcare utilization	Ye, S.; Gilbert, A.; Huang, C. H.; Rocque, G. B.	2019	Journal of Clinical Oncology	37	NA	NA	10.1200/JCO.2019.37.27-suppl.243	Ye 2019	Irrelevant	Excluded by TI/AB review
2048	3.1 TOWARDS DEVELOPING A FRAMEWORK FOR ANALYZING HIGH-RISK [illegible]	Yehudian, S.	2019	Journal of the American Academy of Child and Adolescent [illegible]	58	10	S5	10.1016/j.jaac.2019.07.023	Yehudian 2019	Irrelevant	Excluded by TI/AB review
2049	Assessment of left ventricular function after primary percutaneous coronary intervention for acute myocardial [illegible]	Zaheen, K.; Kamal, E.; Goyal, G.; Amiri, M.; Rahman, R.	2019	European Heart Journal, Supplement	21	SUPPL H	H1-H2	10.1093/eurheartj/suz227	Zaheen 2019	Irrelevant	Excluded by TI/AB review
2050	Racial and Ethnic Disparities in Emergency Department Care and Health Outcomes Among Children in the [illegible]	Zhang, X.; Carabello, M.; Hill, T.; He, K.; Friese, C. R.; Mahajan, P.	2019	Frontiers in Pediatrics	7	NA	NA	10.3389/fped.2019.00525	Zhang 2019	Irrelevant	Excluded by TI/AB review

S4 Table. All studies identified in the literature search

2052	Trends in US physician diagnosis of child physical abuse and neglect injuries, 2006-2014	Zins, Z. P.; Wheeler, K. K.; Brink, F.; Armstrong, M.; Shi, J. X.; Groner, J. I.; Xiang, H.	2019	Child Abuse & Neglect	98	NA	NA	10.1016/j.chiabu.2019.104179	Zins 2019	Irrelevant	Excluded by TI/AB review
2053	Effects of intensive primary care on high-need patient experiences: Findings fromaveterans [illegible]	Zulman, D. M.; Chang, E. T.; Wong, A.; Yoon, J.; Stockdale, S. E.; Ong, M.; Rubenstein, L. V.; Asch, S.	2019	Journal of General Internal Medicine	34	2	S205	10.1007/11606.1525-1497	Zulman 2019	Irrelevant	Excluded by TI/AB review
2054	Effects of a Behavioral Health and Chronic Illness Care Intervention on Patient Outcomes in Primary Care Practices in the [illegible]	Zurovac, Jelena; Peterson, G. Greg; Stewart, Kate A.; Kranker, Keith; Wells, KeriAnn; Gilman, Boyd; Blue, Laura; Day, Timothy; Hoag, Sheila; [illegible]	2019	Journal of Health Care for the Poor & Underserved	30	2	702-720	10.1353/hpu.2019.0051	Zurovac 2019	Irrelevant	Excluded by TI/AB review

S4 Table. All studies identified in the literature search

2055	2020_NMA_AnnualMeeting Abstracts	NA	2020	Journal of the National Medical Association	112	5	S1-S54	10.1016/j.jnma.2020.10.005	NA	Irrelevant	Excluded by TI/AB review
2056	Using Design Thinking to Improve Patient-Provider Communication in the Emergency Department	Aaronson, Emily L.; White, Benjamin A.; Black, Lauren; Sonis, Jonathan D.; Mort, Elizabeth A.	2020	Quality management in health care	29	1	30-34	10.1097/QMH.0000000000000239	Aaronson 2020	Irrelevant	Excluded by TI/AB review

S4 Table. All studies identified in the literature search

2057	Methodology and baseline characteristics of a randomized controlled trial testing a health care professional and peer-support program for patients with chronic obstructive pulmonary disease: The BREATHE2 study	Aboumatar, H.; Naqibuddin, M.; Neiman, J.; Saunders, J.; Kim, S.; Chaudhry, H.; Garcia-Morales, E.; Robinson, N.; McBurney, M.; Jager, L.; Ajayi, T.; Bone, L.; Chung, S.; Farrell, B.; Joo Jin, H.; Linnell, J.; Pirfo, M.; Rand, C.; Riley, P.; Salvaterra, C.; Shea, K.; Singh, J.; Wise, R.	2020	Contemporary Clinical Trials	94	NA	NA	10.1016/j.cct.2020.106023	Aboumatar 2020	Irrelevant	Excluded by TI/AB review

S4 Table. All studies identified in the literature search

2058	Simple Care, Concern Refute Perception of Bias that Fuels Lawsuits	Ahc, Media	2020	ED Legal Letter	31	9	3-Jan	NA	Ahc 2020	Irrelevant	Excluded by TI/AB review
2059	Examining potential bias in emergency department care among homeless and non-homeless individuals	Ahmed, H.; Dennis, J.	2020	Academic Emergency Medicine	27	NA	S160	10.1111/acem.13961	Ahmed 2020	Irrelevant	Excluded by TI/AB review
2060	HIV Symposium	Ahonkhai, A. A.; Fields, E. L.; Secord, E. A.; Golden, W. C.; Collins-Ogle, M. D.	2020	Journal of the National Medical Association	112	5	S43-S44	10.1016/j.jnma.2020.09.165	Ahonkhai 2020	Irrelevant	Excluded by TI/AB review

S4 Table. All studies identified in the literature search

2061	Racial Disparities are Present in the Timing of Radiographic Assessment and Surgical Treatment of Hip Fractures	Ali, I.; Vattigunta, S.; Jang, J. M.; Hannan, C. V.; Ahmed, M. S.; Linton, B.; Kantsiper, M. E.; Bansal, A.; Srikumaran, U.	2020	Clinical Orthopaedics and Related Research	478	3	455-461	10.1097/CORR.0000000000001091	Ali 2020	Irrelevant	Excluded by TI/AB review
2064	High risk neighbourhoods: The effect of neighbourhood level factors on cardiac arrest incidence	Allan, Katherine S.; Ray, Joel G.; Gozdyra, Peter; Morrison, Laurie J.; Kiss, Alexander; Buick, Jason E.; Zhan, Cathy C.; Dorian, Paul	2020	Resuscitation	149	NA	100-108	10.1016/j.resuscitation.2020.02.002	Allan 2020	Irrelevant	Excluded by TI/AB review
2066	Immigrant health: Building bridges in medical education	Allport, B.; Norwood, A.; Gray, M.	2020	Journal of General Internal Medicine	35	SUPPL 1	S735	10.1007/s11606-020-05890-3	Allport 2020	Irrelevant	Excluded by TI/AB review

S4 Table. All studies identified in the literature search

2067	Patient Advocates for Low-Income Adults with Moderate to Severe Asthma: A Randomized Clinical Trial	Apter, A. J.; Perez, L.; Han, X.; Ndicu, G.; Localio, A.; Park, H.; Mullen, A. N.; Klusaritz, H.; Rogers, M.; Cidav, Z.; Bryant-Stephens, T.; Bender, B. G.; [illegible]	2020	Journal of Allergy and Clinical Immunology: In Practice	8	10	3466-3473.e11	10.1016/j.jaip.2020.06.058	Apter 2020	Irrelevant	Excluded by TI/AB review
2068	Penile Prosthesis Implantation and Timing Disparities After Radical Prostatectomy: Results From a Statewide Claims Database	Bajic, P.; Patel, P. M.; Nelson, M. H.; Dornbier, R. A.; Kirshenbaum, E. J.; Baker, M. S.; Farooq, A. V.; McVary, K. T.; Gupta, G. N.; Bresler, L.	2020	Journal of Sexual Medicine	17	6	1175-1181	10.1016/j.jsxm.2020.02.022	Bajic 2020	Irrelevant	Excluded by TI/AB review
2069	Effect of an Early Palliative Care Telehealth Intervention vs Usual Care on Patients With [illegible]	Bakitas, Marie A.; Dionne-Odom, J. Nicholas; Ejem, Deborah B.; Wells, Rachel; Azuero, Andres; Stockdill, [illegible]	2020	JAMA Internal Medicine	180	9	1203-1213	10.1001/jamainternmed.2020.2861	Bakitas 2020	Irrelevant	Excluded by TI/AB review
2070	142: Migraines in pregnancy and select adverse pregnancy outcomes	Bandoli, G.; Baer, R. J.; Jelliffe-Pawlowski, L. L.; Chambers, C.	2020	American Journal of Obstetrics and Gynecology	222	1	S106	10.1016/j.ajog.2019.11.158	Bandoli 2020	Irrelevant	Excluded by TI/AB review

S4 Table. All studies identified in the literature search

2071	Reaching health facilities in situations of emergency:	Banke-Thomas, Aduragbemi; Balogun, Mobolanle; Wright, Ololade;	2020	Reproductive Health	17	1	N.PAG-N.PAG	10.1186/s12978-020-00996-7	Banke-Thomas 2020	Irrelevant	Excluded by TI/AB review
2072	ED visits and readmissions after follow-up for mental health hospitalization	Bardach, N. S.; Bardach, N. S.; Doupnik, S. K.; Rodean, J.; Zima, B. T.; Gay, J. C.; Nash, C.; Tanguturi, Y.; Coker, T. R.	2020	Pediatrics	145	6	NA	10.1542/peds.2019-2872	Bardach 2020	Irrelevant	Excluded by TI/AB review
2074	Intersectionality and relational psychoanalysis: New perspectives on race, gender, and sexuality	Belkin, Max; White, Cleonie	2020	NA	NA	NA	NA	10.4324/9780429344312	Belkin 2020	Irrelevant	Excluded by TI/AB review
2075	Improving geriatric trauma care via a multi-disciplinary age-friendly care pathway	Bharija, A.; Mesias, M.; Storr-Street, N.; Ellsworth, A. M.; Brown, A.; Cheng, N.; Knowlton, L.; Tennakoon, L.; Martin, M.; Lu, A.; Staudemayer, K.	2020	Journal of the American Geriatrics Society	68	SUPPL 1	S16-S17	10.1111/jgs.16431	Bharija 2020	Irrelevant	Excluded by TI/AB review

S4 Table. All studies identified in the literature search

2076	Factors associated with clinical care for food allergy among Medicaid enrolled U.S. children	Bilaver, L.; Fierstein, J.; Kanaley, M.; Gupta, R.	2020	Journal of Allergy and Clinical Immunolog y	145	2	AB183	10.1016/j.ja ci.2019.12.3 16	Bilaver 2020	Irrelevant	Excluded by TI/AB review
2077	Association of serum phosphate and mortality in patients with sepsis	Black, L. P.; Mohseni, M.; Shirazi, E.; Smotherman, C.; Norse, A.; DeVos, E. L.; Fernandez, R.; Sheele, J. M.; Guirgis, F. W.	2020	Academic Emergency Medicine	27	NA	S74	10.1111/ace m.13961	Black 2020	Irrelevant	Excluded by TI/AB review

S4 Table. All studies identified in the literature search

2078	Predictors of linkage to care for a nontargeted emergency department hepatitis C screening program	Blackwell, J. A.; Rodgers, J. B.; Franco, R. A.; Cofield, S. S.; Walter, L. A.; Galbraith, J. W.; Hess, E. P.	2020	American Journal of Emergency Medicine	38	7	1396-1401	10.1016/j.ajem.2019.11.034	Blackwell 2020	Irrelevant	Excluded by TI/AB review
2079	Multicenter cohort study of age-related timeliness of treatment in patients with st-elevation myocardial infarction	Bloos, S. M.; Kaur, K.; Lang, K.; Gavin, N.; Mills, A. M.; Baugh, C. W.; Patterson, B. W.; Podolsky, S.; Salazar, G.; Mumma, B. E.; Tanski, M.; Hadley, K.; Roumie, C.; McNaughton, C.; Yiadom, M. Y. A. B.	2020	Academic Emergency Medicine	27	NA	S128	10.1111/acem.13961	Bloos 2020	Irrelevant	Excluded by TI/AB review
2080	Abstract: Self-advocacy in the diagnostic process among emergency department patients: a qualitative study	Bondal, P. E. C.; Villanueva, M.; Gleason, K. T.	2020	Circulation: Cardiovascular Quality and Outcomes	13	SUPPL 1	NA	NA	Bondal 2020	Irrelevant	Excluded by TI/AB review

S4 Table. All studies identified in the literature search

2081	Healthcare Access, Quality, and Satisfaction Among Albanian Immigrants Using the Emergency Department in Northern Greece	Boutziona, I.; Papanikolaou, D.; Sokolakis, I.; Mytilekas, K. V.; Apostolidis, A.	2020	Journal of immigrant and minority health	22	3	512-525	10.1007/s10903-020-00983-x	Boutziona 2020	Irrelevant	Excluded by TI/AB review
2083	Coordinated multicenter care after surgical resection of rectal cancer at high-volume centers is non-inferior to single-center care	Bruce, A. N.; Song, Y.; Paulson, E. C.; Roses, R. E.; Kelz, R. R.; Fraker, D. L.; Miura, J. T.; Karakousis, G. C.	2020	Annals of Surgical Oncology	27	NA	S42-S43	10.1245/s10434-020-08278-z	Bruce 2020	Irrelevant	Excluded by TI/AB review

S4 Table. All studies identified in the literature search

2084	Pediatric emergency care in a low-income country: Characteristics and outcomes of presentations to a tertiary-care emergency department in [illegible]	Brugnolaro, V.; Fovino, L. N.; Calgaro, S.; Putoto, G.; Muhelo, A. R.; Gregori, D.; Azzolina, D.; Bressan, S.; da Dalt, L.	2020	PLoS One	15	#####	NA	10.1371/journal.pone.0241209	Brugnolaro 2020	Irrelevant	Excluded by TI/AB review
2085	Design of a home-based intervention for Houston-area African-American adults with [illegible]	Bruhl, R. J.; Perkison, W. B.; Hanania, N. A.; McNeill, L. H.; Oluyomi, A. O.; [illegible]	2020	Contemporary Clinical Trials	91	NA	NA	10.1016/j.cct.2020.105977	Bruhl 2020	Irrelevant	Excluded by TI/AB review

S4 Table. All studies identified in the literature search

2087	Pediatric orbital floor fractures and the oculocardiac reflex: Experience from a level I trauma center	Buck, L. S.; Stockton, S.; Spankovich, C.; Jordan, J. R.	2020	American Journal of Otolaryngology - Head and Neck Medicine and Surgery	41	4	NA	10.1016/j.amjoto.2020.102553	Buck 2020	Irrelevant	Excluded by TI/AB review
2088	Low uptake of rapid diagnostic tests for respiratory tract infections in an urban safety net [illegible]	Burrowes, S. A. B.; Rader, A.; Ni, P.; Drainoni, M. L.; Barlam, T. F.	2020	Open Forum Infectious Diseases	7	3	NA	10.1093/ofid/ofaa057	Burrowes 2020	Irrelevant	Excluded by TI/AB review

S4 Table. All studies identified in the literature search

2089	Medicaid Utilization and Spending among Homeless Adults in New Jersey: Implications for Medicaid-Funded Tenancy Support Services	Cantor, J. C.; Chakravarty, S.; Nova, J.; Kelly, T.; Delia, D.; Tiderington, E.; Brown, R. W.	2020	Milbank Quarterly	98	1	106-130	10.1111/1468-0009.12446	Cantor 2020	Irrelevant	Excluded by TI/AB review

S4 Table. All studies identified in the literature search

2090	Association between ambulatory status and call bell use in hospitalized patients‚ÄîA retrospective cohort study	Capo‚ÄêLugo, Carmen E.; Shumock, Kara; Young, Daniel L.; Klein, Lisa; Cassell, Andre; Cvach, Maria; Lavezza, Annette; Friedman, Michael; Bhatia, Elys; Brotman, Daniel J.; Hoyer, Erik H.	2020	Journal of Nursing Management (John Wiley & Sons, Inc.)	28	1	54-62	10.1111/jonm.12888	Capo‚Äê Lugo 2020	Irrelevant	Excluded by TI/AB review
2091	Comparing the Prevalence of Poor Sleep and Stress Metrics in Basic versus Advanced Life Support [illegible]	Cash, Rebecca E.; Anderson, Sarah E.; Lancaster, Kathryn E.; Lu, Bo; Rivard, Madison K.; Camargo, Carlos A.; [illegible]	2020	Prehospital Emergency Care	24	5	644-656	10.1080/10903127.2020.1758259	Cash 2020	Irrelevant	Excluded by TI/AB review

S4 Table. All studies identified in the literature search

2092	Evaluating hepatitiss C exposure and outcomes in young adults seen in the emergency department	Cave, B. A.; Ross, A.; Pasquenza, N.; Laun, K.; Melendez, A.; Parra-Flores, F.; Higdon, K.; Chesman, A.; Sheahan, B.; Shreffler, J.	2020	Hepatology	72	1 SUPPL	586A-587A	10.1002/hep.31579	Cave 2020	Irrelevant	Excluded by TI/AB review
2093	Emergency Department Quality of Care for Sickle Cell Disease in Ontario, Canada: A Population-Based Matched Cohort Study	Chan, D.; Kouyoumdjian, F.; Athale, U.; Pendergrast, J.; Verhovsek, M.	2020	Blood	136	NA	38-39	10.1182/blood-2020-143472	Chan 2020	Irrelevant	Excluded by TI/AB review

S4 Table. All studies identified in the literature search

2095	Development and implementation of a resident-led health equity curriculum	Chary, A.; Molina, M.; Dadabhoy, F. Z.; Landry, A.; Samuels-Kalow, M. E.; Cleveland Manchanda, E. C.	2020	Academic Emergency Medicine	27	NA	S324	10.1111/acem.13961	Chary 2020	Irrelevant	Excluded by TI/AB review
2096	Relationships Between Race/Ethnicity and Health Care Utilization Among Older Post-Acute Home Health Care Patients	Chase, Jo-Ana D.; Russell, David; Huang, Liming; Hanlon, Alexandra; O'Connor, Melissa; Bowles, Kathryn H.	2020	Journal of Applied Gerontology	39	2	201-213	10.1177/0733464818758453	Chase 2020	Irrelevant	Excluded by TI/AB review

S4 Table. All studies identified in the literature search

2098	High Satisfaction with Tele-medicine in a New York City Clinic	Chen, T.; Guo, C.; Tang, W.; Khalili, L.; Askanase, A.	2020	Arthritis and Rheumatology	72	SUPPL 10	1196-1198	10.1002/art.41538	Chen 2020	Irrelevant	Excluded by TI/AB review
2099	Strengthening the U. S. Medication Safety Net by Connecting Abundance to Need	Chima, C. C.; Bruce, M. A.; Pendergrass, D. B.; Thorpe, R. J.; Ward, L. M.; Blackburn, H. F.; Palombo, C. F.; Beech, B. M.	2020	Journal of Health Care for the Poor and Underserved	31	2	503-518	10.1353/hpu.2020.0038	Chima 2020	Irrelevant	Excluded by TI/AB review
2100	Rapid Administration of Ketamine for Abscess Drainage in Children-A Dose Finding Study	Chinta, S. S.; Schrock, C. R.; McAllister, J. D.; Jaffe, D. M.; Liu, J.; Kennedy, R. M.	2020	Pediatric Emergency Care	36	12	E671-E676	10.1097/PEC.0000000000002281	Chinta 2020	Irrelevant	Excluded by TI/AB review

S4 Table. All studies identified in the literature search

2101	Building RAFT: Trafficking Screening Tool Derivation and Validation Methods	Chisolm-Straker, M.; Singer, E.; Rothman, E. F.; Clesca, C.; Strong, D.; Loo, G. T.; Sze, J. J.; d,ÄôEtienne, J. P.; Alanis, N.; Richardson, L. D.	2020	Academic Emergency Medicine	27	4	297-304	10.1111/acem.13888	Chisolm-Straker 2020	Irrelevant	Excluded by TI/AB review
2102	Hepatitis C virus infection characteristics and treatment outcomes in Canadian immigrants	Cooper, Curtis L.; Read, Daniel; Vachon, Marie-Louise; Conway, Brian; Wong, Alexander; Ramji, Alnoor; Borgia, Sergio; Tam, Ed; Barrett, Lisa; Smyth, Dan; Feld, Jordan J.; Lee, Sam	2020	BMC Public Health	20	1	7-Jan	10.1186/s12889-020-09464-0	Cooper 2020	Irrelevant	Excluded by TI/AB review

S4 Table. All studies identified in the literature search

2103	125 Linkage Outcomes for HIV/HCV Co-Infected and HCV Mono-Infected Patients Participating in an Emergency Department Screening Program	Cowan, E.; Hardardt, J.; Brandspiegel, S.; Calderon, Y.	2020	Annals of Emergency Medicine	76	4	S49	10.1016/j.annemergmed.2020.09.136	Cowan 2020	Irrelevant	Excluded by TI/AB review
2104	Paediatric emergency department utilisation rates and maternal migration status in the Born in Bradford cohort: A cross-sectional study	Cred√©, S. H.; Mason, S.; Such, E.; Jacques, R. M.	2020	PLoS Medicine	17	3	e1003043	10.1371/journal.pmed.1003043	Cred√© 2020	Irrelevant	Excluded by TI/AB review

S4 Table. All studies identified in the literature search

2105	Adverse childhood experiences are associated with at-	Cunradi, C. B.; Caetano, R.; Alter, H. J.; Ponicki, W. R.	2020	American Journal of Drug and	46	6	739-748	10.1080/00952990.2020.1823989	Cunradi 2020	Irrelevant	Excluded by TI/AB review
2108	A Patient-Centered Asthma Management Communication Intervention for Rural Latino Children: Protocol for a Waiting-List Randomized Controlled Trial	Dawson, R. M.; Heiney, S. P.; Messias, D. H.; Ownby, D.	2020	Jmir Research Protocols	9	12	NA	10.2196/18977	Dawson 2020	Irrelevant	Excluded by TI/AB review
2109	Telemedicine optimizes early post-operative follow up after synthetic mid-urethral sling (MUS): A randomized multi- [illegible]	Derisavifard, S.; Rueb, J.; Kocher, N.; Giusto, L.; Zahner, P.; Dielubanza, E.; Luo, D.; Li, J.; De Jesus Moreira, R.; Gomelsky, A.; Balzarro, M.; [illegible]	2020	Journal of Urology	203	NA	e444-e445	10.1097/JU.0000000000000869.017	Derisavifard 2020	Irrelevant	Excluded by TI/AB review

S4 Table. All studies identified in the literature search

2110	Telemedicine optimizes early Postoperative Follow up after synthetic mid-urethral sling (MUS): A [illegible]	Derisavifard, S.; Rueb, J.; Kocher, N.; Giusto, L.; Zahner, P.; Luo, D.; Dielubanza, E.; Li, J.; De Jesus Moreira, R.; Gomelsky, A.; [illegible]	2020	Neurourology and Urodynamics	39	NA	S114-S115	10.1002/nau.24307	Derisavifard 2020	Irrelevant	Excluded by TI/AB review
2111	Insurance Does Not Affect Adverse Events While Awaiting Surgery for Ankle Trauma in One System	Dobbins, Adam B.; Krumme, John; Gaddis, Monica; Park, Shin Hye; Varghese, Manna; Brancato, Michael R.; Shaw, Christopher M.; Wambach, Karen	2020	Western Journal of Emergency Medicine: Integrating Emergency Care with Population Health	21	5	1242-1248	10.5811/westjem.2020.5.46861	Dobbins 2020	Irrelevant	Excluded by TI/AB review
2112	131 A Novel Mobile Integrated Health Program for COVID-19 Response	Dorner, S.; Carlson, L. C.; DeSharone, A.; Thompson, R.; Pu, C. T.; Mark, E.; Goldberg, S. A.	2020	Annals of Emergency Medicine	76	4	S52	10.1016/j.annemergmed.2020.09.142	Dorner 2020	Irrelevant	Excluded by TI/AB review
2113	Derivation and Validation of a Clinical Decision Guideline for Influenza Testing in 4 US Emergency [illegible]	Dugas, A. F.; Hsieh, Y. H.; Lovecchio, F.; Moran, G. J.; Steele, M. T.; Talan, D. A.; Rothman, R. E.; Stubbs, A.; Kemble, L.; Beckham, D.; [illegible]	2020	Clinical Infectious Diseases	70	1	49-58	10.1093/cid/ciz171	Dugas 2020	Irrelevant	Excluded by TI/AB review

S4 Table. All studies identified in the literature search

2114	Metal detectors improve perception of safety in the emergency department	Easter, J.; Kehr, W.; Fiorino, D.	2020	Academic Emergency Medicine	27	NA	S132	10.1111/acem.13961	Easter 2020	Irrelevant	Excluded by TI/AB review
2116	Type-II myocardial infarction and chronic myocardial injury rates, invasive [illegible]	Etaher, A.; Gibbs, O. J.; Saad, Y. M.; Frost, S.; Nguyen, T. L.; Ferguson, I.; Juergens, C. P.; Chew, D.; French, J. [illegible]	2020	European Heart Journal - Quality of Care and Clinical [illegible]	6	1	41-48	10.1093/ehjqcco/qcz019	Etaher 2020	Irrelevant	Excluded by TI/AB review
2117	A community-based asthma program: Study design and methods of RVA Breathes	Everhart, R. S.; Mazzeo, S. E.; Corona, R.; Holder, R. L.; Thacker, L. R.; Schechter, M. S.	2020	Contemporary Clinical Trials	97	NA	NA	10.1016/j.cct.2020.106121	Everhart 2020	Irrelevant	Excluded by TI/AB review

S4 Table. All studies identified in the literature search

2118	Hospital-Based Resource Use and Costs Among Patients With Idiopathic Pulmonary Fibrosis Enrolled in the Idiopathic Pulmonary Fibrosis Prospective	Fan, Y.; Bender, S. D.; Conoscenti, C. S.; Davidson-Ray, L.; Cowper, P. A.; Palmer, S. M.; de Andrade, J. A.	2020	Chest	157	6	1522-1530	10.1016/j.chest.2019.12.041	Fan 2020	Irrelevant	Excluded by TI/AB review
2119	Patient characteristics associated with improvements in asthma control and reduction in emergency department visits for older adults with asthma	Federman, A. D.; Thanik, E.; O,ÄôConor, R.; Arora, A.; Wolf, M. S.; Wisnivesky, J. P.	2020	Journal of Asthma	NA	NA	NA	10.1080/02770903.2020.1805753	Federman 2020	Irrelevant	Excluded by TI/AB review
2120	THE TIMING OF FIRST HELP-SEEKING ATTEMPT IN FIRST EPISODE PSYCHOSIS [illegible]	Ferrara, Maria; Guloksuz, Sinan; Mathis, Walter; Raballo, Andrea; Fangyong, Li; I. Hsin Lin; Syed, Sumaiyah; [illegible]	2020	Schizophrenia Bulletin	46	NA	S324-S325	10.1093/schbul/sbaa029.802	Ferrara 2020	Irrelevant	Excluded by TI/AB review

S4 Table. All studies identified in the literature search

2121	The timing of first help-seeking attempt in first episode psychosis can lead to aversive pathways to care. results from the step-ed study	Ferrara, M.; Guloksuz, S.; Mathis, W.; Raballo, A.; Li, F.; Lin, I. H.; Syed, S.; Gallagher, K.; Woods, S.; Tek, C.; Srihari, V.	2020	Schizophrenia Bulletin	46	NA	S324-S325	NA	Ferrara 2020	Irrelevant	Excluded by TI/AB review
2123	Outcomes of depressed patients with and without antidepressant prescription	Francis, A.; Krallman, R. H.; Feldeisen, D.; Montgomery, D. G.; Kline-Rogers, E. M.; Eagle, K.; Bumpus, S.	2020	Circulation: Cardiovascular Quality and Outcomes	13	SUPPL 1	NA	NA	Francis 2020	Irrelevant	Excluded by TI/AB review
2124	"Your Finger or Mine?"-Patient Preferences in the Collection of Fecal Occult Blood Testing in the Emergency Department	Freeman, P.; Couperus, K.; Walsh, R.; Ward, M. J.; McNaughton, C. D.; Bothwell, J.	2020	Military medicine	185	#####	e1393-e1396	10.1093/milmed/usz429	Freeman 2020	Irrelevant	Excluded by TI/AB review

S4 Table. All studies identified in the literature search

2125	Medication Adherence, Health Care Utilization, and Spending Among Privately Insured Adults With Chronic Conditions in the [illegible]	Gillespie, C. W.; Morin, P. E.; Tucker, J. M.; Purvis, L.	2020	American Journal of Medicine	133	6	690-704.e19	10.1016/j.amjmed.2019.12.021	Gillespie 2020	Irrelevant	Excluded by TI/AB review
2126	DOES THE 2018 VA MISSION ACT IMPACT QUALITY OF CARE? AN EXAMPLE OF SHARED DECISION MAKING: [illegible]	Gokhale, S.; Desai, B.; Twing, A.; Shroff, A. R.	2020	Journal of the American College of Cardiology	75	11	3526	10.1016/S0735-1097(20)34153-X	Gokhale 2020	Irrelevant	Excluded by TI/AB review
2127	CLINICAL DECISION MAKING IN OLDER ADULTS WITH COVID-19 IN DEVELOPING [illegible]	G√≥mez-Moreno, C.; Hern√°ndez-Ruiz, V.; Hern√°ndez-Gilsoul, T.; Avila-Funes, J. A.	2020	Revista de investigacion clinica; organo del Hospital de Enfermedades de la [illegible]	72	3	127-134	10.24875/RIC.20000131	G√≥mez-Moreno 2020	Irrelevant	Excluded by TI/AB review

S4 Table. All studies identified in the literature search

2128	Characterisation of 22445 patients attending UK emergency departments with suspected COVID-19 infection: Observational cohort study	Goodacre, S.; Thomas, B.; Lee, E.; Sutton, L.; Loban, A.; Waterhouse, S.; Simmonds, R.; Biggs, K.; Marincowitz, C.; Schutter, J.; Connelly, S.; Sheldon, E.; Hall, J.; Young, E.; Bentley, A.; Challen, K.; Fitzsimmons, C.; Harris, T.; Lecky, F.; Lee, A.; Maconochie, I.; Walter, D.	2020	PLoS One	15	#####	NA	10.1371/journal.pone.0240206	Goodacre 2020	Irrelevant	Excluded by TI/AB review
2129	Correctional health elective	Grassini, M.; Schneberk, T.	2020	Academic Emergency Medicine	27	NA	S326-S327	10.1111/acem.13961	Grassini 2020	Irrelevant	Excluded by TI/AB review

S4 Table. All studies identified in the literature search

2130	486 IMPROVING ACCESS TO CARE REDUCES NEED FOR SURGERY IN IBD PATIENTS REQUIRING BIOLOGIC [illegible]	Gu, P.; Gilman, A. J.; Chang, C.; Moss, E.; Fudman, D.; Kilgore, P. C.; Cvek, U.; Trutschl, M.; Alexander, J. S.; Burstein, E.; Boktor, M.	2020	Gastroenterology	158	6	S-96	10.1016/S0016-5085(20)30922-7	Gu 2020	Irrelevant	Excluded by TI/AB review
2131	Utilizing YouTube as platform for psychiatric emergency patient outreach in Chinese Americans	Guo, J. Z.; Chong, K. P. L.; Woo, B. K. P.	2020	Asian Journal of Psychiatry	50	NA	NA	10.1016/j.ajp.2020.101960	Guo 2020	Irrelevant	Excluded by TI/AB review
2132	Association of Billed Advance Care Planning with End-of-Life Care Intensity for 2017 Medicare Decedents	Gupta, A.; Jin, G.; Reich, A.; Prigerson, H. G.; Ladin, K.; Kim, D.; Ashana, D. C.; Cooper, Z.; Halpern, S. D.; Weissman, J. S.	2020	Journal of the American Geriatrics Society	68	9	1947-1953	10.1111/jgs.16683	Gupta 2020	Irrelevant	Excluded by TI/AB review

S4 Table. All studies identified in the literature search

2133	Does healthcare inequity reflect variations in peoples' abilities to access healthcare? Results from a multi-jurisdictional interventional [illegible]	Haggerty, J.; Levesque, J. F.; Harris, M.; Scott, C.; Dahrouge, S.; Lewis, V.; Dionne, E.; Stocks, N.; Russell, G.	2020	International Journal for Equity in Health	19	1	NA	10.1186/s12939-020-01281-6	Haggerty 2020	Irrelevant	Excluded by TI/AB review
2134	Assault-related, penetrating trauma and gunshot wound recidivism in New Orleans	Hammack, Amanda Yvonne	2020	NA	81	NA	NA	NA	Hammack 2020	Irrelevant	Excluded by TI/AB review
2135	Impact of Massachusetts' ambulance diversion ban on ED destination patterns by race/ethnicity	Hanchate, A. D.; Paasche-Orlow, M.; Baker, W. E.; Lin, M. Y.; Feldman, J.	2020	Journal of General Internal Medicine	35	SUPPL 1	S163-S164	10.1007/s11606-020-05890-3	Hanchate 2020	Irrelevant	Excluded by TI/AB review
2136	Association of Adherence to Weight Telemonitoring with Health Care Use and Death: A Secondary [illegible]	Haynes, S. C.; Tancredi, D. J.; Tong, K.; Hoch, J. S.; Ong, M. K.; Ganiats, T. G.; Evangelista, L. S.; Black, J. T.; [illegible]	2020	JAMA Network Open	3	7	NA	10.1001/jamanetworkopen.2020.10174	Haynes 2020	Irrelevant	Excluded by TI/AB review

S4 Table. All studies identified in the literature search

2137	Evaluating the asthmaxcel mobile application regarding asthma knowledge and clinical outcomes	Hsia, B. C.; Wu, S.; Mowrey, W. B.; Jariwala, S. P.	2020	Respiratory Care	65	8	1112-1119	10.4187/respcare.07550	Hsia 2020	Irrelevant	Excluded by TI/AB review
2138	Routine screening of emergency-admission patients at risk of chronic hepatitis identifies hepatitis B cases and links patients into appropriate [illegible]	Jacob, R.; Prince, D.; Pipicella, J.; Fraser, M.; Alvaro, F.; Maley, M.; Middleton, P.; Foo, H.; Davison, S.; Levy, M.	2020	Journal of Gastroenterology and Hepatology (Australia)	35	SUPPL 1	89	10.1111/jgh.15269	Jacob 2020	Irrelevant	Excluded by TI/AB review
2139	Routine screening of emergency admissions at risk of chronic hepatitis (SEARCH) identifies hepatitis B cases and links [illegible]	Jacob, R.; Prince, D. S.; Pipicella, J. L.; Fraser, M.; Alvaro, F.; Maley, M.; Foo, H.; Middleton, P. M.; Davison, S. A.; Levy, M. T.	2020	Hepatology	72	1 SUPPL	468A-469A	10.1002/hep.31579	Jacob 2020	Irrelevant	Excluded by TI/AB review

S4 Table. All studies identified in the literature search

2141	Protocol for Take-home naloxone in Multicentre Emergency (TIME) settings: feasibility study	Jones, M.; Bell, F.; Benger, J.; Black, S.; Buykx, P.; Dixon, S.; Driscoll, T.; Evans, B.; Edwards, A.; Fuller, G.; Goodacre, S.; Hoskins, R.; Hughes, J.; John, A.; Jones, J.; Moore, C.; Sampson, F.; Watkins, A.; Snooks, H.	2020	Pilot and Feasibility Studies	6	1	NA	10.1186/s40814-020-00626-w	Jones 2020	Irrelevant	Excluded by TI/AB review
2142	Guideline-informed care among medicaid-enrolled children with food allergy	Kanaley, M. K.; Dyer, A. A.; Negris, O. R.; Fierstein, J. L.; Ciaccio, C. E.; Gupta, R. S.; Bilaver, L. A.	2020	American Journal of Managed Care	26	12	505-512	10.37765/ajmc.2020.88538	Kanaley 2020	Irrelevant	Excluded by TI/AB review

S4 Table. All studies identified in the literature search

2143	Perceptions of US Adolescents and Adults With Sickle Cell Disease on Their Quality of Care	Kanter, J.; Gibson, R.; Lawrence, R. H.; Smeltzer, M. P.; Pugh, N. L.; Glassberg, J.; Masese, R. V.; King, A. A.; Calhoun, C.; Hankins, J. S.; [illegible]	2020	JAMA Netw Open	3	5	e206016	10.1001/jamanetworkopen.2020.6016	Kanter 2020	Irrelevant	Excluded by TI/AB review
2147	Emergency department use at the end of life in elderly patients with gastrointestinal malignancies and [illegible]	Kashyap, M.; Harris, J. P.; Chang, D. T.; Pollom, E. L.	2020	Journal of Clinical Oncology	38	4	NA	10.1200/JCO.2020.38.4_suppl.811	Kashyap 2020	Irrelevant	Excluded by TI/AB review
2148	Association Between Patients,Äô Self-Reported Gaps in Care Coordination and Preventable Adverse [illegible]	Kern, L. M.; Reshetnyak, E.; Colantonio, L. D.; Muntner, P. M.; Rhodes, J. D.; Casalino, L. P.; Rajan, M.; Pesko, [illegible]	2020	Journal of General Internal Medicine	35	12	3517-3524	10.1007/s11606-020-06047-y	Kern 2020	Irrelevant	Excluded by TI/AB review
2149	Postoperative Emergency Department Visits after Urinary Stone Surgery: [illegible]	Khanna, A.; Fedrigon, D.; Monga, M.; Gao, T.; Schold, J.; Abouassaly, R.	2020	Journal of Endourology	34	1	93-98	10.1089/end.2019.0399	Khanna 2020	Irrelevant	Excluded by TI/AB review

S4 Table. All studies identified in the literature search

2150	Incidence of Treatment for Opioid Use Disorder following Nonfatal Overdose in Commercially Insured Patients	Kilaru, A. S.; Kilaru, A. S.; Xiong, A.; Lowenstein, M.; Meisel, Z. F.; Perrone, J.; Khatri, U.; Mitra, N.; Delgado, M. K.	2020	JAMA Network Open	3	5	NA	10.1001/jamanetworkopen.2020.5852	Kilaru 2020	Irrelevant	Excluded by TI/AB review
2151	Treatment for opioid use disorder following nonfatal overdose in patients with commercial insurance	Kilaru, A. S.; Perrone, J.; Meisel, Z.; Delgado, M.	2020	Academic Emergency Medicine	27	NA	S135	10.1111/acem.13961	Kilaru 2020	Irrelevant	Excluded by TI/AB review
2152	Incidence of Treatment for Opioid Use Disorder Following Nonfatal Overdose in Commercially Insured Patients...The [illegible]	Kilaru, Austin S.; Xiong, Aria; Lowenstein, Margaret; Meisel, Zachary F.; Perrone, Jeanmarie; Khatri, Utsha; Mitra, Nandita; Delgado, M. Kit	2020	JAMA Network Open	3	5	e205852-e205852	10.1001/jamanetworkopen.2020.5852	Kilaru 2020	Irrelevant	Excluded by TI/AB review

S4 Table. All studies identified in the literature search

2153	Comprehensive and collaborative pharmacist transitions of care service for underserved patients with chronic obstructive pulmonary disease	Kim, J.; Lin, A.; Absher, R.; Makhlouf, T.; Wells, C.	2020	Chronic Obstructive Pulmonary Diseases	8	1	NA	10.15326/JCOPDF.2019.0175	Kim 2020	Irrelevant	Excluded by TI/AB review
2154	Multiple Stakeholder Perspectives on School-Based Responses to Student Suicide Risk in a Diverse Public School District	Kodish, T.; Kim, J. J.; Le, K.; Yu, S. H.; Bear, L.; Lau, A. S.	2020	School Mental Health	12	2	336-352	10.1007/s12310-019-09354-w	Kodish 2020	Irrelevant	Excluded by TI/AB review

S4 Table. All studies identified in the literature search

2155	Health care spending and use among people experiencing unstable housing in the era of accountable care organizations	Koh, K. A.; Racine, M.; Gaeta, J. M.; Goldie, J.; Martin, D. P.; Bock, B.; Takach, M.; O‚Äôconnell, J. J.; Song, Z.	2020	Health Affairs	39	2	214-223	10.1377/hlthaff.2019.00687	Koh 2020	Irrelevant	Excluded by TI/AB review
2156	Health system affiliation of physician organizations and quality of care for Medicare beneficiaries who have high needs	Kranz, A. M.; DeYoreo, M.; Eshete-Roesler, B.; Damberg, C. L.; Totten, M.; Escarce, J. J.; Timbie, J. W.	2020	Health Services Research	55	S3	1118-1128	10.1111/1475-6773.13570	Kranz 2020	Irrelevant	Excluded by TI/AB review

S4 Table. All studies identified in the literature search

2157	Factors related to advance care planning among older African American women: Age, medication, and acute care visits	Kwak, J.; Ellis, J. L.	2020	Palliative & Supportive Care	18	4	413-418	10.1017/S1478951519001007	Kwak 2020	Irrelevant	Excluded by TI/AB review
2158	Outcome of pediatric emergency mental health visits: Incidence and timing of suicide	Lee, J.; Black, T.; Meckler, G.; Doan, Q.	2020	Canadian Journal of Emergency Medicine	22	3	321-330	10.1017/cem.2019.470	Lee 2020	Irrelevant	Excluded by TI/AB review

S4 Table. All studies identified in the literature search

2159	Factors associated with successful mentor matching in an intervention study of youth violence (3372700)	Lennon, T.; Cheng, T.; Johnson, S. L.; Jones, V.; Fein, J.; Ryan, L.	2020	Journal of Investigative Medicine	68	4	912	10.1136/jim-2020-ERM.11	Lennon 2020	Irrelevant	Excluded by TI/AB review
2160	The patient-centered oncology care on health care utilization and cost: A systematic review and meta-analysis	Liang, H.; Tao, L.; Ford, E. W.; Beydoun, M. A.; Eid, S. M.	2020	Health care management review	45	4	364-376	10.1097/HMR.0000000000000226	Liang 2020	Irrelevant	Excluded by TI/AB review

S4 Table. All studies identified in the literature search

2161	The association between Asian patient race/ethnicity and lower satisfaction scores	Liao, L.; Chung, S.; Altamirano, J.; Garcia, L.; Fassiotto, M.; Maldonado, B.; Heidenreich, P.; Palaniappan, L.	2020	BMC Health Services Research	20	1	NA	10.1186/s12913-020-05534-6	Liao 2020	Irrelevant	Excluded by TI/AB review
2162	Race-related differences in acute pain complaints among inner-city women:	Lillis, T. A.; Burns, J.; Aranda, F.; Burgess, H. J.; Purim-Shem-Tov, Y. A.; Bruehl, S.; Beckham,	2020	J Behav Med	43	5	791-806	10.1007/s10865-019-00123-3	Lillis 2020	Irrelevant	Excluded by TI/AB review
2167	Association between linguistic acculturation and cardiovascular medication	Liyanage-Don, N.; Cornelius, T.; Romero, E.; Alcantara, C.; Umland, R.; Ye, S.;	2020	Journal of General Internal Medicine	35	SUPPL 1	S33	10.1007/s11606-020-05890-3	Liyanage-Don 2020	Irrelevant	Excluded by TI/AB review
2169	Prompt Outpatient Care for Older Adults Discharged from the Emergency Department Reduces Recidivism	Magidson, P. D.; Huang, J.; Levitan, E. B.; Westfall, A. O.; Sheehan, O. C.; Roth, D. L.	2020	Western Journal of Emergency Medicine	21	6	198-204	10.5811/WESTJEM.2020.8.47276	Magidson 2020	Irrelevant	Excluded by TI/AB review

S4 Table. All studies identified in the literature search

2170	Emergency Department Presentation After Total Hip and Knee Arthroplasty: A Systematic Review	Maldonado-Rodriguez, N.; Ekhtiari, S.; Khan, M. M.; Ravi, B.; Gandhi, R.; Veillette, C.; Leroux, T.	2020	Journal of Arthroplasty	35	10	3038-3045.e1	10.1016/j.arth.2020.05.022	Maldonado-Rodriguez 2020	Irrelevant	Excluded by TI/AB review
2171	Substance use disorder related social work referral among emergency department patients	Malone, N. C.; Landry, A. M.; Grossestreuer, A. V.	2020	Academic Emergency Medicine	27	NA	S292	10.1111/acem.13961	Malone 2020	Irrelevant	Excluded by TI/AB review
2172	Does a solely spanish-speaking patient population have discrepancies in the emergency [illegible]	Mar√≠n L√≥pez, A. R.; Lugo-Enriquez, K.; Camacho-Acevedo, L.; Adewale, A.; Mora-[illegible]	2020	Academic Emergency Medicine	27	NA	S132	10.1111/acem.13961	Mar√≠nL√≥pez 2020	Irrelevant	Excluded by TI/AB review

S4 Table. All studies identified in the literature search

2173	Home Visits for Children With Asthma Reduce Medicaid Costs	Marshall, E. T.; Guo, J.; Flood, E.; Sandel, M. T.; Sadof, M. D.; Zotter, J. M.	2020	Preventing chronic disease	17	NA	E11	10.5888/pcd17.190288	Marshall 2020	Irrelevant	Excluded by TI/AB review
2174	Primary medication non-adherence at Counties Manukau Health Emergency Department (CMH-[illegible]	Martini, N. D.; Van Der Werf, B.; Bassett-Clarke, D.	2020	BMJ Open	10	7	NA	10.1136/bmjopen-2019-035775	Martini 2020	Irrelevant	Excluded by TI/AB review
2175	Emergency department patients' perceptions of the efficacy and safety of opioid [illegible]	Mazer-Amirshahi, M.; Sun, C.; Koroshetz, L.; Soares, R. W.; Kumar, A.; Calello, D. P.; Perrone, J.; [illegible]	2020	Journal of Opioid Management	16	4	239-246	10.5055/jom.2020.0577	Mazer-Amirshahi 2020	Irrelevant	Excluded by TI/AB review

S4 Table. All studies identified in the literature search

2176	355 Unheard Victims: Multidisciplinary Incidence of Violence among Emergency Department Staff at an Academic Medical Center	McGuire, S.; Clements, C.	2020	Annals of Emergency Medicine	76	4	S137	10.1016/j.annemergmed.2020.09.371	McGuire 2020	Irrelevant	Excluded by TI/AB review
2177	Comparative effectiveness of two individualized acute pain treatment communication tools: the life stories for opioid risk reduction in the emergency department (STORRIED)	Meisel, Z. F.; Cannuscio, C.; Rhodes, K.; Shofer, F.; Perrone, J.; Hess, E. P.; Goldberg, E.; Dolan, A. R.; Rogers, M.; Bellamkonda, V.; Bell, J.; Zyla, M.; Becker, L.; McCollum, S.; Schapira, M.	2020	Academic Emergency Medicine	27	NA	S7	10.1111/acem.13961	Meisel 2020	Irrelevant	Excluded by TI/AB review

S4 Table. All studies identified in the literature search

2178	The mobile medical and mental health care (M3) team: An innovative, integrated model of care for individuals experiencing homelessness in austin, texas	Mercer, T.; Kaiser, K.; Ahearn, R.; Benzer, J.; Correa, M.; Hancock, J.; Kuang, A.; Laguna, C.; Rivera, J.	2020	Journal of General Internal Medicine	35	SUPP L 1	S694-S695	10.1007/s11606-020-05890-3	Mercer 2020	Irrelevant	Excluded by TI/AB review
2179	Healthcare utilization and patient and provider experience with a home visit program for patients discharged from the hospital at high risk for readmission: A mixed methods study	Misra-Hebert, A. D.; Rothberg, M. B.; Fox, J.; Ji, X.; Hu, B.; Milinovich, A.; Zafirau, W.; Onuzuruike, A.; Stange, K. C.	2020	Journal of General Internal Medicine	35	SUPP L 1	S144-S145	10.1007/s11606-020-05890-3	Misra-Hebert 2020	Irrelevant	Excluded by TI/AB review

S4 Table. All studies identified in the literature search

2180	Identifying disparities in patient-centered care experiences between non-Latino white and [illegible]	Mitchell, Jamie A.; Williams, Ed-Dee G.; Li, Yuyi; Tarraf, Wassim	2020	BMC Health Services Research	20	1	9-Jan	10.1186/s12913-020-05357-5	Mitchell 2020	Irrelevant	Excluded by TI/AB review
2181	Hospital Readmission and Emergency Department Revisits of [illegible]	Miyawaki, A.; Hasegawa, K.; Figueroa, J. F.; Tsugawa, Y.	2020	Journal of General Internal Medicine	35	9	2560-2568	10.1007/s11606-020-06029-0	Miyawaki 2020	Irrelevant	Excluded by TI/AB review
2182	Medication Errors Upon Hospital Discharge to Skilled Nursing Facility	Montoya, A.; Beal, J.; Bautista, L.; Begum, R.; Diviney, E.; Jenq, G.; Krivchenya, T.; Nwagwu, V.; Stier, N.	2020	Journal of the American Medical Directors Association	21	3	B32	10.1016/j.jamda.2020.01.088	Montoya 2020	Irrelevant	Excluded by TI/AB review
2183	Association Between Sociodemographic Determinants and Disparities in Stroke Symptom Awareness [illegible]	Mszar, Reed; Mahajan, Shiwani; Valero-Elizondo, Javier; Yahya, Tamer; Sharma, Richa; Grandhi, Gowtham R.; Khera, [illegible]	2020	Stroke (00392499)	51	12	3552-3561	10.1161/STROKEAHA.120.031137	Mszar 2020	Irrelevant	Excluded by TI/AB review

S4 Table. All studies identified in the literature search

2185	Decrease in Urologic Discharge Opioid Prescribing after Mandatory Query of Statewide Prescription Drug [illegible]	Myrga, J. M.; Macleod, L. C.; Bandari, J.; Jacobs, B. L.; Davies, B. J.	2020	Urology	139	NA	84-89	10.1016/j.urology.2020.02.002	Myrga 2020	Irrelevant	Excluded by TI/AB review
2186	Factors that influence family and parental preferences and decision making for unscheduled paediatric healthcare - [illegible]	Nicholson, E.; McDonnell, T.; De Br√∫n, A.; Barrett, M.; Bury, G.; Collins, C.; Hensey, C.; McAuliffe, E.	2020	BMC Health Services Research	20	1	663	10.1186/s12913-020-05527-5	Nicholson 2020	Irrelevant	Excluded by TI/AB review
2187	Impact of a self-monitoring application on pediatric asthma disparities	Nkoy, F. L.; Wilkins, V. L.; Fassl, B. A.; Sheng, X.; Stone, B. L.	2020	International Journal of Medical Informatics	144	NA	NA	10.1016/j.ijmedinf.2020.104294	Nkoy 2020	Irrelevant	Excluded by TI/AB review
2188	Challenges in Reporting the Effect of Radiotherapy on Erectile Function	Nukala, V.; Incrocci, L.; Hunt, A. A.; Ballas, L.; Koontz, B. F.	2020	Journal of Sexual Medicine	17	6	1053-1059	10.1016/j.jsxm.2020.03.008	Nukala 2020	Irrelevant	Excluded by TI/AB review

S4 Table. All studies identified in the literature search

2189	A Clinician's Experience in Initiation and Navigation of a Support Group for [illegible]	Nwabueze, C.	2020	Blood	136	NA	13-14	10.1182/blood-2020-142449	Nwabueze 2020	Irrelevant	Excluded by TI/AB review
2190	Effects of state-level medicaid expansion on veterans health administration dual enrollment and utilization: Potential implications for future coverage expansions	O'Mahen, P. N.; Petersen, L. A.	2020	Medical Care	58	6	526-533	10.1097/MLR.0000000000001327	O'Mahen 2020	Irrelevant	Excluded by TI/AB review
2191	Evaluation of Racial Disparities in Hospice Use and End-of-Life Treatment Intensity in the REGARDS Cohort	Ornstein, K. A.; Roth, D. L.; Huang, J.; Levitan, E. B.; Rhodes, J. D.; Fabius, C. D.; Safford, M. M.; Sheehan, O. C.	2020	JAMA Network Open	NA	NA	NA	10.1001/jamanetworkopen.2020.14639	Ornstein 2020	Irrelevant	Excluded by TI/AB review

S4 Table. All studies identified in the literature search

2193	Patient race, ethnicity, and care in the emergency department: A scoping review	Owens, A.; Holroyd, B. R.; McLane, P.	2020	CJEM	22	2	245-253	10.1017/cem.2019.458	Owens 2020	Irrelevant	Excluded by TI/AB review
2196	37 Impact of Census-Based Reassignment of Nursing Staff to Reduce Emergency Department Patient Wait Times, Lengths of Stay, and Boarding Times	Park, J.; Gogia, K.; Sarhangian, V.; Vasquez, C.; Clark, S.; Chan, C.; Sharma, R.	2020	Annals of Emergency Medicine	76	4	S14-S15	10.1016/j.annemergmed.2020.09.047	Park 2020	Irrelevant	Excluded by TI/AB review
2197	Health care coach support in reducing acute care use in patients with cancer	Patel, M. I.; Smith, K.; Sisay, E.; Park, D. J.	2020	Journal of Clinical Oncology	38	29	NA	10.1200/JCO.2020.38.29	Patel 2020	Irrelevant	Excluded by TI/AB review

S4 Table. All studies identified in the literature search

2198	Head and neck opioid-prescribing patterns in the at-risk, state-subsidized hospital setting	Patel, P. A.; Nagappan, L.; Schmalbach, C. E.	2020	Otolaryngology - Head and Neck Surgery	163	1 SUPPL	P116	10.1177/0194599820934780	Patel 2020	Irrelevant	Excluded by TI/AB review
2199	PRS58 ASSOCIATION BETWEEN ASTHMA SELF-MANAGEMENT KNOWLEDGE, ASTHMA CONTROL, AND HEALTH-[illegible]	Pathan, F.; Young, H. N.; Goode, T. W.	2020	Value in Health	23	NA	S359	10.1016/j.jval.2020.04.1377	Pathan 2020	Irrelevant	Excluded by TI/AB review
2200	Brief online educational intervention improves emergency physicians' and general surgeon's ability to interpret focused [illegible]	Peng, S.; Woo, M.; Glen, P.; Ritcey, B.; Cheung, W.; Kwok, E.; Sheikh, A.	2020	Canadian Journal of Emergency Medicine	22	NA	S81	10.1017/cem.2020.254	Peng 2020	Irrelevant	Excluded by TI/AB review

S4 Table. All studies identified in the literature search

2201	Permanent Supportive Housing With Housing First to Reduce Homelessness and [illegible]	Peng, Y.; Hahn, R. A.; Finnie, R. K. C.; Cobb, J.; Williams, S. P.; Fielding, J. E.; Johnson, R. L.; Montgomery, A. E.; [illegible]	2020	Journal of public health management and practice : JPHMP	26	5	404-411	10.1097/PHH.0000000000001219	Peng 2020	Irrelevant	Excluded by TI/AB review
2202	Cardiac diagnoses and initial presentation to the emergency department may be associated with hospital mortality	Piasecki, R. J.; Bahouth, M. N.; Budhathoki, C.; Newton, H. M.; Duval-Arnould, J. M.; Dennison Himmelfarb, C. R.; [illegible]	2020	Circulation	140	NA	NA	10.1161/circ.140.suppl_2.346	Piasecki 2020	Irrelevant	Excluded by TI/AB review
2203	Can Emoji's assess patients,Äô mood and emotion in the emergency department? An emoji based study	Pourmand, A.; Quan, T.; Amini, S. B.; Sikka, N.	2020	American Journal of Emergency Medicine	38	4	842-843	10.1016/j.ajem.2019.09.008	Pourmand 2020	Irrelevant	Excluded by TI/AB review

S4 Table. All studies identified in the literature search

2204	Supporting black lives ‚Äòmattering‚Äô with flexible health care	Preisz, P.; Preisz, A.	2020	Journal of Paediatrics and Child Health	56	12	1848-1850	10.1111/jpc.15128	Preisz 2020	Irrelevant	Excluded by TI/AB review
2205	Inappropriate antibiotic prescribing for respiratory conditions does not improve press ganey patient satisfaction scores in the emergency department	Pulia, M. S.; Hesse, S.; Schwei, R. J.; Schulz, L. T.; Sethi, A.; Hamedani, A.	2020	Open Forum Infectious Diseases	7	6	NA	10.1093/OFID/OFAA214	Pulia 2020	Irrelevant	Excluded by TI/AB review
2206	Experience of emergency department use among persons with a history of adverse childhood experiences	Purkey, E.; Davison, C.; MacKenzie, M.; Beckett, T.; Korpal, D.; Soucie, K.; Bartels, S.	2020	BMC Health Services Research	20	1	455	10.1186/s12913-020-05291-6	Purkey 2020	Irrelevant	Excluded by TI/AB review

S4 Table. All studies identified in the literature search

2207	252 A Qualitative Study of the Experience of Abuse by Health Care Workers in the Emergency Department	Querin, L. B.; Beck Dallaghan, G. L.; Shenvi, C.	2020	Annals of Emergency Medicine	76	4	S97	10.1016/j.annemergmed.2020.09.266	Querin 2020	Irrelevant	Excluded by TI/AB review
2208	Are There Nationwide Socioeconomic and Demographic Disparities in the Use of Outpatient Orthopaedic [illegible]	Rabah, N. M.; Knusel, K. D.; Khan, H. A.; Marcus, R. E.	2020	Clin Orthop Relat Res	478	5	979-989	10.1097/corr.0000000000001168	Rabah 2020	Irrelevant	Excluded by TI/AB review
2212	Randomized Controlled Trial of Adult Therapeutic Coloring for the Management of Significant Anxiety in the Emergency Department	Rajendran, N.; Mitra, T. P.; Shahrestani, S.; Coggins, A.	2020	Academic Emergency Medicine	27	2	92-99	10.1111/acem.13838	Rajendran 2020	Irrelevant	Excluded by TI/AB review

S4 Table. All studies identified in the literature search

2213	Rates of and reasons for revisits after deliveries with and without severe maternal morbidity	Reid, L.; Fingar, K.; Barrett, M.; McDermott, K.; Ngo-Metzger, Q.; Owens, P.	2020	Health Services Research	55	SUPPL 1	144-145	10.1111/1475-6773.13537	Reid 2020	Irrelevant	Excluded by TI/AB review
2214	Predictors of Overtesting in Pulmonary Embolism Diagnosis	Richardson, S.; Lucas, E.; Cohen, S. L.; Zhang, M.; Qiu, G.; Khan, S.; McGinn, T.	2020	Academic Radiology	27	3	404-408	10.1016/j.acra.2019.04.018	Richardson 2020	Irrelevant	Excluded by TI/AB review
2215	The influence of patient race on the use of diagnostic imaging in United	Ross, A. B.; Kalia, V.; Chan, B. Y.; Li, G.	2020	BMC Health Services Research	20	1	NA	10.1186/s12913-020-05698-1	Ross 2020	Irrelevant	Excluded by TI/AB review
2216	Adherence to postoperative thromboprophylactic medication among gynecologic oncology patients: A subanalysis	Ross, M. E.; Glickman, A.; Brennecke, A.; Tayebnejad, A.; Guntupalli, S. R.	2020	Gynecologic Oncology	158	3	754-759	10.1016/j.ygyno.2020.06.505	Ross 2020	Irrelevant	Excluded by TI/AB review

S4 Table. All studies identified in the literature search

2217	Understanding a vulnerable population: Low health literacy in cancer patients undergoing surgery at an NCI-designated comprehensive cancer center	Rothermel, L. D.; Conley, C. C.; Young, M. F.; Uscanga, Z. L.; Zhu, S. S.; Baez, H. D.; Wilkes, J. G.; Sarode, A. L.; Vadaparampil, S. T.; Fleming, J.	2020	Annals of Surgical Oncology	27	NA	S96	10.1245/s10434-020-08278-z	Rothermel 2020	Irrelevant	Excluded by TI/AB review
2218	The impact of HIV knowledge and attitudes on HIV testing acceptance among patients in an emergency department in the Eastern Cape, South Africa	Ryan, S.; Hahn, E.; Rao, A.; Mwinnyaa, G.; Black, J.; Maharaj, R.; Mvandaba, N.; Nyanisa, Y.; Quinn, T. C.; Hansoti, B.	2020	BMC Public Health	20	1	1066	10.1186/s12889-020-09170-x	Ryan 2020	Irrelevant	Excluded by TI/AB review
2219	The Effect of a County Prehospital FAST-ED Initiative on Endovascular Treatment Times	Rynor, H.; Levine, J.; Souchak, J.; Shashoua, N.; Ramirez, M.; Gonzalez, I. C.; Ramos, V.; Saxena, [illegible]	2020	Journal of Stroke and Cerebrovascular Diseases	29	11	NA	10.1016/j.jstrokecerebrovasdis.2020.105220	Rynor 2020	Irrelevant	Excluded by TI/AB review

S4 Table. All studies identified in the literature search

2220	Improving STEMI management in the emergency department: Examining the role of minority groups and sociodemographic characteristics	Saban, M.; Shachar, T.; Salama, R.; Darawsha, A.	2020	Am J Emerg Med	38	6	1102-1109	10.1016/j.ajem.2019.158380	Saban 2020	Irrelevant	Excluded by TI/AB review
2222	CHARACTERIZATION OF UNPLANNED HOSPITAL VISITS AFTER EGD AND COLONOSCOPY IN MARYLAND	Sakulsaengprapha, V.; Rifkin, S.; Mathews, S. C.	2020	Gastroenterology	158	6	S-1240	10.1016/S0016-5085(20)33762-8	Sakulsaengprapha 2020	Irrelevant	Excluded by TI/AB review

S4 Table. All studies identified in the literature search

2223	Association of Display of Patient Photographs in the Electronic Health Record with Wrong-Patient Order Entry Errors	Salmasian, H.; Blanchfield, B. B.; Joyce, K.; Centeio, K.; Schiff, G. B.; Wright, A.; Baugh, C. W.; Schuur, J. D.; Bates, D. W.; Adelman, J. S.; Landman, A. B.	2020	JAMA Network Open	3	11	NA	10.1001/jamanetworkopen.2020.19652	Salmasian 2020	Irrelevant	Excluded by TI/AB review
2224	Patient interest in video integration for after-hours telemedicine	Sankaran, J.; Menachery, S. M.; Bradshaw, R. D.	2020	Journal of the American Board of Family Medicine	33	5	765-773	10.3122/JABFM.2020.05.190362	Sankaran 2020	Irrelevant	Excluded by TI/AB review
2225	Racial disparities in the management of pediatric trauma in an urban academic emergency department	Sausen, N. J.; Okeke-Eweni, K.; Rhodes, S.; Oral, E.; Moreno-Walton, L.	2020	Academic Emergency Medicine	27	NA	S143	10.1111/acem.13961	Sausen 2020	Irrelevant	Excluded by TI/AB review

S4 Table. All studies identified in the literature search

2228	Practice-level variation in telemedicine use in a pediatric primary care network during the COVID-19 pandemic: [illegible]	Schweiberger, K.; Hoberman, A.; Iagnemma, J.; Schoemer, P.; Squire, J.; Taormina, J.; Wolfson, D.; Ray, K. N.	2020	Journal of Medical Internet Research	22	12	NA	10.2196/24345	Schweiberger 2020	Irrelevant	Excluded by TI/AB review
2229	Predicting Pain-Related 30-Day Emergency Department Return Visits in Middle-Aged and Older Adults	Sheikh, S.; Booth-Norse, A.; Smotherman, C.; Kalynych, C.; Lukens-Bull, K.; Guerrido, E.; Henson, M.; Gautam, S.; Hendry, P.	2020	Pain Medicine (United States)	21	11	2748-2756	10.1093/PM/PNAA213	Sheikh 2020	Irrelevant	Excluded by TI/AB review
2230	Effects of variations in access to care for children with atopic dermatitis	Siegfried, E. C.; Paller, A. S.; Mina-Osorio, P.; Vekeman, F.; Kaur, M.; Mallya, U. G.; Heroux, J.; Miao, R.; Gadkari, A.	2020	Bmc Dermatology	20	1	NA	10.1186/s12895-020-00114-x	Siegfried 2020	Irrelevant	Excluded by TI/AB review
2231	Disdain-discrimination in the surgical discipline: An international european cross-sectional study	Skinner, H.; Holzgang, M.; Koenemann, N.; Burke, J.; Young, A.	2020	British Journal of Surgery	107	SUPPL 3	12-Nov	10.1002/bjs.11641	Skinner 2020	Irrelevant	Excluded by TI/AB review

S4 Table. All studies identified in the literature search

2232	Effect of Physician Gender and Race on Simulated Patients' Ratings and Confidence in Their Physicians: A Randomized Trial	Solnick, R. E.; Peyton, K.; Kraft-Todd, G.; Safdar, B.	2020	JAMA Network Open	3	2	NA	10.1001/jamanetworkopen.2019.20511	Solnick 2020	Irrelevant	Excluded by TI/AB review
2235	Utilization of a multidisciplinary emergency department sepsis huddle to reduce time to antibiotics and improve SEP-1 compliance	Sonis, J. D.; Benzer, T. I.; Black, L.; Filbin, M. R.; Hayes, B. D.; Hibbert, K. A.; Kraus, C.; Raja, A. S.; Temin, E.; Vareschi, M.; White, B. A.; Wilcox, S. R.; Aaronson, E. L.	2020	American Journal of Emergency Medicine	38	11	2400-2404	10.1016/j.ajem.2020.09.014	Sonis 2020	Irrelevant	Excluded by TI/AB review
2236	Diversity and inclusion in organizations	Stone, Dianna L.; Dulebohn, James H.; Lukaszewski, Kimberly M.	2020	Research in human resource management	NA	NA	NA	NA	Stone 2020	Irrelevant	Excluded by TI/AB review

S4 Table. All studies identified in the literature search

2237	A Patient With COVID-19¬†Is Left Behind As Care Goes Virtual	Sundar, K. R.	2020	Health Aff (Millwood)	39	8	1453-1455	10.1377/hlthaff.2020.00447	Sundar 2020	Irrelevant	Excluded by TI/AB review
2238	Medication nonadherence, mental health, opioid use, and inpatient and emergency department use in super-utilizers	Surbhi, S.; Graetz, I.; Wan, J. Y.; Gatwood, J.; Bailey, J. E.	2020	American Journal of Managed Care	26	3	E98-E103	10.37765/ajmc.2020.42642	Surbhi 2020	Irrelevant	Excluded by TI/AB review
2239	PNS117 AS IT GROWS, MEDICARE ADVANTAGE IS ENROLLING MORE LOW-INCOME AND MEDICALLY [illegible]	Teigland, C.; Pulungan, Z.	2020	Value in Health	23	NA	S305-S306	10.1016/j.jval.2020.04.1120	Teigland 2020	Irrelevant	Excluded by TI/AB review

S4 Table. All studies identified in the literature search

2240	Examining the relationship between ambient carbon monoxide, nitrogen dioxide, and mental health-related emergency department visits in California, USA	Thilakaratne, R. A.; Malig, B. J.; Basu, R.	2020	Science of the Total Environment	746	NA	NA	10.1016/j.scitotenv.2020.140915	Thilakaratne 2020	Irrelevant	Excluded by TI/AB review
2241	Emergency department-mediated cancerdiagnosis in the United States	Thompson, C. A.; Sheridan, P.; Murphy, J. D.; Lyratzopoulos, G.	2020	Cancer Epidemiology Biomarkers and Prevention	29	6 SUPPL 2	NA	10.1158/1538-7755.DISP19-PR10	Thompson 2020	Irrelevant	Excluded by TI/AB review

S4 Table. All studies identified in the literature search

2242	Racial and ethnic disparities in care for health system-affiliated physician organizations and non-affiliated physician organizations	Timbie, J. W.; Kranz, A. M.; DeYoreo, M.; Eshete-Roesler, B.; Elliott, M. N.; Escarce, J. J.; Totten, M. E.; Damberg, C. L.	2020	Health Services Research	55	S3	1107-1117	10.1111/1475-6773.13581	Timbie 2020	Irrelevant	Excluded by TI/AB review
2243	Effectiveness of a housing support team intervention with a recovery-oriented approach on hospital and emergency department use by homeless people with severe mental illness: A randomised controlled trial	Tinland, A.; Loubi√®re, S.; Boucekine, M.; Boyer, L.; Fond, G.; Girard, V.; Auquier, P.	2020	Epidemiology and Psychiatric Sciences	NA	NA	NA	10.1017/S2045796020000785	Tinland 2020	Irrelevant	Excluded by TI/AB review

S4 Table. All studies identified in the literature search

2244	Clinical and chest radiography features determine patient outcomes in young and middle-aged adults with COVID-19	Toussie, D.; Voutsinas, N.; Finkelstein, M.; Cedillo, M. A.; Manna, S.; Maron, S. Z.; Jacobi, A.; Chung, M.; Bernheim, A.; Eber, [illegible]	2020	Radiology	297	1	E197-E206	10.1148/radiol.2020201754	Toussie 2020	Irrelevant	Excluded by TI/AB review
2245	The effectiveness of telepharmacy anticoagulation service in the ambulatory care setting: A systematic review and metaanalysis	Tran, R.; Yamzon, J.; Stewart, T.; Hernandez, E.; Cao, D.	2020	JACCP Journal of the American College of Clinical Pharmacy	3	8	1683	10.1002/jac5.1351	Tran 2020	Irrelevant	Excluded by TI/AB review
2246	Evaluation of the association between 30-day readmissions and receipt of home health services in geriatric patients	Trutner, Z. D.; Sorensen, A.; Villaflores, C.; Komberg, J.; Meshkat, S.; Sarkisian, C.	2020	Journal of General Internal Medicine	35	SUPPL 1	S118-S119	10.1007/s11606-020-05890-3	Trutner 2020	Irrelevant	Excluded by TI/AB review

S4 Table. All studies identified in the literature search

2247	Female millennial patient perspectives in the emergency department: A qualitative analysis	Upton, E.; Sangal, R. B.; Blutinger, E.; Khachatryan, M.; Aysola, J.; Mamtani, M.	2020	American Journal of Emergency Medicine	38	11	2462-2464	10.1016/j.ajem.2020.02.026	Upton 2020	Irrelevant	Excluded by TI/AB review
2248	Trial study design to test a bilingual digital health tool for alcohol use disorders among [illegible]	Vaca, F. E.; Dziura, J.; Abujarad, F.; Pantalon, M. V.; Hsiao, A.; Field, C. A.; D'Onofrio, G.	2020	Contemp Clin Trials	96	NA	106104	10.1016/j.cct.2020.106104	Vaca 2020	Irrelevant	Excluded by TI/AB review
2251	Trial study design to test a bilingual digital health tool for alcohol use disorders among Latino emergency department patients	Vaca, F. E.; Dziura, J.; Abujarad, F.; Pantalon, M. V.; Hsiao, A.; Field, C. A.; D'Onofrio, G.	2020	Contemp Clin Trials	97	NA	106128	10.1016/j.cct.2020.106128	Vaca 2020	Irrelevant	Excluded by TI/AB review
2253	Hospitalisation for cirrhosis in Australia: Disparities in presentation and outcomes for [illegible]	Valery, P. C.; Clark, P. J.; Pratt, G.; Bernardes, C. M.; Hartel, G.; Toombs, M.; Irvine, K. M.; Powell, E. E.	2020	International Journal for Equity in Health	19	1	NA	10.1186/s12939-020-1144-6	Valery 2020	Irrelevant	Excluded by TI/AB review

S4 Table. All studies identified in the literature search

2254	Optimizing Pain Management for Children with Sickle Cell Disease Vaso-Occlusive Crisis in the Emergency	Vasconez, W. A.; Aguilar-Velez, C.; Matheus, C.; Sosa, M. A.; Diaz, Y.; Narahari, P.; Middleton, R. D.; Sterling, S.; Rogers,	2020	Blood	136	NA	44	10.1182/blood-2020-139909	Vasconez 2020	Irrelevant	Excluded by TI/AB review
2256	Association of Outpatient Practice-Level	Wadhera, Rishi K.; Bhatt, Deepak L.; Kind, Amy J. H.;	2020	Circulation: Cardiovascular Quality	13	4	e005977-e005977	10.1161/CIRCOUTCOMES.119.0	Wadhera 2020	Irrelevant	Excluded by TI/AB review
2258	(social) profiling youth with T1D: Examining the standard care	Wagner, D. V.; Hartman, A.; Jenisch, C.; Guttmann-Bauman, I.; El-	2020	Diabetes	69	NA	NA	10.2337/db20-747-P	Wagner 2020	Irrelevant	Excluded by TI/AB review
2259	46 Workplace Violence in the Chinese Emergency Department: a 2019 Prospective	Walline, J. H.; Song, P. P.; Hung, K. K.; Graham C, A.	2020	Annals of Emergency Medicine	76	4	S19	10.1016/j.annemergmed.2020.09.056	Walline 2020	Irrelevant	Excluded by TI/AB review
2260	High temperatures and emergency department visits in 18 sites with different climatic characteristics in	Wang, Y.; Liu, Y.; Ye, D.; Li, N.; Bi, P.; Tong, S.; Wang, Y.; Cheng, Y.; Li, Y.; Yao, X.	2020	Environment International	136	NA	NA	10.1016/j.envint.2020.105486	Wang 2020	Irrelevant	Excluded by TI/AB review

S4 Table. All studies identified in the literature search

2261	End-of-life quality metrics among medicare decedents at minority-serving cancer centers: A [illegible]	Wasp, G. T.; Alam, S. S.; Brooks, G. A.; Khayal, I. S.; Kapadia, N. S.; Carmichael, D. Q.; Austin, A. M.; [illegible]	2020	Cancer Med	9	5	1911-1921	10.1002/cam4.2752	Wasp 2020	Irrelevant	Excluded by TI/AB review
2263	Associations between medicare-billed advance care planning (ACP) among seriously ill patients and their [illegible]	Weissman, J.	2020	Health Services Research	55	SUPPL 1	17-18	10.1111/1475-6773.13346	Weissman 2020	Irrelevant	Excluded by TI/AB review
2264	28.6 INPATIENT PSYCHIATRIC HOSPITALIZATIONS AND HEALTH CARE UTILIZATION OF CHILDREN [illegible]	Weleff, J. A.; Wilson, J.; Lopez, R.; Tang, A.; Malone, D.; Tampi, R.; Falcone, T.; Phelan, M.	2020	Journal of the American Academy of Child and Adolescent Psychiatry	59	10	S204	10.1016/j.jaac.2020.08.251	Weleff 2020	Irrelevant	Excluded by TI/AB review

S4 Table. All studies identified in the literature search

2265	Self-Reported Quality, Health, and Cost-Related Outcomes of Care Coordination Among Patients with Complex Health Needs	Wells, Rebecca; Breckenridge, Ellen D.; Sia√±ez, M√≥nica; Tamayo, Loida; Kum, Hye-Chung; Ohsfeldt, Robert L.	2020	Population Health Managemen t	23	1	59-67	10.1089/po p.2019.000 7	Wells 2020	Irrelevant	Excluded by TI/AB review
2266	Patients‚Äô response to an emergency department-based HIV testing program and perception of their friends‚Äô attitudes on HIV testing among patients seeking	Wicken, C.; Avornu, A.; Latkin, C. A.; Davey-Rothwell, M. A.; Kim, J.; Zaidi, R.; Rothman, R.; Hsieh, Y. H.	2020	Journal of Infection and Public Health	13	1	104-109	10.1016/j.ji ph.2019.07. 005	Wicken 2020	Irrelevant	Excluded by TI/AB review

S4 Table. All studies identified in the literature search

2267	Racial Disparities in Health Care Utilization at the End of Life Among New Jersey Medicaid Beneficiaries With Advanced Cancer	Yang, A.; Goldin, D.; Nova, J.; Malhotra, J.; Cantor, J. C.; Tsui, J.	2020	JCO Oncol Pract	16	6	e538-e548	10.1200/jop.19.00767	Yang 2020	Irrelevant	Excluded by TI/AB review
2268	Racial disparities in health care utilizationat the end of life among New Jersey Medicaidbeneficiar [illegible]	Yang, A. W.; Goldin, D.; Nova, J.; Malhotra, J.; Cantor, J.; Tsui, J.	2020	Cancer Epidemiology Biomarkers and Prevention	29	6 SUPPL 2	NA	10.1158/1538-7755.DISP19-C018	Yang 2020	Irrelevant	Excluded by TI/AB review
2269	Influence of time to diagnosis on time to percutaneous coronary intervention for st-elevation myocardial infarction patients: Door-to-electrocardiogram matters	Yiadom, M. Y. A. B.; Gong, W.; Patterson, B. W.; Baugh, C. W.; Mills, A. M.; Gavin, N.; Podolsky, S.; Salazar, G.; Azzo, C.; Dorner, S. C.; Hadley, K.; Lehmann, C.; Vogus, T.; Bernard, G. R.; Storrow, A. B.;	2020	Academic Emergency Medicine	27	NA	S127-S128	10.1111/acem.13961	Yiadom 2020	Irrelevant	Excluded by TI/AB review

S4 Table. All studies identified in the literature search

2270	Determinants of boarding of patients with severe mental illness in hospital	Yoon, Jangho; Bui, Linh N.; Govier, Diana J.; Cahn, Megan A.; Luck, Jeff	2020	Journal of Mental Health Policy and Economics	23	2	61-75	NA	Yoon 2020	Irrelevant	Excluded by TI/AB review
2271	Developing a population-based approach to sepsis care in the united states	Zebrowski, A. M.; Gaieski, D. F.; Buckler, D. G.; Wiebe, D.; Carr, B. G.	2020	Academic Emergency Medicine	27	NA	S20	10.1111/acem.13961	Zebrowski 2020	Irrelevant	Excluded by TI/AB review
2272	Iatrogenic complications of diabetes mellitus: An examination of hospital-acquired diabetic ketoacidosis and severe outpatient hypoglycemia	Zimmerman, Chloe Olivia	2020	NA	81	NA	NA	NA	Zimmerman 2020	Irrelevant	Excluded by TI/AB review

S4 Table. All studies identified in the literature search

2273	Blood culture results and overtreatment of patients with a one-hour sepsis bundle	Zitek, T.; Bourne, M.; Raber, J.; Shir, A.; Ryabtsev, B.	2020	Academic Emergency Medicine	27	NA	S74-S75	10.1111/acem.13961	Zitek 2020	Irrelevant	Excluded by TI/AB review
2274	Physician attitudes on buprenorphine induction in the emergency department: Results from a multistate survey	Zuckerman, M.; Kelly, T.; Heard, K.; Zosel, A.; Marlin, M.; Hoppe, J.	2020	Clinical Toxicology	58	11	1266-1267	10.1080/15563650.2020.1804238	Zuckerman 2020	Irrelevant	Excluded by TI/AB review
2275	SAEM21 Abstracts	NA	2021	Academic Emergency Medicine	28	SUPPL 1	NA	NA	NA	Irrelevant	Excluded by TI/AB review

S4 Table. All studies identified in the literature search

2276	Health insurance, pediatric asthma, and emergency department usage	NA	2021	Public Health Nursing	38	5	931-940	10.1111/phn.12926	NA	Irrelevant	Excluded by TI/AB review
2277	Completion of the two-dose recombinant zoster vaccine series in adults 50 years and older	Ackerson, B.; Qian, L.; Sy, L. S.; Bruxvoort, K.; Wu, J.; Luo, Y.; Diaz-Decaro, J.; Talarico, C.; Tseng, H. F.	2021	Vaccine	39	6	926-932	10.1016/j.vaccine.2020.12.076	Ackerson 2021	Irrelevant	Excluded by TI/AB review
2278	Association of resident characteristics with patterns of patient self-assignment	Agarwal, I.; Grossestreuer, A.; Joseph, J. W.; Leventhal, E. L.; Sanchez, L. D.	2021	American Journal of Emergency Medicine	44	NA	112-115	10.1016/j.ajem.2021.01.081	Agarwal 2021	Irrelevant	Excluded by TI/AB review
2279	Comparing medicare advantage and traditional medicare: A systematic review	Agarwal, R.; Connolly, J.; Gupta, S.; Navathe, A. S.	2021	Health Affairs	40	6	937-944	10.1377/hlthaff.2020.02149	Agarwal 2021	Irrelevant	Excluded by TI/AB review

S4 Table. All studies identified in the literature search

2281	COVID-19 Exposed America's Healthcare Faultlines. What Now?	Ahc, Media	2021	ED Management	33	3	4-Jan	NA	Ahc 2021	Irrelevant	Excluded by TI/AB review
2282	Epidemiology, economic and humanistic burdens of Ocular Surface Chemical Injury: A narrative review	Ahmmed, A. A.; Ting, D. S. J.; Figueiredo, F. C.	2021	Ocular Surface	20	NA	199-211	10.1016/j.jtos.2021.02.006	Ahmmed 2021	Irrelevant	Excluded by TI/AB review

S4 Table. All studies identified in the literature search

2283	An Electronically Delivered Patient-Activation Tool for Intensification of Medications for Chronic Heart Failure With Reduced Ejection Fraction: The EPIC-HF Trial	Allen, Larry A.; Venechuk, Grace; McIlvennan, Colleen K.; Page Ii, Robert L.; Knoepke, Christopher E.; Helmkamp, Laura J.; Khazanie, Prateeti; Peterson, Pamela N.; Pierce, Kenneth; Harger, Geoffrey; Thompson, Jocelyn S.; Dow, Tristan J.; Richards, Lance; Huang, Janice; Strader, James R.;	2021	Circulation	143	5	427-437	10.1161/CIRCULATIONAHA.120.051863	Allen 2021	Irrelevant	Excluded by TI/AB review
2285	COVID-19 vaccination hesitancy among healthcare personnel in the emergency department deserves continued attention	Amin, D. P.; Palter, J. S.	2021	American Journal of Emergency Medicine	48	NA	372-373	10.1016/j.ajem.2021.01.089	Amin 2021	Irrelevant	Excluded by TI/AB review

S4 Table. All studies identified in the literature search

2286	Nebulizer use by black and latinx patients with moderate to severe persistent asthma	Apter, A. J.; Carroll, J.; Maher, N.; Ericson, B.; Cardet, J. C.; Tulchinsky, A.; Rodriguez-Louis, J.; Cohen, R. I.; Colon-Moya, A.; Forth, V.; Morales-Cosme, W.; [illegible]	2021	American Journal of Respiratory and Critical Care Medicine	203	9	NA	10.1164/ajrccm-conference.2021.203.1_MeetingAbstracts.A1598	Apter 2021	Irrelevant	Excluded by TI/AB review
2287	The Effect of Homelessness on Patient Wait Times in the Emergency Department	Ayala, A.; Tegtmeyer, K.; Atassi, G.; Powell, E.	2021	Journal of Emergency Medicine	60	5	661-668	10.1016/j.jemermed.2020.12.031	Ayala 2021	Irrelevant	Excluded by TI/AB review
2293	Access to healthcare among Chinese immigrants living in Seville, Spain	Badanta-Romero, B.; Lucchetti, G.; Barrientos-Trigo, S.	2021	Gaceta sanitaria	35	2	145-152	10.1016/j.gaceta.2019.09.008	Badanta-Romero 2021	Irrelevant	Excluded by TI/AB review
2294	Covid-19 vaccine hesitancy among parents of children and adolescents living in brazil	Bagateli, L. E.; Saeki, E. Y.; Fadda, M.; Agostoni, C.; Marchisio, P.; Milani, G. P.	2021	Vaccines	9	10	NA	10.3390/vaccines9101115	Bagateli 2021	Irrelevant	Excluded by TI/AB review

S4 Table. All studies identified in the literature search

2295	Opioid-related emergency department visits and hospitalizations among patients with chronic gastrointestinal symptoms and disorders dually	Balbale, S. N.; Cao, L.; Trivedi, I.; Stulberg, J. J.; Suda, K. J.; Gellad, W. F.; Evans, C. T.; Jordan, N.; Keefer, L. A.; Lambert, B. L.	2021	American journal of health-system pharmacy : AJHP : official journal of the American	NA	NA	NA	10.1093/ajhp/zxab363	Balbale 2021	Irrelevant	Excluded by TI/AB review
2296	Cops, Clinicians, or Both? Collaborative Approaches to Responding to [illegible]	Balfour, M. E.; Hahn Stephenson, A.; Delany-Brumsey, A.; Winsky, J.; Goldman, M. L.	2021	Psychiatr Serv	NA	NA	appips202000721	10.1176/appi.ps.202000721	Balfour 2021	Irrelevant	Excluded by TI/AB review
2297	Optimizing health outcomes among newly dual eligibility beneficiaries in managed care: Patientprovider language, race and technology	Banerjee, S.; Ma, L.; Lee, C.; Schmittdiel, J.; Grant, R.; Adams, A.	2021	Health Services Research	56	SUPPL 2	49-50	10.1111/1475-6773.13817	Banerjee 2021	Irrelevant	Excluded by TI/AB review
2298	Examining the relationship between authentic leadership and workplace stress among emergency medicine workers	Barrera, Virginia	2021	NA	82	NA	NA	NA	Barrera 2021	Irrelevant	Excluded by TI/AB review

S4 Table. All studies identified in the literature search

2299	Parental preferences for survey mode of administration, interview versus	Barry, F.; Thompson, L. R.; Ravandi, B.; Chang, T. P.; Halterman, J. S.; Szilagyi, P. G.;	2021	Journal of Asthma	58	5	665-673	10.1080/02770903.2020.1728766	Barry 2021	Irrelevant	Excluded by TI/AB review
2300	Multi-dimensional impact of chronic low back pain among underserved african american and latino older adults	Bazargan, M.; Loeza, M.; Ekwegh, T.; Adinkrah, E. K.; Kibe, L. W.; Cobb, S.; Assari, S.; Bazargan-Hejazi, S.	2021	International Journal of Environmental Research and Public Health	18	14	NA	10.3390/ijerph18147246	Bazargan 2021	Irrelevant	Excluded by TI/AB review
2302	Perceived discrimination and blood pressure in individuals aging with traumatic brain injury	Bernier, Rachel A.; Venkatesan, Umesh M.; Soto, Jos√© A.; Rabinowitz, Amanda R.; Hong, Justin S.; Hillary, Frank G.	2021	Rehabilitation Psychology	66	2	148-159	10.1037/rep0000379	Bernier 2021	Irrelevant	Excluded by TI/AB review
2304	Examining frequent emergency department use among children	Beverly, J.; Giannouchos, T.; Callaghan, T.	2021	Autism	25	5	1382-1394	10.1177/13623613219990925	Beverly 2021	Irrelevant	Excluded by TI/AB review

S4 Table. All studies identified in the literature search

2305	Close to home: The effects of systemic racism and implicit bias on our own patients	Bhyravabhotla, K.; Dennar, P.	2021	Journal of General Internal Medicine	36	SUPPL 1	S294	10.1007/s11606-021-06830-5	Bhyravabhotla 2021	Irrelevant	Excluded by TI/AB review

S4 Table. All studies identified in the literature search

2306	Changes in weekend and weekday care quality of emergency medical admissions to 20 hospitals in England during implementation of the 7- day services national health policy	Bion, Julian; Aldridge, Cassie; Girling, Alan J.; Rudge, Gavin; Sun, Jianxia; Tarrant, Carolyn; Sutton, Elizabeth; Willars, Janet; Beet, Chris; Boyal, Amunpreet; Rees, Peter; Roseveare, Chris; Temple, Mark; Watson, Samuel Ian; Yen-Fu, Chen; Clancy, Mike; Rowan, Louise; Lord, Joanne; Mannion, Russell; Hofer, Timothy	2021	BMJ Quality & Safety	30	7	536-546	10.1136/bmjqs-2020-011165	Bion 2021	Irrelevant	Excluded by TI/AB review

S4 Table. All studies identified in the literature search

2307	Racial Discrimination is Associated with Acute Posttraumatic Stress Symptoms and Predicts Future Posttraumatic Stress Disorder Symptom Severity in Trauma-	Bird, C. M.; Webb, E. K.; Schramm, A. T.; Torres, L.; Larson, C.; deRoon-Cassini, T. A.	2021	Journal of traumatic stress	NA	NA	NA	10.1002/jts.22670	Bird 2021	Irrelevant	Excluded by TI/AB review
2309	26 Implementation of a COVID-19 Vaccine Emergency Department Education Program for [illegible]	Bischof, J. J.; Schoeffler, A.; Bashian, E.; Callender, N.; Fuentes, A.; Geyer, E.; More, A.; Webb, T.; Kman, K.	2021	Annals of Emergency Medicine	78	2	S13	10.1016/j.annemergmed.2021.07.027	Bischof 2021	Irrelevant	Excluded by TI/AB review
2310	Piloting a brief intervention plus mobile boosters for drug use among emerging adults receiving emergency department care	Bonar, E. E.; Cunningham, R. M.; Sweezea, E. C.; Blow, F. C.; Drislane, L. E.; Walton, M. A.	2021	Drug and Alcohol Dependence	221	NA	NA	10.1016/j.drugalcdep.2021.108625	Bonar 2021	Irrelevant	Excluded by TI/AB review

S4 Table. All studies identified in the literature search

2311	Markers of social disadvantage and hospital quality of care forpediatric respiratory illness	Bonsmith, K.; Desai, A. D.; Zhou, C.; Kenyon, C.; Johnson, D. P.; Lopez, M.; Tyler, A.; Desai, S.; Mangione-Smith, R.	2021	Pediatrics	147	3	592-593	10.1542/peds.147.3-MeetingAbstract.592	Bonsmith 2021	Irrelevant	Excluded by TI/AB review
2312	Racial Bias Among Emergency Providers: Strategies to Mitigate Its Adverse Effects	Brockett-Walker, C.; Lall, M.; Evans, D. D.; Heron, S.	2021	Adv Emerg Nurs J	43	2	89-101	10.1097/tme.0000000000000352	Brockett-Walker 2021	Irrelevant	Excluded by TI/AB review
2316	Experiences of underrepresented medical students and physicians in the emergency department: Implicit bias to [illegible]	Brown, C.	2021	Academic Emergency Medicine	28	SUPPL 1	S193	10.1111/acem.14249	Brown 2021	Irrelevant	Excluded by TI/AB review

S4 Table. All studies identified in the literature search

2317	The experiences of medical students, residents, fellows, and attendings in the emergency department: Implicit bias to microaggressions	Brown, C.; Daniel, R.; Addo, N.; Knight, S.	2021	Aem Education and Training	5	NA	S49-S56	10.1002/aet2.10670	Brown 2021	Irrelevant	Excluded by TI/AB review
2318	Addressing barriers to healthcare for refugee and immigrantchildren	Buhrman, D. Z.; Permashwar, V.; Sridhar, V.; Rahman, M.; Sorayya, A.	2021	Pediatrics	147	3	641-642	10.1542/peds.147.3-MeetingAbstract.642	Buhrman 2021	Irrelevant	Excluded by TI/AB review
2319	Racial and ethnic disparities in emergency department restraint use: A multicenter retrospective analysis	Carreras Tartak, J. A.; Brisbon, N.; Wilkie, S.; Sequist, T. D.; Aisiku, I. P.; Raja, A.; Macias-Konstantopoulos, W. L.	2021	Acad Emerg Med	28	9	957-965	10.1111/acem.14327	CarrerasTartak 2021	Irrelevant	Excluded by TI/AB review

S4 Table. All studies identified in the literature search

2323	Cultivating Compassion in the Emergency Department During COVID-19 and Racial Unrest	Carson, Cathy A.	2021	Beginnings	41	1	18-20	NA	Carson 2021	Irrelevant	Excluded by TI/AB review
2324	Mitigating the gender gap: How ‚ÄúDOCTOR‚Äù badges affect physician identity	Chang, J.; Arbo, J.; Jones, M. P.; Silverberg, J.; Corbo, J.	2021	American Journal of Emergency Medicine	46	NA	141-145	10.1016/j.ajem.2021.04.037	Chang 2021	Irrelevant	Excluded by TI/AB review
2325	Post-exposure prophylaxis for human immunodeficiency virus after sexual assault in a Midwestern U.S. emergency [illegible]	Cherabie, J. N.; Gleason, E.; Munigala, S.; Fox, B.; Trolard, A.; McCammon, C.; Hilbert, S.; Casabar, E.; Reno, H.; Liang, S. Y.	2021	American Journal of Emergency Medicine	49	NA	117-123	10.1016/j.ajem.2021.05.070	Cherabie 2021	Irrelevant	Excluded by TI/AB review

S4 Table. All studies identified in the literature search

2326	Differences in intensive care unit triage for racial and ethnic minorities with sepsis and acute respiratory failure	Chesley, C. F.; Anesi, G. L.; Schaubel, D.; Lane-Fall, M. B.; Halpern, S. D.	2021	American Journal of Respiratory and Critical Care Medicine	203	9	NA	10.1164/ajrccm-conference.2021.203.1_MeetingAbstracts.A1034	Chesley 2021	Irrelevant	Excluded by TI/AB review
2327	Identifying medication-related problems in pharmacist-run home visits	Chim, C.; Joseph, V.	2021	Journal of the American Pharmacists Association	61	3	e114-e118	10.1016/j.japh.2020.12.017	Chim 2021	Irrelevant	Excluded by TI/AB review
2328	Association between end-of-life cancer care and immigrant status: A retrospective cohort study in Ontario, Canada	Chu, A.; Barbera, L.; Sutradhar, R.; Oz, U. E.; O'Leary, E.; Seow, H.	2021	BMJ Open	11	6	NA	10.1136/bmjopen-2020-042978	Chu 2021	Irrelevant	Excluded by TI/AB review

S4 Table. All studies identified in the literature search

2330	Effectiveness and Reach of the Primary Palliative Care for Emergency Medicine (PRIM-ER) Pilot Study: a Qualitative [illegible]	Chung, F. R.; Turecamo, S.; Cuthel, A. M.; Grudzen, C. R.; Abella, B. S.; Abraham, G.; Reid, E. A.; Cridge, T. A.; Asselta, R.; Ballaraon, B.; [illegible]	2021	Journal of General Internal Medicine	36	2	296-304	10.1007/s11606-020-06302-2	Chung 2021	Irrelevant	Excluded by TI/AB review
2331	Heart Failure Admission Service Triage (H-FAST) Study: Racialized Differences in Perceived Patient Self-Advocacy as a Driver of [illegible]	Cleveland Manchanda, E. C.; Marsh, R. H.; Osuagwu, C.; Decopain Michel, J.; Dugas, J. N.; Wilson, M.; Morse, M.; Lewis, E.; [illegible]	2021	Cureus	13	2	e13381	10.7759/cureus.13381	Cleveland Manchanda 2021	Irrelevant	Excluded by TI/AB review
2333	,ÄúPassing out is a serious thing,Äù: Patient expectations for syncope evaluation and management	Clouser, J. M.; Sirrine, M.; McMullen, C. A.; Cowley, A. M.; Smyth, S. S.; Gupta, V.; Williams, M. V.; Li, J.	2021	Patient Preference and Adherence	15	NA	1213-1223	10.2147/PPA.S307186	Clouser 2021	Irrelevant	Excluded by TI/AB review

S4 Table. All studies identified in the literature search

2334	Quality of Care for Youth Hospitalized for Suicidal Ideation and Self-Harm	Connell, S. K.; Burkhart, Q.; Tolpadi, A.; Parast, L.; Gidengil, C. A.; Yung, S.; Basco, W. T.; Williams, D.; Britto, M. T.; Brittan, M.; Wood, K. E.; Bardach, N.; McGalliard, J.; Mangione-Smith, R.	2021	Acad Pediatr	21	7	1179-1186	10.1016/j.acap.2021.05.019	Connell 2021	Irrelevant	Excluded by TI/AB review
2338	Trust of caregivers: A prospective study of Hispanic vs non-Hispanic emergency department patients	Crawford, C.; Tom Xu, K.; Wallert, J.; Olsson, E.; Torres, R.; Richman, P. B.	2021	Academic Emergency Medicine	28	SUPPL 1	S181	10.1111/acem.14249	Crawford 2021	Irrelevant	Excluded by TI/AB review

S4 Table. All studies identified in the literature search

2339	Community partnerships within a novel dental school urgent care center: Student perceptions	Crivello, B. J.; Whitworth, B. C.; Pazdernik, V. K.; Singh, I.; Freihofer, J. M.; Davis, J. M.	2021	Journal of dental education	85	8	1396-1403	10.1002/jdd.12592	Crivello 2021	Irrelevant	Excluded by TI/AB review
2340	The Cincinnati Prehospital Stroke Scale Compared to Stroke Severity Tools for Large Vessel Occlusion [illegible]	Crowe, Remle P.; Myers, J. Brent; Fernandez, Antonio R.; Bourn, Scott; McMullan, Jason T.	2021	Prehospital Emergency Care	25	1	67-75	10.1080/10903127.2020.1725198	Crowe 2021	Irrelevant	Excluded by TI/AB review
2341	Examining emergency department inequities in Aotearoa New Zealand: Findings from a national [illegible]	Curtis, E.; Paine, S. J.; Jiang, Y.; Jones, P.; Tomash, I.; Healey, O.; Reid, P.	2021	Emerg Med Australas	NA	NA	NA	10.1111/1742-6723.13876	Curtis 2021	Irrelevant	Excluded by TI/AB review
2344	The impact of health literacy, socioeconomic status, and racial disparity in health outcomes	Danso, L. N. N.; Patel, D. R.	2021	Journal of General Internal Medicine	36	SUPPL 1	S295-S296	10.1007/s11606-021-06830-5	Danso 2021	Irrelevant	Excluded by TI/AB review

S4 Table. All studies identified in the literature search

2345	Exploring staff experiences: A case for redesigning the response to aggression and violence in the emergency department	Davids, J.; Murphy, M.; Moore, N.; Wand, T.; Brown, M.	2021	International emergency nursing	57	NA	101017	10.1016/j.ienj.2021.101017	Davids 2021	Irrelevant	Excluded by TI/AB review
2346	A predictive model for identifying patients at risk of delayed transfer of care: a retrospective, cross-sectional [illegible]	Davy, A.; Hill, T.; Jones, S.; Dube, A.; Lea, S. C.; Watts, K. L.; Asaduzzaman, M. D.	2021	Int J Qual Health Care	33	3	NA	10.1093/intqhc/mzab130	Davy 2021	Irrelevant	Excluded by TI/AB review
2348	Association between processes of diabetes care and health care utilization in patients with diabetes: Evidence from a nationally representative US sample	Delevry, D.; Ho, A.; Le, Q. A.	2021	Journal of Diabetes	13	1	78-88	10.1111/1753-0407.13109	Delevry 2021	Irrelevant	Excluded by TI/AB review

S4 Table. All studies identified in the literature search

2349	Identifying patient priorities to improve alcohol and other drug use disorders treatment outcomes during COVID-19	Dembner, A.; Hogan-Sanchez, J.; Kennedy, O.	2021	Alcoholism: Clinical and Experimental Research	45	SUPPL 1	257A	10.1111/acer.14628	Dembner 2021	Irrelevant	Excluded by TI/AB review
2350	Addressing distress management challenges: Recommendations from the consensus panel of the American Psychosocial Oncology Society and the Association of [illegible]	Deshields, T. L.; Wells-Di Gregorio, S.; Flowers, S. R.; Irwin, K. E.; Nipp, R.; Padgett, L.; Zebrack, B.	2021	CA Cancer Journal for Clinicians	71	5	407-436	10.3322/caac.21672	Deshields 2021	Irrelevant	Excluded by TI/AB review
2351	Racial and ethnic differences in patients with Parkinson's Disease across Parkinson's [illegible]	Di Luca, D.; Luo, S.; Davis, T.; Ramirez-Zamora, A.; Rafferty, M.; Dahodwala, N.; Marras, C.	2021	Movement Disorder	36	SUPPL 1	S71	10.1002/mds.28794	DiLuca 2021	Irrelevant	Excluded by TI/AB review
2352	Gluten-free minor cereals and pseudocereals intake in paediatric coeliac patients: our centre [illegible]	Di Mari, C.; Gianolio, L.; Pozzi, E.; Mantegazza, C.; Cocuccio, C.; Groppali, E.; Franchino, G.; [illegible]	2021	Journal of Pediatric Gastroenterology and Nutrition	72	SUPPL 1	269	10.1097/MPG.0000000000003177	DiMari 2021	Irrelevant	Excluded by TI/AB review

S4 Table. All studies identified in the literature search

2353	Clinical indicators of hereditary amyloid	Dyck, P.; Litchy, W.; Dyck, P.	2021	Journal of Neuromuscular	8	SUPPL 1	S95	10.3233/JND-219006	Dyck 2021	Irrelevant	Excluded by TI/AB review
2354	A quasiexperimental study of targeted normoxia in critically ill trauma patients	Dylla, L.; Anderson, E. L.; Douin, D. J.; Jackson, C. L.; Rice, J. D.; Schauer, S. G.; Neumann, R. T.; Bebarta, V. S.; Wright, F. L.; Ginde, A. A.	2021	The journal of trauma and acute care surgery	91	2S Suppl 2	S169-S175	10.1097/TA.0000000000003177	Dylla 2021	Irrelevant	Excluded by TI/AB review
2355	NarxCare Scores Greater Than 300 Are Associated with Adverse Outcomes After Primary THA	Emara, A. K.; Grits, D.; Klika, A. K.; Molloy, R. M.; Krebs, V. E.; Barsoum, W. K.; Higuera-Rueda, C.; Piuzzi, N. S.	2021	Clinical Orthopaedics and Related Research	479	9	1957-1967	10.1097/CORR.0000000000001745	Emara 2021	Irrelevant	Excluded by TI/AB review

S4 Table. All studies identified in the literature search

2356	Patient Preference and Risk Assessment in Opioid Prescribing Disparities: A Secondary Analysis of a Randomized Clinical Trial	Engel-Rebitzer, E.; Dolan, A. R.; Aronowitz, S. V.; Shofer, F. S.; Nguemeni Tiako, M. J.; Schapira, M. M.; Perrone, J.; Hess, E. P.; Rhodes, K. V.; Bellamkonda, V. R.; Cannuscio, C. C.; Goldberg, E.; Bell, J.; Rodgers, M. A.; Zyla, M.; Becker, L. B.; McCollum, S.; Meisel, Z. F.	2021	JAMA Network Open	4	7	NA	10.1001/jamanetworkopen.2021.18801	Engel-Rebitzer 2021	Irrelevant	Excluded by TI/AB review

S4 Table. All studies identified in the literature search

2357	Patient preference and risk in opioid disparities: A secondary analysis of a randomized controlled trial	Engel-Rebitzer, E.; Dolan, A. R.; Shofer, F.; Tiako, M. J. N.; Schapira, M. M.; Meisel, Z. F.; Rhodes, K. V.; Hess, E.; Bellamkonda, V.; Perrone, J.; Cannuscio, C.; Becker, L. B.; Aronowitz, S	2021	Academic Emergency Medicine	28	SUPP L 1	S196-S197	10.1111/acem.14249	Engel-Rebitzer 2021	Irrelevant	Excluded by TI/AB review
2358	Evidence for racial/ethnic disparities in emergency department visits following breast cancer surgery among women in California: a population-based	Falcone, M.; Liu, L. H.; Farias, A.; Zhang, J. N.; Kang, I. E.; Li, M.; Stern, M. C.; Lerman, C.	2021	Breast Cancer Research and Treatment	187	3	831-841	10.1007/s10549-021-06119-5	Falcone 2021	Irrelevant	Excluded by TI/AB review
2359	Emergency physician survey on firearm injury prevention: where can we improve?	Farcy, D. A.; Doria, N.; Moreno-Walton, L.; Gordon, H.; S√°nchez, J.; Cubeddu, L. X.; Ranney, M. L.	2021	Western Journal of Emergency Medicine	22	2	257-265	10.5811/WESTJEM.2020.11.49283	Farcy 2021	Irrelevant	Excluded by TI/AB review

S4 Table. All studies identified in the literature search

2360	COVID-19 vaccine hesitancy among patients in two urban emergency departments	Fern√°ndez-Penny, F. E.; Jolkovsky, E. L.; Shofer, F. S.; Hemmert, K. C.; Valiuddin, H.; Uspal, J. E.; Sands, N. A.; Abella, B. S.	2021	Academic Emergency Medicine	28	10	1100-1107	10.1111/acem.14376	Fern√°ndez-Penny 2021	Irrelevant	Excluded by TI/AB review
2361	The Social Determinants of Mental Health Assessment, Intervention, and Wholistic Health Equity: Part 2	Fink-Samnick, E.	2021	Professional case management	26	5	224-241	10.1097/NCM.0000000000000518	Fink-Samnick 2021	Irrelevant	Excluded by TI/AB review
2362	Moving Toward Non-invasive Hemodynamic and Echocardiographic Monitoring in Critical Pediatric Patients in the Emergency Department: Setting Standards	Fuenmayor-Oramas, F.; Gonz√°lez-Andrade, F.; la Cueva, X. P. D.	2021	SN Comprehensive Clinical Medicine	3	3	818-825	10.1007/s42399-021-00804-8	Fuenmayor-Oramas 2021	Irrelevant	Excluded by TI/AB review

S4 Table. All studies identified in the literature search

2363	Language barriers and timely analgesia for long bone fractures in a pediatric emergency department	Gaba, M.; Vazquez, H.; Homel, P.; Likourezos, A.; See, F.; Thompson, J.; Rizkalla, C.	2021	Western Journal of Emergency Medicine	22	2	225-231	10.5811/WESTJEM.2020.9.48431	Gaba 2021	Irrelevant	Excluded by TI/AB review
2365	Effectiveness of a Nurse-Led Multidisciplinary Intervention vs Usual Care on Advance Care Planning for Vulnerable Older Adults in an Accountable Care Organization: A Randomized	Gabbard, J.; Pajewski, N. M.; Callahan, K. E.; Dharod, A.; Foley, K. L.; Ferris, K.; Moses, A.; Willard, J.; Williamson, J. D.	2021	JAMA Internal Medicine	181	3	361-369	10.1001/jamainternmed.2020.5950	Gabbard 2021	Irrelevant	Excluded by TI/AB review

S4 Table. All studies identified in the literature search

2366	Rib fracture management: An audit investigating the speed and appropriateness of regional interventions	Gallaher, J.; Schneider-Smith, C.; Praman, C.	2021	Anaesthesia	76	SUPPL 5	40	10.1111/anae.15521	Gallaher 2021	Irrelevant	Excluded by TI/AB review
2367	Integrating Interprofessional Trainees into a Complex Care Program for Veterans Experiencing [illegible]	Gelberg, L.; Edwards, S. T.; Hooker, E. R.; Niederhausen, M.; Shaner, A.; Cowan, B. J.; Warde, C. M.	2021	Journal of General Internal Medicine	NA	NA	NA	10.1007/s11606-021-06856-9	Gelberg 2021	Irrelevant	Excluded by TI/AB review
2368	Concordance and Patient-Centered Care in Medicaid Enrollees' Care Experience With Providers	Ghabowen, I. K.; Bhandari, N.	2021	Journal of Patient Experience	8	NA	NA	10.1177/23743735211034028	Ghabowen 2021	Irrelevant	Excluded by TI/AB review

S4 Table. All studies identified in the literature search

2369	Palliative care services in elderly COVID-19 patients in the emergency department	Ghafoor, N.; Ravi, C.; Harth, R.; Gang, M.; Goett, R.; Ramdin, C.	2021	Academic Emergency Medicine	28	SUPP L 1	S261	10.1111/acem.14249	Ghafoor 2021	Irrelevant	Excluded by TI/AB review
2370	Cultural issues in play therapy, 2nd ed	Gil, Eliana; Drewes, Athena A.	2021	NA	NA	NA	NA	NA	Gil 2021	Irrelevant	Excluded by TI/AB review
2371	Feasibility of patient-reported diagnostic errors following emergency department discharge: A pilot [illegible]	Gleason, K. T.; Peterson, S.; Dennison Himmelfarb, C. R.; Villanueva, M.; Wynn, T.; Bondal, P.; Berg, D.; Jerde, [illegible]	2021	Diagnosis	8	2	187-192	10.1515/dx-2020-0014	Gleason 2021	Irrelevant	Excluded by TI/AB review

S4 Table. All studies identified in the literature search

2372	Variation in provider attitudes and treatment recommendations for individuals with schizophrenia and additional marginalized [illegible]	Gonzales, Lauren; L√≥pez-Aybar, Laura; McCullough, Brittany	2021	Psychiatric Rehabilitation Journal	44	2	107-117	10.1037/prj0000461	Gonzales 2021	Irrelevant	Excluded by TI/AB review
2373	Nurses' perceptions and demands regarding COVID-19 care delivery in critical care units and hospital emergency [illegible]	Gonz√°lez-Gil, M. T.; Gonz√°lez-Bl√°zquez, C.; Parro-Moreno, A. I.; Pedraz-Marcos, A.; Palmar-Santos, A.; Otero-Garc√≠a, L.; Navarta-S√°nchez, [illegible]	2021	Intensive & critical care nursing	62	NA	102966	10.1016/j.iccn.2020.102966	Gonz√°lez-Gil 2021	Irrelevant	Excluded by TI/AB review
2374	41 Health Insurance and Duration of Symptoms Prior to Emergency Department Visit: An Analysis of 19,850 Patients With Suspected COVID-19	Gunn, A. H.; Neptune, N.; Cunningham, H. M.; Conner, D.; Adkins, L.; Tait, S. D.; Pung, L.; Courtney, D. M.; Kline, J. A.; Purakal, J. D.	2021	Annals of Emergency Medicine	78	2	S21-S22	10.1016/j.annemergmed.2021.07.042	Gunn 2021	Irrelevant	Excluded by TI/AB review

S4 Table. All studies identified in the literature search

2375	Multi-Dimensional Assessment and Interdisciplinary Care to Reduce Asthma Readmissions in Safety Net Hospitals	Gupta, K.; Walton, R.; Ghani, N.; Vasudevan, V.	2021	Respiratory Care	NA	NA	NA	10.4187/respcare.08646	Gupta 2021	Irrelevant	Excluded by TI/AB review
2377	Sensor-based electronic monitoring for asthma: A randomized controlled trial	Gupta, R. S.; Fierstein, J. L.; Boon, K. L.; Kanaley, M. K.; Bozen, A.; Kan, K.; Vojta, D.; Warren, C. M.	2021	Pediatrics	147	1	NA	10.1542/PEDS.2020-1330	Gupta 2021	Irrelevant	Excluded by TI/AB review
2378	24 COVID-19 Vaccine Hesitancy Among Emergency Department Patients and Caregivers in New York City	Guzman, C.; Firew, T.; Wagh, A.; Stefan, B.; Ruscica, A.; Mitchell, C.; Bollman, E.; Depeter, K.; Hu, N.	2021	Annals of Emergency Medicine	78	2	S12	10.1016/j.annemergmed.2021.07.025	Guzman 2021	Irrelevant	Excluded by TI/AB review

S4 Table. All studies identified in the literature search

2379	'Emergency medical staff's perceptions on cultural value difference-based teamwork issues: A phenomenological study in China': Corrigendum	Hai,Äêping, Yu; Wei,Äêying, Zhang; You,Äêqing, Peng; Yun,Äêying, Hung; chi, Chen; Yang,Äêyang, Li; li,Äêli, Hou	2021	Journal of Nursing Management	29	2	356-356	10.1111/jonm.13197	Hai,Äêping 2021	Irrelevant	Excluded by TI/AB review
2380	Factors Associated with Voluntary Refusal of Emergency Medical System Transport for Emergency Care in Detroit during the Early Phase of the COVID-19 Pandemic	Harrison, N. E.; Ehrman, R. R.; Curtin, A.; Gorelick, D.; Hill, A. B.; Brennan, E.; Dunne, R.	2021	JAMA Network Open	4	8	NA	10.1001/jamanetworkopen.2021.20728	Harrison 2021	Irrelevant	Excluded by TI/AB review

S4 Table. All studies identified in the literature search

2382	Telehealth in emergency medicine: A consensus conference to map the intersection of telehealth and emergency medicine	Hayden, E. M.; Davis, C.; Clark, S.; Joshi, A. U.; Krupinski, E. A.; Naik, N.; Ward, M. J.; Zachrison, K. S.; Olsen, E.; Chang, B. A. P.; Burner, E.; Yadav, K.; Greenwald, P. W.; Chandra, S.	2021	Academic Emergency Medicine	NA	NA	NA	10.1111/acem.14330	Hayden 2021	Irrelevant	Excluded by TI/AB review
2383	Association between Race/Ethnicity and Disparities in Health Care Use before First-Episode Psychosis among Privately Insured Young Patients	Heun-Johnson, H.; Menchine, M.; Axeen, S.; Lung, K.; Claudius, I.; Wright, T.; Seabury, S. A.	2021	JAMA Psychiatry	78	3	311-319	10.1001/jamapsychiatry.2020.3995	Heun-Johnson 2021	Irrelevant	Excluded by TI/AB review
2384	Multivariable Analysis of Patient Satisfaction in the Pediatric Emergency Department	Heyming, T. W.; Donaldson, C. D.; Ehwerhemuepha, L.; Feaster, W.; Fortier, M. A.; Kain, Z. N.	2021	Pediatric Emergency Care	NA	NA	NA	10.1097/PEC.0000000000002514	Heyming 2021	Irrelevant	Excluded by TI/AB review

S4 Table. All studies identified in the literature search

2385	Racial and ethnic disparities in bronchiolitis management in freestanding children's hospitals	Honcoop, Aubree C.; Poitevien, Patricia; Kerns, Ellen; Alverson, Brian; McCulloh, Russell J.	2021	Academic Emergency Medicine	28	9	1043-1050	10.1111/acem.14274	Honcoop 2021	Irrelevant	Excluded by TI/AB review
2386	Changes in emergency department activity and the first covid-19 lockdown: A cross-sectional study	Honeyford, K.; Coughlan, C.; Nijman, R. G.; Expert, P.; Burcea, G.; Maconochie, I.; Kinderlerer, A.; Cooke, G. S.; Costelloe, C. E.	2021	Western Journal of Emergency Medicine	22	3	603-607	10.5811/westjem.2021.2.49614	Honeyford 2021	Irrelevant	Excluded by TI/AB review
2387	159 Comparing Patient Satisfaction in Penile Prosthesis Surgery	Howell, S.; Palasi, S.; Green, T.; Kannady, C.; Slaughter, K.; Wang,	2021	Journal of Sexual Medicine	18	3	S87-S88	10.1016/j.jsxm.2021.01.014	Howell 2021	Irrelevant	Excluded by TI/AB review
2388	Patient characteristics and perspectives of firearm safety discussions in the emergency department	Hudak, L.; Schwimmer, H.; Warnock, W.; Kilborn, S.; Moran, T.; Ackerman, J.; Rupp, J.	2021	Western Journal of Emergency Medicine	22	3	478-487	10.5811/westjem.2021.3.49333	Hudak 2021	Irrelevant	Excluded by TI/AB review

S4 Table. All studies identified in the literature search

2389	40 Facemasks: Perceptions and Use in an Emergency Department Population During COVID-19	Hunt, A.; Kelly, M.; Wilkerson, G.; Anaya, N.; Graterol, J.; Eucker, S.; Anderson, E.; Raja, A.; Morse, S.; Baumann, B.; Rodriguez, R.	2021	Annals of Emergency Medicine	78	2	S21	10.1016/j.annemergmed.2021.07.041	Hunt 2021	Irrelevant	Excluded by TI/AB review
2390	A case report of takotsubo syndrome complicated by ischaemic stroke: The clinical dilemma of anticoagulation	Iuliano, G.; Napoletano, R.; Vecchione, C.; Citro, R.	2021	European Heart Journal - Case Reports	5	3	NA	10.1093/ehjcr/ytab051	Iuliano 2021	Irrelevant	Excluded by TI/AB review
2391	The Canadian Women's Heart Health Alliance ATLAS on the Epidemiology, Diagnosis, and Management of [illegible]	Jaffer, S.; Foulds, H. J. A.; Parry, M.; Gonsalves, C. A.; Pacheco, C.; Clavel, M. A.; Mullen, K. A.; Yip, C. Y. Y.; Mulvagh, S. L.; [illegible]	2021	CJC Open	3	1	11-Jan	10.1016/j.cjco.2020.10.009	Jaffer 2021	Irrelevant	Excluded by TI/AB review

S4 Table. All studies identified in the literature search

2392	Pandemic Birthing: Childbirth Satisfaction,	Janevic, Teresa; Maru, Sheela; Nowlin, Sarah; McCarthy, Katharine;	2021	Maternal & Child Health Journal	25	6	860-869	10.1007/s10995-021-03158-8	Janevic 2021	Irrelevant	Excluded by TI/AB review
2395	The impact of delayed management of fall-related hip fracture management on health outcomes for African [illegible]	Jarman, M. P.; Sokas, C.; Dalton, M. K.; Castillo-Angeles, M.; Uribe-Leitz, T.; Heng, M.; von Keudell, A.; Cooper, Z.; Salim, A.	2021	Journal of Trauma and Acute Care Surgery	90	6	942-950	10.1097/TA.0000000000003149	Jarman 2021	Irrelevant	Excluded by TI/AB review
2396	Effectiveness of tailored peer-based sleep health education and social support in increasing home-[illegible]	Jean-Louis, G.; Seixas, A.; Rogers, A.; Blanc, J.; Jin, P.; Ravenell, J.; Ogedegbe, O.	2021	Sleep	44	SUPPL 2	A186-A187	10.1093/sleep/zsab072.472	Jean-Louis 2021	Irrelevant	Excluded by TI/AB review
2397	Patient-Physician Racial Concordance Associated with Improved Healthcare Use and Lower Healthcare [illegible]	Jetty, A.; Jabbarpour, Y.; Pollack, J.; Huerto, R.; Woo, S.; Petterson, S.	2021	J Racial Ethn Health Disparities	NA	NA	NA	10.1007/s40615-020-00930-4	Jetty 2021	Irrelevant	Excluded by TI/AB review

S4 Table. All studies identified in the literature search

2400	An Exploration of Emergency Department Visits for Home Unintentional Injuries Among Children With Autism Spectrum [illegible]	Jones, V.; Ryan, L.; Rooker, G.; Debinski, B.; Parnham, T.; Mahoney, P.; Shields, W.	2021	Pediatric Emergency Care	NA	NA	NA	10.1097/PEC.0000000000002536	Jones 2021	Irrelevant	Excluded by TI/AB review
2401	Participant-level characteristics differ by recruitment setting when evaluating a behavioral intervention targeting adolescents with asthma	Joseph, C. L. M.; Mahajan, P.; Buzzelli-Stokes, S.; Jacobsen, G.; Johnson, D. A.; Duffy, E.; Williams, R.; Havstad, S.; Zoratti, E.; Ownby, D. R.; Johnson, C. C.; Lu, M.	2021	Journal of Asthma	58	3	370-377	10.1080/02770903.2019.1690661	Joseph 2021	Irrelevant	Excluded by TI/AB review

S4 Table. All studies identified in the literature search

2402	Lesbian, Gay, Bisexual, Transgender, Queer Cultural Competency Training to Improve the Quality of Care: An Evidence	Kaiafas, K. N.; Kennedy, T.	2021	Journal of Emergency Nursing	47	4	654-660	10.1016/j.jen.2020.12.007	Kaiafas 2021	Irrelevant	Excluded by TI/AB review
2404	The impact of racism on emergency health care workers	Kaltiso, Sheri,ÄêAnn O.; Seitz, Roslyn M.; Zdradzinski, Michael J.; Moran, Timothy P.; Heron, Sheryl; Robertson, Jennifer; Lall, Michelle D.	2021	Academic Emergency Medicine	28	9	974-981	10.1111/acem.14347	Kaltiso 2021	Irrelevant	Excluded by TI/AB review
2405	Impact of mental illness on end-of-life emergency department use in elderly patients with gastrointestinal malignancies	Kashyap, M.; Harris, J. P.; Chang, D. T.; Pollom, E. L.	2021	Cancer Medicine	10	6	2035-2044	10.1002/cam4.3792	Kashyap 2021	Irrelevant	Excluded by TI/AB review

S4 Table. All studies identified in the literature search

2406	Prescribing phones to address health equity needs in the COVID-19 Era: The PHONE-CONNECT program	Kazevman, G.; Mercado, M.; Hulme, J.; Somers, A.	2021	Journal of Medical Internet Research	23	4	NA	10.2196/23914	Kazevman 2021	Irrelevant	Excluded by TI/AB review
2407	Racial Inequities in Pediatric Emergency Care	Kharbanda, Anupam B.	2021	NA	4	NA	e2034019-e2034019	10.1001/jamanetworkopen.2020.34019	Kharbanda 2021	Irrelevant	Excluded by TI/AB review
2408	Characteristics of sickle cell patients with frequent emergency department visits and hospitalizations	Kidwell, K.; Albo, C.; Pope, M.; Bowman, L.; Xu, H.; Wells, L.; Barrett, N.; Patel, N.; Allison, A.; Kutlar, A.	2021	PLoS One	16	2-Feb	NA	10.1371/journal.pone.0247324	Kidwell 2021	Irrelevant	Excluded by TI/AB review
2409	OCULOMOTOR NERVE PALSY AS A MANIFESTATION OF CRYPTOCOCCAL MENINGITIS IN A PATIENT WITH	Kim, I.; Wright, T.; Chandler, M.	2021	Chest	160	4	A766	10.1016/j.chest.2021.07.722	Kim 2021	Irrelevant	Excluded by TI/AB review

S4 Table. All studies identified in the literature search

2410	Determinants of racial and ethnic disparities in utilization of hospital-based care for asthma among Medi-Cal children in Los Angeles	Kim, Y.; Pirritano, M.; Parrish, K. M.	2021	Journal of Asthma	NA	NA	NA	10.1080/02770903.2021.1955131	Kim 2021	Irrelevant	Excluded by TI/AB review
2411	Utilization of the emergency department during COVID19 pandemic in pediatric sickle cell	Kirsch, A.; Apollonsky, N.	2021	Pediatric Blood and Cancer	68	SUPPL 3	S33	10.1002/pbc.29060	Kirsch 2021	Irrelevant	Excluded by TI/AB review
2412	Improving rates of timely completion of school asthma medication	Kopa, J.; Hargarten, L.; Sarmiento, A.; Hackley, B.; Scott, R.; Shapiro, A.	2021	Pediatrics	147	3	132-133	10.1542/peds.147.3_MeetingAbstract.132-a	Kopa 2021	Irrelevant	Excluded by TI/AB review

S4 Table. All studies identified in the literature search

2413	Exacerbation of Inequities in Use of Diagnostic Radiology During the Early Stages of Reopening After COVID-19	Lacson, R.; Shi, J.; Kapoor, N.; Eappen, S.; Boland, G. W.; Khorasani, R.	2021	Journal of the American College of Radiology	18	5	696-703	10.1016/j.jacr.2020.12.009	Lacson 2021	Irrelevant	Excluded by TI/AB review
2414	The role of an academic emergency department in advancing equity [illegible]	Landry, A. M.; Brown, I.; Blomkalns, A. L.; Wolfe, R. E.	2021	Academic Emergency Medicine	28	9	1087-1090	10.1111/acem.14164	Landry 2021	Irrelevant	Excluded by TI/AB review
2415	Perceptions of emergency care by sexual and gender minorities in colorado: Barriers, quality, and factors affecting identity disclosure	LaPlant, W. G.; Kattari, L.; Ross, L. K.; Zhan, J.; Druck, J. P.	2021	Western Journal of Emergency Medicine	22	4	903-910	10.5811/WESTJEM.2021.3.50981	LaPlant 2021	Irrelevant	Excluded by TI/AB review

S4 Table. All studies identified in the literature search

2418	Meeting the challenge of caring for persons living with dementia and their care partners and caregivers: A way forward	Larson, Eric B.; Stroud, Clare	2021	NA	NA	NA	NA	10.17226/26026	Larson 2021	Irrelevant	Excluded by TI/AB review
2419	Perceived Care Quality Among Women Receiving Sexual Assault Nurse Examiner Care: Results From a 1-Week Postexamination Survey in a Large [illegible]	Lechner, Megan; Bell, Kathy; Short, Nicole A.; Martin, Sandra L.; Black, Jenny; Buchanan, Jennie A.; Reese, Rhiannon; Ho, Jeffrey D.; Reed, Gordon D.; Platt, [illegible]	2021	JEN: Journal of Emergency Nursing	47	3	449-458	10.1016/j.jen.2020.11.011	Lechner 2021	Irrelevant	Excluded by TI/AB review
2420	Patient satisfaction with subdissociative ketamine vs morphine for emergency treatment of abdominal pain	Lee, R.; Youngblood, G.; Xu, K. T.; Torres, R.; Richman, P. B.	2021	Academic Emergency Medicine	28	SUPPL 1	S96	10.1111/acem.14249	Lee 2021	Irrelevant	Excluded by TI/AB review

S4 Table. All studies identified in the literature search

2421	Patient and healthcare professional satisfaction ratings and safety profile of sufentanil sublingual tablets for treatment of acute pain: A pooled	Leiman, D.; Jov√©, M.; Spahn, G. R.; Palmer, P.	2021	Journal of Pain Research	14	NA	805-813	10.2147/JPR.S291359	Leiman 2021	Irrelevant	Excluded by TI/AB review
2422	Evaluating for Racial Differences in Pain Management of Long-Bone Fractures in a Pediatric Rural Population	Liao, L.; Reyes, L.	2021	Pediatric Emergency Care	37	7	348-351	10.1097/PEC.0000000000001696	Liao 2021	Irrelevant	Excluded by TI/AB review
2425	Quality assessment of discharges against medical advice at a major urban emergency department	Liebow, J.; Ramdin, C.; Muckey, E.; Black, A.; Amin, A.; Gang, M.	2021	Academic Emergency Medicine	28	SUPPL 1	S283	10.1111/acem.14249	Liebow 2021	Irrelevant	Excluded by TI/AB review

S4 Table. All studies identified in the literature search

2426	Racial disparities in treatment and outcomes of children with type 1 diabetes	Lipman, T. H.; Smith, J. A.; Patil, O.; Willi, S. M.; Hawkes, C. P.	2021	Pediatric Diabetes	22	2	241-248	10.1111/pedi.13139	Lipman 2021	Irrelevant	Excluded by TI/AB review
2427	Is there an association between hot weather and poor mental health outcomes? A systematic review [illegible]	Liu, J.; Varghese, B. M.; Hansen, A.; Xiang, J.; Zhang, Y.; Dear, K.; Gourley, M.; Driscoll, T.; Morgan, G.; Capon, A.; Bi, P.	2021	Environment International	153	NA	NA	10.1016/j.envint.2021.106533	Liu 2021	Irrelevant	Excluded by TI/AB review
2428	Patient and Caregiver Outcomes and Experiences With Team-Based Memory Care: A Mixed Methods [illegible]	Liu, T. L.; Yates, T. D.; Taylor, Y. J.; Rossman, W.; Mangieri, D.; Black, S.; Stephens, C.; Connor, C. D.; Skudlarska, B.	2021	Journal of Applied Gerontology	40	8	872-880	10.1177/0733464819888838	Liu 2021	Irrelevant	Excluded by TI/AB review
2429	Association of Hispanic ethnicity and linguistic acculturation with cardiovascular medication adherence in patients with [illegible]	Liyanage-Don, N. A.; Cornelius, T.; Romero, E. K.; Alc√°ntara, C.; Kronish, I. M.	2021	Preventive Medicine Reports	23	NA	NA	10.1016/j.pmedr.2021.101455	Liyanage-Don 2021	Irrelevant	Excluded by TI/AB review

S4 Table. All studies identified in the literature search

2430	Discharge characteristics and care transitions of hospitalized patients with	Loerinc, L. B.; Scheel, A. M.; Evans, S. T.; Shabto, J. M.; O'Keefe, G. A.; O'Keefe, J. B.	2021	Healthcare	9	1	NA	10.1016/j.hjdsi.2020.100512	Loerinc 2021	Irrelevant	Excluded by TI/AB review
2431	Emergency department access during covid-19: Disparities in utilization by race/ethnicity, insurance, and income	Lowe, J.; Brown, I.; Duriseti, R.; Gallegos, M.; Ribeira, R.; Pirrotta, E.; Ewen Wang, N.	2021	Western Journal of Emergency Medicine	22	3	552-560	10.5811/westjem.2021.1.49279	Lowe 2021	Irrelevant	Excluded by TI/AB review
2433	Posttraumatic stress disorder symptom trajectories within the first year following emergency [illegible]	Lowe, S. R.; Ratanatharathorn, A.; Lai, B. S.; van der Mei, W.; Barbano, A. C.; Bryant, R. A.; Delahanty, D. L.; Matsuoka, Y. J.; [illegible]	2021	Psychological Medicine	51	7	1129-1139	10.1017/S0033291719004008	Lowe 2021	Irrelevant	Excluded by TI/AB review
2436	Supporting gender identity and sexual orientation diversity in K-12 schools	Lytle, Megan C.; Sprott, Richard A.	2021	Perspectives on sexual orientation and gender diversity	NA	NA	NA	10.1037/0000211-000	Lytle 2021	Irrelevant	Excluded by TI/AB review

S4 Table. All studies identified in the literature search

2437	A critical ethnographic study of discriminatory social practice during clinical practice in [illegible]	Maake, T. N.; Millar, B. T.; Christopher, L. D.; Naidoo, N.	2021	BMC Health Serv Res	21	1	787	10.1186/s12913-021-06829-y	Maake 2021	Irrelevant	Excluded by TI/AB review
2439	Characteristics and Healthcare Burden of Patients with Schizophrenia Treated in a US Integrated [illegible]	Mahabaleshwarkar, R.; Lin, D. E.; Joshi, K.; Fishman, J.; Blair, T.; Hetherington, T.; Palmer, P.; Patel, C.; [illegible]	2021	Journal of Mental Health Policy and Economics	24	2	47-59	NA	Mahabaleshwarkar 2021	Irrelevant	Excluded by TI/AB review
2440	Differential associations of hand nicotine and urinary cotinine with children's exposure to tobacco smoke and clinical outcomes	Mahabee-Gittens, E. M.; Merianos, A. L.; Jandarov, R. A.; Quintana, P. J. E.; Hoh, E.; Matt, G. E.	2021	Environmental Research	202	NA	NA	10.1016/j.envres.2021.111722	Mahabee-Gittens 2021	Irrelevant	Excluded by TI/AB review
2442	Assessing the Impact of Hospital Dismissal Summary Readability on Patient Outcomes Following [illegible]	Manka, M. G.; Viers, B. R.; Bole, R.; Nichols, P. E.; Boorjian, S. A.; Tollefson, M. K.; Linder, B. J.	2021	Urology	NA	NA	NA	10.1016/j.urology.2021.06.040	Manka 2021	Irrelevant	Excluded by TI/AB review

S4 Table. All studies identified in the literature search

2443	Refusal of emergency medical care: An analysis of patients who left without being [illegible]	Marco, C. A.; Bryant, M.; Landrum, B.; Drerup, B.; Weeman, M.	2021	American Journal of Emergency Medicine	40	NA	115-119	10.1016/j.ajem.2019.158490	Marco 2021	Irrelevant	Excluded by TI/AB review
2444	Caregiver Depressive Symptoms and Primary Medication Nonadherence in Children With [illegible]	Margolis, R. H. F.; Bellin, M. H.; Morphew, T.; Tsoukleris, M.; Bollinger, M. E.; Butz, A.	2021	Journal of Pediatric Health Care	NA	NA	NA	10.1016/j.pedhc.2021.01.005	Margolis 2021	Irrelevant	Excluded by TI/AB review
2445	Use of the spatial access ratio to measure geospatial access to emergency general surgery services in California	McCrum, M. L.; Wan, N.; Lizotte, S. L.; Han, J.; Varghese, T.; Nirula, R.	2021	J Trauma Acute Care Surg	90	5	853-860	10.1097/ta.0000000000003087	McCrum 2021	Irrelevant	Excluded by TI/AB review
2446	Elderly black non-hispanic patients with head and neck squamous cell cancer have the worst survival outcomes	McDermott, J. D.; Eguchi, M.; Morgan, R.; Amini, A.; Goddard, J. A.; Borrayo, E. A.; Karam, S. D.	2021	JNCCN Journal of the National Comprehensive Cancer Network	19	1	57-67	10.6004/JNCCN.2020.7607	McDermott 2021	Irrelevant	Excluded by TI/AB review

S4 Table. All studies identified in the literature search

2447	First Nations members' emergency department experiences in Alberta: a qualitative study	McLane, P.; Bill, L.; Barnabe, C.	2021	CJEM	23	1	63-74	10.1007/s43678-020-00009-3	McLane 2021	Irrelevant	Excluded by TI/AB review
2450	Representation matters: An assessment of diversity in current major textbooks on burn care	McLawhorn, M. M.; Travis, T. E.; Tejiram, S.; Shupp, J. W.; Johnson, L. S.	2021	Journal of Burn Care and Research	42	SUPPL 1	S155	10.1093/jbcr/irab032.255	McLawhorn 2021	Irrelevant	Excluded by TI/AB review
2451	605 Representation Matters: An Assessment of Diversity in Current Major Textbooks on Burn Care	McLawhorn, Melissa M.; Travis, Taryn E.; Tejiram, Shawn; Shupp, Jeffrey W.; Johnson, Laura S.	2021	Journal of Burn Care & Research	42	NA	S155-S155	10.1093/jbcr/irab032.255	McLawhorn 2021	Irrelevant	Excluded by TI/AB review

S4 Table. All studies identified in the literature search

2452	Assault-related concussions: Visit characteristics and outcomes	Means, M.; Corwin, D. J.; Fein, J.; Arbogast, K. B.; Master, C. L.; Myers, R.	2021	Pediatrics	147	3	94-95	10.1542/peds.147.3-MeetingAbstract.94	Means 2021	Irrelevant	Excluded by TI/AB review
2453	Pancreatic Cancer Surgery Following Emergency Department Admission: Understanding [illegible]	Mehta, V. V.; Friedmann, P.; McAuliffe, J. C.; Muscarella, P.; In, H.	2021	Journal of Gastrointestinal Surgery	25	5	1261-1270	10.1007/s11605-020-04614-6	Mehta 2021	Irrelevant	Excluded by TI/AB review
2454	COVID-19: A catalyst for change in telehealth service delivery for opioid use disorder management	Mehtani, N. J.; Ristau, J. T.; Snyder, H.; Surlyn, C.; Eveland, J.; Smith-Bernardin, S.; Knight, K. R.	2021	Subst Abus	42	2	205-212	10.1080/08897077.2021.1890676	Mehtani 2021	Irrelevant	Excluded by TI/AB review

S4 Table. All studies identified in the literature search

2458	Complex Patients Have More Emergency Visits: Don't Punish the Systems That Serve Them	Mick, E. O.; Alcusky, M. J.; Li, N. C.; Eanet, F. E.; Allison, J. J.; Kiefe, C. I.; Ash, A. S.	2021	Medical Care	59	4	362-367	10.1097/MLR.0000000000001515	Mick 2021	Irrelevant	Excluded by TI/AB review

S4 Table. All studies identified in the literature search

2459	Multicultural presentation of chest pain at an emergency department in Australia	Middleton, Paul M.; Wu, Tammy L. L.; Lee, Riccardo Yih,ÄêNan; Ren, Shiquan; McLaws, Mary,ÄêLouise	2021	Emergency Medicine Australasia	33	3	508-516	10.1111/1742-6723.13681	Middleton 2021	Irrelevant	Excluded by TI/AB review
2460	The Emergency Nurses Association: 50 Years of Advocacy and Advancement	Milbrath, Gwyneth; Snyder, Audrey	2021	JEN: Journal of Emergency Nursing	47	2	224-224	10.1016/j.jen.2020.11.007	Milbrath 2021	Irrelevant	Excluded by TI/AB review

S4 Table. All studies identified in the literature search

2461	RACE-IT ‚Äì Rapid Acute Coronary Syndrome Exclusion using	Miller, J.; Cook, B.; Singh-Kucukarslan, G.; Tang, A.; Danagoulian, S.; Heath, G.; Khalifa,	2021	Contemporary Clinical Trials Communications	22	NA	NA	10.1016/j.conctc.2021.100773	Miller 2021	Irrelevant	Excluded by TI/AB review
2462	The impact of the affordable care act on eye-related emergency department utilization in the	Mir, T. A.; Mehta, S.; Qiang, K.; Freedman, I.; Chow, J.	2021	Investigative Ophthalmology and Visual Science	62	8	NA	NA	Mir 2021	Irrelevant	Excluded by TI/AB review
2463	St Andrew's COVID-19 surgery safety study: hand trauma	Miranda, B. H.; Zberea, D. E.; Pinto-Lopes, R.; Zweifel, C. J.; Sierakowski, A.; Sood, M. K.	2021	Annals of the Royal College of Surgeons of England	103	2	96-103	10.1308/rcsann.2020.7011	Miranda 2021	Irrelevant	Excluded by TI/AB review
2464	Community-Level Characteristics of Timely PCP Follow-Up Care and Post-Discharge ER	Mitchell, J.; Li, X.; Decker, P. J.	2021	Hosp Top	NA	NA	8-Jan	10.1080/00185868.2021.1955061	Mitchell 2021	Irrelevant	Excluded by TI/AB review
2465	Eating disorder measures in a sample of military veterans: A focus on gender, age, and race/ethnicity	Mitchell, Karen S.; Masheb, Robin; Smith, Brian N.; Kehle-Forbes, Shannon; Hardin, Sabrina; Vogt,	2021	Psychological Assessment	NA	NA	NA	10.1037/pas0001050 10.1037/pas0001050.supp (Supplemental)	Mitchell 2021	Irrelevant	Excluded by TI/AB review

S4 Table. All studies identified in the literature search

2466	Comparison of Peripapillary Capillary Density in Glaucoma Patients of African and European Descent	Moghimi, S.; Zangwill, L. M.; Hou, H.; Wong, B.; Proudfoot, J.; Penteado, R. C.; Ekici, E.; Bowd, C.; Weinreb, R. N.	2021	Ophthalmology Glaucoma	4	1	51-62	10.1016/j.ogla.2020.07.005	Moghimi 2021	Irrelevant	Excluded by TI/AB review
2467	The association between patient experience and healthcare outcomes using SEER-CAHPS patient experience and outcomes [illegible]	Mohan, C. S.; Rotter, J. S.; Tan, H. J.; Kent, E.; Bjurlin, M. A.; Basch, E.; Samuel, C.; Nielsen, M.; Smith, A. B.	2021	Journal of Geriatric Oncology	12	4	623-631	10.1016/j.jgo.2020.11.006	Mohan 2021	Irrelevant	Excluded by TI/AB review
2468	A comprehensive review of prioritised interventions to improve the health and wellbeing of persons with lived experience of [illegible]	Moledina, Aliza; Magwood, Olivia; Agbata, Eric; Hung, Jui,ÄêHsia; Saad, Ammar; Thavorn, Kednapa; Pottie, Kevin	2021	Campbell Systematic Reviews	17	2	1-136	10.1002/cl2.1154	Moledina 2021	Irrelevant	Excluded by TI/AB review

S4 Table. All studies identified in the literature search

2470	Qualitative study of people who use methamphetamine during COVID-19 informing future emergency department risk [illegible]	Morse, S. C.; Huynh, L.; Fockele, C.; Zatzick, A.; Whiteside, L.	2021	Academic Emergency Medicine	28	SUPP L 1	S103	10.1111/acem.14249	Morse 2021	Irrelevant	Excluded by TI/AB review
2471	Association of Race and Ethnicity with Sedation Management in	Natale, J. E.; Asaro, L. A.; Joseph, J. G.; Ulysse, C.; Ascenzi, J.; Bowens, C.;	2021	Ann Am Thorac Soc	18	1	93-102	10.1513/AnnalsATS.201912-872OC	Natale 2021	Irrelevant	Excluded by TI/AB review
2473	Improving emergency department discharge instruction readability	Neren, D. R.; Davidson, Z.	2021	Academic Emergency Medicine	28	SUPP L 1	S238	10.1111/acem.14249	Neren 2021	Irrelevant	Excluded by TI/AB review
2474	Transition of care for patients from the emergency department with opioid use disorder who receive buprenorphine	Nguyen, L.; Jablonowski, K.; Hallgren, K.; Taniguchi, D.; Whiteside, L.	2021	Academic Emergency Medicine	28	SUPP L 1	S379	10.1111/acem.14249	Nguyen 2021	Irrelevant	Excluded by TI/AB review

S4 Table. All studies identified in the literature search

2475	Reducing the Emergency Department Revolving Door Syndrome for the Poor, Uninsured, [illegible]	Noulaheu, S. F.	2021	Population Health Managemen t	24	3	376-384	10.1089/po p.2020.003 6	Noulaheu 2021	Irrelevant	Excluded by TI/AB review
2476	Editors‚Äô Note and Special Communication: Research Priorities in Child and Adolescent Mental Health Emerging [illegible]	Novins, D. K.; Stoddard, J.; Althoff, R. R.; Charach, A.; Cortese, S.; Cullen, K. R.; Frazier, J. A.; Glatt, S. J.; Henderson, S. W.; [illegible]	2021	Journal of the American Academy of Child and Adolescent Psychiatry	60	5	544-554.e8	10.1016/j.ja ac.2021.03. 005	Novins 2021	Irrelevant	Excluded by TI/AB review
2477	Eating disorder symptomatology in transgender patients: Differences across gender identity and gender affirmation	Nowaskie, Dustin Z.; Filipowicz, Andrew T.; Choi, Yena; Fogel, Janine M.	2021	International Journal of Eating Disorders	54	8	1493-1499	10.1002/eat. 23539	Nowaskie 2021	Irrelevant	Excluded by TI/AB review
2478	Remote dielectric sensing vs chest X-ray in dyspnea	O'Boyle, K.; Hoang, B.; Sekhon, N.; McArthur, R.; Peacock, F.; Rafique, Z.	2021	Academic Emergency Medicine	28	SUPP L 1	S108	10.1111/ace m.14249	O'Boyle 2021	Irrelevant	Excluded by TI/AB review

S4 Table. All studies identified in the literature search

2479	Use of social simulation to advance health equity and improve physician well-being	Okubanjo, O.; Rehman, T.	2021	Academic Emergency Medicine	28	SUPPL 1	S412-S413	10.1111/acem.14253	Okubanjo 2021	Irrelevant	Excluded by TI/AB review
2480	Are Caregivers Of Pediatric Patients With Asthma Willing To Use Telemedicine To Prevent Emergency Room [illegible]	Padam, J.	2021	Journal of Allergy and Clinical Immunology	147	2	AB51	10.1016/j.jaci.2020.12.212	Padam 2021	Irrelevant	Excluded by TI/AB review
2482	Operational processes to reduce inequities of care in the evaluation of chest pain	Parnell, S.; McDonald, S. A.; Diercks, D. B.; Courtney, D. M.; Mayo, M.; Faidley, R.	2021	Academic Emergency Medicine	28	SUPPL 1	S120	10.1111/acem.14249	Parnell 2021	Irrelevant	Excluded by TI/AB review

S4 Table. All studies identified in the literature search

2483	The effect of a lay health worker intervention on acute care use, patient experiences and end-oflife care: Results from [illegible]	Patel, M. I.; Smith, K.; Khateeb, S.; Park, D. J.	2021	Journal of Clinical Oncology	39	15 SUPPL	NA	10.1200/JCO.2021.39.15_suppl.1508	Patel 2021	Irrelevant	Excluded by TI/AB review
2484	Out-of-hospital COVID-19 deaths: Consequences for quality of medical care and accuracy of cause of death coding	Pathak, E. B.; Garcia, R. B.; Menard, J. M.; Salemi, J. L.	2021	Oncology Research	111	S2	S101-S106	10.2105/AJPH.2021.306428	Pathak 2021	Irrelevant	Excluded by TI/AB review
2485	Developing an outlook on other realities: Innovative curricula opening doors to vulnerable patient populations	Pavlic, A. M.; Sonnenberg, T.; Russell, S.; Kahn, S.; Kenzie, M.; Crowe, C.; Lipanot, K.	2021	Academic Emergency Medicine	28	SUPPL 1	S412	10.1111/acem.14253	Pavlic 2021	Irrelevant	Excluded by TI/AB review

S4 Table. All studies identified in the literature search

2486	Racial Disparities in End-of-Life Care Between Black and White Adults With Metastatic Cancer	Perry, L. M.; Walsh, L. E.; Horswell, R.; Miele, L.; Chu, S.; Melancon, B.; Lefante, J.; Blais, C. M.; Rogers, J. L.; [illegible]	2021	Journal of Pain and Symptom Management	61	2	342-+	10.1016/j.jpainsymman.2020.09.017	Perry 2021	Irrelevant	Excluded by TI/AB review
2487	43 COVID-19 in Patients With Cancer	Pettit, N.; Bischof, J. J.; Kline, J.	2021	Annals of Emergency Medicine	78	2	S22	10.1016/j.annemergmed.2021.07.044	Pettit 2021	Irrelevant	Excluded by TI/AB review
2488	Racial disparities in preventable adverse events attributed to poor care coordination: A national study of older adults in the u.s	Pinheiro, L.; Reshetnyak, E.; Safford, M.; Kern, L. M.	2021	Journal of General Internal Medicine	36	SUPPL 1	S73	10.1007/s11606-021-06830-5	Pinheiro 2021	Irrelevant	Excluded by TI/AB review

S4 Table. All studies identified in the literature search

2489	Racial Disparities in Preventable Adverse Events Attributed to Poor Care Coordination Reported in a [illegible]	Pinheiro, L. C.; Reshetnyak, E.; Safford, M. M.; Kern, L. M.	2021	Medical Care	59	10	901-906	10.1097/MLR.0000000000001623	Pinheiro 2021	Irrelevant	Excluded by TI/AB review
2491	Randomized control trial of postpartum visits at 2 and 6 weeks	Pluym, I. D.; Tandel, M. D.; Kwan, L.; Mok, T.; Holliman, K.; Afshar, Y.; Rao, R.	2021	American Journal of Obstetrics and Gynecology MFM	3	4	NA	10.1016/j.ajogmf.2021.100363	Pluym 2021	Irrelevant	Excluded by TI/AB review
2492	Association of limited English proficiency and increased pediatric emergency department revisits	Portillo, E. N.; Stack, A. M.; Monuteaux, M. C.; Curt, A.; Perron, C.; Lee, L. K.	2021	Acad Emerg Med	28	9	1001-1011	10.1111/acem.14359	Portillo 2021	Irrelevant	Excluded by TI/AB review
2496	Preventing Emergency Department Visits for Children With Medical Complexity Through Ambulatory Care: A Systematic [illegible]	Pulcini, C. D.; Coller, R. J.; Houtrow, A. J.; Belardo, Z.; Zorc, J. J.	2021	Academic Pediatrics	21	4	605-616	10.1016/j.acap.2021.01.006	Pulcini 2021	Irrelevant	Excluded by TI/AB review

S4 Table. All studies identified in the literature search

2497	Nonfatal firearm injuries: Utilization and expenditures for children pre- and postinjury	Pulcini, C. D.; Goyal, M. K.; Hall, M.; Gruhler De Souza, H.; Chaudhary, S.; Alpern, E. R.; Fein, J. A.; Fleegler, E. W.	2021	Academic Emergency Medicine	28	8	840-847	10.1111/acem.14318	Pulcini 2021	Irrelevant	Excluded by TI/AB review

S4 Table. All studies identified in the literature search

2498	30EMF COVID-19 Infection Experiences and Social Determinants of Health in North Carolina: A Qualitative Analysis	Purakal, J. D.; Seidenfeld, J.; Tupetz, A.; Vissoci, J.; Silva, L.; Limkakeng, A.; Fiorino, C.; Phillips, A.; Staton, C.	2021	Annals of Emergency Medicine	78	2	S15	10.1016/j.annemergmed.2021.07.031	Purakal 2021	Irrelevant	Excluded by TI/AB review
2499	Diversity and Inclusion in Case Management: Representing and Advocating for All [illegible]	Quick, Ed; Mann, Chikita; Kurland, MaryBeth	2021	Professional case management	26	4	214-216	10.1097/NCM.0000000000000514	Quick 2021	Irrelevant	Excluded by TI/AB review

S4 Table. All studies identified in the literature search

2500	Review article: Implicit bias towards Aboriginal and Torres Strait Islander patients within Australian emergency departments	Quigley, A.; Hutton, J.; Phillips, G.; Dreise, D.; Mason, T.; Garvey, G.; Paradies, Y.	2021	Emerg Med Australas	33	1	18-Sep	10.1111/1742-6723.13691	Quigley 2021	Irrelevant	Excluded by TI/AB review
2503	Decoupling Crisis Response from Policing - A Step Toward Equitable Psychiatric Emergency Services	Rafla-Yuan, Eric; Chhabra, Divya K.; Mensah, Michael O.	2021	New England Journal of Medicine	384	18	1769-1773	10.1056/NEJMms2035710	Rafla-Yuan 2021	Irrelevant	Excluded by TI/AB review
2504	Psychological distress among medically complex veterans with a recent emergency department visit	Ramos, Katherine; Shepherd-Banigan, Megan E.; Stechuchak, Karen M.; Coffman, Cynthia; Oddone, Eugene Z.; Van Houtven, Courtney; Hendrix, Cristina C.; Mahanna, Elizabeth P.; Hastings, Susan Nicole	2021	Psychological services	NA	NA	NA	10.1037/ser0000437	Ramos 2021	Irrelevant	Excluded by TI/AB review

S4 Table. All studies identified in the literature search

2505	Attention-Deficit/Hyperactivity Disorder Medication Adherence in the Transition to Adulthood: Associated Adverse Outcomes for Females and Other Disparities	Rao, K.; Carpenter, D. M.; Campbell, C. I.	2021	Journal of Adolescent Health	NA	NA	NA	10.1016/j.jadohealth.2021.04.025	Rao 2021	Irrelevant	Excluded by TI/AB review
2506	Pediatric non-urgent emergency department visits and prior care-seeking at primary care	Ravi, N.; Gitz, K. M.; Burton, D. R.; Ray, K. N.	2021	BMC Health Services Research	21	1	466	10.1186/s12913-021-06480-7	Ravi 2021	Irrelevant	Excluded by TI/AB review

S4 Table. All studies identified in the literature search

2507	Understanding Variation In Nonurgent Pediatric Emergency Department Use In Communities With Concentrated Disadvantage	Ray, K. N.; Yahner, K. A.; Bey, J.; Martin, K. C.; Planey, A. M.; Culyba, A. J.; Miller, E.	2021	Health Affairs	40	1	156-164	10.1377/hlthaff.2020.00675	Ray 2021	Irrelevant	Excluded by TI/AB review
2508	Secondary Traumatic Stress in Emergency Services Systems (STRESS) Project: Quantifying and [illegible]	Renkiewicz, G. K.; Hubble, M. W.	2021	Prehospital Emergency Care	NA	NA	NA	10.1080/10903127.2021.1943578	Renkiewicz 2021	Irrelevant	Excluded by TI/AB review
2509	Perceptions of public health consequences of marijuana legalization	Resko, Stella M.; Szechy, Kathryn A.; Early, Theresa J.; Ellis, Jennifer D.; Hicks, Danielle; Agius, Elizabeth	2021	Addiction Research & Theory	29	3	255-262	10.1080/16066359.2020.1823971	Resko 2021	Irrelevant	Excluded by TI/AB review
2510	Massive transfusion protocols in the netherlands: Consensus or confusion?	Rijnhout, T. W. H.; Noorman, F.; Van Waes, O.; Van Bohemen, M.; Verhofstad, M.; Hoencamp, R.	2021	Transfusion	61	SUPPL 3	168A	10.1111/trf.16623	Rijnhout 2021	Irrelevant	Excluded by TI/AB review

S4 Table. All studies identified in the literature search

2511	Demography of the National Emergency Medical Services Workforce: A Description of Those Providing Patient Care in the Prehospital Setting	Rivard, Madison K.; Cash, Rebecca E.; Mercer, Christopher B.; Chrzan, Kirsten; Panchal, Ashish R.	2021	Prehospital Emergency Care	25	2	213-220	10.1080/10903127.2020.1737282	Rivard 2021	Irrelevant	Excluded by TI/AB review
2512	Buprenorphine use and disparities in access among emergency department patients with opioid use disorder: A cross-sectional study	Robbins, M.; Haroz, R.; Mazzarelli, A.; Clements, D.; Jones, C. W.; Salzman, M.	2021	Journal of Substance Abuse Treatment	130	NA	NA	10.1016/j.jsat.2021.108405	Robbins 2021	Irrelevant	Excluded by TI/AB review

S4 Table. All studies identified in the literature search

2516	54 The Rapid Evaluation of COVID-19 Vaccination in Emergency Departments for Underserved Patients Study	Rodriguez, R.; Torres, J.; Chang, A.; Haggins, A.; Caldwell, M.; Miller, D.; Wilkerson, G.; O'Laughlin, K.; Chinnock, B.; Lim, S.; Eswaran, V.	2021	Annals of Emergency Medicine	78	2	S28	10.1016/j.annemergmed.2021.07.056	Rodriguez 2021	Irrelevant	Excluded by TI/AB review
2517	The Rapid Evaluation of COVID-19 Vaccination in Emergency Departments for Underserved Patients Study	Rodriguez, R. M.; Torres, J. R.; Chang, A. M.; Haggins, A. N.; Eucker, S. A.; O'Laughlin, K. N.; Anderson, E.; Miller, D. G.; Wilkerson, R. G.; Caldwell, M.; Lim, S. C.; Raja, A. [illegible]	2021	Annals of Emergency Medicine	78	4	502-510	10.1016/j.annemergmed.2021.05.026	Rodriguez 2021	Irrelevant	Excluded by TI/AB review
2518	Evidence-based heatstroke management in the emergency department	Rublee, C.; Dresser, C.; Giudice, C.; Lemery, J.; Sorensen, C.	2021	Western Journal of Emergency Medicine	22	2	186-195	10.5811/WESTJEM.2020.11.49007	Rublee 2021	Irrelevant	Excluded by TI/AB review

S4 Table. All studies identified in the literature search

2519	Using community-based participatory research methods to inform care for patients experiencing homelessness: An [illegible]	Ryus, C. R.; Yang, D.; Tsai, J.; Meldrum, J.; Ngaruiya, C.	2021	AEM Educ Train	5	Suppl 1	S121-s125	10.1002/aet2.10681	Ryus 2021	Irrelevant	Excluded by TI/AB review
2520	Emergency Department Use Among Student-Run Free Clinic Patients: a Cross-[illegible]	Sack, D. E.; Chakravarthy, R.; Gerhart, C. R.; Fowler, M. J.; Miller, R. F.; Weaver, E. O.; [illegible]	2021	Journal of General Internal Medicine	36	3	830-832	10.1007/s11606-020-05743-z	Sack 2021	Irrelevant	Excluded by TI/AB review
2521	Ward vs. Emergency department discharge in patients with COVID-19: Does [illegible]	Saigal, A.; Shah, A. J.; Naidu, S. B.; Brown, J.; Goldring, J. G.; Sood, T.; Lipman, M.; Hurst, J. R.; Mandal, S.	2021	Thorax	76	SUPPL 1	A182	10.1136/thorax-2020-BTSabstracts.316	Saigal 2021	Irrelevant	Excluded by TI/AB review
2523	Ward vs. emergency department discharge in patients with COVID-19: Does it make a difference to [illegible]	Saigal, A.; Shah, A. J.; Naidu, S. B.; Brown, J.; Goldring, J. G.; Sood, T.; Lipman, M.; Hurst, J. R.; Mandal, S.	2021	Gut	70	SUPPL 1	A182	10.1136/thorax-2020-BTSabstracts.316	Saigal 2021	Irrelevant	Excluded by TI/AB review

S4 Table. All studies identified in the literature search

2524	Reduced rate of postpartum readmissions among homeless compared with non-homeless women in New York: A population-based study using serial, cross-sectional data	Sakai-Bizmark, R.; Kumamaru, H.; Estevez, D.; Neman, S.; Bedel, L. E. M.; Mena, L. A.; Marr, E. H.; Ross, M. G.	2021	BMJ Quality and Safety	NA	NA	NA	10.1136/bmjqs-2020-012898	Sakai-Bizmark 2021	Irrelevant	Excluded by TI/AB review
2525	Evaluating the Contribution of Patient-Provider Communication and Cancer Diagnosis to [illegible]	Samuel-Ryals, C. A.; Mbah, O. M.; Hinton, S. P.; Cross, S. H.; Reeve, B. B.; Dusetzina, S. B.	2021	J Gen Intern Med	NA	NA	NA	10.1007/s11606-021-06778-6	Samuel-Ryals 2021	Irrelevant	Excluded by TI/AB review
2528	Incident strokes among american indian individuals with atrial fibrillation	Sanchez, J. M.; Jolly, S. E.; Dewland, T. A.; Tseng, Z. H.; Nah, G.; Vittinghoff, E.; Marcus, G. M.	2021	Journal of the American Heart Association	10	6	NA	10.1161/JAHA.120.019581	Sanchez 2021	Irrelevant	Excluded by TI/AB review

S4 Table. All studies identified in the literature search

2529	Adapting the neurology resident clinic workflow in a large urban hospital during the COVID-19 Pandemic: Did it [illegible]	Sanghani, N.; Hillen, M.	2021	Neurology	96	15 SUPPL 1	NA	NA	Sanghani 2021	Irrelevant	Excluded by TI/AB review
2530	Impact of a National Quality Collaborative on Pediatric Asthma Care Quality by Insurance Status	Schechter, S. B.; Pantell, M. S.; Parikh, K.; Nkoy, F.; McCulloh, R.; Fassl, B.; Kaiser, S. V.	2021	Academic Pediatrics	21	6	1018-1024	10.1016/j.acap.2021.02.009	Schechter 2021	Irrelevant	Excluded by TI/AB review
2531	Employing computational linguistics techniques to identify limited patient health [illegible]	Schillinger, D.; Balyan, R.; Crossley, S. A.; McNamara, D. S.; Liu, J. Y.; Karter, A. J.	2021	Health Services Research	56	1	132-144	10.1111/1475-6773.13560	Schillinger 2021	Irrelevant	Excluded by TI/AB review
2533	Data from emergency medicine palliative care access (EMPallA): a randomized controlled trial comparing the effectiveness of specialty outpatient	Schmucker, A. M.; Flannery, M.; Cho, J.; Goldfeld, K. S.; Grudzen, C.; Blaum, C.; Bischof, J.; Ouchi, K.; Elie, M. C.; Swor, R.; Jubanyik, K.; Berger, J. T.; Chakravarthy, R.; Cooper, R. J.;	2021	BMC Emergency Medicine	21	1	NA	10.1186/s12873-021-00478-4	Schmucker 2021	Irrelevant	Excluded by TI/AB review

S4 Table. All studies identified in the literature search

2535	Self-reported health and life satisfaction in older emergency department patients: sociodemographic, disease-related and care-specific [illegible]	Schneider, A.; Riedlinger, D.; Pigorsch, M.; Holzinger, F.; Deutschbein, J.; Keil, T.; M√∂ckel, M.; Schenk, L.	2021	BMC Public Health	21	1	1440	10.1186/s12889-021-11439-8	Schneider 2021	Irrelevant	Excluded by TI/AB review
2536	Development of a Logic Model to Guide Implementation and Evaluation of a Mobile Integrated Health [illegible]	Seidl, K. L.; Gingold, D. B.; Stryckman, B.; Landi, C.; Sokan, O.; Fletcher, M.; Marcozzi, D.	2021	Population Health Management	24	2	275-281	10.1089/pop.2020.0038	Seidl 2021	Irrelevant	Excluded by TI/AB review
2537	Racial disparities in length of stay after ostomy creation and the role of peri-operative ostomy education	Shao, C.; McLeod, M. C.; Smithson, M.; Theiss, L.; Gunnells, D.; Hollis, R. H.; Chu, D. I.	2021	Diseases of the Colon and Rectum	64	5	187	10.1097/DCR.0000000000002029	Shao 2021	Irrelevant	Excluded by TI/AB review

S4 Table. All studies identified in the literature search

2539	Building bridges not walls: Developing a hospital-based transitional substance use disorder clinic	Sharma, S.; Suzuki, J.; Hardenstine, R.; Monteiro, C.; Takayoshi, K.	2021	American Journal on Addictions	30	3	245	10.1111/ajad.13173	Sharma 2021	Irrelevant	Excluded by TI/AB review
2540	Impact of guideline adherence and race on asthma control in children	Sheikh, S. I.; Ryan-Wenger, N. A.; Pitts, J.; Britt, R.; Paul, G.; Ulrich, L.	2021	World Journal of Pediatrics	NA	NA	NA	10.1007/s12519-021-00458-5	Sheikh 2021	Irrelevant	Excluded by TI/AB review
2541	Delays in Care for Patients with Symptomatic Cholelithiasis: Differences By Race/Ethnicity	Shenoy, R.; Jackson, N.; Zingmond, D. S.; Maggard Gibbons, M. A.; Russell, M. M.	2021	Journal of the American College of Surgeons	233	5	S128-S129	10.1016/j.jamcollsurg.2021.07.247	Shenoy 2021	Irrelevant	Excluded by TI/AB review
2542	Emergency department use among patients with COPD managed in an accredited center at [illegible]	Singh, M.; Hsu, E.; Zaidan, M. F.; Nishi, S. P.; Chen, S.; McCrumb, T.; Bryant, J.; Sharma, G.; Duarte, A. G.	2021	American Journal of Respiratory and Critical Care Medicine	203	9	NA	10.1164/ajrccm-conference.2021.203.1_MeetingAbstracts.A1[illegible]	Singh 2021	Irrelevant	Excluded by TI/AB review

S4 Table. All studies identified in the literature search

2543	Development of a health equity journal club to address health care disparities and improve cultural competence among	Smith, K. J.; Harris, E. M.; Albazzaz, S.; Carter, M. A.	2021	AEM Educ Train	5	Suppl 1	S57-s64	10.1002/aet2.10675	Smith 2021	Irrelevant	Excluded by TI/AB review
2545	Ileostomy patients using patient engagement technology have shorter length of	Smithson, M.; McLeod, M. C.; Theiss, L.; Shao, C.; Kennedy, G. D.; Hollis, R. H.; Chu,	2021	Diseases of the Colon and Rectum	64	5	235-236	10.1097/DCR.0000000000002029	Smithson 2021	Irrelevant	Excluded by TI/AB review
2546	Sex and race differences in safety and effectiveness of the HEART pathway	Snavely, A. C.; Hendley, N.; Stopyra, J. P.; Lenoir, K. M.; Wells, B. J.; Herrington, D. M.; Hiestand, B. C.;	2021	American Heart Journal	232	NA	125-136	10.1016/j.ahj.2020.11.005	Snavely 2021	Irrelevant	Excluded by TI/AB review
2547	Patient factors associated with improved post-hospitalization discharge care and lower emergency department returns	Solnick, R. E.; Bozaan, D.; Jenq, G.; Li, Y.; Vijayasiri, G.; Kocher, K. E.	2021	Academic Emergency Medicine	28	SUPPL 1	S55	10.1111/acem.14249	Solnick 2021	Irrelevant	Excluded by TI/AB review

S4 Table. All studies identified in the literature search

2548	Association of Gun Violence With Emergency Department Visits for Stress-Responsive Complaints	South, E. C.; Stillman, K.; Buckler, D. G.; Wiebe, D.	2021	Annals of Emergency Medicine	77	5	469-478	10.1016/j.annemergmed.2020.10.014	South 2021	Irrelevant	Excluded by TI/AB review
2549	79. Impact of COVID-19 on Access to Eating Disorder Treatment	Spigel, R. T.; Milliren, C.; Vitagliano, J.; Woods, E. R.; Forman, S.; Richmond, T. K.	2021	Journal of Adolescent Health	68	2	S42	10.1016/j.jadohealth.2020.12.088	Spigel 2021	Irrelevant	Excluded by TI/AB review

S4 Table. All studies identified in the literature search

2550	Should the CIWA-Ar be the standard monitoring strategy for alcohol withdrawal syndrome in the intensive care unit?	Steel, T. L.; Giovanni, S. P.; Katsandres, S. C.; Cohen, S. M.; Stephenson, K. B.; Murray, B.; Sobeck, H.; Hough, C. L.; Bradley, K. A.; Williams, E. C.	2021	Addiction science & clinical practice	16	1	21	10.1186/s13722-021-00226-w	Steel 2021	Irrelevant	Excluded by TI/AB review
2552	Association of Cryoprecipitate Use with Survival after Major Trauma in Children Receiving Massive [illegible]	Tama, M. A.; Stone, M. E.; Blumberg, S. M.; Reddy, S. H.; Conway, E. E.; Meltzer, J. A.	2021	JAMA Surgery	156	5	453-460	10.1001/jamasurg.2020.7199	Tama 2021	Irrelevant	Excluded by TI/AB review

S4 Table. All studies identified in the literature search

2553	Restraint use in the emergency department: Racial, ethnic, and language disparities	Tartak, J. A. C.; Brisbon, N.; Wilkie, S.; Sequist, T.; Aisiku, I. P.; Raja, A. S.; Macias-Konstantopoulos, W.	2021	Academic Emergency Medicine	28	SUPP L 1	S195-S196	10.1111/acem.14249	Tartak 2021	Irrelevant	Excluded by TI/AB review

S4 Table. All studies identified in the literature search

2554	Associations Between Socioeconomic Context and Congenital Heart Disease Related Outcomes in Adolescents and Adults	Tillman, A. R.; Colborn, K. L.; Scott, K. A.; Davidson, A. J.; Khanna, A.; Kao, D.; McKenzie, L.; Ong, T.; Rausch, C. M.; Duca, L. M.; Daley, M. F.; Coleman, S.; Costa, [illegible]	2021	American Journal of Cardiology	139	NA	105-115	10.1016/j.amjcard.2020.10.040	Tillman 2021	Irrelevant	Excluded by TI/AB review
2555	Contemporary issues in population health: Disability, pain management, and opioid overdose in [illegible]	Townsend, Tarlise N.	2021	NA	82	NA	NA	NA	Townsend 2021	Irrelevant	Excluded by TI/AB review
2557	Established evidence-based treatment guidelines help mitigate disparities in quality of emergency care	Trent, S. A.; George, N.; Havranek, E. P.; Ginde, A. A.; Haukoos, J. S.	2021	Academic Emergency Medicine	28	9	1051-1060	10.1111/acem.14235	Trent 2021	Irrelevant	Excluded by TI/AB review

S4 Table. All studies identified in the literature search

2558	Primary Care-Based Case Conferences and Reductions in Health Care Utilization	Vest, J. R.; Blackburn, J.; Yeager, V. A.; Haut, D. P.; Halverson, P. K.	2021	Journal of Health Care for the Poor and Underserved	32	3	1288-1300	10.1353/hpu.2021.0132	Vest 2021	Irrelevant	Excluded by TI/AB review
2559	Radiology education among emergency medicine	Villa, S. E.; Wheaton, N.; Lai, S.; Jordan, J.	2021	Western Journal of Emergency Medicine	22	5	1110-1116	10.5811/WESTJEM.2021.6.52470	Villa 2021	Irrelevant	Excluded by TI/AB review
2560	Vulnerability: A concept synthesis and its application to the Emergency Department	Wardrop, R.; Crilly, J.; Ranse, J.; Chaboyer, W.	2021	International emergency nursing	54	NA	100936	10.1016/j.ienj.2020.100936	Wardrop 2021	Irrelevant	Excluded by TI/AB review
2562	Foundations III: A shared, open access emergency medicine senior resident curriculum	Wheaton, N.	2021	Academic Emergency Medicine	28	SUPPL 1	S402	10.1111/acem.14253	Wheaton 2021	Irrelevant	Excluded by TI/AB review
2563	Factors Associated With Emergency Department Visits	Williamson, L. E.; Evans, C. J.; Cripps, R. L.; Leniz, J.;	2021	Journal of the American	22	10	2046-2055.e35	10.1016/j.jamda.2021.06.012	Williamson 2021	Irrelevant	Excluded by TI/AB review

S4 Table. All studies identified in the literature search

2564	DEPRESSION MEDIATES THE EFFECT OF SOCIAL ISOLATION ON	Willock, R. J.; Yan, F.; Quarells, R. C.; Collins, S.; Levy, P.	2021	Journal of the American College of Cardiology	77	18	795	10.1016/S0735-1097(21)02154-9	Willock 2021	Irrelevant	Excluded by TI/AB review
2565	Successful conduct of an	Yamal, J. M.; Parker, S. A.; Jacob, A. P.;	2021	PLoS One	16	1-Jan	NA	10.1371/journal.pone.02	Yamal 2021	Irrelevant	Excluded by TI/AB review
2566	092 Proposed Mechanisms of Erectile Function Improvement with Low Intensity Shockwave Therapy: Vascular [illegible]	Yih, J.; Minton, J.; Gagnon, C.; Goldstein, S.; Goldstein, I.	2021	Journal of Sexual Medicine	18	3	S49	10.1016/j.jsxm.2021.01.062	Yih 2021	Irrelevant	Excluded by TI/AB review
2567	Improving quality in a complex primary care system,ÄîAn example of refugee care and literature review	Young, R. A.; Nelson, M. J.; Castellon, R. E.; Martin, C. M.	2021	Journal of Evaluation in Clinical Practice	27	5	1018-1026	10.1111/jep.13430	Young 2021	Irrelevant	Excluded by TI/AB review
2568	Development and Validation of a Model to Predict Posttraumatic Stress Disorder and Major Depression after a [illegible]	Ziobrowski, H. N.; Kennedy, C. J.; Ustun, B.; House, S. L.; Beaudoin, F. L.; An, X.; Zeng, D.; Bollen, K. A.; Petukhova, M.; [illegible]	2021	JAMA Psychiatry	NA	NA	NA	10.1001/jamapsychiatry.2021.2427	Ziobrowski 2021	Irrelevant	Excluded by TI/AB review

S4 Table. All studies identified in the literature search

2569	A Unique Approach to Quality Improvement Within the Stroke System of Care [illegible]	Zusevics, K. L.; Kaemmerer, N.; Lang, J.; Link, J.; Bluma, D.	2021	Health Promotion Practice	22	2	224-235	10.1177/15248399198 94305	Zusevics 2021	Irrelevant	Excluded by TI/AB review
2570	Poor guideline adherence in a real-world evaluation of hypertensive emergency	Posen, A.; Benken, S.; Kaluzna, S. D.; Sabouni, M.; Miglo, J.; Cai, J.; Gimbar, R. P.	2022	American Journal of Emergency Medicine	51	NA	46-52	10.1016/j.ajem.2021.09.073	Posen 2022	Irrelevant	Excluded by TI/AB review
2573	Discovery of grounded theory: Strategies for qualitative research	Glaser, B. G.; Strauss, A. L.	2017	Discovery of Grounded Theory: Strategies [illegible]	NA	NA	271	10.4324/97802037932 06	Glaser 2017	Irrelevant	Excluded by TI/AB review
2574	NA	NA	2007	National Patient Safety Goals	NA	NA	NA	NA	NA	Irrelevant	Excluded by TI/AB review

S4 Table. All studies identified in the literature search

2575	NA	NA	2010	Maintenance of Certification: Competencies and Criteria	NA	NA	NA	NA	NA	Irrelevant	Excluded by TI/AB review
2576	NA	NA	2011	Consumer Assessment of Healthcare Providers and Systems	NA	NA	NA	NA	NA	Irrelevant	Excluded by TI/AB review
2577	QSR International Pty Ltd. Version 11	NA	2015	QSR International Pty Ltd	NA	NA	NA	NA	NA	Irrelevant	Excluded by TI/AB review
2578	NA	NA	2016	2011 Emergency Department Summary	NA	NA	NA	NA	NA	Irrelevant	Excluded by TI/AB review

S4 Table. All studies identified in the literature search

2579	NA	NA	2020	NA	NA	NA	NA	NA	NA	Irrelevant	Excluded by TI/AB review
2580	Strategies to reduce diagnostic errors: A systematic review	Abimanyi-Ochom, J.; Bohingamu Mudiyanselage, S.; Catchpool, M.; Firipis, M.; Wanni Arachchige Dona, S.; [illegible]	2019	BMC Medical Informatics and Decision Making	19	1	NA	10.1186/s12911-019-0901-1	Abimanyi-Ochom 2019	Irrelevant	Excluded by TI/AB review
2581	Using human factors and systems engineering to	Acher, A. W.; Lecaire, T. J.; Hundt, A. S.; Greenberg, C. C.; Carayon, P.;	2015	Journal of the American College of	221	4	810-820	10.1016/j.jamcollsurg.2015.06.014	Acher 2015	Irrelevant	Excluded by TI/AB review
2582	Racial disparities in intravenous recombinant tissue plasminogen activator use persist at primary [illegible]	Aparicio, H. J.; Carr, B. G.; Kasner, S. E.; Kallan, M. J.; Albright, K. C.; Kleindorfer, D. O.; Mullen, M. T.	2015	Journal of the American Heart Association	4	10	NA	10.1161/JAHA.115.001877	Aparicio 2015	Irrelevant	Excluded by TI/AB review
2583	Seeing Race and Seeming Racist? Evaluating Strategic Colorblindness in [illegible]	Apfelbaum, E. P.; Sommers, S. R.; Norton, M. I.	2008	Journal of Personality and Social Psychology	95	4	918-932	10.1037/a0011990	Apfelbaum 2008	Irrelevant	Excluded by TI/AB review

S4 Table. All studies identified in the literature search

2584	Unhealthy interactions: The role of stereotype threat in health disparities	Aronson, J.; Burgess, D.; Phelan, S. M.; Juarez, L.	2013	American Journal of Public Health	103	1	50-56	10.2105/AJPH.2012.300828	Aronson 2013	Irrelevant	Excluded by TI/AB review
2586	Structural racism and health inequities in the USA: evidence and interventions	Bailey, Z. D.; Krieger, N.; Ag√©nor, M.; Graves, J.; Linos, N.; Bassett, M. T.	2017	The Lancet	389	10077	1453-1463	10.1016/S0140-6736(17)30569-X	Bailey 2017	Irrelevant	Excluded by TI/AB review
2587	It takes patience and persistence to get negative feedback about patients' experiences: A secondary analysis of national inpatient survey data	Barron, D. N.; West, E.; Reeves, R.; Hawkes, D.	2014	BMC Health Services Research	14	1	NA	10.1186/1472-6963-14-153	Barron 2014	Irrelevant	Excluded by TI/AB review

S4 Table. All studies identified in the literature search

2588	Are physicians' attitudes of respect accurately perceived by patients and [illegible]	Beach, M. C.; Roter, D. L.; Wang, N. Y.; Duggan, P. S.; Cooper, L. A.	2006	Patient Education and Counseling	62	3	347-354	10.1016/j.pec.2006.06.004	Beach 2006	Irrelevant	Excluded by TI/AB review
2590	Coordinating care - A perilous journey through the health care system	Bodenheimer, T.	2008	New England Journal of Medicine	358	10	1064	10.1056/NEJMhpr0706165	Bodenheimer 2008	Irrelevant	Excluded by TI/AB review
2591	Better communication in the emergency department	Burley, D.	2011	Emergency Nurse	19	2	32-36	10.7748/en2011.05.19.2.32.c8509	Burley 2011	Irrelevant	Excluded by TI/AB review
2592	The disconfirmation paradigm: Throughput times and emergency department patient satisfaction	Cassidy-Smith, T. N.; Baumann, B. M.; Boudreaux, E. D.	2007	Journal of Emergency Medicine	32	1	13-Jul	10.1016/j.jemermed.2006.05.028	Cassidy-Smith 2007	Irrelevant	Excluded by TI/AB review

S4 Table. All studies identified in the literature search

2593	Physicians and implicit bias: How doctors may unwittingly perpetuate health care disparities	Chapman, E. N.; Kaatz, A.; Carnes, M.	2013	Journal of General Internal Medicine	28	11	1504-1510	10.1007/s11606-013-2441-1	Chapman 2013	Irrelevant	Excluded by TI/AB review
2594	How to achieve health equity	Chin, M. H.	2014	New England Journal of Medicine	371	24	2331-2332	10.1056/NEJMe1412264	Chin 2014	Irrelevant	Excluded by TI/AB review
2595	Meaningful disparities reduction through research and translation programs	Chin, M. H.; Goldmann, D.	2011	JAMA - Journal of the American Medical Association	305	4	404-405	10.1001/jama.2011.26	Chin 2011	Irrelevant	Excluded by TI/AB review
2596	Time to start using evidence-based approaches to patient engagement	Chou, C.	2018	NEJM Catal	NA	NA	NA	NA	Chou 2018	Irrelevant	Excluded by TI/AB review
2597	Delving below the surface: Understanding how race and	Cooper, L. A.; Beach, M. C.; Johnson, R. L.; Inui, T. S.	2006	Journal of General Internal Medicine	21	SUPPL. 1	S21-S27	10.1111/j.1525-1497.2006.00305.x	Cooper 2006	Irrelevant	Excluded by TI/AB review

S4 Table. All studies identified in the literature search

2600	Measuring patients' perceptions of communication with healthcare providers: Do differences in demographic and [illegible]	DeVoe, J. E.; Wallace, L. S.; Fryer Jr, G. E.	2009	Health Expectations	12	1	70-80	10.1111/j.1369-7625.2008.00516.x	DeVoe 2009	Irrelevant	Excluded by TI/AB review
2601	Health care disconnect: gaps in coverage and care for minority adults. Findings from the [illegible]	Doty, M. M.; Holmgren, A. L.	2006	Issue brief (Commonwealth Fund)	21	NA	12-Jan	10.2510/386220	Doty 2006	Irrelevant	Excluded by TI/AB review
2602	The Flexner report - 100 years later	Duffy, T. P.	2011	Yale Journal of Biology and Medicine	84	3	269-276	NA	Duffy 2011	Irrelevant	Excluded by TI/AB review

S4 Table. All studies identified in the literature search

2603	The association of emergency department crowding during treatment for acute coronary syndrome with subsequent posttraumatic stress disorder symptoms	Edmondson, D.; Shimbo, D.; Ye, S.; Wyer, P.; Davidson, K. W.	2013	JAMA Internal Medicine	173	6	472-474	10.1001/jamainternmed.2013.2536	Edmondson 2013	Irrelevant	Excluded by TI/AB review
2604	Communication in emergency medicine: Implications for patient safety	Eisenberg, E. M.; Murphy, A. G.; Sutcliffe, K.; Wears, R.; Schenkel, S.; Perry, S.; Vanaerhoef, M.	2005	Communication Monographs	72	4	390-413	10.1080/03637750500322602	Eisenberg 2005	Irrelevant	Excluded by TI/AB review

S4 Table. All studies identified in the literature search

2605	Differences in physicians' verbal and nonverbal communication with black and white patients at the end of life	Elliott, A. M.; Alexander, S. C.; Mescher, C. A.; Mohan, D.; Barnato, A. E.	2016	Journal of Pain and Symptom Managemen t	51	1	8-Jan	10.1016/j.jpainsymman.2015.07.008	Elliott 2016	Irrelevant	Excluded by TI/AB review
2606	Adjusting for subgroup differences in extreme response tendency in ratings [illegible]	Elliott, M. N.; Haviland, A. M.; Kanouse, D. E.; Hambarsoomian, K.; Hays, R. D.	2009	Health Services Research	44	2P1	542-561	10.1111/j.1475-6773.2008.00922.x	Elliott 2009	Irrelevant	Excluded by TI/AB review
2607	Effects of survey mode, patient mix, and nonresponse on CAHPS ¬Æ hospital survey scores: Methods	Elliott, M. N.; Zaslavsky, A. M.; Goldstein, E.; Lehrman, W.; Hambarsoomians, K.; Beckett, M. K.; Giordano, L.	2009	Health Services Research	44	2P1	501-518	10.1111/j.1475-6773.2008.00914.x	Elliott 2009	Irrelevant	Excluded by TI/AB review

S4 Table. All studies identified in the literature search

2608	The OPTION scale: Measuring the extent that clinicians involve patients in decision-making tasks	Elwyn, G.; Hutchings, H.; Edwards, A.; Rapport, F.; Wensing, M.; Cheung, W. Y.; Grol, R.	2005	Health Expectations	8	1	34-42	10.1111/j.1369-7625.2004.00311.x	Elwyn 2005	Irrelevant	Excluded by TI/AB review
2609	Implementing shared decision making in the NHS	Elwyn, G.; Laitner, S.; Coulter, A.; Walker, E.; Watson, P.; Thomson, R.	2010	BMJ (Online)	341	7780	971-972	10.1136/bmj.c5146	Elwyn 2010	Irrelevant	Excluded by TI/AB review
2610	Emergency Department Communication Links and Patterns	Fairbanks, R. J.; Bisantz, A. M.; Sunm, M.	2007	Annals of Emergency Medicine	50	4	396-406	10.1016/j.annemergmed.2007.03.005	Fairbanks 2007	Irrelevant	Excluded by TI/AB review
2611	Assessing patient perceptions of hospitalist communication skills using the [illegible]	Ferranti, D. E.; Makoul, G.; Forth, V. E.; Rauworth, J.; Lee, J.; Williams, M. V.	2010	Journal of Hospital Medicine	5	9	522-527	10.1002/jhm.787	Ferranti 2010	Irrelevant	Excluded by TI/AB review

S4 Table. All studies identified in the literature search

2612	Patient trust: Is it related to patient-centered behavior of primary care physicians?	Fiscella, K.; Meldrum, S.; Franks, P.; Shields, C. G.; Duberstein, P.; McDaniel, S. H.; [illegible]	2004	Medical Care	42	11	1049-1055	10.1097/00005650-200411000-00003	Fiscella 2004	Irrelevant	Excluded by TI/AB review
2613	Reports and ratings of care: Black and white medicare enrollees	Fongwa, M. N.; Cunningham, W.; Weech-Maldonado, R.; Gutierrez, P. R.; Hays, R. D.	2008	Journal of Health Care for the Poor and Underserved	19	4	1136-1147	10.1353/hpu.0.0074	Fongwa 2008	Irrelevant	Excluded by TI/AB review
2614	Prediction of risk of death and myocardial infarction in the six months after presentation with acute coronary syndrome: [illegible]	Fox, K. A. A.; Dabbous, O. H.; Goldberg, R. J.; Pieper, K. S.; Eagle, K. A.; Van De Werf, F.; Avezum, √Å; Goodman, S. G.; Flather, M. D.; [illegible]	2006	British Medical Journal	333	7578	1091-1094	10.1136/bmj.38985.646481.55	Fox 2006	Irrelevant	Excluded by TI/AB review
2615	The financial impact of health information exchange on emergency department care	Frisse, M. E.; Johnson, K. B.; Nian, H.; Davison, C. L.; Gadd, C. S.; Unertl, K. M.; Turri, P. A.; Chen, Q.	2012	Journal of the American Medical Informatics Association	19	3	328-333	10.1136/amiajnl-2011-000394	Frisse 2012	Irrelevant	Excluded by TI/AB review

S4 Table. All studies identified in the literature search

2616	Emergency Severity Index (ESI): A Triage Tool for Emergency Department Care, [illegible]	Gilboy, N.; Tanabe, T.; Travers, D.; Rosenau, A. M.	2011	Emergency Severity Index (ESI): A Triage Tool for Emergency [illegible]	NA	NA	NA	NA	Gilboy 2011	Irrelevant	Excluded by TI/AB review
2617	Development, implementation, and public	Giordano, L. A.; Elliott, M. N.; Goldstein, E.;	2010	Medical Care Research	67	1	27-37	10.1177/10775587093 41065	Giordano 2010	Irrelevant	Excluded by TI/AB review
2618	Racial/ethnic differences in patients? Perceptions of inpatient care using the [illegible]	Goldstein, E.; Elliott, M. N.; Lehrman, W. G.; Hambarsoomian, K.; Giordano, L. A.	2010	Medical Care Research and Review	67	1	74-92	10.1177/10775587093 41066	Goldstein 2010	Irrelevant	Excluded by TI/AB review
2619	Accuracy of Staff-Initiated Emergency Department Tracking System Timestamps in [illegible]	Gordon, B. D.; Flottemesch, T. J.; Asplin, B. R.	2008	Annals of Emergency Medicine	52	5	504-511	10.1016/j.annemergmed. 2007.11.036	Gordon 2008	Irrelevant	Excluded by TI/AB review
2620	Racial differences in trust and lung cancer patients' perceptions of physician communication	Gordon, H. S.; Street Jr, R. L.; Sharf, B. F.; Kelly, P. A.; Souchek, J.	2006	Journal of Clinical Oncology	24	6	904-909	10.1200/JCO.2005.03. 1955	Gordon 2006	Irrelevant	Excluded by TI/AB review

S4 Table. All studies identified in the literature search

2621	The patient experience in the emergency department: A systematic synthesis of qualitative research	Gordon, J.; Sheppard, L. A.; Anaf, S.	2010	International Emergency Nursing	18	2	80-88	10.1016/j.ienj.2009.05.004	Gordon 2010	Irrelevant	Excluded by TI/AB review
2622	Approach to the Agitated Emergency Department Patient	Gottlieb, M.; Long, B.; Koyfman, A.	2018	Journal of Emergency Medicine	54	4	447-457	10.1016/j.jemermed.2017.12.049	Gottlieb 2018	Irrelevant	Excluded by TI/AB review
2623	Validating the primary provider theory in emergency medicine	Guarisco, J. S.; Bavin, S. A.	2008	Leadership in Health Services	21	2	120-130	10.1108/17511870810870565	Guarisco 2008	Irrelevant	Excluded by TI/AB review

S4 Table. All studies identified in the literature search

2624	How Many Interviews Are Enough?: An Experiment with Data Saturation [illegible]	Guest, G.; Bunce, A.; Johnson, L.	2006	Field Methods	18	1	59-82	10.1177/1525822X05279903	Guest 2006	Irrelevant	Excluded by TI/AB review
2625	Implicit racial/ethnic bias among health care	Hall, W. J.; Chapman, M. V.; Lee, K. M.; Merino,	2015	American Journal of Public	105	12	e60-e76	10.2105/AJPH.2015.302903	Hall 2015	Irrelevant	Excluded by TI/AB review
2626	Associations between patient and emergency department operational characteristics and patient satisfaction scores in an adult population	Handel, D. A.; French, L. K.; Nichol, J.; Momberger, J.; Fu, R.	2014	Annals of Emergency Medicine	64	6	604-608	10.1016/j.annemergmed.2014.07.451	Handel 2014	Irrelevant	Excluded by TI/AB review
2627	Physician communication and patient adherence to treatment: A meta- [illegible]	Haskard Zolnierek, K. B.; Dimatteo, M. R.	2009	Medical Care	47	8	826-834	10.1097/MLR.0b013e31819a5acc	HaskardZolnierek 2009	Irrelevant	Excluded by TI/AB review

S4 Table. All studies identified in the literature search

2630	Three approaches to qualitative content analysis	Hsieh, H. F.; Shannon, S. E.	2005	Qualitative Health Research	15	9	1277-1288	10.1177/1049732305276687	Hsieh 2005	Irrelevant	Excluded by TI/AB review
2631	The impact of delays to admission from the emergency department on inpatient outcomes	Huang, Q.; Thind, A.; Dreyer, J. F.; Zaric, G. S.	2010	BMC Emergency Medicine	10	NA	NA	10.1186/1471-227X-10-16	Huang 2010	Irrelevant	Excluded by TI/AB review
2632	Using Mixed-Methods Sequential Explanatory Design: From Theory to Practice	Ivankova, N. V.; Creswell, J. W.; Stick, S. L.	2006	Field Methods	18	1	20-Mar	10.1177/1525822X05282260	Ivankova 2006	Irrelevant	Excluded by TI/AB review
2633	Understanding African Americans' views of the trustworthiness of physicians	Jacobs, E. A.; Rolle, I.; Ferrans, C. E.; Whitaker, E. E.; Warnecke, R. B.	2006	Journal of General Internal Medicine	21	6	642-647	10.1111/j.1525-1497.2006.00485.x	Jacobs 2006	Irrelevant	Excluded by TI/AB review

S4 Table. All studies identified in the literature search

2635	Racial and ethnic differences in patient perceptions of bias and cultural competence in health care	Johnson, R. L.; Saha, S.; Arbelaez, J. J.; Beach, M. C.; Cooper, L. A.	2004	Journal of General Internal Medicine	19	2	101-110	10.1111/j.1525-1497.2004.30262.x	Johnson 2004	Irrelevant	Excluded by TI/AB review
2637	Knowledge is not power for patients: A systematic review and thematic synthesis of patient-reported barriers and [illegible]	Joseph-Williams, N.; Elwyn, G.; Edwards, A.	2014	Patient Education and Counseling	94	3	291-309	10.1016/j.pec.2013.10.031	Joseph-Williams 2014	Irrelevant	Excluded by TI/AB review
2638	Dissemination and Implementation of Shared Decision Making Into Clinical Practice: A Research Agenda	Kanzaria, H. K.; Booker-Vaughns, J.; Itakura, K.; Yadav, K.; Kane, B. G.; Gayer, C.; Lin, G.; LeBlanc, A.; Gibson, R.; Chen, E. H.; Williams, P.; Carpenter, C. R.	2016	Academic Emergency Medicine	23	12	1368-1379	10.1111/acem.13075	Kanzaria 2016	Irrelevant	Excluded by TI/AB review
2639	Emergency physician perceptions of shared decision-	Kanzaria, H. K.; Brook, R. H.; Probst, M. A.; Harris, D.; Berry, S. H.;	2015	Academic Emergency Medicine	22	4	399-405	10.1111/acem.12627	Kanzaria 2015	Irrelevant	Excluded by TI/AB review

S4 Table. All studies identified in the literature search

2640	Health disparities by race and class: Why both matter	Kawachi, I.; Daniels, N.; Robinson, D. E.	2005	Health Affairs	24	2	343-352	10.1377/hlthaff.24.2.343	Kawachi 2005	Irrelevant	Excluded by TI/AB review
2641	Use and avoidance of seclusion and restraint: Consensus statement of the American Association for emergency psychiatry project [illegible]	Knox, D. K.; Holloman Jr, G. H.	2012	Western Journal of Emergency Medicine	13	1	35-40	10.5811/westjem.2011.9.6867	Knox 2012	Irrelevant	Excluded by TI/AB review
2642	ED overcrowding is associated with an increased frequency of medication errors	Kulstad, E. B.; Sikka, R.; Sweis, R. T.; Kelley, K. M.; Rzechula, K. H.	2010	American Journal of Emergency Medicine	28	3	304-309	10.1016/j.ajem.2008.12.014	Kulstad 2010	Irrelevant	Excluded by TI/AB review
2644	Do patient autonomy preferences matter? Linking	Lee, Y. Y.; Lin, J. L.	2010	Social Science and Medicine	71	10	1811-1818	10.1016/j.socscimed.2010.08.008	Lee 2010	Irrelevant	Excluded by TI/AB review
2645	Not all patients want to participate in decision making:A national	Levinson, W.; Kao, A.; Kuby, A.; Thisted, R. A.	2005	Journal of General Internal Medicine	20	6	531-535	10.1111/j.1525-1497.2005.04101.x	Levinson 2005	Irrelevant	Excluded by TI/AB review

S4 Table. All studies identified in the literature search

2648	Socially-Assigned Race, Healthcare Discrimination and Preventive Healthcare Services	MacIntosh, T.; Desai, M. M.; Lewis, T. T.; Jones, B. A.; Nunez-Smith, M.	2013	PLoS ONE	8	5	NA	10.1371/journal.pone.0064522	MacIntosh 2013	Irrelevant	Excluded by TI/AB review
2649	A decade of studying implicit racial/ethnic bias in healthcare providers using the implicit [illegible]	Maina, I. W.; Belton, T. D.; Ginzberg, S.; Singh, A.; Johnson, T. J.	2018	Social Science and Medicine	199	NA	219-229	10.1016/j.socscimed.2017.05.009	Maina 2018	Irrelevant	Excluded by TI/AB review
2650	Measuring patient views of physician communication skills: Development and testing of the Communication Assessment Tool	Makoul, G.; Krupat, E.; Chang, C. H.	2007	Patient Education and Counseling	67	3 SPEC. ISS.	333-342	10.1016/j.pec.2007.05.005	Makoul 2007	Irrelevant	Excluded by TI/AB review
2651	Preference for same-race health care providers and perceptions of interpersonal discrimination in [illegible]	Malat, J.; Hamilton, M. A.	2006	Journal of Health and Social Behavior	47	2	173-187	10.1177/002214650604700206	Malat 2006	Irrelevant	Excluded by TI/AB review

S4 Table. All studies identified in the literature search

2652	Effectiveness of five-level emergency severity index triage system compared with [illegible]	Maleki, M.; Fallah, R.; Riahi, L.; Delavari, S.; Rezaei, S.	2015	Arch Trauma Res	4	4	NA	NA	Maleki 2015	Irrelevant	Excluded by TI/AB review
2653	The patient experience and health outcomes	Manary, M. P.; Boulding, W.; Staelin, R.; Glickman, S. W.	2013	New England Journal of Medicine	368	3	201-203	10.1056/NEJMp1211775	Manary 2013	Irrelevant	Excluded by TI/AB review
2655	Interventions for improving patients' trust in doctors and groups of doctors	McKinstry, B.; Ashcroft, R. E.; Car, J.; Freeman, G. K.; Sheikh, A.	2006	Cochrane database of systematic reviews (Online)	3	NA	NA	NA	McKinstry 2006	Irrelevant	Excluded by TI/AB review
2656	Development and Testing of Shared Decision Making Interventions for Use in Emergency Care: A Research Agenda	Melnick, E. R.; Probst, M. A.; Schoenfeld, E.; Collins, S. P.; Breslin, M.; Walsh, C.; Kuppermann, N.; Dunn, P.; Abella, B. S.; Boatright, D.; Hess, E. P.	2016	Academic Emergency Medicine	23	12	1346-1353	10.1111/acem.13045	Melnick 2016	Irrelevant	Excluded by TI/AB review

S4 Table. All studies identified in the literature search

2657	Patient perspectives on communication with the medical team: Pilot study using the communication [illegible]	Mercer, L. M.; Tanabe, P.; Pang, P. S.; Gisondi, M. A.; Courtney, D. M.; Engel, K. G.; Donlan, S. M.; Adams, J. G.; [illegible]	2008	Patient Education and Counseling	73	2	220-223	10.1016/j.pec.2008.07.003	Mercer 2008	Irrelevant	Excluded by TI/AB review
2658	Benefits of 'observer effects': Lessons from the field	Monahan, T.; Fisher, J. A.	2010	Qualitative Research	10	3	357-376	10.1177/1468794110362874	Monahan 2010	Irrelevant	Excluded by TI/AB review
2660	Stop the silent misdiagnosis: Patients' preferences matter	Mulley, A. G.; Trimble, C.; Elwyn, G.	2012	BMJ (Online)	345	7883	NA	10.1136/bmj.e6572	Mulley 2012	Irrelevant	Excluded by TI/AB review
2661	Using the communication Assessment Tool in family medicine residency programs	Myerholtz, L.; Simons, L.; Felix, S.; Nguyen, T.; Brennan, J.; Rivera-Tovar, A.; Martin, P.; Hepworth, J.; Makoul, G.	2010	Family Medicine	42	8	567-573	NA	Myerholtz 2010	Irrelevant	Excluded by TI/AB review

S4 Table. All studies identified in the literature search

2662	The patient experience in emergency departments: A review of the literature	Nairn, S.; Whotton, E.; Marshal, C.; Roberts, M.; Swann, G.	2004	Accident and Emergency Nursing	12	3	159-165	10.1016/j.aaen.2004.04.001	Nairn 2004	Irrelevant	Excluded by TI/AB review
2663	A Modern-Day Fight Club? The Stabilization and Management of Acutely Agitated Patients in the Emergency Department	New, A.; Tucci, V. T.; Rios, J.	2017	Psychiatric Clinics of North America	40	3	397-410	10.1016/j.psc.2017.05.002	New 2017	Irrelevant	Excluded by TI/AB review
2664	Health literacy in the "oral exchange": An important element of patient-provider communication	Nouri, S. S.; Rudd, R. E.	2015	Patient Education and Counseling	98	5	565-571	10.1016/j.pec.2014.12.002	Nouri 2015	Irrelevant	Excluded by TI/AB review

S4 Table. All studies identified in the literature search

2665	Standards for reporting qualitative research: A synthesis of recommendations	O'Brien, B. C.; Harris, I. B.; Beckman, T. J.; Reed, D. A.; Cook, D. A.	2014	Academic Medicine	89	9	1245-1251	10.1097/ACM.0000000000000388	O'Brien 2014	Irrelevant	Excluded by TI/AB review
2666	'Unsatisfactory Saturation': A critical exploration of the notion of saturated sample sizes in qualitative research	O'Reilly, M.; Parker, N.	2013	Qualitative Research	13	2	190-197	10.1177/1468794112446106	O'Reilly 2013	Irrelevant	Excluded by TI/AB review
2667	Beyond a good story: From Hawthorne Effect to reactivity in health professions education research	Paradis, E.; Sutkin, G.	2017	Medical Education	51	1	31-39	10.1111/medu.13122	Paradis 2017	Irrelevant	Excluded by TI/AB review
2669	National Testing of the Emergency Department Patient Experience of Care Discharged to Community Survey and [illegible]	Parast, L.; Mathews, M.; Tolpadi, A.; Elliott, M.; Flow-Delwiche, E.; Becker, K.	2019	Medical Care	57	1	42-48	10.1097/MLR.0000000000001005	Parast 2019	Irrelevant	Excluded by TI/AB review

S4 Table. All studies identified in the literature search

2670	Structured approaches to pain management in the ICU	Pasero, C.; Puntillo, K.; Li, D.; Mularski, R. A.; Grap, M. J.; Erstad, B. L.; Varkey, B.; Gilbert, H. C.; Medina, J.; Sessler, C. N.	2009	Chest	135	6	1665-1672	10.1378/chest.08-2333	Pasero 2009	Irrelevant	Excluded by TI/AB review
2671	Preferences for participation in decision making among ethnically diverse patients with anxiety and depression	Patel, S. R.; Bakken, S.	2010	Community Mental Health Journal	46	5	466-473	10.1007/s10597-010-9323-3	Patel 2010	Irrelevant	Excluded by TI/AB review

S4 Table. All studies identified in the literature search

2672	ED Crowding Is Associated with Variable Perceptions of Care Compromise	Pines, J. M.; Garson, C.; Baxt, W. G.; Rhodes, K. V.; Shofer, F. S.; Hollander, J. E.	2007	Academic Emergency Medicine	14	12	1176-1181	10.1197/j.aem.2007.06.043	Pines 2007	Irrelevant	Excluded by TI/AB review
2673	The Impact of Emergency Department Crowding Measures on Time to Antibiotics for Patients With [illegible]	Pines, J. M.; Localio, A. R.; Hollander, J. E.; Baxt, W. G.; Lee, H.; Phillips, C.; Metlay, J. P.	2007	Annals of Emergency Medicine	50	5	510-516	10.1016/j.annemergmed.2007.07.021	Pines 2007	Irrelevant	Excluded by TI/AB review
2674	Improving continuity of care for frequent users of emergency departments: Service user and provider perspectives	Poremski, D.; Harris, D. W.; Kahan, D.; Pauly, D.; Leszcz, M.; O'Campo, P.; Wasylenki, D.; Stergiopoulos, V.	2016	General Hospital Psychiatry	40	NA	55-59	10.1016/j.genhosppsych.2016.01.004	Poremski 2016	Irrelevant	Excluded by TI/AB review
2675	Do experiences with medicare managed care vary according to the proportion of same-race/ethnicity/lang [illegible]	Price, R. A.; Haviland, A. M.; Hambarsoomian, K.; Dembosky, J. W.; Gaillot, S.; Weech-Maldonado, R.; [illegible]	2015	Health Services Research	50	5	1649-1687	10.1111/1475-6773.12292	Price 2015	Irrelevant	Excluded by TI/AB review

S4 Table. All studies identified in the literature search

2676	Black and hispanic patients receive hospice care similar to that of white patients when in the same [illegible]	Price, R. A.; Parast, L.; Haas, A.; Teno, J. M.; Elliott, M. N.	2017	Health Affairs	36	7	1283-1290	10.1377/hlthaff.2017.0151	Price 2017	Irrelevant	Excluded by TI/AB review
2677	Perceived Appropriateness of Shared Decision-making in the Emergency Department: A Survey Study	Probst, M. A.; Kanzaria, H. K.; Frosch, D. L.; Hess, E. P.; Winkel, G.; Ngai, K. M.; Richardson, L. D.	2016	Academic Emergency Medicine	23	4	375-381	10.1111/acem.12904	Probst 2016	Irrelevant	Excluded by TI/AB review
2678	Shared Decisionmaking in the Emergency Department: A Guiding Framework for Clinicians	Probst, M. A.; Kanzaria, H. K.; Schoenfeld, E. M.; Menchine, M. D.; Breslin, M.; Walsh, C.; Melnick, E. R.; Hess, E. P.	2017	Annals of Emergency Medicine	70	5	688-695	10.1016/j.annemergmed.2017.03.063	Probst 2017	Irrelevant	Excluded by TI/AB review

S4 Table. All studies identified in the literature search

2679	Quantifying Magnitude of Group-Level Differences in Patient Experiences with Health Care	Quigley, D. D.; Elliott, M. N.; Setodji, C. M.; Hays, R. D.	2018	Health Services Research	53	NA	3027-3051	10.1111/1475-6773.12828	Quigley 2018	Irrelevant	Excluded by TI/AB review
2680	Reasons for using the emergency department: Results of the EMPATH Study	Ragin, D. F.; Hwang, U.; Cydulka, R. K.; Holson, D.; Haley Jr, L. L.; Richards, C. F.; Becker, B. M.; Richardson, L. D.	2005	Academic Emergency Medicine	12	12	1158-1166	10.1197/j.aem.2005.06.030	Ragin 2005	Irrelevant	Excluded by TI/AB review
2682	Physician-patient communication-An actionable target for reducing overly aggressive care near the end of life	Robinson, J. D.; Jagsi, R.	2016	JAMA Oncology	2	11	1407-1408	10.1001/jamaoncol.2016.1948	Robinson 2016	Irrelevant	Excluded by TI/AB review
2683	Practical barriers to timely primary care access: Impact on adult use of emergency [illegible]	Rust, G.; Ye, J.; Baltrus, P.; Daniels, E.; Adesunloye, B.; Fryer, G. E.	2008	Archives of Internal Medicine	168	15	1705-1710	10.1001/archinte.168.15.1705	Rust 2008	Irrelevant	Excluded by TI/AB review

S4 Table. All studies identified in the literature search

2684	Factors associated with patients' perceptions of health care providers' [illegible]	Rutten, L. J. F.; Augustson, E.; Wanke, K.	2006	Journal of Health Communication	11	SUPPL. 1	135-146	10.1080/10810730600639596	Rutten 2006	Irrelevant	Excluded by TI/AB review
2685	Care coordination gaps due to lack of interoperability in the United States: A qualitative study and literature review	Samal, L.; Dykes, P. C.; Greenberg, J. O.; Hasan, O.; Venkatesh, A. K.; Volk, L. A.; Bates, D. W.	2016	BMC Health Services Research	16	1	NA	10.1186/s12913-016-1373-y	Samal 2016	Irrelevant	Excluded by TI/AB review
2686	Patients' preference for involvement in medical decision making: A [illegible]	Say, R.; Murtagh, M.; Thomson, R.	2006	Patient Education and Counseling	60	2	102-114	10.1016/j.pec.2005.02.003	Say 2006	Irrelevant	Excluded by TI/AB review
2687	Taking Your Qualitative Research to the Next Level: A Guide for the [illegible]	Schneider, N. C.; Coates, W. C.; Yarris, L. M.	2017	AEM Education and Training	1	4	368-378	10.1002/aet2.10065	Schneider 2017	Irrelevant	Excluded by TI/AB review
2690	The Physician-as-Stakeholder: An Exploratory Qualitative Analysis of Physicians‚Äô Motivations for [illegible]	Schoenfeld, E. M.; Goff, S. L.; Elia, T. R.; Khordipour, E. R.; Poronsky, K. E.; Nault, K. A.; Lindenauer, P. K.; Mazor, K. M.	2016	Academic Emergency Medicine	23	12	1417-1427	10.1111/acem.13043	Schoenfeld 2016	Irrelevant	Excluded by TI/AB review

S4 Table. All studies identified in the literature search

2691	A Qualitative Analysis of Attending Physicians' Use of Shared Decision-Making: Implications for Resident Education	Schoenfeld, E. M.; Goff, S. L.; Elia, T. R.; Khordipour, E. R.; Poronsky, K. E.; Nault, K. A.; Lindenauer, P. K.; Mazor, K. M.	2018	Journal of graduate medical education	10	1	43-50	10.4300/JGME-D-17-00318.1	Schoenfeld 2018	Irrelevant	Excluded by TI/AB review
2692	Development and psychometric properties of the Shared Decision Making Questionnaire - physician version (SDM-Q-Doc)	Scholl, I.; Kriston, L.; Dirmaier, J.; Buchholz, A.; H√§rter, M.	2012	Patient Education and Counseling	88	2	284-290	10.1016/j.pec.2012.03.005	Scholl 2012	Irrelevant	Excluded by TI/AB review
2694	Improving communication of diagnostic radiology findings	Schwartz, L. H.; Panicek, D. M.; Berk, A. R.; Li, Y.; Hricak, H.	2011	Radiology	260	1	174-181	10.1148/radiol.11101913	Schwartz 2011	Irrelevant	Excluded by TI/AB review

S4 Table. All studies identified in the literature search

2696	Does patient-provider racial/ethnic concordance influence ratings of trust in people with HIV [illegible]	Sohler, N. L.; Fitzpatrick, L. K.; Lindsay, R. G.; Anastos, K.; Cunningham, C. O.	2007	AIDS and Behavior	11	6	884-896	10.1007/s10461-007-9212-0	Sohler 2007	Irrelevant	Excluded by TI/AB review
2697	Emergency department patient experience: a systematic review of the literature	Sonis, J. D.; Aaronson, E. L.; Lee, R. Y.; Philpotts, L. L.; White, B. A.	2017	J Patient Exp	5	2	101-106	NA	Sonis 2017	Irrelevant	Excluded by TI/AB review
2698	Interpersonal processes of care survey: Patient-reported measures for diverse groups	Stewart, A. L.; N√°poles-Springer, A. M.; Gregorich, S. E.; Santoyo-Olsson, J.	2007	Health Services Research	42	3 I	1235-1256	10.1111/j.1475-6773.2006.00637.x	Stewart 2007	Irrelevant	Excluded by TI/AB review

S4 Table. All studies identified in the literature search

2699	Do EPs change their clinical behaviour in the hallway or when a companion is present? A cross-sectional survey	Stoklosa, H.; Scannell, M.; Ma, Z.; Rosner, B.; Hughes, A.; Bohan, J. S.	2018	Emergency Medicine Journal	35	7	406-411	10.1136/emermed-2017-207119	Stoklosa 2018	Irrelevant	Excluded by TI/AB review
2700	Reliability and Validity of Scores on the Emergency Severity Index Version 3	Tanabe, P.; Gimbel, R.; Yarnold, P. R.; Kyriacou, D. N.; Adams, J. G.	2004	Academic Emergency Medicine	11	1	59-65	10.1197/j.aem.2003.06.013	Tanabe 2004	Irrelevant	Excluded by TI/AB review
2701	Measuring patients' trust in physicians when assessing quality of care	Thom, D. H.; Hall, M. A.; Pawlson, L. G.	2004	Health Affairs	23	4	124-132	10.1377/hlthaff.23.4.124	Thom 2004	Irrelevant	Excluded by TI/AB review
2702	Patient Satisfaction as a Function of Emergency Department Previsit Expectations	Toma, G.; Triner, W.; McNutt, L. A.	2009	Annals of Emergency Medicine	54	3	360-367.e6	10.1016/j.annemergmed.2009.01.024	Toma 2009	Irrelevant	Excluded by TI/AB review

S4 Table. All studies identified in the literature search

2703	Consolidated criteria for reporting qualitative research (COREQ): A 32-item checklist for [illegible]	Tong, A.; Sainsbury, P.; Craig, J.	2007	International Journal for Quality in Health Care	19	6	349-357	10.1093/intqhc/mzm042	Tong 2007	Irrelevant	Excluded by TI/AB review
2705	How patients' trust relates to their involvement in medical care	Trachtenberg, F.; Dugan, E.; Hall, M. A.	2005	Journal of Family Practice	54	4	344-352	NA	Trachtenberg 2005	Irrelevant	Excluded by TI/AB review
2706	Talking/not talking about race: The enregisterments of culture in higher education discourses	Urciuoli, B.	2009	Journal of Linguistic Anthropology	19	1	21-39	10.1111/j.1548-1395.2009.01017.x	Urciuoli 2009	Irrelevant	Excluded by TI/AB review
2707	Shedding the cobra effect: Problematising thematic emergence, triangulation, saturation and member checking	Varpio, L.; Ajjawi, R.; Monrouxe, L. V.; O'Brien, B. C.; Rees, C. E.	2017	Medical Education	51	1	40-50	10.1111/medu.13124	Varpio 2017	Irrelevant	Excluded by TI/AB review

S4 Table. All studies identified in the literature search

2708	"Sign right here and you're good to go": A content analysis of audiotaped emergency department discharge instructions	Vashi, A.; Rhodes, K. V.	2011	Annals of Emergency Medicine	57	4	315-322.e1	10.1016/j.annemergmed.2010.08.024	Vashi 2011	Irrelevant	Excluded by TI/AB review
2710	Patient Ethnicity Affects Triage Assessments and Patient	Vigil, J. M.; Coulombe, P.; Alcock, J.; Kruger, E.; Stith, S. S.;	2016	Medicine (United States)	95	14	NA	10.1097/MD.0000000000003191	Vigil 2016	Irrelevant	Excluded by TI/AB review
2711	Advancing Health Services Research to Eliminate Health Care Disparities	Wasserman, J.; Palmer, R. C.; Gomez, M. M.; Berzon, R.; Ibrahim, S. A.; Ayanian, J. Z.	2019	American journal of public health	109	S1	S64-S69	10.2105/AJPH.2018.304922	Wasserman 2019	Irrelevant	Excluded by TI/AB review
2712	The impact of judge selection on standard setting for a patient	Wayne, D. B.; Cohen, E.; Makoul, G.; McGaghie, W. C.	2008	Academic medicine : journal of the	83	10 Suppl	S17-20	10.1097/acm.0b013e318183e7bd	Wayne 2008	Irrelevant	Excluded by TI/AB review

S4 Table. All studies identified in the literature search

2713	Are the Uninsured Responsible for the Increase in Emergency Department Visits in the United States?	Weber, E. J.; Showstack, J. A.; Hunt, K. A.; Colby, D. C.; Grimes, B.; Bacchetti, P.; Callaham, M. L.	2008	Annals of Emergency Medicine	52	2	108-115.e1	10.1016/j.annemergmed.2008.01.327	Weber 2008	Irrelevant	Excluded by TI/AB review
2714	Survey response style and differential use of CAHPS rating scales by hispanics	Weech-Maldonado, R.; Elliott, M. N.; Oluwole, A.; Schiller, K. C.; Hays, R. D.	2008	Medical Care	46	9	963-968	10.1097/MLR.0b013e3181791924	Weech-Maldonado 2008	Irrelevant	Excluded by TI/AB review
2715	Language and regional differences in evaluations of medicare managed care by hispanics	Weech-Maldonado, R.; Fongwa, M. N.; Gutierrez, P.; Hays, R. D.	2008	Health Services Research	43	2	552-568	10.1111/j.1475-6773.2007.00796.x	Weech-Maldonado 2008	Irrelevant	Excluded by TI/AB review

S4 Table. All studies identified in the literature search

2716	NA	Weinick, R. M.; Becker, K.; Parast, L.; Stucky, B. D.; Elliott, M. N.; Mathews, M.	2014	Emergency Department Patient Experience of Care Survey: Developme [illegible]	NA	NA	NA	NA	Weinick 2014	Irrelevant	Excluded by TI/AB review
2717	Using standardized encounters to understand reported racial/ethnic disparities in [illegible]	Weinick, R. M.; Elliott, M. N.; Volandes, A. E.; Lopez, L.; Burkhart, Q.; Schlesinger, M.	2011	Health Services Research	46	2	491-509	10.1111/j.1475-6773.2010.01214.x	Weinick 2011	Irrelevant	Excluded by TI/AB review
2718	Estimating the Degree of Emergency Department Overcrowding in [illegible]	Weiss, S. J.; Derlet, R.; Arndahl, J.; Ernst, A. A.; Richards, J.; Fern√°ndez- [illegible]	2004	Academic Emergency Medicine	11	1	38-50	10.1197/j.aem.2003.07.017	Weiss 2004	Irrelevant	Excluded by TI/AB review
2719	Color-Blind and Multicultural Strategies in Medical Settings	West, T. V.; Schoenthaler, A.	2017	Social Issues and Policy Review	11	1	124-158	10.1111/sipr.12029	West 2017	Irrelevant	Excluded by TI/AB review

S4 Table. All studies identified in the literature search

2720	Socially-assigned race and health: A scoping review with global implications for population health equity	White, K.; Lawrence, J. A.; Tchangalova, N.; Huang, S. J.; Cummings, J. L.	2020	International Journal for Equity in Health	19	1	NA	10.1186/s12939-020-1137-5	White 2020	Irrelevant	Excluded by TI/AB review
2721	Communication Patterns in a UK Emergency Department	Woloshynowych, M.; Davis, R.; Brown, R.; Vincent, C.	2007	Annals of Emergency Medicine	50	4	407-413	10.1016/j.annemergmed.2007.08.001	Woloshynowych 2007	Irrelevant	Excluded by TI/AB review
2722	Experiences of Individuals Who Were Physically Restrained in the Emergency Department	Wong, A. H.; Ray, J. M.; Rosenberg, A.; Crispino, L.; Parker, J.; McVaney, C.; Iennaco, J. D.; Bernstein, S. L.; Pavlo, A. J.	2020	JAMA Network Open	3	1	NA	10.1001/jamanetworkopen.2019.19381	Wong 2020	Irrelevant	Excluded by TI/AB review

S4 Table. All studies identified in the literature search

2723	Physical Restraint Use in Adult Patients Presenting to a General Emergency Department	Wong, A. H.; Taylor, R. A.; Ray, J. M.; Bernstein, S. L.	2019	Annals of Emergency Medicine	73	2	183-192	10.1016/j.annemergmed.2018.06.020	Wong 2019	Irrelevant	Excluded by TI/AB review
2724	Racial Code Words, Re-Memberings and Black Kids,Äô Civic [illegible]	Woodson, A. N.	2019	Anthropology and Education Quarterly	50	1	26-47	10.1111/aeq.12277	Woodson 2019	Irrelevant	Excluded by TI/AB review
2725	Achieving health equity: A guide for health care organizations. IHI white paper	Wyatt, R.; Laderman, M.; Botwinick, L.; Mate, K.; Whittington, J.	2016	Achieving Health Equity: A Guide for Health Care Organizations	NA	NA	NA	NA	Wyatt 2016	Irrelevant	Excluded by TI/AB review

S4 Table. All studies identified in the literature search

2726	Examining the presence, consequences, and reduction of implicit bias in health care: A narrative review	Zestcott, C. A.; Blair, I. V.; Stone, J.	2016	Group Processes and Intergroup Relations	19	4	528-542	10.1177/1368430216642029	Zestcott 2016	Irrelevant	Excluded by TI/AB review
2727	Racial Differences in Pediatric Emergency Department Triage Scores	Zook, H. G.; Kharbanda, A. B.; Flood, A.; Harmon, B.; Puumala, S. E.; Payne, N. R.	2016	Journal of Emergency Medicine	50	5	720-727	10.1016/j.jemermed.2015.02.056	Zook 2016	Irrelevant	Excluded by TI/AB review

S4 Table. All studies identified in the literature search

2728	Reasons for emergency room use among U.S. adults aged 18,Äì64: National health interview survey, 2013 and 2014	Gindi, R. M.; Black, L. I.; Cohen, R. A.	2016	National Health Statistics Reports	NA	NA	NA	NA	Gindi 2016	Irrelevant	Excluded by TI/AB review
2729	A Conceptual Framework for Optimizing the Equity of Hospital,ÄêBased Emergency Care: The Structure of [illegible]	HSUAN, CHARLEEN; CARR, BRENDAN G.; VANNESS, DAVID; WANG, YINAN; LESLIE, DOUGLAS L.; [illegible]	2023	Milbank Quarterly	101	1	74-125	10.1111/1468-0009.12609	HSUAN 2023	Irrelevant	Excluded by TI/AB review
2730	A Systematic Review of Characteristics of Students With Emotional Disturbance in Special Education Research.	Chow, Jason C.; Morse, Ashley; Zhao, Hongyang; Kingsbery, Corinne; Murray, Rebecca; Soni, Isha	2023	Remedial & Special Education	44	5	409-422	10.1177/07419325221125890	Chow 2023	Irrelevant	Excluded by TI/AB review

S4 Table. All studies identified in the literature search

2731	Addressing Adultification of Black Pediatric Patients in the Emergency Department: A Framework to Decrease [illegible]	Koch, Amie; Kozhumam, Arthi	2022	Health Promotion Practice	23	4	555-559	10.1177/15248399211049207	Koch 2022	Irrelevant	Excluded by TI/AB review
2732	Addressing Emergency Department Care for Patients Experiencing Incarceration: A Narrative Review.	Armstrong, Rachel E.; Hendershot, Kristopher A.; Newton, Naomi P.; Panakos, Patricia D.	2023	Western Journal of Emergency Medicine: Integrating Emergency Care with Population Health	24	4	654-661	10.5811/westjem.59057	Armstrong 2023	Irrelevant	Excluded by TI/AB review
2733	Addressing healthcare bias in caring for patients with sickle cell disease.	Marr, Christianne; Schmitgal, Daniel; Parsh, Bridget CNS	2022	Nursing	52	3	11-Oct	10.1097/01.NURSE.0000820064.60540.ff	Marr 2022	Irrelevant	Excluded by TI/AB review

S4 Table. All studies identified in the literature search

2734	Addressing racial/ethnic inequities in vaccine hesitancy and uptake: lessons learned from the California [illegible]	AuYoung, Mona; Rodriguez Espinosa, Patricia; Chen, Wei-ting; Juturu, Preeti; Young, Maria-Elena De Trinidad; Casillas, Alejandra; Adkins-[illegible]	2023	Journal of Behavioral Medicine	46	2-Jan	153-166	10.1007/s10865-022-00284-8	AuYoung 2023	Irrelevant	Excluded by TI/AB review
2735	Age,Äêrelated differences in symptoms in older emergency department patients with COVID,Äê19: Prevalence and outcomes in a multicenter cohort.	Goldberg, Elizabeth M.; Southerland, Lauren T.; Meltzer, Andrew C.; Pagenhardt, Justine; Hoopes, Ryan; Camargo, Carlos A.; Kline, Jeffrey A.	2022	Journal of the American Geriatrics Society	70	7	1918-1930	10.1111/jgs.17816	Goldberg 2022	Irrelevant	Excluded by TI/AB review

S4 Table. All studies identified in the literature search

2736	An Outpatient Pain Plan and Emergency Department Pain Pathway for Adults With Sickle Cell Disease.	Mercado, Stephanie Harris	2023	Topics in Pain Management	38	10	5-Jan	10.1097/01.TPM.0000937036.58946.07	Mercado 2023	Irrelevant	Excluded by TI/AB review

S4 Table. All studies identified in the literature search

2737	Analysis of Racial and Ethnic Diversity of Population Served and Imaging Used in US Children's Hospital Emergency Departments.	Samuels-Kalow, Margaret E.; De Souza, Heidi G.; Neuman, Mark I.; Alpern, Elizabeth; Marin, Jennifer R.; Hoffmann, Jennifer; Hall, Matt; Aronson, Paul L.; Peltz, Alon; Wells, Jordee; Gutman, Colleen K.; Simon, Harold K.; Shanahan, Kristen; Goyal, Monika K.	2022	JAMA Network Open	5	6	e2213951-e2213951	10.1001/jamanetworkopen.2022.13951	Samuels-Kalow 2022	Irrelevant	Excluded by TI/AB review
2738	Anti‚Äêracism in the emergency department: Navigating clinician experiences of racism.	NA	2022	Emergency Medicine Australasia	34	1	116-119	10.1111/1742-6723.13916	NA	Irrelevant	Excluded by TI/AB review

S4 Table. All studies identified in the literature search

2739	Assessing the Relationship Between Race, Language, and Surgical Admissions in the Emergency [illegible]	Rigney, Grant H.; Ghoshal, Soham; Mercaldo, Sarah; Cheng, Debby; Parks, Jonathan J.; Velmahos, George C.; Lev, Michael H.; [illegible]	2023	Western Journal of Emergency Medicine: Integrating Emergency Care with [illegible]	24	2	141-148	10.5811/westjem.2022.10.57276	Rigney 2023	Irrelevant	Excluded by TI/AB review
2740	Association of Participation in the Maryland Primary Care Program With COVID-19	Gruber, Emily; Perman, Chad; Grisham, Rachel; Adashi, Eli Y.; Haft, Howard	2023	JAMA Network Open	6	1	e2249791-e2249791	10.1001/jamanetworkopen.2022.49791	Gruber 2023	Irrelevant	Excluded by TI/AB review
2741	Association of Self-reported Primary Care Physician Tolerance for Uncertainty With Variations in Resource Use and [illegible]	Begin, Arabella S.; Hidrue, Michael K.; Lehrhoff, Sara; Lennes, Inga T.; Armstrong, Katrina; Weilburg, Jeffrey B.; del Carmen, Marcela G.; Wasfy, Jason H.	2022	JAMA Network Open	5	9	e2229521-e2229521	10.1001/jamanetworkopen.2022.29521	Begin 2022	Irrelevant	Excluded by TI/AB review
2742	Association of Social Needs and Housing Status Among Urban Emergency Department Patients.	Wormley, Kadia; Dickson, Drusia; Alter, Harrison; Njoku, Ndidi; Imani, Partow; Anderson, Erik S.	2022	Western Journal of Emergency Medicine: Integrating Emergency Care with [illegible]	23	6	802-810	10.5811/westjem.2022.8.55705	Wormley 2022	Irrelevant	Excluded by TI/AB review

S4 Table. All studies identified in the literature search

2743	Building Culturally Competent Compassion in Nurses Caring for Vulnerable [illegible]	Hernandez, Nathalya Casallas; Leal, Liliana Marcela Reina; Brito, Maria Jos√© Menezes	2022	Journal of Holistic Nursing	40	4	359-369	10.1177/08980101211062708	Hernandez 2022	Irrelevant	Excluded by TI/AB review
2745	Care of Bullet-related Injuries: A Cross-sectional Study of Instructions and Prescriptions Provided on Discharge from the Emergency [illegible]	Hayes, Jane M.; Punch, L. J.; Mueller, Kristen L.	2023	Western Journal of Emergency Medicine: Integrating Emergency Care with Population Health	24	2	363-367	10.5811/westjem.2022.11.57574	Hayes 2023	Irrelevant	Excluded by TI/AB review
2746	Centering Equity in Direct Education Curriculum Selection: A New Tool From Washington State SNAP-Ed...Society for [illegible]	Moss, Jen; Schupp, Courtney; Grigsby, Eileen; Markworth, Adrienne	2023	Journal of Nutrition Education & Behavior	55	7	64-64	10.1016/j.jneb.2023.05.141	Moss 2023	Irrelevant	Excluded by TI/AB review

S4 Table. All studies identified in the literature search

2747	Clinical and cost-effectiveness of paramedic administered fascia iliaca compartment block for emergency hip fracture (RAPID 2)-protocol for an individually randomised parallel-group trial.	Kingston, Mark; Jones, Jenna; Black, Sarah; Evans, Bridie; Ford, Simon; Foster, Theresa; Goodacre, Steve; Jones, Marie-Louise; Jones, Sian; Keen, Leigh; Longo, Mirella; Lyons, Ronan A.; Pallister, Ian; Rees, Nigel; Siriwardena, Aloysius Niroshan; Watkins, Alan; Williams, Julia; Wilson, Helen; Snooks, Helen	2022	Trials	23	1	15-Jan	10.1186/s13063-022-06522-3	Kingston 2022	Irrelevant	Excluded by TI/AB review

S4 Table. All studies identified in the literature search

2748	Closing the Gap: A Systematic Review and Meta-Analysis of Enhanced Recovery After Surgery Protocols in Primary Cleft Palate Repair.	Asadourian, Paul A.; Lu Wang, Marcos; Demetres, Michelle R.; Imahiyerobo, Thomas A.; Otterburn, David M.	2023	Cleft Palate Craniofacial Journal	60	10	1230-1240	10.1177/10556656221096631	Asadourian 2023	Irrelevant	Excluded by TI/AB review
2749	Community-Level Characteristics of Timely PCP Follow-Up Care and Post-Discharge ER Usage.	Mitchell, Jordan; Li, Xiao; Decker, Phillip J.	2023	Hospital Topics	101	1	8-Jan	10.1080/00185868.2021.1955061	Mitchell 2023	Irrelevant	Excluded by TI/AB review

S4 Table. All studies identified in the literature search

2750	Comprehensive In-Patient Education Consult for Asthma Exacerbations.	Baker, Joyce A.; Moore, Heather M.; Brinton, John T.; Federico, Monica J.	2022	Respiratory Care	67	6	682-687	10.4187/respcare.08778	Baker 2022	Irrelevant	Excluded by TI/AB review
2751	Considerations for development of child abuse and neglect phenotype with implications for reduction of racial bias: a qualitative study.	Landau, Aviv Y; Blanchard, Ashley; Cato, Kenrick; Atkins, Nia; Salazar, Stephanie; Patton, Desmond U; Topaz, Maxim	2022	Journal of the American Medical Informatics Association	29	3	512-519	10.1093/jamia/ocab275	Landau 2022	Irrelevant	Excluded by TI/AB review
2752	Cops, Clinicians, or Both? Collaborative Approaches to Responding to [illegible]	Balfour, Margaret E.; Hahn Stephenson, Arlene; Delany-Brumsey, Ayesha; Winsky, Jason; [illegible]	2022	Psychiatric Services	73	6	658-669	10.1176/appi.ps.202000721	Balfour 2022	Irrelevant	Excluded by TI/AB review

S4 Table. All studies identified in the literature search

2753	Defining two novel sub models of the Anglo-American paramedic system: A Delphi study.	Makrides, Timothy; Ross, Linda; Gosling, Cameron; Acker, Joseph; O'Meara, Peter	2022	Australasian Emergency Care	25	3	229-234	10.1016/j.auec.2021.11.001	Makrides 2022	Irrelevant	Excluded by TI/AB review
2754	Diabetes, Gaps in Care Coordination, and Preventable Adverse Events.	Colvin, Calvin L.; Akinyelure, Oluwasegun P.; Rajan, Mangala; Safford, Monika M.; Carson, April P.; Muntner, Paul; Colantonio, Lisandro [illegible]	2023	American Journal of Managed Care	29	6	e162-e168	10.37765/ajmc.2023.89374	Colvin 2023	Irrelevant	Excluded by TI/AB review
2755	Disparities Exist in the Application of Low Tidal-volume Ventilation in the Emergency Department.	Self, Michael; Kennis, Brent; Lafree, Andrew; Tainter, Christopher R.; Lopez, Jesus; Malhotra, Atul; [illegible]	2023	Western Journal of Emergency Medicine: Integrating Emergency [illegible]	24	3	502-510	10.5811/westjem.59291	Self 2023	Irrelevant	Excluded by TI/AB review

S4 Table. All studies identified in the literature search

2756	Disparities in Emergency Medical Services Care Delivery in the United States: A Scoping Review.	Farcas, Andra M.; Joiner, Anjni P.; Rudman, Jordan S.; Ramesh, Karthik; Torres, Gilberto; Crowe, Remle P.; Curtis, Travis; Tripp, Rickquel; Bowers, Karen; von Isenburg, Megan; Logan, Robert; Coaxum [illegible]	2023	Prehospital Emergency Care	27	8	1058-1071	10.1080/10903127.2022.2142344	Farcas 2023	Irrelevant	Excluded by TI/AB review
2757	Disparities in Lupus and the Role of Social Determinants of Health: Current State of Knowledge and Directions for Future Research [illegible]	Buie, Joy; McMillan, Emma; Kirby, Jillian; Cardenas, Leigh Ann; Eftekhari, Sanaz; Feldman, Candace H.; Gawuga, Cyrena; Knight, Andrea M.; Lim, S. Sam; [illegible]	2023	ACR Open Rheumatology	5	9	454-464	10.1002/acr2.11590	Buie 2023	Irrelevant	Excluded by TI/AB review
2758	Disparities in Pediatric Emergency Department Length of Stay and Utilization Associated With Primary Language.	Lowe, Jeremiah T. BA; Monteiro, Kristina A.; Zonfrillo, Mark R.; Lowe, Jeremiah T	2022	Pediatric Emergency Care	38	4	e1192-e1197	10.1097/PEC.0000000000002545	Lowe 2022	Irrelevant	Excluded by TI/AB review

S4 Table. All studies identified in the literature search

2759	Does ethnicity affect pain management for people with advanced disease? A mixed methods cross-national systematic review [illegible]	Clarke, Gemma; Chapman, Emma; Crooks, Jodie; Koffman, Jonathan; Ahmed, Shenaz; Bennett, Michael I.	2022	BMC Palliative Care	21	1	11-Jan	10.1186/s12904-022-00923-6	Clarke 2022	Irrelevant	Excluded by TI/AB review
2761	Effect of Health Information Exchange Plus a Care Transitions Intervention on [illegible]	Boockvar, Kenneth S.; Koufacos, Nicholas S.; May, Justine; Schwartzkopf, [illegible]	2022	JGIM: Journal of General Internal Medicine	37	16	4054-4061	10.1007/s11606-022-07397-5	Boockvar 2022	Irrelevant	Excluded by TI/AB review
2762	Eliminating pain disparities for children in the emergency department.	Martin, Sarah R.; Heyming, Theodore; Kain, Alexandra S.; Krauss, Baruch S.; Campos, Belinda	2023	Academic Emergency Medicine	30	10	1075-1077	10.1111/acem.14723	Martin 2023	Irrelevant	Excluded by TI/AB review

S4 Table. All studies identified in the literature search

2763	Emergency department nurses' perceptions of caring behaviors toward individuals with mental illness: A secondary analysis.	McIntosh, Jennifer T.	2023	International Emergency Nursing	68	NA	N.PAG-N.PAG	10.1016/j.ienj.2023.101271	McIntosh 2023	Irrelevant	Excluded by TI/AB review
2764	Emergency Department Use by Children and Youth with Mental Health Conditions: A Health Equity Agenda.	Hoge, Michael A.; Vanderploeg, Jeffrey; Paris Jr., Manuel; Lang, Jason M.; Olezeski, Christy	2022	Community Mental Health Journal	58	7	1225-1239	10.1007/s10597-022-00937-7	Hoge 2022	Irrelevant	Excluded by TI/AB review

S4 Table. All studies identified in the literature search

2765	Emergency Department Use in Black Individuals With Diabetes.	Rovner, Barry; Casten, Robin; Nightingale, Ginah; Leiby, Benjamin E.; Kelley, Megan; Rising, Kristin	2023	Diabetes Spectrum	NA	NA	391-397	10.2337/ds22-0091	Rovner 2023	Irrelevant	Excluded by TI/AB review
2766	Emergency department utilization among patients who receive outpatient specialty care for headache: A retrospective cohort study analysis.	Phelan, Michael P.; Thompson, Nicolas R.; Ahmed, Zubair; Lapin, Brittany; Udeh, Belinda; Anderson, Eric; Katzan, Irene; Walker, Laura E.	2023	Headache: The Journal of Head & Face Pain	63	4	472-483	10.1111/head.14456	Phelan 2023	Irrelevant	Excluded by TI/AB review

S4 Table. All studies identified in the literature search

2767	Emergency Medical Services Prenotification is Associated with Reduced Odds of In-Hospital Mortality in Stroke Patients.	Nielsen, Victoria M.; Song, Glory; DeJoie-Stanton, Claudine; Zachrison, Kori S.	2023	Prehospital Emergency Care	27	5	639-645	10.1080/10903127.2022.2079784	Nielsen 2023	Irrelevant	Excluded by TI/AB review
2768	Enhanced Preoperative Education Pathways: A Step Toward Reducing Disparities in Total Joint Arthroplasty Outcomes.	Turcotte, Justin J.; Brennan, Jane C.; Holbert, S. Elliott; Dolle, Steffanie S.; King, Paul J.	2022	Journal of Arthroplasty	37	7	1233-1233	10.1016/j.arth.2022.03.029	Turcotte 2022	Irrelevant	Excluded by TI/AB review
2769	Enhanced Telemedicine in a Mobile Integrated Health Program to Improve Healthcare Access [illegible]	Somers, Sarah; Fitzpatrick, Suzanna; Hicks, Courtney; Gingold, Daniel B.	2022	Online Journal of Nursing Informatics	26	2	10-Mar	NA	Somers 2022	Irrelevant	Excluded by TI/AB review

S4 Table. All studies identified in the literature search

2770	Ensuring effective intercultural communication in the emergency department.	Tuohy, Dympna; Wallace, Evan	2022	Emergency Nurse	30	4	33-40	10.7748/en.2022.e2122	Tuohy 2022	Irrelevant	Excluded by TI/AB review
2771	Environmental and structural factors driving poor quality of care: An examination of nursing homes [illegible]	Travers, Jasmine L.; Castle, Nicholas; Weaver, Susan H.; Perera, Uduwanage G.; Wu, Bei; Dick, Andrew W.; Stone, [illegible]	2023	Journal of the American Geriatrics Society	71	10	3040-3048	10.1111/jgs.18459	Travers 2023	Irrelevant	Excluded by TI/AB review

S4 Table. All studies identified in the literature search

2772	Essential Principles to Create an Equitable, Inclusive, and Diverse EMS Workforce and Work Environment: A Position Statement and Resource Document.	Owusu-Ansah, Sylvia; Tripp, Rickquel; N. Weisberg, Stacy; P. Mercer, Mary; Whitten-Chung, Kimberly	2023	Prehospital Emergency Care	27	5	552-556	10.1080/10903127.2023.2187103	Owusu-Ansah 2023	Irrelevant	Excluded by TI/AB review
2773	Evaluation of the Emergency Severity Index in US Emergency Departments for the Rate of Mistriage.	Sax, Dana R.; Warton, E. Margaret; Mark, Dustin G.; Vinson, David R.; Kene, Mamata V.; Ballard, Dustin W.; Vitale, Tina J.; McGaughey, [illegible]	2023	JAMA Network Open	6	3	e233404-e233404	10.1001/jamanetworkopen.2023.3404	Sax 2023	Irrelevant	Excluded by TI/AB review

S4 Table. All studies identified in the literature search

2774	Evaluation of the use of individualized patient care plans in frequent emergency department visitors with pain complaints.	Blind, Fred; Melton, James; Karp, Juliana; Oldano, Karen; Homa, Karen; Blanco, Alexandra; Leoni, Reanna; Pazanese, Anthony	2022	International Journal of Emergency Medicine	15	1	7-Jan	10.1186/s12245-022-00440-6	Blind 2022	Irrelevant	Excluded by TI/AB review
2775	Evidence-Based Guidelines for Prehospital Pain Management: Recommendations.	Lindbeck, George; Shah, Manish I.; Braithwaite, Sabina; Powell, Jonathan R.; Panchal, Ashish R.; Browne, Lorin R.; Lang, Eddy S.; Burton, Brooke; Coughenour, Jeffrey; Crowe, Remle P.; Degn, Hannah; Hedges, Mary; Gasper, James; Guild, Kyle; Mattera,	2023	Prehospital Emergency Care	27	2	144-153	10.1080/10903127.2021.2018073	Lindbeck 2023	Irrelevant	Excluded by TI/AB review

S4 Table. All studies identified in the literature search

2776	Examining emergency department inequities in Aotearoa New Zealand: Findings from a national retrospective observational study examining Indigenous emergency care outcomes.	Curtis, Elana; Paine, Sarah,ÄêJane; Jiang, Yannan; Jones, Peter; Tomash, Inia; Healey, Olivia; Reid, Papaarangi	2022	Emergency Medicine Australasia	34	1	16-23	10.1111/1742-6723.13876	Curtis 2022	Irrelevant	Excluded by TI/AB review
2777	Experiences of Transgender Participants in Emergency Departments: Findings from the [illegible]	Thompson-Blum, Daniel N.; Coleman, Todd A.; Phillips, Natalie E.; Richardson, Sean; Travers, Robb; [illegible]	2021	Transgender Health	6	6	358-368	10.1089/trgh.2020.0112	Thompson-Blum 2021	Irrelevant	Excluded by TI/AB review

S4 Table. All studies identified in the literature search

2778	Experiences of UK African-Caribbean prostate cancer survivors of discharge to primary care.	Margariti, C.; Gannon, K.; Thompson, R.; Walsh, J.; Green, J.	2021	Ethnicity & Health	26	8	1115-1129	10.1080/13557858.2019.1606162	Margariti 2021	Irrelevant	Excluded by TI/AB review
2779	Exploring a cross-cultural system-wide Alliance empowering Aboriginal Community Controlled Health [illegible]	Gordon, Stuart; Davis, Bob	NA	International Journal of Integrated Care (IJIC)	22	NA	2-Jan	10.5334/ijic.ICIC22100	NA	Irrelevant	Excluded by TI/AB review
2780	Factors Influencing Healthcare Utilization Among Patients at Three Free Clinics.	Petrilli, J.; Strang, L.; Von Haunalter, E.; Costa, J.; Coughlin, E.; Mhaskar, R.	2022	Journal of Community Health	47	4	604-609	10.1007/s10900-022-01083-7	Petrilli 2022	Irrelevant	Excluded by TI/AB review

S4 Table. All studies identified in the literature search

2781	Fast Track to Oncology From an Emergency Department's Incidental Finding Using Navigation to Address Social Determinants of Health...Academy of Oncology Nurse & Patient	Silva, Jeanne; Lagmay-Fuentes, Portia; Patel, Avni; Gaughran, Amanda; Ferguson, Odalys	2023	Journal of Oncology Navigation & Survivorship	14	10	331-332	NA	Silva 2023	Irrelevant	Excluded by TI/AB review
2782	Frailty, gaps in care coordination, and preventable adverse events.	Akinyelure, Oluwasegun P.; Colvin, Calvin L.; Sterling, Madeline R.; Safford, Monika M.; Muntner, Paul; Colantonio, Lisandro D.; Kern, Lisa M.	2022	BMC Geriatrics	22	1	10-Jan	10.1186/s12877-022-03164-7	Akinyelure 2022	Irrelevant	Excluded by TI/AB review

S4 Table. All studies identified in the literature search

2783	Health Care Utilization in the 6 Months Following SARS-CoV-2 Infection.	Tartof, Sara Y.; Malden, Deborah E.; Liu, In-Lu Amy; Sy, Lina S.; Lewin, Bruno J.; Williams, Joshua T. B.; Hambidge, Simon J.; Alpern, Jonathan D.; Daley, Matthew F.; [illegible]	2022	JAMA Network Open	5	8	e2225657-e2225657	10.1001/jamanetworkopen.2022.25657	Tartof 2022	Irrelevant	Excluded by TI/AB review
2784	Identifying facilitators and barriers to integrated and equitable care for community-dwelling older [illegible]	Kehoe MacLeod, Krystal; Flores, Karyle Nama; Chandra, Kavish	2023	International Journal for Equity in Health	22	1	10-Jan	10.1186/s12939-023-01900-y	KehoeMacLeod 2023	Irrelevant	Excluded by TI/AB review
2785	Identifying Racial Disparity in Utilization and Outcomes of Hip Arthroscopy using Machine Learning...AOSSM 2023 ,Äì [illegible]	Yining Lu; Marigi, Erick; Alder, Kareme; Mickley, John; Camp, Christopher; Levy, Bruce; Krych, Aaron; Okoroha, Kelechi	2023	Orthopaedic Journal of Sports Medicine	11	NA	504-507	10.1177/2325967123S00325	YiningLu 2023	Irrelevant	Excluded by TI/AB review

S4 Table. All studies identified in the literature search

2786	Impact of Sexual Assault Survivor Identity on Patient Care in the Emergency Department.	Chalmers, Kristen; Parameswaran, Ramya; Dussault, Nicole; Farnan, Jeanne; Oyola, Sonia; Carter, Keme	2023	Journal of Interpersonal Violence	38	4-Mar	3244-3278	10.1177/08862605221104522	Chalmers 2023	Irrelevant	Excluded by TI/AB review
2787	Impact of socio-economic status and race on emergency hospital admission outcomes: Analysis from hospital admissions between 2001 and 2012	Davidson, Thomas; Mirza, Farhaan; Baig, Mirza M	2022	Health Services Management Research	35	3	127-133	10.1177/09514848211012189	Davidson 2022	Irrelevant	Excluded by TI/AB review
2788	Improving Electronic Patient Portal Use in Groups with Low Utilization at a Family Medicine Practice.	Saif, Nadia; Blackstone, Sarah R.; Sebring, Amanda; Compton, Rebekah	2022	Online Journal of Nursing Informatics	26	2	15-Apr	NA	Saif 2022	Irrelevant	Excluded by TI/AB review

S4 Table. All studies identified in the literature search

2789	Improving Health Outcomes in Vulnerable Populations: The Medical-Legal Partnership‚ÄîColorado's Experience.	Sauaia, Angela	2022	Journal of Health Care for the Poor & Underserved	33	2	580-589	10.1353/hpu.2022.0048	Sauaia 2022	Irrelevant	Excluded by TI/AB review
2790	Improving Postpartum Care: Identifying Opportunities to Reduce Postpartum Emergency Room [illegible]	Harrell, Taylor; Howell, Elizabeth A.; Balbierz, Amy; Guel, Luz; Pena, Juan; Janevic, Teresa; Gorbenko, Ksenia	2022	Maternal & Child Health Journal	26	4	913-922	10.1007/s10995-021-03282-5	Harrell 2022	Irrelevant	Excluded by TI/AB review
2791	Incidence of Tendon Ruptures in the US: Comparison of Differences Across Racial Groups...American Orthopaedic Society for Sports Medicine Annual Meeting, July 13- [illegible]	Berkay, Fehmi; Minhas, Arjun; Krishnamurthy, Anil; Lyons, Joseph	2023	Orthopaedic Journal of Sports Medicine	11	NA	40-42	10.1177/2325967123S00338	Berkay 2023	Irrelevant	Excluded by TI/AB review

S4 Table. All studies identified in the literature search

2792	Intranasal Fentanyl to Reduce Pain and Improve Oral Intake in the Management of Children With Painful Infectious Mouth Lesions.	Ruffin Jr, Thomas B.; Salinero, Efren; Papa, Linda; Cramm, Kelly; Florez, Camilo; Chen, J. Gene; Ramirez, Jose; Ruffin, Thomas B Jr	2022	Pediatric Emergency Care	38	8	363-366	10.1097/PEC.0000000000002779	RuffinJr 2022	Irrelevant	Excluded by TI/AB review
2793	Investigating racial disparities within an emergency department rapid-triage system.	Boley, Sean; Sidebottom, Abbey; Vacquier, Marc; Watson, David; Olsen, Jeremy; Echols, Kelsey; Friedman, Sara	2022	American Journal of Emergency Medicine	60	NA	65-72	10.1016/j.ajem.2022.07.030	Boley 2022	Irrelevant	Excluded by TI/AB review
2794	Long-term Medication Adherence and Preventive vs Reactive Care [illegible]	Karpes Matusevich, Aliza R.; Xavier, Christy; Rasu, Rafia S.	2022	American Journal of Managed Care	28	10	e378-e387	10.37765/ajmc.2022.89255	KarpesMatusevich 2022	Irrelevant	Excluded by TI/AB review

S4 Table. All studies identified in the literature search

2795	Measuring Racial Inequities In The Quality Of Care Across Oncology Practices In The US.	Keating, Nancy L.; Landrum, Mary Beth; Samuel-Ryals, Cleo; Sinaiko, Anna D.; Wright, Alexi; Brooks, Gabriel A.; Bai, Barbara; Zaslavsky, Alan M.	2022	Health Affairs	41	4	598-606	10.1377/hlthaff.2021.01594	Keating 2022	Irrelevant	Excluded by TI/AB review
2797	Nationwide racial/ethnic disparities in US emergency department visits and hospitalizations for gout.	Yokose, Chio; McCormick, Natalie; Lu, Na; Joshi, Amit D; Jackson, Lesley; Kohler, Minna J; Yinh, Janeth; Zhang, Yuqing; Hsu, John; Dalbeth, Nicola; Saag, Kenneth G; Choi, Hyon K.	2023	Rheumatology	62	6	2247-2251	10.1093/rheumatology/keac590	Yokose 2023	Irrelevant	Excluded by TI/AB review

S4 Table. All studies identified in the literature search

2798	Nurse, Provider, and Emergency Department Technician: Perceptions and Experiences of Violence and Aggression in the Emergency Department.	Boles, Jean M.; Maccarone, Diane; Brown, Beverly; Archer, Alexandra; Trotter, Michael G.; Friedman, Nicholas M.G.; Chittams, Jesse; Mazzone, Leighann; Ballinghoff, James; Burchill, Christian N.; Cacchione	2023	Journal of Emergency Nursing	49	3	431-440	10.1016/j.jen.2022.07.008	Boles 2023	Irrelevant	Excluded by TI/AB review
2799	Operator-specific outcomes in endoscopic sleeve gastroplasty: a propensity-matched analysis of the US population using a multicenter database.	Ali, Hassam; Inayat, Faisal; Malik, Talia F.; Patel, Pratik; Nawaz, Gul; Taj, Sobaan; Rehman, Attiq Ur; Afzal, Arslan; Ishtiaq, Rizwan; Afzal, Muhammad Sohaib; Advani, Rashmi; Watson, Rabindra R.	2023	Baylor University Medical Center Proceedings	36	5	592-599	10.1080/08998280.2023.2228180	Ali 2023	Irrelevant	Excluded by TI/AB review

S4 Table. All studies identified in the literature search

2800	Patient experiences of a care transition intervention for Veterans to reduce emergency department visits.	Seidenfeld, Justine; Ramos, Katherine; Bruening, Rebecca A.; Sperber, Nina R.; Stechuchak, Karen M.; Hastings, Susan N.	2023	Academic Emergency Medicine	30	4	388-397	10.1111/acem.14661	Seidenfeld 2023	Irrelevant	Excluded by TI/AB review
2801	Patient Sociodemographics and Comorbidities and Birth Hospital Characteristics Associated With [illegible]	Zarrin, Haley; Vargas-Torres, Carmen; Janevic, Teresa; Stern, Toni; Lin, Michelle P.	2023	JAMA Network Open	6	3	e233927-e233927	10.1001/jamanetworkopen.2023.3927	Zarrin 2023	Irrelevant	Excluded by TI/AB review
2802	Patient-Level and County-Level Trends in Nonfatal Opioid-Involved Overdose Emergency [illegible]	Casillas, Shannon M.; Pickens, Cassandra M.; Stokes, Erin K.; Walters, Josh; Vivolo-Kantor, Alana	2022	MMWR: Morbidity & Mortality Weekly Report	71	34	1073-1080	10.15585/mmwr.mm7134a1	Casillas 2022	Irrelevant	Excluded by TI/AB review
2803	Pediatric emergency care in New York City during the COVID-19 pandemic shutdown and reopening periods.	Liang, Tian; Chamdawala, Haamid S.; Tay, Ee Tein; Chao, Jennifer; Waseem, Muhammad; Lee, Horton; Mortel, David; Agoritsas, [illegible]	2022	American Journal of Emergency Medicine	56	NA	137-144	10.1016/j.ajem.2022.03.049	Liang 2022	Irrelevant	Excluded by TI/AB review

S4 Table. All studies identified in the literature search

2804	Practicing cultural humility toward Black and Brown communities in the ED.	Wall, Aishia	2023	Nursing	53	4	41-44	10.1097/01.NURSE.0000920456.10204.1a	Wall 2023	Irrelevant	Excluded by TI/AB review
2805	Pragmatic Clinical Trial to Improve Patient Experience Among Adults During Transitions from Hospital to Home: the [illegible]	LaBedz, Stephanie L.; Prieto-Centurion, Valentin; Mutso, Amelia; Basu, Sanjib; Bracken, Nina E.; Calhoun, Elizabeth A.; DiDomenico, [illegible]	2022	JGIM: Journal of General Internal Medicine	37	16	4103-4111	10.1007/s11606-022-07461-0	LaBedz 2022	Irrelevant	Excluded by TI/AB review
2806	Predicting short-term outcomes after transcatheter aortic valve replacement for aortic stenosis.	Savitz, Samuel T.; Leong, Thomas; Sung, Sue Hee; Kitzman, Dalane W.; McNulty, Edward; Mishell, Jacob; Rassi, Andrew; Ambrosy, Andrew P.; Go, Alan S.	2023	American Heart Journal	256	NA	60-72	10.1016/j.ahj.2022.11.007	Savitz 2023	Irrelevant	Excluded by TI/AB review

S4 Table. All studies identified in the literature search

2807	Prehospital Evaluation of the FAST-ED as a Secondary Stroke Screen to Identify Large Vessel Occlusion Strokes.	Dowbiggin, Patricia L.; Infinger, Allison E.; Purick, Gabrielle T.; Swanson, Douglas R.; Asimos, Andrew; Rhoten, Jeremy B.; VonCannon, Shellie; Dometrovich, Melissa; Studnek, Jonathan R.	2022	Prehospital Emergency Care	26	3	333-338	10.1080/10903127.2021.1979701	Dowbiggin 2022	Irrelevant	Excluded by TI/AB review
2808	Prehospital time for patients with acute cardiac complaints: A rural health [illegible]	Ashburn, Nicklaus P.; Snavely, Anna C.; Angi, Ryan M.; Scheidler, James F.; Crowe, Remle P.; [illegible]	2022	American Journal of Emergency Medicine	52	NA	64-68	10.1016/j.ajem.2021.11.038	Ashburn 2022	Irrelevant	Excluded by TI/AB review
2809	Prevalence of Behavioral Flags in the Electronic Health Record Among Black and White Patients Visiting the Emergency Department.	Agarwal, Anish K.; Seeburger, Emily; O'Neill, Gerald; Nwakanma, Chidinma C.; Marsh, Lillian E.; Soltany, Kevin Alexander; South, Eugenia C.; Friedman, Ari B.	2023	JAMA Network Open	6	1	e2251734-e2251734	10.1001/jamanetworkopen.2022.51734	Agarwal 2023	Irrelevant	Excluded by TI/AB review

S4 Table. All studies identified in the literature search

2810	Prospective Evaluation of a Machine-Learning Prediction Model for Missed Radiology Appointments.	Rothenberg, Steven; Bame, Bill; Herskovitz, Ed	2022	Journal of Digital Imaging	35	6	1690-1693	10.1007/s10278-022-00670-3	Rothenberg 2022	Irrelevant	Excluded by TI/AB review
2811	Psychosocial risk factors of erectile dysfunction among heterosexual men living with HIV.	Akbas, Selin; Alc√©na-Stiner, Danielle C.; McMahon, James M.	2023	AIDS Care	35	2	253-260	10.1080/09540121.2022.2039357	Akbas 2023	Irrelevant	Excluded by TI/AB review
2812	Quality Assurance Standards for Outreach Cleft Lip and Cleft Palate Repair Programs in Low-Resource Settings.	Chahine, Elsa M.; Ramly, Elie P.; Marston, Alexander P.; Teng, Sarena N.; Nader, Marie; Kassam, Serena N.; Kantar, Rami S.; Hamdan, Usama S.	2023	Cleft Palate Craniofacial Journal	60	6	773-779	10.1177/10556656221074883	Chahine 2023	Irrelevant	Excluded by TI/AB review

S4 Table. All studies identified in the literature search

2813	Quality emergency care (QEC) in resource limited settings: A concept analysis.	Mamalelala, Tebogo T	2022	International Emergency Nursing	64	NA	N.PAG-N.PAG	10.1016/j.ienj.2022.101198	Mamalelala 2022	Irrelevant	Excluded by TI/AB review
2814	Race and Ethnicity and Prehospital Use of Opioid or Ketamine Analgesia in Acute Traumatic Injury.	Brunson, Dalton C.; Miller, Kate A.; Matheson, Loretta W.; Carrillo, Eli	2023	JAMA Network Open	6	10	e2338070-e2338070	10.1001/jamanetworkopen.2023.38070	Brunson 2023	Irrelevant	Excluded by TI/AB review

S4 Table. All studies identified in the literature search

2815	Race, Healthcare, and Health Disparities: A Critical Review and Recommendations for Advancing Health Equity.	Macias-Konstantopoulos, Wendy L.; Collins, Kimberly A.; Diaz, Rosemarie; Duber, Herbert C.; Edwards, Courtney D.; Hsu, Antony P.; Ranney, Megan L.; Riviello, Ralph J.; Wettstein, Zachary S.; Sachs, Carolyn J.	2023	Western Journal of Emergency Medicine: Integrating Emergency Care with Population Health	24	5	906-918	10.5811/westjem.58408	Macias-Konstantopoulos 2023	Irrelevant	Excluded by TI/AB review

S4 Table. All studies identified in the literature search

2817	Racial and Ethnic Differences in Insurer Classification of Nonemergent Pediatric Emergency Department Visits.	Pomerantz, Alexander; De Souza, Heidi G.; Hall, Matthew; Neuman, Mark I.; Goyal, Monika K.; Samuels-Kalow, Margaret E.; Aronson, Paul L.; Alpern, Elizabeth R.; Simon, Harold K.; Hoffmann, Jennifer A.; Wells, Jordee M.;	2023	JAMA Network Open	6	5	e2311752-e2311752	10.1001/jamanetworkopen.2023.11752	Pomerantz 2023	Irrelevant	Excluded by TI/AB review
2818	Racial and ethnic differences in low‚Äêvalue pediatric emergency care.	Li, Joyce; Ramgopal, Sriram; Marin, Jennifer R.	2022	Academic Emergency Medicine	29	6	698-709	10.1111/acem.14468	Li 2022	Irrelevant	Excluded by TI/AB review
2819	Racial and Ethnic Disparities in Hospitalization and Clinical Outcomes Among Patients with COVID-19.	Serrano, Felipe; Blutinger, Erik J.; Vargas-Torres, Carmen; Bilal, Saadiyah; Counts, Christopher; Straight, Matthew; Lin, [illegible]	2022	Western Journal of Emergency Medicine: Integrating Emergency Care with [illegible]	23	5	601-612	10.5811/westjem.2022.3.53065	Serrano 2022	Irrelevant	Excluded by TI/AB review

S4 Table. All studies identified in the literature search

2820	Racial And Ethnic Disparities In Preventable Hospitalizations And ED Visits Five Years After [illegible]	Asako S. Moriya; Chakravarty, Sujoy	2023	Health Affairs	42	1	26-34	10.1377/hlthaff.2022.00460	Asako 2023	Irrelevant	Excluded by TI/AB review
2821	Racial Disparities in Care Interactions and Clinical Outcomes in Black Versus White Nursing Home Residents With Dementia.	Resnick, Barbara; Van Haitsma, Kimberly; Kolanowski, Ann; Galik, Elizabeth; Boltz, Marie; Ellis, Jeanette; Behrens, Liza; Eshraghi, Karen	2022	Journal of Nursing Care Quality	37	3	282-288	10.1097/NCQ.0000000000000606	Resnick 2022	Irrelevant	Excluded by TI/AB review

S4 Table. All studies identified in the literature search

2823	Racial equity in linkage to inpatient opioid use disorder treatment in patients that received emergency care.	Faiz, Jessica; Bernstein, Ed; Dugas, Julianne N.; Schechter-Perkins, Elissa M.; Nentwich, Lauren; Nelson, Kerrie P.; Cleveland Manchanda, Emily C.; Young, Ludy; Pare, Joseph R.	2022	American Journal of Emergency Medicine	54	NA	221-227	10.1016/j.ajem.2022.01.037	Faiz 2022	Irrelevant	Excluded by TI/AB review
2824	Racial implicit bias among obstetricians and midwives, and information on Down syndrome screening...14th European Public [illegible]	Azria, E.; Rousseau, A.; Anselem, O.; Bonnet, M. P.; Deneux, C.; Sauvegrain, P.; Richetin, J.	2021	European Journal of Public Health	31	NA	iii147-iii147	NA	Azria 2021	Irrelevant	Excluded by TI/AB review
2825	Racial inequities in emergency department wait times for pregnancy-related concerns.	Deichen Hansen, Megan E.; Goldfarb, Samantha S.; Mercouffer, Ariadna; Dark, Tyra; Lateef, Hanna; Harman, Jeffrey S.	2022	Women's Health (17455057)	18	NA	7-Jan	10.1177/17455057221129388	DeichenHansen 2022	Irrelevant	Excluded by TI/AB review

S4 Table. All studies identified in the literature search

2827	Reduced Postdischarge Incidents After Implementation of a Hospital-to-Home Transition Intervention for Children With Medical Complexity.	Huth, Kathleen; Hotz, Arda; Emara, Norah; Robertson, Bryanna; Leaversuch, Marissa; Mercer, Alexandra N.; Khan, Alisa; Campos, Maria-Lucia; Liss, Isabella; Hahn, Phillip D.; Graham, Dionne A.; Rossi, Laura; Thomas	2023	Journal of Patient Safety	19	7	493-500	10.1097/pts.0000000000001155	Huth 2023	Irrelevant	Excluded by TI/AB review
2828	Reduced rate of postpartum readmissions among homeless compared with non-homeless women in New York: a population-based study using serial, cross-sectional data	Bizmark, Rie Sakai; Kumamaru, Hiraku; Estevez, Dennys; Neman, Sophia; Bedel, Lauren E. M.; Mena, Laurie A.; Marr, Emily H.; Ross, Michael G.	2022	BMJ Quality & Safety	31	4	267-277	10.1136/bmjqs-2020-012898	Bizmark 2022	Irrelevant	Excluded by TI/AB review
2829	Sociodemographic Disparities in Queue Jumping for Emergency Department Care.	Sangal, Rohit B.; Su, Huifeng; Khidir, Hazar; Parwani, Vivek; Liebhardt, Beth; Pinker, Edieal J.; Meng, Lesley; Venkatesh, Arjun K.; Ulrich, Andrew	2023	JAMA Network Open	6	7	e2326338-e2326338	10.1001/jamanetworkopen.2023.26338	Sangal 2023	Irrelevant	Excluded by TI/AB review

S4 Table. All studies identified in the literature search

2830	Structured Outpatient Diabetes Education and Telehealth Follow-up to Improve HbA 1c.	Harris-Hardaway, Marina B.; Novotny, Nancy L.; Sawyer, Jennifer Dana; Schmitt, Erin	2023	Journal of Nursing Care Quality	38	3	220-225	10.1097/NCQ.0000000000000686	Harris-Hardaway 2023	Irrelevant	Excluded by TI/AB review
2831	Study: Follow‚Äêup care for children lacking after emergency care visit.	Enos, Gary	2023	Mental Health Weekly	33	9	7-Jan	10.1002/mhw.33554	Enos 2023	Irrelevant	Excluded by TI/AB review
2832	Testing dental quality measures: Emergency department visits for nontraumatic dental conditions and subsequent follow-up dental visits.	Obadan-Udoh, Enihomo; Herndon, Jill Boylston; Kohli, Richie; McKernan, Susan; Jura, Matthew; Momany, Elizabeth; Arora, Garima; Sehgal, Harjit Singh; Yansane, Alfa-Ibrahim; Mertz, Elizabeth; Ojha, Diptee; Amundson	2023	Journal of the American Dental Association (JADA)	154	6	507-518	10.1016/j.adaj.2023.03.008	Obadan-Udoh 2023	Irrelevant	Excluded by TI/AB review

S4 Table. All studies identified in the literature search

2833	The association of race with CPR quality following out-of-hospital cardiac arrest.	Schmicker, Robert H.; Blewer, Audrey; Lupton, Joshua R.; Aufderheide, Tom P.; Wang, Henry E.; Idris, Ahamed H.; Aramendi, Elisabete; Hagahmed, Mohamed B.; Traynor, Owen T.; Colella, M. Riccardo; Daya, Mohamud R.	2022	Resuscitatio n	170	NA	194-200	10.1016/j.re suscitation.2 021.11.038	Schmicke r 2022	Irrelevant	Excluded by TI/AB review
2834	The power of the lived experience: improving awareness of cardiovascular care for minority ethnic service users.	Jones, Lorna	2023	British Journal of Cardiac Nursing	18	1	3-Jan	10.12968/bj ca.2023.000 1	Jones 2023	Irrelevant	Excluded by TI/AB review

S4 Table. All studies identified in the literature search

2835	User and community coping responses to service delivery gaps in emergency obstetric care provision in a rural community in [illegible]	Ayawine, Alice; Atinga, Roger A.	2022	Health & Social Care in the Community	30	6	e4513-e4521	10.1111/hsc.13855	Ayawine 2022	Irrelevant	Excluded by TI/AB review

S4 Table. All studies identified in the literature search

2836	Vaccine Acceptance During a Novel Student-led Emergency Department COVID-19 Vaccination Program.	Eastin, Carly; Moore, Brendan; Moulton, Aaron; Lefler, Luke; Haydar, Fuad; Sweere, Morgan; Jones, Gavin; Sparks, Crystal; Porter, Austin; Allison, M. Kathryn; Eastin, Travis	2023	Western Journal of Emergency Medicine: Integrating Emergency Care with Population Health	24	3	436-446	10.5811/westjem.58728	Eastin 2023	Irrelevant	Excluded by TI/AB review

S4 Table. All studies identified in the literature search

2837	"What Made My Eating Disorder Worse?" The Impact of the COVID-19 Pandemic from the Perspective of Adolescents with Anorexia Nervosa.	Gilsbach, Susanne; Herpertz-Dahlmann, Beate	2023	Nutrients	15	5	1242	10.3390/nu15051242	Gilsbach 2023	Irrelevant	Excluded by TI/AB review
2838	Technology-enabled virtual ward for COVID management of the elderly and immunocompromised in Singapore: a [illegible]	Ko, S.Q.; Kumar, S.K.; Jacob, J.; Hooi, B.M.Y.; Soo, M.; Nashi, N.; Cruz, M.T.D.; Wah, Y.A.; Xin, W.Z.; Smitasin, N.; Lum, L.; [illegible]	2023	BMC Infectious Diseases	23	1	NA	10.1186/s12879-023-08040-2	Ko 2023	Irrelevant	Excluded by TI/AB review

S4 Table. All studies identified in the literature search

2840	Health-related quality of life disparities among Hispanic/Latinx patients with nephrolithiasis	Flores, A.R.; Abedi, G.; Girgiss, C.B.; Berger, J.H.; Penniston, K.L.; Li, S.; Friedlander, D.F.; Bechis, S.K.; Sur, R.L.	2023	Urolithiasis	51	1	NA	10.1007/s00240-023-01414-w	Flores 2023	Irrelevant	Excluded by TI/AB review
2841	Ensuring Equitable Care in Diabetes Management Among Patients of Health Resources & Services Administration,Äì Funded Health Centers in the United States	Pourat, N.; Chen, X.; Lu, C.; Zhou, W.; Hair, B.; Bolton, J.; Sripipatana, A.	2023	Diabetes Spectrum	36	1	69-77	10.2337/ds22-0016	Pourat 2023	Irrelevant	Excluded by TI/AB review
2842	Is there equity of patient health outcomes across models of general practice in Aotearoa New Zealand? A national cross-sectional study	Sheridan, N.; Love, T.; Kenealy, T.; Aguirre-Duarte, N.; Arroll, B.; Atmore, C.; Carryer, J.; Crampton, P.; Dowell, A.; Fishman, T.; Gauld, R.; Harwood, M.; Hoare, [illegible]	2023	International Journal for Equity in Health	22	1	NA	10.1186/s12939-023-01893-8	Sheridan 2023	Irrelevant	Excluded by TI/AB review

S4 Table. All studies identified in the literature search

2843	An analysis of racial inequities in emergency department triage among patients with stroke-like symptoms in the United States	Neves, G.; DeToledo, J.; Morris, J.; Xu, K.T.	2023	BMC Emergency Medicine	23	1	NA	10.1186/s12873-023-00865-z	Neves 2023	Irrelevant	Excluded by TI/AB review

S4 Table. All studies identified in the literature search

2844	Humanistic and Economic Burden of Patients with Cardiorenal Metabolic Conditions: A Systematic Review	Ferdinand, K.C.; Norris, K.C.; Rodbard, H.W.; Trujillo, J.M.	2023	Diabetes Therapy	14	12	1979-1996	10.1007/s13300-023-01464-8	Ferdinand 2023	Irrelevant	Excluded by TI/AB review

S4 Table. All studies identified in the literature search

2845	Association of substance use with outcomes in mildly ill COVID-19 outpatients	Pobee, R.; Cable, T.; Chan, D.; Herrick, J.; Durkalski-Mauldin, V.; Korley, F.; Callaway, C.; Del Rios, M.	2023	American Journal of Emergency Medicine	74	(Pobee R., rpobee@uic.edu; Del Rios M., marina-delrios@uiowa.edu) UI Health/University of Illinois Chicago College of Medic[illegible]	27-31	10.1016/j.ajem.2023.09.017	Pobee 2023	Irrelevant	Excluded by TI/AB review

S4 Table. All studies identified in the literature search

2846	The association between specific narrative elements and patient perspectives on acute pain treatment	Engel-Rebitzer, E.; Dolan, A.; Shofer, F.S.; Schapira, M.M.; Hess, E.P.; Rhodes, K.V.; Bellamkonda, V.R.; Msw, E.G.; Bell, J.; Schwarz, L.; Schiller, [illegible]	2023	American Journal of Emergency Medicine	74	(Engel-Rebitzer E., eengelrebitzer@partners.org; [illegible]	84-89	10.1016/j.ajem.2023.09.016	Engel-Rebitzer 2023	Irrelevant	Excluded by TI/AB review

S4 Table. All studies identified in the literature search

2847	Racial Disparities in Emergency Department Physical Restraint Use: A Systematic Review and Meta-Analysis	Eswaran, V.; Molina, M.F.; Hwong, A.R.; Dillon, D.G.; Alvarez, L.; Allen, I.E.; Wang, R.C.	2023	JAMA Internal Medicine	183	11	1229-1237	10.1001/jamainternmed.2023.4832	Eswaran 2023	Irrelevant	Excluded by TI/AB review

S4 Table. All studies identified in the literature search

2848	Cognitive Function and Postoperative Outcomes in Patients with Head and Neck Cancer	Larrabee, K.; Meeks, N.; Williams, A.M.; Springer, K.; Siddiqui, F.; Chang, S.S.; Ghanem, T.; Wu, V.F.; Momin, S.; Tam, S.	2023	Laryngoscope	133	11	2999-3005	10.1002/lary.30677	Larrabee 2023	Irrelevant	Excluded by TI/AB review
2849	Diagnostic error among vulnerable populations presenting to the emergency department with cardiovascular and cerebrovascular or [illegible]	Herasevich, S.; Soleimani, J.; Huang, C.; Pinevich, Y.; Dong, Y.; Pickering, B.W.; Murad, M.H.; Barwise, A.K.	2023	BMJ Quality and Safety	32	11	676-688	10.1136/bmjqs-2022-015038	Herasevich 2023	Irrelevant	Excluded by TI/AB review

S4 Table. All studies identified in the literature search

2850	Association of program-specific variation in bariatric surgery volume for Medicaid patients and access to care: a tale of inequality?	Somerset, A.E.; Wood, M.H.; Bonham, A.J.; Carlin, A.M.; Finks, J.; Ghaferi, A.A.; Varban, O.A.	2023	Surgical Endoscopy	37	11	8570-8576	10.1007/s00464-023-10411-4	Somerset 2023	Irrelevant	Excluded by TI/AB review
2851	Preference for and impact of telehealth vs in-person asthma visits among Black and Latinx adults	Ugalde, I.C.; Ratigan, A.; Merriman, C.; Cui, J.; Ericson, B.; Busse, P.; Carroll, J.K.; Casale, T.; Celed√≥n, J.C.; Coyne-Beasley, T.; Fagan, M.; [illegible]	2023	Annals of Allergy, Asthma and Immunology	131	5	614-627.e2	10.1016/j.anai.2023.07.012	Ugalde 2023	Irrelevant	Excluded by TI/AB review

S4 Table. All studies identified in the literature search

2852	Implementing a peer-supported, integrated strategy for substance use disorder care in an outpatient infectious disease clinic is associated with improved patient outcomes	Falade-Nwulia, O.; Agee, T.; Kelly, S.M.; Park, J.N.; Schwartz, S.; Hsu, J.; Schweizer, N.; Jones, J.; Keruly, J.; Shah, N.; Lesko, C.R.; Lucas, G.M.; Sulkowski, M.	2023	International Journal of Drug Policy	121	(Falade-Nwulia O., ofalade1@jhmi.edu; Agee T.; [illegible]	NA	10.1016/j.drugpo.2023.104191	Falade-Nwulia 2023	Irrelevant	Excluded by TI/AB review
2853	Presentation and Outcomes of Elective and Nonelective Complex Endovascular Repair for Thoracoabdominal and Juxtarenal Aortic Aneurysms	Krebs, J.R.; Fazzone, B.; Anderson, E.M.; Ueland, W.; Spratt, J.R.; Back, M.R.; Shahid, Z.; Huber, T.S.; Upchurch, G.R.; Cooper, M.A.	2023	Annals of Vascular Surgery	97	(Krebs J.R.; Fazzone B.; Anderson E.M.; Ueland W.; Back [illegible]	248-256	10.1016/j.avsg.2023.09.001	Krebs 2023	Irrelevant	Excluded by TI/AB review

S4 Table. All studies identified in the literature search

2854	P1.25-03 Comparing Timeliness of Treatment for Surgery vs Radiation for Early-Stage Lung Cancer	Zhu, J.; Kantor, S.; Zhang, J.; Yip, R.; Henschke, C.I.; Yankelevitz, D.F.	2023	Journal of Thoracic Oncology	18	11	S268	10.1016/j.jtho.2023.09.457	Zhu 2023	Irrelevant	Excluded by TI/AB review
2855	Socioeconomic, Racial, and Insurance Disparities in Clinical Outcomes After Surgery Among Patients With Idiopathic Scoliosis	Thornley, P.; Garner, S.; Rogers, K.J.; Yorgova, P.; Gabos, P.G.; Shah, S.A.	2023	Journal of pediatric orthopedics	NA	(Thornley P.) Department of Orthopaedics, Nemours Children's Health, Wilmington,	NA	10.1097/BPO.0000000000002551	Thornley 2023	Irrelevant	Excluded by TI/AB review

S4 Table. All studies identified in the literature search

2856	Disparities in Emergency Department Waiting Times for Acute Gastrointestinal Bleeding: Results from the National Hospital Ambulatory Medical Care Survey, 2009-2018	Aghaie Meybodi, M.; Shaikh, A.; Hashemipour, R.; Ahlawat, S.	2023	Journal of Clinical Gastroenterology	57	9	901-907	10.1097/MCG.0000000000000001805	AghaieMeybodi 2023	Irrelevant	Excluded by TI/AB review

S4 Table. All studies identified in the literature search

2857	Screening for the high-need population using single institution versus state-wide admissions discharge transfer feed	Balucan, F.S.; French, B.; Shi, Y.; Kripalani, S.; Vasilevskis, E.E.	2023	BMC health services research	23	1	1111	10.1186/s12913-023-10017-5	Balucan 2023	Irrelevant	Excluded by TI/AB review

S4 Table. All studies identified in the literature search

2858	Initial steps in addressing the challenges of elder mistreatment evaluation: Protocol for evaluating the Vulnerable Elder Protection Team	Baek, D.; Elman, A.; Gottesman, E.; Shaw, A.; Makaroun, L.K.; Stern, M.E.; Mulcare, M.R.; Sullivan, M.; Pino, C.; McAuley, J.; Lofaso, V.M.; Chang, E.-S.; Hancock, D.; [illegible]	2023	BMJ Open	13	10	NA	10.1136/bmjopen-2023-071694	Baek 2023	Irrelevant	Excluded by TI/AB review

S4 Table. All studies identified in the literature search

2859	Generative Artificial Intelligence for Chest Radiograph Interpretation in the Emergency Department	Huang, J.; Neill, L.; Wittbrodt, M.; Melnick, D.; Klug, M.; Thompson, M.; Bailitz, J.; Loftus, T.; Malik, S.; Phull, A.; Weston, V.; Heller, J.A.; Etemadi, M.	2023	JAMA Network Open	6	10	E2336100	10.1001/jamanetworkopen.2023.36100	Huang 2023	Irrelevant	Excluded by TI/AB review

S4 Table. All studies identified in the literature search

2860	Race differences in cardiac testing rates for patients with chest pain in a multisite cohort	Popp, L.M.; Ashburn, N.P.; Snavely, A.C.; Allen, B.R.; Christenson, R.H.; Madsen, T.; Mumma, B.E.; Nowak, R.; Stopyra, J.P.; Wilkerson, R.G.; Mahler, S.A.	2023	Academic Emergency Medicine	30	10	1020-1028	10.1111/acem.14762	Popp 2023	Irrelevant	Excluded by TI/AB review
2861	Virtual triage from freestanding emergency departments: a propensity score-weighted analysis of short-term outcomes in emergency general surgery	Lorenz, W.; Yang, H.; Paton, L.; Barbat, S.; Matthews, B.; Reinke, C.E.; Schiffern, L.; Baimas-George, M.	2023	Surgical Endoscopy	37	10	7901-7907	10.1007/s00464-023-10241-4	Lorenz 2023	Irrelevant	Excluded by TI/AB review

S4 Table. All studies identified in the literature search

2862	Pain management in adult patients with sickle cell disease in the emergency department: how does current practice compare with existing [illegible]	Gulilat, M.; Tunji-Ajayi, L.; Thompson, S.; Poku, M.-P.; Appiah-Boateng, R.; Navarro, N.; Sheikh, H.; Hulme, J.; Bryan, J.; Azubuike, K.; Guerrier, U.; Armstrong, L.; [illegible]	2023	Canadian Journal of Emergency Medicine	25	10	836-844	10.1007/s43678-023-00579-y	Gulilat 2023	Irrelevant	Excluded by TI/AB review
2863	An Integrated Patient-Centred Medical Home (PCMH) Care Model Reduces Prospective [illegible]	Sum, G.; Sim, S.Y.H.; Chay, J.; Ho, S.H.; Ginting, M.L.; Lim, Z.Z.B.; Yoong, J.; Wong, C.H.	2023	International Journal of Environmental Research and Public Health	20	19	NA	10.3390/ijerph20196848	Sum 2023	Irrelevant	Excluded by TI/AB review
2864	Sustained virologic response from hepatitis C from an emergency department screening & linkage program: A retrospective review	Walter, L.A.; Wilson, L.; Farmer, M.; Roberson, T.; Hand, D.T.; Franco, R.	2023	American Journal of Emergency Medicine	72	(Walter L.A., lwalter@uabmc.edu; Wilson L.) Department of [illegible]	178-182	10.1016/j.ajem.2023.07.050	Walter 2023	Irrelevant	Excluded by TI/AB review

S4 Table. All studies identified in the literature search

2865	A successful campaign to increase use of the sepsis order set in the emergency department	Berlyand, Y.; Black, L.; Lee, A.H.-Y.; Aaronson, E.L.; Copenhaver, M.S.; Filbin, M.R.; Mort, E.A.; Dutta, S.; Rhee, C.; Hibbert, K.A.; Turno, D.-J.C.; Durocher, K.E.; Aristizabal, M.E.; Sonis, J.D.	2023	American Journal of Emergency Medicine	72	(Berly and Y.; Black L.; Lee A.H.-Y.; Aaron son E.L.; Filbin M.R.; [illegible]	216-218	10.1016/j.ajem.2023.07.052	Berlyand 2023	Irrelevant	Excluded by TI/AB review
2866	Natural Language Processing Identification of Symptoms in Emergency Department Visits in Patients Receiving Radiation [illegible]	Qian, A.S.; Friesner, I.; Chen, J.J.; Boreta, L.; Braunstein, S.E.; Hong, J.C.	2023	International Journal of Radiation Oncology Biology Physics	117	2	S144	10.1016/j.ijrobp.2023.06.558	Qian 2023	Irrelevant	Excluded by TI/AB review

S4 Table. All studies identified in the literature search

2867	Prevalence and correlates of knife-related victimization: Insights from the 2019 Nationwide Emergency Department Sample	Vaughn, M.G.; Dell, N.A.; Long, C.; Qian, A.; DeLisi, M.	2023	Preventive Medicine	175	(Vaughn M.G.; Long C.) School of Social Work, Saint Louis University	NA	10.1016/j.ypmed.2023.107680	Vaughn 2023	Irrelevant	Excluded by TI/AB review
2868	Requirement for Discharge in the Care of a Responsible Adult in Procedural Sedation in the Emergency Department: [illegible]	Ayandeh, A.; Farrell, N.; Sheng, A.Y.	2023	Journal of Emergency Medicine	65	4	e272-e279	10.1016/j.jemermed.2023.05.010	Ayandeh 2023	Irrelevant	Excluded by TI/AB review
2869	327 Does Guideline Implementation Affect Sex and Race/Ethnicity Differences in [illegible]	Murray, E.; Haukoos, J.; Trent, S.	2023	Annals of Emergency Medicine	82	4	S142-S143	10.1016/j.annemergmed.2023.08.352	Murray 2023	Irrelevant	Excluded by TI/AB review

S4 Table. All studies identified in the literature search

2870	284 Nationwide Capabilities Assessment of Emergency Department Care of Patients With Opioid Use Disorder: 2022 to [illegible]	Hawk, K.; Weiner, S.; Sharma, P.; Rothenberg, C.; Sharma, D.; Goyal, P.; Rudowski, K.; Sambell, M.; Venkatesh, A.	2023	Annals of Emergency Medicine	82	4	S125	10.1016/j.annemergmed.2023.08.308	Hawk 2023	Irrelevant	Excluded by TI/AB review
2871	285 Trauma Evaluation and Outcomes of Incarcerated and Non-Incarcerated [illegible]	Suh, M.; Manisundaram, N.; Robinson, D.; Eswaran, V.	2023	Annals of Emergency Medicine	82	4	S125-S126	10.1016/j.annemergmed.2023.08.309	Suh 2023	Irrelevant	Excluded by TI/AB review
2872	150EMF Pilot Study of an Innovative Model to Provide Multi-modal Cognitive Behavioral Theory-Informed Physical Therapy (CBT-[illegible]	Eucker, S.; O'Regan, A.; Winger, J.; Shelby, R.; Brower, M.; Finkelstein, S.; Chan, E.; Gordee, A.; Kuchibhatla, M.; Simon, C.	2023	Annals of Emergency Medicine	82	4	S65	10.1016/j.annemergmed.2023.08.171	Eucker 2023	Irrelevant	Excluded by TI/AB review
2873	37 The Patient Voice Project: A Qualitative Analysis of Patient Experiences With New York City Emergency Departments [illegible]	Sanky, C.; Pelin, C.; Park, S.; Mathur, D.; Bilbro, N.; Li, T.; Becker, L.; Carr, B.; Rhodes, K.	2023	Annals of Emergency Medicine	82	4	S15-S16	10.1016/j.annemergmed.2023.08.056	Sanky 2023	Irrelevant	Excluded by TI/AB review

S4 Table. All studies identified in the literature search

2874	214 Effect of an Emergency Department Waiting Room Outreach Intervention on [illegible]	Jasperse, N.; Fleischman, R.; Yadav, K.	2023	Annals of Emergency Medicine	82	4	S97	10.1016/j.annemergmed.2023.08.237	Jasperse 2023	Irrelevant	Excluded by TI/AB review
2875	274 Patient Utilization and Feedback After a Novel Pain Coach Educator and Integrative Pain Management Toolkit Session in [illegible]	Hendry, P.; Sheikh, S.; Montague, M.; Lott, M.; Velasquez, E.; Menze, N.; Curtis, M.; Perl, K.; Bartow, V.; Crisp, A.	2023	Annals of Emergency Medicine	82	4	S121-S122	10.1016/j.annemergmed.2023.08.298	Hendry 2023	Irrelevant	Excluded by TI/AB review
2876	90 Addressing Substance Use Disorder to Decrease Hospital Readmissions and Improve Behavioral Health Patient Outcomes	Bernard, J.; Carrillo, V.; Cascante, J.; Ali, N.; Rubenstein, B.; Luna, C.; Nyirenda, T.; Ogedegbe, C.	2023	Annals of Emergency Medicine	82	4	S37-S38	10.1016/j.annemergmed.2023.08.110	Bernard 2023	Irrelevant	Excluded by TI/AB review

S4 Table. All studies identified in the literature search

2877	188 A Qualitative Investigation of Black Patient Perspectives on Racism and Emergency Care	Agarwal, A.; Gonzales, R.; Sagan, C.; Nijim, S.; Merchant, R.; Asch, D.; South, E.	2023	Annals of Emergency Medicine	82	4	S86	10.1016/j.annemergmed.2023.08.208	Agarwal 2023	Irrelevant	Excluded by TI/AB review
2878	172 Who Fills Out Emergency Department Patient Satisfaction Surveys?	Zitek, T.; Weber, L.; Roitman, A.; Corbea, C.; Sherman, D.; Nunez, T.; Puron, L.; Shalaby, M.; Kresch, F.; Farcy, D.	2023	Annals of Emergency Medicine	82	4	S78	10.1016/j.annemergmed.2023.08.194	Zitek 2023	Irrelevant	Excluded by TI/AB review
2879	130 Older Adult Patient Engagement in Advanced Care Planning	Albert, E.; Crispo, M.; Strout, T.; Bunting, S.	2023	Annals of Emergency Medicine	82	4	S56	10.1016/j.annemergmed.2023.08.151	Albert 2023	Irrelevant	Excluded by TI/AB review

S4 Table. All studies identified in the literature search

2880	158 The Emergency Department as An Entry Point for Patients With Mpox: Opportunities for Improvement	Dashler, G.; Rudolph, D.; Brady, M.; Qiao, L.; Saheed, M.; Rothman, R.; Hsieh, Y.-H.; Hansoti, B.	2023	Annals of Emergency Medicine	82	4	S69	10.1016/j.annemergmed.2023.08.180	Dashler 2023	Irrelevant	Excluded by TI/AB review
2881	324 Comparison of Outcomes After Treatment of Asymptomatic Hypertension in the Emergency Department in Admitted Patients	Galla, S.; Xing, Y.; Zhao, L.; Todd, B.	2023	Annals of Emergency Medicine	82	4	S141	10.1016/j.annemergmed.2023.08.349	Galla 2023	Irrelevant	Excluded by TI/AB review

S4 Table. All studies identified in the literature search

2882	278 Pain Control Guidance Reduces Opioid Disparities But Not Prescriber Flexibility	Lee, C.; Rusoja, E.; Ward, K.; Auerbach, A.; Wick, E.; Bongiovanni, T.	2023	Annals of Emergency Medicine	82	4	S123	10.1016/j.annemergmed.2023.08.302	Lee 2023	Irrelevant	Excluded by TI/AB review

S4 Table. All studies identified in the literature search

2883	245 Patterns of Chemical Restraint Use for Emergency Department Patients With Acute Mental Health Distress	Lippert, S.; Xu, R.; Tucker, L.-Y.; Rabbani, J.; Rauchwerger, A.; Kene, M.	2023	Annals of Emergency Medicine	82	4	S111	10.1016/j.annemergmed.2023.08.267	Lippert 2023	Irrelevant	Excluded by TI/AB review
2884	305 The Association Between Frailty and Hospital Stays Among Geriatric Trauma Patients: A Retrospective Cohort Analysis	Adeyemi, O.; Grudzen, C.; DiMaggio, C.; Cuthel, A.; Arcila-Mesa, M.; Wittman, I.; Konda, S.; Tyrie, L.; Poracky, H.; Meyman, P.; Chodosh, J.	2023	Annals of Emergency Medicine	82	4	S133-S134	10.1016/j.annemergmed.2023.08.330	Adeyemi 2023	Irrelevant	Excluded by TI/AB review

S4 Table. All studies identified in the literature search

2885	190 Health Disparities in Emergency Department Administration of Buprenorphine for Treatment of Opioid Use Disorder	Koerber, S.; Huynh, D.; Farrington, S.; Springer, K.; Patel, M.; Manteuffel, J.	2023	Annals of Emergency Medicine	82	4	S87	10.1016/j.annemergmed.2023.08.210	Koerber 2023	Irrelevant	Excluded by TI/AB review
2886	35 Improving Adherence to Best Practices and Clinical Outcomes in Difficult Intravenous Access Patients	Zimmerman, T.; Mielke, N.; Bahl, A.	2023	Annals of Emergency Medicine	82	4	S15	10.1016/j.annemergmed.2023.08.054	Zimmerman 2023	Irrelevant	Excluded by TI/AB review

S4 Table. All studies identified in the literature search

2887	A CASE OF THORACIC ENDOMETRIOSIS PRESENTING WITH TENSION CATAMENIAL HEMOTHORAX	Nam, Y.; Miodownik, H.O.P.E.; Ramanathan, R.; Viswanathan, A.; Dweck, E.Z.R.A.	2023	Chest	164	4	A3720-A3721	10.1016/j.chest.2023.07.2420	Nam 2023	Irrelevant	Excluded by TI/AB review
2888	DIFFUSE ALVEOLAR HEMORRHAGE IN THE SETTING OF GRANULOMATOSIS WITH POLYANGIITIS: A CASE OF COMPLEXITY IN PRESENTATION AND MANAGEMENT	Clar, D.; O'Rear, C.O.D.Y.; Sarwar, H.M.	2023	Chest	164	4	A5584-A5585	10.1016/j.chest.2023.07.3610	Clar 2023	Irrelevant	Excluded by TI/AB review

S4 Table. All studies identified in the literature search

2889	ADVANCING RESPIRATORY HEALTH EQUITY THROUGH COMMUNITY-BASED EFFORTS FOR CHRONIC OBSTRUCTIVE PULMONARY DISEASE	Odhiambo, L.; Wright, S.; Joseph, K.; Moore, A.; Norman, J.; Crafton, B.; Jackson, L.; Islam, K.M.; Healy, W.J.; Taskar, V.; Forseen, C.J.	2023	Chest	164	4	A5028-A5029	10.1016/j.chest.2023.07.3256	Odhiambo 2023	Irrelevant	Excluded by TI/AB review
2890	PATIENTS WHO CHOOSE RPM ARE HIGHER RISK: SELF-SELECTION BIAS IN REMOTE MONITORING INTERVENTIONS FOR CHRONIC	Teresi, R.K.; Hendricks, A.; Murray, R.K.; Polsky, M.B.; Moraveji, N.	2023	Chest	164	4	A3884	10.1016/j.chest.2023.07.2532	Teresi 2023	Irrelevant	Excluded by TI/AB review

S4 Table. All studies identified in the literature search

2891	IMPROVING DIAGNOSTIC SAFETY AND SAVING LIVES THROUGH THE PANDEMIC BY SEPSIS REMOTE MONITORING AND A NURSE, CLINICAL CARE TEAM, AND TECHNICAL EXPERT TRIAD MODEL OF CARE	Wolfe, H.; Cichetti, J.; Edleblute, E.; Kashyap, R.; Proud, B.; Seim, M.	2023	Chest	164	4	A3813	10.1016/j.chest.2023.07.2482	Wolfe 2023	Irrelevant	Excluded by TI/AB review

S4 Table. All studies identified in the literature search

2892	ROTHIA MUCILAGINOSA PNEUMONIA IN AN OTHERWISE IMMUNOCOMPETENT ADULT WITH COVID-19	Khan, M.; Abdul Raffay, E.; Naeem, I.; Khan, S.; Basyal, B.	2023	Chest	164	4	A1471-A1472	10.1016/j.chest.2023.07.1016	Khan 2023	Irrelevant	Excluded by TI/AB review

S4 Table. All studies identified in the literature search

2893	THE ASSOCIATION OF RENIN-ANGIOTENSIN-ALDOSTERONE INHIBITORS AND ANTIPLATELET AGENTS WITH THE INFECTIVITY AND SEVERITY OF COVID-19: RETROSPECTIVE OBSERVATIONAL STUDY	Goldenberg, D.D.; Flowers, R.C.; Werne, R.; Mintz, J.O.E.L.; Kumar, N.E.I.L.; De Andrade Arnaud, F.; Medina, J.A.; Zaw, C.; Rozenbaum Lerner, G.U.I.L.; Lopez Ruiz, A.; Gosine, A.D.; Quartin, A.	2023	Chest	164	4	A1592-A1593	10.1016/j.chest.2023.07.1094	Goldenberg 2023	Irrelevant	Excluded by TI/AB review

S4 Table. All studies identified in the literature search

2894	81.1 Standardization of Diagnosis of Somatic Symptom Disorders in the Emergency Department and Inpatient Settings	Sherker, J.L.	2023	Journal of the American Academy of Child and Adolescent Psychiatry	62	10	S118-S119	10.1016/j.jaac.2023.07.524	Sherker 2023	Irrelevant	Excluded by TI/AB review

S4 Table. All studies identified in the literature search

2895	The Association Between Sociodemographic Factors, Social Determinants of Health, and Spine Surgical Patient Portal Utilization	Owolo, E.; Petitt, Z.; Charles, A.; Ba√´ta, C.; Poehlein, E.; Green, C.; Cook, C.; Sperber, J.; Chandiramani, A.; Roman, M.; Rory Goodwin, C.; Erickson, M.	2023	Clinical Spine Surgery	36	8	301-309	10.1097/BSD.0000000000001458	Owolo 2023	Irrelevant	Excluded by TI/AB review

S4 Table. All studies identified in the literature search

2896	Impact of individual, household, and area characteristics on health and social care outcomes for people with multimorbidity: [illegible]	MacRae, C.; Mercer, S.W.; Lawson, A.; Marshall, A.; Pearce, J.; Abubakar, E.; Zheng, C.; van den Akker, M.; Williams, T.; Swann, O.; Pollock, L.; Rawlings, A.; Fry, [illegible]	2023	PLoS ONE	18	#####	NA	10.1371/journal.pone.0282867	MacRae 2023	Irrelevant	Excluded by TI/AB review
2898	ASSESSMENT OF AREA DEPRIVATION INDICES AND OUTCOMES IN PEDIATRIC PATIENTS WITH INFLAMMATORY BOWEL DISEASE	Hasan, F.; Kim, V.; Silver, E.; Tomer, G.	2023	Journal of Pediatric Gastroenterology and Nutrition	77	1	S452-S454	NA	Hasan 2023	Irrelevant	Excluded by TI/AB review

S4 Table. All studies identified in the literature search

2899	Capturing emergency dispatch address points as geocoding candidates to quantify delimited confidence in residential geolocation	Klaus, C.A.; Henry, K.A.; Il'yasova, D.	2023	International journal of health geographics	22	1	25	10.1186/s12942-023-00347-2	Klaus 2023	Irrelevant	Excluded by TI/AB review

S4 Table. All studies identified in the literature search

2900	Incidence of Discharge Against Medical Advice in Queensland Hospital Emergency Departments Among Indigenous Patients from 2016 to 2021	Mahmoud, I.; Sanjida, S.; Schwenn, P.; Hashem, I.A.; Collin, H.; Chu, K.; Bainbridge, R.; Hou, X.-Y.	2023	Journal of racial and ethnic health disparities	NA	(Mahmoud I.) Department of Family and Community Medicine and Behavioral Sciences, College of Medicine, University of Sharia[illegible]	NA	10.1007/s40615-023-01786-0	Mahmoud 2023	Irrelevant	Excluded by TI/AB review

S4 Table. All studies identified in the literature search

2901	Flexible-Interval High-Sensitivity Troponin Velocity for the Detection of Acute Coronary Syndromes	Suh, E.H.; Probst, M.A.; Tichter, A.M.; Ranard, L.S.; Amaranto, A.; Chang, B.C.; Huynh, P.A.; Kratz, A.; Lee, R.J.; Rabbani, L.E.; Sacco, D.L.; Einstein, A.J.	2023	American Journal of Cardiology	203	(Suh E.H., ehs2109@columbia.edu; Probst M.A.; Chang B.C.; Sacco D.L.)	240-247	10.1016/j.amjcard.2023.06.080	Suh 2023	Irrelevant	Excluded by TI/AB review
2902	The role of traditional social determinants of health in referral patterns, timing of surgery, and Chiari Health Index for Pediatrics	Kelly, K.A.; Guidry, B.S.; Wong, G.W.; Thomas, H.C.; Girgis, M.; Shannon, C.N.; Wellons, J.C.; Cools, M.J.	2023	Journal of neurosurgery. Pediatrics	NA	(Kelly K.A.; Guidry B.S.; Wong G.W.; Thom[illegible]	6-Jan	10.3171/2023.7.PEDS22114	Kelly 2023	Irrelevant	Excluded by TI/AB review

S4 Table. All studies identified in the literature search

2903	Trial Participation and Outcomes among English-Speaking and Spanish-Speaking Patients with Appendicitis Randomized to Antibiotics: A Secondary Analysis of the CODA Randomized Clinical Trial	Serrano, E.; Voldal, E.C.; Machado-Aranda, D.; Deugarte, D.A.; Kao, L.; Drake, T.; Winchell, R.; Cuschieri, J.; Krishnadasan, A.; Talan, D.A.; Siparsky, N.; Ayoung-Chee, P.; Self, W.H.; McGonagill, P.; Mandell, K.A.; Liang, M.K.; Dodwad, S.-J.; Thompson, C.M.; Padilla, R.M.; Fleischman, R.; Price, T.P.; Jones, A.; Bernardi, K.; Garcia, L.; Evans, H.L.; Sanchez, S.E.; Odom, S.; Comstock, B.A.; Heagerty, P.J.; Lawrence, S.O.;	2023	JAMA Surgery	158	9	901-908	10.1001/jamasurg.2023.2277	Serrano 2023	Irrelevant	Excluded by TI/AB review

S4 Table. All studies identified in the literature search

2904	Racial and Ethnic Disparities in Patients With Inflammatory Bowel Disease: An Online Survey	Shah, S.; Shillington, A.C.; Kabagambe, E.K.; Deering, K.L.; Babin, S.; Capelouto, J.; Pulliam, C.; Patel, A.; LaChappelle, B.; Liu, J.	2023	Inflammatory bowel diseases	NA	(Shah S.; Kabagambe E.K.; Babin S.; Capelouto J.) Department	NA	10.1093/ibd/izad194	Shah 2023	Irrelevant	Excluded by TI/AB review
2905	Primary care and emergency department utilization patterns: Differences between White and Black low-acuity patients	Park, C.S.; Sams, M.; Nobay, F.; Morgan, A.; Adler, D.; Abar, B.	2023	Academic Emergency Medicine	30	9	965-968	10.1111/acem.14729	Park 2023	Irrelevant	Excluded by TI/AB review
2906	Intensive Longitudinal Assessment of Adolescents to Predict Suicidal Thoughts and Behaviors	Auerbach, R.P.; Lan, R.; Galfalvy, H.; Alqueza, K.L.; Cohn, J.F.; Crowley, R.N.; Durham, K.; Joyce, K.J.; Kahn, L.E.; Kamath, R.A.; Morency, L.-P.; Porta, G.; Srinivasan, A.; Zelazny, J.; Brent, D.A.; Allen	2023	Journal of the American Academy of Child and Adolescent Psychiatry	62	9	1010-1020	10.1016/j.jaac.2023.03.018	Auerbach 2023	Irrelevant	Excluded by TI/AB review

S4 Table. All studies identified in the literature search

2907	Referral pathways for chronic pain patients from Canadian emergency departments: emergency physicians‚Äô practices, perspectives, and recommendations	Grant, K.L.; McParland, A.L.; Francispragasam, M.; Oxciano, P.	2023	Canadian Journal of Emergency Medicine	25	9	761-767	10.1007/s43678-023-00566-3	Grant 2023	Irrelevant	Excluded by TI/AB review

S4 Table. All studies identified in the literature search

2908	Racial Disparities in Pediatric Inflammatory Bowel Disease Care: Differences in Outcomes and Health Service Utilization Between Black and White Children	Smith, J.; Liu, C.; Beck, A.; Fei, L.; Brokamp, C.; Meryum, S.; Whaley, K.G.; Minar, P.; Hellmann, J.; Denson, L.A.; Margolis, P.; Dhaliwal, J.	2023	Journal of Pediatrics	260	(Smith J.; Meryum S.; Whaley K.G.; Minar P.; Hellmann J.; Denson L.A.; Margolis P.; Dhaliwal J., Jasbir.dhaliwal@cchmc.org) Division of [illegible]	NA	10.1016/j.jpeds.2023.113522	Smith 2023	Irrelevant	Excluded by TI/AB review
2909	Dietary Supplements for Erectile Dysfunction: Analysis of Marketed Products, [illegible]	Petre, G.C.; Francini-Pesenti, F.; Vitagliano, A.; Grande, G.; Ferlin, A.; Garolla, A.	2023	Nutrients	15	17	NA	10.3390/nu15173677	Petre 2023	Irrelevant	Excluded by TI/AB review

S4 Table. All studies identified in the literature search

2910	Emergency Departments: An Underutilized Resource for Expanding COVID-19 Vaccine Coverage in Children	Hart, R.; Feygin, Y.; Kluthe, T.; Quinn, K.; Rao, S.; Baumer-Mouradian, S.H.	2023	Vaccines	11	9	NA	10.3390/vaccines11091445	Hart 2023	Irrelevant	Excluded by TI/AB review
2911	Do physicians counsel patients regarding radiation risks of CT?: A survey of emergency department patients	Sullivan, E.; Danboise, B.; Hunt, K.; Hamblen, M.; Simmons, M.; Kumar, S.; Richman, P.	2023	American Journal of Emergency Medicine	71	(Sullivan E.; Danboise B.; Hunt K.; Hamblen M.; Simmons	233-234	10.1016/j.ajem.2023.07.004	Sullivan 2023	Irrelevant	Excluded by TI/AB review
2912	Emergency Providers' Knowledge and Attitudes Toward Hospice and Palliative Care: A Cross-Sectional Analysis Across 35 Emergency Departments in the	Adeyemi, O.J.; Siman, N.; Goldfeld, K.S.; Cuthel, A.M.; Bouillon-Minois, J.-B.; Grudzen, C.R.	2023	Journal of Palliative Medicine	26	9	1252-1260	10.1089/jpm.2022.0545	Adeyemi 2023	Irrelevant	Excluded by TI/AB review

S4 Table. All studies identified in the literature search

2913	Expanding Diabetes Screening to Identify Undiagnosed Cases Among Emergency Department Patients	Lee, D.C.; Reddy, H.; Koziatek, C.A.; Klein, N.; Chitnis, A.; Creary, K.; Francois, G.; Akindutire, O.; Femia, R.; Caldwell, R.	2023	Western Journal of Emergency Medicine	24	5	962-966	10.5811/westjem.59957	Lee 2023	Irrelevant	Excluded by TI/AB review

S4 Table. All studies identified in the literature search

2914	Emergency Department Observation Unit Patients Want Evaluation and Treatment for Hypercholesterolemia: A Health Belief Model Study	Ashburn, N.P.; Snavely, A.C.; Stanek, L.S.; Shapiro, M.D.; Rikhi, R.R.; Chado, M.A.; Stopyra, J.P.; Mahler, S.A.	2023	Critical Pathways in Cardiology	22	3	91-94	10.1097/HPC.0000000000000324	Ashburn 2023	Irrelevant	Excluded by TI/AB review
2915	A Qualitative Analysis of Patients' Experiences with an ED Diagnosis of GI Cancer	Adams, A.; Heinert, S.; Sanchez, L.; Karasz, A.; Elena Ramos, M.; Sarkar, S.; Rapkin, B.; In, H.	2023	Academic emergency medicine : official journal of the Society [illegible]	NA	(Adams A.; In H.) Rutgers [illegible]	NA	10.1111/acem.14797	Adams 2023	Irrelevant	Excluded by TI/AB review

S4 Table. All studies identified in the literature search

2916	Association of neighborhood social determinants of health, race and ethnicity, and severe maternal morbidity on the [illegible]	Holtzclaw, R.; Bose-Brill, S.; Fareed, N.	2023	medRxiv	NA	(Holtzclaw R.; Fareed N., naleef.fareed [illegible]	NA	10.1101/2023.08.07.23293765	Holtzclaw 2023	Irrelevant	Excluded by TI/AB review
2917	Identifying violence against persons at a safety-net hospital: Evidence from the first 6 months of implementation	Beauchamp, A.M.; Kalra, A.; Scroggins, H.; Pahl, B.; Pitt, A.; Skaliks, A.; Jetelina, K.K.	2023	Health Services Research	58	4	800-806	10.1111/1475-6773.13997	Beauchamp 2023	Irrelevant	Excluded by TI/AB review

S4 Table. All studies identified in the literature search

2918	Healthcare Utilization and Mortality After Hospice Live Discharge Among Medicare Patients With and Without Alzheimer,Äôs Disease and Related Dementias	Zhang, Y.; Shao, H.; Zhang, M.; Li, J.	2023	Journal of General Internal Medicine	38	10	2272-2278	10.1007/s11606-023-08031-8	Zhang 2023	Irrelevant	Excluded by TI/AB review
2919	Quality of life 1 month after acute pulmonary embolism in emergency department patients	Weekes, A.J.; Davison, J.; Lupez, K.; Raper, J.D.; Thomas, A.M.; Cox, C.A.; Esener, D.; Boyd, J.S.; Nomura, J.T.; Murphy, K.; Ockerse, P.M.; Leech, S.; Johnson, J.; Abrams, E.; Kelly, C.; O'Connell, N.S.	2023	Academic Emergency Medicine	30	8	819-831	10.1111/acem.14692	Weekes 2023	Irrelevant	Excluded by TI/AB review
2920	Disparities in emergency department and urgent care opioid prescribing before and after randomized [illegible]	Crowley, A.P.; Sun, C.; Yan, X.; Navathe, A.; Liao, J.M.; Patel, M.S.; Pagnotti, D.; Shen, Z.; Delgado, M.K.	2023	Academic Emergency Medicine	30	8	809-818	10.1111/acem.14717	Crowley 2023	Irrelevant	Excluded by TI/AB review

S4 Table. All studies identified in the literature search

2921	Evaluation of emergency department treat-and-release encounters after major gastrointestinal	Brajcich, B.C.; Johnson, J.K.; Holl, J.L.; Bilimoria, K.Y.; Ager, M.S.; Chung, J.; Joung, R.H.S.; Iroz, C.B.; Odell, D.D.; Bentrem, D.J.; [illegible]	2023	Journal of Surgical Oncology	128	2	402-408	10.1002/jso.27292	Brajcich 2023	Irrelevant	Excluded by TI/AB review
2922	Disparities in Utilization of Same-Day Discharge Following Appendectomy in [illegible]	Sullivan, G.A.; Sincavage, J.; Reiter, A.J.; Hu, A.J.; Rangel, M.; Smith, C.J.; Ritz, E.M.; Shah, A.N.; Gulack, [illegible]	2023	Journal of Surgical Research	288	(Sullivan G.A.; Reiter A.J.; Hu [illegible]	9-Jan	10.1016/j.jss.2023.02.033	Sullivan 2023	Irrelevant	Excluded by TI/AB review
2923	Designing and developing a digital equity dashboard for the emergency department	Yi, S.; Burke, C.; Reilly, A.; Straube, S.; Graterol, J.; Peabody, C.R.	2023	JACEP Open	4	4	NA	10.1002/emp2.12997	Yi 2023	Irrelevant	Excluded by TI/AB review

S4 Table. All studies identified in the literature search

2924	Evolving Management of Acute Mastoiditis: Analysis of the Pediatric Health Information System Database	Friesen, T.L.; Hall, M.; Ramchandar, N.; Berry, J.G.; Jiang, W.	2023	Otolaryngology - Head and Neck Surgery (United States)	169	2	382-389	10.1002/ohn.286	Friesen 2023	Irrelevant	Excluded by TI/AB review

S4 Table. All studies identified in the literature search

2925	Disparities in adherence and emergency department utilization among people with epilepsy: A machine learning approach	Bensken, W.P.; Vaca, G.F.-B.; Williams, S.M.; Khan, O.I.; Jobst, B.C.; Stange, K.C.; Sajatovic, M.; Koroukian, S.M.	2023	Seizure	110	(Bensken W.P., wpb27@case.edu; Williams S.M.; Stange K.C.; Koroukian S.M.)	169-176	10.1016/j.seizure.2023.06.021	Bensken 2023	Irrelevant	Excluded by TI/AB review

S4 Table. All studies identified in the literature search

2926	Shared Decision-making Lowers Medical Expenditures and the Effect Is Amplified in Racially-Ethnically Concordant Relationships	Brown, T.T.; Hurley, V.B.; Rodriguez, H.P.; Lee, J.; Gupta, N.; Toolsie, G.; Markarian, S.; Valenzuela, S.	2023	Medical Care	61	8	528-535	10.1097/MLR.0000000000001881	Brown 2023	Irrelevant	Excluded by TI/AB review

S4 Table. All studies identified in the literature search

2927	Prehospital and emergency department pediatric readiness for injured children: A statement from the American College of Surgeons Committee on Trauma	Ross, S.W.; Campion, E.; Jensen, A.R.; Gray, L.; Gross, T.; Namias, N.; Goodloe, J.M.; Bulger, E.M.; Fischer, P.E.; Fallat, M.E.	2023	Journal of Trauma and Acute Care Surgery	95	2	E6-E10	10.1097/TA.0000000000003997	Ross 2023	Irrelevant	Excluded by TI/AB review
2928	Predictors of Delayed Diagnosis of Pediatric CNS Tumors in the Emergency Department	Young, A.L.; Monuteaux, M.C.; Cooney, T.M.; Michelson, K.A.	2023	Pediatric Emergency Care	39	8	617-622	10.1097/PEC.0000000000002943	Young 2023	Irrelevant	Excluded by TI/AB review
2929	ESOPHOGEAL LEUKOPLAKIA IN PATIENT WITH BLEEDING GASTRIC ULCER	Gummadi, A.; Kudaravalli, P.; Adu-Gyamfi, K.O.; Chandrasekar, V.; Sridhar, S.; Yap, J.	2023	Journal of Investigative Medicine	71	6	NP109-NP110	10.1177/10815589231176919	Gummadi 2023	Irrelevant	Excluded by TI/AB review

S4 Table. All studies identified in the literature search

2930	Stressful Life Events and Healthcare Utilization Among U.S. Children Aged 2-17 Years	Black, L.I.; Ng, A.E.; Zablotsky, B.	2023	National health statistics reports	NA	190	12-Jan	NA	Black 2023	Irrelevant	Excluded by TI/AB review
2932	Clinical characteristics and long-term outcomes in patients with cyclic vomiting syndrome: A 15-year experience at [illegible]	Partovi, O.; Patel, M.; Kovacic, K.; Petrova, A.; Garacchi, Z.; Venkatesan, T.	2023	Neurogastro enterology and Motility	35	7	NA	10.1111/nm o.14571	Partovi 2023	Irrelevant	Excluded by TI/AB review
2933	Telehealth Follow-Up After Inguinal Hernia Repair in Veterans	Abbitt, D.; Choy, K.; Castle, R.; Jones, T.S.; Wikiel, K.J.; Barnett, C.C.; Moore, J.T.; Robinson, T.N.; Jones, E.L.	2023	Journal of Surgical Research	287	(Abbit t D., daniell e.abbit t@cua nschut [illegible]	186-192	10.1016/j.js s.2023.02.0 30	Abbitt 2023	Irrelevant	Excluded by TI/AB review

S4 Table. All studies identified in the literature search

2934	Intersectional characterization of emergency department (ED) staff experiences of racism: a survey of ED healthcare [illegible]	Cruz-Kan, K.; Dufault, B.; Fesehaye, L.; Kornelsen, J.; Hrymak, C.; Zubert, S.; Ratana, P.; Leeies, M.	2023	Canadian Journal of Emergency Medicine	25	7	617-626	10.1007/s43678-023-00533-y	Cruz-Kan 2023	Irrelevant	Excluded by TI/AB review
2935	Disproportionate impact of abortion restriction: Implications for emergency department clinicians	Ferro, H.P.; Williams, K.; Holbrook, D.S.; O'Conor, K.J.	2023	American Journal of Emergency Medicine	69	(Ferro H.P., hferro1@jhmi.edu; O'Conor K.J., kjo@jhmi.edu) Johns Hopkins University School of	160-166	10.1016/j.ajem.2023.04.022	Ferro 2023	Irrelevant	Excluded by TI/AB review

S4 Table. All studies identified in the literature search

2936	Management of low back pain in Australian emergency departments for culturally and linguistically diverse [illegible]	Chen, Q.; Maher, C.G.; Rogan, E.; Machado, G.	2023	Emergency Medicine Journal	40	7	486-492	10.1136/emermed-2022-212718	Chen 2023	Irrelevant	Excluded by TI/AB review

S4 Table. All studies identified in the literature search

2937	Comparison of long-term survival with continued medical therapy, vagus nerve stimulation, and cranial epilepsy surgery in paediatric patients with drug-resistant epilepsy in the USA: an observational cohort study	Zhang, L.; Hall, M.; Lam, S.K.	2023	The Lancet Child and Adolescent Health	7	7	455-462	10.1016/S2352-4642(23)00082-2	Zhang 2023	Irrelevant	Excluded by TI/AB review

S4 Table. All studies identified in the literature search

2938	Research data warehouse: using electronic health records to conduct population-based observational studies	Chen, W.; Xie, F.; McCarthy, D.P.; Reynolds, K.L.; Lee, M.; Coleman, K.J.; Getahun, D.; Koebnick, C.; Jacobsen, S.J.	2023	JAMIA Open	6	2	NA	10.1093/jamiaopen/ooad039	Chen 2023	Irrelevant	Excluded by TI/AB review

S4 Table. All studies identified in the literature search

2939	Performance of a prehospital HEART score in patients with possible myocardial infarction: A prospective evaluation	Cooper, J.G.; Ferguson, J.; Donaldson, L.A.; Black, K.M.M.; Livock, K.J.; Horrill, J.L.; Davidson, E.M.; Scott, N.W.; Lee, A.J.; Fujisawa, T.; Lee, K.K.; Anand, A.; Shah, A.S.V.; Mills, N.L.	2023	Emergency Medicine Journal	40	7	474-481	10.1136/emermed-2022-213003	Cooper 2023	Irrelevant	Excluded by TI/AB review

S4 Table. All studies identified in the literature search

2940	THE #IVF JOURNEY: CONTENT ANALYSIS OF IN-VITRO-FERTILIZATION VIDEOS ON TIKTOK	Peipert, B.J.; Wu, J.; Taylor-Cho, I.A.; Lunn, S.J.; Swartz, J.J.; Acharya, K.	2023	Fertility and Sterility	120	1	e18-e19	10.1016/j.fertnstert.2023.05.037	Peipert 2023	Irrelevant	Excluded by TI/AB review

S4 Table. All studies identified in the literature search

2941	Availability Of Ccta In Safety-net Hospitals Across The United States	Delshad, J.; Bayona, C.; Weis, T.; Eboh, O.; Zeltser, R.; Hai, O.; Makaryus, A.	2023	Journal of Cardiovascular Computed Tomography	17	4	S82	10.1016/j.jcct.2023.05.203	Delshad 2023	Irrelevant	Excluded by TI/AB review

S4 Table. All studies identified in the literature search

2942	Standardizing and Improving Care for Pediatric Agitation Management in the Emergency Department	Hoffmann, J.A.; Pergjika, A.; Liu, L.; Janssen, A.C.; Walkup, J.T.; Johnson, J.K.; Alpern, E.R.; Corboy, J.B.	2023	Pediatrics	152	1	NA	10.1542/peds.2022-059586	Hoffmann 2023	Irrelevant	Excluded by TI/AB review

S4 Table. All studies identified in the literature search

2943	Real-World Persistence, Adherence, Hypoglycemia, and Health Care Resource Utilization in People With Type 2 Diabetes Who Continued With the Second-Generation Basal Insulin Analog Insulin Glargine 300 Units/mL or Switched to a First-Generation Basal Insuli	Edelman, S.; Goldman, J.; Malone, D.C.; Preblick, R.; Munaga, K.; Li, X.; Gill, J.; Gangi, S.	2023	Clinical Diabetes	41	3	425-434	10.2337/cd22-0096	Edelman 2023	Irrelevant	Excluded by TI/AB review
2944	Association of Chest Radiography With Outcomes in Pediatric Pneumonia: A [illegible]	Geanacopoulos, A.T.; Neuman, M.I.; Lipsett, S.C.; Monuteaux, M.C.; Michelson, K.A.	2023	Hospital Pediatrics	13	7	614-622	10.1542/hpeds.2023-007142	Geanacopoulos 2023	Irrelevant	Excluded by TI/AB review
2945	COMORBIDITIES AND PATIENT-REPORTED STIGMA AMONG PATIENTS STARTING [illegible]	Henry, S.G.; Moulin, A.K.; Ritley, D.; Loureiro, S.F.; Fleuret, J.E.; Tseregounis, I.E.; Magnan, E.M.	2023	Journal of General Internal Medicine	38	(Henry S.G.; Tseregounis I.E.) Intern [illegible]	S307-S308	10.1007/s11606-023-08226-z	Henry 2023	Irrelevant	Excluded by TI/AB review

S4 Table. All studies identified in the literature search

2946	NON-AVOIDABLE EMERGENCY CARE FAILS TO REBOUND AFTER PANDEMIC	Leuchter, R.K.; Mafi, J.N.; Craff, M.; Vangala, S.; Tseng, C.-H.; Arbanas, J.C.; Damberg, C.; Sarkisian, C.; Kahn, K.L.	2023	Journal of General Internal Medicine	38	(Leuchter R.K.; Mafi J.N.; Vangala S.;	S272-S273	10.1007/s11606-023-08226-z	Leuchter 2023	Irrelevant	Excluded by TI/AB review
2947	USE OF COMPANIONS WHO SUPPORT MEDICAL COMMUNICATION AMONG OLDER	Oshita, J.Y.	2023	Journal of General Internal Medicine	38	(Oshita J.Y.) Medicine,Clinical and	S212	10.1007/s11606-023-08226-z	Oshita 2023	Irrelevant	Excluded by TI/AB review
2948	THE EFFECTS OF TELEMEDICINE ON DISPARITIES IN ACCESS TO U.S. AMBULATORY	Mafi, J.N.; Vangala, S.; Craff, M.; Arbanas, J.C.; Cui, M.; Rockwelll, M.S.; Tseng, C.-H.; Skinner, D.; Hadfield, M.;	2023	Journal of General Internal Medicine	38	(Mafi J.N.; Vangala S.; Arbanas J.C.;	S275-S276	10.1007/s11606-023-08226-z	Mafi 2023	Irrelevant	Excluded by TI/AB review
2949	RACIAL DISPARITIES IN OPIOID PRESCRIBING, PATIENT SATISFACTION AND DOWNSTREAM OPIOID MISUSE RISK AFTER	Tiako, M.J.N.; Dolan, A.; Shofer, F.S.; South, E.; Schapira, M.M.; Meisel, Z.	2023	Journal of General Internal Medicine	38	(Tiako M.J.N.) Department of Medicine, Brigham	S201	10.1007/s11606-023-08226-z	Tiako 2023	Irrelevant	Excluded by TI/AB review

S4 Table. All studies identified in the literature search

2950	XYLAZINE WOUNDS, WITHDRAWAL, AND CLINICAL OUTCOMES FOR HOSPITALIZED [illegible]	Thakrar, A.P.; Black, M.; Spadaro, T.; Le, T.; Perrone, J.; Lowenstein, M.	2023	Journal of General Internal Medicine	38	(Thakrar A.P.; Lowenstein M.) Natio [illegible]	S326-S327	10.1007/s11606-023-08226-z	Thakrar 2023	Irrelevant	Excluded by TI/AB review
2951	Outcomes and characteristics of patients hospitalized for COVID-19 in British Columbia, Ontario and Quebec during the Omicron wave	Lee, T.; Cheng, M.P.; Vinh, D.C.; Lee, T.C.; Tran, K.C.; Winston, B.W.; Sweet, D.; Boyd, J.H.; Walley, K.R.; Haljan, G.; McGeer, A.; Lamontagne, F.; Fowler, R.; Maslove, [illegible]	2023	CMAJ open	11	4	E672-E683	10.9778/cmajo.20220194	Lee 2023	Irrelevant	Excluded by TI/AB review
2952	Disparity in Healthcare in Systemic Lupus Erythematosus: A Single-Center Study	Reed, J.; Kim, E.; Ivory, C.	2023	Journal of Rheumatology	50	7	70	10.3899/jrheum.2023-0216	Reed 2023	Irrelevant	Excluded by TI/AB review

S4 Table. All studies identified in the literature search

2953	Incidence of Tendon Ruptures in the US: Comparison of Differences Across Racial Groups	Berkay, F.; Minhas, A.; Krishnamurthy, A.; Lyons, J.	2023	Orthopaedic Journal of Sports Medicine	11	7	NA	10.1177/2325967123S00338	Berkay 2023	Irrelevant	Excluded by TI/AB review
2954	Identifying Racial Disparity in Utilization and Outcomes of Hip Arthroscopy using [illegible]	Lu, Y.; Marigi, E.; Alder, K.; Mickley, J.; Camp, C.; Levy, B.; Krych, A.; Okoroha, K.	2023	Orthopaedic Journal of Sports Medicine	11	7	NA	10.1177/2325967123S00325	Lu 2023	Irrelevant	Excluded by TI/AB review
2955	Injury Patterns and Hospital Admission After Trauma Among People Experiencing Homelessness	Silver, C.M.; Thomas, A.C.; Reddy, S.; Sullivan, G.A.; Plevin, R.E.; Kanzaria, H.K.; Stey, A.M.	2023	JAMA Network Open	6	6	E2320862	10.1001/jamanetworkopen.2023.20862	Silver 2023	Irrelevant	Excluded by TI/AB review

S4 Table. All studies identified in the literature search

2956	Examination of the Accuracy of Existing Overdose Surveillance Systems	Griffith, J.; Chambers, L.C.; Hallowell, B.D.; Gaipo, A.; Mailloux, C.; Baird, J.; Beaudoin, F.L.; Samuels, E.A.	2023	JAMA Network Open	6	6	E2320789	10.1001/jamanetworkopen.2023.20789	Griffith 2023	Irrelevant	Excluded by TI/AB review

S4 Table. All studies identified in the literature search

2957	Factors Associated with Unmet Supportive Care Needs and Emergency Department Visits and Hospitalizations in Ambulatory Oncology	Penedo, F.J.; Natori, A.; Fleszar-Pavlovic, S.E.; Sookdeo, V.D.; Macintyre, J.; Medina, H.; Moreno, P.I.; Crane, T.E.; Moskowitz, C.; Calfa, C.L.; Schlumbrecht, M.	2023	JAMA Network Open	6	6	E2319352	10.1001/jamanetworkopen.2023.19352	Penedo 2023	Irrelevant	Excluded by TI/AB review

S4 Table. All studies identified in the literature search

2958	Influence of Race on Utilization and Outcomes in Shoulder Arthroplasty: A Systematic Review	Zareef, U.; Paul, R.W.; Sudah, S.Y.; Erickson, B.J.; Menendez, M.E.	2023	JBJS Reviews	11	6	NA	10.2106/JBJS.RVW.23.00003	Zareef 2023	Irrelevant	Excluded by TI/AB review

S4 Table. All studies identified in the literature search

2959	Interfacility Transfer of Uninsured vs Insured Patients with ST-Segment Elevation Myocardial Infarction in [illegible]	Ward, M.J.; Nikpay, S.; Shermeyer, A.; Nallamothu, B.K.; Rokos, I.; Self, W.H.; Hsia, R.Y.	2023	JAMA Network Open	6	6	E2317831	10.1001/jamanetworkopen.2023.17831	Ward 2023	Irrelevant	Excluded by TI/AB review
2960	The effects of behavioral health integration in Medicaid managed care on access to [illegible]	McConnell, K.J.; Edelstein, S.; Hall, J.; Levy, A.; Danna, M.; Cohen, D.J.; Lindner, S.; Un√°tzer, J.; Zhu,	2023	Health Services Research	58	3	622-633	10.1111/1475-6773.14132	McConnell 2023	Irrelevant	Excluded by TI/AB review
2961	Qualitative analysis of values and motivation reported by families utilizing a paediatric virtual care emergency clinic launched during the SARS-CoV-2 pandemic	Sanderson, V.; Vujcic, B.; Coulson, S.; Lim, R.	2023	Canadian Journal of Emergency Medicine	25	6	529-533	10.1007/s43678-023-00505-2	Sanderson 2023	Irrelevant	Excluded by TI/AB review

S4 Table. All studies identified in the literature search

2962	Central Retinal Artery Occlusion: Time to Presentation and Diagnosis	Shah, R.; Gilbert, A.; Melles, R.; Patel, A.; Do, T.; Wolek, M.; Vora, R.A.	2023	Ophthalmology Retina	7	6	527-531	10.1016/j.oret.2023.01.005	Shah 2023	Irrelevant	Excluded by TI/AB review
2963	An evaluation of satisfaction with emergency department care in children and adolescents with mental health concerns	Lategan, C.; Newton, A.S.; Thull-Freedman, J.; Stang, A.; Lang, E.; Arnold, P.; Stubbs, M.; Freedman, S.B.	2023	Canadian Journal of Emergency Medicine	25	6	498-507	10.1007/s43678-023-00511-4	Lategan 2023	Irrelevant	Excluded by TI/AB review
2964	The impact of an emergency department peer navigator (EDPN) program in improving clinical outcomes and healthcare utilization in an [illegible]	Santos, C.; Ramdin, C.; Becceril, J.; Al Kik, B.; Jefri, M.; Nelson, L.	2023	American Journal of Emergency Medicine	68	(Santos C., Cynthia.D.Santos@Rutgers.edu; Ramdi[illegible]	16-Oct	10.1016/j.ajem.2023.02.031	Santos 2023	Irrelevant	Excluded by TI/AB review

S4 Table. All studies identified in the literature search

2965	A focused retrospective study on differences in IBD characteristics between Black and White patients in the south	Burstiner, L.S.; Owings, A.H.; Tacy, C.; Perez, M.; Royer, A.; Hreish, Y.; Johnson, J.; Barr, M.; Laird, H.; Tarugu, S.; Robinson, T.O.; Hosseini-Carroll, P.; Liu, J.; Glover, S.C.	2023	American Journal of the Medical Sciences	365	6	488-495	10.1016/j.amjms.2023.02.013	Burstiner 2023	Irrelevant	Excluded by TI/AB review

S4 Table. All studies identified in the literature search

2966	Default bias in medical patient simulators: Differences in availability and procedures	Schwieters, K.R.; Morris, N.L.; Craig, C.M.	2023	Human Factors in Healthcare	3	(Schwieters K.R., schwi154@umn.edu; Morris N.L.; Craig C.M.) Depar	NA	10.1016/j.hfh.2023.100040	Schwieters 2023	Irrelevant	Excluded by TI/AB review
2967	Maximizing Equity in Acute Coronary Syndrome Screening across Sociodemographic Characteristics of Patients	Bunney, G.; Bloos, S.M.; Graber-Naidich, A.; Pasao, M.A.; Kabeer, R.; Kim, D.; Miller, K.; Yiadom, M.Y.A.B.	2023	Diagnostics	13	12	NA	10.3390/diagnostics13122053	Bunney 2023	Irrelevant	Excluded by TI/AB review
2968	Elective Peripheral Vascular Intervention in the Smoking Claudicant	Patel, R.J.; Zarrintan, S.; Gaffey, A.C.; Malas, M.	2023	Journal of Vascular Surgery	77	6	e66-e67	10.1016/j.jvs.2023.03.071	Patel 2023	Irrelevant	Excluded by TI/AB review

S4 Table. All studies identified in the literature search

2969	THE ‚ÄúAFTER HOURS‚Äù EFFECT AND ROLE OF ENDOSCOPY SETTING ON OUTCOMES IN PATIENTS WITH ESOPHAGEAL FOOD IMPACTION: RESULTS FROM A MULTISITE STUDY	Wadhavkar, N.; Roussel, B.; Monteiro, J.; Bains, K.; Bhowmick, K.; Mohamed, M.; Kalligeros, M.; Fine, S.	2023	Gastrointestinal Endoscopy	97	6	AB995	10.1016/j.gie.2023.04.1538	Wadhavkar 2023	Irrelevant	Excluded by TI/AB review

S4 Table. All studies identified in the literature search

2971	OPERATOR-SPECIFIC OUTCOMES IN ENDOSCOPIC SLEEVE GASTROPLASTY: A PROPENSITY-MATCHED ANALYSIS	Ali, H.; Patel, P.; Pamarthy, R.; Sarfraz, S.; Malik, T.; Inayat, F.; Farooq, M.; Advani, R.; Watson, R.	2023	Gastrointestinal Endoscopy	97	6	AB28-AB29	10.1016/j.gie.2023.04.083	Ali 2023	Irrelevant	Excluded by TI/AB review

S4 Table. All studies identified in the literature search

2972	RACIAL DISPARITIES IN ENDOSCOPIC SLEEVE GASTROPLASTY IN THE UNITED STATES	Ali, H.; Patel, P.; Pamarthy, R.; Malik, T.; Sarfraz, S.; Mohan, B.; Poola, S.; Asokkumar, R.; Nava, G.L.; Adler, D.	2023	Gastrointestinal Endoscopy	97	6	AB26	10.1016/j.gie.2023.04.080	Ali 2023	Irrelevant	Excluded by TI/AB review
2973	PCR130 Beliefs Regarding Patient Experience in the Emergency Department Among Individuals Living with Sickle Cell Disease	Ibiloye, E.; Odonkor, G.N.; Barner, J.C.; Brown, C.M.; Kang, H.A.; Bamgbade, B.A.	2023	Value in Health	26	6	S336	10.1016/j.jval.2023.03.1906	Ibiloye 2023	Irrelevant	Excluded by TI/AB review

S4 Table. All studies identified in the literature search

2975	P-409 Outcomes of patients with unresectable hepatocellular carcinoma who developed an infection while receiving atezolizumab	Esmail, A.; Xu, J.; Burns, E.; Gee, K.; Wiechmann, C.; Zhang, Y.; Kieser, R.; Gong, Z.; Umoru, G.; Shah, S.; Sun, K.; Heyne, K.; Abdelrahim, M.	2023	Annals of Oncology	34	(Esmail A.; Xu J.; Burns E.; Gee K.; Wiechmann C.; Zhang Y.; Umoru G.; Shah S.; Heyne K.; Abdelrahim M.) Houston Methodist [illegible]	S156	10.1016/j.annonc.2023.04.465	Esmail 2023	Irrelevant	Excluded by TI/AB review

S4 Table. All studies identified in the literature search

2977	Decreasing Racial Disparities in Preventable Emergency Department Visits Through Hospital Health Information Technology [illegible]	Wang, N.; Chen, J.	2023	Telemedicine journal and e-health : the official journal of the American Telemedicin [illegible]	29	6	841-850	10.1089/tmj.2022.0199	Wang 2023	Irrelevant	Excluded by TI/AB review
2978	Advance Care Planning Impacts on Downstream Healthcare Utilization for High-risk Patients with Metastatic Cancer [illegible]	Hall, J.; Taparra, K.; Bagshaw, H.; Pollom, E.; Gensheimer, M.; Chin, A.	2023	American Journal of Clinical Oncology: Cancer Clinical Trials	46	6	S90	10.1097/COC.0000000000001009	Hall 2023	Irrelevant	Excluded by TI/AB review
2979	Patterns of Care for Central Retinal Artery Occlusion (CRAO): A Retrospective, Observational, Cohort Study	Lee, T.; Hsu, S.T.; Boisvert, C.J.; Grory, B.M.	2023	Investigative Ophthalmology and Visual Science	64	8	4106	NA	Lee 2023	Irrelevant	Excluded by TI/AB review
2980	Do the Right Thing-Intensive Intervention and Resulting Costs for Youth with High Social Risk and Type 1 Diabetes (T1D)	Reed, A.; Wong, J.C.; Naranjo, D.; Padmanabhan, R.; Navarro, R.; Koblinski, E.; Torres, K.A.; Cruz, C.; Harris, M.A.; Wagner, D.V.	2023	Diabetes	72	(Reed A.; Wong J.C.; Naranjo D.; Padmanabh [illegible]	NA	10.2337/db23-53-LB	Reed 2023	Irrelevant	Excluded by TI/AB review

S4 Table. All studies identified in the literature search

2981	THE JOURNEY TOWARD DISEASE MANAGEMENT : A NATIONAL SURVEY OF GOUT [illegible]	Cooper, J.; Winokur, M.; Serota, K.	2023	Annals of the Rheumatic Diseases	82	(Cooper J.; Winokur M.; Serota K.) [illegible]	2104	10.1136/annrheumdis-2023-eular.602	Cooper 2023	Irrelevant	Excluded by TI/AB review
2982	Adverse Clinical Outcomes Among Patients With Acute Low-risk Pulmonary Embolism and Concerning Computed Tomography [illegible]	O'Hare, C.; Grace, K.A.; Schaeffer, W.J.; Hyder, S.N.; Stover, M.; Liles, A.L.; Khaja, M.S.; Cranford, J.A.; Kocher, K.E.; Barnes, G.D.; Greineder, C.F.	2023	JAMA Network Open	6	5	E2311455	10.1001/jamanetworkopen.2023.11455	O'Hare 2023	Irrelevant	Excluded by TI/AB review
2983	Health mediation: an intervention mode for improving emergency department care and support for patients living in precarious [illegible]	Na√Øt Salem, R.; Rotily, M.; Apostolidis, T.; Odena, S.; Lamouroux, A.; Chischportich, C.; Persico, N.; Auquier, P.	2023	BMC health services research	23	1	495	10.1186/s12913-023-09522-4	Na√ØtSalem 2023	Irrelevant	Excluded by TI/AB review

S4 Table. All studies identified in the literature search

2984	School-Based Telemedicine and Reduced Avoidable Emergency Care Among Rural Pediatric Patients	Mayfield, C.A.; Priem, J.S.; Effinger, T.; McGinnis, S.; Grinton, P.	2023	Telemedicine journal and e-health : the official journal of the American Telemedicine Association	NA	(Mayfield C.A.) Department of Community Health	NA	10.1089/tmj.2023.0013	Mayfield 2023	Irrelevant	Excluded by TI/AB review
2985	Characteristics and health service use of Medicaid-insured individuals filling naloxone under a standing order in Louisiana	Sugarman, O.K.; Breithaupt, J.; Wang, X.; Bachhuber, M.A.	2023	Journal of the American Pharmacists Association	63	3	904-908.e1	10.1016/j.japh.2022.12.025	Sugarman 2023	Irrelevant	Excluded by TI/AB review
2986	Black Americans Nearing End of Life in Home Health: Structural Vulnerability and [illegible]	Bigger, S.; Doyon, K.; Glenn, L.	2023	Journal of Pain and Symptom Management	65	5	e645-e646	10.1016/j.jpainsymman.2023.02.261	Bigger 2023	Irrelevant	Excluded by TI/AB review
2987	Becoming More Common: Managing an Uncommon Disease with Unexpected [illegible]	Rouchou, B.; Jersak, T.; Linebarger, J.S.	2023	Journal of Pain and Symptom Management	65	5	e545-e546	10.1016/j.jpainsymman.2023.02.074	Rouchou 2023	Irrelevant	Excluded by TI/AB review

S4 Table. All studies identified in the literature search

2988	Efficacy and tolerability of oral morphine versus oxycodone/ acetaminophen for analgesia in the emergency department	Harvey, H.; Cacciapuoti, M.; Ramadan, L.; Brodie, K.; Gibson, C.; Tefera, E.; Yu, G.G.; Motov, S.M.; Nelson, L.S.; Mazer-Amirshahi, M.	2023	Journal of Opioid Management	19	3	273-280	10.5055/jom.2023.0782	Harvey 2023	Irrelevant	Excluded by TI/AB review

S4 Table. All studies identified in the literature search

2989	Structural Racism in Behavioral Health Presentation and Management	Rainer, T.; Lim, J.K.; He, Y.; Perdomo, J.; Nash, K.A.; Kistin, C.J.; Tolliver, D.G.; McIntyre, E.; Hsu, H.E.	2023	Hospital Pediatrics	13	5	461-470	10.1542/hpeds.2023-007133	Rainer 2023	Irrelevant	Excluded by TI/AB review

S4 Table. All studies identified in the literature search

2990	Advanced practice provider‚Äìled clinic for care transitions in newly diagnosed venous thromboembolism: establishment and utilization	Frank, C.; Kasthuri, R.; Key, N.S.; Mooberry, M.; Wilson, S.R.; Moll, S.	2023	Research and Practice in Thrombosis and Haemostasis	7	4	NA	10.1016/j.rpth.2023.100198	Frank 2023	Irrelevant	Excluded by TI/AB review

S4 Table. All studies identified in the literature search

2991	Medical-Legal Partnership Effects on Mental Health, Health Care Use, and Quality of Life in Primary Care: A Randomized Clinical Trial	Liaw, W.; Northrup, T.F.; Stotts, A.L.; Bakos-Block, C.; Suchting, R.; Chen, A.; Hernandez, A.; Finzetto, L.; Green, C.; Murphy, T.	2023	Journal of the American Board of Family Medicine	36	3	414-424	10.3122/JABFM.2022.220349R1	Liaw 2023	Irrelevant	Excluded by TI/AB review

S4 Table. All studies identified in the literature search

2992	Care processes and outcomes of deprivation across the clinical course of kidney disease: findings from a high-income country with universal healthcare	Sawhney, S.; Blakeman, T.; Blana, D.; Boyers, D.; Fluck, N.; Nath, M.; Methven, S.; Rzewuska, M.; Black, C.	2023	Nephrology Dialysis Transplantation	38	5	1170-1182	10.1093/ndt/gfac224	Sawhney 2023	Irrelevant	Excluded by TI/AB review

S4 Table. All studies identified in the literature search

2994	Artificial Urinary Sphincter Outcomes and Durability: Equivalent Surgical Success Among Diverse Patient Characteristics	Vasan, R.; Miller, D.; Patnaik, S.; Taylor, C.; Morrill, C.; Rusilko, P.J.	2023	Journal of the American College of Surgeons	236	5	S141-S142	10.1097/01.XCS.0000933576.88165.e9	Vasan 2023	Irrelevant	Excluded by TI/AB review

S4 Table. All studies identified in the literature search

2995	Diagnostic Delays of MSK Infections and Clinical Pathway Execution	Grubenhoff, J.; Bakel, L.A.; Searns, J.	2023	Diagnosis	10	2	A11-A12	NA	Grubenhoff 2023	Irrelevant	Excluded by TI/AB review

S4 Table. All studies identified in the literature search

2996	Nothing About Us Without Us: Creation of a Family Advisory Board to Drive Diagnostic Equity	Mack, A.; Schaffer, J.; Rasooly, I.; Nawab, U.; Kratchman, A.; Colfer, A.	2023	Diagnosis	10	2	A23-A24	NA	Mack 2023	Irrelevant	Excluded by TI/AB review

S4 Table. All studies identified in the literature search

2997	Social Determinants of Health Curriculum for Fourth-Year Medical Students Rotating in an Urban, Safety-Net Emergency Department	Koul, R.; Mayo, K.; Kim, A.	2023	Western Journal of Emergency Medicine	24	3.1	S60-S61	10.5811/westjem.61126	Koul 2023	Irrelevant	Excluded by TI/AB review

S4 Table. All studies identified in the literature search

2998	Engagement in a Longitudinal Nurse-Led Supportive Care Program for Persons Living With Advanced Cancer	Yamarik, R.L.; Chiu, L.A.; Mara, F.; Cuthel, A.; Adeyemi, O.J.; Van Allen, K.; Goldfeld, K.; Grudzen, C.; Brody, A.A.; Schrag, D.	2023	Academic Emergency Medicine	30	(Yamarik R.L.) Long Beach Veterans Affairs, United States (Chiu L.A.; Mara F.; Van Allen K.; Goldfeld K.) New York University [illegible]	260	10.1111/acem.14718	Yamarik 2023	Irrelevant	Excluded by TI/AB review

S4 Table. All studies identified in the literature search

2999	Emergency Care Experiences of Persons With Dementia: A Qualitative Study With Care Partners (AGEM and ADIEM Sponsored	Chary, A.N.; Ramont, V.; Obi, T.; Santangelo, I.; Rivera, A.; Hernandez, N.; Naik, A.; Liu, S.W.; Kennedy, M.	2023	Academic Emergency Medicine	30	(Chary A.N.) Brigham and Womens Hospital, Harvard Medical School, United States (Ramont V.; Rivera A.; Hernandez [illegible]	263	10.1111/acem.14718	Chary 2023	Irrelevant	Excluded by TI/AB review

S4 Table. All studies identified in the literature search

3000	Outcomes of Patients That Rule Out Within One Hour Using High-Sensitivity Cardiac Troponin Protocols	Connor, S.; Nassereddine, H.; Morton, T.; Klausner, H.A.; Cook, B.; Tuttle, J.; Wanis, N.; Gunaga, S.; Xu, N.; Wittenberg, S.; Krupp, S.S.; McCord, J.; Miller, J.	2023	Academic Emergency Medicine	30	(Nassereddine H.; Klausner H.A.; Tuttle J.; Wanis N.; McCord J.) Henry Ford Health System, United States (Gunaga S.) Henry Ford	240	10.1111/acem.14718	Connor 2023	Irrelevant	Excluded by TI/AB review

S4 Table. All studies identified in the literature search

3001	Inequities Among Emergency Department Hallway Utilization	Tuffuor, K.; Sangal, R.; Su, H.; Meng, L.; Pinker, E.; Tarabar, A.; Van Tonder, R.; Ulrich, A.; Venkatesh, A.K.; Parwani, V.	2023	Academic Emergency Medicine	30	(Tuffuor K.) Yale University, School of Medicine, United States (Sangal R.; Su H.; Tarabar A.; Van Tonder R.; Ulrich A.; Venkatesh A.K.; Parwa[illegible]	310	10.1111/acem.14718	Tuffuor 2023	Irrelevant	Excluded by TI/AB review

S4 Table. All studies identified in the literature search

3002	The Impact of Inpatient Unit Occupancy on Emergency Department Boarding Time	Huang, S.; Scheulen, J.J.; Fahimi, J.; Herzke, C.; Lautzenheiser, M.; Rupani, H.; Biddison, L.D.; Dezube, R.; Iwashyna, T.; Engineer, L.; Berry, S.	2023	Academic Emergency Medicine	30	(Huang S.; Scheulen J.J.; Lautzenheiser M.; Rupani H.; Biddison L.D.; Dezube R.) Johns Hopkins Hospital, United States (Fahimi J.) [illegible]	83	10.1111/acem.14718	Huang 2023	Irrelevant	Excluded by TI/AB review

S4 Table. All studies identified in the literature search

3003	Consensus-Based Best Practice Guidelines for Performing Educational Ultrasound in the Emergency Department	Chao, S.; Liu, Y.T.; Taylor, L.A.; Tucker, R.V.; Huang, R.	2023	Academic Emergency Medicine	30	(Chao S.; Tucker R.V.; Huang R.) University of Michigan, United States (Liu Y.T.) Harbor-UCLA Medical Center, [illegible]	332	10.1111/acem.14718	Chao 2023	Irrelevant	Excluded by TI/AB review

S4 Table. All studies identified in the literature search

3004	From the Emergency Department to Sustained Virologic Response for Hepatitis C (Emergency Medicine Transmissible Infectious Disease and Epidemics (EMTIDE) Interest Group Sponsored)	Wilson, L.; Walter, L.; Farmer, M.; Franco, R.; Roberson, T.F.	2023	Academic Emergency Medicine	30	(Walter L.; Franco R.) University of Alabama at Birmingham, United States (Farmer M.; Roberson T.F.) UAB Heersink School of Medic[illegible]	105	10.1111/acem.14718	Wilson 2023	Irrelevant	Excluded by TI/AB review

S4 Table. All studies identified in the literature search

3005	Features and Outcomes of Patients With Traumatic Subarachnoid Hemorrhage and Comorbid Neurological Injury	Rinaldi, R.; Mileto, A.J.; May, P.; Melnychuk, E.	2023	Academic Emergency Medicine	30	(Rinaldi R.) Geisinger Commonwealth School of Medicine, Unite [illegible]	13-Dec	10.1111/acem.14718	Rinaldi 2023	Irrelevant	Excluded by TI/AB review
3006	The Effect of Community-Acquired Pneumonia on 6-Month Employment Loss in Older Patients	Resser, J.; Han, J.H.; Baughman, A.; Hart, K.W.; Johnson, K.D.; Finelli, L.; Weiss, T.W.; Self, W.H.	2023	Academic Emergency Medicine	30	(Resser J.; Baughman A.; Hart K.W.; [illegible]	152-153	10.1111/acem.14718	Resser 2023	Irrelevant	Excluded by TI/AB review

S4 Table. All studies identified in the literature search

3007	Rapid Diagnostic Testing of Gonorrhea and Chlamydia Improves Antimicrobial Stewardship in the Emergency Department	Rech, M.; Smith, A.; Harrington, A.; Santarossa, M.; Adams, J.; Acquisto, N.	2023	Academic Emergency Medicine	30	(Rech M.) Loyola University Medical Center, United States (Acquisto N.) University of Rochester Medical Center, Unite[illegible]	153-154	10.1111/acem.14718	Rech 2023	Irrelevant	Excluded by TI/AB review

S4 Table. All studies identified in the literature search

3008	Predictors of Emergency Department Return in Patients Presenting With Skin and Soft Tissue Infections in an Urban, Tertiary Care Emergency Department	Simpson, B.; Han, K.; Yee, S.; Lykins, J.D.; Aurora, T.	2023	Academic Emergency Medicine	30	(Simpson B.; Han K.; Yee S.; Lykins J.D.; Aurora T.) Virginia Commonwealth University, United States	154	10.1111/acem.14718	Simpson 2023	Irrelevant	Excluded by TI/AB review

S4 Table. All studies identified in the literature search

3009	Women With Chest Pain Have Lower Hospitalization, Cardiac Testing, and Adverse Outcome Rates	Byouk, J.; Davis, J.; Snavely, A.C.; Ashburn, N.P.; Nicacio, D.; Pearson, D.A.; Stopyra, J.P.; Mahler, S.A.	2023	Academic Emergency Medicine	30	(Snavely A.C.; Ashburn N.P.) Wake Forest University, School of Medicine, United States (Nicacio D.; Pearson D.A.) Carolinas Medical [illegible]	16	10.1111/acem.14718	Byouk 2023	Irrelevant	Excluded by TI/AB review

S4 Table. All studies identified in the literature search

3010	Illicit Fentanyl Use in Emergency Department Patients: A Descriptive Analysis of Demographics and Outcomes (Social EM and [illegible]	Ryan, Z.; Walter, L.; Robinett, D.A.; Li, L.; Brown, J.; McNellage, L.; Edwards, A.R.; Booth, J.S.; Shufflebarger, E.; McCleskey, B.	2023	Academic Emergency Medicine	30	(Ryan Z.) University of Alabama, Birmingha[illegible]	246	10.1111/acem.14718	Ryan 2023	Irrelevant	Excluded by TI/AB review

S4 Table. All studies identified in the literature search

3011	Nonwhite Patients With Acute Chest Pain Have Lower Hospitalization, Cardiac Testing, and Adverse Outcome Rates	Davis, J.; Byouk, J.; Snavely, A.C.; Ashburn, N.P.; Nicacio, D.; Stopyra, J.P.; Pearson, D.A.; Mahler, S.A.	2023	Academic Emergency Medicine	30	(Snavely A.C.; Ashburn N.P.) Wake Forest University, School of Medicine, United States (Nicacio D.; Pearson D.A.) Carolinas Medical al	16-17	10.1111/acem.14718	Davis 2023	Irrelevant	Excluded by TI/AB review

S4 Table. All studies identified in the literature search

3012	Emergency Department-Initiated Medication for Opioid Use Disorder: Impact on Quality of Life	Walter, L.; Covington, W.L.; Hudson, J.	2023	Academic Emergency Medicine	30	(Walter L.) University of Alabama at Birmingham, United States (Covington W.L.) University of Alabama, United States (Huds[illegible]	247	10.1111/acem.14718	Walter 2023	Irrelevant	Excluded by TI/AB review

S4 Table. All studies identified in the literature search

3013	Trauma, Pregnancy, and Prisons, Oh My: An Undergraduate Medical Education Elective on Incarceration and Health	Suh, M.I.	2023	Academic Emergency Medicine	30	(Suh M.I.) Baylor College of Medicine, United States	415	10.1111/acem.14718	Suh 2023	Irrelevant	Excluded by TI/AB review

S4 Table. All studies identified in the literature search

3015	Who Do Emergency Departments Really Serve: A Comparative Analysis of Hospital Patients and Local Populations	Morris, J.; Zachrison, K.S.; Boggs, K.M.; Gao, J.; Espinola, J.A.; Cash, R.E.; Camargo, C.A.; Samuels-Kalow, M.E.	2023	Academic Emergency Medicine	30	(Morris J.; Zachrison K.S.; Cash R.E.) Harvard Medical School, Massachusetts General Hospital, United States (Boggs [illegible]	136-137	10.1111/acem.14718	Morris 2023	Irrelevant	Excluded by TI/AB review

S4 Table. All studies identified in the literature search

3016	Prevalence of Behavioral Flags in the Electronic Health Record Among Black and White Emergency Department Patients	Agarwal, A.K.; Seeburger, E.F.; O'Neill, G.; Nwakanma, C.; Marsh, L.; Soltany, K.; South, E.; Friedman, A.	2023	Academic Emergency Medicine	30	(Agarwal A.K.; Seeburger E.F.; O'Neill G.; Nwakanma C.; Marsh L.; Soltany K.; South E.; Friedman A.) University of Pennsylvani[illegible]	340-341	10.1111/acem.14718	Agarwal 2023	Irrelevant	Excluded by TI/AB review

S4 Table. All studies identified in the literature search

3017	Measuring Errors in Language Preference Data: Gaps in Achieving Health Equity	Amin, D.; Rizvanolli, L.; Reyes, J.; Haidar, Z.; Christian, E.	2023	Academic Emergency Medicine	30	(Amin D.) Cook County Stroger Hospital, United States (Rizvanolli L.; Christian E.) Department of Emergency Medicine, Cook Count[illegible]	182	10.1111/acem.14718	Amin 2023	Irrelevant	Excluded by TI/AB review

S4 Table. All studies identified in the literature search

3019	Factors Associated With Door-In to Door-Out Goal Times in Stroke Transfers: Findings From	Royan, R.; Stamm, B.; Giurcanu, M.; Messe, S.; Jauch, E.C.; Prabhakaran, S.	2023	Academic Emergency Medicine	30	(Royan R.) Northwestern University, Feinberg School of Medicine-McGaw, United States (Stamm B.) Northwestern University, [illegible]	92	10.1111/acem.14718	Royan 2023	Irrelevant	Excluded by TI/AB review
3020	Addressing Gaps in LGBTQ Care in Emergency Medicine: A Mixed-Methods Study	Wang, V.J.; Terrazos-Moreno, R.; Jubanyik, K.; Olezeski, C.	2023	Academic Emergency Medicine	30	(Wang V.J.; Terrazos-More [illegible]	252	10.1111/acem.14718	Wang 2023	Irrelevant	Excluded by TI/AB review

S4 Table. All studies identified in the literature search

3021	Comparison of Severity and Outcomes of New Onset Diabetes Before and During the COVID-19 pandemic	Talukder, M.H.; Sethuramun, U.; Okaruefe, C.O.; Sambor, P.; Oliphant, K.; Arkatkar, A.; Sundaralingam, S.; Prabhu, J.; Spencer, P.; Kannikeswaran, N.	2023	Academic Emergency Medicine	30	(Sethuramun U.; Okaruefe C.O.) Division of Emergency Medicine, Children's Hospital of Michigan, Central Michigan University, United [illegible]	228	10.1111/acem.14718	Talukder 2023	Irrelevant	Excluded by TI/AB review

S4 Table. All studies identified in the literature search

3022	Health Equity in Stroke Prophylaxis for New Atrial Fibrillation in the Emergency Department	Anwar, M.; Hopkins, B.; McCracken, C.; Kinney, E.; Kim, J.; Nguyen, T.; Bissell, K.; Gosman, A.; DeCarlo, A.; Kea, B.	2023	Academic Emergency Medicine	30	(Anwar M.; Bissell K.; Gosman A.; DeCarlo A.) Oregon Health and Science University, United States (Hopkins B.; Nguyen T.) OHSU-[illegible]	207	10.1111/acem.14718	Anwar 2023	Irrelevant	Excluded by TI/AB review

S4 Table. All studies identified in the literature search

3023	Use of an Integrated Communication Tool Improves Stroke Care for Patients With Large Vessel Occlusion	Affeldt, Z.S.; Sabayan, B.; Droegemueller, C.J.; Brogan, M.E.; Kashyap, B.; Herrmann, A.; Stanfield, S.C.; Westgard, B.C.; Jeffrey, L.; Jason, C.; Torok, C.; Malek, A.; Nyamao, T.; Seth, S.; Dizon, J.; Graff, J.S.;	2023	Academic Emergency Medicine	30	(Affeldt Z.S.; Sabayan B.; Droegemueller C.J.; Brogan M.E.;	94	10.1111/acem.14718	Affeldt 2023	Irrelevant	Excluded by TI/AB review
3024	COVID-19 Complications in Pediatric Patients: Machine Learning Models Identify Early Predictors of Severe Outcomes	Badaki-Makun, O.; Levin, S.; Hinson, J.S.; Zhao, X.; Smith, A.; Naim, S.; Lau, B.	2023	Academic Emergency Medicine	30	(Badaki-Makun O.; Levin S.; Hinson J.S.) Beckman Coulter	256	10.1111/acem.14718	Badaki-Makun 2023	Irrelevant	Excluded by TI/AB review

S4 Table. All studies identified in the literature search

3025	Automated Measures of Mis-triage to Assess Accuracy of the Emergency Severity Index Among Pediatric Patients	Zhang, J.Y.; Warton, M.; Reed, M.E.; Rauchwerger, A.S.; Vitale-McDowell, T.J.; McGauhey, K.R.; Ballard, D.W.; Kene, M.V.; Sax, D.R.	2023	Academic Emergency Medicine	30	(Zhang J.Y.; Warton M.; Reed M.E.; Rauchwerger A.S.) Kaiser Perma	233	10.1111/acem.14718	Zhang 2023	Irrelevant	Excluded by TI/AB review
3027	Enhancing an Expedited Partner Therapy Program With an Electronic Alert System in the Emergency Department	Loszewski, C.; Rammal, J.-A.; Nassereddine, H.; Miller, J.; MacDonald, N.C.; Klausner, H.A.; Clarke, B.; Manteuffel, J.	2023	Academic Emergency Medicine	30	(Loszewski C.; Nassereddine H.; Klausner H.A.; Manteuffel J.) Henry Ford Health System, Unite	237	10.1111/acem.14718	Loszewski 2023	Irrelevant	Excluded by TI/AB review

S4 Table. All studies identified in the literature search

3028	Predictors of Death in Acute Care Settings Among Older Adults With Advanced Cancer	Yilmaz, S.; Loh, K.P.; Mohamed, M.; Adler, D.H.; Epstein, R.; Wittink, M.; Kehoe, L.; Arana-Chicas, E.; Gudina, A.; Hardy, S.; McGuire, J.; Rieth, K.; Tylock, R.; Wells, M.; Anthony, N.; Giguere, J.; Jamieson, L.; Mohile, S.	2023	Academic Emergency Medicine	30	(Yilmaz S.) University of Rochester, School of Medicine and Dentistry, United States (Adler D.H.) University of Rochester, Unite	54	10.1111/acem.14718	Yilmaz 2023	Irrelevant	Excluded by TI/AB review

S4 Table. All studies identified in the literature search

3029	Disparities in Hospital Length of Stay Among Severely Ill Patients With Community Acquired Sepsis and Acute Respiratory Failure Across 27 Hospitals	Chesley, C.F.; Chowdhury, M.; Small, D.S.; Schaubel, D.; Liu, V.; Lane-Fall, M.B.; Halpern, S.D.; Anesi, G.L.	2023	American Journal of Respiratory and Critical Care Medicine	207	1	NA	10.1164/ajrccm-conference.2023.B16	Chesley 2023	Irrelevant	Excluded by TI/AB review

S4 Table. All studies identified in the literature search

3030	Inspiring Health Advances in Lung Care (INHALE): An Opportunity to Address Pediatric Asthma Health Disparities in Michigan	Lewis, T.C.; Lugogo, N.L.; Sjoding, M.W.; Freed, G.; Sagana, R.L.; Kovalski, A.; Dowling, J.; Vorce, T.; Mohan, A.; Zinsser, D.; Oliver, C.; Stoermer-Grossman, K.; Farhat, N.	2023	American Journal of Respiratory and Critical Care Medicine	207	1	NA	10.1164/ajrccm-conference.2023.C102	Lewis 2023	Irrelevant	Excluded by TI/AB review

S4 Table. All studies identified in the literature search

3031	A National Partnership With the COPD Foundation to Implement the COPD Virtual Medication Reconciliation and Education: COPD V-M(ED) Pilot - An Interim Analysis	Gray, S.E.; Austin, J.; Willard, K.; Press, V.G.	2023	American Journal of Respiratory and Critical Care Medicine	207	1	NA	10.1164/ajrccm-conference.2023.C102	Gray 2023	Irrelevant	Excluded by TI/AB review
3032	Medication Non-adherence and Health-related Outcomes in	Robbins, E.W.; Bradley, K.V.; Baird, G.L.; Badesch, D.B.; Burger, C.D.;	2023	American Journal of Respiratory and Critical	207	1	NA	10.1164/ajrccm-conference.2023.A26	Robbins 2023	Irrelevant	Excluded by TI/AB review

S4 Table. All studies identified in the literature search

3033	An emergency department quality improvement intervention for decreasing time to pain medication in sickle cell disease	Bailey, R.; Tunji-Ajayi, L.; Thompson, S.; Poku, M.; Appiah-Boateng, R.; Navarro, N.; Sheikh, H.; Hulme, J.; Gulilat, M.; Bryan, J.	2023	Canadian Journal of Emergency Medicine	25	(Bailey R.; Tunji-Ajayi L.; Thompson S.; [illegible]	S26-S27	10.1007/s43678-023-00508-z	Bailey 2023	Irrelevant	Excluded by TI/AB review
3034	Bridging the gap: a qualitative assessment of multi-disciplinary emergency service provider's experience with patients experiencing [illegible]	Wiens RN BScN, B.; Beaven, J.; Viste, D.; Ghosh, S.; Vanden Berg, S.	2023	Canadian Journal of Emergency Medicine	25	(Wiens RN BScN B.; Beaven J.; Viste D.; Ghosh [illegible]	S53	10.1007/s43678-023-00508-z	WiensRN BScN 2023	Irrelevant	Excluded by TI/AB review
3035	Application of the Modified Early Obstetrical Warning System (MEOWS) in postpartum patients in a Canadian emergency [illegible]	Kaur, J.; Thompson, C.; McLeod, S.; Varner, C.	2023	Canadian Journal of Emergency Medicine	25	(Kaur J.; Thompson C.; McLeod S.; Varner C.)	S54-S55	10.1007/s43678-023-00508-z	Kaur 2023	Irrelevant	Excluded by TI/AB review

S4 Table. All studies identified in the literature search

3036	A community-based participatory research study to evaluate an ED outreach worker program for people experiencing [illegible]	Snider, C.; Xiao, S.; Handford, C.; Pinto, A.	2023	Canadian Journal of Emergency Medicine	25	(Snider C.; Xiao S.; Handford C.; Pinto [illegible]	S59	10.1007/s43678-023-00508-z	Snider 2023	Irrelevant	Excluded by TI/AB review

S4 Table. All studies identified in the literature search

3037	2021 ABA Annual Meeting Abstracts/Poster	NA	2023	Journal of Burn Care and Research	44	3	NA	NA	NA	Irrelevant	Excluded by TI/AB review

S4 Table. All studies identified in the literature search

3038	A177: Racial disparities in breast cancer patients with spinal metastases: An analysis of 1,072 patients over a decade	Mixon, A.; Huffman, W.; Hirase, T.; Marco, R.; Taraballi, F.; Saifi, C.	2023	Global Spine Journal	13	2	106S-107S	10.1177/21925682231166108	Mixon 2023	Irrelevant	Excluded by TI/AB review

S4 Table. All studies identified in the literature search

3039	P621: A retrospective analysis of concomitant spinal injuries in the setting of polytrauma at a tertiary hospital in Singapore	Qiu, T.; Yang, C.; Lim, S.; Ng, Z.X.	2023	Global Spine Journal	13	2	536S-537S	10.1177/21925682231166109	Qiu 2023	Irrelevant	Excluded by TI/AB review

S4 Table. All studies identified in the literature search

3040	P526: Cervical myelopathy surgical outcomes between two neighboring medical institutions: A comparative analysis	Kantorek, N.; Bagley, C.	2023	Global Spine Journal	13	2	489S-490S	10.1177/21925682231166109	Kantorek 2023	Irrelevant	Excluded by TI/AB review

S4 Table. All studies identified in the literature search

3041	P310: Portal utilization in surgical spine patients: Sociodemographic factors and social determinants of health	Owolo, E.; Petitt, Z.; Charles, A.; Baeta, C.; Poehlein, E.; Green, C.; Cook, C.; Sperber, J.; Erickson, M.; Goodwin, C.R.	2023	Global Spine Journal	13	2	379S	10.1177/21925682231166109	Owolo 2023	Irrelevant	Excluded by TI/AB review
3042	Virtual Triage from Freestanding Emergency Departments: A Propensity-Score Weighted Analysis of Short-Term [illegible]	Lorenz, W.; Baimas-George, M.; Yang, H.; Paton, B.L.; Barbat, S.; Matthews, B.D.; Reinke, C.E.; Schiffern, L.	2023	Surgical Endoscopy	37	(Lorenz W.; Baimas-George M.; Yang [illegible]	S231	10.1007/s00464-023-10071-4	Lorenz 2023	Irrelevant	Excluded by TI/AB review

S4 Table. All studies identified in the literature search

3043	Building an understanding of Ethnic minority people's Service Use Relating to Emergency care for injuries: the BE SURE study [illegible]	Baghdadi, F.; Evans, B.A.; Goodacre, S.; John, P.A.; Hettiarachchi, T.; John, A.; Lyons, R.A.; Porter, A.; Safari, S.; Siriwardena, A.N.; [illegible]	2023	BMJ Open	13	4	NA	10.1136/bmjopen-2022-069596	Baghdadi 2023	Irrelevant	Excluded by TI/AB review
3044	Caregiver Perspectives on Including Children in Alternative Emergency Medical Services [illegible]	Ward, C.E.; Singletary, J.; Campanella, V.; Page, C.; Simpson, J.N.	2023	Prehospital emergency care	NA	(Ward C.E.; Campanella V.; Page [illegible]	17-Jan	10.1080/10903127.2023.2206480	Ward 2023	Irrelevant	Excluded by TI/AB review
3045	The impact of COVID-19 on psychiatric clinical encounters among low-income racially-diverse children	Rusk, S.A.; DiBari, J.; Mason, D.M.; Li, M.; Hong, X.; Wang, G.; Pearson, C.; Mirolli, G.; Cheng, T.L.; Kogan, M.D.; Zuckerman, B.; [illegible]	2023	Journal of child psychology and psychiatry, and allied disciplines	NA	(Rusk S.A.; Li M.; Hong X.; Wang G.; [illegible]	NA	10.1111/jcpp.13809	Rusk 2023	Irrelevant	Excluded by TI/AB review
3046	Racial and Ethnic Disparities in Emergency Department Wait Times for Headache	Pierre Louis, K.M.; Harman, J.S.	2023	Journal of racial and ethnic health disparities	NA	(Pierre Louis K.M.) Florida State University [illegible]	NA	10.1007/s40615-023-01580-y	PierreLouis 2023	Irrelevant	Excluded by TI/AB review

S4 Table. All studies identified in the literature search

3047	Trauma clinic follow-up: Predictors of nonattendance and patient-reported reasons for no show	Prescher, H.; Haravu, P.; Silva, A.K.; Reid, R.R.; Wang, F.	2023	Trauma (United Kingdom)	25	2	137-149	10.1177/14604086211052795	Prescher 2023	Irrelevant	Excluded by TI/AB review

S4 Table. All studies identified in the literature search

3048	Telehealth follow-up after cholecystectomy is safe in veterans	Abbitt, D.; Choy, K.; Castle, R.; Carmichael, H.; Jones, T.S.; Wikiel, K.J.; Barnett, C.C.; Moore, J.T.; Robinson, T.N.; Jones, E.L.	2023	Surgical Endoscopy	37	4	3201-3207	10.1007/s00464-022-09501-6	Abbitt 2023	Irrelevant	Excluded by TI/AB review

S4 Table. All studies identified in the literature search

3049	Accuracy and clinical use of biliary point-of-care ultrasound: A retrospective cohort study	Archer, J.; Beck, S.	2023	EMA - Emergency Medicine Australasia	35	2	218-224	10.1111/1742-6723.14099	Archer 2023	Irrelevant	Excluded by TI/AB review
3050	HIV Pre-Exposure Prophylaxis in the Emergency Department: A Systematic Review	Gormley, M.A.; Nagy, T.R.; Moschella, P.; Lu, Z.; Rodriguez, J.; Roth, P.	2023	Annals of Emergency Medicine	81	4	468-481	10.1016/j.annemergmed.2022.07.015	Gormley 2023	Irrelevant	Excluded by TI/AB review
3053	Toward Improving Patient Equity in a Pediatric Emergency Department: A Framework for Implementation	Hartford, E.A.; Thomas, A.A.; Kerwin, O.; Usoro, E.; Yoshida, H.; Burns, B.; Rutman, L.E.; Migita, R.; Bradford, M.; Akhter, S.	2023	Annals of Emergency Medicine	81	4	385-392	10.1016/j.annemergmed.2022.11.015	Hartford 2023	Irrelevant	Excluded by TI/AB review

S4 Table. All studies identified in the literature search

3054	A Mixed-Methods Assessment of Coronavirus Disease of 2019‚ÄìEra Telehealth Acute Care Visits in the [illegible]	Sprecher, E.; Conroy, K.; Krupa, J.; Shah, S.; Chi, G.W.; Graham, D.; Starmer, A.J.	2023	Journal of Pediatrics	255	(Sprecher E., eli.sprecher@childrens.harvar[illegible]	121-127.e2	10.1016/j.jpeds.2022.10.036	Sprecher 2023	Irrelevant	Excluded by TI/AB review
3055	California's early transition to electric vehicles: Observed health and air quality co-benefits	Garcia, E.; Johnston, J.; McConnell, R.; Palinkas, L.; Eckel, S.P.	2023	Science of the Total Environment	867	(Garcia E., garc991@usc.edu; Johnston J.; [illegible]	NA	10.1016/j.scitotenv.2023.161761	Garcia 2023	Irrelevant	Excluded by TI/AB review
3056	Patient, family member, and ambulance staff experiences of prehospital acute pain management in adults: A systematic review and meta-synthesis	Whitley, G.A.; Wijegoonewardene, N.; Nelson, D.; Curtis, F.; Ortega, M.; Siriwardena, A.N.	2023	JACEP Open	4	2	NA	10.1002/emp2.12940	Whitley 2023	Irrelevant	Excluded by TI/AB review

S4 Table. All studies identified in the literature search

3057	Engagement, Advance Care Planning, and Hospice Use in a Telephonic Nurse-Led Palliative Care Program for Persons Living [illegible]	Liddicoat Yamarik, R.; Chiu, L.A.; Flannery, M.; Van Allen, K.; Adeyemi, O.; Cuthel, A.M.; Brody, A.A.; Goldfeld, K.S.; Schrag, D.; Grudzen, [illegible]	2023	Cancers	15	8	NA	10.3390/cancers15082310	Liddicoat Yamarik 2023	Irrelevant	Excluded by TI/AB review
3058	Beyond Binge Eating: The Impact of Implicit Biases in Healthcare on Youth with Disordered Eating and Obesity	Roberts, K.J.; Chaves, E.	2023	Nutrients	15	8	NA	10.3390/nu15081861	Roberts 2023	Irrelevant	Excluded by TI/AB review

S4 Table. All studies identified in the literature search

3059	Racial and Ethnic Differences in Emergency Department Wait Times for Patients with Substance Use Disorder	Goldfarb, S.S.; Graves, K.; Geletko, K.; Hansen, M.D.; Kinsell, H.; Harman, J.	2023	Journal of Emergency Medicine	64	4	481-487	10.1016/j.jemermed.2023.02.015	Goldfarb 2023	Irrelevant	Excluded by TI/AB review

S4 Table. All studies identified in the literature search

3060	Early vs late filling of new heart failure prescription and its association with avoidable hospital admissions	Poonawalla, I.B.; Sutherland, A.; Chung, L.; Dixon, S.W.; Ellis, J.J.	2023	Journal of Managed Care and Specialty Pharmacy	29	4	350-356	10.18553/jmcp.2023.29.4.350	Poonawalla 2023	Irrelevant	Excluded by TI/AB review

S4 Table. All studies identified in the literature search

3061	Musculoskeletal Literacy in Orthopedics: Associated Factors, Effects on Patient-Physician Communication and Intervention Strategies	Gruson, K.I.; Tabeayo, E.; Stallone, S.; Bedeir, Y.H.; Carlos Rodriguez-Merchan, E.	2023	Archives of Bone and Joint Surgery	11	4	227-235	10.22038/ABJS.2022.68429.3237	Gruson 2023	Irrelevant	Excluded by TI/AB review

S4 Table. All studies identified in the literature search

3062	Clinico-Epidemiological Study of Snake Bite Presenting to the Emergency Department of a Tertiary Care Hospital	Arjun, H.M.; Shinde, V.; Bhattaram, S.; Shaikh, Z.	2023	Toxicology International	30	2	187-192	10.18311/ti/2023/v30i2/32375	Arjun 2023	Irrelevant	Excluded by TI/AB review

S4 Table. All studies identified in the literature search

3064	Addressing Healthcare Disparities in Patients Undergoing Cervical Spine Surgery	Gonzalez, G.A.; Mitchell, D.; Wang, V.; Leibold, A.T.; Harrop, J.S.; Schmidt, R.	2023	Clinical Neurosurgery	69	(Gonzalez G.A.; Mitchell D.; Wang V.; Leibold A.T.; Harrop J.S.; Schmidt R.)	102-103	NA	Gonzalez 2023	Irrelevant	Excluded by TI/AB review
3066	LONGER SURGICAL WAIT TIMES AFTER VISIT TO UROLOGY CLINIC IS ASSOCIATED [illegible]	Srirangapatanam, S.; Hicks, C.; Chi, T.; Stoller, M.; Bayne, D.	2023	Journal of Urology	209	(Srirangapatanam S.) Orlando, FL, [illegible]	e1086	10.1097/JU.0000000000003349.16	Srirangapatanam 2023	Irrelevant	Excluded by TI/AB review

S4 Table. All studies identified in the literature search

3067	QUALITY METRICS FOR PATIENTS REQUIRING URGENT SURGICAL INTERVENTION - COMPARING A FREESTANDING EMERGENCY DEPARTMENT TO A HOSPITAL-BASED EMERGENCY DEPARTMENT	Jeng, G.; Meyer, A.; Hass, A.; Cruzen, E.; Duroseau, Y.; Richstone, L.	2023	Journal of Urology	209	(Jeng G.; Hass A.) Hempstead, NY, United States (Meyer A.; Cruzen E.; Duroseau Y.; Richstone L.) New York,	e58-e59	10.1097/JU.0000000000003217.14	Jeng 2023	Irrelevant	Excluded by TI/AB review

S4 Table. All studies identified in the literature search

3068	RACIAL, ETHNIC, AND SEXUAL ORIENTATION REPRESENTATION ON ERECTILE DYSFUNCTION ACADEMIC WEBSITES	Channa, L.; Pinto, K.; Staff, I.; McLaughlin, T.; Bieniek, J.	2023	Journal of Urology	209	(Channa L.) Farmington, CT, United States (Pinto K.; Staff I.; McLaughlin T.; Bieniek J.) Hartford, CT, United States	e138	10.1097/JU.0000000000003227.12	Channa 2023	Irrelevant	Excluded by TI/AB review

S4 Table. All studies identified in the literature search

3069	THE ASSOCIATION BETWEEN FRAILTY, LOW TESTOSTERONE, AND POSTOPERATIVE OUTCOMES AMONG MEN UNDERGOING TRANSURETHRAL RESECTION OF BLADDER TUMOR	Greenberg, D.R.; Lin, J.S.; Kumar, S.K.S.R.; Mi, X.; Schaeffer, E.M.; Meeks, J.M.; Brannigan, R.E.; Halpern, J.A.	2023	Journal of Urology	209	(Greenberg D.R.; Lin J.S.; Kumar S.K.S.R.; Mi X.; Schaeffer E.M.; Meeks J.M.; Brannigan R.E.; Halpern J.A.) Chicago, [illegible]	e295	10.1097/JU.0000000000003247.01	Greenberg 2023	Irrelevant	Excluded by TI/AB review

S4 Table. All studies identified in the literature search

3070	Opportunities to improve opt-out bloodborne virus screening in two large London emergency departments	Fairhead, C.; Durkin, R.; Hart, J.; Burns, F.; Pinto, J.; Hunter, A.; Macdonald, D.; Barber, T.J.	2023	HIV Medicine	24	(Fairhead C.; Durkin R.; Hart J.; Burns F.; Pinto J.; Hunter A.; Macdonald D.; Barber T.J.) Royal Free London NHS Foundation Trust	17-18	10.1111/hiv.13477	Fairhead 2023	Irrelevant	Excluded by TI/AB review

S4 Table. All studies identified in the literature search

3071	Hyperammonemia and First- Degree Atrio-Ventricular Block in Adult Male from Valproic Acid Toxicity	Wilson, N.	2023	CNS Spectrums	28	2	253-254	NA	Wilson 2023	Irrelevant	Excluded by TI/AB review

S4 Table. All studies identified in the literature search

3072	Value-based Acute Care at Home: Sustainability, Savings, and Reduction in Emergency Department and Inpatient Service Utilization	Andrews, C.; Stuck, A.; Crowley, C.	2023	Journal of the American Geriatrics Society	71	(Andrews C.; Stuck A.; Crowley C.) Clinical,West Health Institute, San Diego, CA, United States	S72	10.1111/jgs.18336	Andrews 2023	Irrelevant	Excluded by TI/AB review

S4 Table. All studies identified in the literature search

3073	Impact of a Community Paramedic Program on Older Adults 30-Day Emergency Department Revisit	Simpson, M.; Pagel, P.; Sergi, C.; Liao, V.	2023	Journal of the American Geriatrics Society	71	(Simpson M.; Sergi C.; Liao V.) Advo [illegible]	S77	10.1111/jgs.18336	Simpson 2023	Irrelevant	Excluded by TI/AB review
3074	Reducing Behavioral and Psychological Symptoms of Dementia for Acutely-Ill Persons with Dementia via Patient [illegible]	Sinvani, L.; Ardito, S.; Strunk, A.; Gromova, V.; Liu, Y.; Schantz, E.; Arroon, A.; Ilyas, A.; Levin, J.; Makhnevich, A.; D'Angelo, S.; Boltz, M.	2023	Journal of the American Geriatrics Society	71	(Sinvani L.; Ardito S.; Strunk A.; Gromova V.; [illegible]	S10	10.1111/jgs.18336	Sinvani 2023	Irrelevant	Excluded by TI/AB review

S4 Table. All studies identified in the literature search

3075	Identifying and reducing racial disparities in pain treatment with geriatric emergency department programming	Durham, T.; Runels, T.; Gruber, E.; Haskins, K.; Vaughan, C.P.; Hwang, U.	2023	Journal of the American Geriatrics Society	71	(Durham T.; Hwang U.) GRECC, James J Peters VA Medical Center, New York, NY, United States (Runels T.) VA Pain Research Infor[illegible]	S151-S152	10.1111/jgs.18336	Durham 2023	Irrelevant	Excluded by TI/AB review
3076	Correlates of Confidence that Physicians Know their Patients' End-of-Life (EOL)Preferences: Does My Doctor [illegible]	McDonald, E.; Gangavati, A.; Johnson, K.; Platt, A.; Olsen, M.; Williams, S.; Rhodes, R.	2023	Journal of the American Geriatrics Society	71	(McDonald E.; Gangavati A.; Williams S[illegible]	S281	10.1111/jgs.18336	McDonald 2023	Irrelevant	Excluded by TI/AB review

S4 Table. All studies identified in the literature search

3077	Characteristics of Older Adults with Positive Neurocognitive Screens in the Geriatric ED	Sonnleitner, M.; Tolia, V.M.; Castillo, E.M.	2023	Journal of the American Geriatrics Society	71	(Sonnleitner M.) University of Hawai'i Syste[illegible]	S318-S319	10.1111/jgs.18336	Sonnleitner 2023	Irrelevant	Excluded by TI/AB review
3078	Relationship Between Time of Hospital Arrival and Clinical and Fluid Biomarker Outcomes in Traumatic Brain Injury	Anandarajah, A.; Walter, A.; Gugger, J.; Lynch, C.; Schneider, A.; Sandsmark, D.; Diaz-Arrastia, R.	2023	Neurology	100	17	NA	10.1212/WNL.0000000000203423	Anandarajah 2023	Irrelevant	Excluded by TI/AB review

S4 Table. All studies identified in the literature search

3079	Demographic factors associated with unplanned healthcare resource utilization (HRU) among people with epilepsy (PWE)	Urban, A.; Kirkpatrick, L.; Rajasekaran, V.; Fong, J.; Bagic, A.; Thirumala, P.	2023	Neurology	100	17	NA	10.1212/WNL.0000000000203959	Urban 2023	Irrelevant	Excluded by TI/AB review
3081	Assessing the Impact of COVID-19 on IBD Outcomes Among Vulnerable Patient Populations in a Large Metropolitan Center	Odufalu, F.-D.; Sewell, J.L.; Rudrapatna, V.; Somsouk, M.; Mahadevan, U.	2023	Inflammatory bowel diseases	NA	(Odufalu F.-D.) Division of Gastroenterology and Liver Diseases, Depar	NA	10.1093/ibd/izad041	Odufalu 2023	Irrelevant	Excluded by TI/AB review
3083	Patient and Hospital Characteristics Associated with Delayed Diagnosis of Appendicitis	Reyes, A.M.; Royan, R.; Feinglass, J.; Thomas, A.C.; Stey, A.M.	2023	JAMA Surgery	158	3	E227055	10.1001/jamasurg.2022.7055	Reyes 2023	Irrelevant	Excluded by TI/AB review

S4 Table. All studies identified in the literature search

3084	HEALTHCARE UTILIZATION FOR US VETERANS WITH HEART FAILURE [illegible]	Brownell, N.K.; Shannon, E.; Doan, Q.; Fonarow, G.C.; Ziaeian, B.	2023	Journal of the American College of Cardiology	81	8	536	10.1016/S0735-1097(23)00980-4	Brownell 2023	Irrelevant	Excluded by TI/AB review
3085	ACCELERATED PROTOCOL FOR MYOCARDIAL INFARCTION (MI) RULE-OUT WITHIN 1-HOUR OF PRESENTATION REDUCES [illegible]	McCord, J.K.; Cook, B.; Fadel, R.; Gandolfo, C.; Parikh, S.; Klausner, H.; Abdul-Nour, K.; Lewandowski, A.; Hudson, M.P.; Perrotta, G.S.; Zweig, B.; Gunaga, S.; Lanfear, D.E.; [illegible]	2023	Journal of the American College of Cardiology	81	8	1125	10.1016/S0735-1097(23)01569-3	McCord 2023	Irrelevant	Excluded by TI/AB review

S4 Table. All studies identified in the literature search

3086	ASSOCIATION BETWEEN FOOD DESERTS AND 30-DAY READMISSION FOR HEART FAILURE: A SINGLE CENTER STUDY	Alebna, P.L.; Gyekye, K.; Guo, X.; Nkeangu, F.; Gharbin, J.; Alebna, P.A.; Montgomery, M.	2023	Journal of the American College of Cardiology	81	8	1975	10.1016/S0735-1097(23)02419-1	Alebna 2023	Irrelevant	Excluded by TI/AB review

S4 Table. All studies identified in the literature search

3087	Association Between Hospital Adoption of an Emergency Department Treatment Pathway for Opioid Use Disorder and Patient Initiation of Buprenorphine After Discharge	Solomon, K.T.; O'Connor, J.; Gibbons, J.B.; Kilaru, A.S.; Feder, K.A.; Xue, L.; Saloner, B.; Stuart, E.A.; Cole, E.S.; Hulsey, E.; Meisel, Z.; Patel, E.; Donohue, J.M.	2023	JAMA health forum	4	3	e230245	10.1001/jamahealthforum.2023.0245	Solomon 2023	Irrelevant	Excluded by TI/AB review

S4 Table. All studies identified in the literature search

3088	Geographic Variation in Acute Pediatric Mental Health Utilization	Winckler, B.; Nguyen, M.; Khare, M.; Patel, A.; Crandal, B.; Jenkins, W.; Fisher, E.; Rhee, K.E.	2023	Academic Pediatrics	23	2	448-456	10.1016/j.acap.2022.07.026	Winckler 2023	Irrelevant	Excluded by TI/AB review

S4 Table. All studies identified in the literature search

3089	End-of-life health care use among socially isolated and cognitively impaired older adults	Kotwal, A.A.; Cenzer, I.S.; Yaffe, K.; Perissinotto, C.; Smith, A.K.	2023	Journal of the American Geriatrics Society	71	3	880-887	10.1111/jgs.18131	Kotwal 2023	Irrelevant	Excluded by TI/AB review
3090	Racial and Ethnic Disparities in Emergency Department Wait Times for Children: Analysis [illegible]	Gorski, J.K.; Alpern, E.R.; Lorenz, D.J.; Ramgopal, S.	2023	Academic Pediatrics	23	2	381-386	10.1016/j.acap.2022.10.013	Gorski 2023	Irrelevant	Excluded by TI/AB review

S4 Table. All studies identified in the literature search

3091	Traumatic Injury in Pregnancy: A Propensity Score-Matched Analysis	Ali, A.; Simpson, J.T.; Tatum, D.; Sedhom, J.A.; Broome, J.; McGrew, P.R.; Duchesne, J.; Taghavi, S.	2023	Journal of Surgical Research	283	(Ali A.; Simpson J.T.; Tatum D.; Sedhom J.A.; Broo	1018-1025	10.1016/j.jss.2022.10.087	Ali 2023	Irrelevant	Excluded by TI/AB review
3092	Descriptive analysis of emergency medical services 72-hour repeat patient encounters in a single, Urban Agency	Supples, M.W.; Liao, M.; O'Donnell, D.P.; Duszynski, T.J.; Glober, N.K.	2023	American Journal of Emergency Medicine	65	(Supples M.W., mwsupple@wakehealth.edu) Department of Emergency	113-117	10.1016/j.ajem.2022.12.025	Supples 2023	Irrelevant	Excluded by TI/AB review
3093	A Conceptual Framework for Optimizing the Equity of Hospital-Based Emergency Care: The Structure of Hospital Transfer Networks	Hsuan, C.; Carr, B.G.; Vanness, D.; Wang, Y.; Leslie, D.L.; Dunham, E.; Rogowski, J.A.	2023	Milbank Quarterly	101	1	74-125	10.1111/1468-0009.12609	Hsuan 2023	Irrelevant	Excluded by TI/AB review

S4 Table. All studies identified in the literature search

3094	Impact of a trauma recovery center on emergency department utilization for victims of violence	Pyles, O.; Richards, R.; Galligher, A.; Du, J.; Brooks, J.; Southerland, L.T.	2023	American Journal of Emergency Medicine	65	(Pyles O.; Du J.; Brooks J.) The Ohio State [illegible]	125-129	10.1016/j.ajem.2022.12.024	Pyles 2023	Irrelevant	Excluded by TI/AB review
3095	Facing Healthcare Access Challenges With Specialty Care Clinics in Central Appalachia	Mullins, C.M.; Helton, R.; Owens-Tyson, T.; Hill-Collins, P.; Domby, S.N.	2023	Journal of Radiology Nursing	42	1	43-51	10.1016/j.jradnu.2022.09.006	Mullins 2023	Irrelevant	Excluded by TI/AB review

S4 Table. All studies identified in the literature search

3096	154. Treatment of Chlamydia and Gonorrhea in a Chicago Health System: Observations of Treatment Practices Before and After Updated CDC Guidelines	Hardy, T.; Bernstein, K.; Schwartz, A.	2023	Journal of Adolescent Health	72	3	S87	10.1016/j.jadohealth.2022.11.176	Hardy 2023	Irrelevant	Excluded by TI/AB review

S4 Table. All studies identified in the literature search

3097	70. Description of an Integrated Adolescent Mental Health Clinic for Inner-City Youth at a School-Based Health Center (SBHC) in Washington, DC: An Innovative Approach	Ezeana, M.; Aramburu, M.; Chanani, S.	2023	Journal of Adolescent Health	72	3	S42-S43	10.1016/j.jadohealth.2022.11.091	Ezeana 2023	Irrelevant	Excluded by TI/AB review

S4 Table. All studies identified in the literature search

3098	67. Evaluating Outcomes for Adolescent/Young Adult Patients with High-Risk PHQ-9 Scores in an Academic Adolescent/Young Adult Program	Edwards, J.G.; Vitkovskiy, P.; Pelletier, A.; Butler, N.G.; Jhe, G.; Tsappis, M.; Richmond, T.; Borus, J.	2023	Journal of Adolescent Health	72	3	S41	10.1016/j.jadohealth.2022.11.088	Edwards 2023	Irrelevant	Excluded by TI/AB review

S4 Table. All studies identified in the literature search

3099	68. Evaluation of Adolescent Patients‚Äô Trust in Their Physicians in a Pediatric Emergency Department	Efrem, E.; Badolato, G.; Lemke, M.; Goyal, M.	2023	Journal of Adolescent Health	72	3	S41-S42	10.1016/j.jadohealth.2022.11.089	Efrem 2023	Irrelevant	Excluded by TI/AB review

S4 Table. All studies identified in the literature search

3100	Engaging Seriously Ill and Vulnerable Older Adults in a Nurse-Led Telephonic Intervention: The Emergency Medicine Palliative [illegible]	Yamarik, R.L.; Flannery, M.; Van Allen, K.; Cuthel, A.; Curtis, J.; Chiu, L.; Brandon, I.; Goldfeld, K.; Grudzen, C.	2023	Journal of Pain and Symptom Management	65	3	e296	10.1016/j.jpainsymman.2022.12.115	Yamarik 2023	Irrelevant	Excluded by TI/AB review
3101	Biliary colic in the emergency department: A state-wide analysis of one-year costs and clinical outcomes	Makutonin, M.; Moghatederi, A.; Newton, S.; Ma, Y.; Meltzer, A.C.	2023	Surgery Open Science	12	(Makutonin M.; Newton S.; Meltzer A.C., ameltzer@mfa.gw [illegible]	13-Sep	10.1016/j.sopen.2023.02.002	Makutonin 2023	Irrelevant	Excluded by TI/AB review
3102	Pathways connecting socioeconomic marginalization and overdose: A qualitative narrative synthesis	van Draanen, J.; Jamula, R.; Karamouzian, M.; Mitra, S.; Richardson, L.	2023	International Journal of Drug Policy	113	(van Draanen J.; Jamula R.; Karamouzian M.; [illegible]	NA	10.1016/j.drugpo.2023.103971	vanDraanen 2023	Irrelevant	Excluded by TI/AB review

S4 Table. All studies identified in the literature search

3103	Predictors of Incident Heart Failure Diagnosis Setting: Insights From the Veterans Affairs Healthcare [illegible]	Tisdale, R.L.; Fan, J.; Calma, J.; Cyr, K.; Podchiyska, T.; Stafford, R.S.; Maron, D.J.; Hernandez-Boussard, [illegible]	2023	JACC: Heart Failure	11	3	347-358	10.1016/j.jchf.2022.11.013	Tisdale 2023	Irrelevant	Excluded by TI/AB review
3104	Utility of Wrist-Wearable Data for Assessing Pain, Sleep, and Anxiety Outcomes after Traumatic Stress Exposure	Straus, L.D.; An, X.; Ji, Y.; McLean, S.A.; Neylan, T.C.; Cakmak, A.S.; Richards, A.; Clifford, G.D.; Liu, M.; Zeng, D.; House, S.L.; Beaudoin, F.L.; [illegible]	2023	JAMA Psychiatry	80	3	220-229	10.1001/jamapsychiatry.2022.4533	Straus 2023	Irrelevant	Excluded by TI/AB review
3105	Associations of Preexisting Vascular Risk Factors With Outcomes After Traumatic Brain Injury: A TRACK-TBI Study	Schneider, A.L.C.; Barber, J.; Temkin, N.; Gardner, R.C.; Manley, G.; Diaz-Arrastia, R.; Sandsmark, D.	2023	Journal of Head Trauma Rehabilitation	38	2	E88-E98	10.1097/HTR.0000000000000798	Schneider 2023	Irrelevant	Excluded by TI/AB review

S4 Table. All studies identified in the literature search

3106	Investigating suicide related behaviours across sexual orientation and neighbourhood deprivation levels: A cohort study using linked health administrative data	Azra, K.K.; Nielsen, A.; Kim, C.; Dusing, G.J.; Chum, A.	2023	PLoS ONE	18	3-Mar	NA	10.1371/journal.pone.0282910	Azra 2023	Irrelevant	Excluded by TI/AB review
3107	Results of a Cluster Randomized Trial Implementing a Rapid High-Sensitivity Troponin I Protocol	Cook, B.; Gandolfo, C.; Klausner, H.A.; McCord, J.M.; Miller, J.B.; Mills, N.; Levy, P.D.; Krupp, S.S.	2023	Journal of Emergency Medicine	64	3	419-420	10.1016/j.jemermed.2023.03.017	Cook 2023	Irrelevant	Excluded by TI/AB review
3108	Patient Factors Associated with Death of COVID-19 Patients in an Urban Emergency Department	Tusa, A.F.; Jones, A.T.; Harris, D.; Janz, D.; Moreno, L.A.; Fondong, M.; DeBlieux, P.M.C.; Rhodes, S.	2023	Journal of Emergency Medicine	64	3	422-423	10.1016/j.jemermed.2023.03.021	Tusa 2023	Irrelevant	Excluded by TI/AB review

S4 Table. All studies identified in the literature search

3109	Clinical and biochemical predictors of intensive care unit admission among patients with [illegible]	Khan, A.A.; Ata, F.; Bashir, M.; Iqbal, P.; Kartha, A.	2023	World Journal of Diabetes	14	3	271-278	10.4239/wjd.v14.i3.271	Khan 2023	Irrelevant	Excluded by TI/AB review
3110	Adherence to Diabetes Medications and Health Care Use during the COVID-19 Pandemic among High-Risk Patients	Yoon, J.; Chen, C.; Chao, S.; Wong, E.; Rosland, A.-M.	2023	Journal of the American Board of Family Medicine	36	2	289-302	10.3122/jabfm.2022.220319R1	Yoon 2023	Irrelevant	Excluded by TI/AB review
3111	Interpersonal Primary Care Continuity for Chronic Conditions Is Associated with Fewer Hospitalizations and Emergency Department Visits	Gaglioti, A.H.; Li, C.; Baltrus, P.T.; She, Z.; Douglas, M.D.; Moore, M.A.; Rao, A.; Immergluck, L.C.; Ayer, T.; Bazemore, A.; Rust, G.; Mack, D.H.	2023	Journal of the American Board of Family Medicine	36	2	303-312	10.3122/jabfm.2022.220015R1	Gaglioti 2023	Irrelevant	Excluded by TI/AB review

S4 Table. All studies identified in the literature search

3113	Impact of COVID-19 Pandemic on Food Insecurity in an Urban Emergency Department Patient [illegible]	Enayati, D.; Chan, V.; Koenig, G.; Povey, K.; Nhoung, H.K.; Becker, L.R.; Saulters, K.J.; Breed, R.; Jarris, Y.; [illegible]	2023	Western Journal of Emergency Medicine	24	2	127-134	10.5811/westjem.2023.1.59007	Enayati 2023	Irrelevant	Excluded by TI/AB review
3115	Patient-Centered Outcomes of an Emergency Department Social	Gupta, R.; Wang, A.; Wang, D.; Ortiz, D.; Kurian, K.; Halmer, T.; Jaung, M.S.	2023	Western Journal of Emergency Medicine	24	2	193-200	10.5811/westjem.2022.10.57096	Gupta 2023	Irrelevant	Excluded by TI/AB review
3116	Mobile emergency department care to nursing home residents: a novel outreach service	Udesen, S.E.J.; Rasmussen, C.-H.; Mikkelsen, S√∏.; Andersen, N.; Brabrand, M.; Lassen, A.T.	2023	Age and Ageing	52	3	NA	10.1093/ageing/afad025	Udesen 2023	Irrelevant	Excluded by TI/AB review

S4 Table. All studies identified in the literature search

3117	Among-Hospital Variation in Intensive Care Unit Admission Practices and Associated Outcomes for Patients with Acute Respiratory Failure	Anesi, G.L.; Dress, E.; Chowdhury, M.; Wang, W.; Small, D.S.; Delgado, M.K.; Bayes, B.; Szymczak, J.E.; Glassman, L.W.; Barreda, F.X.; Weiner, J.Z.; Escobar, G.J.; Halpern, S.D.; Liu, V.X.	2023	Annals of the American Thoracic Society	20	3	406-413	10.1513/AnnalsATS.202205-429OC	Anesi 2023	Irrelevant	Excluded by TI/AB review

S4 Table. All studies identified in the literature search

3118	An outpatient pain plan and ED pain pathway for adults with sickle cell disease	Mercado, S.H.	2023	JAAPA : official journal of the American Academy of Physician Assistants	36	3	20-23	10.1097/01.JAA.0000920956.33631.26	Mercado 2023	Irrelevant	Excluded by TI/AB review

S4 Table. All studies identified in the literature search

3120	NUBE Abstract	NA	2023	Western Journal of Emergency Medicine	24	2.1	NA	NA	NA	Irrelevant	Excluded by TI/AB review

S4 Table. All studies identified in the literature search

3121	‚ÄúI am Still in Pain, but I Have Tools Now‚Äù: A Case Study on Multidisciplinary Pain and Anxiety Management for a Cancer Patient Living with Bone Metastases and	Tao, C.; Farfour, H.; Schneekloth, T.	2023	Psycho-Oncology	32	(Tao C.; Farfour H.; Schneekloth T.) Mayo Clinic Arizo	74	10.1002/pon.6090	Tao 2023	Irrelevant	Excluded by TI/AB review
3122	EFFECT OF LANGUAGE BARRIERS ON HOSPITAL READMISSIONS AND ED REVISITATIONS	Ho, I.; Kolar, G.; Barwise, A.; Dugani, C.; Brumbraugh, J.; Kleinman Sween, J.; Kiliaki, S.; Kirchoff, R.	2023	Journal of Hospital Medicine	18	(Ho I.; Kolar G.; Dugani C.; Brumbraugh J.; Kleinman Swee	S128-S129	10.1002/jhm.13090	Ho 2023	Irrelevant	Excluded by TI/AB review
3123	INTEGRATING COMMUNITY HEALTH WORKERS INTO INTERPROFESSIONAL HOSPITAL MEDICINE BASED TEAMS	Jaffe, R.; Yeager, A.; Bharel, S.; Hayden, G.; Ostroff, P.; Horowitz, R.	2023	Journal of Hospital Medicine	18	(Jaffe R.; Yeager A.; Bharel S.; Hayden G.; Ostroff P.) Sidne	S34-S35	10.1002/jhm.13090	Jaffe 2023	Irrelevant	Excluded by TI/AB review

S4 Table. All studies identified in the literature search

3124	DISPARITIES AND HESITANCY IN COVID HOSPITALIZATION REDUCING MONOCLONAL ANTIBODY TREATMENT-AT-A-FIELD-HOSPITAL	Gupta, I.; Siddiqui, Z.; Kantsiper, M.; Shaikh, Y.; Vaeth, M.J.; Foyez, M.; ElHabashy, M.; Purekal, S.	2023	Journal of Hospital Medicine	18	(Gupta I.) Johns Hopkins University, School of Medicine, United d	S96-S97	10.1002/jhm.13090	Gupta 2023	Irrelevant	Excluded by TI/AB review
3125	Can accurate demographic information about people who use prescription medications nonmedically be derived from Twitter?	Yang, Y.-C.; Al-Garadi, M.A.; Love, J.S.; Cooper, H.L.F.; Perrone, J.; Sarker, A.	2023	Proceedings of the National Academy of Sciences of the United States of America	120	8	NA	10.1073/pnas.2207391120	Yang 2023	Irrelevant	Excluded by TI/AB review
3126	Effect of COVID-19 Vaccine Messaging Platforms in Emergency Departments on [illegible]	Rodriguez, R.M.; Nichol, G.; Eucker, S.A.; Chang, A.M.; O'Laughlin, K.N.; Pauley, A.; Rising, K.L.; Eswaran, V.; [illegible]	2023	JAMA Internal Medicine	183	2	115-123	10.1001/jamainternmed.2022.5909	Rodriguez 2023	Irrelevant	Excluded by TI/AB review

S4 Table. All studies identified in the literature search

3127	A prospective observational study comparing outcomes before and after the introduction of an intubation protocol during the COVID-19 pandemic	Black, H.; Hall, T.; Hrymak, C.; Funk, D.; Siddiqui, F.; Sokal, J.; Satoudian, J.; Foster, K.; Kowalski, S.; Dufault, B.; Leeies, M.	2023	Canadian Journal of Emergency Medicine	25	2	123-133	10.1007/s43678-022-00422-w	Black 2023	Irrelevant	Excluded by TI/AB review

S4 Table. All studies identified in the literature search

3129	Interactive association of chronic illness and food insecurity with emergency department utilization among school-age children in the United States: A cross-sectional study	Ghani, F.; Wang, H.; Manning, S.E.; Sambamoorthi, U.	2023	Health Science Reports	6	2	NA	10.1002/hsr2.1123	Ghani 2023	Irrelevant	Excluded by TI/AB review

S4 Table. All studies identified in the literature search

3130	Postacute Care Readmission and Resource Utilization in Patients from Socioeconomically Distressed Communities after Total Joint Arthroplasty	Magnuson, J.A.; Griffin, S.A.; Venkat, N.; Gold, P.A.; Courtney, P.M.; Krueger, C.A.	2023	Clinical Orthopaedics and Related Research	481	2	202-210	10.1097/CORR.0000000000002185	Magnuson 2023	Irrelevant	Excluded by TI/AB review

S4 Table. All studies identified in the literature search

3131	It's a Hard Knock Life: How Kids With Mild Traumatic Brain Injuries Are Treated	Krishnan, K.; Su, A.; Sigman, G.; Joyce, C.; Rech, M.A.; Long, C.M.	2023	Pediatric Emergency Care	39	2	74-79	10.1097/PEC.0000000000002611	Krishnan 2023	Irrelevant	Excluded by TI/AB review

S4 Table. All studies identified in the literature search

3132	Emergency Department Utilization, Hospital Admissions, and Office-Based Physician Visits Among Under-Resourced African American and Latino Older Adults	Cobb, S.; Bazargan, M.; Assari, S.; Barkley, L.; Bazargan-Hejazi, S.	2023	Journal of racial and ethnic health disparities	10	1	205-218	10.1007/s40615-021-01211-4	Cobb 2023	Irrelevant	Excluded by TI/AB review

S4 Table. All studies identified in the literature search

3134	Biosimilar Infliximab Switching is Not Associated with Adverse Outcomes: A Real-World Effectiveness Analyses in a National U.S. Cohort of U.S. Military Veterans with Inflammatory Bowel Disease	Hou, J.K.; Pham, C.; Sansgiry, S.; Modi, V.; Waljee, A.K.	2023	Journal of Crohn's and Colitis	17	(Hou J.K.) Baylor College of Medicine 5207 Contour Place, Section of [illegible]	i880-i881	10.1093/ecco-jcc/jjac190.0880	Hou 2023	Irrelevant	Excluded by TI/AB review
3135	RANDOMIZATION SCHEMES USING ELECTRONIC HEALTH SYSTEMS TO FACILITATE [illegible]	Haukoos, J.; Toerper, M.; Houk, R.; Hopkins, E.; Galbraith, J.; Hsieh, Y.-H.; Lyons, M.; White, D.; Rowan, S.; Rothman, R.	2023	Clinical Trials	20	(Haukoos J.) Denver Health Medic[illegible]	42-43	10.1177/17407745221132063	Haukoos 2023	Irrelevant	Excluded by TI/AB review
3136	Prevalence of Undiagnosed Diabetes Identified by a Novel Electronic Medical Record Diabetes Screening Program in an [illegible]	Danielson, K.K.; Rydzon, B.; Nicosia, M.; Maheswaren, A.; Eisenberg, Y.; Lin, J.; Layden, B.T.	2023	JAMA Network Open	6	1	E2253275	10.1001/jamanetworkopen.2022.53275	Danielson 2023	Irrelevant	Excluded by TI/AB review

S4 Table. All studies identified in the literature search

3138	Environmental justice analysis of wildfire-related PM2.5 exposure using low-cost sensors in California	Kramer, A.L.; Liu, J.; Li, L.; Connolly, R.; Barbato, M.; Zhu, Y.	2023	Science of the Total Environment	856	(Kramer A.L.; Liu J.; Li L.; Connolly R.; [illegible]	NA	10.1016/j.scitotenv.2022.159218	Kramer 2023	Irrelevant	Excluded by TI/AB review
3139	Moderators and Mediators of a Digital Cognitive Behavior Therapy-Guided Self-Help Intervention for Eating Disorders: Informing Future [illegible]	Graham, A.K.; Fitzsimmons-Craft, E.E.; Sadeh-Sharvit, S.; Balantekin, K.N.; Eichen, D.M.; Firebaugh, M.-L.; Goel, N.J.; Monterubio, G.E.; [illegible]	2023	Journal of Consulting and Clinical Psychology	91	5	280-284	10.1037/ccp0000786	Graham 2023	Irrelevant	Excluded by TI/AB review
3142	Asthma, chronic obstructive pulmonary disease, and asthma-COPD overlap among US working adults	Syamlal, G.; Dodd, K.E.; Mazurek, J.M.	2023	Journal of Asthma	60	4	718-726	10.1080/02770903.2022.2089997	Syamlal 2023	Irrelevant	Excluded by TI/AB review

S4 Table. All studies identified in the literature search

3143	Predictors of outpatient follow-up care after adult emergency department asthma visits and association with 30-day outcomes	Abbott, E.E.; Vargas-Torres, C.; Karwoska Kligler, S.; Spadafore, S.; Lin, M.P.	2023	Journal of Asthma	60	5	938-945	10.1080/02770903.2022.2109166	Abbott 2023	Irrelevant	Excluded by TI/AB review
3144	Pulse Oximetry Screening: Association of State Mandates with Emergency Hospitalizations	Sakai-Bizmark, R.; Kumamaru, H.; Marr, E.H.; Bedel, L.E.M.; Mena, L.A.; Baghaee, A.; Nguyen, M.; Estevez, D.; Wu, F.; Chang, R.-K.R.	2023	Pediatric Cardiology	44	1	67-74	10.1007/s00246-022-03027-3	Sakai-Bizmark 2023	Irrelevant	Excluded by TI/AB review

S4 Table. All studies identified in the literature search

3145	African American Patients Experience Worse Outcomes than Hispanic Patients Following Bariatric Surgery: an Analysis Using the MBSAQIP Data Registry	Robinson, J.N.; Ayuso, S.A.; Thompson, K.J.; Kuwada, T.; Gersin, K.S.; Barbat, S.; Bauman, R.; McKillop, I.H.; Nimeri, A.	2023	Obesity Surgery	33	1	57-67	10.1007/s11695-022-06333-0	Robinson 2023	Irrelevant	Excluded by TI/AB review
3146	Demographic characteristics, outcomes and experience of patients using virtual urgent care services from 14 emergency department led sites in Ontario	McLeod, S.L.; Mondoux, S.; Hall, J.N.; Dainty, K.; McCarron, J.; Tarride, J.-E.; Abraham, L.; Tse, S.; Lim, R.; Fitzgibbon, M.; Montpellier, R.-A.; Rivlin, L.; Rodriguez, C.; Beck, L.; McLean, L.; Rosenfield, D.;	2023	Canadian Journal of Emergency Medicine	25	1	65-73	10.1007/s43678-022-00407-9	McLeod 2023	Irrelevant	Excluded by TI/AB review

S4 Table. All studies identified in the literature search

3147	Perspectives of COVID-19 vaccine‚Äìhesitant emergency department patients to inform messaging platforms to promote vaccine uptake	Gentsch, A.T.; Butler, J.; O'Laughlin, K.; Eucker, S.A.; Chang, A.; Duber, H.; Geyer, R.E.; Guth, A.; Kanzaria, H.K.; Pauley, A.; Rising, K.L.; Chavez, C.L.; Tupetz, A.; Rodriguez, R.M.	2023	Academic Emergency Medicine	30	1	32-39	10.1111/acem.14620	Gentsch 2023	Irrelevant	Excluded by TI/AB review

S4 Table. All studies identified in the literature search

3148	A Quality Framework to Address Racial and Ethnic Disparities in Emergency Department Care	Khidir, H.; Salhi, R.; Sabbatini, A.K.; Franks, N.M.; Green, A.; Richardson, L.D.; Terry, A.; Vasquez, N.; Goyal, P.; Kocher, K.; Venkatesh, A.K.; Lin, M.P.	2023	Annals of Emergency Medicine	81	1	47-56	10.1016/j.annemergmed.2022.08.010	Khidir 2023	Irrelevant	Excluded by TI/AB review
3149	Integration of the patient-centered medical home to deliver a care bundle for chronic obstructive pulmonary disease management	Portillo, E.C.; Lehmann, M.R.; Hagen, T.L.; Costner, M.G.; Kettner, J.T.; Bhardwaj, S.D.; McFarland, M.S.; Henderson, B.Y.; Bowersox, N.W.; Zhao, Q.; Chui, M.A.	2023	Journal of the American Pharmacists Association	63	1	212-219	10.1016/j.japh.2022.10.003	Portillo 2023	Irrelevant	Excluded by TI/AB review
3150	Palliative Medicine Referral and End-of-Life Interventions Among Racial and Ethnic Minority Patients With Advanced or Recurrent Gynecologic [illegible]	Tabuyo-Martin, A.; Torres-Morales, A.; Pitteloud, M.J.; Kshetry, A.; Oltmann, C.; Pearson, J.M.; Khawand, M.; Schlumbrecht, M.P.; Sanchez, J.C.	2023	Cancer Control	30	(Tabuyo-Martin A., adt66@miami.edu; Pearson [illegible]	NA	10.1177/10732748231157191	Tabuyo-Martin 2023	Irrelevant	Excluded by TI/AB review

S4 Table. All studies identified in the literature search

3151	Population-Level Provider Compliance with Provincial Treatment Guidelines for the Management of Gonorrhea in Adolescents and Adults in Alberta, Canada; 2000 to 2019	Ugarte-Torres, A.; Diaz Pallares, C.; Niruban, J.S.; Smyczek, P.; Gratrix, J.; Strong, D.; Singh, A.E.	2023	Sexually Transmitted Diseases	50	1	20-Nov	10.1097/OLQ.0000000000001721	Ugarte-Torres 2023	Irrelevant	Excluded by TI/AB review

S4 Table. All studies identified in the literature search

3152	Utilization of a Guideline-recommended Imaging Paradigm for Pregnant Patients with Suspicion of Pulmonary Embolism	McCandlish, J.A.; Naidich, J.J.; Feizullayeva, C.; Makhnevich, A.; Barish, M.A.; Sanelli, P.C.; Cohen, S.L.	2023	Journal of Thoracic Imaging	38	1	23-28	10.1097/RTI.0000000000000676	McCandlish 2023	Irrelevant	Excluded by TI/AB review

S4 Table. All studies identified in the literature search

3153	Factors Associated With a Discharge Against Medical Advice From an Emergency Department in Adult Patients With Appendicitis	Keneally, R.J.; Mazzeffi, M.A.; Estroff, J.M.; Yi, J.N.; Maman, S.R.; Heinz, E.R.; Chow, J.H.	2023	Journal of Emergency Medicine	64	1	40-46	10.1016/j.jemermed.2022.10.006	Keneally 2023	Irrelevant	Excluded by TI/AB review

S4 Table. All studies identified in the literature search

3154	A Machine Learning Approach to Support Urgent Stroke Triage Using Administrative Data and Social Determinants of Health at Hospital Presentation: Retrospective Study	Chen, M.; Tan, X.; Padman, R.	2023	Journal of Medical Internet Research	25	(Chen M.) Department of Information Systems & Business Analytics, College of Business, Florida International University, Miami [illegible]	NA	10.2196/36477	Chen 2023	Irrelevant	Excluded by TI/AB review

S4 Table. All studies identified in the literature search

3156	Disparities Associated With Electronic Behavioral Alerts for Safety and Violence Concerns in the Emergency Department	Haimovich, A.D.; Taylor, R.A.; Chang-Sing, E.; Brashear, T.; Cramer, L.D.; Lopez, K.; Wong, A.H.	2023	Annals of Emergency Medicine	NA	(Haimovich A.D.) Department of Emergency Medicine, Beth Israel Deaconess Medical Center, Boston, MA, United States (Taylo[illegible]	NA	10.1016/j.annemergmed.2023.04.004	Haimovich 2023	Irrelevant	Excluded by TI/AB review

S4 Table. All studies identified in the literature search

3159	Perceptions of Acute Care Telemedicine Among Caregivers for Persons Living with Dementia: A Qualitative Study	Chary, A.; Hernandez, N.; Rivera, A.P.; Ramont, V.; Obi, T.; Santangelo, I.; Ritchie, C.; Singh, H.; Hayden, E.; Naik, A.D.; Liu, S.; Kennedy, M.	2023	Journal of Applied Gerontology	NA	(Chary A., Anita.chary@bcm.edu) Department of Emergency Medicine, Baylor College of Medicine, Houston, TX, United States (Char	NA	10.1177/07334648231198018	Chary 2023	Irrelevant	Excluded by TI/AB review

S4 Table. All studies identified in the literature search

3160	An educational intervention in the emergency department seeking to improve COVID-19 vaccination rates among [illegible]	DeWaard, S.; Dewyer, Z.; Al Assil, T.; Gallap, R.; Patrick, L.; Fukuda, N.; Ford, M.	2023	Infection, Disease and Health	NA	(DeWaard S., Shelby.DeWaard@wmed.edu [illegible]	NA	10.1016/j.idh.2023.07.001	DeWaard 2023	Irrelevant	Excluded by TI/AB review

S4 Table. All studies identified in the literature search

3161	The acceptability, feasibility, and preliminary efficacy of a supported online self-help treatment program for binge-eating disorder	Rom, S.; Miskovic-Wheatley, J.; Barakat, S.; Aouad, P.; Kim, M.; Fuller-Tyszkiewicz, M.; Maguire, S.	2023	Frontiers in Psychiatry	14	(Rom S., sean.rom@sydney.edu.au; Fuller-Tyszkiewicz M.) Faculty of Health, School of Psychology, Deakin University, Geelong, [illegible]	NA	10.3389/fpsyt.2023.1229261	Rom 2023	Irrelevant	Excluded by TI/AB review

S4 Table. All studies identified in the literature search

3162	Association of High-Deductible Health Plans With Health Care Use and Costs for Patients With Cardiovascular Disease	Gupta, R.; Yang, L.; Lewey, J.; Navathe, A.S.; Groeneveld, P.W.; Khatana, S.A.M.	2023	Journal of the American Heart Association	12	19	NA	10.1161/JAHA.123.030730	Gupta 2023	Irrelevant	Excluded by TI/AB review

S4 Table. All studies identified in the literature search

3163	Intersecting gender identity and racial/ethnic inequities in eating disorder risk factors, symptoms, and diagnosis among U.S. college students: An intersectional multilevel analysis of individual heterogeneity and discriminatory accuracy	Gordon, A.R.; Beccia, A.L.; Egan, N.; Lipson, S.K.	2023	International Journal of Eating Disorders	NA	(Gordon A.R., allegrag@bu.edu) Department of Community Health Sciences, Boston University School of Public Health, Boston [illegible]	NA	10.1002/eat.24089	Gordon 2023	Irrelevant	Excluded by TI/AB review

S4 Table. All studies identified in the literature search

3164	What Patients Call Their Inhalers Is Associated with ‚ÄúAsthma Attacks‚Äù	Forth, V.E.; Cardet, J.C.; Chang, K.-L.; Ericson, B.; Hurley, L.P.; Maher, N.E.; Staton, E.W.; Sosa, B.T.; Israel, E.; Carroll, J.K.; Fagan, M.; Fuhlbrigge, A.L.; Hernandez, P.A.; Kruse, J.; Manning, B.K.; Pace, W.D.; Phipatanakul, W.; Shields, J.B.; Rodriguez-Louis, J.; Cui, J.; Salciccioli, J.D.; Mosnaim, G.; Omole, F.	2023	Journal of the American Board of Family Medicine	36	4	650-661	10.3122/JABFM.2022.220270R2	Forth 2023	Irrelevant	Excluded by TI/AB review

S4 Table. All studies identified in the literature search

3165	Natural Language Processing ,Äî A Surveillance Stepping Stone to Identify Child Abuse	Shum, M.; Hsiao, A.; Teng, W.; Asnes, A.; Amrhein, J.; Tiyyagura, G.	2023	Academic Pediatrics	NA	(Shum M., shumm@chop.edu; Hsiao A.; Asnes A.; Tiyyagura G.) Department of Pediatrics (M Shum, A Hsiao, A Asnes, and [illegible]	NA	10.1016/j.acap.2023.08.015	Shum 2023	Irrelevant	Excluded by TI/AB review

S4 Table. All studies identified in the literature search

3166	A Qualitative Study of Racial, Ethnic, and Cultural Experiences of Minority Clinicians During Agitation Care in the Emergency Department	Agboola, I.K.; Rosenberg, A.; Robinson, L.; Brashear, T.K.; Eixenberger, C.; Shah, D.; Pavlo, A.J.; Im, D.D.; Ray, J.M.; Coupet, E.; Wong, A.H.	2023	Annals of Emergency Medicine	NA	(Agboola I.K.) Department of Emergency Medicine [illegible]	NA	10.1016/j.annemergmed.2023.09.014	Agboola 2023	Irrelevant	Excluded by TI/AB review
3167	Emergency department use after outpatient thyroidectomy across three states	Visenio, M.R.; Reddy, S.; Sturgeon, C.; Elaraj, D.M.; Ritter, H.E.; McDow, A.D.; Merkow, R.P.; Bilimoria, K.Y.; Yang, A.D.	2023	Surgery (United States)	NA	(Visenio M.R.; Merkow R.P.; Bilimoria K.Y.; Yang A.D. [illegible]	NA	10.1016/j.surg.2023.09.027	Visenio 2023	Irrelevant	Excluded by TI/AB review

S4 Table. All studies identified in the literature search

3168	Associations Between Physical Activity Vital Sign in Patients and Health Care Utilization in a Health Care System, 2018-2020	Lin, C.Y.; Ball, T.J.; Gentile, N.L.; McDonald, V.F.; Humbert, A.T.	2023	Journal of physical activity & health	20	1	28-34	10.1123/jpah.2022-0266	Lin 2023	Irrelevant	Excluded by TI/AB review

S4 Table. All studies identified in the literature search

3169	THE IMPLEMENTATION OF A NOVEL DKA TREATMENT PROTOCOL IN A COMMUNITY HOSPITAL SETTING	Pouliot, J.; Jennison, M.; Wright, M.; Bullock, B.; Jerkins, T.	2023	Critical Care Medicine	51	1	93	10.1097/01.ccm.0000906608.16393.b5	Pouliot 2023	Irrelevant	Excluded by TI/AB review
3170	Improving time to antibiotics in patients undergoing emergency laparotomy	Hoade, L.; Coakley, M.; Duncan, T.; McKnight, G.; Jardine, N.; Churchill, S.	2023	Anaesthesia	78	(Hoade L.; Coakley M.; Duncan T.; McKnight G.; Jardine N.; Churchill S.)	62	10.1111/anae.15924	Hoade 2023	Irrelevant	Excluded by TI/AB review

S4 Table. All studies identified in the literature search

3171	Prevalence of COVID-19 among hematology/oncology patients and providers of a community-facing [illegible]	Khan, A.; Kareff, S.A.; Barreto-Coelho, P.; Iyer, S.; Pico, B.; Stanchina, M.; Dutcher, G.; De Oliveira Novaes, [illegible]	2023	Cancer Epidemiology Biomarkers and Prevention	32	1	NA	10.1158/1538-7755.DISP22-B123	Khan 2023	Irrelevant	Excluded by TI/AB review
3172	Inequities in nancial burden, quality of life, and physical functioning among cancer survivors: A nationally-representative Medical Expenditures Panel Survey (MEPS) study	Mobley, E.M.; Garvan, G.; Norez, D.; Mitchell, H.; Salloum, R.; Gutter, M.S.; Awad, Z.T.; Braithwaite, D.; Anton, S.; Gurka, M.; Parker, A.S.	2023	Cancer Epidemiology Biomarkers and Prevention	32	1	NA	10.1158/1538-7755.DISP22-A051	Mobley 2023	Irrelevant	Excluded by TI/AB review
3173	GAPS IN CARE FOLLOWING FIRST TIME SEIZURE: A RETROSPECTIVE STUDY	Jenkins, C.I.; Cabrera, A.J.; Goldenholz, D.M.; Estes, M.; Casassa, C.M.	2023	Journal of Investigative Medicine	71	1	179	10.1177/108155892211 42328	Jenkins 2023	Irrelevant	Excluded by TI/AB review
3174	Health Care Utilization among Homeless-Experienced Adults Who Were [illegible]	Fine, D.R.; Joyce, A.; Chang, Y.; Lewis, E.; Weinstock, K.; Wright, J.; Gaeta, J.; [illegible]	2023	Substance use & misuse	58	9	1115-1120	10.1080/10826084.2023.2212279	Fine 2023	Irrelevant	Excluded by TI/AB review

S4 Table. All studies identified in the literature search

3175	Expansion of the Veterans Health Administration Network and Surgical Outcomes	Graham, L.A.; Schoemaker, L.; Rose, L.; Morris, A.M.; Aouad, M.; Wagner, T.H.	2022	JAMA Surgery	157	12	1115-1123	10.1001/jamasurg.2022.4978	Graham 2022	Irrelevant	Excluded by TI/AB review

S4 Table. All studies identified in the literature search

3176	Paediatric frequent use of emergency medical services: a systematic review	Scott, J.; Khanom, A.; Straw, J.; Strickland, A.; Porter, A.; Snooks, H.	2022	Emergency Medicine Journal	40	1	20-27	10.1136/emermed-2021-211701	Scott 2022	Irrelevant	Excluded by TI/AB review

S4 Table. All studies identified in the literature search

3177	Association between Individual Primary Care Physician Merit-based Incentive Payment System Score and Measures of Process and [illegible]	Bond, A.M.; Schpero, W.L.; Casalino, L.P.; Zhang, M.; Khullar, D.	2022	JAMA	328	21	2136-2146	10.1001/jama.2022.20619	Bond 2022	Irrelevant	Excluded by TI/AB review
3178	Barriers to recruitment into emergency department-initiated palliative care: a sub-study of a multi-site, randomized controlled trial	Brickey, J.; Flannery, M.; Cuthel, A.; Cho, J.; Grudzen, C.R.; Blaum, C.; Southerland, L.; Bischof, J.; Ouchi, K.; Elie, M.-C.; Swor, R.; Jubanyik, K.; Goldfeld, K.S.; Cohen, S.E.; Kim, [illegible]	2022	BMC Palliative Care	21	1	NA	10.1186/s12904-021-00899-9	Brickey 2022	Irrelevant	Excluded by TI/AB review
3182	Identifying the vulnerable regions of emergency medical services based on the three-stage of accessibility: a case study in Xi,Äôan, China	Xu, N.; Bai, J.; Yan, R.	2022	International Journal for Equity in Health	21	1	NA	10.1186/s12939-022-01653-0	Xu 2022	Irrelevant	Excluded by TI/AB review

S4 Table. All studies identified in the literature search

3183	Impact of insurance type in postoperative emergency department utilization and clinical outcomes following ventral [illegible]	Renshaw, S.; Kenawy, D.; Azap, R.; Gupta, A.; Poulose, B.; Collins, C.	2022	Surgical Endoscopy	36	12	9416-9423	10.1007/s00464-022-09287-7	Renshaw 2022	Irrelevant	Excluded by TI/AB review
3184	Road traffic density and recurrent asthma emergency department visits among Medicaid enrollees in New [illegible]	Insaf, T.Z.; Adeyeye, T.; Adler, C.; Wagner, V.; Proj, A.; McCauley, S.; Matson, J.	2022	Environmental Health: A Global Access Science Source	21	1	NA	10.1186/s12940-022-00885-5	Insaf 2022	Irrelevant	Excluded by TI/AB review
3185	Quality of end-of-life care in multiple myeloma: A 13-year analysis of a population-based cohort in Ontario, Canada	Mohyuddin, G.R.; Sinnarajah, A.; Gayowsky, A.; Chan, K.K.W.; Seow, H.; Mian, H.	2022	British Journal of Haematology	199	5	688-695	10.1111/bjh.18401	Mohyuddin 2022	Irrelevant	Excluded by TI/AB review
3186	Collection of patient race, ethnicity, and language data in emergency departments: a national survey	Velmurugiah, N.; Gill, J.; Chau, B.; Rahavi, A.; Shen, C.; Morakis, H.; Brubacher, J.R.	2022	Canadian Journal of Emergency Medicine	24	8	832-836	10.1007/s43678-022-00388-9	Velmurugiah 2022	Irrelevant	Excluded by TI/AB review

S4 Table. All studies identified in the literature search

3187	A transition of care model from hospital to community for Hispanic/Latino adult patients with diabetes: design and rationale for a [illegible]	Corsino, L.; Padilla, B.I.	2022	Pilot and Feasibility Studies	8	1	NA	10.1186/s40814-022-01203-z	Corsino 2022	Irrelevant	Excluded by TI/AB review

S4 Table. All studies identified in the literature search

3189	Outcomes in ED patients with non-specific ECG findings and low high-sensitivity troponin	Alshaikh, L.M.; Apple, F.S.; Christenson, R.H.; deFilippi, C.R.; Limkakeng, A.T.; McCord, J.; Nowak, R.M.; Singer, A.J.; Peacock, W.F.	2022	JACEP Open	3	6	NA	10.1002/emp2.12844	Alshaikh 2022	Irrelevant	Excluded by TI/AB review

S4 Table. All studies identified in the literature search

3190	Protocol for a randomised placebo-controlled trial investigating the efficacy and safety of a vitamin-mineral formula targeting dysregulated [illegible]	Rucklidge, J.J.; Blampied, F.M.; Manna, L.; Sherwin, A.; Bagshaw, S.; Mulder, R.T.; Boden, J.	2022	Contemporary Clinical Trials Communications	30	(Rucklidge J.J., julia.rucklidge@canterbury.ac.nz; [illegible]	NA	10.1016/j.conctc.2022.101027	Rucklidge 2022	Irrelevant	Excluded by TI/AB review
3191	Emergency department returns and early follow-up visits after heart failure hospitalization: Cohort study [illegible]	Solnick, R.E.; Vijayasiri, G.; Li, Y.; Kocher, K.E.; Jenq, G.; Bozaan, D.	2022	PLoS ONE	17	#####	NA	10.1371/journal.pone.0279394	Solnick 2022	Irrelevant	Excluded by TI/AB review
3192	Rapid Response Team Activations in Oncologic Ambulatory Sites: Characteristics, Interventions, and Outcomes	Majeed, J.; Chawla, S.; Bondar, E.; Chimonas, S.; Martin, S.C.; O'Sullivan, M.; Jones, D.	2022	JCO Oncology Practice	18	12	E1961-E1970	10.1200/OP.22.00436	Majeed 2022	Irrelevant	Excluded by TI/AB review

S4 Table. All studies identified in the literature search

3193	Healthcare Utilization for Medicaid-Insured Children with Medical Complexity: Differences by Sociodemographic Characteristics	Ming, D.Y.; Jones, K.A.; White, M.J.; Pritchard, J.E.; Hammill, B.G.; Bush, C.; Jackson, G.L.; Raman, S.R.	2022	Maternal and child health journal	26	12	2407-2418	10.1007/s10995-022-03543-x	Ming 2022	Irrelevant	Excluded by TI/AB review

S4 Table. All studies identified in the literature search

3194	Tonic and Phasic Neurophysiological Relationships With Adverse Posttraumatic Outcomes Vary by Racial/Ethnic Group	Harnett, N.; Fani, N.; Carter, S.; Sanchez, L.; Jovanovic, T.; Rowland, G.; LeBois, L.; Ely, T.; Van Rooij, S.; Seligowski, A.; Bruce, S.; House, S.; McLean, S.; Stevens, J.; Ressler, K.	2022	Neuropsychopharmacology	47	(Harnett N.; Fani N.; Carter S.; Sanchez L.; Jovanovic T.; Rowland G.; LeBois L.; Ely T.; Van Rooij S.; Seligowski A.; Bruce S.; House [illegible]	504-505	10.1038/s41386-022-01486-z	Harnett 2022	Irrelevant	Excluded by TI/AB review

S4 Table. All studies identified in the literature search

3195	THE SHARK-FIN THAT EMERGES AND IMMERGES: A CASE REPORT OF TRANSIENT AND RIPETITIVE TRIANGULAR QRS-ST-T	Valcher, S.; Alessandro, V.; Letizia, B.; Martina, B.; Elena, C.; Mauro, C.; Condorelli, G.	2022	European Heart Journal, Supplement	24	(Valcher S.; Alessandro V.; Letizia B.; Martina B.; Elena C.; Mauro C.; Condorelli G.) IRCCS Humanitas Research Hospital, Italy	K197	10.1093/eurheartjsupp/suac121.555	Valcher 2022	Irrelevant	Excluded by TI/AB review

S4 Table. All studies identified in the literature search

3196	Increased Early Syphilis Detection and Treatment in an Urban Emergency Department During the COVID-19 Pandemic	Lipps, A.; Malvestutto, C.; Koletar, S.L.; Dick, M.; Lindsey, S.E.; Bazan, J.A.	2022	Open Forum Infectious Diseases	9	(Lipps A.; Malvestutto C.) Ohio State University, Wexner Medical Center, Columbus, OH, United States (Koletar S.L.; Lindsey [illegible]	S902	10.1093/ofid/ofac492.1832	Lipps 2022	Irrelevant	Excluded by TI/AB review

S4 Table. All studies identified in the literature search

3197	The clinical and economic impact of a community-based, hybrid model of in-person and virtual care in a Canadian rural setting: A cross-sectional [illegible]	Fitzsimon, J.; Belanger, C.; Glazier, R.H.; Green, M.E.; Peixoto, C.; Mahdavi, R.; Plumptre, L.; Bjerre, L.M.	2022	medRxiv	NA	(Fitzsimon J., jfitzsi2@uottawa.ca; Belanger [illegible]	NA	10.1101/2022.11.29.22282725	Fitzsimon 2022	Irrelevant	Excluded by TI/AB review

S4 Table. All studies identified in the literature search

3198	Understanding patient preferences for emergency care for lower triage acuity presentations during GP hours: a qualitative study in Australia	Korczak, V.; Yakubu, K.; Angell, B.; Middleton, P.; Dinh, M.; Lung, T.; Jan, S.	2022	BMC health services research	22	1	1442	10.1186/s12913-022-08857-8	Korczak 2022	Irrelevant	Excluded by TI/AB review

S4 Table. All studies identified in the literature search

3199	The Sloop family: Addressing rural health disparities through service and education	Flynn, M.S.; Mosca, P.J.	2022	Journal of medical biography	NA	(Flynn M.S.; Mosca P.J.) 12277 Duke Unive [illegible]	96777202211378 90	10.1177/09677720221137890	Flynn 2022	Irrelevant	Excluded by TI/AB review
3200	Transition from pediatric to adult care in type 1 diabetes mellitus: a longitudinal analysis of age at transfer and gap in care	Soliman, D.; Crowley, M.J.; Manning, A.; Rikhi, A.; Chiswell, K.; Goldstein, B.A.; Maslow, G.	2022	BMJ Open Diabetes Research and Care	10	6	NA	10.1136/bmjdrc-2022-002937	Soliman 2022	Irrelevant	Excluded by TI/AB review
3201	Chronic Medication Nonadherence and Potentially Preventable Healthcare Utilization and Spending Among Medicare Patients	Zhang, Y.; Flory, J.H.; Bao, Y.	2022	Journal of General Internal Medicine	37	14	3645-3652	10.1007/s11606-021-07334-y	Zhang 2022	Irrelevant	Excluded by TI/AB review

S4 Table. All studies identified in the literature search

3202	Randomized Trial Evaluating Health System Expenditures with Transitional Care Services for Adults with No Usual Source of Care at Discharge	Ackermann, R.T.; Liss, D.T.; French, D.D.; Cooper, A.J.; Aikman, C.; Schaeffer, C.	2022	Journal of General Internal Medicine	37	15	3832-3838	10.1007/s11606-022-07473-w	Ackermann 2022	Irrelevant	Excluded by TI/AB review

S4 Table. All studies identified in the literature search

3203	Use of health care utilization as a metric of intervention success may perpetuate racial disparities: An outcome evaluation of a homeless transitional care program	Nohria, R.; Biederman, D.J.; Sloane, R.; Thibault, A.	2022	Public Health Nursing	39	6	1271-1279	10.1111/phn.13121	Nohria 2022	Irrelevant	Excluded by TI/AB review
3204	Characteristics and Outcomes of Patients Transferred for Treatment of Acute Limb Ischemia	Endicott, K.M.; Morton, C.; Tolaymat, B.; Toursavadkohi, S.; Nagarsheth, K.	2022	Annals of Vascular Surgery	87	(Endicott K.M.) Inova Heart and Vascu[illegible]	515-521	10.1016/j.avsg.2022.05.030	Endicott 2022	Irrelevant	Excluded by TI/AB review

S4 Table. All studies identified in the literature search

3205	Racial Disparities in Acute Radiation-Related Toxicities among Patients Diagnosed with Head and Neck Cancer	Cheunkarndee, T.; Kut, C.; Lee, E.E.; Fair, P.T.; McNutt, T.R.; Fakhry, C.; Quon, H.	2022	International Journal of Radiation Oncology Biology Physics	114	3	e289	10.1016/j.ijrobp.2022.07.1315	Cheunkarndee 2022	Irrelevant	Excluded by TI/AB review

S4 Table. All studies identified in the literature search

3206	(48) Implicit Bias and the Misdiagnosis of a Patient with Anti-NMDA Receptor Encephalitis: A Case Report	Cacodcar, N.; Dundar, C.; Somesan, G.; Cheong, J.A.	2022	Journal of the Academy of Consultation-Liaison Psychiatry	63	(Cacodcar N.; Dundar C.; Somesan G.) University of Florida,(Cheong J.A.) U Florida and VISN 8/NF SGVHS,	S136	10.1016/j.jaclp.2022.10.051	Cacodcar 2022	Irrelevant	Excluded by TI/AB review
3207	(46) Examining Racial Bias in the Use of Restraints During Behavioral Emergencies	Arbit, D.; Askalsky, P.; Ying, P.; Caravella, R.A.	2022	Journal of the Academy of Consultation-Liaison Psychiatry	63	(Arbit D.; Caravella R.A.) NYU Langone Health [illegible]	S135	10.1016/j.jaclp.2022.10.049	Arbit 2022	Irrelevant	Excluded by TI/AB review

S4 Table. All studies identified in the literature search

3208	Real-world impact of flaring on patient-reported outcomes and healthcare resource utilisation in systemic lupus [illegible]	Costenbader, K.H.; Hoskin, B.; Atkinson, C.; Bell, D.; Pike, J.; Lofland, J.H.; Berry, P.; Karyekar, C.S.; Touma, Z.	2022	Clinical and Experimental Rheumatology	40	11	2023-2031	10.55563/clinexprheumatol/k9yyeq	Costenbader 2022	Irrelevant	Excluded by TI/AB review
3209	Understanding Influenza and SARS-CoV-2 Vaccine Hesitancy in Racial and Ethnic Minority Caregivers	Baumer-Mouradian, S.H.; Hart, R.J.; Visotcky, A.; Fraser, R.; Prasad, S.; Levas, M.; Nimmer, M.; Brousseau, D.C.	2022	Vaccines	10	11	NA	10.3390/vaccines10111968	Baumer-Mouradian 2022	Irrelevant	Excluded by TI/AB review
3210	Characteristics and Outcomes of Children Discharged With Nasoenteral Feeding Tubes	Ahearn, M.A.; Stephens, J.R.; Zwemer, E.K.; Hall, M.; Ahuja, A.; Chatterjee, A.; Coletti, H.; Fuchs, J.; Lewis, E.; Liles, E.A.; Reade, E.; Sutton, A.G.; [illegible]	2022	Hospital Pediatrics	12	11	969-976	10.1542/hpeds.2022-006627	Ahearn 2022	Irrelevant	Excluded by TI/AB review

S4 Table. All studies identified in the literature search

3211	Risk Factors and Long-term Outcomes in Caregiver-Dependent Trauma Survivors: A Multi-institutional Study	Castillo-Angeles, M.; Herrera-Escobar, J.P.; Hau, K.M.; Serventi-Gleeson, J.; Kaafarani, H.M.A.; Sanchez, S.E.; Salim, A.	2022	Journal of the American College of Surgeons	235	5	S295	10.1097/01.XCS.0000895324.61281.0b	Castillo-Angeles 2022	Irrelevant	Excluded by TI/AB review
3212	The Role of CT in Diagnosing Perianal Abscesses and Prolonged Wait Time in the Emergency Department	Gnana Dev, R.; Malkoc, A.; Lebedevskiy, O.; Hussain, F.; Kim, E.	2022	Journal of the American College of Surgeons	235	5	S57-S58	10.1097/01.XCS.0000893364.98937.6a	GnanaDev 2022	Irrelevant	Excluded by TI/AB review
3213	Fresh Frozen Plasma and Platelets Is Associated with Better Outcomes Even in Trauma Patients Requiring Submassive Transfusion	Jehan, F.S.; Sabegh, B.Z.; Shnaydman, I.; Hanna, K.; Bronstein, M.; Khan, M.N.; Klein, J.; Con, J.; Policastro, A.J.; Prabhakaran, K.	2022	Journal of the American College of Surgeons	235	5	S280-S281	10.1097/01.XCS.0000895212.34817.8b	Jehan 2022	Irrelevant	Excluded by TI/AB review

S4 Table. All studies identified in the literature search

3214	Pneumothorax Was More Common in Covid-Positive Patients during the Delta Surge and Remains Prognostic for Very Poor Outcomes: A Retrospective Cohort Study	Davis, D.; Barbera, A.; Rosever, K.; Jacklin, K.; Reyes, P.; Wills, W.; Bugajski, A.	2022	Journal of the American College of Surgeons	235	5	S88-S89	10.1097/01.XCS.0000896468.49434.28	Davis 2022	Irrelevant	Excluded by TI/AB review

S4 Table. All studies identified in the literature search

3215	Adiposity and Racial Disparities Burden in an Adolescent Cohort: A 3-Year Pragmatic Experience	Velasquez, A.; Velasquez-Mieyer, P.A.; Watkins, S.; Mou, X.; Nieto, R.; Neira, C.P.; Mechanick, J.; Cherrington, A.; Levy, M.; Grant, C.; Rodriguez, P.A.V.	2022	Obesity	30	(Velasquez A.) Memphis, TN, United States (Velasquez-Mieye[illegible]	163-164	10.1002/oby.23626	Velasquez 2022	Irrelevant	Excluded by TI/AB review

S4 Table. All studies identified in the literature search

3216	Pseudohypoparathyroidism Type 1B: A Medication Compliance Challenge	Sacco, S.B.; Echegoyen, F.B.; Lagari-Libhaber, V.	2022	Journal of the Endocrine Society	6	(Sacco S.B.; Echegoyen F.B.; Lagari-Libhaber V.)	A203-A204	10.1210/jendso/bvac150.418	Sacco 2022	Irrelevant	Excluded by TI/AB review
3217	Media and technology usage and attitudes in emergency department patients	Goldfine, C.E.; Knapp, A.; Goodman, G.R.; Hasdianda, M.A.; Huang, H.; Marshall, A.D.; Keschner, [illegible]	2022	Frontiers in Digital Health	4	(Goldfine C.E., cgoldfine@bwh.ha[illegible]	NA	10.3389/fdgth.2022.894683	Goldfine 2022	Irrelevant	Excluded by TI/AB review

S4 Table. All studies identified in the literature search

3218	Trends in US Emergency Department Use after Sexual Assault, 2006-2019	Vogt, E.L.; Jiang, C.; Jenkins, Q.; Millette, M.J.; Caldwell, M.T.; Mehari, K.S.; Marsh, E.E.	2022	JAMA Network Open	5	10	E2236273	10.1001/jamanetworkopen.2022.36273	Vogt 2022	Irrelevant	Excluded by TI/AB review
3219	Racial Disparities in Hospitalization Among Patients Who Receive a Diagnosis of Acute Coronary Syndrome in the Emergency [illegible]	Islek, D.; Ali, M.K.; Manatunga, A.; Alonso, A.; Vaccarino, V.	2022	Journal of the American Heart Association	11	19	NA	10.1161/JAHA.122.025733	Islek 2022	Irrelevant	Excluded by TI/AB review
3220	End-of-life healthcare utilization and palliative care use among older adults with limited [illegible]	Abedini, N.C.; Downey, L.; Engelberg, R.A.; Curtis, J.R.; Sharma, R.K.	2022	Journal of the American Geriatrics Society	70	10	2847-2857	10.1111/jgs.17913	Abedini 2022	Irrelevant	Excluded by TI/AB review

S4 Table. All studies identified in the literature search

3221	Healthcare quality during pediatric mental health boarding: A qualitative analysis	McCarty, E.J.; Nagarajan, M.K.; Halloran, S.R.; Brady, R.E.; House, S.A.; Leyenaar, J.K.	2022	Journal of Hospital Medicine	17	10	783-792	10.1002/jhm.12906	McCarty 2022	Irrelevant	Excluded by TI/AB review
3223	Resident assistant training level is not associated with patient spinal fusion outcomes	Borja, A.J.; Ahmad, H.S.; Ghenbot, Y.; Na, J.; McClintock, S.D.; Mueller, K.B.; Burkhardt, J.-K.; Yoon, J.W.; Malhotra, N.R.	2022	Clinical Neurology and Neurosurgery	221	(Borja A.J.; Ahmad H.S.; Ghenbot [illegible]	NA	10.1016/j.clineuro.2022.107388	Borja 2022	Irrelevant	Excluded by TI/AB review

S4 Table. All studies identified in the literature search

3224	Patient perspectives of the climate of diversity, equity, and inclusion in the emergency department	Davuluri, K.; Goyal, N.; Gomez Acevedo, H.; Folt, J.; Jayaprakash, N.; Slezak, M.; Caldwell, M.T.	2022	JACEP Open	3	5	NA	10.1002/emp2.12798	Davuluri 2022	Irrelevant	Excluded by TI/AB review
3225	Invited Editorial: Patient perspectives of the climate of diversity, equity, and inclusion in the emergency department	March, J.; Feng, S.-Y.; Donnelly, E.A.	2022	JACEP Open	3	5	NA	10.1002/emp2.12805	Mar-22	Irrelevant	Excluded by TI/AB review
3226	270 Code De-Escalation: Effectiveness and Feasibility Pilot Study of Intervention to Decrease Restraint [illegible]	Bukhman, A.; Im, D.; Powell, R.; Grant, J.; Schmelzer, N.; Baymon, D.; Callahan, C.; Kim, I.; Waters, B.; Sanchez, L.; Chen, P.	2022	Annals of Emergency Medicine	80	4	S118	10.1016/j.annemergmed.2022.08.297	Bukhman 2022	Irrelevant	Excluded by TI/AB review

S4 Table. All studies identified in the literature search

3227	90 Economic Impact: A Cluster Randomized Trial of a Rapid, High-Sensitivity Cardiac Troponin I Protocol	Miller, J.; Nassereddine, H.; Jennings, K.; Connor, S.; Modi, S.; Yan, J.; McCord, J.; Cook, B.; Danagoulian, S.	2022	Annals of Emergency Medicine	80	4	S45	10.1016/j.annemergmed.2022.08.113	Miller 2022	Irrelevant	Excluded by TI/AB review
3228	338 A National Snapshot of Social Determinants of Health Documentation in Emergency Departments	Ryus, C.; Janke, A.; Granovsky, R.; Granovsky, M.	2022	Annals of Emergency Medicine	80	4	S144	10.1016/j.annemergmed.2022.08.365	Ryus 2022	Irrelevant	Excluded by TI/AB review
3229	8 Comparison of One-year Outcomes by Management Type in Patients Presenting to the Emergency Department With Uncomplicated [illegible]	Makutonin, M.; Newton, S.; Tse, J.; Ma, Y.; Moghtaderi, A.; Meltzer, A.	2022	Annals of Emergency Medicine	80	4	S4	10.1016/j.annemergmed.2022.08.030	Makutonin 2022	Irrelevant	Excluded by TI/AB review

S4 Table. All studies identified in the literature search

3230	93 Patients With Sickle Cell Disease Pain Crises Are Often Undertreated in the Out-of-Hospital Setting: A Multi- [illegible]	Johnson, R.; Hern, H.G.; Taigman, M.; Whim, A.; Crowe, R.	2022	Annals of Emergency Medicine	80	4	S46	10.1016/j.annemergmed.2022.08.116	Johnson 2022	Irrelevant	Excluded by TI/AB review
3231	165 Trends in Emergency Department Dental Pain Visits During the COVID19 Pandemic	Nilz, M.; Sharma, D.; Schneider, S.; Vithalani, A.	2022	Annals of Emergency Medicine	80	4	S74-S76	10.1016/j.annemergmed.2022.08.189	Nilz 2022	Irrelevant	Excluded by TI/AB review
3232	164 The Impact of Hallway Placement on Emergency Department Operations for Discharged Patients with Abdominal Pain	Sangal, R.; Su, H.; Meng, L.; Venkatesh, A.	2022	Annals of Emergency Medicine	80	4	S74	10.1016/j.annemergmed.2022.08.188	Sangal 2022	Irrelevant	Excluded by TI/AB review

S4 Table. All studies identified in the literature search

3233	91 The Promising Use of an Emergency Department Observation Unit to Manage Patients With Opioid Use Disorder	Tran, T.; Dym, A.; Rosania, A.; Nelson, L.; Ramdin, C.; Santos, C.	2022	Annals of Emergency Medicine	80	4	S45	10.1016/j.annemergmed.2022.08.114	Tran 2022	Irrelevant	Excluded by TI/AB review
3234	399 Racial and Ethnic Variation in Emergency Department Disposition and Access to Hospital-[illegible]	Khidir, H.; Janke, A.; Rothenberg, C.; Li, S.-X.; Venkatesh, A.	2022	Annals of Emergency Medicine	80	4	S170-S171	10.1016/j.annemergmed.2022.08.419	Khidir 2022	Irrelevant	Excluded by TI/AB review
3235	37 Inequities Among Emergency Department Hallway Utilization	Sangal, R.; Su, H.; Meng, L.; Venkatesh, A.	2022	Annals of Emergency Medicine	80	4	S17	10.1016/j.annemergmed.2022.08.060	Sangal 2022	Irrelevant	Excluded by TI/AB review

S4 Table. All studies identified in the literature search

3236	96 Using a Digital ,ÄúEquity Dashboard,Äù to Understand Language Disparities in Time to Pain Medication	Burke, C.; Yi, S.; Straube, S.; Graterol, J.; Peabody, C.	2022	Annals of Emergency Medicine	80	4	S47-S48	10.1016/j.annemergmed.2022.08.119	Burke 2022	Irrelevant	Excluded by TI/AB review
3237	49 Comparing the Safety and Efficacy of a Rapid High-Sensitivity Cardiac Troponin I Protocol Between [illegible]	Miller, J.; Gunaga, S.; Krupp, S.; Klausner, H.; Plemmons, E.; Nasseredine, H.; Tuttle, J.; Husain, A.; Cook, B.; McCord, J.	2022	Annals of Emergency Medicine	80	4	S26	10.1016/j.annemergmed.2022.08.072	Miller 2022	Irrelevant	Excluded by TI/AB review
3238	249 Differences in Emergency Department Sepsis Care: Do Race, Sex, and Language Matter?	Nacier, C.; Wilson, T.; George, N.; Lin, T.; Brister, J.; Madsen, T.; Napoli, A.; Jenouri, I.; Tyminski, C.; Samuels, E.	2022	Annals of Emergency Medicine	80	4	S109	10.1016/j.annemergmed.2022.08.271	Nacier 2022	Irrelevant	Excluded by TI/AB review

S4 Table. All studies identified in the literature search

3239	72 Association of Limited English Proficiency and Increased Emergency Department Waiting Room Lengths of Stay	Lim, T.; Campbell, R.; Jones, D.; Mullan, A.; Lichen, I.; Knier, C.; Bellamkonda, V.	2022	Annals of Emergency Medicine	80	4	S36-S37	10.1016/j.annemergmed.2022.08.095	Lim 2022	Irrelevant	Excluded by TI/AB review
3240	351 The Accuracy of Handheld Ultrasound in the Evaluation of Symptomatic Pregnant Patients [illegible]	Asikhia, O.; Bhatnagar, N.; Au, A.; Lewiss, R.; Fields, M.; Chang, A.; Maloney, K.; Chu, T.; Bollinger, [illegible]	2022	Annals of Emergency Medicine	80	4	S151	10.1016/j.annemergmed.2022.08.380	Asikhia 2022	Irrelevant	Excluded by TI/AB review
3241	329 Epidemiology and Outcomes of Out-of-Hospital Cardiac Arrest Patients in Flint, MI	Kenerson, R.; Cranford, J.; Reece, R.	2022	Annals of Emergency Medicine	80	4	S140	10.1016/j.annemergmed.2022.08.357	Kenerson 2022	Irrelevant	Excluded by TI/AB review
3242	285 Care for the Detainee in the Emergency Department Utilizing Simulation	Elue, J.; Vasa, A.; Winfield, A.; Amin, D.	2022	Annals of Emergency Medicine	80	4	S124	10.1016/j.annemergmed.2022.08.312	Elue 2022	Irrelevant	Excluded by TI/AB review

S4 Table. All studies identified in the literature search

3243	312 Racial Differences in Adult Emergency Department Triage Assignment Across An Urban-Rural Health [illegible]	Puissant, M.; Gunnell, H.; Cutler, A.; Scharnetzki, E.; Strout, T.; Agarwal, I.	2022	Annals of Emergency Medicine	80	4	S133-S134	10.1016/j.annemergmed.2022.08.340	Puissant 2022	Irrelevant	Excluded by TI/AB review
3244	235 COVID-19 Booster Vaccine Hesitancy in the Emergency Department	Molina, M.; Nichol, G.; Eucker, S.; Arreguin, M.; Morse, D.; Pauley, A.; Chavez, C.; O'Laughlin, K.; Rodriguez, R.	2022	Annals of Emergency Medicine	80	4	S103	10.1016/j.annemergmed.2022.08.258	Molina 2022	Irrelevant	Excluded by TI/AB review
3245	75 Disparities in Emergency Department Wait Times for Female, Transgender Female, Black and Non-English-Speaking Patients	Canellas, M.; Perakis, G.; Pachamanova, D.; Lami, O.S.; Tsiourvas, A.	2022	Annals of Emergency Medicine	80	4	S39	10.1016/j.annemergmed.2022.08.098	Canellas 2022	Irrelevant	Excluded by TI/AB review
3246	219 Socioeconomic and Racial/Ethnic Disparities in Out-	Crowe, R.; Kennel, J.; Fernandez, A.; Bourn, S.; Burton, B.; Van Vleet, L.;	2022	Annals of Emergency Medicine	80	4	S96-S97	10.1016/j.annemergmed.2022.08.243	Crowe 2022	Irrelevant	Excluded by TI/AB review

S4 Table. All studies identified in the literature search

3247	139 Inequities of Emergency Department Queue Jumping	Sangal, R.; Su, H.; Meng, L.; Venkatesh, A.	2022	Annals of Emergency Medicine	80	4	S64-S65	10.1016/j.annemergmed.2022.08.163	Sangal 2022	Irrelevant	Excluded by TI/AB review
3248	142 Emergency Department-Initiated Buprenorphine for Opioid Use Disorder: Impact on Patient Outcomes at a Community Hospital	Lee, D.; Biggs, D.; Neyman, G.; Ashok, N.; Johnkunthy, N.; Gevorgian, H.; Vassallo, A.; Maguire, N.	2022	Annals of Emergency Medicine	80	4	S66	10.1016/j.annemergmed.2022.08.166	Lee 2022	Irrelevant	Excluded by TI/AB review
3249	198 Rapid Resuscitation for Hemorrhagic Shock: Hemodynamically Unstable Patients May Do Better Than Those Presenting with Normal Vitals	De Maio, V.; Wanda, L.; James, E.; Adejumo, F.; Piehl, M.	2022	Annals of Emergency Medicine	80	4	S89-S90	10.1016/j.annemergmed.2022.08.222	DeMaio 2022	Irrelevant	Excluded by TI/AB review

S4 Table. All studies identified in the literature search

3250	121 Social and Structural Influences of Emergency Care Seeking Behaviors in Documented [illegible]	Ramirez, C.; Garcia, V.; Mendoza, C.; Mansilla, M.; Alata, C.; Burner, E.	2022	Annals of Emergency Medicine	80	4	S58	10.1016/j.annemergmed.2022.08.145	Ramirez 2022	Irrelevant	Excluded by TI/AB review
3251	61 Designing and Developing A Digital Equity Dashboard For the Emergency Department	Yi, S.; Burke, C.; Reilly, A.; Straube, S.; Graterol, J.; Peabody, C.	2022	Annals of Emergency Medicine	80	4	S31-S32	10.1016/j.annemergmed.2022.08.084	Yi 2022	Irrelevant	Excluded by TI/AB review
3252	RISK FACTORS FOR COVID-19 CRITICAL ILLNESS AMONG TWO URBAN PUBLIC HOSPITALS	Graziani, C.; A Staggers, K.; Kumari Narendra, D.; Li, A.N.G.; M Patel, S.; El Sahly, H.A.N.A.; A Hanania, N.	2022	Chest	162	4	A475-A476	10.1016/j.chest.2022.08.365	Graziani 2022	Irrelevant	Excluded by TI/AB review
3253	DOCTORS WITHOUT "BOARDERS": EFFECTS OF BOARDING MEDICAL ICU PATIENTS	Sheldon, D.R.E.W.; Choi, J.H.; Castillo, V.L.; Vartanyan, A.R.A.; Lee, M.M.	2022	Chest	162	4	A2642	10.1016/j.chest.2022.08.2158	Sheldon 2022	Irrelevant	Excluded by TI/AB review

S4 Table. All studies identified in the literature search

3254	ASSOCIATION OF SEPSIS SYMPTOMS AT ED PRESENTATION WITH RACE AND ETHNICITY	Newby, R.; Mishra, P.; Barreda, F.; Myers, L.; Kipnis, P.; Liu, V.	2022	Chest	162	4	A1452	10.1016/j.chest.2022.08.1221	Newby 2022	Irrelevant	Excluded by TI/AB review
3255	DILATED CARDIOMYOPATHY WITH CARDIOGENIC SHOCK SECONDARY [illegible]	Richardson, A.; Cecchini, A.; K Solanki, K.	2022	Chest	162	4	A281-A282	10.1016/j.chest.2022.08.214	Richardson 2022	Irrelevant	Excluded by TI/AB review
3256	Impact of COVID-19 pandemic on emergency department attendances for young people	Solanke, F.; Easton, S.; Selby, A.; James, D.; Roberts, G.	2022	Archives of Disease in Childhood	107	10	E27	10.1136/archdischild-2021-323389	Solanke 2022	Irrelevant	Excluded by TI/AB review

S4 Table. All studies identified in the literature search

3258	Opportunity loss: care team job satisfaction and urgent care, emergency department, and hospital costs for patients with [illegible]	Mundt, M.P.; Zakletskaia, L.I.	2022	Family Practice	39	5	868-874	10.1093/fampra/cmac023	Mundt 2022	Irrelevant	Excluded by TI/AB review

S4 Table. All studies identified in the literature search

3259	Racial and socioeconomic disparities in the advanced treatment of medically intractable pediatric epilepsy	Kandregula, S.; Terrell, D.; Beyl, R.; Freelin, A.; Guthikonda, B.; Notarianni, C.; Toms, J.	2022	Neurosurgical focus	53	4	E2	10.3171/2022.7.FOCUS22338	Kandregula 2022	Irrelevant	Excluded by TI/AB review

S4 Table. All studies identified in the literature search

3260	INCREASED EMERGENCY DEPARTMENT VISITS FOR FOREIGN BODY INGESTIONS REQUIRING [illegible]	Spad, M.-A.; Aiyuk, A.; Fatch, S.; Raghunathan, S.; Rajendra, C.; Ali, S.	2022	Journal of Pediatric Gastroenterology and Nutrition	75	(Spad M.-A.; Aiyuk A.; Fatch S.; Raghu [illegible]	S170-S171	NA	Spad 2022	Irrelevant	Excluded by TI/AB review

S4 Table. All studies identified in the literature search

3261	North American Society for Pediatric Gastroenterology, Hepatology and Nutrition Scientific Abstracts	NA	2022	Journal of Pediatric Gastroenterology and Nutrition	75	NA	NA	NA	NA	Irrelevant	Excluded by TI/AB review

S4 Table. All studies identified in the literature search

3262	OUTPATIENT INTEGRATION OF CARE FOR PEDIATRIC PATIENTS WITH GASTROSTOMY TUBES	Chan, P.Z.; Oh, P.S.; Rose, M.	2022	Journal of Pediatric Gastroenterology and Nutrition	75	(Chan P.Z.; Rose M.) Pediatric GI, Weill Cornell Medic[illegible]	S14-S15	NA	Chan 2022	Irrelevant	Excluded by TI/AB review
3263	Barriers to Inflammatory Bowel Disease (IBD) Care in a Racially and Ethnically Diverse Population: Patient Survey and Chart [illegible]	Shah, S.; Kabagambe, E.K.; Shillington, A.; Patel, A.A.; Capelouto, J.A.; Deering, K.L.; Elmayan, A.; Babin, S.; LaChappelle, B.; Liu, J.	2022	American Journal of Gastroenterology	117	10	S764	10.14309/01.ajg.0000860856.23990.7d	Shah 2022	Irrelevant	Excluded by TI/AB review
3264	Clinical Setting and Outcomes in Patients Undergoing Endoscopic Disimpaction of Esophageal Food Bolus	Bains, K.; Roussel, B.; Monteiro, J.F.; Bhowmick, K.; Kalligeros, M.; Fine, S.	2022	American Journal of Gastroenterology	117	10	S437	10.14309/01.ajg.0000859156.28164.b9	Bains 2022	Irrelevant	Excluded by TI/AB review

S4 Table. All studies identified in the literature search

3265	Acupuncture for acute musculoskeletal pain management in the emergency department and continuity clinic: a protocol for an adaptive pragmatic randomised controlled trial	Eucker, S.A.; Glass, O.; Staton, C.A.; Knisely, M.R.; O'regan, A.; De Larco, C.; Mill, M.; Dixon, A.; Tumsuden, O.; Walker, E.; Dalton, J.C.; Limkakeng, A.; Maxwell, A.M.W.; Gordee, A.; Kuchibhatla, M.; Chow, S.	2022	BMJ Open	12	9	NA	10.1136/bmjopen-2022-061661	Eucker 2022	Irrelevant	Excluded by TI/AB review
3266	Social determinants of participant recruitment and retention in a prospective cohort	Wells, J.M.; Galarneau, J.-M.; Minich, N.M.; Cohen, D.M.; Clinton, K.; Taylor, H.G.; Bigler, E.D.;	2022	Frontiers in Neurology	13	(Wells J.M., Jordee.Wells @nationwid	NA	10.3389/fneur.2022.961024	Wells 2022	Irrelevant	Excluded by TI/AB review

S4 Table. All studies identified in the literature search

3268	Incidence and trends of decision regret following elective hernia repair	Howard, R.; Ehlers, A.; Delaney, L.; Solano, Q.; Fry, B.; Englesbe, M.; Dimick, J.; Telem, D.	2022	Surgical Endoscopy	36	9	6609-6616	10.1007/s00464-021-08766-7	Howard 2022	Irrelevant	Excluded by TI/AB review
3269	Factors Related to Biologic Adherence and Outcomes Among Moderate-to-Severe Asthma Patients	Osazuwa-Peters, O.L.; Greiner, M.A.; Oberle, A.; Oakes, M.; Thomas, S.M.; Bosworth, H.	2022	Journal of Allergy and Clinical Immunology: In Practice	10	9	2355-2366	10.1016/j.jaip.2022.05.022	Osazuwa-Peters 2022	Irrelevant	Excluded by TI/AB review

S4 Table. All studies identified in the literature search

3270	Telepsychiatry services across an emergency department network: A mixed methods study of [illegible]	Vakkalanka, J.P.; Nataliansyah, M.M.; Merchant, K.A.S.; Mack, L.J.; Parsons, S.; Mohr, N.M.; Ward, M.M.	2022	American Journal of Emergency Medicine	59	(Vakkalanka J.P., priyanka-vakkal [illegible]	79-84	10.1016/j.ajem.2022.06.053	Vakkalanka 2022	Irrelevant	Excluded by TI/AB review
3271	Risk Factors for Preventable Emergency Department Use After Outpatient Hand Surgery	Shetty, P.N.; Guarino, G.M.; Zhang, G.; Sanghavi, K.K.; Giladi, A.M.	2022	Journal of Hand Surgery	47	9	855-864	10.1016/j.jhsa.2022.05.012	Shetty 2022	Irrelevant	Excluded by TI/AB review
3272	100. Disparities in elective spine surgery for Medicaid beneficiaries: a systematic review	Badin, D.; Ortiz-Babilonia, C.; Musharbash, F.; Jain, A.	2022	Spine Journal	22	9	S54-S55	10.1016/j.spinee.2022.06.118	Badin 2022	Irrelevant	Excluded by TI/AB review
3273	P92. The association between patient portal utilization and clinical outcomes	Baeta, C.; Owolo, E.; Charles, A.; Petitt, Z.; Poehlein, E.; Cook, C.; Sperber, J.; Goodwin, C.R.; Erickson, M.; Green, C.	2022	Spine Journal	22	9	S170	10.1016/j.spinee.2022.06.349	Baeta 2022	Irrelevant	Excluded by TI/AB review

S4 Table. All studies identified in the literature search

3274	P88. Influence of time of discharge and length of stay on 30-day outcomes after elective lumbar spine surgery	Sastry, R.; Hagan, M.; Telfeian, A.E.; Gokaslan, Z.L.	2022	Spine Journal	22	9	S168-S169	10.1016/j.spinee.2022.06.345	Sastry 2022	Irrelevant	Excluded by TI/AB review
3275	154. Social Vulnerability Index (SVI) can be used to predict health care resource utilization and persistent opioid use after elective lumbar [illegible]	Gerlach, E.B.; Plantz, M.A.; Arpey, N.; Swiatek, P.; Alvandi, B.; Marx, J.; Goedderz, C.; Divi, S.; Hsu, W.K.; Patel, A.A.	2022	Spine Journal	22	9	S82	10.1016/j.spinee.2022.06.173	Gerlach 2022	Irrelevant	Excluded by TI/AB review

S4 Table. All studies identified in the literature search

3276	Geographic proximity to primary care providers as a risk-assessment criterion for quality performance measures	Bell, N.; L√≤pez-De Fede, A.; Cai, B.; Brooks, J.	2022	PLoS ONE	17	9-Sep	NA	10.1371/journal.pone.0273805	Bell 2022	Irrelevant	Excluded by TI/AB review
3277	Dynamic accessibility by car to tertiary care emergency services in Cali, Colombia, in 2020: Cross- [illegible]	Cuervo, L.G.; Martinez-Herrera, E.; Osorio, L.; Hatcher-Roberts, J.; Cuervo, D.; Bula, M.O.; Pinilla, L.F.; Piquero, F.; Jaramillo, C.	2022	BMJ Open	12	9	NA	10.1136/bmjopen-2022-062178	Cuervo 2022	Irrelevant	Excluded by TI/AB review
3278	Does Socioeconomic Profile Predict Etiology of Firearm Injuries To Head And Neck?	Stern, E.; Manhan, A.J.; Smith, R.; Abramowicz, S.; Amin, D.	2022	Journal of Oral and Maxillofacial Surgery	80	9	S12	10.1016/j.joms.2022.07.021	Stern 2022	Irrelevant	Excluded by TI/AB review

S4 Table. All studies identified in the literature search

3279	Postpartum Hospital Readmissions and Emergency Department Visits Among High-Risk, Medicaid-Insured Women in New York City	Glazer, K.B.; Harrell, T.; Balbierz, A.; Howell, E.A.	2022	Journal of Women's Health	31	9	1305-1313	10.1089/jwh.2021.0338	Glazer 2022	Irrelevant	Excluded by TI/AB review
3281	Stage-of-change Assessment Predicts Short-term Treatment Engagement for Opioid Use Disorder Patients Initiated on [illegible]	Reuter, Q.; Larkin, G.L.; Vellanki, M.D.S.; Dos Santos, A.; McKinnon, J.; Jouriles, N.; Seaberg, D.	2022	Western Journal of Emergency Medicine	23	5	684-692	10.5811/westjem.2022.3.53197	Reuter 2022	Irrelevant	Excluded by TI/AB review
3282	Association of health care utilization and costs with patient-reported outcomes in patients with ankylosing spondylitis	Ogdie, A.; Hwang, M.; Veeranki, P.; Portelli, A.; Sison, S.; Shafrin, J.; Pedro, S.; Kim, N.; Yi, E.; Michaud, K.	2022	Journal of Managed Care and Specialty Pharmacy	28	9	1008-1020	NA	Ogdie 2022	Irrelevant	Excluded by TI/AB review

S4 Table. All studies identified in the literature search

3283	Complement activation products vs standard ANA testing: Treatment outcomes, diagnosis, and economic impact (CAPSTONE) in systemic lupus erythematosus	O,ÄôMalley, T.; Xie, F.; Su, Y.; Clinton, C.; Zack, D.J.; Haechung, C.; Grabner, M.; Curtis, J.R.	2022	Journal of Managed Care and Specialty Pharmacy	28	9	1021-1032	NA	O,ÄôMalley 2022	Irrelevant	Excluded by TI/AB review
3284	Risk Factors and Neurological Outcomes Associated With Circulatory Shock After Moderate-Severe Traumatic Brain Injury: A [illegible]	Toro, C.; Hatfield, J.; Temkin, N.; Barber, J.; Manley, G.; Ohnuma, T.; Komisarow, J.; Foreman, B.; Korley, F.K.; Vavilala, M.S.; Laskowitz, D.T.; [illegible]	2022	Neurosurgery	91	3	427-436	10.1227/neu.0000000000002042	Toro 2022	Irrelevant	Excluded by TI/AB review
3285	Delivery of High-Quality Head and Neck Microvascular Reconstruction in Underserved Patients With a [illegible]	Faries, E.; Hernandez, D.J.; Huang, A.T.; Haskins, A.D.; Shum, J.	2022	Otolaryngology - Head and Neck Surgery	167	1	P198	10.1177/01945998221076	Faries 2022	Irrelevant	Excluded by TI/AB review

S4 Table. All studies identified in the literature search

3286	A 10-Year Analysis of Pediatric Airway Foreign Bodies by Demographics, Disposition, and Foreign Body	Hoerter, J.; Hosaka, L.; Saba, E.; Liang, J.	2022	Otolaryngology - Head and Neck Surgery	167	1	P251-P252	10.1177/019459982211076	Hoerter 2022	Irrelevant	Excluded by TI/AB review
3287	Socioeconomic Barriers to Care for Children With Tracheostomies Utilizing [illegible]	Fadel, M.; McCoy, J.; Shaffer, A.D.; Kurland, K.; Simons, J.	2022	Otolaryngology - Head and Neck Surgery	167	1	P132	10.1177/019459982211 07672	Fadel 2022	Irrelevant	Excluded by TI/AB review

S4 Table. All studies identified in the literature search

3288	National Analysis of Predictors of Time-to-Operation for Facial Fractures	Sharma, R.; Landeen, K.; Davis, S.; Yang, S.; Stephan, S.; Patel, P.	2022	Otolaryngology - Head and Neck Surgery	167	1	P189	10.1177/01945998221076	Sharma 2022	Irrelevant	Excluded by TI/AB review

S4 Table. All studies identified in the literature search

3289	Safe Day of Surgery Discharge for Artificial Urinary Sphincters - No Change in Outcomes or Complications	Vasan, R.; Myrga, J.; Chun, B.; Miller, D.; Taylor, C.; Morrill, C.; Hacker, E.; Rusilko, P.	2022	Journal of Endourology	36	(Vasan R.; Myrga J.; Chun B.; Miller D.; Taylor C.; Morrill C.; Hacker E.; Rusilko P.) UPMC, United States	A256	10.1089/end.2022.36001.abstracts	Vasan 2022	Irrelevant	Excluded by TI/AB review

S4 Table. All studies identified in the literature search

3290	The Problem of Pain in Rheumatology: Clinical Profiles Associated with Concomitant Diagnoses with Chronic Overlapping Pain Conditions	Falasinnu, T.; Chaichian, Y.; Simard, J.	2022	Arthritis and Rheumatology	74	(Falasinnu T.) Stanford School of Medicine, Stanford, CA, United States (Chaichian Y.) Stanford University, Stanford, CA, Unite[illegible]	2392-2394	10.1002/art.42355	Falasinnu 2022	Irrelevant	Excluded by TI/AB review

S4 Table. All studies identified in the literature search

3291	A United States Nationwide Analysis of Total Knee Arthroplasty in Patients < 21 Years Old	Gibbons, J.; Kahlenberg, C.A.; Jannat-Khah, D.; Christ, A.B.; Goodman, S.; Sculco, P.; Figgie, M.; Mehta, B.	2022	Arthritis and Rheumatology	74	(Gibbons J.; Kahlenberg C.A.; Jannat-Khah D.; Goodman S.; Sculco P.; Figgie M.) Hospital for Special Surgery, New York, NY, United	1734-1737	10.1002/art.42355	Gibbons 2022	Irrelevant	Excluded by TI/AB review

S4 Table. All studies identified in the literature search

3292	Smartphone-based remote monitoring (RM) in chronic heart failure reduces emergency hospital attendances unplanned admissions and secondary care costs: A retrospective cohort study	Padayachee, Y.; Shah, M.; Auton, A.; Samways, J.; Quaife, N.; Kamalati, T.; Tenorio, I.; Bachtiger, P.; Howard, J.P.; Cole, G.D.; Barton, C.; Peters, N.S.; Plymen, C.M.; Zaman, S.	2022	European Respiratory Journal	60	(Padayachee Y.; Auton A.; Samways J.; Quaife N.; Tenorio I.; Bachtiger P.; Howard J.P.; Cole G.D.; Barton C.;	2816	10.1093/eurheartj/ehac544.2816	Padayachee 2022	Irrelevant	Excluded by TI/AB review

S4 Table. All studies identified in the literature search

3293	Real-world experience of hospitalized COVID-19 patients requiring mechanical ventilation	Rasouliyan, L.; Althoff, A.G.; Kumar, V.; Long, S.; Rao, M.B.	2022	Pharmacoepidemiology and Drug Safety	31	(Rasouliyan L.; Althoff A.G.; Kumar V.; Long S.; Rao M.B.) OMNY Health, United States	508	10.1002/pds.5518	Rasouliyan 2022	Irrelevant	Excluded by TI/AB review
3294	Children with COVID-19: The real-world hospital experience	Rasouliyan, L.; Kumar, V.; Althoff, A.G.; Long, S.; Rao, M.B.	2022	Pharmacoepidemiology and Drug Safety	31	(Rasouliyan L.; Kumar V.; Althoff A.G.; Long S.; [illegible]	371	10.1002/pds.5518	Rasouliyan 2022	Irrelevant	Excluded by TI/AB review

S4 Table. All studies identified in the literature search

3295	Patient Perceptions about Opioid Risk Communications Within the Context of a Randomized Clinical Trial	Dolan, A.R.; Goldberg, E.B.; Cannuscio, C.C.; Abrams, M.P.; Feuerstein-Simon, R.; Luna Marti, X.; [illegible]	2022	JAMA Network Open	5	8	E2227650	10.1001/jamanetworkopen.2022.27650	Dolan 2022	Irrelevant	Excluded by TI/AB review
3296	Examining the incidence of acute stress in pediatric trauma patients	Schauss, E.; Hawes, K.; Roberts, S.; Clayton, J.M.; Li, C.; Littlejohn, A.; Bartelli, D.; Williams, R.	2022	Trauma Surgery and Acute Care Open	7	1	NA	10.1136/tsaco-2022-000946	Schauss 2022	Irrelevant	Excluded by TI/AB review

S4 Table. All studies identified in the literature search

3297	Geographic variation in the treatment of spinal disorders: association with health care professional availability, and population socioeconomic status, race, and ethnicity. A retrospective cohort study	Elton, D.; Zhang, M.; Okaya, A.	2022	medRxiv	NA	(Elton D., david.elton@optum.com; Zhang M.; Okaya A.) Optum Labs, Minnetonka, MN, United States	NA	10.1101/2022.08.15.22278722	Elton 2022	Irrelevant	Excluded by TI/AB review

S4 Table. All studies identified in the literature search

3298	Do racial disparities exist at various time points during an emergency department visit for chest pain	Allamby, C.; Scott, T.; Krizo, J.; Consing, K.; Mangira, C.; Simon, E.L.	2022	American Journal of Emergency Medicine	58	(Allamby C.; Scott T.; Consing K.) Cleveland Clinic Akron General, Department	4-Jan	10.1016/j.ajem.2022.04.029	Allamby 2022	Irrelevant	Excluded by TI/AB review
3299	Prediction of Influenza Complications: Development and Validation of a Machine Learning Prediction Model to Improve and [illegible]	Wolk, D.M.; Lanyado, A.; Tice, A.M.; Shermohammed, M.; Kinar, Y.; Goren, A.; Chabris, C.F.; Meyer, M.N.; Shoshan, A.; Abedi, [illegible]	2022	Journal of Clinical Medicine	11	15	NA	10.3390/jcm11154342	Wolk 2022	Irrelevant	Excluded by TI/AB review

S4 Table. All studies identified in the literature search

3301	Impact of the COVID-19 pandemic on pediatric emergency department utilization for head injuries	Satoskar, S.; Badaki, O.B.; Gielen, A.C.; McDonald, E.M.; Ryan, L.M.	2022	Journal of Investigative Medicine	70	6	1416-1422	10.1136/jim-2021-002292	Satoskar 2022	Irrelevant	Excluded by TI/AB review

S4 Table. All studies identified in the literature search

3302	Palliative medicine referral and end-of-life interventions among minority patients with advanced or metastatic gynecologic cancer (433)	Tabuyo-Martin, A.; Morales, A.T.; Pitteloud, M.; Kshetry, A.; Schlumbrecht, M.; Sanchez, J.	2022	Gynecologic Oncology	166	(Tabuyo-Martin A.; Kshetry A.) University of Miami / Jackson Memorial Hospital, Miami, FL, United States (Morales A.T.; Pittelo[illegible]	S218	10.1016/S0090-8258(22)01655-9	Tabuyo-Martin 2022	Irrelevant	Excluded by TI/AB review

S4 Table. All studies identified in the literature search

3303	Cognitive deficit identified via patient reported outcome measures may be a predictor for post-operative health care utilization (131)	Saini, A.; Orav, E.; Philp, L.; Mazina, V.; Sullivan, M.; Alimena, S.; del Carmen, M.; Goodman, A.; Eisenhauer, E.; Sisodia, R.	2022	Gynecologic Oncology	166	(Saini A.) University of Massachusetts, Boston, MA	S82	10.1016/S0090-8258(22)01356-7	Saini 2022	Irrelevant	Excluded by TI/AB review
3304	Association between Implementation of a Geriatric Trauma Clinical Pathway and Changes in Rates of Delirium in Older Adults with Traumatic Injury	Park, C.; Bharija, A.; Mesias, M.; Mitchell, A.; Krishna, P.; Storr-Street, N.; Brown, A.; Martin, M.; Lu, A.C.; Staudenmayer, K.L.	2022	JAMA Surgery	157	8	676-683	10.1001/jamasurg.2022.1556	Park 2022	Irrelevant	Excluded by TI/AB review
3305	POINT OF CONTACT: LGBTQ ISSUES IN THE CHILDREN'S ED	Davenport, L.; Rees, D.	2022	Archives of Disease in Childhood	107	(Davenport L.; Rees D.)	A501-A502	10.1136/archdischild-2022-rcpch.807	Davenport 2022	Irrelevant	Excluded by TI/AB review

S4 Table. All studies identified in the literature search

3306	Developing a Longitudinal Cultural Competency Curriculum	Ghei, R.; Evans, M.; Chung, A.; Cardell, A.	2022	Western Journal of Emergency Medicine	23	4.1	S47	NA	Ghei 2022	Irrelevant	Excluded by TI/AB review
3307	A Novel Social Emergency Medicine Curriculum: An Alternative to Lecture- Based Didactics	Vuong, A.; Janeway, H.; Amen, A.; Fryling, L.; Garrett, A.; Jordan, J.; Wheaton, N.	2022	Western Journal of Emergency Medicine	23	4.1	S60	NA	Vuong 2022	Irrelevant	Excluded by TI/AB review

S4 Table. All studies identified in the literature search

3308	Emergency Department Quality of Care for Sickle Cell Disease in Ontario, Canada: [illegible]	Chan, D.C.H.; Kouyoumdjian, F.G.; Pang, A.J.; Niu, Y.; Athale, U.H.; Pendergrast, J.; Verhovsek, M.M.	2022	medRxiv	NA	(Chan D.C. H., derek. chan @med[illegible]	NA	10.1101/2022.07.06.22277294	Chan 2022	Irrelevant	Excluded by TI/AB review
3310	Care coordination for rural residents with chronic disease: Predictors of improved outcomes	Hansen, A.R.; McLendon, S.F.; Rochani, H.	2022	Public Health Nursing	39	4	760-769	10.1111/phn.13038	Hansen 2022	Irrelevant	Excluded by TI/AB review
3311	Transition for Adolescents and Young Adults with Sickle Cell Disease in a US Midwest Urban Center: A Multilevel Perspective on Barriers, Facilitators, and Future Directions	Calhoun, C.; Luo, L.; Baumann, A.A.; Bauer, A.; Shen, E.; McKay, V.; Hooley, C.; James, A.; King, A.A.	2022	Journal of Pediatric Hematology /Oncology	44	5	E872-E880	10.1097/MPH.0000000000002322	Calhoun 2022	Irrelevant	Excluded by TI/AB review

S4 Table. All studies identified in the literature search

3313	A national evaluation of emergency department thoracotomy practices: Will a high-risk, low-yield procedure reveal potential management practice bias?	Elkbuli, A.; Fanfan, D.; Newsome, K.; Sutherland, M.; Liu, H.; McKenney, M.; Ang, D.	2022	Surgery (United States)	172	1	410-420	10.1016/j.surg.2021.11.031	Elkbuli 2022	Irrelevant	Excluded by TI/AB review

S4 Table. All studies identified in the literature search

3314	Lost in Follow-Up: Predictors of Patient No-Shows to Clinic Follow-Up After Abdominal Injury	Hope Wilkinson, K.; Brandolino, A.; McCormick, A.; Deshpande, D.; Bergner, C.; Carver, T.; de Moya, M.; Milia, D.	2022	Journal of Surgical Research	275	(Hope Wilkinson K., katheryn.hope@gmail.com) Department of	15-Oct	10.1016/j.jss.2021.12.021	HopeWilkinson 2022	Irrelevant	Excluded by TI/AB review
3316	Profound Impact of Insurance Payor and Socioeconomic Status in Total Hip Arthroplasty Outcomes: Results From a High [illegible]	Koressel, J.E.; Perez, B.A.; DeAngelis, R.D.; Kerbel, Y.E.; Sheth, N.P.; Nelson, C.L.	2022	Journal of Arthroplasty	37	7	S434-S438	10.1016/j.arth.2022.03.018	Koressel 2022	Irrelevant	Excluded by TI/AB review
3317	Glaucoma Treatment Outcomes in Open Angle Glaucoma Patients of African Descent	Siesky, B.; Harris, A.; Belamkar, A.; Zukerman, R.; Horn, A.; Verticchio Vercellin, A.; Mendoza, K.A.; Sidoti, P.A.; Oddone, F.	2022	Journal of Glaucoma	31	7	479-487	10.1097/IJG.0000000000002027	Siesky 2022	Irrelevant	Excluded by TI/AB review

S4 Table. All studies identified in the literature search

3318	Predictors of Symptom Worsening or Improvement Using Remote Patient Reported Outcomes (PRO) Technology	Wujcik, D.; Dudley, W.	2022	Value in Health	25	7	S297	10.1016/j.jval.2022.04.064	Wujcik 2022	Irrelevant	Excluded by TI/AB review
3319	Evaluation of Cost and Health Utilization Outcomes of Medicare Beneficiaries Enrolled in a Medication Synchronization Program	Waghmare, P.; Huang, C.Y.; Green, W.M.; Jaynes, H.A.; Snyder, M.E.; Zillich, A.J.	2022	Value in Health	25	7	S302	10.1016/j.jval.2022.04.090	Waghmare 2022	Irrelevant	Excluded by TI/AB review
3320	Healthcare-seeking behaviour, barriers to care and predictors of symptom improvement among patients with [illegible]	Hertz, J.T.; Sakita, F.M.; Kweka, G.L.; Loring, Z.; Thielman, N.M.; Temu, G.; Bartlett, J.A.	2022	International Health	14	4	373-380	10.1093/inthealth/ihz095	Hertz 2022	Irrelevant	Excluded by TI/AB review

S4 Table. All studies identified in the literature search

3322	Improving Utilization of an After-Hours Phone Triage Service: A Resident Quality Improvement Initiative	Abraham, C.; Avis, E.; Caddle, S.; Lane, M.; Friedman, S.	2022	Quality management in health care	31	3	191-195	10.1097/QMH.0000000000000346	Abraham 2022	Irrelevant	Excluded by TI/AB review

S4 Table. All studies identified in the literature search

3323	The impact of community nursing program on healthcare utilization: A program evaluation	Xu, Y.; Koh, X.H.; Chua, Y.T.S.; Tan, C.G.I.; Aloweni, F.A.B.; Yap, B.E.J.; Tan, P.C.; Chua, X.; Lim, Y.K.S.; Oh, H.C.; Teo, S.H.S.; Lim, S.F.	2022	Geriatric nursing (New York, N.Y.)	46	(Xu Y.; Chua Y.T.S., stella.chua.y.t@sgh.com.sg; Tan	69-79	10.1016/j.gerinurse.2022.04.024	Xu 2022	Irrelevant	Excluded by TI/AB review
3324	Reducing Emergency Department Use for Ambulatory Oncology Patients Requiring Palliative Care: A Quality Improvement	Huynh, L.; Nolen, A.; Bonares, M.; Lau, C.; Dos Santos Sousa, S.; Freund, E.; Rapp, E.; Lau, F.	2022	Palliative Medicine	36	1 SUPPL	19	10.1177/02692163221093145	Huynh 2022	Irrelevant	Excluded by TI/AB review
3325	Meeting the Challenges of COVID-19: Evaluation of Nurse-Led Changes to Telephonic Assessment	Squeri, B.; Gayton, M.; Huang, J.; Chavez, S.; Souffront, K.	2022	Home healthcare now	40	4	214-222	10.1097/NHH.0000000000001081	Squeri 2022	Irrelevant	Excluded by TI/AB review

S4 Table. All studies identified in the literature search

3326	Evaluation of Clinician Adherence to M. genitalium Testing Guidance in Emergency Departments at an Academic Medical Center	Shen, V.; McCrate, B.; Reese, L.H.; Erickson, R.	2022	JACCP Journal of the American College of Clinical Pharmacy	5	7	752	10.1002/jac5.1661	Shen 2022	Irrelevant	Excluded by TI/AB review
3328	User preferences for and engagement with text messages to support antihypertensive medication adherence: Findings from a pilot study evaluating an emergency department-based behavioral	Nelson, L.A.; Spieker, A.J.; Kripalani, S.; Rothman, R.L.; Roumie, C.L.; Coco, J.; Fabbri, D.; Levy, P.; Collins, S.P.; McNaughton, C.D.	2022	Patient Education and Counseling	105	6	1606-1613	10.1016/j.pec.2021.10.011	Nelson 2022	Irrelevant	Excluded by TI/AB review

S4 Table. All studies identified in the literature search

3330	Changes in the prevalence and sociodemographic correlates of eating disorder symptoms from 2013 to 2020 among a large national sample of U.S. young adults: A repeated cross-sectional study	Romano, K.A.; Lipson, S.K.; Beccia, A.L.; Quatromoni, P.A.; Gordon, A.R.; Murgueitio, J.	2022	International Journal of Eating Disorders	55	6	776-789	10.1002/eat.23709	Romano 2022	Irrelevant	Excluded by TI/AB review
3331	The use of verbal de-escalation in intoxicated emergency department patients	Klein, L.R.; Driver, B.E.; Stang, J.; Ahmed, F.; Kim, E.; Carrabre, K.; Cole, J.B.; Miner, J.R.; Martel, M.	2022	American Journal of Emergency Medicine	56	(Klein L.R., laurenruthklein@gmail.com; [illegible]	348-350	10.1016/j.ajem.2021.09.029	Klein 2022	Irrelevant	Excluded by TI/AB review

S4 Table. All studies identified in the literature search

3332	Having pride in our care: Striving for equitable care in the emergency department for LGBTQIA patients	Harrison, C.; Meates, M.	2022	EMA - Emergency Medicine Australasia	34	3	442-445	10.1111/1742-6723.13991	Harrison 2022	Irrelevant	Excluded by TI/AB review

S4 Table. All studies identified in the literature search

3333	National Trends and Prescription Patterns in Opiate Analgesia for Urolithiasis Presenting to Emergency Departments: Analysis of the National Hospital Ambulatory Medical Care Survey, 2006-	Zhu, D.; Macdonald, E.J.; Lesko, R.P.; Watts, K.L.	2022	Urology	164	(Zhu D.; Macdonald E.J.; Lesko R.P.; Watts K.L., kwatts@montefiore.org)	80-87	10.1016/j.urology.2021.09.047	Zhu 2022	Irrelevant	Excluded by TI/AB review
3334	Neighborhood Socioeconomic Disadvantages Associated With Prolonged Lengths of Stay, Nonhome Discharges, and [illegible]	Khlopas, A.; Grits, D.; Sax, O.C.; Chen, Z.; Orr, M.N.; Klika, A.K.; Mont, M.A.; Piuzzi, N.S.	2022	Journal of Arthroplasty	37	6	S37-S43.e1	10.1016/j.arth.2022.01.032	Khlopas 2022	Irrelevant	Excluded by TI/AB review

S4 Table. All studies identified in the literature search

3335	Therapy outcome of day treatment for people with anorexia nervosa before and during the COVID-19 pandemic	Carr, A.; Toloza, C.; Li, Z.; Nazar, B.P.; Himmerich, H.	2022	Brain and Behavior	12	6	NA	10.1002/brb3.2604	Carr 2022	Irrelevant	Excluded by TI/AB review

S4 Table. All studies identified in the literature search

3337	Health care utilization among women of reproductive age living in public husing: Associations across six public housing sites in [illegible]	Headen, I.E.; Dubbin, L.; Canchola, A.J.; Kersten, E.; Yen, I.H.	2022	Preventive Medicine Reports	27	(Headen I.E., ieh27@drexel.edu) Drexel [illegible]	NA	10.1016/j.pmedr.2022.101797	Headen 2022	Irrelevant	Excluded by TI/AB review
3338	Racial disparities in presentation and short-term outcomes for patients with acute type B aortic dissection	Sorour, A.A.; Kirksey, L.; Laczynski, D.J.; Hoell, N.G.; Bena, J.; Kalahasti, V.; Roselli, E.E.; Smolock, C.J.; Lyden, S.P.; Caputo, F.J.	2022	Journal of Vascular Surgery	75	6	1855-1863.e2	10.1016/j.jvs.2022.01.010	Sorour 2022	Irrelevant	Excluded by TI/AB review
3339	A Pragmatic, Stepped-Wedge, Cluster-controlled Clinical Trial of Real-Time Pneumonia Clinical Decision [illegible]	Dean, N.C.; Vines, C.G.; Carr, J.R.; Rubin, J.G.; Webb, B.J.; Jacobs, J.R.; Butler, A.M.; Lee, J.; Jephson, A.R.; Jenson, N.; Walker, [illegible]	2022	American Journal of Respiratory and Critical Care Medicine	205	11	1330-1336	10.1164/rccm.202109-2092OC	Dean 2022	Irrelevant	Excluded by TI/AB review

S4 Table. All studies identified in the literature search

3340	Examining delays in diagnosis for slipped capital femoral epiphysis from a health disparities perspective	Purcell, M.; Reeves, R.; Mayfield, M.	2022	PLoS ONE	17	6-Jun	NA	10.1371/journal.pone.0269745	Purcell 2022	Irrelevant	Excluded by TI/AB review
3341	Urban air pollution and emergency department visits related to central nervous system diseases	Lukina, A.O.; Burstein, B.; Szyszkowicz, M.	2022	PLoS ONE	17	6-Jun	NA	10.1371/journal.pone.0270459	Lukina 2022	Irrelevant	Excluded by TI/AB review
3342	Potentially Inappropriate Prescribing in Hospitalized Older Adult High-Cost Health Care Users: A Pilot Study	Sanh, M.; Holbrook, A.; Macdonald, P.D.M.; Lee, J.	2022	Canadian Journal of Hospital Pharmacy	75	3	219-224	10.4212/cjhp.3122	Sanh 2022	Irrelevant	Excluded by TI/AB review

S4 Table. All studies identified in the literature search

3343	Exploring the health care utilization of children and youth in the care of child welfare: a retrospective matched cohort study	Breneol, S.; Rose, H.; Brown, M.; Andreou, P.; Bishop, A.; Mitchell, C.	2022	Family Practice	39	3	360-366	10.1093/fampra/cmab160	Breneol 2022	Irrelevant	Excluded by TI/AB review
3344	Association of Referral Source and Substance Use with Hepatitis C Virus Outcomes at a Southern [illegible]	Burton, H.J.; Khatiwada, A.; Chung, D.; Meissner, E.G.	2022	Southern Medical Journal	115	6	352-357	10.14423/SMJ.0000000000001402	Burton 2022	Irrelevant	Excluded by TI/AB review

S4 Table. All studies identified in the literature search

3347	P151: Disparities in elective spine surgery for Medicaid beneficiaries: A systematic review	Badin, D.; Ortiz-Babilonia, C.; Musharbash, F.; Jain, A.	2022	Global Spine Journal	12	3	283S-284S	10.1177/21925682221096075	Badin 2022	Irrelevant	Excluded by TI/AB review

S4 Table. All studies identified in the literature search

3348	ADAPTIVE INTERVENTIONS FOR ALCOHOL USE AND VIOLENCE AMONG YOUTH IN THE EMERGENCY DEPARTMENT: A SMART STUDY	Walton, M.A.; Seewald, L.; Kidwell, K.M.; Pearson, C.; Ngo, Q.; Blow, F.C.; Carter, P.	2022	Alcoholism: Clinical and Experimental Research	46	(Walton M.A.; Seewald L.; Kidwell K.M.; Pearson C.; Ngo Q.; Blow F.C.; Carter P.) University of Michigan, Ann Arbor, MI, United [illegible]	51A	10.1111/acer.14831	Walton 2022	Irrelevant	Excluded by TI/AB review

S4 Table. All studies identified in the literature search

3349	PALLIATIVE HOME CARE AND EMERGENCY DEPARTMENT VISITS IN THE LAST 30 AND 90 DAYS OF LIFE: A RETROSPECTIVE COHORT STUDY OF PATIENTS WITH CANCER	Mracek, J.; Earp, M.; Sinnarajah, A.	2022	Supportive Care in Cancer	30	(Mracek J.) University of Calgary, Canada(Earp M.) Department of Oncology, Cumming School of Medicine, University of Calgar[illegible]	S123-S125	10.1007/s00520-022-07099-y	Mracek 2022	Irrelevant	Excluded by TI/AB review

S4 Table. All studies identified in the literature search

3350	INNOVATIONS IN INTEGRATED CARE FOR INDIVIDUALS EXPERIENCING HOMELESSNESS	Mercer, T.; Correa, M.F.; Grove, L.; Miller, J.; Unroe, B.; Kaiser, K.; Benzer, J.K.	2022	Journal of General Internal Medicine	37	(Mercer T.; Grove L.) Population Health, University of Texas at Austin Dell Medical School, Austin, TX, United States (Corre[illegible]	S577-S578	10.1007/s11606-022-07653-8	Mercer 2022	Irrelevant	Excluded by TI/AB review

S4 Table. All studies identified in the literature search

3351	WE WANT TO HELP: INTEGRATING PRECLINICAL MEDICAL STUDENTS INTO THE HEALTH SYSTEM TO PROACTIVELY CONNECT PATIENTS TO RESOURCES	Hamzat, I.D.; Portieles Pena, R.; Nakshatri, S.; Fletcher, S.; Selkridge, I.; Parker, W.; Shah, S.D.; Volerman, A.	2022	Journal of General Internal Medicine	37	(Hamzat I.D.; Portieles Pena R.; Nakshatri S.; Fletcher S.; Selkridge I.) University of Chicago Pritzker, School of Medicine, Chicago	S602-S603	10.1007/s11606-022-07653-8	Hamzat 2022	Irrelevant	Excluded by TI/AB review

S4 Table. All studies identified in the literature search

3352	BRINGING HEALTHCARE TO PATIENTS' HOMES - DESCRIPTION OF A MOBILE INTEGRATED HEALTH PROGRAM IN DETROIT, MICHIGAN	Hagerman, T.K.; Derkowski, T.; Drake, S.M.; Miller, J.	2022	Journal of General Internal Medicine	37	(Hagerman T.K.) Departments of Emergency Medicine and Internal Medicine, Henry Ford Hospital, Detroit, MI, United States (Derk [illegible]	S561	10.1007/s11606-022-07653-8	Hagerman 2022	Irrelevant	Excluded by TI/AB review

S4 Table. All studies identified in the literature search

3353	WHEN POOR COMMUNICATION IS A QUESTION OF LIFE OR DEATH: A CASE OF HYPERKALEMIA DUE TO A MEDICATION ERROR DURING TRANSITIONS OF CARE	Degnall, E.; Schulson, L.	2022	Journal of General Internal Medicine	37	(Degnall E.) Department of Internal Medicine, Boston Medical Center, Boston, MA, United States (Schulson L.) Health Care [illegible]	S545	10.1007/s11606-022-07653-8	Degnall 2022	Irrelevant	Excluded by TI/AB review

S4 Table. All studies identified in the literature search

3354	PILOT PHASE REPORT OF A NOVEL MOBILE SUBSTANCE USE DISORDER TREATMENT PROGRAM IN AN ACADEMIC HEALTH SYSTEM	Walsh, J.P.	2022	Journal of General Internal Medicine	37	(Walsh J.P.) Medicine, University of Rochester Medical Center, Department of Medicine, Rochester, NY, United States	S586	10.1007/s11606-022-07653-8	Walsh 2022	Irrelevant	Excluded by TI/AB review

S4 Table. All studies identified in the literature search

3355	A NOVEL PHARMACY LIAISON-PATIENT NAVIGATOR INTERVENTION TO ADDRESS HEALTH-RELATED SOCIAL NEEDS AT AN URBAN SAFETY-NET HOSPITAL	Borden, C.G.; Ashe, E.M.; De La Vega, P.B.; Gast, V.; Saint-Phard, T.; Lasser, K.E.	2022	Journal of General Internal Medicine	37	(Borden C.G.; De La Vega P.B.; Gast V.; Saint-Phard T.; Lasser K.E.) General Internal Medicine, Boston Medical Center, [illegible]	S131-S132	10.1007/s11606-022-07653-8	Borden 2022	Irrelevant	Excluded by TI/AB review

S4 Table. All studies identified in the literature search

3356	READMISSION RATES AMONG PATIENTS AMONG PATIENTS EXPERIENCING HOMELESSNESS IN AN INTEGRATED HEALTHCARE SYSTEM	Yin, C.; Banerjee, S.; Dyer, W.T.; Schmittdiel, J.	2022	Journal of General Internal Medicine	37	(Yin C.; Banerjee S.) Graduate Medical Education, Kaiser Permanente Northern California, Oakland, CA, United States (Dyer W.T.; Schmi[illegible]	S190-S191	10.1007/s11606-022-07653-8	Yin 2022	Irrelevant	Excluded by TI/AB review

S4 Table. All studies identified in the literature search

3357	COST-RELATED RESIDENTIAL MOVES ARE ASSOCIATED WITH ADVERSE HEALTH OUTCOMES AND BEHAVIORS AMONG CALIFORNIA'S RENTERS	Chen, K.L.; Wisk, L.E.; Nuckols, T.K.; Ponce, N.A.; Choi, K.; Nau, C.L.; Zimmerman, F.J.	2022	Journal of General Internal Medicine	37	(Chen K.L.; Wisk L.E.) General Internal Medicine and Health Services Research, University of California, Los Angeles David Geffe[illegible]	S253	10.1007/s11606-022-07653-8	Chen 2022	Irrelevant	Excluded by TI/AB review

S4 Table. All studies identified in the literature search

3358	MEASURING CONTINUITY OF CARE FOR PATIENTSWITH DIABETES: WHICH TYPE OF PROVIDER VISITS TO INCLUDE?	Riggs, K.R.; Presley, C.A.; Agne, A.; Howell, C.R.; Huang, L.; Levitan, E.B.; Cherrington, A.	2022	Journal of General Internal Medicine	37	(Riggs K.R.) VA Clinic Birmingham, Birmingham, AL, United States (Huang L.) University of Alabama, Birmingham Schoo	S299	10.1007/s11606-022-07653-8	Riggs 2022	Irrelevant	Excluded by TI/AB review

S4 Table. All studies identified in the literature search

3359	HEALTH SERVICES UTILIZATION AMONG HOMELESSEXPERIENCED ADULTS WHO ENGAGED WITH A MOBILE ADDICTION PROGRAM	Fine, D.R.; Lewis, E.; Chang, Y.; Wright, J.; Gaeta, J.; Song, Z.; Baggett, T.P.	2022	Journal of General Internal Medicine	37	(Lewis E.; Wright J.; Gaeta J.; Baggett T.P.) Boston Health Care for the Homeless Program, Boston, MA, United States (Lewis E.; [illegible]	S278-S279	10.1007/s11606-022-07653-8	Fine 2022	Irrelevant	Excluded by TI/AB review

S4 Table. All studies identified in the literature search

3360	HOME HEALTH CARE, SELF-REPORTED GAPS IN CARE COORDINATION, AND SELF-REPORTED PREVENTABLE ADVERSE EVENTS AMONG OLDER ADULTS	Sterling, M.R.; Lau, J.D.; Rajan, M.; Akinyelure, O.P.; Safford, M.M.; Kern, L.M.	2022	Journal of General Internal Medicine	37	(Sterling M.R.; Lau J.D.; Rajan M.; Safford M.M.; Kern L.M.) Medicine, Weill Cornell Medicine, New	S280-S281	10.1007/s11606-022-07653-8	Sterling 2022	Irrelevant	Excluded by TI/AB review
3361	IMPACT OF STROKE SEVERITY AND PRIOR PSYCHOLOGICAL DISTRESS ON THREAT PERCEPTIONS IN THE [illegible]	Liyanage-Don, N.; Cornelius, T.; Jurado, A.; Chang, B.; Willey, J.; Kronish, I.	2022	Psychosomatic Medicine	84	5	A8	NA	Liyanage-Don 2022	Irrelevant	Excluded by TI/AB review

S4 Table. All studies identified in the literature search

3362	Racial disparities in surgical outcomes in patients with pancreatic ductal adenocarcinoma (PDAC) undergoing pancreas surgery	Saad, M.; Gartrelle, K.; Shah, R.A.; Clark, J.; Steffes, C.P.; Kwon, D.S.	2022	Journal of Clinical Oncology	40	16	NA	10.1200/JCO.2022.40.16_suppl.e16255	Saad 2022	Irrelevant	Excluded by TI/AB review
3363	Outcomes of patients with advanced urothelial cancer who develop infection while on treatment with pembrolizumab	Kieser, R.B.; Xu, J.; Burns, E.; Muhsen, I.; Shah, S.M.; Umoru, G.; Mylavarapu, C.; Sun, K.; Zhang, Y.; Crenshaw, A.; Esmail, A.; Guerrero, C.; Gong, Z.; Gee, K.; Heyne, K.; Singh, M.; Zhang, J.; Efstathiou, E.; Bernicker, E.; Abdelrahim, M.	2022	Journal of Clinical Oncology	40	16	NA	10.1200/JCO.2022.40.16_suppl.4573	Kieser 2022	Irrelevant	Excluded by TI/AB review

S4 Table. All studies identified in the literature search

3364	Association between remote monitoring and acute care visits in high-risk patients initiating intravenous antineoplastic therapy	Daly, R.M.; Nicholas, K.; Flynn, J.; Panageas, K.; Silva, N.; Duck, E.; Zervoudakis, A.; Holland, J.C.; Salvaggio, R.; Begue, A.; Wagner, I.; Sokolowski, S.; Zablocki, M.; Chiu, Y.O.; Kuperman, G.; Simon, B.A.; Perchick, W.; Reidy [illegible]	2022	Journal of Clinical Oncology	40	16	NA	10.1200/JCO.2022.40.16_suppl.1578	Daly 2022	Irrelevant	Excluded by TI/AB review
3365	Feasibility study of integrated ePROs in routine care during radiation therapy for head and neck cancer	Katzel, J.A.; VanDenEeden, S.K.; Liu, R.; Leimpeter, A.D.; Briones, C.B.; Lewis, D.L.; Lee-Enriquez, N.M.; Yan, J.M.; Altschuler, A.; Gillis, A.M.; Patel, [illegible]	2022	Journal of Clinical Oncology	40	16	NA	10.1200/JCO.2022.40.16_suppl.e13612	Katzel 2022	Irrelevant	Excluded by TI/AB review

S4 Table. All studies identified in the literature search

3366	Quality of end-of-life care for patients with multiple myeloma: A 12-year analysis of a population-based cohort	Mohyuddin, G.R.; Sinnarajah, A.; Gayowsky, A.; Chan, K.K.; Seow, H.; Mian, H.	2022	Journal of Clinical Oncology	40	16	NA	10.1200/JCO.2022.40.16_suppl.12031	Mohyuddin 2022	Irrelevant	Excluded by TI/AB review
3367	The effect of a multilevel community health worker-led intervention on health-related quality of life, patient activation, acute care use, and total costs of care: A randomized	Patel, M.I.; Kapphahn, K.; Salava, D.; Orchard, P.; Curry, R.; Filazzola, A.; Riley, A.; Krajcinovic, I.	2022	Journal of Clinical Oncology	40	16	NA	10.1200/JCO.2022.40.16_suppl.6500	Patel 2022	Irrelevant	Excluded by TI/AB review

S4 Table. All studies identified in the literature search

3368	Contemporary real-world associations between performance status and clinical outcomes in patients with cancer: A retrospective cohort study	Kumar, D.; Neeman, E.; Zhu, S.; Sun, H.; Kotak, D.; Liu, R.	2022	Journal of Clinical Oncology	40	16	NA	10.1200/JCO.2022.40.16_suppl.6578	Kumar 2022	Irrelevant	Excluded by TI/AB review

S4 Table. All studies identified in the literature search

3369	APN-Driven COVID-19 Monitoring Model for Kidney Transplant Patients	Yoo, J.; Patel, S.; Clevy-Schneller, T.; Desai, A.; Sodhi, R.; Jain, D.; Akkina, S.	2022	American Journal of Transplantation	22	(Yoo J.; Patel S.; Clevy-Schneller T.; Desai A.; Sodhi R.; Jain D.; Akkina S.) Loyola University Medical Center, Maywood, [illegible]	776	10.1111/ajt.17073	Yoo 2022	Irrelevant	Excluded by TI/AB review

S4 Table. All studies identified in the literature search

3370	Inequities in Glycemic Outcomes for Patients with Type 1 Diabetes: Six-Year (2016-2021) Longitudinal Follow-Up by [illegible]	Ebekozien, O.; Noor, N.; Kamboj, M.K.; Odugbesan, O.; Majidi, S.; Hopkins, R.; Dewit, E.L.; Izquierdo, R.; Agarwal, S.; Albanese-Oneill, A.; [illegible]	2022	Diabetes	71	(Ebekozien O.; Noor N.; Kamboj M.K.; [illegible]	NA	10.2337/db22-167-OR	Ebekozien 2022	Irrelevant	Excluded by TI/AB review
3371	Emergency Department Care Transition Programs - Value-Based Care Interventions That Need System-[illegible]	Biese, K.; Lash, T.A.; Kennedy, M.	2022	JAMA Network Open	5	5	E2213160	10.1001/jamanetworkopen.2022.13160	Biese 2022	Irrelevant	Excluded by TI/AB review
3372	Changing HCW attitudes: a case study of normalizing HIV service delivery in emergency departments	Rao, A.; Chen, V.H.; Hill, S.; Reynolds, S.J.; Redd, A.D.; Stead, D.; Hoffmann, C.; Quinn, T.C.; Hansoti, B.	2022	BMC health services research	22	1	629	10.1186/s12913-022-07942-2	Rao 2022	Irrelevant	Excluded by TI/AB review

S4 Table. All studies identified in the literature search

3373	Health literacy and long-term health outcomes following myocardial infarction: Protocol for a multicentre, [illegible]	Beauchamp, A.; Talevski, J.; Nicholls, S.J.; Wong Shee, A.; Martin, C.; Van Gaal, W.; Oqueli, E.; Ananthapavan, J.; Sharma, L.; O'Neil, A.; Brennan-Olsen, [illegible]	2022	BMJ Open	12	5	NA	10.1136/bmjopen-2021-060480	Beauchamp 2022	Irrelevant	Excluded by TI/AB review
3374	Relationships between erectile dysfunction, prostate cancer treatment type and inflatable penile [illegible]	Shen, C.; Jain, K.; Shah, T.; Schaefer, E.; Zhou, S.; Fried, D.; Helmer, D.A.; Sadeghi-Nejad, H.	2022	Investigative and Clinical Urology	63	3	316-324	10.4111/ICU.20210445	Shen 2022	Irrelevant	Excluded by TI/AB review

S4 Table. All studies identified in the literature search

3375	Orthopedic Specialty Hospital Reasons for Transfer and Subsequent Outcomes	D'Amore, T.; Blaber, O.; Magnuson, J.A.; Sutton, R.M.; Haag, T.; Krueger, C.A.	2022	Journal of Arthroplasty	37	5	819-823	10.1016/j.arth.2022.01.058	D'Amore 2022	Irrelevant	Excluded by TI/AB review

S4 Table. All studies identified in the literature search

3376	Disparities in cardiovascular outcomes among emergency department patients with mental illness	Kumar, S.; Duber, H.C.; Kreuter, W.; Sabbatini, A.K.	2022	American Journal of Emergency Medicine	55	(Kumar S., shilpak@uw.edu) University of Washington School of Medicine, Seattle, WA, United States (Duber H.C.; Sabbatini A.K.)	51-56	10.1016/j.ajem.2022.02.037	Kumar 2022	Irrelevant	Excluded by TI/AB review

S4 Table. All studies identified in the literature search

3377	The effects of acute pain on cognitive skills in emergency department patients	Marco, C.A.; Studebaker, H.; Harrington, M.; Ganz, E.; Boodt, B.; Hunt, T.; Costin, A.; Joseph, C.; Ely, I.	2022	American Journal of Emergency Medicine	55	(Marco C.A., Catherine.marco@wright.edu; Boodt B.; Hunt T.)	72-75	10.1016/j.ajem.2022.03.001	Marco 2022	Irrelevant	Excluded by TI/AB review
3378	Advance Care Planning for Emergency Department Patients with COVID-19 Infection: An Assessment of a Physician Training Program (QI411)	Markwalter, D.; Casey, M.; Price, L.; Bohrmann, T.; Tsujimoto, T.; Lavin, K.; Hanson, L.; Lin, F.-C.; Platts-Mills, T.	2022	Journal of Pain and Symptom Management	63	5	891	10.1016/j.jpainsymman.2022.02.098	Markwalter 2022	Irrelevant	Excluded by TI/AB review
3379	Intensity of End-of-Life Care and Palliative Care Utilization Among Older Hospitalized Patients with Limited English Proficiency: A [illegible]	Abedini, N.; Downey, L.; Engelberg, R.; Curtis, J.R.; Sharma, R.	2022	Journal of Pain and Symptom Management	63	5	863-864	10.1016/j.jpainsymman.2022.02.050	Abedini 2022	Irrelevant	Excluded by TI/AB review

S4 Table. All studies identified in the literature search

3380	Follow-up after post-partum psychiatric emergency department visits: an equity-focused population-based [illegible]	Barker, L.C.; Brown, H.K.; Bronskill, S.E.; Kurdyak, P.; Austin, P.C.; Hussain-Shamsy, N.; Fung, K.; Vigod, S.N.	2022	The Lancet Psychiatry	9	5	389-401	10.1016/S2215-0366(22)00099-2	Barker 2022	Irrelevant	Excluded by TI/AB review
3381	The Role of Posttraumatic Stress Symptoms on Pain Catastrophizing among Acutely Injured Patients	Pacella-LaBarbara, M.L.; Plaitano, E.; Suffoletto, B.; Kuhn, E.; Jaramillo, S.; Repine, M.; Germain, A.; Callaway, C.	2022	Journal of Pain	23	5	52-53	10.1016/j.jpain.2022.03.198	Pacella-LaBarbara 2022	Irrelevant	Excluded by TI/AB review

S4 Table. All studies identified in the literature search

3382	Comparing patient satisfaction in penile prosthesis surgery after radical prostatectomy versus the general population.	Howell, S.; Palasi, S.; Green, T.; Kannady, C.; Slaughter, K.; Wang, R.; Yang, B.	2022	Journal of Sexual Medicine	19	5	S192-S193	10.1016/j.jsxm.2022.03.439	Howell 2022	Irrelevant	Excluded by TI/AB review

S4 Table. All studies identified in the literature search

3384	Race/Ethnicity and Insurance's Impact on Delays to Kidney Stone Surgery Scheduling	Turner, E.N.; Talwar, R.; Familusi, O.O.; Michel, K.; Harris, J.E.L.; Ziemba, J.	2022	Urology	163	(Turner E.N., esther.nivasch@pennmedicine.upenn.edu; Talwar R.; Familusi O.O.; Michel K.;	196-201	10.1016/j.urology.2022.01.070	Turner 2022	Irrelevant	Excluded by TI/AB review
3385	SOCIOECONOMIC AND HEALTHCARE UTILIZATION FACTORS ASSOCIATED WITH EMERGENCY DEPARTMENT VISITS IN CROHN'S DISEASE PATIENTS- A US SINGLE CENTER EXPERIENCE	Howard, E.; Sylla, M.; Ommerborn, M.J.; Clark, C.R.; Silva, P.S.D.	2022	Gastroenterology	162	7	S-483	10.1016/S0016-5085(22)61146-6	Howard 2022	Irrelevant	Excluded by TI/AB review

S4 Table. All studies identified in the literature search

3386	CLINICAL CHARACTERISTICS AND LONG-TERM OUTCOMES IN PATIENTS WITH CYCLIC VOMITING SYNDROME: A 12-YEAR EXPERIENCE AT A TERTIARY REFERRAL CENTER	Partovi, O.; Patel, M.; Cheng, S.; Garacci, Z.; Venkatesan, T.	2022	Gastroenterology	162	7	S-69-S-70	10.1016/S0016-5085(22)60171-9	Partovi 2022	Irrelevant	Excluded by TI/AB review
3387	FACTORS ASSOCIATED WITH PROLONGED WAIT TIMES FOR GASTROINTESTINAL BLEEDING IN US EMERGENCY DEPARTMENTS: NATIONAL TRENDS DURING 2009	Meybodi, M.A.; Shaikh, A.; Hashemipour, R.; Ahlawat, S.	2022	Gastroenterology	162	7	S-272	10.1016/S0016-5085(22)60643-7	Meybodi 2022	Irrelevant	Excluded by TI/AB review

S4 Table. All studies identified in the literature search

3388	CLINICAL CHARACTERISTICS, OUTCOMES AND PREDICTORS OF DISABILITY IN CHRONIC PANCREATITIS: RESULTS FROM AN ACADEMIC PANCREAS	Cartelle, A.L.; Shah, I.; Bocchino, R.L.; Ahmed, A.; Freedman, S.D.; Kothari, D.; Sheth, S.G.	2022	Gastroenterology	162	7	S-566	10.1016/S0016-5085(22)61340-4	Cartelle 2022	Irrelevant	Excluded by TI/AB review

S4 Table. All studies identified in the literature search

3389	DISPARITIES IN HEALTHCARE UTILIZATION AND OUTCOMES IN PATIENTS WITH INFLAMMATORY BOWEL DISEASE	Zullow, S.; Sivasailam, B.; Galati, J.S.; Nakamura, T.; Malter, L.; Axelrad, J.E.	2022	Gastroenterology	162	7	S-484	10.1016/S0016-5085(22)61150-8	Zullow 2022	Irrelevant	Excluded by TI/AB review

S4 Table. All studies identified in the literature search

3390	EMTALA Violation? Examining Inequity in the Emergency Transfer of Psychiatric Patients	Tatum, J.; Ament, A.; Schmajuk, M.; Sanborn, K.; Maldonado, J.	2022	Journal of the Academy of Consultation-Liaison Psychiatry	63	(Tatum J.; Ament A.; Sanborn K.; Maldonado J.) Stanford University,(Schmajuk M.) Stanford University School of Medicine,	S96-S97	10.1016/j.jaclp.2022.03.199	Tatum 2022	Irrelevant	Excluded by TI/AB review

S4 Table. All studies identified in the literature search

3391	(PO-062) Collaborative Care in Acute Settings: An Integrated Approach to Psychiatric Care within an Academic Emergency Department	Owen, J.; Slocum, S.; Ray, J.; Zosel, A.; Hernandez-Meier, J.; Martin, I.	2022	Journal of the Academy of Consultation-Liaison Psychiatry	63	(Owen J.; Ray J.; Zosel A.; Hernandez-Meier J.; Martin I.) Medical College of Wisconsin,(Slocum S.) Geisel Schoo	S30-S31	10.1016/j.jaclp.2022.03.067	Owen 2022	Irrelevant	Excluded by TI/AB review
3392	(PO-168) Culture Bound Syndrome or Idiom of Distress? A Case of Brief Jinn Possession with Atypical Features in Afghani Patient	Cote, A.; Mukherji, S.	2022	Journal of the Academy of Consultation-Liaison Psychiatry	63	(Cote A.) University of Washington, (Mukherji [illegible]	S77	10.1016/j.jaclp.2022.03.160	Cote 2022	Irrelevant	Excluded by TI/AB review

S4 Table. All studies identified in the literature search

3393	Integrative Approaches to Pediatric Chronic Pain in an Urban Safety-Net Hospital: Cost Savings, Clinical Benefits, and Safety	Sommers, E.; D'Amico, S.; Goldstein, L.; Gardiner, P.	2022	Journal of Integrative and Complementary Medicine	28	5	445-453	10.1089/jicm.2021.0296	Sommers 2022	Irrelevant	Excluded by TI/AB review
3394	A Primary Care Telehealth Pilot Program to Improve Access: Associations with Patients' Health Care Utilization and Costs	Gujral, K.; Scott, J.Y.; Ambady, L.; Dismuke-Greer, C.E.; Jacobs, J.; Chow, A.; Oh, A.; Yoon, J.	2022	Telemedicine journal and e-health : the official journal of the American Telemedicine Association	28	5	643-653	10.1089/tmj.2021.0284	Gujral 2022	Irrelevant	Excluded by TI/AB review
3395	Use of Neuroimaging for Patients with Dizziness Who Present to Outpatient Clinics [illegible]	Adams, M.E.; Karaca-Mandic, P.; Marmor, S.	2022	JAMA Otolaryngology - Head and Neck Surgery	148	5	465-473	10.1001/jamaoto.2022.0329	Adams 2022	Irrelevant	Excluded by TI/AB review

S4 Table. All studies identified in the literature search

3396	Food Insecurity does not Increase Peri-Operative Complications after Bariatric Surgery	Mathson, L.; Kindel, T.; Lak, K.; Gould, J.; Higgins, R.M.; Alter-Troilo, K.	2022	Surgical Endoscopy	36	1 SUPPL	S92	10.1007/s00464-022-09220-y	Mathson 2022	Irrelevant	Excluded by TI/AB review
3397	Impact of Outpatient Follow-Up on Likelihood of Emergency Department Revisit Within 30 Days Following Index Visit for Acute Asthma Exacerbation	Abbott, E.; Karwoska Kligler, S.; Spadafore, S.; Vargas-Torres, C.; Lin, M.	2022	American Journal of Respiratory and Critical Care Medicine	205	1	NA	10.1164/ajrccm-conference.2022.205.1_MeetingAbstracts.A5095	Abbott 2022	Irrelevant	Excluded by TI/AB review
3398	Differences in Treatment Decision-Making and Use Among White and Black Individuals with Asthma: Results [illegible]	Nyenhuis, S.M.; Foggs, M.B.; Winders, T.A.; Gardner, D.D.; Nelson, M.; Balmer, J.; Carter, J.; Simone, L.	2022	American Journal of Respiratory and Critical Care Medicine	205	1	NA	10.1164/ajrccm-conference.2022.205.1_MeetingAbstracts.A3496	Nyenhuis 2022	Irrelevant	Excluded by TI/AB review

S4 Table. All studies identified in the literature search

3399	The Impact of Air Pollution on Global DNA Methylation in Asthmatic Patients Living in Western Pennsylvania	Macedonia, T.V.; Tang, W.; Byrwa-Hill, B.M.; Wenzel, S.E.	2022	American Journal of Respiratory and Critical Care Medicine	205	1	NA	10.1164/ajrccm-conference.2022.205.1_MeetingAbstracts.A3574	Macedonia 2022	Irrelevant	Excluded by TI/AB review
3400	Sex-Specific Differences in L-Arginine Metabolism of Asthma Patients	Althoff, M.D.; Sharma, S.; Grasemann, H.; Federman, A.; Wisnivesky, J.P.; Holguin, F.	2022	American Journal of Respiratory and Critical Care Medicine	205	1	NA	10.1164/ajrccm-conference.2022.205.1_MeetingAbstracts.A1248	Althoff 2022	Irrelevant	Excluded by TI/AB review
3401	Using Qualitative Methods to Assess Patient-Reported Outcome Measures of Dyspnea and Self-Efficacy Among Adult Emergency [illegible]	Lin, M.P.; Ramirez, M.; Teresi, J.; Richardson, L.	2022	American Journal of Respiratory and Critical Care Medicine	205	1	NA	10.1164/ajrccm-conference.2022.205.1_MeetingAbstracts.A1322	Lin 2022	Irrelevant	Excluded by TI/AB review

S4 Table. All studies identified in the literature search

3402	Quantifying the Economic Burden of Hospitalization from Neuromyelitis Optica: Results of a Cross-sectional Survey	Rice, D.; Holroyd, K.; Kei Pua, D.; Levy, M.; Mateen, F.; Bhattacharyya, S.	2022	Neurology	98	18 SUPPL	NA	NA	Rice 2022	Irrelevant	Excluded by TI/AB review
3403	Patterns of Seeking Emergent Stroke Care During the COVID-19 Pandemic: A Nebraska Stroke Center Perspective	Dibs, M.; Faulkner, M.; Sohail, A.	2022	Neurology	98	18 SUPPL	NA	NA	Dibs 2022	Irrelevant	Excluded by TI/AB review

S4 Table. All studies identified in the literature search

3404	Hispanics and Chest Pain: An Opportunity to Address Healthcare Disparities in A Residency Training Program	Jimenez, K.P.; Burgess, K.; Joung, Y.; Nyange, C.; Charaf, C.; Chang, E.Y.	2022	Circulation: Cardiovascular Quality and Outcomes	15	(Jimenez K.P.; Burgess K.; Joung Y.; Nyange C.; Charaf C.; Chang E.Y.)	NA	10.1161/circoutcomes.15.suppl_1.225	Jimenez 2022	Irrelevant	Excluded by TI/AB review
3405	12-month Clinical Outcomes among Patients with Heart Failure and Reduced to Preserved Ejection Fraction Based on Sacubitril/valsartan [illegible]	Albert, N.M.; Bena, J.F.; Morrison, S.L.; Bradley Williams, J.; Faulkenberg, K.; Levay, M.; Shen, X.	2022	Circulation: Cardiovascular Quality and Outcomes	15	(Albert N.M.; Bena J.F.; Morrison S.L.; [illegible]	NA	10.1161/circoutcomes.15.suppl_1.34	Albert 2022	Irrelevant	Excluded by TI/AB review

S4 Table. All studies identified in the literature search

3406	Practice Variation in Use of Neuroimaging Among Infants With Concern for Abuse Treated in Children's Hospitals	Henry, M.K.; Schilling, S.; Shults, J.; Feudtner, C.; Katcoff, H.; Egbe, T.I.; Johnson, M.A.; Andronikou, S.; Wood, J.N.	2022	JAMA Network Open	5	4	E225005	10.1001/jamanetworkopen.2022.5005	Henry 2022	Irrelevant	Excluded by TI/AB review
3407	US emergency care patterns among nurse practitioners and physician assistants [illegible]	Mafi, J.N.; Chen, A.; Guo, R.; Choi, K.; Smulowitz, P.; Tseng, C.-H.; Ladapo, J.A.; Landon, B.E.	2022	BMJ Open	12	4	NA	10.1136/bmjopen-2021-055138	Mafi 2022	Irrelevant	Excluded by TI/AB review

S4 Table. All studies identified in the literature search

3408	Systematic review of first responder post-deployment or post-incident psychosocial interventions	Tan, L.; Petrie, K.; Deady, M.; Bryant, R.A.; Harvey, S.B.	2022	Occupational medicine (Oxford, England)	72	3	160-169	10.1093/occmed/kqab182	Tan 2022	Irrelevant	Excluded by TI/AB review

S4 Table. All studies identified in the literature search

3409	In-Hospital Cardiac Arrest in United States Emergency Departments, 2010,Äì2018	Sung, C.-W.; Lu, T.-C.; Wang, C.-H.; Chou, E.H.; Ko, C.-H.; Huang, C.-H.; Chen, W.-J.; Tsai, C.-L.	2022	Frontiers in Cardiovascular Medicine	9	(Sung C.-W.) Department of Emergency Medicine, National Taiwan University Hospital Hsin-Chu Branch, Hsinchu, Taiwan(Lu [illegible]	NA	10.3389/fcvm.2022.874461	Sung 2022	Irrelevant	Excluded by TI/AB review

S4 Table. All studies identified in the literature search

3410	Visualising Emergency Department Wait Times; Rapid Iterative Testing to Determine Patient Preferences for Displays	Walker, K.; Potter, E.; Hwang, I.; Dwyer, T.; Egerton-Warburton, D.; Joe, K.; Hutton, J.; Freeman, S.; Flynn, D.	2022	medRxiv	NA	(Walker K., katie_walker01@yahoo.com.au) Casey Hospital, Mona	NA	10.1101/2022.03.30.22273211	Walker 2022	Irrelevant	Excluded by TI/AB review
3411	The Impact of an Integrated Care Management Program on Acute Care Use and Outpatient Appointment Attendance Among High-Risk Patients With Lupus	Williams, J.N.; Taber, K.; Huang, W.; Collins, J.; Cunningham, R.; McLaughlin, K.; Vogeli, C.; Wichmann, L.; Feldman, C.H.	2022	ACR Open Rheumatology	4	4	338-344	10.1002/acr2.11391	Williams 2022	Irrelevant	Excluded by TI/AB review

S4 Table. All studies identified in the literature search

3412	Exploring the perspectives of healthcare providers that care for non-insured individuals utilizing emergency departments in Toronto	Siu, C.; Rao, S.; Hayman, K.; Hulme, J.; Gajaria, A.	2022	Canadian Journal of Emergency Medicine	24	3	283-287	10.1007/s43678-022-00263-7	Siu 2022	Irrelevant	Excluded by TI/AB review
3413	Patients From Medically Underserved Areas Are at Increased Risk for Nonhome Discharge and Emergency Department Return After Total Joint Arthroplasty	Holbert, S.E.; Cheema, M.; Brennan, J.C.; MacDonald, J.H.; King, P.J.; Turcotte, J.J.	2022	Journal of Arthroplasty	37	4	609-615	10.1016/j.arth.2021.12.033	Holbert 2022	Irrelevant	Excluded by TI/AB review
3415	Changes in Emergency Department Practice of Adolescent Abnormal Uterine Bleeding after [illegible]	Isaacson, E.; Isa, R.; Monge, M.; Salazar, C.	2022	Journal of Pediatric and Adolescent Gynecology	35	2	199-200	10.1016/j.jpag.2022.01.019	Isaacson 2022	Irrelevant	Excluded by TI/AB review

S4 Table. All studies identified in the literature search

3416	Disruption of Pediatric Emergency Department Use during the COVID-19 Pandemic	Sen, B.; Brisendine, A.E.; Ghosh, P.	2022	Southern Medical Journal	115	4	250-255	10.14423/SMJ.0000000000001383	Sen 2022	Irrelevant	Excluded by TI/AB review
3417	Emergency Department/Urgent Care as Usual Source of Care and Clinical Outcomes in CKD: Findings [illegible]	Toth-Manikowski, S.M.; Hsu, J.Y.; Fischer, M.J.; Cohen, J.B.; Lora, C.M.; Tan, T.C.; He, J.; Greer, R.C.; Weir, M.R.; Zhang, X.; [illegible]	2022	Kidney Medicine	4	4	NA	10.1016/j.xkme.2022.100424	Toth-Manikowski 2022	Irrelevant	Excluded by TI/AB review
3418	Contemporary Evaluation of Gender, Race, and Socioeconomics with Outcomes in Heart Failure	Mizrahi, I.; Shah, P.; Lum, C.; Khan, Z.; Huang, R.; Nagamine, T.; Lee, D.; Shimabuku, L.; Shiraishi, K.; [illegible]	2022	Journal of Heart and Lung Transplantation	41	4	S217	10.1016/j.healun.2022.01.1681	Mizrahi 2022	Irrelevant	Excluded by TI/AB review

S4 Table. All studies identified in the literature search

3419	Ethnic Disparities in Penile Prosthesis Surgery: An Analysis of Hispanic Patients	Suarez-Sarmiento, A.; Perito, P.; Fakhoury, M.; Levitt, B.	2022	Journal of Sexual Medicine	19	4	S13-S14	10.1016/j.jsxm.2022.01.036	Suarez-Sarmiento 2022	Irrelevant	Excluded by TI/AB review
3421	Factors Associated With Inflatable Penile Prosthesis Underutilization After Prostate Cancer Treatment	Abdelmalek, G.; Warren, C.; Shen, C.; Shah, T.; Lulla, T.; Schaefer, E.; Zhou, S.; Fried, D.; Helemer, D.; Sadeghi-Nejad, H.	2022	Journal of Sexual Medicine	19	4	S62-S63	10.1016/j.jsxm.2022.01.385	Abdelmalek 2022	Irrelevant	Excluded by TI/AB review
3423	Racial Disparities in Sexual Health Care Received by Kidney Transplant Recipients in a Metropolitan Area	Santamaria, A.; Yoffe, D.A.; Modiri, N.; Shahinyan, R.; Jayadevan, R.; Islam, M.; Eleswarapu, S.; Mills, J.	2022	Journal of Sexual Medicine	19	4	S77	10.1016/j.jsxm.2022.01.412	Santamaria 2022	Irrelevant	Excluded by TI/AB review

S4 Table. All studies identified in the literature search

3425	Shifting Away From Emergency Department and Office-Based Urgent Care: No Place Like Home?	Solomon, N.A.; Fendrick, A.M.	2022	American Journal of Managed Care	28	4	145-146	10.37765/ajmc.2022.88857	Solomon 2022	Irrelevant	Excluded by TI/AB review

S4 Table. All studies identified in the literature search

3426	"It closes the gap when the ball is dropped": patient perspectives of a novel smartphone app for regional care coordination after hospital encounters	Guzman, A.; Brown, T.; Liss, D.T.	2022	mHealth	8	(Guzman A., adriana.guzman@northwestern.edu; Brown T.; Liss D.T.) Division of General Internal Medicine and Geriatrics, Northwester[illegible]	NA	10.21037/mhealth-21-49	Guzman 2022	Irrelevant	Excluded by TI/AB review

S4 Table. All studies identified in the literature search

3427	IDF21-0133 Improved Diabetes Outcomes & Decreased Complications Since 2007 By A Primary Healthcare Team In Chilliwack BC Canada	Leong, W.; Sloan, P.; Smith, Q.A.; Ross, C.C.; Page, D.A.; Voth, B.R.; Viljoen, L.J.	2022	Diabetes Research and Clinical Practice	186	(Leong W.) BC Provincial Diabetes Evaluation, Knowledge Translation Phase, Vancouver, Canada(Sloan P.; Smith Q.A.; Ross C.C.; Page	NA	10.1016/j.diabres.2022.109714	Leong 2022	Irrelevant	Excluded by TI/AB review

S4 Table. All studies identified in the literature search

3428	Troponin is unrelated to outcomes in heart failure patients discharged from the emergency department	Fermann, G.J.; Schrock, J.W.; Levy, P.D.; Pang, P.; Butler, J.; Chang, A.M.; Char, D.; Diercks, D.; Han, J.H.; Hiestand, B.; Hogan, C.; Jenkins, C.A.; Kampe, C.; Khan, Y.; Kumar, V.A.; Lee, S.; Lindenfeld, J.; Liu, D.; Miller, K.F.; Peacock, W.F.; Reilly, C.M.; Robichaux, C.; Rothman, R.L.; Self, W.H.; Singer, A.J.; Sterling, S.A.; Storrow, A.B.; Stubblefield, W.B.; Walsh, C.; Wilburn, J.; Collins, S.P.	2022	JACEP Open	3	2	NA	10.1002/emp2.12695	Fermann 2022	Irrelevant	Excluded by TI/AB review

S4 Table. All studies identified in the literature search

3431	Emergency department presentations in palliative care patients: a retrospective cohort study	Taylor, P.; Stone, T.; Simpson, R.; Kyeremateng, S.; Mason, S.	2022	BMJ supportive & palliative care	NA	(Taylor P., drpaulmtaylor@gmail.com; Stone T.; Simpson R.; Maso	NA	10.1136/bmjspcare-2022-003563	Taylor 2022	Irrelevant	Excluded by TI/AB review
3432	TWO YEAR EXPERIENCE WITH HIGH-SENSITIVITY TROPONIN TESTING:INTERPRETATIVE VALUES FOR IMPROVING PATIENT DISPOSITION	Tanaka, J.; Patolia, H.; Lobaugh, B.; Vleet, C.V.; Klem, I.; Borawski, J.; Doshi, P.; Lowenstern, A.; Newby, L.K.; Tcheng, J.E.; Rymer, J.	2022	Journal of the American College of Cardiology	79	9	982	10.1016/S0735-1097(22)01973-8	Tanaka 2022	Irrelevant	Excluded by TI/AB review

S4 Table. All studies identified in the literature search

3433	RACE-IT-RAPID MYOCARDIAL INFARCTION EXCLUSION USING AN ACCELERATED HIGH-SENSITIVITY CARDIAC TROPONIN I PROTOCOL: A PROSPECTIVE TRIAL	McCord, J.K.; Cook, B.; Gandolfo, C.; Parikh, S.; Klausner, H.; Abdul-Nour, K.; Lewandowski, A.; Hudson, M.P.; Perrotta, G.S.; Zweig, B.; Gunaga, S.; Lanfear, D.E.; Gindi, R.; Levy, P.D.; Mills, N.L.; Mahler, S.; Kim, H.E.; Danagoulian, S.; Tang, A.; Nassereddine, H.; Oudeif, A.; Malette, K.; Krupp, S.; Keerie, C.; Miller, J.	2022	Journal of the American College of Cardiology	79	9	951	10.1016/S0735-1097(22)01942-8	McCord 2022	Irrelevant	Excluded by TI/AB review
3434	A RANDOMIZED CONTROLLED TRIAL OF CLINICAL OUTCOMES AFTER VIEWING VIDEO EDUCATION ON DANGER [illegible]	Albert, N.M.; Babcock, C.; Gray-Leyko, A.M.; Bena, J.F.; Morrison, S.L.; Sayler, D.; Levay, M.	2022	Journal of the American College of Cardiology	79	9	394	10.1016/S0735-1097(22)01385-7	Albert 2022	Irrelevant	Excluded by TI/AB review

S4 Table. All studies identified in the literature search

3435	Risk of eating disorders in a representative sample of Italian adolescents: prevalence and association with self-reported [illegible]	D,ÄôAnna, G.; Lazzeretti, M.; Castellini, G.; Ricca, V.; Cassioli, E.; Rossi, E.; Silvestri, C.; Voller, F.	2022	Eating and Weight Disorders	27	2	701-708	10.1007/s40519-021-01214-4	D,ÄôAnna 2022	Irrelevant	Excluded by TI/AB review
3436	Caregiver Depressive Symptoms and Primary Medication Nonadherence in Children With [illegible]	Margolis, R.H.F.; Bellin, M.H.; Morphew, T.; Tsoukleris, M.; Bollinger, M.E.; Butz, A.	2022	Journal of Pediatric Health Care	36	2	136-143	10.1016/j.pedhc.2021.01.005	Margolis 2022	Irrelevant	Excluded by TI/AB review
3437	Involuntary Psychiatric Hospitalization: How Patient Characteristics Affect Decision-Making	Bhalla, I.P.; Siegel, K.; Chaudhry, M.; Li, N.; Torbati, S.; Nuckols, T.; Danovitch, I.	2022	Psychiatric Quarterly	93	1	297-310	10.1007/s11126-021-09939-2	Bhalla 2022	Irrelevant	Excluded by TI/AB review

S4 Table. All studies identified in the literature search

3438	Association of Race and Neighborhood Disadvantage with Patient Engagement in a Home-Based [illegible]	Fritz, B.A.; Ramsey, B.; Taylor, D.; Shoup, J.P.; Schmidt, J.M.; Guinn, M.; Maddox, T.M.	2022	Journal of General Internal Medicine	37	4	838-846	10.1007/s11606-021-07207-4	Fritz 2022	Irrelevant	Excluded by TI/AB review
3439	Impact of Social Determinants on the Burden of Asthma and Eczema: Results from a US Patient Survey	Bukstein, D.A.; Friedman, A.; Gonzalez Reyes, E.; Hart, M.; Jones, B.L.; Winders, T.	2022	Advances in Therapy	39	3	1341-1358	10.1007/s12325-021-02021-0	Bukstein 2022	Irrelevant	Excluded by TI/AB review
3440	Executive summary of the 2021 SAEM consensus conference: From bedside to policy: Advancing social emergency medicine and [illegible]	Schoenfeld, E.M.; Lin, M.P.; Samuels-Kalow, M.E.	2022	Academic Emergency Medicine	29	3	354-363	10.1111/acem.14451	Schoenfeld 2022	Irrelevant	Excluded by TI/AB review

S4 Table. All studies identified in the literature search

3441	Improved asthma outcomes among at-risk children in a pharmacist-led, interdisciplinary school-based health clinic: A pilot study of the [illegible]	Elliott, J.P.; Morphew, T.; Gentile, D.; Williams, P.; Barrett, C.; Sossong, N.	2022	Journal of the American Pharmacists Association	62	2	519-525.e1	10.1016/j.japh.2021.11.008	Elliott 2022	Irrelevant	Excluded by TI/AB review

S4 Table. All studies identified in the literature search

3442	Changes in Rates and Modality of Interpreter Use for Pediatric Emergency Department Patients in the COVID-19 Era	Hartford, E.A.; Carlin, K.; Rutman, L.E.; Lion, K.C.	2022	Joint Commission Journal on Quality and Patient Safety	48	3	139-146	10.1016/j.jcjq.2021.11.003	Hartford 2022	Irrelevant	Excluded by TI/AB review
3443	Associated factors, assessment, management, and outcomes of patients who present to the emergency [illegible]	Phillips, T.M.; Moloney, C.; Sneath, E.; Beccaria, G.; Issac, H.; Mullens, A.B.; Gow, J.; Rana, R.; King, A.	2022	Respiratory Medicine	193	(Phillips T.M., Tania.Phillips@usq.edu.	NA	10.1016/j.rmed.2022.106747	Phillips 2022	Irrelevant	Excluded by TI/AB review

S4 Table. All studies identified in the literature search

3444	Outcomes of Incidental Lung Nodules With Structured Recommendations and Electronic Tracking	Bagga, B.; Fansiwala, K.; Thomas, S.; Chung, R.; Moore, W.H.; Babb, J.S.; Horwitz, L.I.; Blecker, S.; Kang, S.K.	2022	Journal of the American College of Radiology	19	3	407-414	10.1016/j.jacr.2021.09.046	Bagga 2022	Irrelevant	Excluded by TI/AB review
3445	Outcomes of a controlled trial with visiting therapy dog teams on pain in adults in an emergency department	Carey, B.; Dell, C.A.; Stempien, J.; Tupper, S.; Rohr, B.; Carr, E.; Cruz, M.; Acoose, S.; Butt, P.; Broberg, L.; Collard, L.; Fele-Slaferek, L.; Fornssler, C.; Goodridge, D.; Gunderson, J.; McKenzie, H.;	2022	PLoS ONE	17	3-Mar	NA	10.1371/journal.pone.0262599	Carey 2022	Irrelevant	Excluded by TI/AB review

S4 Table. All studies identified in the literature search

3446	Access to domestic violence advocacy by race, ethnicity and gender: The impact of a digital warm handoff from the emergency [illegible]	Brignone, L.; Gomez, A.M.	2022	PLoS ONE	17	3-Mar	NA	10.1371/journal.pone.0264814	Brignone 2022	Irrelevant	Excluded by TI/AB review
3447	Perspectives of individuals with sickle cell disease on barriers to care	Phillips, S.; Chen, Y.; Masese, R.; Noisette, L.; Jordan, K.; Jacobs, S.; Hsu, L.L.; Melvin, C.L.; Treadwell, M.; Shah, N.; Tanabe, P.; [illegible]	2022	PLoS ONE	17	3-Mar	NA	10.1371/journal.pone.0265342	Phillips 2022	Irrelevant	Excluded by TI/AB review
3449	Acceptability of HIV Testing for Adolescents and young Adults by Delivery Model: A Systematic Review	Leistikow, P.T.; Patel, V.; Nouryan, C.; Cervia, J.S.	2022	Journal of Investigative Medicine	70	3	829-836	10.1136/jim-2021-002056	Leistikow 2022	Irrelevant	Excluded by TI/AB review

S4 Table. All studies identified in the literature search

3450	Ileostomy Patients Using Patient Engagement Technology Experience Decreased Length of Stay	Smithson, M.; McLeod, M.C.; Theiss, L.; Shao, C.; Kennedy, G.; Hollis, R.; Chu, D.I.; Hardiman, K.M.	2022	Journal of gastrointestinal surgery : official journal of the Society for Surgery of the Alimentary Tract	26	3	635-642	10.1007/s11605-021-05158-z	Smithson 2022	Irrelevant	Excluded by TI/AB review

S4 Table. All studies identified in the literature search

3451	Social and structural determinants and their associations with patient experience in the emergency department	Unger, S.; Orr, Z.; Avraham Alpert, E.; Davidovitch, N.; Shoham-Vardi, I.	2022	International emergency nursing	61	(Unger S., shifrag@g.jct.ac.il) Faculty of Life and Health Sciences, Jerusalem College of Technology, Jerusalem, Israel; Faculty of Health Scienc[illegible]	101131	10.1016/j.ienj.2021.101131	Unger 2022	Irrelevant	Excluded by TI/AB review

S4 Table. All studies identified in the literature search

3452	COVID infection rates, clinical outcomes, and racial/ethnic and gender disparities before and after Omicron emerged in the US	Wang, L.; Berger, N.A.; Kaelber, D.C.; Davis, P.B.; Volkow, N.D.; Xu, R.	2022	medRxiv	NA	(Wang L.; Xu R., rxx@case.edu) Center for Artificial Intelligence in Drug Discovery, Case Western Reserve University School of [illegible]	NA	10.1101/2022.02.21.22271300	Wang 2022	Irrelevant	Excluded by TI/AB review

S4 Table. All studies identified in the literature search

3453	Human Trafficking ICD-10 Code Utilization in Pediatric Tertiary Care Centers Within the United States	Garg, A.; Panda, P.; Malay, S.; Slain, K.N.	2022	Frontiers in Pediatrics	10	(Garg A., anjaliga07@gmail.com) Department [illegible]	NA	10.3389/fped.2022.818043	Garg 2022	Irrelevant	Excluded by TI/AB review
3454	Association between Neighborhood-Level Social Determinants of Health and Access to Pediatric [illegible]	Bouchard, M.E.; Kan, K.; Tian, Y.; Casale, M.; Smith, T.; De Boer, C.; Linton, S.; Abdullah, F.; Ghomrawi, H.M.K.	2022	JAMA Network Open	5	2	NA	10.1001/jamanetworkopen.2021.48865	Bouchard 2022	Irrelevant	Excluded by TI/AB review
3455	Gender Differences in Patients,Äô Experience of Care in the Emergency Department	Chen, P.G.; Tolpadi, A.; Elliott, M.N.; Hays, R.D.; Lehrman, W.G.; Stark, D.S.; Parast, L.	2022	Journal of General Internal Medicine	37	3	676-679	10.1007/s11606-021-06862-x	Chen 2022	Irrelevant	Excluded by TI/AB review

S4 Table. All studies identified in the literature search

3458	Revisiting racial disparities in ED CT utilization during the Affordable Care Act era: 2009,Äì2018 data from the NHAMCS	Al-Dulaimi, R.; Duong, P.-A.; Chan, B.Y.; Fuller, M.J.; Ross, A.B.; Dunn, D.P.	2022	Emergency Radiology	29	1	125-132	10.1007/s10140-021-01991-6	Al-Dulaimi 2022	Irrelevant	Excluded by TI/AB review

S4 Table. All studies identified in the literature search

3460	Evaluation of advanced practice provider related return visits to a pediatric emergency department and their outcomes	Hussain, B.; Kannikeswaran, N.; Mathew, R.; Arora, R.	2022	American Journal of Emergency Medicine	52	(Hussain B., bhussain@dmc.org) Pediatric Emergency Medicine Fellow, Children's	174-178	10.1016/j.ajem.2021.11.040	Hussain 2022	Irrelevant	Excluded by TI/AB review
3461	Patient Use Of Non-Standard Terms For Asthma Inhalers Is Associated With Worse Asthma Morbidity Outcomes	Forth, V.; Hernandez, P.A.; Carroll, J.; Ericson, B.; Cui, J.; Fagan, M.; Fuhlbrigge, A.; Hurley, L.; Kruse, J.; Maher, N.; Manning, B.; Mosnaim, G.; Pace, W.; Phipatanakul, W.; Rodriguez Louis, J.;	2022	Journal of Allergy and Clinical Immunology	149	2	AB88	10.1016/j.jaci.2021.12.313	Forth 2022	Irrelevant	Excluded by TI/AB review

S4 Table. All studies identified in the literature search

3462	Urticaria and/or angioedema events secondary to mRNA COVID-19 vaccinations - Updates from a national case registry	Anvari, S.; Samarakoon, U.; Fu, X.; Jaggers, J.; Gonzalez-Estrada, A.; Chong, H.J.; Van Meerbeke, S.; Petrov, A.; Milkovich, L.; Harigan, E.; Khan, D.; Blumenthal, K.	2022	Journal of Allergy and Clinical Immunology	149	2	AB314	10.1016/j.jaci.2021.12.006	Anvari 2022	Irrelevant	Excluded by TI/AB review
3463	Antibiotic expectations of patients attending an emergency department with upper respiratory tract infections: clinical and [illegible]	Tan, R.; Huang, Z.; Guo, H.; Weng, Y.; Chow, A.	2022	International Journal of Antimicrobial Agents	59	2	NA	10.1016/j.ijantimicag.2021.106511	Tan 2022	Irrelevant	Excluded by TI/AB review
3464	Streptococcal Pharyngitis Compliance With National Testing Guidelines in a Pediatric Emergency [illegible]	Thompson, J.M.; Zagel, A.L.; Spaulding, A.B.; Krause, E.A.; Arms, J.L.	2022	Pediatric Emergency Care	38	2	E519-E523	10.1097/PEC.0000000000002512	Thompson 2022	Irrelevant	Excluded by TI/AB review

S4 Table. All studies identified in the literature search

3465	Multivariable Analysis of Patient Satisfaction in the Pediatric Emergency Department	Heyming, T.W.; Donaldson, C.D.; Ehwerhemuepha, L.; Feaster, W.; Fortier, M.A.; Kain, Z.N.	2022	Pediatric Emergency Care	38	2	E544-E549	10.1097/PEC.0000000000002514	Heyming 2022	Irrelevant	Excluded by TI/AB review
3466	Evaluating adolescent substance use and suicide in the pediatric emergency department	Quimby, E.G.; Brogan, L.; Atte, T.; Diamond, G.; Fein, J.A.	2022	Pediatric Emergency Care	38	2	E595-E599	10.1097/PEC.0000000000002453	Quimby 2022	Irrelevant	Excluded by TI/AB review

S4 Table. All studies identified in the literature search

3467	An evaluation of the safety of telephone first consultations in physiotherapy MSK practice	Clarkson, C.; Gibbons, Y.; Roe, A.; Whitby, E.; Carter, H.; Williamson, A.; Yerburgh, R.; Smith, R.; Smith, B.	2022	Physiotherapy (United Kingdom)	114	(Clarkson C.; Gibbons Y.; Roe A.; Whitby E.; Carter H.;	e75	10.1016/j.physio.2021.12.012	Clarkson 2022	Irrelevant	Excluded by TI/AB review
3468	Racial and Ethnic Differences and Clinical Outcomes of Patients With Coronavirus Disease 2019 (COVID-19) Presenting to the Emergency Department	Wiley, Z.; Ross-Driscoll, K.; Wang, Z.; Smothers, L.; Mehta, A.K.; Patzer, R.E.	2022	Clinical Infectious Diseases	74	3	387-394	10.1093/cid/ciab290	Wiley 2022	Irrelevant	Excluded by TI/AB review
3469	Effectiveness of an algorithm-based care pathway for patients with non-valvular atrial fibrillation presenting to the emergency department	Masica, A.; Brown, R.; Farzad, A.; Garrett, J.S.; Wheelan, K.; Nguyen, H.L.; Ogola, G.O.; Kudyakov, R.; McDonald, B.; Boyd, B.; Patel, A.; Delaughter, C.	2022	JACEP Open	3	1	NA	10.1002/emp2.12608	Masica 2022	Irrelevant	Excluded by TI/AB review

S4 Table. All studies identified in the literature search

3470	A pilot mixed-methods randomized controlled trial of verbal versus electronic screening for adverse social determinants of health	Macias-Konstantopoulos, W.; Ciccolo, G.; Muzikansky, A.; Samuels-Kalow, M.	2022	JACEP Open	3	1	NA	10.1002/emp2.12678	Macias-Konstantopoulos 2022	Irrelevant	Excluded by TI/AB review
3471	An interdisciplinary program for familiar faces with chronic pain visiting the emergency department,Äîrandomized controlled trial	Shergill, Y.; Poulin, P.; Rice, D.; Rash, J.A.; Hebert, G.; Tennant, E.; Khoo, E.-L.; Romanow, H.; Singer, L.; Jarvis, V.; Nathan, H.; Smyth, C.	2022	JACEP Open	3	1	NA	10.1002/emp2.12628	Shergill 2022	Irrelevant	Excluded by TI/AB review
3472	Evaluating culturally and linguistically integrated care for Latinx adults with mental and substance use disorders	Walter, A.W.; Morocho, C.; Chassler, D.; Sousa, J.; De Jes√∫s, D.; Longworth-Reed, L.; Stewart, E.; Guzman, M.; Sostre, J.; Linsenmeyer, A.; Lundgren, L.	2022	Ethnicity & health	27	2	407-419	10.1080/13557858.2019.1685653	Walter 2022	Irrelevant	Excluded by TI/AB review

S4 Table. All studies identified in the literature search

3473	Patient-Physician Racial Concordance Associated with Improved Healthcare Use and Lower Healthcare [illegible]	Jetty, A.; Jabbarpour, Y.; Pollack, J.; Huerto, R.; Woo, S.; Petterson, S.	2022	Journal of racial and ethnic health disparities	9	1	68-81	10.1007/s40615-020-00930-4	Jetty 2022	Irrelevant	Excluded by TI/AB review
3474	Evaluating racial disparities in healthcare system utilization and caregiver burden among older adults with dementia	Pereira, C.; LaRoche, A.; Arredondo, B.; Pugh, E.; Disbrow, E.; Reekes, T.H.; Brickell, E.; Boettcher, A.; Sawyer, R.J.	2022	The Clinical neuropsychologist	36	2	353-366	10.1080/13854046.2021.1951844	Pereira 2022	Irrelevant	Excluded by TI/AB review
3475	COVID-19 vaccine hesitancy among metroatlanta healthcare worker	Kandiah, S.; Iheaku, O.; Hanna, J.; Johnson, K.; Farrque, M.; Shin, S.; Carroll, K.; Franks, N.; Wiley, Z.; Kulshreshtha, A.	2022	Journal of Investigative Medicine	70	2	676	10.1136/jim-2022-SRMC.460	Kandiah 2022	Irrelevant	Excluded by TI/AB review

S4 Table. All studies identified in the literature search

3476	Standardizing the central line associated bloodstream infection protocol in pediatric intestinal failure patients at oklahoma children's hospital	Meenan, C.; Graham, D.; Gupta, N.; Talathi, S.	2022	Journal of Investigative Medicine	70	2	712-713	10.1136/jim-2022-SRMC.533	Meenan 2022	Irrelevant	Excluded by TI/AB review
3477	Impact of race/ethnicity and socioeconomic status on emergency department visits in patients with hepatocellular [illegible]	Lin, D.; Tan, A.; Kilgore, K.M.; Teigland, C.; Hernandez, S.; Slay, D.L.; Schroeder, A.L.; Kim, S.	2022	Journal of Clinical Oncology	40	4 SUPPL	NA	10.1200/JCO.2022.40.4-suppl.385	Lin 2022	Irrelevant	Excluded by TI/AB review

S4 Table. All studies identified in the literature search

3478	Differences in glaucoma perception and medication adherence among patients of African and European descent	Poleon, S.; Abu, S.; Thomas, T.; Racette, L.	2022	International Journal of Clinical Pharmacy	44	1	279	10.1007/s11096-021-01373-5	Poleon 2022	Irrelevant	Excluded by TI/AB review
3479	Implementation of the TRAIN Initiative and Its Effect on Sentinel Injury Evaluation	Wickens, M.C.; Heyming, T.; Knudsen-Robbins, C.; Lara, B.; Wong, D.; Schomberg, J.	2022	Pediatrics	149	(Wickens M.C.; Heyming T.; Knudsen-Robbins C.; Lara B.; Wong D.; Schomberg	NA	NA	Wickens 2022	Irrelevant	Excluded by TI/AB review

S4 Table. All studies identified in the literature search

3480	Improving Language Access for Patients with Limited English Proficiency (LEP) During Emergency Room Care	Slater, G.N.; Forero Molina, M.A.; Marshall, M.K.; McCormick, A.	2022	Pediatrics	149	(Slater G.N.; Forero Molina M.A.; Marshall M.K.; McCormick A.)	NA	NA	Slater 2022	Irrelevant	Excluded by TI/AB review
3481	Geographic Patterns of Mental Health Utilization	Winckler, B.; Nguyen, M.B.; Khare, M.; Patel, A.; Crandal, B.R.; Jenkins, W.; Fisher, E.S.; Rhee, K.E.	2022	Pediatrics	149	(Winckler B.; Nguyen M.B.; Khare M.; Patel A.; Crand	NA	NA	Winckler 2022	Irrelevant	Excluded by TI/AB review

S4 Table. All studies identified in the literature search

3482	Identifying and Understanding Patients Utilizing the Emergency Department with Gastrostomy Issues	Rodriguez, V.; Shanefield, S.; Nettle, R.; Islam, S.	2022	Pediatrics	149	(Rodriguez V.; Shanefield S.; Nettle R.; [illegible]	NA	NA	Rodriguez 2022	Irrelevant	Excluded by TI/AB review
3483	Pediatric Disaster Triage Emodule for Evaluating and Improving Pediatric Triage Decisions	Ren, D.; Simpson, J.N.; Goodwin, T.	2022	Pediatrics	149	(Ren D.; Simpson J.N.; Goodwin T.)	NA	NA	Ren 2022	Irrelevant	Excluded by TI/AB review

S4 Table. All studies identified in the literature search

3484	Racial and Ethnic Disparitiesin Physical Restraint Use for Pediatric Patients in the Emergency Department	Tolliver, D.G.; Nash, K.; Taylor, R.; Calhoun, A.; Auerbach, M.; Mateo, C.; Grossman, M.; Venkatesh, A.; [illegible]	2022	Pediatrics	149	(Tolliver D.G.; Nash K.; Taylor R.; [illegible]	NA	NA	Tolliver 2022	Irrelevant	Excluded by TI/AB review
3485	Disruptions in Care for Medicare Beneficiaries with Severe Mental Illness during the COVID-19 Pandemic	Busch, A.B.; Huskamp, H.A.; Raja, P.; Rose, S.; Mehrotra, A.	2022	JAMA Network Open	5	1	E2145677	10.1001/jamanetworkopen.2021.45677	Busch 2022	Irrelevant	Excluded by TI/AB review
3486	First Nations status and emergency department triage scores in Alberta: a retrospective cohort study	McLane, P.; Barnabe, C.; Mackey, L.; Bill, L.; Rittenbach, K.; Holroyd, B.R.; Bird, A.; Healy, B.; Janvier, K.; Louis, E.; Rosychuk, R.J.	2022	CMAJ. Canadian Medical Association Journal	194	2	E37-E45	10.1503/cmaj.210779	McLane 2022	Irrelevant	Excluded by TI/AB review

S4 Table. All studies identified in the literature search

3487	Opioid-related emergency department visits and hospitalizations among patients with chronic gastrointestinal symptoms and disorders dually enrolled in the Department of Veterans Affairs and Medicare Part D	Balbale, S.N.; Cao, L.; Trivedi, I.; Stulberg, J.J.; Suda, K.J.; Gellad, W.F.; Evans, C.T.; Jordan, N.; Keefer, L.A.; Lambert, B.L.	2022	American Journal of Health-System Pharmacy	79	2	78-93	10.1093/ajhp/zxab363	Balbale 2022	Irrelevant	Excluded by TI/AB review
3488	Comparison of outcomes from COVID infection in pediatric and adult patients before and after the emergence of [illegible]	Wang, L.; Berger, N.A.; Kaelber, D.C.; Davis, P.B.; Volkow, N.D.; Xu, R.	2022	medRxiv	NA	(Wang L.; Xu R., rxx@case.edu) [illegible]	NA	10.1101/2021.12.30.21268495	Wang 2022	Irrelevant	Excluded by TI/AB review

S4 Table. All studies identified in the literature search

3489	Creation and validation of a citywide pediatric asthma registry for	Shelef, D.Q.; Badolato, G.M.; Badh, R.; Owotomo, O.; Kachroo, N.;	2022	Journal of Asthma	59	5	901-909	10.1080/02770903.2021.1895213	Shelef 2022	Irrelevant	Excluded by TI/AB review
3491	Noninvasive ventilation use in pediatric status asthmaticus	Usala, C.; Wilson, P.	2022	Journal of Asthma	59	7	1338-1342	10.1080/02770903.2021.1941085	Usala 2022	Irrelevant	Excluded by TI/AB review
3492	Determinants of racial and ethnic disparities in utilization of hospital-based care for asthma among Medi-Cal children in Los Angeles	Kim, Y.; Pirritano, M.; Parrish, K.M.	2022	Journal of Asthma	59	8	1521-1530	10.1080/02770903.2021.1955131	Kim 2022	Irrelevant	Excluded by TI/AB review
3493	Effects of Maryland,Äôs global budget revenue model on emergency department utilization and revisits	Galarraga, J.E.; DeLia, D.; Huang, J.; Woodcock, C.; Fairbanks, R.J.; Pines, J.M.	2022	Academic Emergency Medicine	29	1	83-94	10.1111/acem.14351	Galarraga 2022	Irrelevant	Excluded by TI/AB review

S4 Table. All studies identified in the literature search

3494	Long-term predictors of loss of asthma control in school-aged well-controlled children with mild [illegible]	Hauerslev, M.; Garpvall, K.; Marckmann, M.; Hermansen, M.N.; Hansen, K.S.; Chawes, B.L.	2022	Pediatric Pulmonology	57	1	81-89	10.1002/ppul.25710	Hauerslev 2022	Irrelevant	Excluded by TI/AB review
3495	Ten year trends in hospital encounters for pediatric asthma: an Indiana experience	Rogerson, C.; He, T.; Rowan, C.; Tu, W.; Mendonca, E.	2022	Journal of Asthma	59	12	2421-2430	10.1080/02770903.2021.2010750	Rogerson 2022	Irrelevant	Excluded by TI/AB review
3496	Improved 10-year cost savings for patients served by the Boston Children‚Äôs Hospital Community Asthma Initiative	Woods, E.R.; Sommer, S.J.; Bryson, E.A.; Shreeve, K.M.; Graham, D.; Nethersole, S.; Bhaumik, U.	2022	Journal of Asthma	59	11	2258-2266	10.1080/02770903.2021.2010746	Woods 2022	Irrelevant	Excluded by TI/AB review

S4 Table. All studies identified in the literature search

3497	Severe asthma exacerbation rates are increased among female, Black, Hispanic, and younger adult patients: results from the US CHRONICLE¬†study	Lugogo, N.; Judson, E.; Haight, E.; Trudo, F.; Chipps, B.E.; Trevor, J.; Ambrose, C.S.	2022	Journal of Asthma	59	12	2495-2508	10.1080/02770903.2021.2018701	Lugogo 2022	Irrelevant	Excluded by TI/AB review

S4 Table. All studies identified in the literature search

3499	Barriers in Managing Acute Ureteric Colic Clinical Review and Commentary	Chislett, B.; Qu, L.G.	2022	Research and Reports in Urology	14	(Chislett B., bodiechislett@gmail.com; Qu L.G.) Department of Urology, Austin Health, Heidelberg, VIC, Australia(Qu L.G.) Youn[illegible]	49-56	10.2147/RRU.S250249	Chislett 2022	Irrelevant	Excluded by TI/AB review

S4 Table. All studies identified in the literature search

3501	Potential for Use of Portable Ultrasound Devices in Rural and Remote Settings in Australia and Other Developed Countries: A Systematic Review	Shaddock, L.; Smith, T.	2022	Journal of Multidisciplinary Healthcare	15	(Shaddock L.) Medical Radiation Science, School of Health	605-625	10.2147/JMDH.S359084	Shaddock 2022	Irrelevant	Excluded by TI/AB review
3502	Pain Reduction Emergency Protocol A Prospective Study Evaluating Impact of a Nurse-initiated Protocol on PainManagement and Parental	Guiner, A.; Street, M.H.; Oke, O.; Young, V.B.; Hennes, H.	2022	Pediatric Emergency Care	38	1	E157-E164	10.1097/PEC.0000000000002193	Guiner 2022	Irrelevant	Excluded by TI/AB review

S4 Table. All studies identified in the literature search

3503	Hypoglycemia in a Pediatric Emergency Department Single-Center Experience on 402 Children	Papini, L.; Piga, S.; Dionisi-Vici, C.; Parisi, P.; Degli Atti, M.L.C.; Marcias, M.; Garrone, S.; Scialanga, B.; Taurisano, R.; Reale, A.; Villa, M.P.; Raucci, U.	2022	Pediatric Emergency Care	38	1	E404-E409	10.1097/PEC.0000000000002305	Papini 2022	Irrelevant	Excluded by TI/AB review

S4 Table. All studies identified in the literature search

3504	Portrait of the Emergency Department at Orotta National Referral and Teaching Hospital in Asmara, Eritrea: A Prospective Observational Study	Mengistu, S.T.; Achila, O.O.; Negash, S.T.; Tesfaldet, Y.; Tewolde, N.Y.; Yohannes, N.A.; Embaye, P.T.; Yemane, F.; Abraha, B.; Hagos, G.; Tewolde, Y.	2022	Open Access Emergency Medicine	14	(Mengistu S.T., tekleisam7@gmail.com) Nakfa Hospital	195-215	10.2147/OAEM.S352638	Mengistu 2022	Irrelevant	Excluded by TI/AB review
3505	Influence of the COVID-19 pandemic on pain and oral health-related quality of life in women with temporomandibular disorder	Mendon√ßa, A.K.R.; Fontoura, L.P.G.; Da Rocha, T.D.; Fontenele, R.C.; Nunes, T.N.B.; Regis, R.R.; Pinto-Fiamengui, L.M.S.	2022	Dental Press Journal of Orthodontics	27	3	NA	10.1590/2177-6709.27.3.e2220422.oar	Mendon√ßa 2022	Irrelevant	Excluded by TI/AB review
3506	Medication Adherence of Antibiotics Used in Vulnerable Population at Selected Tertiary Care Hospital in [illegible]	Kumar, A.; Panda, S.K.; Nair, D.S.; Srilakshmi, P.; Sreenivasan, S.	2022	Journal of Drug and Alcohol Research	11	6	NA	10.4303/jdar/236182	Kumar 2022	Irrelevant	Excluded by TI/AB review

S4 Table. All studies identified in the literature search

3507	Perceived racism or racial discrimination and the risk of adverse obstetric outcomes: a systematic review	Pereira, G.M.V.; Pimentel, V.M.; Surita, F.G.; Silva, A.D.; Brito, L.G.O.	2022	Sao Paulo Medical Journal	140	5	705-718	10.1590/1516-3180.2021.0505.R1.07042022	Pereira 2022	Irrelevant	Excluded by TI/AB review

S4 Table. All studies identified in the literature search

3508	Third wave COVID-19 delta variant breakthrough infection in a Hispanic-dominant suburb of Miami, Florida: ethical [illegible]	Kara, S.; Lazovic, G.; Chohan, F.; Lawrence, J.A.; Sukaina, M.; Edaki, O.; Nedd, K.	2022	Therapeutic Advances in Vaccines and Immunotherapy	10	(Kara S., Skara@larkinhospital.com) Depar [illegible]	NA	10.1177/25151355221128086	Kara 2022	Irrelevant	Excluded by TI/AB review

S4 Table. All studies identified in the literature search

3510	Care transitions intervention reduces ED revisits in cognitively impaired patients	Shah, M.N.; Jacobsohn, G.C.; Jones, C.M.C.; Green, R.K.; Caprio, T.V.; Cochran, A.L.; Cushman, J.T.; Lohmeier, M.; Kind, A.J.H.	2022	Alzheimer's and Dementia: Translational Research and Clinical Interventions	8	1	NA	10.1002/trc2.12261	Shah 2022	Irrelevant	Excluded by TI/AB review

S4 Table. All studies identified in the literature search

3511	Delayed diagnosis of serious paediatric conditions in 13 regional emergency departments	Michelson, K.A.; McGarghan, F.L.E.; Patterson, E.E.; Samuels-Kalow, M.E.; Waltzman, M.L.; Greco, K.F.	2022	BMJ Quality and Safety	NA	(Michelson K.A., kenneth.michelson@childrens.harvard.edu; McGarghan F.L.E.; Patterson E.E.; Waltzman M.L.) Division of Emergency Medicine [illegible]	NA	10.1136/bmjqs-2022-015314	Michelson 2022	Irrelevant	Excluded by TI/AB review

S4 Table. All studies identified in the literature search

3512	Association between delays to patient admission from the emergency department and all-cause 30-day mortality	Jones, S.; Moulton, C.; Swift, S.; Molyneux, P.; Black, S.; Mason, N.; Oakley, R.; Mann, C.	2022	Emergency Medicine Journal	NA	(Jones S.) Department of Population Health [illegible]	NA	10.1136/emermed-2021-211572	Jones 2022	Irrelevant	Excluded by TI/AB review
3513	Impacts of the Pandemic on Social Determinants of Health in an Academic Emergency Department	Findlay, S.; Okoro, U.; Lee, S.; Harland, K.; Evers, M.; Gaffney, E.; McCormick, M.; Buresh, C.	2022	Western Journal of Emergency Medicine	23	6	811-816	10.5811/westjem.2022.8.56145	Findlay 2022	Irrelevant	Excluded by TI/AB review
3514	Racial and Socioeconomic Disparities in Patients With Meningioma: A Retrospective Cohort Study	Hadley, C.C.; Khan, A.B.; Gadot, R.; Bayley, J.C.; Shetty, A.; Mandel, J.; Jalali, A.; Gallagher, K.K.; Sweeney, A.D.; Harmanci, A.O.; [illegible]	2022	Neurosurgery	90	1	114-123	10.1227/NEU.0000000000001751	Hadley 2022	Irrelevant	Excluded by TI/AB review

S4 Table. All studies identified in the literature search

3515	A quality improvement project to improve medication administration times in paediatric status epilepticus	Dunphy, M.; O'Brien, R.; McSweeney, N.; O'Mahony, O.	2022	Developmental Medicine and Child Neurology	64	SUPPL 1	7	10.1111/dmcn.15122	Dunphy 2022	Irrelevant	Excluded by TI/AB review
3516	Emergency medicine: Diversity in discipline,	Halsey-Nichols, M.; Ibrahim, A.; Querin, L.	2022	Western Journal of Emergency Medicine	23	1.1	S52	NA	Halsey-Nichols 2022	Irrelevant	Excluded by TI/AB review
3517	Raising bias awareness in students	Carey, J.; Mackey, C.; Tran, M.	2022	Western Journal of Emergency Medicine	23	1.1	S29-S30	NA	Carey 2022	Irrelevant	Excluded by TI/AB review

S4 Table. All studies identified in the literature search

3518	Alcohol Use Disorder is Associated with Increased Post-Discharge Emergency Department Use in Older Trauma Patients	Nakamoto, M.; Ishii, M.; Kaplan, S.; Bentov, I.; Reed, M.J.; Pham, T.	2022	Epidemiology	70	SUPPL 1	S287	10.1111/jgs.v70.S1	Nakamoto 2022	Irrelevant	Excluded by TI/AB review
3519	Lower Health Care Utilization and Costs in Community-Dwelling Low-Income Seniors Enrolled in the GRACE (Geriatric Resources for [illegible]	Kannan, A.; Wen, A.; Ibrao, M.; Caldwell, J.; Borrell, V.; Okamoto, G.; Masaki, K.	2022	Epidemiology	70	SUPPL 1	S117	10.1111/jgs.17755	Kannan 2022	Irrelevant	Excluded by TI/AB review
3520	Healthcare Utilization in Home-Based vs. Clinic-Based Primary Care: A Matched Difference-in-Difference [illegible]	Yefimova, M.; Lestoquoy, A.; Garvert, D.; Kling, S.; Winget, M.; Sheffrin, M.; Martin, M.	2022	Epidemiology	70	SUPPL 1	S158	10.1111/jgs.17755	Yefimova 2022	Irrelevant	Excluded by TI/AB review

S4 Table. All studies identified in the literature search

3521	Closing the Gap: A Systematic Review of Enhanced Recovery After Surgery Protocols in Cleft Palate Repair	Asadourian, P.; Lu Wang, M.; Demetres, M.; Otterburn, D.	2022	Cleft Palate-Craniofacial Journal	59	4 SUPPL	90-91	10.1177/10556656221079212	Asadourian 2022	Irrelevant	Excluded by TI/AB review

S4 Table. All studies identified in the literature search

3522	Access and Utilization of Asthma Medications Among Patients Who Receive Care in Federally Qualified Health Centers	Ali, A.M.; Gaglioti, A.H.; Stone, R.H.; Crawford, N.D.; Dobbin, K.K.; Guglani, L.; Young, H.N.	2022	Journal of primary care & community health	13	(Ali A.M.) University of Wisconsin-Madison, WI, Madison, United States (Gaglioti A.H.) Morehouse School of Medicine, Atlant[illegible]	21501319221101202	10.1177/21501319221101202	Ali 2022	Irrelevant	Excluded by TI/AB review

S4 Table. All studies identified in the literature search

3523	Tranq dope opioid overdose: Clinical outcomes for emergency department patients with illicit opioid overdose adulterated with xylazine	Love, J.; Levine, M.; Aldy, K.; Brent, J.; Krotulski, A.; Logan, B.; Vargas-Torres, C.; Wax, P.; Manini, A.	2022	Clinical Toxicology	60	(Love J., jennifer.love@mountsinai.org; Vargas-Torres C.; Manini A.) Icahn School of	7	10.1080/15563650.2022.2107776	Love 2022	Irrelevant	Excluded by TI/AB review
3524	Financial toxicity in patients with advanced solid malignancies participating in earlyphase clinical trials	Blanter, J.; Werner, M.; Kier, M.W.; Hapanowicz, O.; Itani, M.; Ahmad, M.; DeMerchant, M.; Paul Eder, J.; Galsky, M.D.; Hammad, A.; King, P.; Lachowicz, M.; Lucas, N.; [illegible]	2022	Journal of Clinical Oncology	40	28	267	10.1200/JCO.2022.40.28_suppl.267	Blanter 2022	Irrelevant	Excluded by TI/AB review

S4 Table. All studies identified in the literature search

3525	Exploring cost and utilization outcomes of Huntsman at Home: Which patients benefit most from a novel oncology hospital at home program?	ONeil, B.; Coombs, L.A.; Haaland, B.; Ying, J.; McPherson, J.P.; Kirchhoff, A.C.; Ulrich, C.; Huber, J.S.; Beck, A.C.; Mooney, K.	2022	Journal of Clinical Oncology	40	28	15	10.1200/JCO.2022.40.28_suppl.15	ONeil 2022	Irrelevant	Excluded by TI/AB review
3526	Human-centered design to improve clinical decision support systems (CDSS) to engage in serious illness communication (SIC) with patients with cancer in a gastrointestinal oncology clinic	Ganta, T.; Lehrman, S.; Durkovic, I.; Royer, J.; Tsembelis, B.; Liu, M.; Freeman, R.; Kia, A.; Parchure, P.; Keyzner, A.; Jain, M.; Mazumdar, M.; Pintova, S.; Bhardwaj, A.S.; Smith, C.B.	2022	Journal of Clinical Oncology	40	28	433	10.1200/JCO.2022.40.28_suppl.433	Ganta 2022	Irrelevant	Excluded by TI/AB review
3527	Patient (pt) experience with a smartphone application (app) and biosensor for	Ramsey, S.D.; Shankaran, V.; Bansal, A.; Yu, K.; Glascock, M.; Kreizenbeck, K.L.; [illegible]	2022	Journal of Clinical Oncology	40	28	443	10.1200/JCO.2022.40.28_suppl.443	Ramsey 2022	Irrelevant	Excluded by TI/AB review

S4 Table. All studies identified in the literature search

3528	Acute care utilization at the end of life: Does race/ethnicity matter?	Rodriguez, G.M.; Rosas, L.G.; Patel, M.I.	2022	Journal of Clinical Oncology	40	28	189	10.1200/JCO.2022.40.28_suppl.189	Rodriguez 2022	Irrelevant	Excluded by TI/AB review
3529	Association between In-Person vs Telehealth Follow-up and Rates of Repeated Hospital Visits [illegible]	Shah, V.V.; Villaflores, C.W.; Chuong, L.H.; Leuchter, R.K.; Kilaru, A.S.; Vangala, S.; [illegible]	2022	JAMA Network Open	NA	(Shah V.V.) Department of Emerg	E2237783	10.1001/jamanetworkopen.2022.37783	Shah 2022	Irrelevant	Excluded by TI/AB review

S4 Table. All studies identified in the literature search

3531	Bridging the gap: Development of an experiential learning-based health disparities curriculum	Caretta-Weyer, HA; Hess, JM	2022	AEM EDUCATION AND TRAINING	6	6	NA	10.1002/aet2.10820	Caretta-Weyer 2022	Irrelevant	Excluded by TI/AB review
3532	Disparities in Reperfusion Therapy and Time Delays Among Patients With Ischemic Stroke and a History of [illegible]	Mackenhauer, J; Christensen, EF; Andersen, G; Mainz, J; Johnsen, SP	2022	STROKE	53	11	3375-3385	10.1161/STROKEAHA.122.038591	Mackenhauer 2022	Irrelevant	Excluded by TI/AB review
3533	Success of prehospital tracheal intubation during cardiac arrest varies based [illegible]	Shekhar, AC; Effiong, A; Mann, NC; Blumen, IJ	2022	TRENDS IN ANAESTHESIA AND CRITICAL	45	NA	42-45	10.1016/j.tacc.2022.05.006	Shekhar 2022	Irrelevant	Excluded by TI/AB review
3534	Equitable and Timely Care of Febrile Neonates A Cross-Sectional Study	Nash, KA; Kimia, A; Fleegler, EW; Guedj, R	2021	PEDIATRIC EMERGENCY CARE	37	12	E1351-E1357	10.1097/PEC.0000000000002034	Nash 2021	Irrelevant	Excluded by TI/AB review

S4 Table. All studies identified in the literature search

3537	Human centered design workshops as a meta-solution to diagnostic disparities	Wiegand, AA; Dukhanin, V; Sheikh, T; Zannath, F; Jajodia, A; Schrandt, S; Haskell, H; McDonald, KM	2022	DIAGNOSIS	9	4	458-467	10.1515/dx-2022-0025	Wiegand 2022	Irrelevant	Excluded by TI/AB review
3541	Health care utilization among women of reproductive age living in public housing: Associations across six public housing sites in San Francisco	Headen, IE; Dubbin, L; Canchola, AJ; Kersten, E; Yen, IH	2022	PREVENTIVE MEDICINE REPORTS	27	NA	NA	10.1016/j.pmedr.2022.101797	Headen 2022	Irrelevant	Excluded by TI/AB review
3542	Racial/Ethnic Differences in Pediatric Emergency Department Wait Times	Pediat Emergency Care Appl Res Net; Johnson, TJ; Goyal, MK; Lorch, SA; Chamberlain, JM; Bajaj, L; Alessandrini, EA; Simmons, T; Casper, TC; Olsen, CS; [illegible]	2022	PEDIATRIC EMERGENCY CARE	38	2	E929-E935	10.1097/PEC.0000000000002483	PediatEmergencyCareApplResNet 2022	Irrelevant	Excluded by TI/AB review

S4 Table. All studies identified in the literature search

3544	Factors that contribute to disparities in time to acute leukemia diagnosis in young people: an in depth qualitative interview study	Ding, L; Szymczak, JE; Evans, E; Canepa, E; Martin, AE; Contractor, F; Aplenc, R; Joseph, G; Winestone, LE	2022	BMC CANCER	22	1	NA	10.1186/s12885-022-09547-8	Ding 2022	Irrelevant	Excluded by TI/AB review
3547	A Qualitative Analysis to Understand	Poon, IO; Skelton, F; Bean, LR; Guinn, D; Jemerson, T; Mbue,	2022	PHARMACY	10	1	NA	10.3390/pharmacy10010014	Poon 2022	Irrelevant	Excluded by TI/AB review
3549	Racism in healthcare: a scoping review	Hamed, S; Bradby, H; Ahlberg, BM; Thapar-Bj√∂rkert, S	2022	BMC PUBLIC HEALTH	22	1	NA	10.1186/s12889-022-13122-y	Hamed 2022	Irrelevant	Excluded by TI/AB review
3550	Health Effects of Policing in Hospitals: a Narrative Review	Gallen, K; Sonnenberg, J; Loughran, C; Smith, MJ; Sheppard, M; Schuster, K; Kaufman, E; Song, JS; Hall, EC	2023	JOURNAL OF RACIAL AND ETHNIC HEALTH DISPARITIES	10	2	870-882	10.1007/s40615-022-01275-w	Gallen 2023	Irrelevant	Excluded by TI/AB review

S4 Table. All studies identified in the literature search

3552	US Older Adults with Multiple Chronic Conditions Perceptions of Provider-Patient [illegible]	Davis-Ajami, ML; Lu, ZQK; Wu, J	2023	JOURNAL OF GENERAL INTERNAL MEDICINE	38	6	1459-1467	10.1007/s11606-022-07899-2	Davis-Ajami 2023	Irrelevant	Excluded by TI/AB review
3554	Disparities in Pediatric Mental and Behavioral Health Conditions	Hoffmann, JA; Alegr√≠a, M; Alvarez, K; Anosike, A; Shah, PP; Simon, KM; Lee, LK	2022	PEDIATRICS	150	4	NA	10.1542/peds.2022-058227	Hoffmann 2022	Irrelevant	Excluded by TI/AB review
3555	Asthma and the social determinants of health	Grant, T; Croce, E; Matsui, EC	2022	ANNALS OF ALLERGY ASTHMA & IMMUNO[illegible]	128	1	11-May	10.1016/j.anai.2021.10.002	Grant 2022	Irrelevant	Excluded by TI/AB review
3556	Implicit Racial Bias in Pediatric Orthopaedic Surgery	Guzek, R; Goodbody, CM; Jia, L; Sabatini, CS; Sankar, WN; Williams, BA; Shah, AS	2022	JOURNAL OF PEDIATRIC ORTHOPAEDICS	42	7	393-399	10.1097/BPO.0000000000002170	Guzek 2022	Irrelevant	Excluded by TI/AB review

S4 Table. All studies identified in the literature search

3559	Racial discrimination and health: health professionals' actions in providing care women in the induced abortion process	Ferreira, AP; Cardoso, GCP; Nichele, CDT; Girianelli, VR; Silva, AB; Godinho, MR	2021	CIENCIA & SAUDE COLETIVA	26	10	4623-4633	10.1590/1413-812320212610.10532021	Ferreira 2021	Irrelevant	Excluded by TI/AB review
3560	Integrated Care Pathways for Black Persons With Traumatic Brain Injury: A Critical Transdisciplinary Scoping Review of the Clinical Care Journey	Omar, S; Nixon, S; Colantonio, A	2023	TRAUMA VIOLENCE & ABUSE	24	3	1254-1281	10.1177/15248380211062221	Omar 2023	Irrelevant	Excluded by TI/AB review
3562	Impact of Native Language, English Proficiency, and Language Concordance on Interpersonal Care During Evaluation of Acute Coronary Syndrome	Edelman, DS; Palmer, DM; Romero, EK; Chang, BP; Kronish, IM	2023	JOURNAL OF GENERAL INTERNAL MEDICINE	38	4	946-953	10.1007/s11606-022-07794-w	Edelman 2023	Irrelevant	Excluded by TI/AB review

S4 Table. All studies identified in the literature search

3564	Hispanic Farmers Experience Shorter EMS Response Times but Longer Emergency Department Length of Stay [illegible]	Young, CM; Panjwani, S; Druar, N; Cahan, MA	2022	WORLD JOURNAL OF SURGERY	46	12	2872-2881	10.1007/s00268-022-06729-0	Young 2022	Irrelevant	Excluded by TI/AB review
3565	Disparities in Health Care Spending and Utilization Among Black and White Medicaid Enrollees	Wallace, J; Lollo, A; Duchowny, KA; Lavallee, M; Ndumele, CD	2022	JAMA HEALTH FORUM	3	6	NA	10.1001/jamahealthforum.2022.1398	Wallace 2022	Irrelevant	Excluded by TI/AB review
3567	Racial disparities in the management of emergency department patients presenting [illegible]	Khatri, UG; Delgado, MK; South, E; Friedman, A	2022	ANNALS OF EPIDEMIOLOGY	69	NA	16-Sep	10.1016/j.annepidem.2022.02.003	Khatri 2022	Irrelevant	Excluded by TI/AB review

S4 Table. All studies identified in the literature search

3569	Racial disparities in observers' attention to and estimations of others' pain	Kissi, A; Van Ryckeghem, DML; Mende-Siedlecki, P; Hirsh, A; Vervoort, T	2022	PAIN	163	4	745-752	10.1097/j.pain.0000000000002419	Kissi 2022	Irrelevant	Excluded by TI/AB review
3573	Reliever-Triggered Inhaled Glucocorticoid in Black and Latinx Adults with Asthma	Israel, E; Cardet, JC; Carroll, JK; Fuhlbrigge, AL; She, L; Rockhold, FW; Maher, NE; Fagan, M; Forth, VE; Yawn, [illegible]	2022	NEW ENGLAND JOURNAL OF MEDICINE	386	16	1505-1518	10.1056/NEJMoa2118813	Israel 2022	Irrelevant	Excluded by TI/AB review

S4 Table. All studies identified in the literature search

3575	Racial Disparities in Outcomes Following Open Treatment of Pediatric Femoral Shaft Fractures	Malyavko, A; Quan, TD; Kraft, D; Mathur, A; Ramamurti, P; Tabaie, S	2022	CUREUS JOURNAL OF MEDICAL SCIENCE	14	12	NA	10.7759/cureus.33149	Malyavko 2022	Irrelevant	Excluded by TI/AB review
3584	Emergency Medical Services Prehospital Response to the COVID-19 Pandemic in the US: A Brief Literature Review	Ventura, CAI; Denton, EE; David, JA; Schoenfelder, BJ; Mela, L; Lumia, RP; Rudi, RB; Haldar, B	2022	OPEN ACCESS EMERGENCY MEDICINE	14	NA	249-272	10.2147/OAEM.S366006	Ventura 2022	Irrelevant	Excluded by TI/AB review
3586	Decreased Rate of Presentation, but Worsened Racial-Ethnic Disparity in Acute Gastrointestinal Bleeding During Coronavirus 2019 Shutdown: A Retrospective Cohort Study	Reddy, S; Patel, B; Baldelli, L; Majithia, RT; Dougherty, MK	2022	CLINICAL AND EXPERIMENTAL GASTROENTEROLOGY	15	NA	67-77	10.2147/CEG.S348574	Reddy 2022	Irrelevant	Excluded by TI/AB review

S4 Table. All studies identified in the literature search

3589	The relationship between stroke system organization and disparities in access to stroke center care in California	Zachrison, KS; Samuels-Kalow, ME; Li, SJ; Yan, ZY; Reeves, MJ; Hsia, RY; Schwamm, LH; Camargo, CA	2022	JOURNAL OF THE AMERICAN COLLEGE OF EMERGENCY [illegible]	3	2	NA	10.1002/emp2.12706	Zachrison 2022	Irrelevant	Excluded by TI/AB review
3590	Unequal Housing Conditions And Code Enforcement Contribute To Asthma Disparities In Boston, Massachusetts	Lemire, E; Samuels, EA; Wang, WY; Haber, A	2022	HEALTH AFFAIRS	41	4	563-572	10.1377/hlthaff.2021.01403	Lemire 2022	Irrelevant	Excluded by TI/AB review
3591	Factors influencing the initiation of adjuvant endocrine therapy in patients with estrogen receptor-positive [illegible]	Levy, J; Farag, F; Cole, J	2022	BREAST CANCER RESEARCH AND TREATMENT	196	1	207-213	10.1007/s10549-022-06735-9	Levy 2022	Irrelevant	Excluded by TI/AB review

S4 Table. All studies identified in the literature search

3592	Social determinants and inequitable maternal and perinatal outcomes in Aotearoa New [illegible]	Dawson, P; Auvray, B; Jaye, C; Gauld, R; Hay-Smith, J	2022	WOMENS HEALTH	18	NA	NA	10.1177/17455065221075913	Dawson 2022	Irrelevant	Excluded by TI/AB review
3593	Patient Ethnicity and Pediatric Visits to the Emergency Department for Fever	Shapiro, DJ; Fine, AM	2021	PEDIATRIC EMERGENCY CARE	37	11	555-559	10.1097/PEC.0000000000001945	Shapiro 2021	Irrelevant	Excluded by TI/AB review
3596	Air Quality Index and Emergency Department Visits and Hospitalizations for Childhood Asthma	Rosser, F; Han, YY; Rothenberger, SD; Forno, E; Mair, C; Celed√≥n, JC	2022	ANNALS OF THE AMERICAN THORACIC SOCIETY	19	7	1139-1148	10.1513/AnnalsATS.202105-539OC	Rosser 2022	Irrelevant	Excluded by TI/AB review

S4 Table. All studies identified in the literature search

3597	Block by block: Building on our knowledge to better care for LGBTQIA plus patients	Driver, L; Egan, DJ; Hsiang, E; Lall, MD; Moll, J; Ritchie, AM; Sonn, BJ; Totten, VY; Williams, DB; McGregor, AJ	2022	AEM EDUCATION AND TRAINING	6	NA	S57-S63	10.1002/aet2.10755	Driver 2022	Irrelevant	Excluded by TI/AB review

S4 Table. All studies identified in the literature search

3600	A Video-Based Consent Tool: Development and Effect of Risk-Benefit Framing on Intention to Randomize	Writing Grp CODA Collaborative; Lois, A; Kohler, JE; Monsell, SE; Pullar, KM; Victory, J; Odom, SR; Fischkoff, K; Kaji, AH; Evans, HL; Sohn, V; Kao, LS; [illegible]	2023	JOURNAL OF SURGICAL RESEARCH	283	NA	357-367	10.1016/j.jss.2022.10.089	WritingGrpCODACollaborative 2023	Irrelevant	Excluded by TI/AB review
3602	Repeat hospital transfers among long stay nursing home residents: a mixed methods analysis of age, race, code status and clinical complexity	Vogelsmeier, A; Popejoy, L; Fritz, E; Canada, K; Ge, B; Brandt, L; Rantz, M	2022	BMC HEALTH SERVICES RESEARCH	22	1	NA	10.1186/s12913-022-08036-9	Vogelsmeier 2022	Irrelevant	Excluded by TI/AB review
3603	Association of Individual and Familial History of Correctional Control With Health Outcomes of Patients in a Primary Care Center	MacKenzie, O; Goldman, J; Chin, M; Duffy, B; Martino, S; Ramsey, S; Jim√©nez, MC; Vanjani, R	2021	JAMA NETWORK OPEN	4	11	NA	10.1001/jamanetworkopen.2021.33384	MacKenzie 2021	Irrelevant	Excluded by TI/AB review

S4 Table. All studies identified in the literature search

3604	Socioeconomic and Psychosocial Predictors of Magnetic Resonance Imaging After Cervical and Thoracic Spine [illegible]	Hagan, MJ; Pertsch, NJ; Leary, OP; Xi, K; Zheng, BY; Camara-Quintana, JQ; Niu, TY; Sullivan, PZ; Abinader, JF; Telfeian, AE; [illegible]	2022	WORLD NEUROSURGERY	161	NA	E757-E766	10.1016/j.wneu.2022.02.093	Hagan 2022	Irrelevant	Excluded by TI/AB review
3606	Social Risk Interventions and Health Care Utilization for Pediatric Asthma A Systematic Review and Meta-analysis	Tyris, J; Keller, S; Parikh, K	2022	JAMA PEDIATRICS	176	2	NA	10.1001/jamapediatrics.2021.5103	Tyris 2022	Irrelevant	Excluded by TI/AB review
3607	Access to quality health resources and environmental toxins affect the relationship [illegible]	Adise, S.; Marshall, A. T.; Kan, E.; Sowell, E. R.	2022	Front Public Health	10	NA	1061049	10.3389/fpubh.2022.1061049	Adise 2022	Irrelevant	Excluded by TI/AB review
3612	Contributions of Key Components of a Medical Home on Child Health Outcomes	Arthur, N. S. M.; Blewett, L. A.	2023	Matern Child Health J	27	3	476-486	10.1007/s10995-022-03539-7	Arthur 2023	Irrelevant	Excluded by TI/AB review

S4 Table. All studies identified in the literature search

3617	Bias in Care: Impact of Ethnicity on Time to Emergent Surgery Varies Between Subspecialties	Blumenthal, S. R.; Fryhofer, G. W.; Serra-Lopez, V.; Pierrie, S. N.; Mehta, S.	2023	J Am Acad Orthop Surg Glob Res Rev	7	6	NA	10.5435/JAAOSGlobal-D-23-00060	Blumenthal 2023	Irrelevant	Excluded by TI/AB review
3622	Centering patient perspectives to achieve injury-related health equity in trauma care systems: Improving trauma [illegible]	Conrick, K. M.; Mills, B.; St Vil, C.; Dotolo, D.; Solano, E.; Bulger, E. M.; Arbabi, S.; Herrenkohl, M.; Vavilala, M. S.; [illegible]	2023	Injury	54	9	110847	10.1016/j.injury.2023.110847	Conrick 2023	Irrelevant	Excluded by TI/AB review
3631	Gaps in the Identification of Child Race and Ethnicity in a Pediatric Emergency Department	Gutman, C. K.; Lion, K. C.; Waidner, L.; Bryan, L.; Sizemore, A.; Holland, C.; Montero, C.; Fernandez, R.	2023	Western Journal of Emergency Medicine	24	3	547-551	10.5811/westjem.59145	Gutman 2023	Irrelevant	Excluded by TI/AB review

S4 Table. All studies identified in the literature search

3633	Ambulance diversion and ED destination by race/ethnicity: evaluation of Massachusetts' ambulance diversion ban	Hanchate, A. D.; Baker, W. E.; Paasche-Orlow, M. K.; Feldman, J.	2022	BMC Health Services Research	22	1	987	10.1186/s12913-022-08358-8	Hanchate 2022	Irrelevant	Excluded by TI/AB review
3636	Relationship between criminal legal system exposure and health care [illegible]	Hawks, L. C.; Walker, R. J.; Egede, L. E.	2023	Journal of the National Medical Association	115	2	244-253	10.1016/j.jnma.2023.01.015	Hawks 2023	Irrelevant	Excluded by TI/AB review
3641	Association between hospital competition and quality of prostate cancer care	Jayadevappa, R.; Malkowicz, S. B.; Vapiwala, N.; Guzzo, T. J.; Chhatre, S.	2023	BMC Health Services Research	23	1	828	10.1186/s12913-023-09851-4	Jayadevappa 2023	Irrelevant	Excluded by TI/AB review

S4 Table. All studies identified in the literature search

3644	Improving Care Linkage for Racial-Ethnic Minority Youths Receiving Emergency Department Treatment for Suicidality: SAFETY-A	Kodish, T.; Lau, A. S.; Belin, T. R.; Berk, M. S.; Asarnow, J. R.	2023	Psychiatric Services	74	4	419-422	10.1176/appi.ps.20220129	Kodish 2023	Irrelevant	Excluded by TI/AB review
3648	A Pilot of Digital Whiteboards for Improving Patient Satisfaction in the Emergency Department: Nonrandomized Controlled Trial	Marshall, A. D. A.; Hasdianda, M. A.; Miyawaki, S.; Jambaulikar, G. D.; Cao, C.; Chen, P.; Baugh, C. W.; Zhang, H.; McCabe, J.; Steinbach, L.; King, S.; Friedman, J.; Su, J.; Landman, A. B.; Chai, P. R.	2023	JMIR Form Res	7	NA	e44725	10.2196/44725	Marshall 2023	Irrelevant	Excluded by TI/AB review

S4 Table. All studies identified in the literature search

3652	Association of the Affordable Care Act Medicaid Expansion with Trauma Outcomes and Access to Rehabilitation among Young Adults: Findings Overall, by Race and Ethnicity, and Community Income Level	Metzger, G. A.; Asti, L.; Quinn, J. P.; Chisolm, D. J.; Xiang, H.; Deans, K. J.; Cooper, J. N.	2021	Journal of the American College of Surgeons	233	6	776-793.e16	10.1016/j.jamcollsurg.2021.08.694	Metzger 2021	Irrelevant	Excluded by TI/AB review
3654	Disparities in Perceived Availability of Healthful Foods, Dietary Behaviors, Diet Quality, and Obesity Among Mothers from Low-Income Households:	Molitor, F.; Kehl, S.	2023	Health Equity	7	1	235-242	10.1089/heq.2022.0127	Molitor 2023	Irrelevant	Excluded by TI/AB review

S4 Table. All studies identified in the literature search

3656	Paediatric use of emergency medical services in India: A retrospective cohort study of one million children	Newberry, J. A.; Rao, S. J.; Matheson, L.; Anurudran, A. S.; Acker, P.; Darmstadt, G. L.; Mahadevan, S. V.; Rao, G. V. R.; Strehlow, M.	2022	J Glob Health	12	NA	4080	10.7189/jogh.12.04080	Newberry 2022	Irrelevant	Excluded by TI/AB review
3658	Veteran perspectives of epilepsy care: Impact of Veteran satisfaction, knowledge, and proactivity	Panahi, S.; Kennedy, E.; Roghani, A.; VegaYon, G.; VanCott, A.; Gugger, J. J.; Raquel Lopez, M.; Jo Pugh, M.	2023	Epilepsy & Behavior	144	NA	109218	10.1016/j.yebeh.2023.109218	Panahi 2023	Irrelevant	Excluded by TI/AB review

S4 Table. All studies identified in the literature search

3662	Research Priorities for Pediatric Emergency Care to Address Disparities by Race, Ethnicity, and Language	Portillo, E. N.; Rees, C. A.; Hartford, E. A.; Foughty, Z. C.; Pickett, M. L.; Gutman, C. K.; Shihabuddin, B. S.; Fleegler, E. W.; Chumpitazi, C. E.; Johnson, T. J.; Schnadower, D.; Shaw, K. N.	2023	JAMA Netw Open	6	11	e2343791	10.1001/jamanetworkopen.2023.43791	Portillo 2023	Irrelevant	Excluded by TI/AB review
3671	Community Socioeconomic and Urban-Rural Disparities in Prehospital Notification of Stroke by Emergency Medical Services in North Carolina	Shams, R. B.; Chari, S. V.; Cui, E. R.; Fernandez, A. R.; Brice, J. H.; Winslow, J. E.; Jauch, E. C.; Patel, M. D.	2023	Southern Medical Journal	116	9	765-771	10.14423/smj.0000000000001601	Shams 2023	Irrelevant	Excluded by TI/AB review
3673	Experiences of Black Adults Evaluated in a Locked Psychiatric Emergency Unit: A Qualitative Study	Smith, C. M.; Daley, L. A.; Lea, C.; Daniel, K.; Tweedy, D. S.; Thielman, N. M.; Staplefoote-Boynton, B. L.; Aimone, E.; Gagliardi, J. P.	2023	Psychiatric Services	74	10	1063-1071	10.1176/appi.ps.20220533	Smith 2023	Irrelevant	Excluded by TI/AB review

S4 Table. All studies identified in the literature search

3675	A Retrospective Study of Disparities in an Academic Ophthalmic Emergency Department	Szypko, C.; Hall, N.; Ta, T.; Gardiner, M. F.; Lorch, A. C.	2021	J Acad Ophthalmol (2017)	13	2	e277-e287	10.1055/s-0041-1736439	Szypko 2021	Irrelevant	Excluded by TI/AB review
3681	A global perspective on suicides and suicide attempts	Paul, Elise	2021	Oxford textbook of suicidology and suicide prevention., 2nd ed.	NA	NA	93-98	NA	Paul 2021	Irrelevant	Excluded by TI/AB review
3684	Centering the lens of social justice	Borden, Eucharia; Leimena, Meagan Lyon; Sumser, Bridget	2022	The Oxford textbook of palliative social work., 2nd ed.	NA	NA	13-Mar	NA	Borden 2022	Irrelevant	Excluded by TI/AB review
3687	Children of stigmatized parents: Concerns and fostering holistic development	Gautam, Aishwarya; Bhadra, Subhasis	2022	Child safety, welfare and well-being: Issues and challenges., 2nd ed.	NA	NA	197-213	10.1007/978-981-16-9820-0_12	Gautam 2022	Irrelevant	Excluded by TI/AB review

S4 Table. All studies identified in the literature search

3689	Coordination of care with psychiatry	Bregman, Benjamin L.; Powsner, Seth	2021	Behavioral emergencies for healthcare providers., 2nd ed.	NA	NA	397-407	10.1007/978-3-030-52520-0_39	Bregman 2021	Irrelevant	Excluded by TI/AB review
3693	Data-informed decision support to improve pediatric and maternal care quality under Medicaid managed care settings	Symum, Hasan	2022	Dissertation Abstracts International: Section B: The Sciences and Engineering	83	2-B	NA	NA	Symum 2022	Irrelevant	Excluded by TI/AB review
3694	Diet quality differs by race/ethnicity among mothers and their children from supplemental nutrition assistance program,Äìeducation households	Molitor, Fred; Doerr, Celeste	2021	Health Equity	5	1	633-636	10.1089/heq.2021.0007	Molitor 2021	Irrelevant	Excluded by TI/AB review

S4 Table. All studies identified in the literature search

3695	Does participation in the community outreach for asthma care and healthy lifestyles (COACH) program alter subsequent use of hospital services for children discharged with asthma?	Pinto, Jamie M.; Navallo, Lauren J.; Petrova, Anna	2021	Journal of Asthma	58	2	231-239	10.1080/02770903.2019.1672719	Pinto 2021	Irrelevant	Excluded by TI/AB review
3700	Erectile problems in polish straight, bisexual, and gay men: Does sexual identity really matter?	Grabski, Bartosz; Kasparek, Krzysztof; Koziara, Karolina; Mijas, Magdalena	2022	Journal of Sex Research	NA	NA	NA	10.1080/00224499.2022.2074952	Grabski 2022	Irrelevant	Excluded by TI/AB review
3701	Essential knowledge and qualities	Hays, Pamela A.	2022	Addressing cultural complexities in counseling and clinical practice: An intersectional approach., 4th ed.	NA	NA	21-40	10.1037/0000277-002	Hays 2022	Irrelevant	Excluded by TI/AB review

S4 Table. All studies identified in the literature search

3707	Intersections of personal vs collective trauma during the COVID‚Äê19 pandemic: The hijacking of the human imagination	Kalsched, Donald	2021	The Journal of Analytical Psychology	66	3	443-462	10.1111/1468-5922.12697	Kalsched 2021	Irrelevant	Excluded by TI/AB review
3709	Mild traumatic brain injury: Antecedents and aftermath	Oldenburg, Christian	2022	Dissertation Abstracts International: Section B: The Sciences and Engineering	83	2-B	NA	NA	Oldenburg 2022	Irrelevant	Excluded by TI/AB review
3712	Patterns of incident dementia codes during the COVID‚Äê19 pandemic at an integrated healthcare system	Borson, Soo; Chen, Aiyu; Wang, Susan E.; Nguyen, Huong Q.	2021	Journal of the American Geriatrics Society	69	12	3389-3396	10.1111/jgs.17527	Borson 2021	Irrelevant	Excluded by TI/AB review

S4 Table. All studies identified in the literature search

3715	Practice with Arab American families	Abu-Ras, Wahiba; Azhar, Sameena	2021	Multicultural perspectives in working with families: A handbook for the helping professions., 4th ed.	NA	NA	227-249	NA	Abu-Ras 2021	Irrelevant	Excluded by TI/AB review
3716	Psychological aspects of hereditary cancer risk counseling and genetic testing: Toward an expanded and more equitable view	Aspinwall, Lisa G.; Taber, Jennifer M.; Kohlmann, Wendy; Bautista, Lilly B.	2022	Psychological aspects of cancer: A guide to emotional and psychological consequences of cancer, their causes, and their management., 2nd ed.	NA	NA	359-398	10.1007/978-3-030-85702-8_22	Aspinwall 2022	Irrelevant	Excluded by TI/AB review

S4 Table. All studies identified in the literature search

3717	Psychological distress among medically complex veterans with a recent emergency department visit	Ramos, Katherine; Shepherd-Banigan, Megan E.; Stechuchak, Karen M.; Coffman, Cynthia; Oddone, Eugene Z.; Van Houtven, Courtney; Hendrix, Cristina C.; Mahanna, Elizabeth P.; Hastings, Susan Nicole	2022	Psychological Services	19	2	353-359	10.1037/ser0000437	Ramos 2022	Irrelevant	Excluded by TI/AB review
3718	Psychosocial risk factors of erectile dysfunction among heterosexual men living with hiv	Akbas, Selin; Alc√©na-Stiner, Danielle C.; McMahon, James M.	2022	AIDS Care	NA	NA	NA	10.1080/09540121.2022.2039357	Akbas 2022	Irrelevant	Excluded by TI/AB review
3720	Racism by another name: Black students, overrepresentation, and the carceral state of special education	Hines, Dororthy E.; Boveda, Mildred; Lindo, Endia J.	2021	NA	NA	NA	NA	NA	Hines 2021	Irrelevant	Excluded by TI/AB review
3721	Re/formation and identity: The intersectionality of development, culture, and immigration	Johnson, Deborah J.; Chuang, Susan S.; Glozman, Jenny	2022	NA	NA	NA	NA	10.1007/978-3-030-86426-2	Johnson 2022	Irrelevant	Excluded by TI/AB review

S4 Table. All studies identified in the literature search

3722	'Sex and drug overdose mortality trends, 1999-2020': In reply	Friedman, Joseph; Hansen, Helena	2022	JAMA Psychiatry	79	8	834-834	10.1001/jamapsychiatry.2022.1685	Friedman 2022	Irrelevant	Excluded by TI/AB review
3723	Shared trauma, shared resilience during a pandemic: Social work in the time of COVID-19	Tosone, Carol	2021	NA	NA	NA	NA	10.1007/978-3-030-61442-3	Tosone 2021	Irrelevant	Excluded by TI/AB review
3725	Sport, physical education, and social justice: Religious, sociological, psychological, and capability perspectives	Watson, Nick J.; Jarvie, Grant; Parker, Andrew	2021	NA	NA	NA	NA	NA	Watson 2021	Irrelevant	Excluded by TI/AB review
3726	Substance abuse	White-Ryan, Linda; Heyman, Janna	2021	Multicultural perspectives in working with families: A handbook for the helping professions., 4th ed.	NA	NA	333-345	NA	White-Ryan 2021	Irrelevant	Excluded by TI/AB review

S4 Table. All studies identified in the literature search

3729	The need for community-responsive and flexible sex ed for historically marginalized youth	Caruso, Isabella; Valdez, Elizabeth Salerno; Lovell, Camille Collins; Chan, Jazmine; Beatriz, Elizabeth; Gubrium, Aline	2022	Sexuality Research & Social Policy: A Journal of the NSRC	NA	NA	NA	10.1007/s13178-022-00717-8	Caruso 2022	Irrelevant	Excluded by TI/AB review
3730	The Routledge handbook of health communication, 3rd ed	Thompson, Teresa L.; Harrington, Nancy Grant	2022	NA	NA	NA	NA	NA	Thompson 2022	Irrelevant	Excluded by TI/AB review
